# Rapid sp^3^-Enriched Scaffold Generation via a Selective Aziridine Amide Ring-Opening Reaction

**DOI:** 10.1021/acs.joc.3c02952

**Published:** 2024-02-10

**Authors:** Masahito Abe, Jeremy S. Coleman, Christopher C. Presley, Nathan D. Schley, Craig W. Lindsley

**Affiliations:** † Warren Center for Neuroscience Drug Discovery, Department of Pharmacology, Vanderbilt University, Franklin, Tennessee 37067, United States; ‡ Department of Chemistry, Vanderbilt University, Nashville, Tennessee 37235, United States; § Department of Biochemistry, Vanderbilt University, Nashville, Tennessee 37232, United States

## Abstract

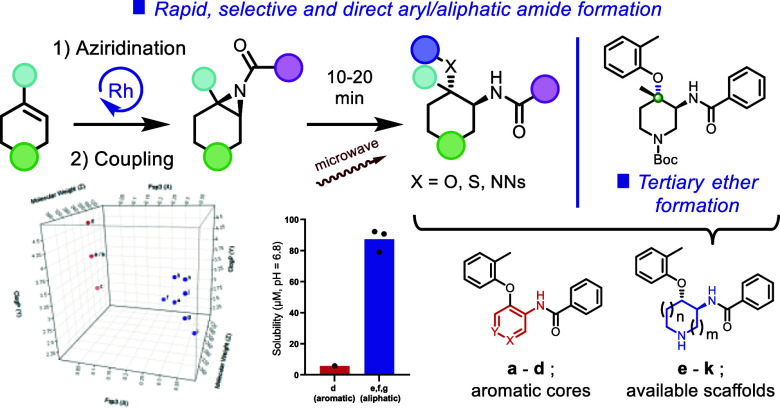

Sp^3^-enriched small molecules play a critical role in developing drug candidates. While designing analogues with greater sp^3^ character, a methodology utilizing a less explored cyclic-aziridine amide ring-opening reaction to generate sp^3^-enriched scaffolds has been developed and reported. This methodology enables rapid access to substructures with higher fsp^3^ values, attracting greater attention within the past few decades. The reaction exhibits a wide reaction scope, featuring a highly sterically hindered phenolic ether, thiophenolic ethers, protected aniline formations, and aliphatic/heteroaromatic ring-containing aziridine amides as substrates. Additionally, this reaction provides access to congested tertiary ether formations through regioselective transformation, applicable to an extensive range of drug discovery targets, construction of complex small molecules, and natural product syntheses. The scaffolds developed show improved physicochemical properties.

## Introduction

Fsp^3^, a fraction of sp^3^ carbon atoms, has a significant impact on the drug-likeness of small molecules and is well known as a verified indicator of physicochemical properties in drug discovery.^1^ A higher fsp^3^ could reduce the promiscuity of a drug candidate to increase the success rate of its clinical trial.^2^ However, the enantioselective or diastereoselective introduction of higher fsp^3^ motifs can be challenging due to their selectivity control. Meanwhile, aniline substructures are frequently encountered in pharmacologically active compounds,^3^ such as natural products and small molecule probes, since carbon–nitrogen bonds on aromatic rings are highly transformative. However, the motif has been well known as an alert structure with the potential for mutagenic character, resulting from a high tendency of bioactivation by CYP450 and reactive metabolite formation.^4^ Thus, an alternative aniline motif, including bicyclo[1.1.1]pentane (BCP), bicyclo[2.2.2]octane (BCO), and cubane (CUB), as benzene isosteres has been explored and examined for decades to achieve a higher safety profile as drug candidates.^5^

**Scheme 1:**
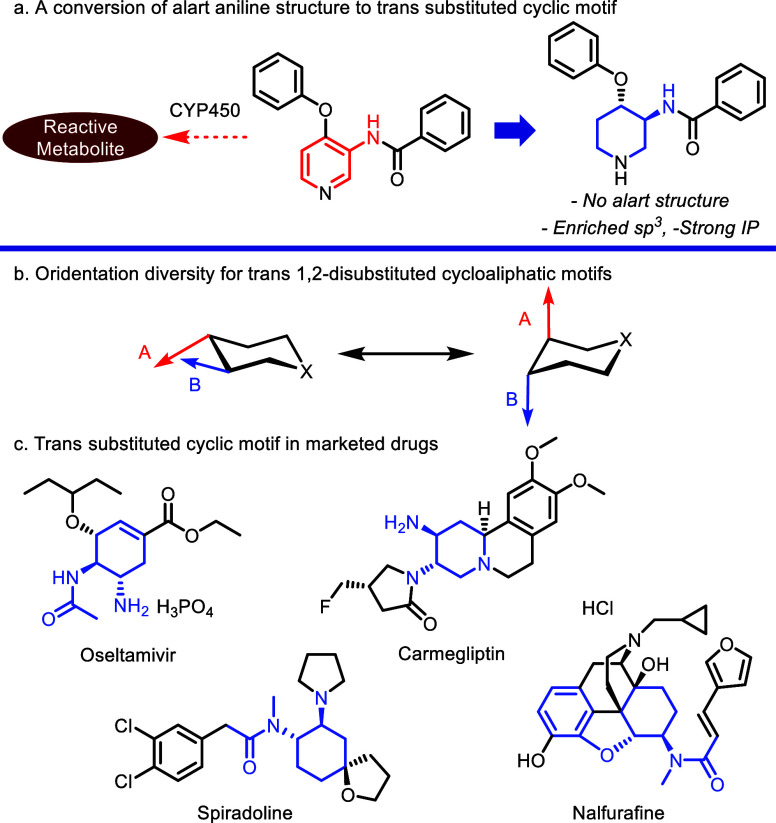
Significance of *Trans* 1,2-Disubsituted Cycloaliphatic Motifs in Drug Discovery

Case in point, a methodology for the replacement of *o*-substituted benzamides with *trans*-substituted cyclic analogues has been explored to prevent the generation of mutagenic structural motifs and to obtain enriched sp^3^ motifs (Scheme 1a). Moreover, this transformation is favorable to secure significant intellectual property. As depicted in Scheme 1b, the 1,2-*trans*-disubstituted cyclic structural motif has a diversity of dihedral angles and orientations between substituents depending on the steric bulk, ring size, and nature of the substituted functional groups. Since this motif can fill a space in a unique and tunable direction with its 3D structure, it has been an attractive scaffold in drug discovery for decades. Consequently, this motif is present in a wide range of marketed drugs, including the neuramidase inhibitor Oseltamivir,^6^ which contains a multisubstituted cyclohexane central core, Carmegliptin,^7^ a dipeptidyl peptidase 4 (DPP-4) inhibitor, and fused-piperidine core analogue, and κ-opioid receptor (KOR) agonists Spiradoline^8^ and Nalfurafine.^9^ (Scheme 1c).

**Scheme 2:**
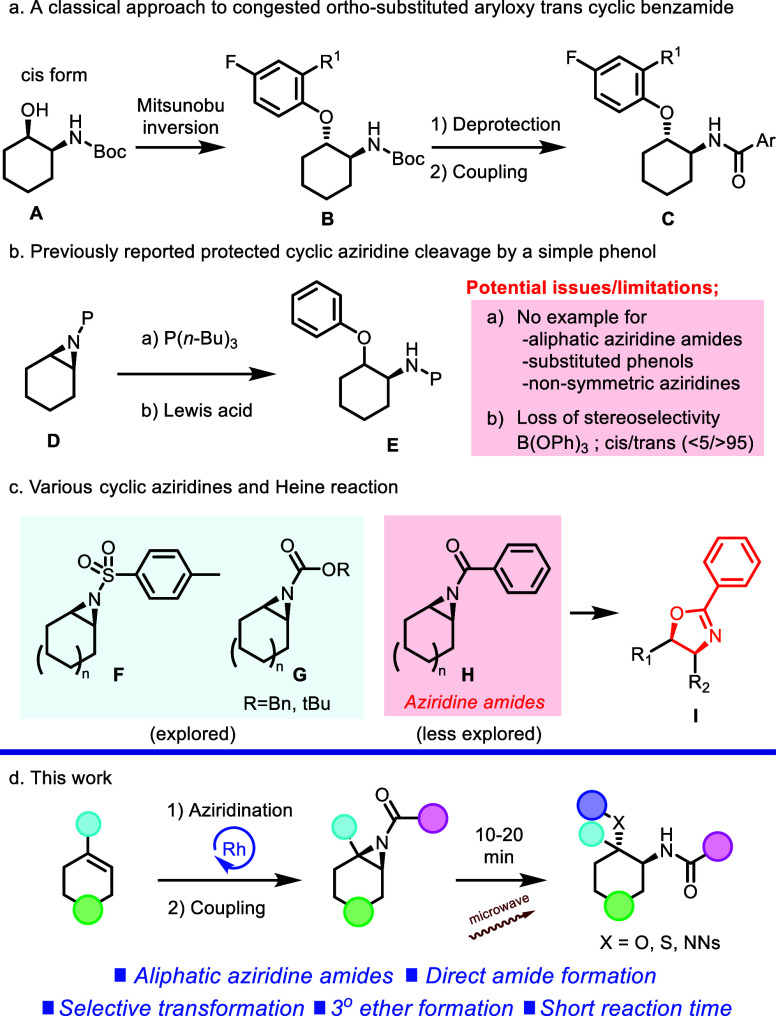
Previously Reported Methods for *Trans* 1,2-Disubsituted Cycloaliphatic Motifs and This Work

However, due to the sterically congested nature of this type of substitution, there are limited synthetic routes to afford *ortho*-substituted aryloxy *trans* cyclic benzamide (Scheme 2).

Typically, known synthetic approaches employ *N*-protected reagents and intermediates and include the use of Mitsunobu inversion of *cis*-substituted *N*-Boc protected alcohols (A), followed by deprotection of the protecting group and a coupling reaction (Scheme 2a).^10^ Additionally, a simple nonsubstituted aryloxy *trans* cyclic benzamide can be accessed via protected cyclic aziridine ring cleavage (Scheme 2).

Although several *N*-protected cyclic aziridine ring-opening reactions, which are exemplified as applications of an aliphatic phosphine reagent P(*n*-Bu)_3_,^11^ Lewis acid B(OPh)_3_,^12^ BF_3_OEt_2_,^13^ and Sn(OTf)_2_,^13^ are known in the literature,^14^ such transformations tend to be limited in scope. Specifically, reports for this type of transformation tend to be restricted to simple nucleophiles with minimal steric encumbrance and are often confined to symmetric azidirine substrates. And, under acidic conditions such as B(OPh)_3_, the reaction shows a potential issue of incomplete *cis*/*trans* diastereoselectivity as a result of its cationic mechanism.

Importantly, the reactions have been limited to aziridines protected with classical protecting groups (Boc or Cbz) and arylsulfonamide-type substitution patterns (Ts or Ns) without expanding their scope to arylamide-type substitution.^15^ This explored substrate bias comes from the higher reactivity of sulfonylated or Boc/Cbz protected substitutions due to their higher electron withdrawing properties, and its inherent nature of the aziridine benzamide substructure. As shown in Scheme 2c, it is well known that the aziridine benzamide substructure (H) tends to undergo the Heine reaction,^16^ which can be easily induced by acidic^17^ or nucleophilic^18^ conditions; therefore, the detailed studies have been hampered.

Additionally, these types of transformations typically require long reaction times and relatively harsh conditions, which often limit the reaction scope. Therefore, by utilizing basic conditions, we sought to develop a methodology for an aziridine ring-opening reaction that would address these issues and focus specifically on underexplored diverse amide-substituted aziridine substrates. Herein, we report the detailed study of an efficient sp^3^-enriched scaffold generation through a cyclic symmetrical and nonsymmetrical aziridine amide ring-opening reaction, as well as a feature of generated molecules. This reaction features: (1) a wide range of reaction scope including sterically hindered *o*-substituted nucleophiles and aliphatic/heteroaromatic ring-containing aziridine amides as substrates; (2) the selective formation of a tertiary ether; (3) direct *trans* 1,2-disubstituted amide scaffold generation without a multistep deprotection/functionalization; (4) stereoselective conversion and regioselective conversion for the nonsymmetric cyclic aziridine amide; and (5) a short reaction time (10–20 min). These goals were ultimately realized using a 2-step sequence (Rh_2_(esp)_2_^19^-catalyzed nitrene transfer reaction from cyclic olefins^20^ and an amide coupling reaction), which allows for the rapid generation of a wide range of sp^3^-enriched cyclic motifs (Scheme 2d).

## Results and Discussion

Since regioselectivity of nonsymmetric 6-membered cyclic aziridine rings with *o*-substituted phenol nucleophiles has been underexplored, we conducted an extensive reaction optimization campaign with a piperidine-based aziridine benzamide. We employed various operationally simple bases, the sterically hindered *o*-cresol as a nucleophile, and a variety of solvents while considering the p*K*_a_ value (p*K*_a_ = 10.3) of *o*-cresol (Table 1).

**Table 1:**
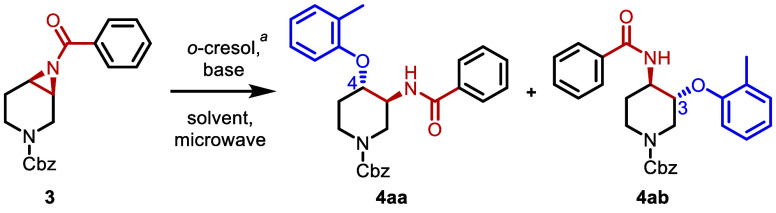
Optimization of the Reaction Conditions^a^

entry	base	solvent	temp (°C)	time (min)	**4aa** + **4ab** (%)^b^	ratio (**4aa**/**4ab**)
1	none	THF	110	20	0	-
2	Cs_2_CO_3_	THF	110	20	96	3.0/1
3	DBU	THF	110	20	3	-
4	Li_2_CO_3_	DMF	110	20	10	2.9/1
5	Na_2_CO_3_	DMF	110	20	22	4.0/1
6	K_2_CO_3_	DMF	110	20	82	4.4/1
7	Cs_2_CO_3_	DMF	110	20	85	4.0/1
8	K_2_CO_3_	CH_3_CN	110	20	29	3.9/1
9	K_2_CO_3_	acetone	110	20	22	3.8/1
10	K_2_CO_3_	toluene	110	20	0	-
11	K_2_CO_3_	DCE	110	20	0	-
12	K_2_CO_3_	DMF	80	20	43	4.9/1
13	K_2_CO_3_	DMF	140	20	88	3.7/1
14	Cs_2_CO_3_	THF	140	20	95	2.7/1
15	K_2_CO_3_	DMF	110	10	51	4.5/1
16	Cs_2_CO_3_	THF	140	10	93	2.8/1

a Reactions were performed with **3** (1.0 equiv), base (1.2 equiv), and *o*-cresol (1.2 equiv) in solvent (0.10 M) under microwave irradiation.

b Yield determined via LCMS using a calibration curve and naphthalene as an internal standard.

During the preliminary study, we tested various bases [Li_2_CO_3_, Na_2_CO_3_, K_2_CO_3_, Cs_2_CO_3_, *N*,*N*-diisopropylethylamine (DIPEA), pyridine, 1,8-diazabicyclo[5.4.0]undec-7-ene (DBU), Supporting Information, Table S1 and entry 3] in tetrahydrofuran (THF) under microwave irradiation to determine that Cs_2_CO_3_ was the most effective base, resulting in a high yield (96%) in a time-efficient manner (20 min) (entry 2). Gratifyingly, no epimerization was observed in this study. As for regioselectivity, nucleophilic addition occurred predominantly at the C4 position, which is consistent with the results observed for other nucleophiles with 6-membered piperidines.^21^ As a control experiment, a reaction without a base was conducted to show the necessity for the reaction to proceed (entry 1). In the next step, other solvent systems were explored for their effects on regioselectivity. To our pleasure, utilizing several bases with *N*,*N*-dimethylformamide (DMF) boosted regioselectivity up to 4.4/1 (entry 4–7). Other solvents [acetonitrile (CH_3_CN), acetone, toluene, and 1,2-dichloroethane (DCE)] were also tested to show that DMF was the most efficient in terms of both yield and selectivity (entry 6, 8–11). Next, optimization of reaction temperature using K_2_CO_3_/DMF was conducted to show that 110 °C was well balanced in both yield and selectivity (entry 6, 12, and 13). In a similar manner, using Cs_2_CO_3_ in THF at higher temperatures gave comparable yields but less selectivity (entry 2 and 14). Finally, a shorter 10 min reaction time favorable to rapid library generation, was performed (entry 15 and 16). The Cs_2_CO_3_/THF protocol was preferred in terms of the high yield (93%) although regioselectivity was moderate (2.4/1). As a control experiment, the previously reported phosphine conditions using P(*n*-Bu)_3_^11^ was attempted, but no product formation was observed after 10 min (Table S1). Considering the high regioselectivity and yield in an efficient reaction time for nonsymmetric aziridine formation, we selected the condition described in entry 6 as the standard condition for nonsymmetric aziridines such as **3**. For a symmetric cyclic aziridine **1** (5-membered ring; *n* = *m* = 1, 7-membered ring; *n* = *m* = 2, Scheme 2), the condition described in entry 16 was selected as a standard high yielding reaction condition since regioselectivity is unnecessary due to its symmetric plane.

**Scheme 3:**
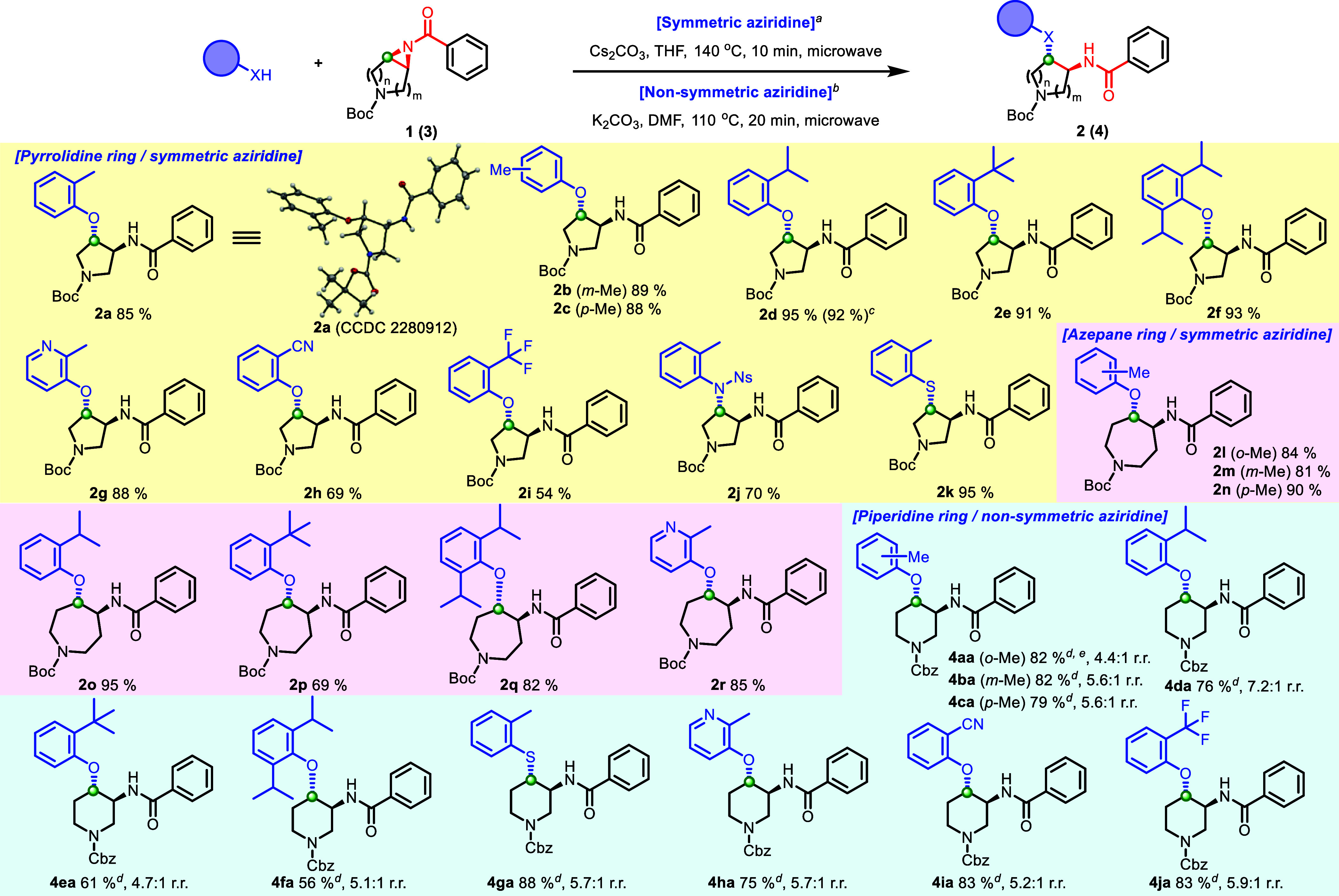
Substrate/Nucleophile Scope of a Selective Aziridine Amide Ring-Opening Reaction All reactions for symmetric aziridine amides were performed with 1 (1.0 equiv), Cs_2_CO_3_ (1.2 equiv), and *o*-substituted nucleophile (1.2 equiv) in THF (0.10 M) at 140 °C for 10 min under microwave irradiation; isolated yield. ^b^All reactions for nonsymmetric aziridine amides were performed with **3** (1.0 equiv), K_2_CO_3_ (1.2 equiv), and *o*-substituted phenol (1.2 equiv) in DMF (0.10 M) at 110 °C for 20 min under microwave irradiation; isolated yield. ^c^Isolated yield on a 1.0 mmol scale. ^d^Yield indicated as a combined amount of regio isomers. ^e^Yield determined via LCMS. ORTEP, ellipsoids are set at a 50% probability.

**Scheme 4:**
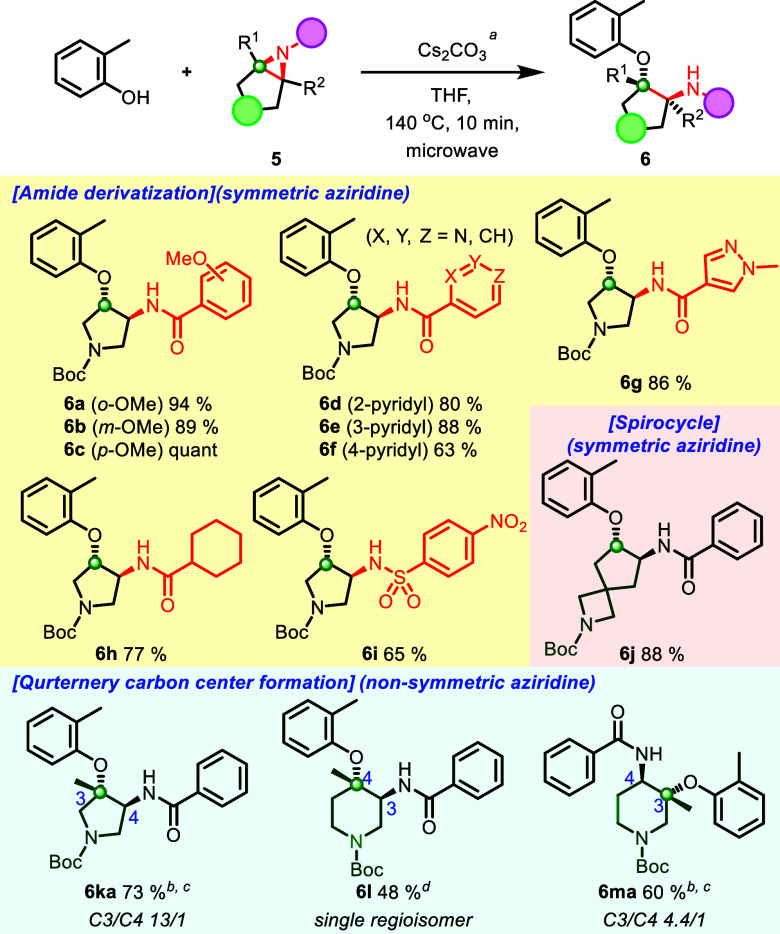
Reaction Scope of Various Amide Groups and Various Cyclic Aziridines for Aziridine Ring Opening Unless otherwise specified, all reactions were performed with **5** (1.0 equiv), Cs_2_CO_3_ (1.2 equiv), and *o*-cresol (1.2 equiv) in THF (0.10 M) at 140 °C for 10 min under microwave irradiation; isolated yield. ^b^Yield indicated as a combined amount of regio isomers. ^c^Cs_2_CO_3_ (5.0 equiv), *o*-cresol (5.0 equiv) for 40 min. ^d^Cs_2_CO_3_ (5.0 equiv), *o*-cresol (5.0 equiv) for 80 min.

With optimized conditions for cyclic aziridine amide ring openings in hand, we sought to explore the substrate and nucleophile scope for these reaction conditions (Schemes 3 and 4). As a general method for preparing aziridine amide substrates, 2-step procedures, including an aziridination reaction for the corresponding cyclic olefin and the subsequent coupling reaction with variety of carboxylic acids, were utilized successfully. This was suitable to increase accessibility for a broader range of substrates (Scheme S1). N–H free aziridination was achieved through a nitrene transfer reaction by employing Du Bois’ catalyst Rh_2_(esp)_2_,^19^ which conditions were originally reported by Kürti et al.^20^ The amide formation was conducted through a 1-[bis(dimethylamino)methylene]-1*H*-1,2,3-triazolo[4,5-*b*]pyridinium 3-oxid hexafluorophosphate (HATU) coupling or acylation with acid anhydride. For symmetric 5-membered pyrrolidine aziridine amides, a broad range of nucleophiles could be applied to give *trans* substituted pyrrolidine analogues (Scheme 3). Importantly, substituted pyrrolidines are one of the lead structures in many drugs and drug-like small molecules,^22^ where the motif is ranked among the top five common nitrogen-containing heterocycles in a survey of FDA-approved drugs.^23^ Gratifyingly, the *o*-, *m*-, and *p*-Me substituted cresols gave the desired products in high yields (**2a**–**2c**, 85–88%). The *o*-Me-substituted phenolic ether **2a** was confirmed by X-ray analysis to demonstrate clear relative *trans* stereochemistry (CCDC; 2280912). In addition, further sterically hindered *o*-substituted phenols, including di-*o*-substituted phenols, provided an excellent yield (**2d**–**2f**). These results highlight the utility of the methodology since this sterically congested phenolic ether formation can be challenging from coupling between a corresponding sterically hindered alcohol and an electron-rich arylhalide or arylboronic acid. On a 1.0 mmol scale, the ring-opening reaction provided *o*-*i*Pr-substituted phenolic ether (**2d**) in a comparable yield (92%) compared to the 89.2 μmol scale reaction (95%). In a similar manner, an electron-deficient pyridinol and phenols gave the desired ring-opened products (**2g**–**2i**). In addition to phenols, 4-nitrobenzenesulfonyl (4-Ns) protected aniline, and an *o*-substituted thiophenol provided the desired products in high yields (**2j** and **2k**), which exemplifies a broad range of nucleophile scopes. Additionally, a resulting 4-Ns group in **2j** could be deprotected orthogonal to the Boc group to afford **2j′** (89%), which demonstrates the possibility for increased functionalization of the scaffold (Supporting Information). The substrate scope of the aziridine amide ring-opening reaction could be expanded to a symmetric 7-membered azepane aziridine amide. In a similar manner, all nucleophiles, including sterically hindered phenols, electron-deficient phenols, and a pyridinol, afforded the corresponding *trans*-substituted azepane analogues in good yields (**2l**–**2r**, 69–95%).

As for nonsymmetric piperidine aziridine amides (**3**), the optimal condition, which was identified in Table 1, entry 6, could also be applied in excellent yields while maintaining regioselectivity (4.4–7.1:1) to afford *trans* substituted piperidine analogues (Scheme 3). In addition to a pyrrolidine ring, a piperidine ring is one of the lead scaffolds in drug discovery development. The piperidine ring was the most prevalent nitrogen ring system and also found in over 70 unique small-molecule drugs in FDA-approved drugs.^23^ The utilization of cresols and sterically hindered phenols maintained regioselectivity with good yields (**4aa**–**4fa**, 56–82%, 4.4–7.2:1) Notably from these analogues, an *o*-*i*Pr substitution demonstrated greater regioselectivity than other substituents (7.2:1). In addition, the *o*-thiocresol gave the ring-opened product in an excellent yield with high regioselectivity (**4ga**, 88%, 5.7:1) as similar results were observed for an electron-deficient pyridinol and phenols (**4ha**–**4ja**, 75–83%, 5.2–5.9:1).

Encouraged by these results, we further explored the reaction scope regarding various amide groups and cyclic aziridines, including congested trisubstituted patterns (Scheme 4). A pyrrolidine aziridine amide, with an electron-rich benzamide and electron-deficient pyridine amide, showed robust reactivity to afford ring-opened products (**6a**–**6f**, 63%-quant). Pleasingly, a 5-membered heteroaromatic ring and an aliphatic aziridine amide also generated the desired product in high yields (**6g**, 85%, **6h**, 77%), which features a novel and broad substrate scope. In addition, a 4-Ns functionalized aziridine and a Boc-protected azetidine spirocycle bearing aziridine amides produced ring-opened products (**6i**, 65%, and **6j**, 88%). The 4-Ns protecting group can be further deprotected in the presence of the Boc group, and spiroazetidine ring products allow for further functionalization with high fsp^3^ fragments.

Lastly, we explored aziridine amides that include trisubstituted cyclic aziridine amides (**5k**–**5m**). Interestingly, the reaction for them afforded the tertiary ether in moderate to good yield in a selective manner (**6ka**–**6ma**, 4.4:1 to single isomer, 48–73%). Notably, these findings are the first reported examples of tertiary ether formation in cyclic aziridine amide ring-opening reactions, and their utility could be helpful for constructing sterically congested structural motifs.^24^ Meanwhile, the 6-membered lactam aziridine amide substrate (**5n** in Supporting Information) did not provide any ring-opening product. This may be due to the acidity of the α-proton, which can result in aziridine ring decomposition via β-elimination. Limitations with nucleophiles were observed as reactions with an aliphatic alcohol, and aniline nucleophiles did not undergo the ring-opening reaction due to lack of nucleophile acidity.

**Scheme 5:**
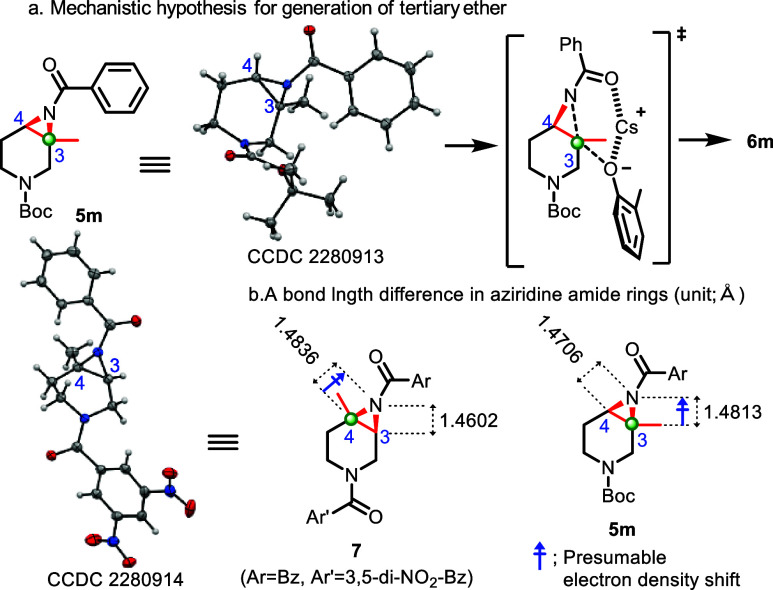
Mechanistic Hypothesis for the Regioselectivity

As a mechanistic hypothesis for the ring-opening reaction, including tertiary ether formation, it is hypothesized that a nucleophilic SN_2_ substitution via a 6-membered transition state occurs predominantly or exclusively at the more substituted site in the aziridine amide ring (Scheme 5a), considering the resulting stereochemical relationships (**6ka**–**6ma**) in Scheme 4. To investigate the regioselectivity, each bond length within the aziridine amide ring was measured by X-ray crystallographic analysis (**5m**; CCDC 2280913, **7**; CCDC 2280914). As depicted in Scheme 5b, the C4–N bond in **7** was longer (1.4836 Å) than the C3–N bond (1.4602 Å), while a slight difference was observed in **5m** (C3–N bond; 1.4813 Å vs C4–N bond; 1.4706 Å). This means that a longer bond is weaker and more reactive, which is consistent with its regioselectivity for tertiary ether formation for **6l** (corresponding **7**) and **6ma** (corresponding **5m**). The longer bond length at the more substituted carbon may be due to the electron-donating property of alkyl groups, suggesting that more alkyl substitutions in the aziridine ring would donate electrons at the more substituted carbon and induce an electron density shift from a more substituted carbon to the nitrogen atom of the aziridine (Scheme 5b, C4–N bond in **7** and C3–N bond in **5m**). Consequently, a more substituted carbon would increase the C–N bond length and its reactivity for nucleophiles. Second, this bond length difference could also be understood from the standpoint of a stereoelectronic effect. Presumably, an overlapping of the filled σ_C–H_ orbital of the alkyl group or Me group at the more substituted carbon with the empty σ*_C–N_ orbital of the aziridine ring can contribute to stabilization of the longer bond length.^24^ Third, the longer bond in the aziridine ring could be caused by steric repulsion between the Me group of the substituted site and the aziridine N-Bz moiety. In the analysis of X-ray crystal structures of **5m** and **7**, the Me group at the aziridine ring is closely located with the N(aziridine)–C(C=O of benzoyl group) moiety, generating steric repulsion.

**Figure 1:**
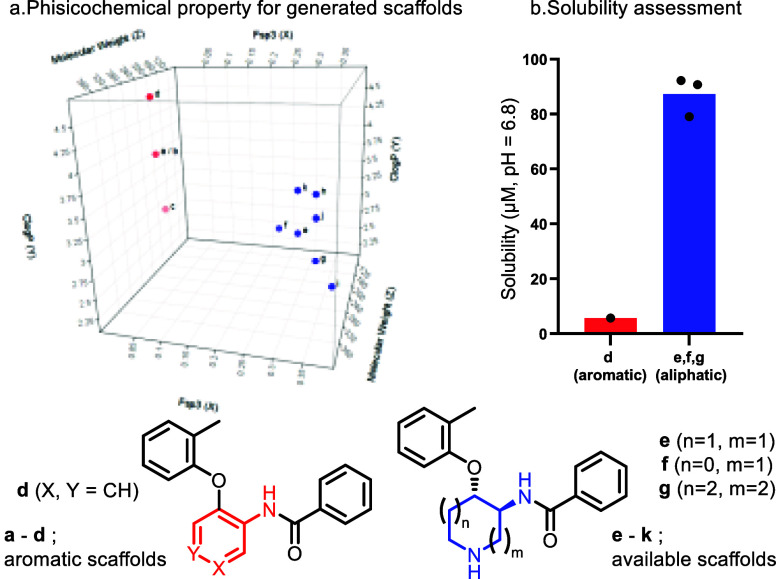
Evaluation of generated scaffolds.

Finally, we compared the calculated physicochemical properties between newly generated aliphatic scaffolds (Figure 1a, **e**–**k**) and corresponding aromatic core analogues as a reference (Figure 1a, **a**–**d**). The calculation for the generated aliphatic scaffolds was performed for the Boc deprotected form (Figure 1a, **a**–**d**, Supporting Information, Table S6). As displayed in Figure 1a, fsp^3^ of the aliphatic central core analogues is significantly increased (**e**–**k**; 0.334 vs **a**–**d**; 0.053) compared to the aromatic central core analogues. Notably, the aliphatic central core scaffolds maintain a sufficient molecular weight (average; **e**–**k**; 318 vs **a**–**d**; 301) and exhibit slightly lower clog *P* (**e**–**k**; 3.23 vs **a**–**d**; 3.97). In addition to this calculation, kinetic solubility for both selected generated aliphatic scaffolds (**e**–**g**) and corresponding aromatic analogue (**d**) was assessed. As shown in Figure 1b, the available 1,2-*trans* disubstituted cyclic scaffolds showed significantly improved kinetic solubility at neutral pH (**e**; a pyrrolidine ring, 79.07 μM, **f**; a piperidine ring, 90.67 μM, **g**; an azepane ring; 92.17 μM) compared to aromatic core analogues (**d**; 5.74 μM). The increased fsp^3^ and the additional amine functionality would contribute to increase their solubility significantly. These results highlight that this methodology is practical for obtaining higher fsp^3^ scaffolds with improved kinetic solubility but also avoids the production of a mutagenic alert structure to improve the potential tox profile.

## Conclusions

In summary, we have described the rapid generation of sp^3^-enriched scaffolds by developing a novel selective cyclic aziridine amide ring-opening reaction with high regioselectivity. This methodology is highlighted by a wide reaction scope range, which encompasses the formation of a sterically hindered phenolic ether, aliphatic and heteroaromatic rings containing aziridine amides as substrates, and the regioselective formation of a tertiary ether. Additionally, the direct generation of *trans*-substituted amide scaffolds can be obtained without the use of a protecting group for aziridine functionalization. Further, this methodology allows for a stereoselective and regioselective conversion of aziridine amides, is operationally straightforward, and can be performed in a short reaction time. Efforts to understand the extensive regioselective reaction mechanics associated with this novel methodology, expand the current reaction scope, and further applications in drug discovery and natural product synthesis are underway in our laboratory and will be reported in due course.

## Experimental Section

### General Information

Unless otherwise noted, all reagents were purchased from commercial sources and used without further purification. Low-resolution mass spectra were observed on a Waters QDa (Performance) SQ MS with an ESI source. Samples were introduced via an Acquity I-Class PLUS UPLC comprised of a BSM, FL-SM, CH-A, and PDA. UV absorption was generally observed at 215 and 254 nm; 4 nm bandwidth. All NMR spectra were measured on a 400 MHz Bruker AV-400 instrument. ^1^H chemical shifts are reported as δ values in ppm relative to the residual solvent peak (CDCl_3_ = 7.26, CD_3_OD = 3.31, (CD_3_)_2_SO = 2.50). Since most of the *trans* 1,2-disubstituted cyclic products obtained appeared as a mixture of rotamers in the NMR spectra at rt, NMR experiments at 343 K for these compounds were performed to coalesce the signals, which is indicated in parentheses where appropriate. Data are reported as follows: chemical shift, multiplicity (br = broad, s = singlet, d = doublet, t = triplet, q = quartet, dd = doublet of doublets, ddd = doublet of doublet of doublets, td = triplet of doublets, m = multiplet), coupling constant, and integration. ^13^C chemical shifts are reported as δ values in ppm relative to the residual solvent peak (CDCl_3_ = 77.16, CD_3_OD = 49.0, (CD_3_)_2_SO = 39.52). High-resolution mass spectra were observed on an Agilent 6540 UHD Q-TOF with an ESI source. Automated normal phase flash column chromatography was conducted on a Biotage Isolera One or a Teledyne ISCO CombiFlash system. Reverse-Phase HPLC was conducted on a Gilson preparative reversed-phase HPLC system. Microwave synthesis was performed in a Biotage Initiator^+^ microwave synthesis reactor. The used power range for maintaining 140 °C in THF was 133–143 W, and the range for 110 °C in DMF was 56–66 W from magnetron at 2.45 GHz.

### General Procedure A: Aziridination

To a solution of a cyclic olefin (2.10 mmol, 1.0 equiv) in 1,1,1,3,3,3-hexafluoroisopropanol (HFIP, 5.25 mL, 49.8 mmol, 0.042M), pyridine (408 μL, 5.05 mmol, 2.4 equiv), hydroxylamine-*o*-sulfonic acid (571.0 mg, 5.05 mmol, 2.4 equiv), and Rh_2_(esp)_2_ (16.0 mg, 0.021 mmol, 1 mol %) were added at 25 °C. The mixture was stirred at rt for 16 h. To this mixture, sat. aq. NaHCO_3_ (10.0 mL) was added, and the mixture was extracted with DCM (3 × 10.0 mL) and concentrated. The crude product was purified by column chromatography [0–20% MeOH/DIPEA (99/1) in DCM] to give the corresponding aziridine.

### General Procedure B: Coupling. (B-(a)) via HATU Coupling

An aziridine amine (5.43 mmol, 1.0 equiv), benzoic acid (862 mg, 7.06 mmol, 1.3 equiv), HATU (2683 mg, 7.06 mmol, 1.3 equiv), and DIPEA (2.363 mL, 13.6 mmol, 2.5 equiv) were dissolved in DCM (10.9 mL, 0.10 M) and stirred at rt overnight. To this mixture, sat. aq. NaHCO_3_ (10.0 mL) was added, and the mixture was extracted with DCM (3 × 10.0 mL) and concentrated. This crude material was purified by column chromatography (0–100% EtOAc in hexane) to give the corresponding aziridine amide.

### (B-(b)) via Acid Anhydride Coupling

To a mixture of an aziridine amine (1.18 mmol, 1.0 equiv) and triethylamine (427 μL, 3.07 mmol, 2.6 equiv) in DCM (11.8 mL, 0.10 M) at 0 °C, benzoic anhydride (640 mg, 2.83 mmol, 2.4 equiv) was added. The mixture was stirred at 0 °C for 30 min. To this mixture, sat. aq. NaHCO_3_ (10.0 mL) was added, and the mixture was extracted with DCM (3 × 10.0 mL) and concentrated. This crude material was purified by column chromatography (0–100% EtOAc in hexane) to give the corresponding aziridine amide.

### General Procedure C: Ring-Opening Reaction. (C-(a)) High Yielding Condition

All reactions were performed in a sealed vial. To a solution of an aziridine amide (89.2 μmol, 1.0 equiv) in THF (892 μL, 0.10 M) was added Cs_2_CO_3_ (35.1 mg, 107 μmol, 1.2 equiv) followed by nucleophiles (107 μmol, 1.2 equiv). The resulting mixture was stirred at 140 °C for 10 min under microwave irradiation. After cooling to rt, sat. aq. NaHCO_3_ (1.0 mL) was added, and the mixture was extracted with DCM (3 × 1.0 mL) and concentrated. The crude residue was purified by column chromatography or RP-HPLC to give the *trans* substituted cyclic amide.

### (C-(b)) Selective Condition

All reactions were performed in a sealed vial. To a solution of an aziridine amide (89.2 μmol, 1.0 equiv) in DMF (892 μL, 0.10 M) was added K_2_CO_3_ (15.0 mg, 107 μmol, 1.2 equiv) followed by nucleophiles (107 μmol, 1.2 equiv). The resulting mixture was stirred at 110 °C for 20 min under microwave irradiation. After cooling to rt, sat. aq NaHCO_3_ (1.0 mL) was added, and the mixture was extracted with DCM (3 × 1.0 mL) and concentrated. The crude residue was purified by column chromatography or RP-HPLC to give the *trans* substituted cyclic amide.

### Crystal Structures

Deposition Numbers 2280912 (for compound **2a**), 2280913 (for compound **5m**), and 2280914 (for compound **7**) contain the supplementary crystallographic data for this paper. These data are provided free of charge by the joint Cambridge Crystallographic Data Centre and Fachinformationszentrum Karlsruhe Access Structures service.

### Portion of the Data is Presented below; for a More Comprehensive Data Set, Kindly Refer to the Supporting Information (SI)

#### *tert*-Butyl 6-methyl-3,7-diazabicyclo[4.1.0]heptane-3-carboxylate (**S2b**)

Followed General Procedure A with *tert*-butyl 4-methyl-3,6-dihydropyridine-1(2*H*)-carboxylate (415 mg, 2.10 mmol) to give *tert*-butyl 6-methyl-3,7-diazabicyclo[4.1.0]heptane-3-carboxylate (288 mg, 1.36 mmol, 64%) as a colorless oil. ^1^H NMR (400 MHz, DMSO-*d*_6_, 343 K): δ 3.70 (dd, *J* = 13.9, 4.5 Hz, 1H), 3.38–3.27 (m, 2H), 3.01–2.89 (m, 1H), 2.02 (d, *J* = 4.5 Hz, 1H), 1.74–1.65 (m, 1H), 1.63–1.53 (m, 1H), 1.39 (s, 9H), 1.23 (s, 3H). ^13^C{^1^H} NMR (101 MHz, DMSO-*d*_6_, 343 K): δ 153.8, 78.1, 42.4, 38.0, 34.6, 32.7, 29.5, 27.8, 24.4. HRMS (TOF, ES+) C_11_H_21_N_2_O_2_ [M + H]^+^ calcd mass 213.1598, found 213.1603.

#### *tert*-Butyl 6-benzoyl-3,6-diazabicyclo[3.1.0]hexane-3-carboxylate (**1a**)

Followed General Procedure B-(a) with *tert*-butyl 3,6-diazabicyclo[3.1.0]hexane-3-carboxylate (1.000 g, 5.43 mmol) and benzoic acid to give *tert*-butyl 6-benzoyl-3,6-diazabicyclo[3.1.0]hexane-3-carboxylate (1.085 g, 3.77 mmol, 69%) as a white solid after purification by column chromatography (0–100% EtOAc in hexane). ^1^H NMR (400 MHz, DMSO-*d*_6_, 298 K): δ 7.89–7.85 (m, 2H), 7.66–7.59 (m, 1H), 7.55–7.48 (m, 2H), 3.70 (d, *J* = 12.2 Hz, 1H), 3.63 (d, *J* = 12.1 Hz, 1H), 3.53–3.48 (m, 2H), 3.25 (dd, *J* = 12.1, 1.9 Hz, 1H), 3.20 (dd, *J* = 12.2, 2.0 Hz, 1H), and 1.29 (s, 9H). ^13^C{^1^H} NMR (101 MHz, DMSO-*d*_6_, 298 K): δ 174.8, 153.3, 132.9, 132.7, 128.6, 128.3, 78.8, 45.3, 44.8, 40.9, 40.1, 28.0. HRMS (TOF, ES+) C_16_H_20_N_2_NaO_3_ [M + Na]^+^ Calcd mass 311.1366, found 311.1373.

#### *tert*-Butyl 7-benzoyl-6-methyl-3,7-diazabicyclo[4.1.0]heptane-3-carboxylate (**5l**)

Followed General Procedure B-(b) with *tert*-butyl 6-methyl-3,7-diazabicyclo[4.1.0]heptane-3-carboxylate (250 mg, 1.18 mmol) to give *tert*-butyl 7-benzoyl-6-methyl-3,7-diazabicyclo[4.1.0]heptane-3-carboxylate (268 mg, 0.847 mmol, 72%) as a colorless oil after purification by column chromatography (0–100% EtOAc in hexane). ^1^H NMR (400 MHz, DMSO-*d*_6_, 343 K): δ 7.88–7.82 (m, 2H), 7.65–7.58 (m, 1H), 7.56–7.49 (m, 2H), 3.85 (dd, *J* = 14.3, 4.1 Hz, 1H), 3.65 (d, *J* = 14.3 Hz, 1H), 3.51–3.40 (m, 1H), 3.26–3.15 (m, 1H), 2.83 (d, *J* = 3.9 Hz, 1H), 2.16 (dt, *J* = 14.4, 4.8 Hz, 1H), 1.84–1.73 (m, 1H), 1.40 (s, 9H), 1.06 (s, 3H). ^13^C{^1^H} NMR (101 MHz, DMSO-*d*_6_, 343 K): δ 176.9, 154.1, 134.3, 132.5, 128.5, 128.2, 78.8, 43.8, 41.7, 39.3, 38.4, 28.8, 28.0, 21.0. HRMS (TOF, ES+) C_18_H_25_N_2_O_3_ [M + H]^+^ calcd mass 317.1860, found 317.1869.

#### *tert*-Butyl (3*SR*,4*SR*)-3-benzamido-4-(*o*-tolyloxy)pyrrolidine-1-carboxylate (**2a**)

Followed General Procedure C-(a) with *tert*-butyl 6-benzoyl-3,6-diazabicyclo[3.1.0]hexane-3-carboxylate (25 mg, 86.7 μmol) and *o*-cresol to give *tert*-butyl (3*S*,4*S*)-3-benzamido-4-(2-methylphenoxy)pyrrolidine-1-carboxylate (29.1 mg, 85%) as a white solid after purification by column chromatography (0–100% EtOAc in hexane). ^1^H NMR (400 MHz, DMSO-*d*_6_, 343 K, δ) 8.55 (d, *J* = 6.4 Hz, 1H), 7.91–7.83 (m, 2H) 7.58–7.50 (m, 1H), 7.50–7.42 (m, 2H), 7.23–7.11 (m, 3H), 6.88 (td, *J* = 6.9, 1.8 Hz, 1H), 4.87 (dt, *J* = 4.5, 2.1 Hz, 1H), 4.59–4.49 (m, 1H), 3.79–3.68 (m, 2H), 3.52–3.40 (m, 2H), 2.14 (s, 3H), 1.43 (s, 9H). ^13^C{^1^H} NMR (101 MHz, DMSO-*d*_6_, 343 K): δ 166.7, 154.7, 153.5, 133.9, 131.0, 130.4, 127.8, 127.2, 126.6, 126.5, 120.8, 113.3, 78.4, 27.9, 15.4. (δ 78.7, 53.4, 49.1, 48.9 are additional peaks observed in HSQC, but not clear in 1D-^13^C{^1^H} NMR.) HRMS (TOF, ES+) C_23_H_28_N_2_NaO_4_ [M + Na]^+^ calcd mass 419.1941, found 419.1939.

#### Benzyl (3*SR*,4*SR*)-3-benzamido-4-(*m*-tolyloxy)piperidine-1-carboxylate (**4ba**)

Followed General procedure C-(b) with benzyl 7-benzoyl-3,7-diazabicyclo[4.1.0]heptane-3-carboxylate (30.0 mg, 89.2 μmol) and *m*-cresol to give benzyl (3*SR*,4*SR*)-3-benzamido-4-(*m*-tolyloxy)piperidine-1-carboxylate as the main product [32.5 mg, 81%, Regio isomer ratio = 5.6:1 (determined by LCMS)]. Purification was conducted by RP-HPLC (55–95% MeCN in 0.05% aqueous NH_4_OH). Isolated as a white solid. ^1^H NMR (400 MHz, MeOD, 298 K): δ 7.67–7.60 (m, 2H), 7.53–7.44 (m, 1H), 7.42–7.25 (m, 7H), 7.10 (t, *J* = 7.5 Hz, 1H), 6.84–6.77 (m, 2H), 6.73 (d, *J* = 7.5 Hz, 1H), 5.16–5.11 (m, 2H), 4.58–4.50 (m, 1H), 4.22 (td, *J* = 8.3, 4.4 Hz, 1H), 4.08 (dd, *J* = 13.6, 3.9 Hz, 1H), 3.99–3.89 (m, 1H), 3.30–3.22 (m, 2H), 2.26–2.16 (m, 4H), 1.73–1.59 (m, 1H). ^13^C{^1^H} NMR (101 MHz, MeOD, 298 K): δ 170.6, 159.2, 157.0, 140.8, 138.0, 135.6, 132.7, 130.3, 129.6, 129.4, 129.1, 128.9, 128.4, 123.3, 118.3, 114.4, 76.5, 68.5, 51.8, 46.6, 42.4, 30.3, 21.5. HRMS (TOF, ES+) C_27_H_29_N_2_O_4_ [M + H]^+^ calcd mass 445.2122, found 445.2122.

#### Benzyl (3*RS*,4*RS*)-4-benzamido-3-(*m*-tolyloxy)piperidine-1-carboxylate (**4bb**)

Isolated as a white solid. ^1^H NMR (400 MHz, DMSO-*d*_6_, 343 K): δ 8.22 (d, *J* = 8.0 Hz, 1H), 7.79–7.70 (m, 2H), 7.52–7.46 (m, 1H), 7.45–7.39 (m, 2H), 7.38–7.29 (m, 5H), 7.10 (t, *J* = 7.8 Hz, 1H), 6.81–6.70 (m, 3H), 5.15 (d, *J* = 12.6 Hz, 1H), 5.09 (d, *J* = 12.6 Hz, 1H), 4.39–4.32 (m, 1H), 4.31–4.21 (m, 1H), 4.16 (dd, *J* = 13.4, 2.7 Hz, 1H), 3.96–3.86 (m, 1H), 3.26–3.15 (m, 1H), 3.13–3.09 (m, 1H), 2.21 (s, 3H), 2.01–1.92 (m, 1H), 1.72–1.60 (m, 1H). ^13^C{^1^H} NMR (101 MHz, DMSO-*d*_6_, 343 K): δ 166.3, 157.6, 154.6, 138.9, 136.8, 134.8, 130.9, 129.1, 128.3, 128.0, 127.7, 127.3, 127.1, 121.9, 116.7, 112.9, 74.1, 66.4, 50.2, 45.7, 41.9, 29.2, 20.8. HRMS (TOF, ES+) C_27_H_29_N_2_O_4_ [M + H]^+^ calcd mass 445.2122, found 445.2123.

## Data Availability

The data underlying this study are available in the published article and its Supporting Information.

## Supporting Information Available

Experimental procedures, characterization data, X-ray crystallographic data and detailed physicochemical property data (PDF)
