# Supplementary material for: Rapid sp3-Enriched Scaffold Generation via a Selective Aziridine Amide Ring-Opening Reaction
Source: J Org Chem. 2024 Feb 10;89(5):3500–8. doi: 10.1021/acs.joc.3c02952 (PMC10913065; doi:10.1021/acs.joc.3c02952)
Supplement: Supplementary file 1 — jo3c02952_si_001.pdf [file jo3c02952_si_001.pdf]

Supporting Information

Rapid sp^3 enriched scaffold generation via a selective aziridine amide ring opening reaction

Masahito Abe[a]*, Jeremy S. Coleman[a], Christopher C. Presley[a], Nathan D. Schley[b] and Craig W. Lindsley[a, b, c]

[a]Warren Center for Neuroscience Drug Discovery, Department of Pharmacology, Vanderbilt University, Franklin, Tennessee, 37067, United States

[b]Department of Chemistry, Vanderbilt University, Nashville, Tennessee, 37235, United States

[c]Department of Biochemistry, Vanderbilt University, Nashville, Tennessee, 37232, United States

*Email: masahito.abe@vanderbilt.edu

Table of Contents

1. **General Information** **S2**
2. **Optimization of a selective aziridine amide ring opening reaction** **S4**
3. **General Synthetic Scheme** **S6**
4. **Synthetic procedures and characterization data for aziridines and aziridine amides** **S9**
5. **Synthetic procedures and characterization data for *trans* substituted cyclic amides** **S20**
6. **Synthetic procedures and characterization data for reference compounds** **S49**
7. **^{1}H, ^{13}C{^{1}H} NMR, 2D NMR spectra and key relative stereochemistry determination** **S51**
8. **X ray crystallographic data** **S168**
9. **Physicochemical property calculation for generated library** **S175**
10. **Kinetic solubility assay** **S176**
11. **References** **S177**

1. General Information

Unless otherwise noted, all of the reagents were purchased from commercial sources and used without further purification. 3,7-diazabicyclo[4.1.0]heptane-3-carboxylic acid, phenyl methyl ester (CAS No. 444188-88-9) was purchased from Combi-blocks and *tert*-Butyl 3,6-diazabicyclo[3.1.0]hexane-3-carboxylate (CAS No. 1262407-18-0) was purchased from Synthonix or Enamine. **Low-resolution mass** spectra were observed on a Waters QDa (Performance) SQ MS with an ESI source. MS parameters were as follows: cone voltage: 15 V, capillary voltage: 0.8 kV, probe temperature: 600 °C. Samples were introduced via an Acquity I-Class PLUS UPLC comprised of a BSM, FL-SM, CH-A, and PDA. UV absorption was generally observed at 215 and 254 nm; 4 nm bandwidth. Column and Gradient conditions were follows: (Condition 1.) Column: Phenomenex EVO C18, 1.0 x 50 mm, 1.7 µm. Column temperature: 55 °C. Flow rate: 0.4 mL/min. Default gradient: 5% to 95% CH_3CN (0.05% TFA) in H_2O (0.05% TFA) over 1.4 min (curve 6), hold at 95% CH_3CN for 0.1 min. (Condition 2.) Column: Waters Acquity UPLC BEH C18, 1.0 x 50 mm, 1.7 µm. Column temperature: 55 °C. Flow rate: 0.35 mL/min. Default gradient: 5% to 95% CH_3CN in H_2O (5 mM NH_4HCO_3) over 1.4 min (curve 5), hold at 95% CH_3CN for 0.1 min. "Polar" (2% to 70% CH_3CN in H_2O (5 mM NH_4HCO_3) over 0.8 min (curve 5), transition to 95% CH_3CN over 0.1 min (curve 5), hold at 95% CH_3CN for 0.6 min.) and "Non-Polar" (40% to 95% CH_3CN in H_2O (5 mM NH_4HCO_3) over 1.4 min (curve 5), hold at 95% CH_3CN for 0.1 min.) gradients were also available. All **NMR** spectra were measured on a 400 MHz Bruker AV-400 instrument. ^1H chemical shifts are reported as δ values in ppm relative to the residual solvent peak ($CDCl_3$= 7.26, CD_3OD = 3.31, $(CD_3)_2SO$ = 2.50). Since most of the *trans* 1,2-disubstituted cyclic products obtained appeared as a mixture of rotamers in the NMR spectra at rt, NMR experiments at 343 K for these compounds were performed to coalesce the signals, which is indicated in parentheses where appropriate. Data are reported as follows: chemical shift, multiplicity (br = broad, s = singlet, d = doublet, t = triplet, q = quartet, dd = doublet of doublets, ddd = doublet of doublet of doublets, td = triplet of doublets, m = multiplet), coupling constant, and integration. ^{13}C chemical shifts are reported as δ values in ppm relative to the residual solvent peak ($CDCl_3$ = 77.16, CD_3OD = 49.0, $(CD_3)_2SO$ = 39.52). **High resolution mass** spectra were observed on an Agilent 6540 UHD Q-TOF with an ESI source. MS parameters were as follows: fragmentor: 150, capillary voltage: 3500 V, nebulizer pressure: 60 psi, drying gas flow: 13 L/min, drying gas temperature: 275 °C. Samples were introduced via an Agilent 1290 UHPLC comprised of a G4220A binary pump, G4226A ALS, G1316C TCC, and G4212A DAD with ULD flow cell. UV absorption was observed at 215 nm and 254 nm with a 4 nm bandwidth. Column: ACQUITY Premier BEH C18, 1.7 µm, 2.1 x 50 mm. Gradient conditions: 5% to 95% CH_3CN in H_2O (0.1% Formic Acid) over 1 min, hold at 95% CH_3CN for 0.1 min, 0.5 mL/min, 40 °C. **Automated normal phase flash column chromatography** was conducted on a Biotage Isolera 1 or a Teledyne ISCO CombiFlash system**. Reverse Phase HPLC** was conducted on a Gilson preparative reversed-phase HPLC system comprised of a 333 aqueous pump with solvent-selection valve, 334 organic pump, GX-271 or GX-281 liquid hander, two column switching valves, and a 155 UV detector. Absorbance was monitored at 215 and 254 nm. Column: Phenomenex Axia-packed Gemini C18, 5 µm. Mobile phase: CH_3CN in H_2O (0.1% TFA) or CH_3CN in H_2O (0.05% v/v NH_4OH)

under the specified gradient, then hold 95% CH_3CN in 5% aqueous phase, 50 mL/min, 23 °C. **Microwave** synthesis was performed in a Biotage Initiator$^+$ microwave synthesis reactor. The used power range for maintaining 140 °C in THF was 133–143W and the range for 110 °C in DMF was 56–66W from magnetron at 2.45 GHz.

2. Optimization of a selective aziridine amide ring opening reaction

2.1 Standard curve generation

Standard Curve for Condition Optimization was generated by first preparing a 500 µM solution of internal standard (naphtalene) in MeOH. To 1 mL of naphthalene solution, was added 15 µL of compound **4aa** from stock solutions prepared at the following concentrations: 100 mM, 50 mM, 25 mM and 12.5 mM. The ratio of DAD integrals (compound **4aa** vs naphtalene) was observed at 215 nm and a linear regression line was plotted (Figure S1). To optimize methodology conditions, 15 µL of reaction mixture was added to 1 mL of 500 µM naphthalene solution in MeOH and compound **4aa** was quantified. The ratio of integrals was used to extrapolate a concentration of a target material **4aa**.

Figure S1. Standard curve generated by internal standard.

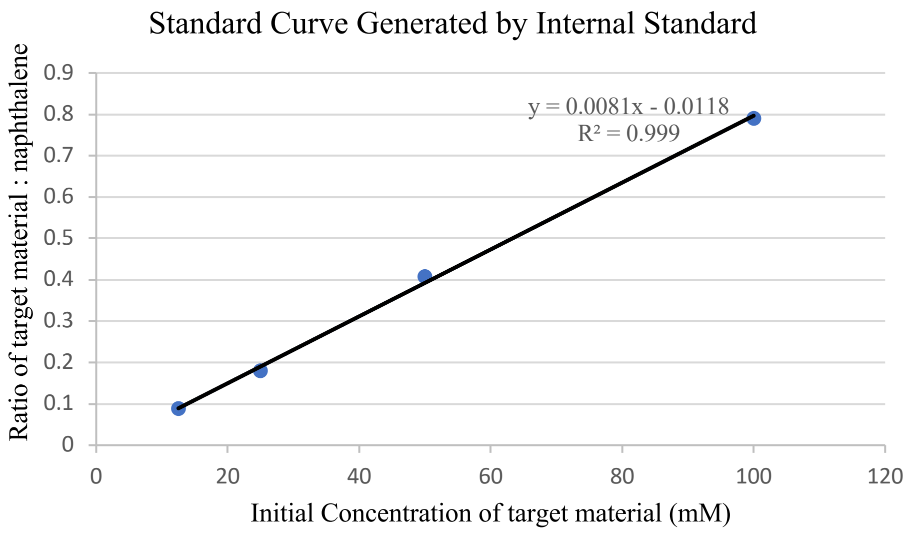

2.2 Table of optimization study

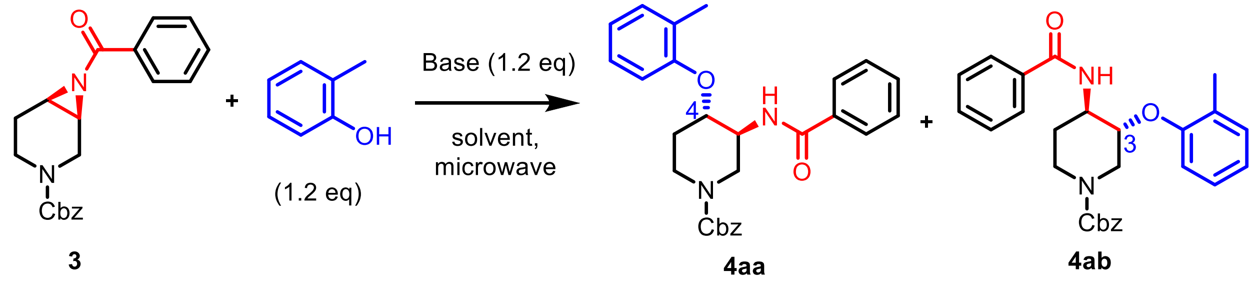

entry	base	solvent	temp (°C)	time (min)	**4aa+4ab** (%)	ratio (**4aa/4ab**)	comment
1	none	THF	110	20	0	-	Base screening
2	Li_2CO_3	THF	110	20	0	-	
3	Na_2CO_3	THF	110	20	0	-	
4	K_2CO_3	THF	110	20	0	-	
5	Cs_2CO_3	THF	110	20	96	3.0/1	
6	DIPEA	THF	110	20	0	-	
7	Pyridine	THF	110	20	0	-	
8	DBU	THF	110	20	3	-	
9	Li_2CO_3	DMF	110	20	10	2.9/1	Solvent/base optimization
10	Na_2CO_3	DMF	110	20	22	4.0/1	
11	K_2CO_3	DMF	110	20	82	4.4/1	
12	Cs_2CO_3	DMF	110	20	85	4.0/1	
13	K_2CO_3	CH_3CN	110	20	29	3.9/1	
14	K_2CO_3	acetone	110	20	22	3.8/1	
15	K_2CO_3	toluene	110	20	0	-	
16	K_2CO_3	DCE	110	20	0	-	
17	K_2CO_3	THF/H_2O (9/1)	110	20	0	-	
18	K_2CO_3	DMF	80	20	43	4.9/1	Reaction temperature optimization
19	K_2CO_3	DMF	140	20	88	3.7/1	
20	Cs_2CO_3	THF	140	20	95	2.7/1	
21	K_2CO_3	DMF	110	10	51	4.5/1	Reaction time optimization
22	Cs_2CO_3	THF	140	10	93	2.8/1	
23	$P\textit{n}Bu_3$	toluene	110	20	0	-	Control experiment

Table S1. Optimization of a selective aziridine ring opening reaction

3. General Synthetic Scheme

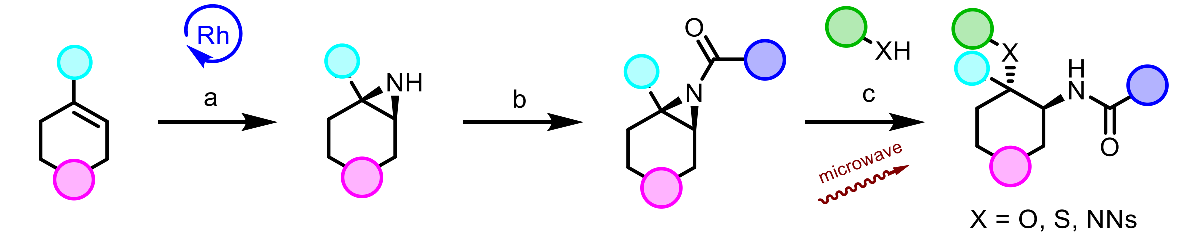

Cyclic olefin (**S1**) | Cyclic aziridine (**S2**) | Cyclic aziridine amide | *trans* substituted cyclic amide

Scheme S1. General synthetic scheme a) $Rh_2(esp)_2$ (1 mol%), $H_2N\text{-}OSO_3H$ (2.4 equiv), pyridine (2.4 equiv), HFIP, rt. b) (a) $R\text{-}CO_2H$ (1.3 equiv), HATU (1.3 equiv), DIPEA (2.5 equiv), DCM, rt. or (b) $(R\text{-}CO)_2O$ (2.4 equiv), NEt_3 (2.6 equiv), DCM, 0 °C. c) (a) NuH (1.2 equiv), Cs_2CO_3 (1.2 equiv), THF, 140 °C, microwave irradiation, 10 min or NuH (1.2 equiv), K_2CO_3 (1.2 equiv), DMF, 110 °C, microwave irradiation, 20 min. HFIP = 1,1,1,3,3,3-hexafluoro-2-propanol, HATU = 1-[bis(dimethylamino)methylene]-1*H*-1,2,3-triazolo[4,5-b]pyridinium 3-oxid hexafluorophosphate, DIPEA = diisopropylethylamine, DCM = dichloromethane, DMF = *N*, *N*-dimethylformamide

As a general method for preparing aziridine amide substrates, 2 step procedures including an aziridination reaction for the corresponding cyclic olefin (**S1**) and the subsequent coupling reaction with variety of carboxylic acids were utilized successfully (Scheme S1). N-H free aziridination was achieved through a nitrene transfer reaction by employing Du Bois' catalyst $Rh_2(esp)_2$,[1] which conditions were originally reported by Kurti et al.[2] The amide formation was conducted through a HATU coupling or acylation with acid anhydride. As for the key ring opening reaction, an optimized condition obtained in Table S1 was applied.

General Procedure A: Aziridination

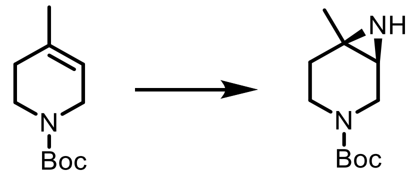

***tert*-Butyl 6-methyl-3,7-diazabicyclo[4.1.0]heptane-3-carboxylate (S2b).** To a solution of *tert*-butyl 4-methyl-3,6-dihydropyridine-1(2*H*)-carboxylate (415 mg, 2.10 mmol) in 1,1,1,3,3,3-hexafluoro-2-propanol (HFIP, 5.25 mL, 49.8 mmol), pyridine (408 µL, 5.05 mmol),

hydroxylamine-*o*-sulfonic acid (571.0 mg, 5.05 mmol) and $Rh_2(esp)_2$ (16.0 mg, 0.021 mmol) were added at 25 °C. The mixture was stirred at rt for 16 h. To this mixture, sat. aq. $NaHCO_3$ (10.0 mL) was added, and the mixture was extracted with DCM (3 × 10.0 mL) and concentrated. Crude product was purified by column chromatography [0–20% MeOH/DIPEA (99/1) in DCM] to give *tert*-butyl 6-methyl-3,7-diazabicyclo[4.1.0]heptane-3-carboxylate (288 mg, 1.36 mmol, 64%) as a colorless oil. ^1H NMR (400 MHz, DMSO-d_6, 343 K): δ 3.70 (dd, J = 13.9, 4.5 Hz, 1H), 3.38–3.27 (m, 2H), 3.01–2.89 (m, 1H), 2.02 (d, J = 4.5 Hz, 1H), 1.74–1.65 (m, 1H), 1.63–1.53 (m, 1H), 1.39 (s, 9H), 1.23 (s, 3H). $^{13}C\{^1H\}$ NMR (101 MHz, DMSO-d_6, 343 K): δ 153.8, 78.1, 42.4, 38.0, 34.6, 32.7, 29.5, 27.8, 24.4. HRMS (TOF, ES+) $C_{11}H_{21}N_2O_2$ [M + H]+ calcd mass 213.1598, found 213.1603.

General Procedure B: Coupling

(B-(a)) via HATU Coupling

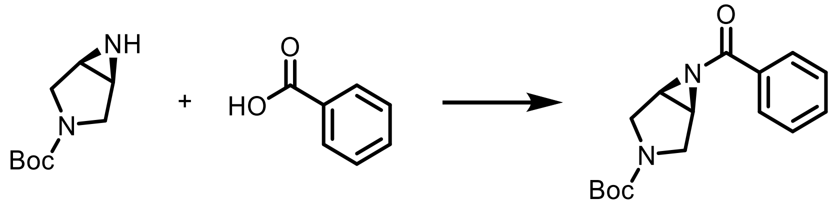

***tert*-Butyl 6-benzoyl-3,6-diazabicyclo[3.1.0]hexane-3-carboxylate (1a).** *tert*-butyl 3,6-diazabicyclo[3.1.0]hexane-3-carboxylate (1.000 g, 5.43 mmol), benzoic acid (862 mg, 7.06 mmol), HATU (2683 mg, 7.06 mmol) and DIPEA (2.363 mL, 13.6 mmol) were dissolved in DCM (10.9 mL) and stirred at rt overnight. To this mixture, sat. aq. $NaHCO_3$ (10.0 mL) was added, and the mixture was extracted with DCM (3 × 10.0 mL) and concentrated. This crude material was purified by column chromatography (0–100% EtOAc in hexane) to give *tert*-butyl 6-benzoyl-3,6-diazabicyclo[3.1.0]hexane-3-carboxylate (1.085 g, 3.77 mmol, 69%) as a white solid. ^1H NMR (400 MHz, DMSO-d_6, 298 K): δ 7.89–7.85 (m, 2H), 7.66–7.59 (m, 1H), 7.55–7.48 (m, 2H), 3.70 (d, J = 12.2 Hz, 1H), 3.63 (d, J = 12.1 Hz, 1H), 3.53–3.48 (m, 2H), 3.25 (dd, J = 12.1, 1.9 Hz, 1H), 3.20 (dd, J = 12.2, 2.0 Hz, 1H), 1.29 (s, 9H). $^{13}C\{^1H\}$ NMR (101 MHz, DMSO-d_6, 298 K): δ 174.8, 153.3, 132.9, 132.7, 128.6, 128.3, 78.8, 45.3, 44.8, 40.9, 40.1, 28.0. HRMS (TOF, ES+) $C_{16}H_{20}N_2NaO_3$ [M + Na]+ calcd mass 311.1366, found 311.1373.

(B-(b)) via Acid anhydride coupling

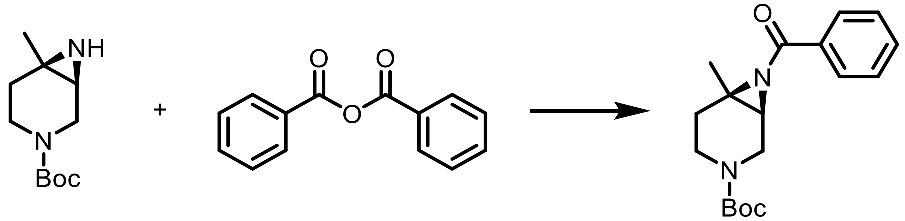

***tert*-Butyl 7-benzoyl-6-methyl-3,7-diazabicyclo[4.1.0]heptane-3-carboxylate (5l).** To a mixture of *tert*-butyl 6-methyl-3,7-diazabicyclo[4.1.0]heptane-3-carboxylate (250 mg, 1.18 mmol) and triethylamine (427 µL, 3.07 mmol) in DCM (11.8 mL) at 0 °C, benzoic anhydride (640

mg, 2.83 mmol) was added. The mixture was stirred at 0 °C for 30 min. To this mixture, sat. aq. $NaHCO_3$ (10.0 mL) was added, and the mixture was extracted with DCM (3 × 10.0 mL) and concentrated. This crude material was purified by column chromatography (0–100% EtOAc in hexane) to give *tert*-butyl 7-benzoyl-6-methyl-3,7-diazabicyclo[4.1.0]heptane-3-carboxylate (268 mg, 0.847 mmol, 72%) as a colorless oil. ^{1}H NMR (400 MHz, DMSO-d_6, 343 K): δ 7.88–7.82 (m, 2H), 7.65–7.58 (m, 1H), 7.56–7.49 (m, 2H), 3.85 (dd, J = 14.3, 4.1 Hz, 1H), 3.65 (d, J = 14.3 Hz, 1H), 3.51–3.40 (m, 1H), 3.26–3.15 (m, 1H), 2.83 (d, J = 3.9 Hz, 1H), 2.16 (dt, J = 14.4, 4.8 Hz, 1H), 1.84–1.73 (m, 1H), 1.40 (s, 9H), 1.06 (s, 3H). $^{13}C\{^{1}H\}$ NMR (101 MHz, DMSO-d_6, 343 K): δ 176.9, 154.1, 134.3, 132.5, 128.5, 128.2, 78.8, 43.8, 41.7, 39.3, 38.4, 28.8, 28.0, 21.0. HRMS (TOF, ES+) $C_{18}H_{25}N_2O_3$ $[M + H]^+$ calcd mass 317.1860, found 317.1869.

General Procedure C: Ring Opening Reaction

(C-(a)) High Yielding Condition

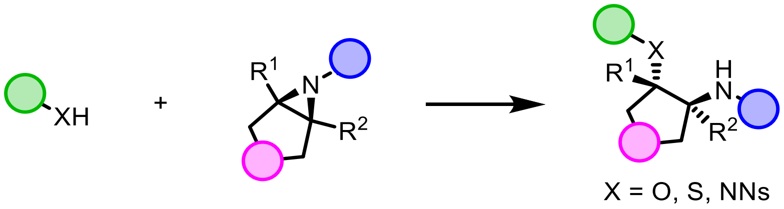

All reactions were performed in a sealed vial. To a solution of an aziridine amide (89.2 µmol, 1.0 equiv) in THF (892 µL, 0.10 M) was added Cs_2CO_3 (35.1 mg, 107 µmol, 1.2 equiv) followed by a nucleophile (107 µmol, 1.2 equiv). The resulting mixture was stirred at 140 °C for 10 min under microwave irradiation. After cooling to rt, sat. aq. $NaHCO_3$ (1.0 mL) was added, and the mixture was extracted with DCM (3 × 1.0 mL) and concentrated. The crude residue was purified by column chromatography or RP-HPLC to give the *trans* substituted cyclic amide.

(C-(b)) Selective Condition

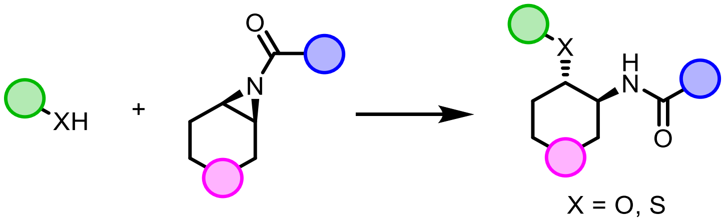

All reactions were performed in a sealed vial. To a solution of an aziridine amide (89.2 µmol, 1.0 equiv) in DMF (892 µL, 0.10 M) was added K_2CO_3 (15.0 mg, 107 µmol, 1.2 equiv) followed by a nucleophile (107 µmol, 1.2 equiv). The resulting mixture was stirred at 110 °C for 20 min under microwave irradiation. After cooling to rt, sat. aq. $NaHCO_3$ (1.0 mL) was added, and the mixture was extracted with DCM (3 × 1.0 mL) and concentrated. The crude residue was purified by column chromatography or RP-HPLC to give the *trans* substituted cyclic amide.

4. Synthetic procedures and characterization data for aziridines and aziridine amides

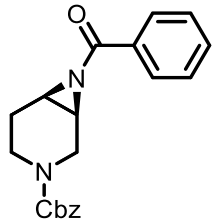

Benzyl 7-benzoyl-3,7-diazabicyclo[4.1.0]heptane-3-carboxylate (3). Followed General Procedure B-(b) with benzyl 3,7-diazabicyclo[4.1.0]heptane-3-carboxylate (906 mg, 3.90 mmol) to give benzyl 7-benzoyl-3,7-diazabicyclo[4.1.0]heptane-3-carboxylate (265 mg, 20%) as a colorless oil after purification by column chromatography (0–100% EtOAc in hexane) and RP-HPLC (45–95% MeCN in 0.05% aqueous NH_4OH). ^{1}H NMR (400 MHz, DMSO-d_6, 343 K): δ 7.92 (d, J = 7.4 Hz, 2H), 7.65–7.57 (m, 1H), 7.54–7.42 (m, 2H), 7.40–7.25 (m, 5H), 5.11 (s, 2H), 4.05 (d, J = 14.5 Hz, 1H), 3.75 (dd, J = 14.5, 3.3 Hz, 1H), 3.53–3.45 (m, 1H), 3.33–3.23 (m, 1H), 3.00–2.95 (m, 1H), 2.94–2.89 (m, 1H), 2.12–2.01 (m, 1H), 2.01–1.90 (m, 1H). ^{13}C{^{1}H} NMR (101 MHz, DMSO-d_6, 343 K): δ 178.1, 154.7, 136.7, 132.7, 132.4, 128.3, 128.3, 128.0, 127.4, 127.1, 66.0, 42.0, 38.5, 34.9, 34.6, 22.1. HRMS (TOF, ES+) $C_{20}H_{21}N_2O_3$ $[M + H]^+$ calcd mass 337.1547, found 337.1554.

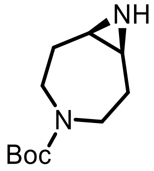

***tert*-Butyl 4,8-diazabicyclo[5.1.0]octane-4-carboxylate (S2a).** Followed General Procedure A with *tert*-butyl 2,3,6,7-tetrahydro-1*H*-azepine-1-carboxylate (500 mg, 2.53 mmol) to give *tert*-butyl 4,8-diazabicyclo[5.1.0]octane-4-carboxylate (335 mg, 66%) as a colorless oil after purification by column chromatography [0–20% MeOH/DIPEA (99/1) in DCM]. ^{1}H NMR (400 MHz, DMSO-d_6, 298 K): δ 3.56–3.41 (m, 2H), 2.79 (t, J = 12.9 Hz, 1H), 2.69 (t, J = 12.7 Hz, 1H), 2.15–2.05 (m, 2H), 1.99–1.73 (m, 4H), 1.37 (s, 9H), 1.21 (t, J = 9.8 Hz, 1H). ^{13}C{^{1}H} NMR (101 MHz, DMSO-d_6, 298 K): δ 154.1, 78.3, 43.8, 43.4, 32.1, 31.9, 29.3, 28.6, 28.3, HRMS (TOF, ES+) $C_{11}H_{21}N_2O_2$ $[M + H]^+$ calcd mass 213.1598, found 213.1594.

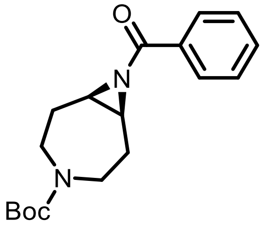

***tert*-Butyl 8-benzoyl-4,8-diazabicyclo[5.1.0]octane-4-carboxylate (1b).** Followed General Procedure B-(a) with *tert*-butyl 4,8-diazabicyclo[5.1.0]octane-4-carboxylate (355 mg, 1.67 mmol) to give *tert*-butyl 8-benzoyl-4,8-diazabicyclo[5.1.0]octane-4-carboxylate (220 mg, 42%) as a

white solid after purification by column chromatography (0–100% EtOAc in hexane). ^{1}H NMR (400 MHz, DMSO-d_6, 298 K): δ 7.88–7.82 (m, 2H), 7.66–7.60 (m, 1H), 7.56–7.50 (m, 2H), 3.82–3.66 (m, 2H), 2.97–2.86 (m, 1H), 2.86–2.77 (m, 3H), 2.30–2.17 (m, 2H), 2.06–1.92 (m, 2H), 1.41 (s, 9H). ^{13}C{^{1}H} NMR (101 MHz, DMSO-d_6, 298 K): δ 179.7, 153.8, 132.9, 132.8, 128.8, 128.6, 78.6, 43.9, 43.5, 40.9, 40.5, 28.4, 28.1, 27.7. HRMS (TOF, ES+) $C_{18}H_{25}N_2O_3$ $[M + H]^+$ calcd mass 317.1860, found 317.1856.

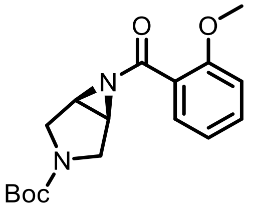

***tert*-Butyl 6-(2-methoxybenzoyl)-3,6-diazabicyclo[3.1.0]hexane-3-carboxylate (5a).** Followed General Procedure B-(a) with *tert*-butyl 3,6-diazabicyclo[3.1.0]hexane-3-carboxylate (250 mg, 1.36 mmol) and 2-methoxybenzoic acid to give *tert*-butyl 6-(2-methoxybenzoyl)-3,6-diazabicyclo[3.1.0]hexane-3-carboxylate (307 mg, 71%) as a white solid after purification by column chromatography (0–100% EtOAc in hexane). ^{1}H NMR (400 MHz, $CDCl_3$, 298 K): δ 7.79 (dd, J = 7.7, 1.8 Hz, 1H), 7.51–7.42 (m, 1H), 7.03–6.94 (m, 2H), 3.91 (s, 3H), 3.81 (t, J = 11.8 Hz, 2H), 3.39–3.33 (m, 1H), 3.32–3.27 (m, 1H), 3.27–3.18 (m, 2H), 1.42 (s, 9H). ^{13}C{^{1}H} NMR (101 MHz, $CDCl_3$, 298 K): δ 175.8, 158.5, 154.5, 133.7, 131.9, 123.1, 120.7, 111.9, 80.0, 56.0, 46.2, 45.9, 41.6, 41.4, 28.5. HRMS (TOF, ES+) $C_{17}H_{23}N_2O_4$ $[M + H]^+$ calcd mass 319.1652, found 319.1656.

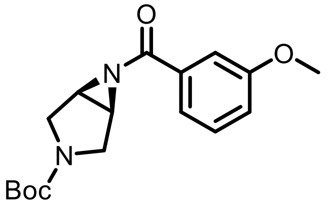

***tert*-Butyl 6-(3-methoxybenzoyl)-3,6-diazabicyclo[3.1.0]hexane-3-carboxylate (5b).** Followed General Procedure B-(a) with *tert*-butyl 3,6-diazabicyclo[3.1.0]hexane-3-carboxylate (250 mg, 1.36 mmol) and 3-methoxybenzoic acid to give *tert*-butyl 6-(3-methoxybenzoyl)-3,6-diazabicyclo[3.1.0]hexane-3-carboxylate (256 mg, 59%) as a pale brown solid after purification by column chromatography (0–100% EtOAc in hexane). ^{1}H NMR (400 MHz, $CDCl_3$, 298 K): δ 7.54 (dd, J = 7.9, 1.5 Hz, 1H), 7.46 (dd, J = 2.7, 1.5 Hz, 1H), 7.36 (t, J = 7.9 Hz, 1H), 7.13–7.06 (m, 1H), 3.98–3.89 (m, 2H), 3.84 (s, 3H), 3.41–3.27 (m, 4H), 1.43 (s, 9H). ^{13}C{^{1}H} NMR (101 MHz, $CDCl_3$, 298 K): δ 176.8, 159.8, 154.6, 134.5, 129.6, 121.3, 119.4, 113.5, 80.2, 55.5, 46.4, 46.2, 41.6, 41.2, 28.5. HRMS (TOF, ES+) $C_{17}H_{22}N_2NaO_4$ $[M + Na]^+$ calcd mass 341.1472, found 341.1477.

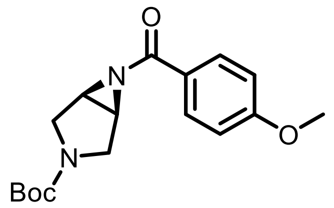

***tert*-butyl 6-(4-methoxybenzoyl)-3,6-diazabicyclo[3.1.0]hexane-3-carboxylate (5c).** Followed General Procedure B-(a) with *tert*-butyl 3,6-diazabicyclo[3.1.0]hexane-3-carboxylate (250 mg, 1.36 mmol) and 4-methoxybenzoic acid to give *tert*-butyl 6-(4-methoxybenzoyl)-3,6-diazabicyclo[3.1.0]hexane-3-carboxylate (199 mg, 46%) as a white solid after purification by column chromatography (0–100% EtOAc in hexane) and RP-HPLC (23-63% MeCN in 0.05% aqueous NH_4OH). ^1H NMR (400 MHz, $CDCl_3$, 298 K): δ 7.97–7.89 (m, 2H), 6.97–6.89 (m, 2H), 3.97–3.89 (m, 2H), 3.87 (s, 3H), 3.39–3.35 (m, 1H), 3.34 (d, J = 2.6 Hz, 1H), 3.32–3.29 (m, 2H), 1.44 (s, 9H). $^{13}C\{^1H\}$ NMR (101 MHz, $CDCl_3$, 298 K): δ 176.6, 163.5, 154.6, 131.1, 125.6, 113.9, 80.1, 55.6, 46.6, 46.4, 41.6, 41.0, 28.5. HRMS (TOF, ES+) $C_{17}H_{23}N_2O_4$ [M + H]+ calcd mass 319.1652, found 319.1659.

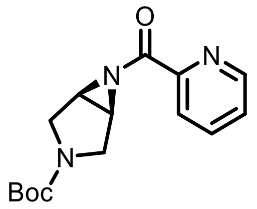

***tert*-butyl 6-picolinoyl-3,6-diazabicyclo[3.1.0]hexane-3-carboxylate (5d).** Followed General Procedure B-(a) with *tert*-butyl 3,6-diazabicyclo[3.1.0]hexane-3-carboxylate (250 mg, 1.36 mmol) and picolinic acid to give *tert*-butyl 6-picolinoyl-3,6-diazabicyclo[3.1.0]hexane-3-carboxylate (307 mg, 71%) as a white solid after purification by column chromatography (0–100% EtOAc in Hexane). ^1H NMR (400 MHz, $CDCl_3$, 298 K): δ 9.18–9.13 (m, 1H), 8.80–8.74 (m, 1H), 8.23–8.15 (m, 1H), 7.40 (dd, J = 8.0, 4.8 Hz, 1H), 3.96–3.88 (m, 2H), 3.42–3.37 (m, 2H), 3.35–3.26 (m, 2H), 1.41 (s, 9H). $^{13}C\{^1H\}$ NMR (101 MHz, $CDCl_3$, 298 K): δ 174.1, 154.4, 150.6, 149.3, 137.0, 126.8, 124.0, 80.0, 46.0, 45.8, 41.3, 40.8, 28.4. HRMS (TOF, ES+) $C_{15}H_{20}N_3O_3$ [M + H]+ calcd mass 290.1499, found 290.1504.

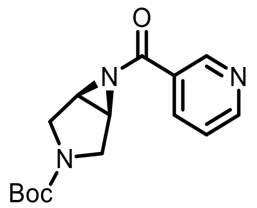

***tert*-Butyl 6-nicotinoyl-3,6-diazabicyclo[3.1.0]hexane-3-carboxylate (5e).** Followed General Procedure B-(a) with *tert*-butyl 3,6-diazabicyclo[3.1.0]hexane-3-carboxylate (250 mg, 1.36 mmol) and nicotinic acid to give *tert*-butyl 6-nicotinoyl-3,6-diazabicyclo[3.1.0]hexane-3-carboxylate (189 mg, 48%) as a pale yellow solid after purification by column chromatography (0–100% EtOAc in hexane) and RP-HPLC (0-40% MeCN in 0.05% aqueous NH_4OH). ^1H NMR

(400 MHz, $CDCl_3$, 298 K): δ 9.16 (s, 1H), 8.77 (d, *J* = 4.8 Hz, 1H), 8.23–8.15 (m, 1H), 7.40 (dd, *J* = 8.0, 4.8 Hz, 1H), 3.96–3.89 (m, 2H), 3.41–3.38 (m, 2H), 3.30 (dd, *J* = 12.3, 4.7 Hz, 2H), 1.41 (s, 9H). $^{13}C\{^1H\}$ NMR (101 MHz, $CDCl_3$, 298 K): δ 175.0, 154.4, 153.5, 150.2, 136.3, 128.9, 123.6, 80.4, 46.1, 45.9, 41.4, 41.3, 28.5. HRMS (TOF, ES+) $C_{15}H_{20}N_3O_3$ $[M + H]^+$ calcd mass 290.1499, found 290.1502.

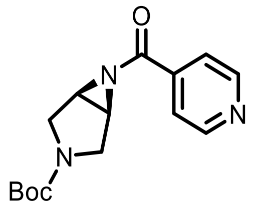

***tert*-Butyl 6-isonicotinoyl-3,6-diazabicyclo[3.1.0]hexane-3-carboxylate (5f).** Followed General Procedure B-(a) with *tert*-butyl 3,6-diazabicyclo[3.1.0]hexane-3-carboxylate (250 mg, 1.36 mmol) and isonicotinic acid to give *tert*-butyl 6-isonicotinoyl-3,6-diazabicyclo[3.1.0]hexane-3-carboxylate (156 mg, 40%) as a white solid after purification by column chromatography (0–100% EtOAc in hexane) and RP-HPLC (5–45% MeCN in 0.05% aqueous NH_4OH). ^1H NMR (400 MHz, DMSO-d_6, 343 K): δ 8.80–8.75 (m, 2H), 7.75–7.69 (m, 2H), 3.69 (d, *J* = 12.3 Hz, 2H), 3.57 (s, 2H), 3.25 (d, *J* = 12.3 Hz, 2H), 1.32 (s, 9H). $^{13}C\{^1H\}$ NMR (101 MHz, DMSO-d_6, 343 K): δ 173.3, 153.1, 150.3, 139.4, 121.4, 78.7, 44.8, 40.5, 27.7. HRMS (TOF, ES+) $C_{15}H_{20}N_3O_3$ $[M + H]^+$ calcd mass 290.1499, found 290.1502

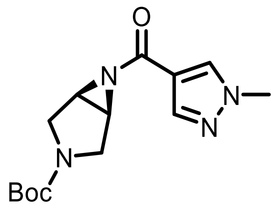

***tert*-Butyl 6-(1-methyl-1*H*-pyrazole-4-carbonyl)-3,6-diazabicyclo[3.1.0]hexane-3-carboxylate (5g).** Followed General Procedure B-(a) with *tert*-butyl 3,6-diazabicyclo[3.1.0]hexane-3-carboxylate (250 mg, 1.36 mmol) and 1-methyl-1*H*-pyrazole-4-carboxylic acid to give *tert*-butyl 6-(1-methyl-1*H*-pyrazole-4-carbonyl)-3,6-diazabicyclo[3.1.0]hexane-3-carboxylate (172 mg, 43%) as a white solid after purification by column chromatography (0–100% EtOAc in hexane) and RP-HPLC (0–40% MeCN in 0.05% aqueous NH_4OH). ^1H NMR (400 MHz, $CDCl_3$, 298 K): δ 7.87 (s, 1H), 7.84 (s, 1H), 3.98–3.90 (m, 2H), 3.94 (s, 3H), 3.36–3.29 (m, 4H), 1.43 (s, 9H). $^{13}C\{^1H\}$ NMR (101 MHz, $CDCl_3$, 298 K): δ 171.8, 154.6, 140.5, 132.9, 118.4, 80.2, 46.5, 46.3, 41.2, 40.6, 39.5, 28.5. HRMS (TOF, ES+) $C_{14}H_{20}N_4NaO_3$ $[M + Na]^+$ calcd mass 315.1428, found 315.1431.

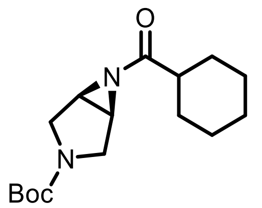

***tert*-Butyl 6-(cyclohexanecarbonyl)-3,6-diazabicyclo[3.1.0]hexane-3-carboxylate (5h).** Followed General Procedure B-(a) with *tert*-butyl 3,6-diazabicyclo[3.1.0]hexane-3-carboxylate (250 mg, 1.36 mmol) and cyclohexanecarboxylic acid to give *tert*-butyl 6-(cyclohexanecarbonyl)-3,6-diazabicyclo[3.1.0]hexane-3-carboxylate (205 mg, 51%) as a white solid after purification by column chromatography (0–100% EtOAc in hexane). ^{1}H NMR (400 MHz, $CDCl_3$, 298 K): δ 3.90 (dd, *J* = 18.3, 12.2 Hz, 2H), 3.26 (d, *J* = 12.2 Hz, 2H), 3.20–3.16 (m, 2H), 2.28 (tt, *J* = 11.4, 3.6 Hz, 1H), 1.94–1.83 (m, 2H), 1.82–1.74 (m, 2H), 1.69–1.61 (m, 1H), 1.50–1.45 (m, 1H), 1.43 (s, 9H), 1.35–1.18 (m, 3H), 0.92–0.81 (m, 1H). $^{13}C\{^{1}H\}$ NMR (101 MHz, $CDCl_3$, 298 K): δ 186.3, 154.7, 80.2, 46.1, 45.9, 39.8, 39.6, 29.9, 29.1, 28.5, 25.9, 25.8, 25.7. HRMS (TOF, ES+) $C_{16}H_{26}N_2NaO_3$ $[M + Na]^+$ calcd mass 317.1836, found 317.1839.

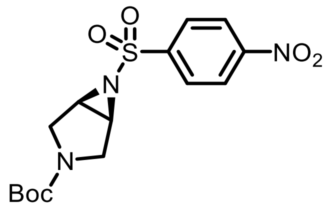

***tert*-Butyl 6-((4-nitrophenyl)sulfonyl)-3,6-diazabicyclo[3.1.0]hexane-3-carboxylate (5i).** To a mixture of *tert*-butyl 3,6-diazabicyclo[3.1.0]hexane-3-carboxylate (1000 mg, 5.43 mmol) and triethylamine (1.13 mL, 8.14 mmol) in DCM (10.9 mL), 4-nitrobenzenesulfonyl chloride (1443 mg, 6.51 mmol) was added at rt and the mixture was stirred at rt for 5 h. To this mixture, sat. aq. $NaHCO_3$ (10.0 mL) was added, and the mixture was extracted with DCM (3 × 10 mL) and concentrated. The crude product was purified by column chromatography (0–100% EtOAc in hexane) to give a mixture of *tert*-butyl 6-((4-nitrophenyl)sulfonyl)-3,6-diazabicyclo[3.1.0]hexane-3-carboxylate and ring opening product by Cl. To this mixture in MeCN (10.9 mL), K_2CO_3 (1.14 g, 8.14 mmol) was added, and the mixture was stirred at 110 °C for 30 min under microwave irradiation. After cooling to rt, sat. aq. $NaHCO_3$ (10.0 mL) was added, and the mixture was extracted with DCM (3 × 10.0 mL) and concentrated. The crude product was purified by column chromatography (0–60% EtOAc in Hexane) to give *tert*-butyl 6-((4-nitrophenyl)sulfonyl)-3,6-diazabicyclo[3.1.0]hexane-3-carboxylate (434 mg, 1.18 mmol, 22% (2 steps)) as a pale yellow solid. ^{1}H NMR (400 MHz, $CDCl_3$, 298 K): δ 8.45–8.37 (m, 2H), 8.20–8.12 (m, 2H), 3.76–3.67 (m, 3H), 3.62 (dd, *J* = 5.7, 2.6 Hz, 1H), 3.46–3.36 (m, 2H), 1.41 (s, 9H). $^{13}C\{^{1}H\}$ NMR (101 MHz, $CDCl_3$, 298 K): δ 154.3, 150.9, 144.1, 129.3, 124.6, 80.6, 47.5, 47.1, 45.2, 45.1, 28.5. HRMS (TOF, ES+) $C_{15}H_{19}N_3NaO_6S$ $[M + Na]^+$ calcd mass 392.0887, found 392.0882.

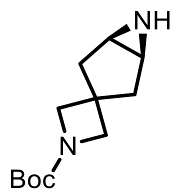

***tert*-Butyl 6'-azaspiro[azetidine-3,3'-bicyclo[3.1.0]hexane]-1-carboxylate (S2f).** Followed General Procedure A with *tert*-butyl 2-azaspiro[3.4]oct-6-ene-2-carboxylate (976 mg, 4.66 mmol) to give *tert*-butyl 6-(3-methoxybenzoyl)-3,6-diazabicyclo[3.1.0]hexane-3-carboxylate (708 mg, 68%) as a white solid after purification by column chromatography [0–20% MeOH/DIPEA (99/1) in DCM]. ^{1}H NMR (400 MHz, $CDCl_3$, 298 K): δ 3.78 (s, 2H), 3.76 (s, 2H), 3.66 (s, 2H), 2.57 (s, 2H), 2.23 (d, J = 13.6 Hz, 2H), 1.81 (d, J = 13.6 Hz, 2H), 1.41 (s, 9H). ^{13}C{^{1}H} NMR (101 MHz, $CDCl_3$, 298 K): δ 156.3, 79.2, 66.8, 57.4, 40.3, 36.2, 34.4, 28.5. HRMS (TOF, ES+) $C_{12}H_{21}N_2O_2$ $[M + H]^+$ calcd mass 225.1598, found 225.1605.

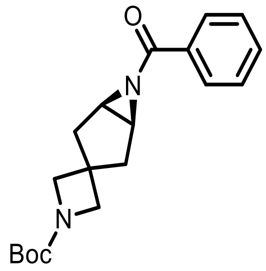

***tert*-Butyl 6'-benzoyl-6'-azaspiro[azetidine-3,3'-bicyclo[3.1.0]hexane]-1-carboxylate (5j).** Followed General Procedure B-(a) with tert-butyl 6'-azaspiro[azetidine-3,3'-bicyclo[3.1.0]hexane]-1-carboxylate (92.2 mg, 0.411 mmol) to give *tert*-butyl 6'-benzoyl-6'-azaspiro[azetidine-3,3'-bicyclo[3.1.0]hexane]-1-carboxylate (89.1 mg, 66%) as a white solid after purification by column chromatography (0–100% EtOAc in Hexane). ^{1}H NMR (400 MHz, $CDCl_3$, 298 K): δ 7.97–7.92 (m, 2H), 7.59–7.51 (m, 1H), 7.49–7.41 (m, 2H), 3.88 (s, 2H), 3.78 (s, 2H), 3.21 (s, 2H), 2.52 (d, J = 13.8 Hz, 2H), 1.91 (d, J = 13.8 Hz, 2H), 1.44 (s, 9H). ^{13}C{^{1}H} NMR (101 MHz, $CDCl_3$, 298 K): δ 178.5, 156.3, 133.3, 132.9, 128.9, 128.6, 79.6, 65.9, 56.8, 42.7, 39.6, 36.8, 28.5. HRMS (TOF, ES+) $C_{19}H_{25}N_2O_3$ $[M + H]^+$ calcd mass 329.1860, found 329.1864.

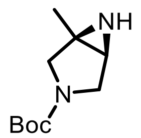

***tert*-Butyl 1-methyl-3,6-diazabicyclo[3.1.0]hexane-3-carboxylate (S2c).** Followed General Procedure A with *tert*-butyl 3-methyl-2,5-dihydro-1*H*-pyrrole-1-carboxylate (350 mg, 1.91 mmol) to give *tert*-butyl 1-methyl-3,6-diazabicyclo[3.1.0]hexane-3-carboxylate as a pale yellow oil. This material was used immediately for the next reaction due to its instability. HRMS (TOF, ES+) $C_{10}H_{19}N_2O_2$ $[M + H]^+$ calcd mass 199.1441, found 199.1441.

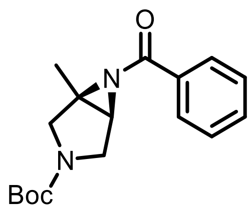

***tert*-Butyl 6-benzoyl-1-methyl-3,6-diazabicyclo[3.1.0]hexane-3-carboxylate (5k).** Followed General Procedure B-(b) with *tert*-butyl 1-methyl-3,6-diazabicyclo[3.1.0]hexane-3-carboxylate (61 mg, 308 mmol) to give *tert*-butyl 6-benzoyl-1-methyl-3,6-diazabicyclo[3.1.0]hexane-3-carboxylate [192.4 mg, 33% (2 steps)] as a white solid after purification by column chromatography (0–100% EtOAc in hexane) and RP-HPLC (17–57% MeCN in 0.05% aqueous NH_4OH). ^1H NMR (400 MHz, DMSO-*d*$_6$, 343 K): δ 7.81–7.76 (m, 2H), 7.63–7.57 (m, 1H), 7.54–7.47 (m, 2H), 3.70–3.50 (m, 2H), 3.33–3.20 (m, 2H), 3.11 (d, *J* = 12.0 Hz, 1H), 1.49 (s, 3H), 1.28 (s, 9H). $^{13}C\{^1H\}$ NMR (101 MHz, DMSO-*d*$_6$, 343 K): δ 174.5, 152.8, 133.6, 132.1, 128.1, 127.7, 78.5, 48.3, 48.0, 45.0, 44.6, 27.7, 15.7. HRMS (TOF, ES+) $C_{17}H_{23}N_2O_3$ $[M + H]^+$ calcd mass 303.1703, found 303.1703

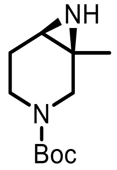

***tert*-Butyl 1-methyl-3,7-diazabicyclo[4.1.0]heptane-3-carboxylate (S2d).** Followed General Procedure A with *tert*-butyl 5-methyl-3,6-dihydropyridine-1(2*H*)-carboxylate (500 mg, 2.53 mmol) to give *tert*-butyl 6-(3-methoxybenzoyl)-3,6-diazabicyclo[3.1.0]hexane-3-carboxylate (358 mg, 67%) as a colorless oil after purification by column chromatography [0–20% MeOH/DIPEA (99/1) in DCM]. ^1H NMR (400 MHz, DMSO-d6, 343 K): δ 3.57 (d, *J* = 13.6 Hz, 1H), 3.29–3.21 (m, 2H), 3.04–2.93 (m, 1H), 2.02–1.98 (m, 1H), 1.79–1.65 (m, 2H), 1.39 (s, 9H), 1.21 (s, 3H). $^{13}C\{^1H\}$ NMR (101 MHz, DMSO-d6, 343 K): δ 154.0, 78.1, 47.0, 38.1, 34.4, 32.6, 27.9, 23.3, 22.5. HRMS (TOF, ES+) $C_{11}H_{21}N_2O_2$ $[M + H]^+$ calcd mass 213.1598, found 213.1605.

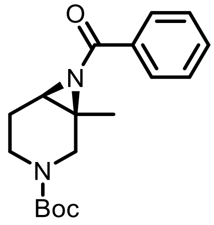

***tert*-Butyl 7-benzoyl-1-methyl-3,7-diazabicyclo[4.1.0]heptane-3-carboxylate (5m).** Followed General Procedure B-(a) with *tert*-butyl 1-methyl-3,7-diazabicyclo[4.1.0]heptane-3-carboxylate (176 mg, 0.831 mmol) to give *tert*-butyl 7-benzoyl-1-methyl-3,7-diazabicyclo[4.1.0]heptane-3-

carboxylate (179 mg, 68%) as a white solid after purification by column chromatography (0–100% EtOAc in Hexane). ^{1}H NMR (400 MHz, DMSO-d_6, 343 K): δ 7.96–7.89 (m, 2H), 7.66–7.59 (m, 1H), 7.56–7.48 (m, 2H), 4.18 (d, J = 14.4 Hz, 1H), 3.69–3.52 (m, 1H), 3.25 (d, J = 14.4 Hz, 1H), 3.00–2.84 (m, 1H), 2.80 (t, J = 3.5 Hz, 1H), 1.98–1.90 (m, 2H), 1.42 (s, 9H), 0.99 (s, 3H). ^{13}C{^{1}H} NMR (101 MHz, DMSO-d_6, 343 K): δ 177.0, 154.1, 134.3, 132.3, 128.2, 128.1, 78.6, 46.7, 42.9, 39.1, 38.5, 27.8, 21.3, 17.8. HRMS (TOF, ES+) $C_{18}H_{25}N_2O_3$ $[M + H]^+$ calcd mass 317.1860, found 317.1857.

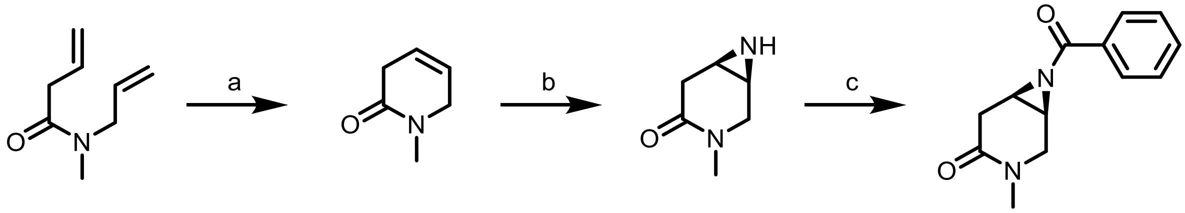

S1a S2e 5n

Scheme S2. Synthesis of 6-membered lactam aziridine benzamide a) *N*-allyl-*N*-methylbut-3-enamide (1.0 equiv), Grubbs II (5 mol%), DCM, rt, 54 %. b) $Rh_2(esp)_2$ (1 mol%), $H_2N\text{-}OSO_3H$ (2.4 equiv), pyridine (2.4 equiv), HFIP, rt. c) benzoic acid (1.3 equiv), HATU (1.3 equiv), NEt_3 (2.5 equiv), DCM, rt, 8.2 % (2 steps). Grubbs II = Grubbs Catalyst 2nd Generation, (1,3-bis(2,4,6-trimethylphenyl)-2-imidazolidinylidene)dichloro(phenylmethylene)(tricyclohexyl phosphine)ruthenium

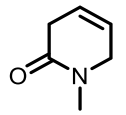

1-Methyl-3,6-dihydropyridin-2(1*H*)-one (S1a). To a solution of *N*-allyl-*N*-methylbut-3-enamide (1.00 g, 7.18 mmol), which was prepared following the known procedure[3], in DCM (143 mL), Grubbs catalyst II (305 mg, 0.359 mmol) was added at rt. The mixture was stirred for 18 h under N_2. The reaction mixture was concentrated, and the crude material was purified by column chromatography (0–100% EtOAc in hexane) to give 1-methyl-3,6-dihydropyridin-2(1*H*)-one (434 mg, 3.91 mmol, 54% yield) as a colorless oil. ^{1}H NMR (400 MHz, $CDCl_3$, 298 K): δ 5.80–5.73 (m, 1H), 5.72–5.65 (m, 1H), 3.95–3.87 (m, 2H), 2.99 (s, 3H), 2.97–2.90 (m, 2H). ^{13}C{^{1}H} NMR (101 MHz, $CDCl_3$, 298 K): δ 167.3, 123.0, 120.7, 51.2, 34.2, 32.0. HRMS (TOF, ES+) $C_6H_{10}NO$ $[M + H]^+$ calcd mass 112.0757, found 112.0761.

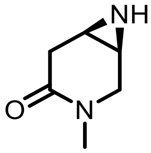

3-Methyl-3,7-diazabicyclo[4.1.0]heptan-4-one (S2e). Followed General Procedure A with 1-methyl-3,6-dihydropyridin-2(1H)-one (173 mg, 1.56 mmol) to give 3-methyl-3,7-

diazabicyclo[4.1.0]heptan-4-one as a yellow oil after purification by column chromatography [0–20% MeOH/DIPEA (99/1) in DCM]. This material was used immediately for the next reaction due to its instability. HRMS (TOF, ES+) $C_6H_{11}N_2O$ $[M + H]^+$ calcd mass 127.0866, found 127.0869.

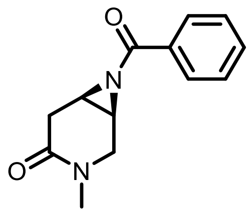

7-Benzoyl-3-methyl-3,7-diazabicyclo[4.1.0]heptan-4-one (5n). Followed General Procedure B-(a) with 3-methyl-3,7-diazabicyclo[4.1.0]heptan-4-one in the previous step to give 7-benzoyl-3-methyl-3,7-diazabicyclo[4.1.0]heptan-4-one [29.3 mg, 8.2% (2 steps)] as a colorless oil after purification by column chromatography (0–100% EtOAc in hexane). ^1H NMR (400 MHz, $CDCl_3$, 298 K): δ 7.96–7.91 (m, 2H), 7.58–7.51 (m, 1H), 7.47–7.40 (m, 2H), 3.82 (dd, J = 13.9, 2.1 Hz, 1H), 3.72 (d, J = 13.9 Hz, 1H), 3.14 (dt, J = 6.6, 1.9 Hz, 1H), 3.09 (d, J = 17.8 Hz, 1H), 3.02–2.97 (m, 1H), 2.95 (s, 3H), 2.69 (dd, J = 17.8, 2.8 Hz, 1H). $^{13}C\{^1H\}$ NMR (101 MHz, $CDCl_3$, 298 K): δ 178.8, 166.2, 133.2, 132.8, 129.2, 128.7, 48.1, 37.0, 35.1, 34.3, 32.5. HRMS (TOF, ES+) $C_{13}H_{15}N_2O_2$ $[M + H]^+$ calcd mass 231.1128, found 231.1130.

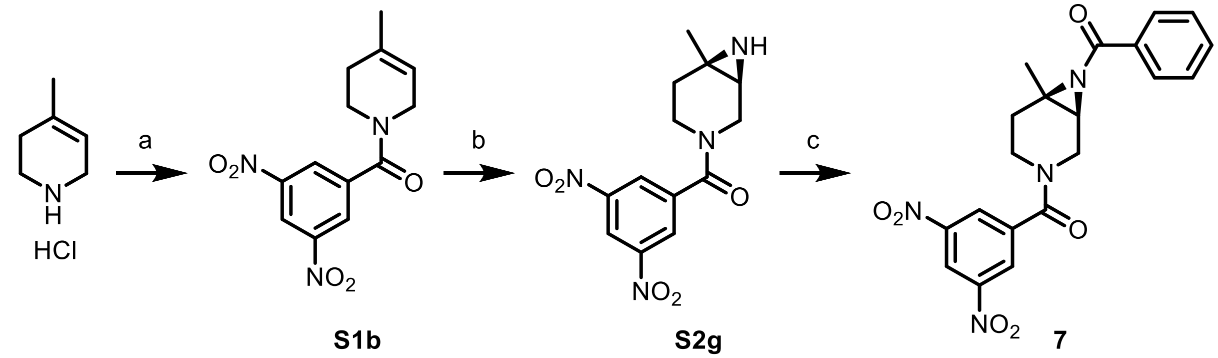

Scheme S3. Synthesis of 4-methyl substituted piperidine aziridine benzamide (**7**) a) 4-methyl-1,2,3,6-tetrahydropyridine hydrochloride (1.0 equiv), 3,5-dinitrobenzoic acid (1.2 equiv), HATU (1.5 equiv), DIPEA (3.0 equiv), DCM, rt, 89 %. b) $Rh_2(esp)_2$ (1 mol%), $H_2N\text{-}OSO_3H$ (2.4 equiv), pyridine (2.4 equiv), HFIP, rt, 61%. c) Bz_2O (2.4 equiv), NEt_3 (2.6 equiv), DCM, 0 °C, 93 %.

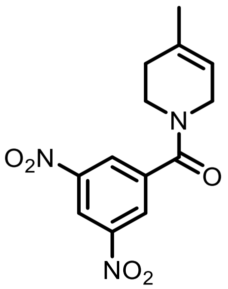

(3,5-Dinitrophenyl)(4-methyl-3,6-dihydropyridin-1(2*H*)-yl)methanone (S1b). 4-methyl-1,2,3,6-tetrahydropyridine hydrochloride (700 mg, 5.24 mmol), 3,5-dinitrobenzoic acid (1.334 mg, 6.29 mmol), HATU (2988 mg, 7.86 mmol) and DIPEA (2.738 mL, 15.7 mmol) were dissolved in DCM (10.5 mL) and stirred at rt overnight. To this mixture, sat. aq. $NaHCO_3$ (10.0 mL) was added, and the mixture was extracted with DCM (3 × 10.0 mL) and concentrated. This crude material was purified by column chromatography (0–100% EtOAc in Hexane) to give (3,5-dinitrophenyl)(4-methyl-3,6-dihydropyridin-1(2*H*)-yl)methanone (1.351 g, 4.64 mmol, 89%) as a yale yellow solid. ^1H NMR (400 MHz, DMSO-d_6, 343 K): δ 8.87 (t, J = 2.1 Hz, 1H), 8.58 (d, J = 2.1 Hz, 2H), 5.55–5.24 (m, 1H), 4.15–3.27 (m, 4H), 2.14–2.05 (m, 2H), 1.72 (s, 3H). $^{13}C\{^1H\}$ NMR (101 MHz, DMSO-d_6, 343 K): δ 164.6, 148.0, 139.0, 132.5, 126.9, 118.8, 117.1, 46.2, 43.7, 41.5, 29.2, 22.3. (Note; 1 carbon peak next to nitrogen in the piperidine ring is split into 2 peaks at 46.2, 41.5, which is consistent with HSQC analysis.) HRMS (TOF, ES+) $C_{13}H_{14}N_3O_5$ $[M + H]^+$ calcd mass 292.0928, found 292.0922.

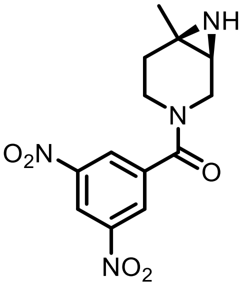

(3,5-Dinitrophenyl)((1*SR*,6*RS*)-6-methyl-3,7-diazabicyclo[4.1.0]heptan-3-yl)methanone (S2g). Followed General Procedure A with (3,5-dinitrophenyl)(4-methyl-3,6-dihydropyridin-1(2H)-yl)methanone (500 mg, 1.72 mmol) to give (3,5-dinitrophenyl)((1*SR*,6*RS*)-6-methyl-3,7-diazabicyclo[4.1.0]heptan-3-yl)methanone (321 mg, 61%) as a white solid after purification by column chromatography (0–20% MeOH/DIPEA (99/1) in DCM). ^1H NMR (400 MHz, DMSO-d_6, 343 K): δ 8.85 (t, J = 2.1 Hz, 1H), 8.75–8.36 (m, 2H), 4.10–3.32 (m, 3H), 2.25–1.95 (m, 1H), 1.88–1.64 (m, 2H), 1.28 (s, 3H), 1.19 (s, 1H). $^{13}C\{^1H\}$ NMR (101 MHz, DMSO-d_6, 343 K): δ 165.1, 147.9, 139.2, 127.2, 118.6, 46.4, 41.7, 37.9, 34.6, 32.8, 29.9, 28.5, 24.6. (Note; 2 carbon peaks next to nitrogen in the piperidine ring are split into 3 peaks at 46.4, 41.7, 37.9 ppm and the other methylene carbon in the ring is split into 2 peaks at 29.9, 28.5 ppm, which are consistent with HSQC analysis.) HRMS (TOF, ES+) $C_{13}H_{15}N_4O_5$ $[M + H]^+$ calcd mass 307.1037, found 307.1035.

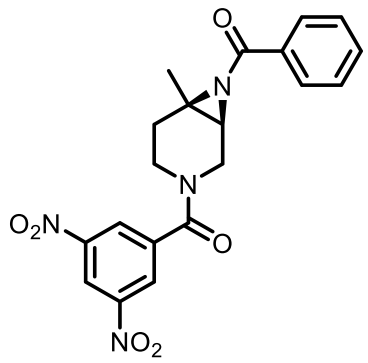

((1*SR*,6RS)-7-Benzoyl-6-methyl-3,7-diazabicyclo[4.1.0]heptan-3-yl)(3,5-dinitrophenyl)methanone (7). Followed General Procedure B-(b) with (3,5-dinitrophenyl)((1*SR*,6*RS*)-6-methyl-3,7-diazabicyclo[4.1.0]heptan-3-yl)methanone (220 mg, 0.718 mmol) to give ((1*SR*,6*RS*)-7-benzoyl-6-methyl-3,7-diazabicyclo[4.1.0]heptan-3-yl)(3,5-dinitrophenyl)methanone (275 mg, 93%) as a white solid after purification by column chromatography (0–100% EtOAc in hexane) and RP-HPLC (14–54% MeCN in 0.05% aqueous NH_4OH). ^{1}H NMR (400 MHz, DMSO-d_6, 343 K): δ 8.89–8.85 (m, 1H), 8.62–8.57 (m, 2H), 7.91–7.84 (m, 2H), 7.68–7.59 (m, 1H), 7.57–7.49 (m, 2H), 4.45–3.23 (m, 4H), 3.09–2.74 (m, 1H), 2.38–2.06 (m, 1H), 2.03–1.91 (m, 1H), 1.11 (s, 3H). ^{13}C{^{1}H} NMR (101 MHz, DMSO-d_6, 343 K): δ 176.7, 165.3, 148.0, 138.8, 134.0, 132.5, 128.4, 128.1, 127.1, 118.8, 45.1, 44.1, 41.7, 40.5, 39.0, 37.5, 29.1, 28.0, 20.7. (Note; 3 methylene carbon peaks in the piperidine ring are split into 2 peaks at 45.1/40.7 ppm (next to nitrogen), 41.7/37.5 ppm (next to nitrogen) and 29.1/28.0 ppm, which are consistent with HSQC analysis.) HRMS (TOF, ES+) $C_{20}H_{19}N_4O_6$ $[M + H]^+$ calcd mass 411.1299, found 411.1297.

5. Synthetic procedures and characterization data for *trans* substituted cyclic amides

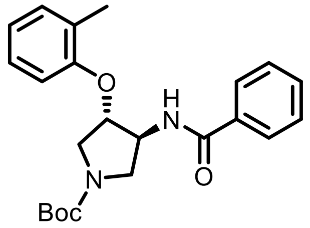

***tert*-Butyl (3*SR*,4*SR*)-3-benzamido-4-(*o*-tolyloxy)pyrrolidine-1-carboxylate (2a).** Followed General Procedure C-(a) with *tert*-butyl 6-benzoyl-3,6-diazabicyclo[3.1.0]hexane-3-carboxylate (25 mg, 86.7 µmol) and *o*-cresol to give *tert*-butyl (3*SR*,4*SR*)-3-benzamido-4-(2-methylphenoxy)pyrrolidine-1-carboxylate (29.1 mg, 85%) as a white solid after purification by column chromatography (0–100% EtOAc in hexane). ^{1}H NMR (400 MHz, DMSO-d_6, 343 K): δ 8.55 (d, *J* = 6.4 Hz, 1H), 7.91–7.83 (m, 2H) 7.58–7.50 (m, 1H), 7.50–7.42 (m, 2H), 7.23–7.11 (m, 3H), 6.88 (td, *J* = 6.9, 1.8 Hz, 1H), 4.87 (dt, *J* = 4.5, 2.1 Hz, 1H), 4.59–4.49 (m, 1H), 3.79–3.68 (m, 2H), 3.52–3.40 (m, 2H), 2.14 (s, 3H), 1.43 (s, 9H). ^{13}C{^{1}H} NMR (101 MHz, DMSO-d_6, 343 K): δ 166.7, 154.7, 153.5, 133.9, 131.0, 130.4, 127.8, 127.2, 126.6, 126.5, 120.8, 113.3, 78.4, 27.9, 15.4. (δ 78.7, 53.4, 49.1, 48.9 are additional peaks observed in HSQC, but not clear in 1D-^{13}C{^{1}H} NMR.) HRMS (TOF, ES+) $C_{23}H_{28}N_2NaO_4$ [M + Na]$^+$ calcd mass 419.1941, found 419.1939.

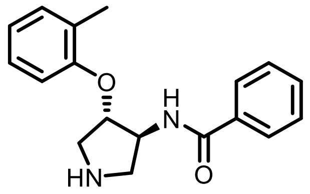

***N*-((3*SR*,4*SR*)-4-(*o*-Tolyloxy)pyrrolidin-3-yl)benzamide (f).** To a solution of *tert*-butyl (3*SR*,4*SR*)-3-benzamido-4-(2-methylphenoxy)pyrrolidine-1-carboxylate (24.5 mg, 117 µmol) in DCM (0.5 mL), TFA (0.5 mL) was added at rt. The mixture was stirred at rt for 10 min and concentrated. To this mixture, sat. aq. $NaHCO_3$ (1.0 mL) was added, and the mixture was extracted with DCM (3 × 1.0 mL) and concentrated. The crude material was purified by RP-HPLC (9–49% MeCN in 0.05% aqueous NH_4OH) and concentrated to give *N*-((3*SR*,4*SR*)-4-(*o*-tolyloxy)pyrrolidin-3-yl)benzamide (16.0 mg, 87%) as a white solid. ^{1}H NMR (400 MHz, $CDCl_3$, 298 K): δ 7.86–7.78 (m, 2H), 7.54–7.47 (m, 1H), 7.47–7.38 (m, 2H), 7.21–7.16 (m, 2H), 7.13 (dd, *J*=7.5, 1.4, 1H), 6.87 (dt, *J*=7.4, 4.1, 1H), 6.78 (d, *J*=7.1, 1H), 4.84–4.78 (m, 1H), 4.67–4.59 (m, 1H), 3.53 (dd, *J*=11.3, 5.8, 1H), 3.35 (dd, *J*=12.3, 4.6, 1H), 3.20 (d, *J*=12.3, 1H), 2.98 (dd, *J*=11.3, 2.4, 1H), 2.66–2.62 (m, 1H), 2.21 (s, 3H). ^{13}C{^{1}H} NMR (101 MHz, $CDCl_3$, 298 K): δ 167.6, 155.4, 134.2, 131.9, 130.9, 128.7, 127.2, 127.2, 127.1, 121.0, 112.6, 81.5, 56.2, 51.5, 51.0, 16.5. HRMS (TOF, ES+) $C_{18}H_{21}N_2O_2$ [M + H]$^+$ calcd mass 297.1598, found 297.1601.

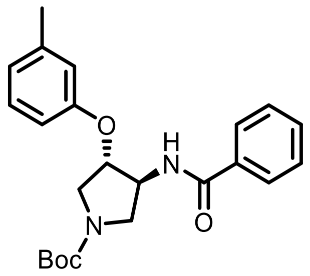

***tert*-Butyl (3*SR*,4*SR*)-3-benzamido-4-(3-methylphenoxy)pyrrolidine-1-carboxylate (2b).** Followed General Procedure C-(a) with *tert*-Butyl 6-benzoyl-3,6-diazabicyclo[3.1.0]hexane-3-carboxylate (25 mg, 86.7 µmol) and *m*-cresol to give *tert*-butyl (3*SR*,4*SR*)-3-benzamido-4-(3-methylphenoxy)pyrrolidine (30.7 mg, 89%) as a white solid after purification by column chromatography (0–100% EtOAc in hexane). ^{1}H NMR (400 MHz, DMSO-d_6, 343 K): δ 8.56 (d, *J* = 6.4 Hz, 1H), 7.92–7.84 (m, 2H) 7.57–7.51 (m, 1H), 7.50–7.43 (m, 2H), 7.18 (t, *J* = 7.7 Hz, 1H), 6.95–6.87 (m, 2H), 6.82–6.76 (m, 1H), 4.87 (dt, *J* = 4.6, 2.1 Hz, 1H), 4.51 (tt, *J* = 6.2, 2.5 Hz, 1H), 3.75 (dd, J = 12.2, 4.6 Hz, 1H), 3.70 (dd, *J* = 11.5, 6.5 Hz, 1H), 3.49–3.41 (m, 2H), 2.29 (s, 3H), 1.44 (s, 9H). ^{13}C{^{1}H} NMR (101 MHz, DMSO-d_6, 343 K): δ 166.7, 156.7, 153.4, 138.9, 133.9, 131.0, 129.0, 127.8, 127.2, 121.7, 116.1, 112.3, 78.4, 53.5, 49.3, 48.6, 27.9, 20.7. (A δ 78.3 is an additional peak observed in HSQC, but not clear in 1D-^{13}C{^{1}H} NMR.) HRMS (TOF, ES+) $C_{23}H_{29}N_2O_4$ $[M + H]^+$ calcd mass 397.2122, found 397.2119.

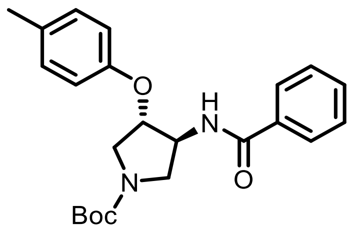

***tert*-Butyl (3*SR*,4*SR*)-3-benzamido-4-(4-methylphenoxy)pyrrolidine-1-carboxylate (2c).** Followed General Procedure C-(a) with *tert*-butyl 6-benzoyl-3,6-diazabicyclo[3.1.0]hexane-3-carboxylate (25 mg, 86.7 µmol) and *p*-cresol to give *tert*-butyl (3*SR*,4*SR*)-3-benzamido-4-(4-methylphenoxy)pyrrolidine-1-carboxylate (30.1 mg, 87%) as a white solid after purification by column chromatography (0–100% EtOAc in hexane). ^{1}H NMR (400 MHz, DMSO-d_6, 343 K): δ 8.55, (d, *J* = 6.4 Hz, 1H), 7.92–7.82 (m, 2H), 7.58–7.50 (m, 1H), 7.50–7.42 (m, 2H), 7.14–7.06 (m, 2H), 7.04–6.94 (m, 2H), 4.84 (dt, *J* = 4.5, 2.1 Hz, 1H), 4.55–4.45 (m, 1H), 3.77–3.65 (m, 2H), 3.48–3.40 (m, 2H), 2.25 (s, 3H), 1.44 (s, 9H). ^{13}C{^{1}H} NMR (101 MHz, DMSO-d_6, 343 K): δ 166.6, 154.5, 153.4, 133.9, 131.0, 129.8, 129.6, 127.8, 127.2, 115.4, 78.4, 53.4, 49.3, 48.6, 27.9, 19.7. (A δ 78.3 is an additional peak observed in HSQC, but not in ^{13}C{^{1}H} NMR.) HRMS (TOF, ES+) $C_{23}H_{29}N_2O_4$ $[M + H]^+$ calcd mass 397.2122, found 397.2121.

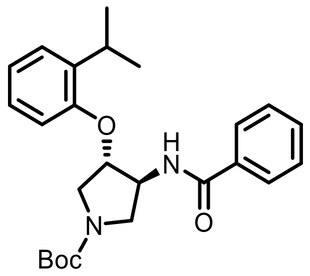

***tert*-Butyl (3*SR*, 4*SR*)-3-benzamido-4-(2-isopropylphenoxy)pyrrolidine-1-carboxylate (2d).** Followed General Procedure C-(a) with *tert*-butyl 6-benzoyl-3,6-diazabicyclo[3.1.0]hexane-3-carboxylate (25 mg, 86.7 µmol) and 2-isopropylphenol to give *tert*-butyl (3*SR*,4*SR*)-3-benzamido-4-(2-isopropylphenoxy)pyrrolidine-1-carboxylate (35 mg, 95%) as a white solid after purification by column chromatography (0–100% EtOAc in hexane). *On a 1 mmol scale (288 mg), 392 mg (92%) was obtained after column chromatography*. ^{1}H NMR (400 MHz, DMSO-d_6, 343 K): δ 8.58 (d, *J* = 6.4 Hz 1H), 7.93–7.85 (m, 2H), 7.58–7.50 (m, 1H), 7.50–7.43 (m, 2H), 7.23–7.13 (m, 3H), 6.93 (td, *J* = 7.3, 1.4 Hz, 1H), 4.90 (dt, *J* = 4.3, 2.0 Hz, 1H), 4.61–4.54 (m, 1H), 3.78–3.68 (m, 2H), 3.50 (d, *J* = 12.3 Hz, 1H), 3.46 (dd, *J* = 11.6, 2.6 Hz, 1H), 3.20 (dq, J = 6.9, 6.9 Hz, 1H), 1.43 (s, 9H), 1.16 (dd, *J* = 6.9, 4.9 Hz, 6H). ^{13}C{^{1}H} NMR (101 MHz, DMSO-d_6, 343 K): δ 166.6, 153.6, 153.5, 136.6, 133.9, 131.0, 127.8, 127.2, 126.4, 126.0, 120.9, 112.9, 78.3, 53.7, 49.2, 48.8, 27.9, 26.6, 22.1, 22.0. (A δ 78.2 is an additional peak observed in HSQC, but not clear in 1D-^{13}C{^{1}H} NMR.) HRMS (TOF, ES+) $C_{25}H_{32}N_2NaO_4$ [M + Na]$^+$ calcd mass 447.2254, found 447.2241.

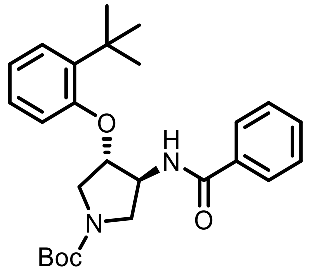

***tert*-Butyl (3*SR*,4*SR*)-3-benzamido-4-(2-tert-butylphenoxy)pyrrolidine-1-carboxylate (2e).** Followed General Procedure C-(a) with *tert*-butyl 6-benzoyl-3,6-diazabicyclo[3.1.0]hexane-3-carboxylate (25 mg, 86.7 µmol) and 2-*tert*-butylphenol to give *tert*-butyl (3*SR*,4*SR*)-3-benzamido-4-(2-*tert*-butylphenoxy)pyrrolidine-1-carboxylate (34.6 mg, 91%) as a white solid after purification by column chromatography (0–100% EtOAc in hexane). ^{1}H NMR (400 MHz, DMSO-d_6, 343 K): δ 8.62 (d, *J* = 6.3 Hz, 1H), 7.94–7.88 (m, 2H), 7.58–7.51 (m, 1H), 7.50–7.44 (m, 2H), 7.29 (brs, 1H), 7.26 (dd, *J* =7.8, 1.7 Hz, 1H), 7.21–7.15 (m, 1H), 6.89 (td, *J* = 7.5, 1.2 Hz, 1H), 4.96 (dt, *J* = 4.0, 1.8 Hz, 1H), 4.65–4.58 (m, 1H), 3.79–3.70 (m, 2H), 3.58 (d, *J* = 12.3 Hz, 1H), 3.47 (dd, *J* = 11.7, 2.3 Hz, 1H), 1.42 (s, 9H), 1.33 (s, 9H). ^{13}C{^{1}H} NMR (101 MHz, DMSO-d_6, 343 K): δ 166.7, 154.8, 153.4, 137.2, 133.8, 131.0, 127.8, 127.2, 126.8, 126.2, 120.2, 112.4, 78.3, 34.0, 29.4, 27.9. (δ 77.4, 53.2, 49.1, 48.7 are additional peaks observed in HSQC,

but not clear in 1D-$^{13}C\{^1H\}$ NMR.) HRMS (TOF, ES+) $C_{26}H_{34}N_2NaO_4$ $[M + Na]^+$ calcd mass 461.2411, found 461.2412.

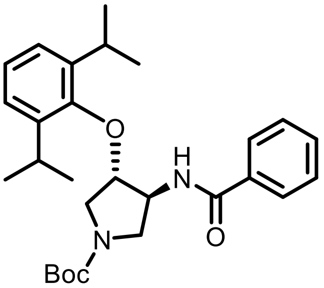

***tert*-Butyl (3*SR*,4*SR*)-3-benzamido-4-(2,6-diisopropylphenoxy)pyrrolidine-1-carboxylate (2f).** Followed General Procedure C-(a) with *tert*-butyl 6-benzoyl-3,6-diazabicyclo[3.1.0]hexane-3-carboxylate (25 mg, 86.7 µmol) and 2,6-diisopropylphenol to give *tert*-butyl (3*SR*,4*SR*)-3-benzamido-4-(2,6-diisopropylphenoxy)pyrrolidine-1-carboxylate (37.7 mg, 93%) as a white solid after purification by column chromatography (0–100% EtOAc in hexane). ^1H NMR (400 MHz, DMSO-d_6, 343 K): δ 8.50 (d, J = 6.4 Hz, 1H), 7.88–7.82 (m, 2H), 7.56–7.49 (m, 1H), 7.49–7.41 (m, 2H), 7.15–7.04 (m, 3H), 4.66 (tt, J = 6.4, 2.1 Hz, 1H), 4.46 (q, J = 2.6 Hz, 1H), 3.81 (dd, J = 11.5, 6.4 Hz, 1H), 3.53 (d, J = 11.6 Hz, 1H), 3.47–3.42 (m, 2H), 3.34 (dq, J = 6.9, 6.9 Hz, 2H), 1.45 (s, 9H), 1.18 (d, J = 6.8 Hz, 6H), 1.18 (d, J = 6.8 Hz, 6H), 1.14 (d, J = 6.8 Hz, 6H). $^{13}C\{^1H\}$ NMR (101 MHz, DMSO-d_6, 343 K): δ 166.4, 153.3, 150.3, 141.2, 134.0, 130.9, 127.8, 127.2, 124.3, 123.7, 78.3, 49.0, 47.9, 27.9, 25.7, 23.8, 23.3. (δ 84.4, 54.0 are additional peaks observed in HSQC, but not clear in 1D-$^{13}C\{^1H\}$ NMR.) HRMS (TOF, ES+) $C_{28}H_{38}N_2NaO_4$ $[M + Na]^+$ calcd mass 489.2724, found 489.2721.

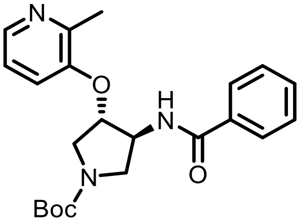

***tert*-Butyl (3*SR*,4*SR*)-3-benzamido-4-[(2-methyl-3-pyridyl)oxy]pyrrolidine-1-carboxylate (2g).** Followed General Procedure C-(a) with *tert*-butyl 6-benzoyl-3,6-diazabicyclo[3.1.0]hexane-3-carboxylate (25 mg, 86.7 µmol) and 2-methyl-3-pyridinol to give *tert*-butyl (3*SR*,4*SR*)-3-benzamido-4-[(2-methyl-3-pyridyl)oxy]pyrrolidine-1-carboxylate (30.5 mg, 88%) as a white solid after purification by column chromatography (0–100% EtOAc in hexane). ^1H NMR (400 MHz, DMSO-d_6, 343 K): δ 8.59 (d, J = 6.4 Hz, 1H), 8.05 (dd, J = 4.7, 1.3 Hz, 1H), 7.91–7.84 (m, 2H), 7.68 (d, J = 8.2 Hz, 1H), 7.57–7.51 (m, 1H), 7.51–7.43 (m, 2H), 7.19 (dd, J = 8.2, 4.7 Hz, 1H), 4.90 (dt, J = 4.3, 2.0 Hz, 1H), 4.54 (dt, J = 6.8, 3.9 Hz, 1H), 3.79–3.71 (m, 2H), 3.54–3.44 (m, 2H), 2.35 (s, 3H), 1.44 (s, 9H). $^{13}C\{^1H\}$ NMR (101 MHz, DMSO-d_6, 343 K): δ 166.8, 153.5, 150.9, 148.2, 140.7, 133.8, 131.0, 127.8, 127.2, 121.6, 119.7, 78.4, 53.5, 49.3, 48.5, 27.9, 18.6. (A δ 78.8 is an additional peak observed in HSQC, but not clear in

$^{13}C\{^1H\}$ NMR.) HRMS (TOF, ES+) $C_{22}H_{28}N_3O_4$ $[M + H]^+$ calcd mass 398.2074, found 398.2076.

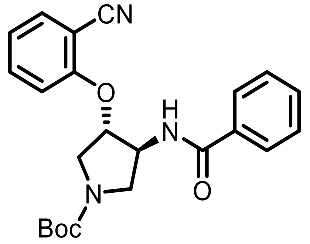

***tert*-Butyl (3*SR*,4*SR*)-3-benzamido-4-(2-cyanophenoxy)pyrrolidine-1-carboxylate (2h).** Followed General Procedure C-(a) with *tert*-butyl 6-benzoyl-3,6-diazabicyclo[3.1.0]hexane-3-carboxylate (25 mg, 86.7 µmol) and 2-cyanophenol to give *tert*-butyl (3*SR*,4*SR*)-3-benzamido-4-(2-cyanophenoxy)pyrrolidine-1-carboxylate (24.5 mg, 69%) as a white solid after purification by column chromatography (0–100% EtOAc in hexane). ^1H NMR (400 MHz, DMSO-d_6, 343 K): δ 8.64 (d, J = 6.2 Hz, 1H), 7.93–7.85 (m, 2H), 7.74–7.62 (m, 3H), 7.58–7.51 (m, 1H), 7.47 (dd, J = 8.2, 6.7 Hz, 2H), 7.18–7.12 (m, 1H), 5.07 (dt, J = 4.1, 1.8 Hz, 1H), 4.57–4.50 (m, 1H), 3.81–3.71 (m, 2H), 3.55 (d, J = 12.6 Hz, 1H), 3.51 (dd, J = 11.7, 2.3 Hz, 1H), 1.45 (s, 9H). $^{13}C\{^1H\}$ NMR (101 MHz, DMSO-d_6, 343 K): δ 166.9, 158.3, 153.4, 134.6, 133.7, 133.4, 131.1, 127.8, 127.3, 121.6, 115.6, 114.5, 101.5, 78.5, 53.6, 49.2, 48.4, 27.9. (A δ 79.7 is an additional peak observed in HSQC, but not clear in $^{13}C\{^1H\}$ NMR.) HRMS (TOF, ES+) $C_{23}H_{26}N_3O_4$ $[M + H]^+$ calcd mass 408.1918, found 408.1919.

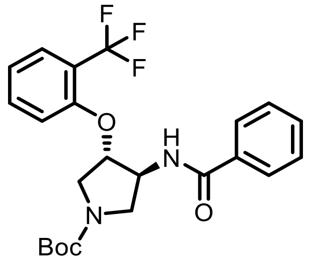

***tert*-Butyl (3*SR*,4*SR*)-3-benzamido-4-[2-(trifluoromethyl)phenoxy]pyrrolidine-1-carboxylate (2i).** Followed General Procedure C-(a) with *tert*-butyl 6-benzoyl-3,6-diazabicyclo[3.1.0]hexane-3-carboxylate (25 mg, 86.7 µmol) and 2-(trifluoromethyl)phenol to give *tert*-butyl (3*SR*,4*SR*)-3-benzamido-4-[2-(trifluoromethyl)phenoxy]pyrrolidine-1-carboxylate (21 mg, 54%) as a white solid after purification by column chromatography (0–100% EtOAc in Hexane). ^1H NMR (400 MHz, DMSO-d_6, 343 K): δ 8.62 (d, J = 6.2 Hz, 1H), 7.93–7.86 (m, 2H), 7.68–7.60 (m, 3H), 7.57–7.51 (m, 1H), 7.50–7.44 (m, 2H), 7.17–7.11 (m, 1H), 5.06 (dt, J = 3.9, 1.7 Hz, 1H), 4.53 (t, J = 6.3 Hz, 1H), 3.76–3.65 (m, 2H), 3.57–3.51 (m, 1H), 3.48 (dd, J = 11.6, 2.1 Hz, 1H), 1.43 (s, 9H). $^{13}C\{^1H\}$ NMR (101 MHz, DMSO-d_6, 343 K): δ 166.8, 154.3, 153.4, 133.8, 131.1, 127.8, 127.3, 126.6 (q, J = 5.3 Hz), 122.4 (q, J = 272.6 Hz), 120.7, 117.8 (q, J = 30.8 Hz), 114.7, 78.4, 49.1, 48.4, 27.9. (δ 78.9, 53.1 are additional peaks observed in HSQC, but

not clear in 1D-$^{13}C\{^1H\}$ NMR.) HRMS (TOF, ES+) $C_{23}H_{25}F_3N_2NaO_4$ $[M + Na]^+$ calcd mass 473.1659, found 473.1655.

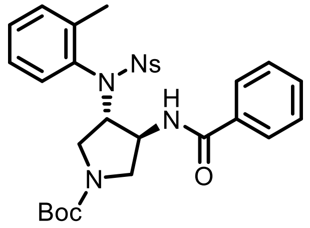

***tert*-Butyl (3*SR*,4*SR*)-3-benzamido-4-((4-nitro-*N*-(*o*-tolyl)phenyl)sulfonamido)pyrrolidine-1-carboxylate (2j).** Followed General Procedure C-(a) with *tert*-butyl 6-benzoyl-3,6-diazabicyclo[3.1.0]hexane-3-carboxylate (25.7 mg, 89.2 μmol), Cs_2CO_3 (58.5 mg, 178 μmol, 2.0 equiv) and 4-nitro-*N*-(*o*-tolyl)benzenesulfonamide (52.1 mg, 107 μmol, 2.0 equiv) to give *tert*-butyl (3*SR*,4*SR*)-3-benzamido-4-((4-nitro-N-(*o*-tolyl)phenyl)sulfonamido)pyrrolidine-1-carboxylate (36.4 mg, 70%) as a white solid after purification by RP-HPLC (39–79% MeCN in 0.05% aqueous NH_4OH). HRMS (TOF, ES+) $C_{29}H_{33}N_4O_7S$ $[M + H]^+$ calcd mass 581.2064, found 581.2061. Since ^1H and $^{13}C\{^1H\}$ NMR spectra showed complex pattern when the temperature of NMR measurement was raised up to 343 K, NMR characterization of this product was conducted in the deprotected form of the 4-Ns group.

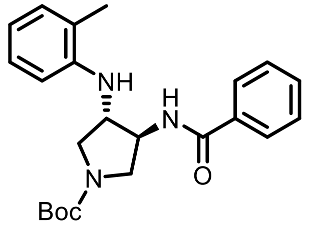

***tert*-Butyl (3*SR*,4*SR*)-3-benzamido-4-(*o*-tolylamino)pyrrolidine-1-carboxylate (2j').** To a mixture of *tert*-butyl (3*SR*,4*SR*)-3-benzamido-4-((4-nitro-*N*-(*o*-tolyl)phenyl)sulfonamido)pyrrolidine-1-carboxylate (22.8 mg, 39.3 μmol), Cs_2CO_3 (19.3 mg, 58.9 μmol) in MeCN (393 μL), thiophenol (4.8 μL, 47.1 μmol) was added and the mixture was stirred at rt for 6 h. After concentrating the reaction mixture, it was filtered and purified by RP-HPLC (33–73% MeCN in 0.05% aqueous NH_4OH) to give *tert*-butyl (3*SR*,4*SR*)-3-benzamido-4-(*o*-tolylamino)pyrrolidine-1-carboxylate (13.8 mg, 89 %) as a colorless oil. ^1H NMR (400 MHz, DMSO-d_6, 343 K): δ 8.50 (d, J = 7.1 Hz, 1H), 7.87–7.81 (m, 2H), 7.55–7.49 (m, 1H), 7.49–7.42 (m, 2H), 7.05–6.94 (m, 2H), 6.79–6.72 (m, 1H), 6.55 (td, J = 7.3, 1.1 Hz, 1H), 5.01–4.68 (m, 1H), 4.62–4.53 (m, 1H), 4.06–3.97 (m, 1H), 3.88 (dd, J = 11.0, 6.7 Hz, 1H), 3.74 (dd, J = 11.0, 7.2 Hz, 1H), 3.35 (dd, J = 11.0, 6.2 Hz, 1H), 3.21 (dd, J = 11.0, 5.7 Hz, 1H), 2.10 (s, 3H), 1.43 (s, 9H). $^{13}C\{^1H\}$ NMR (101 MHz, DMSO-d_6, 343 K): δ 167.1, 153.4, 145.3, 134.0, 130.9, 129.6, 127.8, 127.1, 126.4, 122.1, 116.4, 110.3, 78.3, 56.9, 53.6, 50.0, 48.3, 28.0, 17.1. HRMS (TOF, ES+) $C_{23}H_{30}N_3O_3$ $[M + H]^+$ calcd mass 396.2282, found 396.2276.

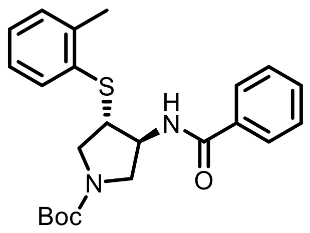

***tert*-Butyl (3*SR*,4*SR*)-3-benzamido-4-(*o*-tolylsulfanyl)pyrrolidine-1-carboxylate (2k).**
Followed General Procedure C-(a) with *tert*-butyl 6-benzoyl-3,6-diazabicyclo[3.1.0]hexane-3-carboxylate (25 mg, 86.7 µmol) and *o*-thiocresol to give *tert*-butyl (3*SR*,4*SR*)-3-benzamido-4-(*o*-tolylsulfanyl)pyrrolidine-1-carboxylate (34 mg, 95%) as a colorless oil after purification by column chromatography (0–100% EtOAc in hexane). ^{1}H NMR (400 MHz, DMSO-d_6, 343 K): δ 8.53 (d, J = 7.2 Hz, 1H), 7.86–7.78 (m, 2H), 7.57–7.49 (m, 2H), 7.49–7.41 (m, 2H), 7.27–7.21 (m, 1H), 7.21–7.14 (m, 2H), 4.44 (tt, J = 7.1, 5.2 Hz, 1H), 3.92–3.72 (m, 3H), 3.34–3.24 (m, 2H), 2.35 (s, 3H), 1.42 (s, 9H). ^{13}C{^{1}H} NMR (101 MHz, DMSO-d_6, 343 K): δ 166.4, 153.1, 138.3, 134.0, 132.7, 131.0, 129.9, 127.8, 127.1, 126.8, 126.3, 78.5, 53.6, 49.8, 49.2, 47.9, 27.9, 19.8. HRMS (TOF, ES+) $C_{23}H_{28}N_2NaO_3S$ $[M + Na]^+$ calcd mass 435.1713, found 435.1714

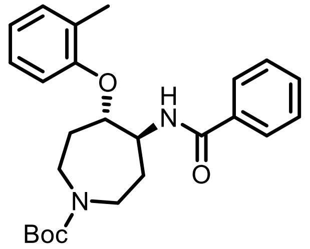

***tert*-Butyl (4*SR*,5*SR*)-4-benzamido-5-(2-methylphenoxy)azepane-1-carboxylate (2l).**
Followed General Procedure C-(a) with *tert*-butyl 8-benzoyl-4,8-diazabicyclo[5.1.0]octane-4-carboxylate (25 mg, 79.0 µmol) and *o*-cresol to give *tert*-butyl (4*SR*,5*SR*)-4-benzamido-5-(2-methylphenoxy)azepane-1-carboxylate (28.3 mg, 84%) as a white solid after purification by column chromatography (0–100% EtOAc in hexane). ^{1}H NMR (400 MHz, DMSO-d_6, 343 K): δ 8.09 (d, J = 8.0 Hz, 1H), 7.77–7.71 (m, 2H), 7.51–7.45 (m, 1H), 7.45–7.37 (m, 2H), 7.17–7.10 (m, 1H), 7.10–7.04 (m, 1H), 7.00 (d, J = 8.1 Hz, 1H), 6.80 (td, J = 7.3, 1.1 Hz, 1H), 4.59–4.50 (m, 1H), 4.36 (qd, J = 8.2, 4.1 Hz, 1H), 3.71–3.59 (m, 1H), 3.56–3.32 (m, 3H), 2.23–2.11 (m, 1H), 2.06 (s, 3H), 2.04–1.79 (m, 3H), 1.44 (s, 9H). ^{13}C{^{1}H} NMR (101 MHz, DMSO-d_6, 343 K): δ 165.9, 155.3, 154.2, 134.8, 130.6, 130.3, 127.7, 126.9, 126.4, 126.3, 119.9, 112.3, 78.3, 77.2, 52.1, 30.4, 29.3, 27.9, 15.5. (δ 40.7, 40.3 are additional peaks observed in HSQC, but not clear in 1D-^{13}C{^{1}H} NMR.) HRMS (TOF, ES+) $C_{25}H_{32}N_2NaO_4$ $[M + Na]^+$ calcd mass 447.2254, found 447.2251.

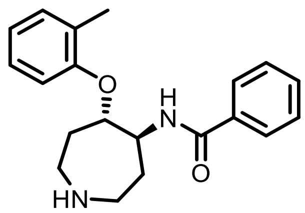

***N*-((4*SR*,5*SR*)-5-(*o*-Tolyloxy)azepan-4-yl)benzamide (g).** To a solution of *tert*-butyl (4*SR*,5*SR*)-4-benzamido-5-(2-methylphenoxy)azepane-1-carboxylate (17.7 mg, 41.7 μmol) in DCM (0.5 mL), TFA (0.5 mL) was added at rt. The mixture was stirred at rt for 10 min and concentrated. To this mixture, sat. aq. $NaHCO_3$ (1.0 mL) was added, and the mixture was extracted with DCM (3x1.0 mL) and concentrated. The crude material was purified by RP-HPLC (6-46% MeCN in 0.05% aqueous NH_4OH) and concentrated to give *N*-((4*SR*,5*SR*)-5-(*o*-tolyloxy)azepan-4-yl)benzamide (10.8 mg, 80%) as a white solid. ^1H NMR (400 MHz, $CDCl_3$, 298 K): δ 7.97 (d, *J* = 9.2 Hz, 1H), 7.85–7.78 (m, 2H), 7.54–7.48 (m, 1H), 7.47–7.41 (m, 2H), 7.20–7.11 (m, 2H), 7.03 (d, *J* = 8.1 Hz, 1H), 6.85 (td, *J* = 7.4, 1.1 Hz, 1H), 4.99–4.90 (m, 1H), 4.82–4.75 (m, 1H), 3.21–3.08 (m, 2H), 3.06–2.88 (m, 2H), 2.44–2.32 (m, 1H), 2.25 (s, 3H), 2.07–1.97 (m, 3H), 1.95–1.83 (m, 1H). $^{13}C\{^1H\}$ NMR (101 MHz, $CDCl_3$, 298 K): δ 166.7, 155.4, 134.7, 131.5, 131.0, 128.7, 127.4, 127.1, 120.6, 112.5, 74.9, 49.6, 45.0, 43.5, 32.9, 25.6, 16.7. HRMS (TOF, ES+) $C_{20}H_{25}N_2O_2$ [M + H]+ calcd mass 325.1911, found 325.1915.

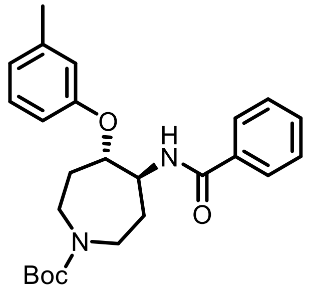

***tert*-Butyl (4*SR*,5*SR*)-4-benzamido-5-(3-methylphenoxy)azepane-1-carboxylate (2m).** Followed General Procedure C-(a) with *tert*-butyl 8-benzoyl-4,8-diazabicyclo[5.1.0]octane-4-carboxylate (25 mg, 79.0 μmol) and *m*-cresol to give *tert*-butyl (4*SR*,5*SR*)-4-benzamido-5-(3-methylphenoxy)azepane-1-carboxylate (27.2 mg, 81%) as a white solid after purification by column chromatography (0–100% EtOAc in hexane). ^1H NMR (400 MHz, DMSO-*d*$_6$, 343 K): δ 8.08 (d, *J* = 8.0 Hz, 1H), 7.78–7.68 (m, 2H), 7.53–7.45 (m, 1H), 7.45–7.37 (m, 2H), 7.12 (t, *J* = 7.9 Hz, 1H), 6.77–6.69 (m, 3H), 4.53 (td, *J* = 8.2, 2.8 Hz, 1H), 4.28 (qd, *J* = 8.3, 4.0 Hz, 1H), 3.69–3.57 (m, 1H), 3.51 (ddd, *J* = 14.1, 7.0, 3.8 Hz, 1H), 3.45–3.30 (m, 2H), 2.24 (s, 3H), 2.14 (ddt, *J* = 15.0, 6.9, 3.5 Hz, 1H), 2.01 (ddt, *J* = 14.6, 7.3, 3.6 Hz, 1H), 1.97–1.78 (m, 2H), 1.44 (s, 9H). $^{13}C\{^1H\}$ NMR (101 MHz, DMSO-*d*$_6$, 343 K): δ 165.8, 157.5, 138.6, 134.7, 130.6, 128.8, 127.7, 126.9, 121.2, 116.6, 112.8, 78.3, 77.6, 52.3, 40.9, 30.1, 29.5, 27.9, 20.7. (A δ 40.2 is an additional peak observed in HSQC, but not clear in $^{13}C\{^1H\}$ NMR.) HRMS (TOF, ES+) $C_{25}H_{32}N_2NaO_4$ $[M + Na]^+$ calcd mass 447.2254, found 447.2247.

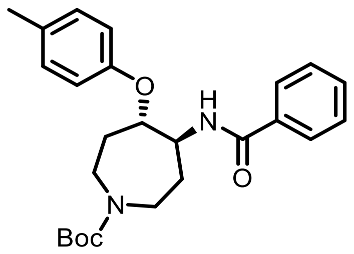

***tert*-Butyl (4*SR*,5*SR*)-4-benzamido-5-(4-methylphenoxy)azepane-1-carboxylate (2n).** Followed General Procedure C-(a) with *tert*-butyl 8-benzoyl-4,8-diazabicyclo[5.1.0]octane-4-carboxylate (25 mg, 79.0 µmol) and *p*-cresol to give *tert*-butyl (4*SR*,5*SR*)-4-benzamido-5-(4-methylphenoxy)azepane-1-carboxylate (30.2 mg, 90%) as a white solid after purification by column chromatography (0–100% EtOAc in hexane). ^{1}H NMR (400 MHz, DMSO-d_6, 343 K): δ 8.08 (d, *J* = 7.8 Hz, 1H), 7.78–7.70 (m, 2H), 7.52–7.45 (m, 1H), 7.45–7.38 (m, 2H), 7.08–7.01 (m, 2H), 6.86–6.79 (m, 2H), 4.49 (td, *J* = 8.1, 2.7 Hz, 1H), 4.27 (qd, *J* = 8.2, 4.0 Hz, 1H), 3.68–3.58 (m, 1H), 3.55–3.46 (m, 1H), 3.44–3.29 (m, 2H), 2.21 (s, 3H), 2.13 (ddt, *J* = 15.0, 6.9, 3.4 Hz, 1H), 2.02 (ddt, *J* = 14.7, 7.3, 3.6 Hz, 1H), 1.96–1.78 (m, 2H), 1.44 (s, 9H). ^{13}C{^{1}H} NMR (101 MHz, DMSO-d_6, 343 K): δ 165.8, 155.3, 154.2, 134.8, 130.6, 129.5, 129.3, 127.7, 126.9, 115.9, 78.3, 77.9, 52.3, 40.9, 30.1, 29.4, 27.9, 19.7. (A δ 40.2 is an additional peak observed in HSQC, but not clear in^{13}C{^{1}H} NMR.) HRMS (TOF, ES+) $C_{25}H_{32}N_2NaO_4$ [M + Na]$^+$ calcd mass 447.2254, found 447.2243.

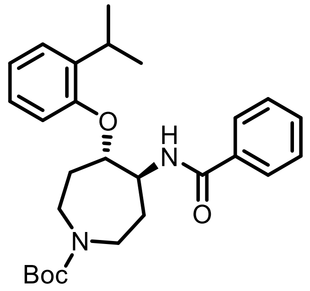

***tert*-Butyl (4*SR*,5*SR*)-4-benzamido-5-(2-isopropylphenoxy)azepane-1-carboxylate (2o).** Followed General Procedure C-(a) with *tert*-butyl 8-benzoyl-4,8-diazabicyclo[5.1.0]octane-4-carboxylate (25 mg, 79.0 µmol) and 2-isopropylphenol to give *tert*-butyl (4*SR*,5*SR*)-4-benzamido-5-(2-isopropylphenoxy)azepane-1-carboxylate (33.9 mg, 95%) after purification by column chromatography (0–100% EtOAc in hexane) as a white solid. ^{1}H NMR (400 MHz, DMSO-d_6, 343 K): δ 8.12 (d, *J* = 8.1 Hz, 1H), 7.78–7.72 (m, 2H), 7.51–7.44 (m, 1H), 7.44–7.36 (m, 2H), 7.17–7.09 (m, 2H), 7.00 (d, *J* = 8.0 Hz, 1H), 6.86 (td, *J* = 7.4, 1.1 Hz, 1H), 4.59 (td, *J* = 8.1, 2.5 Hz, 1H), 4.37 (qd, *J* = 8.2, 4.1 Hz, 1H), 3.70–3.60 (m, 1H), 3.55–3.33 (m, 3H), 3.23 (hept, *J* = 7.0 Hz, 1H), 2.23–2.12 (m, 1H), 2.07–1.81 (m, 3H), 1.44 (s, 9H), 1.11 (d, *J* = 6.9 Hz, 3H), 1.00 (d, *J* = 6.9 Hz, 3H). ^{13}C{^{1}H} NMR (101 MHz, DMSO-d_6, 343 K): δ 165.8, 154.2, 154.0, 136.7, 134.7, 130.5, 127.7, 126.9, 126.2, 125.7, 120.1, 112.1, 78.3, 76.7, 52.2, 30.6, 29.2, 27.9, 25.8, 22.4, 22.2. (δ 40.7, 40.3 are additional peaks observed in HSQC, but not clear in 1D-

$^{13}C\{^1H\}$ NMR.) HRMS (TOF, ES+) $C_{27}H_{36}N_2NaO_4$ $[M + Na]^+$ calcd mass 475.2567, found 475.2563.

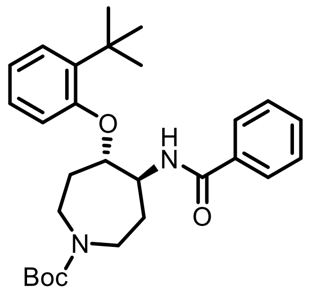

***tert*-Butyl (4*SR*,5*SR*)-4-benzamido-5-(2-tert-butylphenoxy)azepane-1-carboxylate (2p).** Followed General Procedure C-(a) with *tert*-butyl 8-benzoyl-4,8-diazabicyclo[5.1.0]octane-4-carboxylate (25 mg, 79.0 µmol) and 2-*tert*-butylphenol to give *tert*-butyl (4*SR*,5*SR*)-4-benzamido-5-(2-*tert*-butylphenoxy)azepane-1-carboxylate (25.3 mg, 69%) after purification by column chromatography (0–100% EtOAc in hexane) as a white solid. ^1H NMR (400 MHz, DMSO-d_6, 343 K): δ 8.20 (d, J = 8.0 Hz, 1H), 7.77–7.69 (m, 2H), 7.50–7.43 (m, 1H), 7.43–7.35 (m, 2H), 7.20 (dd, J = 7.7, 1.7 Hz, 1H), 7.19–7.13 (m, 1H), 7.00 (d, J = 8.1 Hz, 1H), 6.82 (td, J = 7.5, 1.2 Hz, 1H), 4.78–4.69 (m, 1H), 4.39 (ddq, J = 12.2, 8.3, 4.0 Hz, 1H), 3.72–3.61 (m, 1H), 3.50–3.44 (m, 2H), 3.39 (ddd, J = 14.5, 9.5, 2.8 Hz, 1H), 2.18 (dtd, J = 15.1, 5.1, 2.7 Hz, 1H), 2.04 (ddt, J = 14.1, 7.0, 3.3 Hz, 1H), 1.98–1.79 (m, 2H), 1.44 (s, 9H), 1.27 (s, 9H). $^{13}C\{^1H\}$ NMR (101 MHz, DMSO-d_6, 343 K): δ 165.7, 155.0, 154.2, 137.5, 134.8, 130.5, 127.7, 126.9, 126.6, 126.2, 119.5, 112.1, 78.4, 75.7, 52.6, 40.6, 34.0, 31.1, 29.5, 28.9, 27.9. (A δ 40.5 is an additional peak observed in HSQC, but not clear in $^{13}C\{^1H\}$ NMR.) HRMS (TOF, ES+) $C_{28}H_{38}N_2NaO_4$ $[M + Na]^+$ calcd mass 489.2724, found 489.2725.

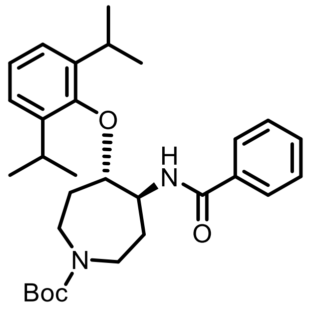

***tert*-Butyl (4*SR*,5*SR*)-4-benzamido-5-(2,6-diisopropylphenoxy)azepane-1-carboxylate (2q).** Followed General Procedure C-(a) with *tert*-butyl 8-benzoyl-4,8-diazabicyclo[5.1.0]octane-4-carboxylate (25 mg, 79.0 µmol) and 2,6-diisopropylphenol to give *tert*-butyl (4*SR*,5*SR*)-4-benzamido-5-(2,6-diisopropylphenoxy)azepane-1-carboxylate (32.1 mg, 82%) as a colorless oil after purification by column chromatography (0–100% EtOAc in hexane). ^1H NMR (400 MHz, DMSO-d_6, 343 K): δ 8.20 (d, J = 8.2 Hz, 1H), 7.84–7.78 (m, 2H), 7.52–7.47 (m, 1H), 7.46–7.40 (m, 2H), 7.10–6.99 (m, 3H), 4.41–4.31 (m, 1H), 4.19 (dt, J = 7.9, 5.7 Hz, 1H), 3.71–3.61 (m, 1H), 3.44 (dt, J = 14.1, 5.0 Hz, 1H), 3.40–3.22 (m, 4H), 2.04–1.96 (m, 2H), 1.87–1.80 (m, 2H),

1.40 (s, 9H), 1.13 (d, J = 2.9 Hz, 6H), 1.11 (d, J = 3.0 Hz, 6H). $^{13}C\{^1H\}$ NMR (101 MHz, DMSO-d_6, 343 K): δ 165.6, 154.1, 149.8, 141.6, 134.9, 130.5, 127.7, 126.9, 123.8, 123.8, 82.5, 78.3, 53.4, 40.8, 30.5, 28.7, 27.9, 25.7, 23.8, 23.1. (A δ 41.0 is an additional peak observed in HSQC, but not clear in $^{13}C\{^1H\}$ NMR.) HRMS (TOF, ES+) $C_{30}H_{43}N_2O_4$ $[M + H]^+$ calcd mass 495.3217, found 495.3218.

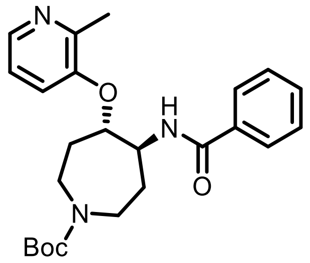

***tert*-Butyl (4*SR*,5*SR*)-4-benzamido-5-[(2-methyl-3-pyridyl)oxy]azepane-1-carboxylate (2r).** Followed General Procedure C-(a) with *tert*-butyl 8-benzoyl-4,8-diazabicyclo[5.1.0]octane-4-carboxylate (25 mg, 79.0 µmol) and 2-methyl-3-pyridinol to give *tert*-butyl (4*SR*,5*SR*)-4-benzamido-5-[(2-methyl-3-pyridyl)oxy]azepane-1-carboxylate (28.5 mg, 85%) as a colorless oil after purification by column chromatography (0–100% EtOAc in hexane). ^1H NMR (400 MHz, DMSO-d_6, 343 K): δ 8.14 (d, J = 8.2 Hz, 1H), 7.96 (dd, J = 4.7, 1.3 Hz, 1H), 7.76–7.67 (m, 2H), 7.53–7.45 (m, 1H), 7.45–7.34 (m, 3H), 7.15 (dd, J = 8.3, 4.7 Hz, 1H), 4.57 (td, J = 8.1, 2.6 Hz, 1H), 4.36 (qd, J = 8.2, 4.0 Hz, 1H), 3.72–3.59 (m, 1H), 3.58–3.49 (m, 1H), 3.49–3.34 (m, 2H), 2.26 (s, 3H), 2.22–2.13 (m, 1H), 2.08–1.92 (m, 2H), 1.92–1.81 (m, 1H), 1.44 (s, 9H). $^{13}C\{^1H\}$ NMR (101 MHz, DMSO-d_6, 343 K): δ 165.9, 154.2, 151.5, 148.1, 139.7, 134.7, 130.6, 127.7, 126.9, 121.5, 118.7, 78.4, 77.7, 52.1, 40.9, 30.4, 29.2, 27.9, 18.7. (A δ 40.1 is an additional peak observed in HSQC, but not clear in $^{13}C\{^1H\}$ NMR.) HRMS (TOF, ES+) $C_{24}H_{32}N_3O_4$ $[M + H]^+$ calcd mass 426.2387, found 426.2390.

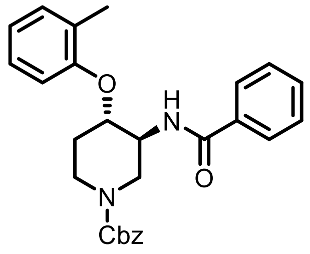

Benzyl (3*SR*,4*SR*)-3-benzamido-4-(o-tolyloxy)piperidine-1-carboxylate (4aa). Followed General Procedure C-(b) with benzyl 7-benzoyl-3,7-diazabicyclo[4.1.0]heptane-3-carboxylate (30.0 mg, 89.2 µmol) and *o*-cresol to give benzyl (3*SR*,4*SR*)-3-benzamido-4-(*o*-tolyloxy)piperidine-1-carboxylate as the main product [82% (determined by internal standard described in section 2.), Regio isomer ratio = 4.4 : 1]. Purification was conducted by column chromatography (0–100% EtOAc in hexane). Isolated as a white solid. ^1H NMR (400 MHz, DMSO-d_6, 343 K): δ 8.23 (d, J = 8.1 Hz, 1H), 7.79–7.62 (m, 2H), 7.54–7.47 (m, 1H), 7.45–7.39 (m, 2H), 7.39–7.35 (m, 4H), 7.35–7.26 (m, 1H), 7.17–7.03 (m, 3H), 6.82 (td, J = 7.0, 1.8 Hz,

1H), 5.13 (s, 2H), 4.61–4.51 (m, 1H), 4.23–4.13 (m, 1H), 3.98 (dd, *J* = 13.2, 2.9 Hz, 1H), 3.93–3.81 (m, 1H), 3.30–3.18 (m, 2H), 2.31–2.21 (m, 1H), 2.09 (s, 3H), 1.67–1.54 (m, 1H). $^{13}C\{^{1}H\}$ NMR (101 MHz, DMSO-d_6, 343 K): δ 166.4, 155.4, 154.3, 136.7, 134.4, 130.8, 130.3, 128.1, 127.8, 127.5, 127.1, 126.9, 126.7, 126.4, 120.3, 113.4, 75.2, 66.1, 49.6, 45.4, 40.9, 28.9, 15.5. HRMS (TOF, ES+) $C_{27}H_{29}N_2O_4$ $[M + H]^+$ calcd mass 445.2122, found 445.2126.

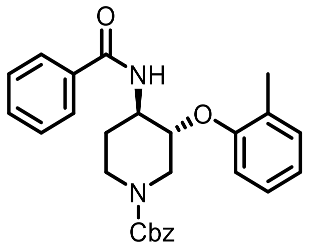

Benzyl (3*RS*,4*RS*)-4-benzamido-3-(*o*-tolyloxy)piperidine-1-carboxylate (4ab). Isolated as a white solid.^{1}H NMR (400 MHz, DMSO-d_6, 343 K): δ 8.22 (d, *J* = 7.0 Hz, 1H), 7.78–7.73 (m, 2H), 7.52–7.46 (m, 1H), 7.45–7.30 (m, 7H), 7.11–7.03 (m, 3H), 6.82 (td, *J* = 7.1, 1.8 Hz, 1H), 5.16 (d, *J* = 12.6 Hz, 1H), 5.07 (d, *J* = 12.6 Hz, 1H), 4.40–4.30 (m, 2H), 4.16 (d, *J* = 11.9 Hz, 1H), 3.94–3.85 (m, 1H), 3.32–3.23 (m, 2H), 2.06 (s, 3H), 2.03–1.94 (m, 1H), 1.74–1.62 (m, 1H). $^{13}C\{^{1}H\}$ NMR (101 MHz, DMSO-d_6, 343 K): δ 166.2, 155.4, 154.4, 136.5, 134.6, 130.7, 130.4, 128.1, 127.8, 127.5, 127.2, 126.9, 126.6, 126.4, 120.5, 112.7, 74.0, 66.2, 49.7, 45.4, 41.7, 28.9, 15.3. HRMS (TOF, ES+) $C_{27}H_{29}N_2O_4$ $[M + H]^+$ calcd mass 445.2122, found 445.2123.

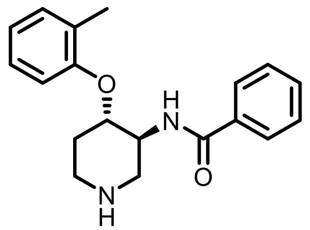

***N*-((3*SR*,4*SR*)-4-(*o*-Tolyloxy)piperidin-3-yl)benzamide (e).** To a flask containing benzyl (3*SR*,4*SR*)-3-benzamido-4-(*o*-tolyloxy)piperidine-1-carboxylate (17.7 mg, 39.8 µmol) was added MeOH (1.00 mL) followed by Pd/C (10 % w/v, 8.9 mg). A hydrogen balloon was added, and the flask was vacuum purged. The mixture was allowed to stir for 4 h at rt. The mixture was filtered through a Celite pad and concentrated. The crude material was purified by RP-HPLC (15–45% MeCN in 0.05% aqueous NH_4OH) and concentrated to give *N*-((3*SR*,4*SR*)-4-(*o*-tolyloxy)pyrrolidin-3-yl)benzamide (9.2 mg, 74%) as a white solid. ^{1}H NMR (400 MHz, $CDCl_3$, 298 K): δ = 7.87–7.80 (m, 2H), 7.56–7.49 (m, 1H), 7.49–7.41 (m, 2H), 7.30–7.11 (m, 4H), 6.86 (td, *J*=7.3, 1.3, 1H), 4.69–4.60 (m, 1H), 4.42–4.32 (m, 1H), 3.44–3.36 (m, 1H), 3.20–3.08 (m, 1H), 2.89–2.76 (m, 2H), 2.27 (s, 3H), 2.13–2.00 (m, 1H), 1.90–1.78 (m, 1H). $^{13}C\{^{1}H\}$ NMR (101 MHz, $CDCl_3$, 298 K): δ 167.0, 155.4, 134.5, 131.7, 130.9, 128.7, 127.3, 127.2, 127.2, 120.6, 112.6, 71.1, 47.7, 46.7, 42.3, 28.0, 16.6. HRMS (TOF, ES+) $C_{19}H_{23}N_2O_2$ $[M + H]^+$ calcd mass 311.1754, found 311.1757.

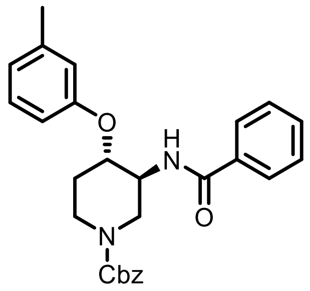

Benzyl (3*SR*,4*SR*)-3-benzamido-4-(*m*-tolyloxy)piperidine-1-carboxylate (4ba). Followed General Procedure C-(b) with benzyl 7-benzoyl-3,7-diazabicyclo[4.1.0]heptane-3-carboxylate (30.0 mg, 89.2 µmol) and *m*-cresol to give benzyl (3*SR*,4*SR*)-3-benzamido-4-(*m*-tolyloxy)piperidine-1-carboxylate as the main product [32.5 mg, 81%, Regio isomer ratio = 5.6 : 1 (determined by LCMS)]. Purification was conducted by RP-HPLC (55-95% MeCN in 0.05% aqueous NH_4OH). Isolated as a white solid. ^{1}H NMR (400 MHz, MeOD, 298 K): δ 7.67–7.60 (m, 2H), 7.53–7.44 (m, 1H), 7.42–7.25 (m, 7H), 7.10 (t, *J* = 7.5 Hz, 1H), 6.84–6.77 (m, 2H), 6.73 (d, *J* = 7.5 Hz, 1H), 5.16–5.11 (m, 2H), 4.58–4.50 (m, 1H), 4.22 (td, *J* = 8.3, 4.4 Hz, 1H), 4.08 (dd, *J* = 13.6, 3.9 Hz, 1H), 3.99–3.89 (m, 1H), 3.30–3.22 (m, 2H), 2.26–2.16 (m, 4H), 1.73–1.59 (m, 1H). ^{13}C{^{1}H} NMR (101 MHz, MeOD, 298 K): δ 170.6, 159.2, 157.0, 140.8, 138.0, 135.6, 132.7, 130.3, 129.6, 129.4, 129.1, 128.9, 128.4, 123.3, 118.3, 114.4, 76.5, 68.5, 51.8, 46.6, 42.4, 30.3, 21.5. HRMS (TOF, ES+) $C_{27}H_{29}N_2O_4$ $[M + H]^+$ calcd mass 445.2122, found 445.2122.

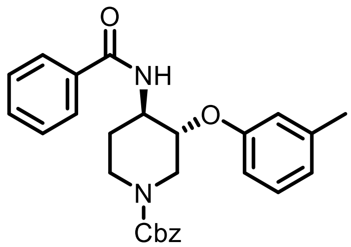

Benzyl (3*RS*,4*RS*)-4-benzamido-3-(*m*-tolyloxy)piperidine-1-carboxylate (4bb). Isolated as a white solid. ^{1}H NMR (400 MHz, DMSO-d_6, 343 K): δ 8.22 (d, *J* = 8.0 Hz, 1H), 7.79–7.70 (m, 2H), 7.52–7.46 (m, 1H), 7.45–7.39 (m, 2H), 7.38–7.29 (m, 5H), 7.10 (t, *J* = 7.8 Hz, 1H), 6.81–6.70 (m, 3H), 5.15 (d, *J* = 12.6 Hz, 1H), 5.09 (d, *J* = 12.6 Hz, 1H), 4.39–4.32 (m, 1H), 4.31–4.21 (m, 1H), 4.16 (dd, *J* = 13.4, 2.7 Hz, 1H), 3.96–3.86 (m, 1H), 3.26–3.15 (m, 1H), 3.13–3.09 (m, 1H), 2.21 (s, 3H), 2.01–1.92 (m, 1H), 1.72–1.60 (m, 1H). ^{13}C{^{1}H} NMR (101 MHz, DMSO-d_6, 343 K): δ 166.3, 157.6, 154.6, 138.9, 136.8, 134.8, 130.9, 129.1, 128.3, 128.0, 127.7, 127.3, 127.1, 121.9, 116.7, 112.9, 74.1, 66.4, 50.2, 45.7, 41.9, 29.2, 20.8. HRMS (TOF, ES+) $C_{27}H_{29}N_2O_4$ $[M + H]^+$ calcd mass 445.2122, found 445.2123.

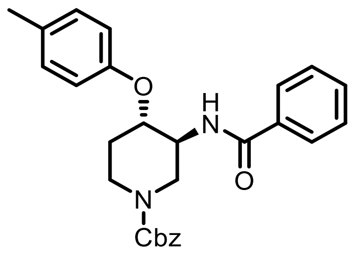

Benzyl (3*SR*,4*SR*)-3-benzamido-4-(*p*-tolyloxy)piperidine-1-carboxylate (4ca). Followed General Procedure C-(b) with benzyl 7-benzoyl-3,7-diazabicyclo[4.1.0]heptane-3-carboxylate (30.0 mg, 89.2 μmol) and *p*-cresol to give benzyl (3*SR*,4*SR*)-3-benzamido-4-(*p*-tolyloxy)piperidine-1-carboxylate as the main product [31.5 mg, 79%, Regio isomer ratio = 5.6 : 1 (determined by LCMS)]. Purification was conducted by RP-HPLC (50–90% MeCN in 0.05% aqueous NH_4OH). Isolated as a white solid. ^1H NMR (400 MHz, MeOD, 298 K): δ 7.67–7.60 (m, 2H), 7.52–7.44 (m, 1H), 7.42–7.26 (m, 7H), 7.03 (d, J = 8.5 Hz, 2H), 6.89 (d, J = 8.5 Hz, 2H), 5.13 (s, 2H), 4.55–4.46 (m, 1H), 4.26–4.17 (m, 1H), 4.07 (dd, J = 13.4, 3.8 Hz, 1H), 3.97–3.89 (m, 1H), 3.29–3.22 (m, 2H), 2.22 (s, 3H), 2.17 (s, 1H), 1.71–1.58 (m, 1H). $^{13}C\{^1H\}$ NMR (101 MHz, MeOD, 298 K): δ 170.6, 157.0, 138.0, 135.6, 132.6, 131.9, 131.0, 129.6, 129.4, 129.1, 128.9, 128.4, 117.5, 76.6, 68.5, 51.8, 46.6, 42.4, 30.2, 20.6. HRMS (TOF, ES+) $C_{27}H_{29}N_2O_4$ $[M + H]^+$ calcd mass 445.2122, found 445.2123.

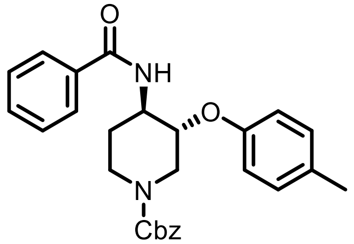

Benzyl (3*RS*,4*RS*)-4-benzamido-3-(*p*-tolyloxy)piperidine-1-carboxylate (4cb). Isolated as a white solid. ^1H NMR (400 MHz, DMSO-d_6, 343 K): δ 8.21 (d, J = 7.7 Hz, 1H), 7.79–7.72 (m, 2H), 7.53–7.29 (m, 8H), 7.03 (d, J = 8.2 Hz, 2H), 6.88–6.81 (m, 2H), 5.14 (d, J = 12.6 Hz, 1H), 5.08 (d, J = 12.6 Hz, 1H), 4.35–4.21 (m, 2H), 4.13 (dd, J = 13.3, 3.0 Hz, 1H), 3.95–3.85 (m, 1H), 3.26–3.14 (m, 1H), 3.13–3.09 (m, 1H), 2.21 (s, 3H), 2.01–1.92 (m, 1H), 1.71–1.58 (m, 1H). $^{13}C\{^1H\}$ NMR (101 MHz, DMSO-d_6, 343 K): δ 166.1, 155.3, 154.3, 136.5, 134.6, 130.7, 129.8, 129.5, 128.1, 127.8, 127.5, 127.2, 126.9, 115.8, 74.2, 66.2, 49.9, 45.4, 41.7, 29.0, 19.7. HRMS (TOF, ES+) $C_{27}H_{29}N_2O_4$ $[M + H]^+$ calcd mass 445.2122, found 445.2123

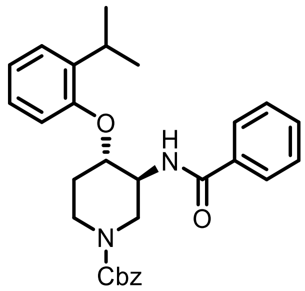

Benzyl (3*SR*,4*SR*)-3-benzamido-4-(2-isopropylphenoxy)piperidine-1-carboxylate (4da). Followed General Procedure C-(b) with benzyl (3*SR*,4*SR*)-3-benzamido-4-(2-isopropylphenoxy)piperidine-1-carboxylate (30.0 mg, 89.2 µmol) and 2-isopropylphenol to give benzyl (3*SR*,4*SR*)-3-benzamido-4-(*p*-tolyloxy)piperidine-1-carboxylate as the main product [32.2 mg, 76%, Regio isomer ratio = 7.2 : 1 (determined by LCMS)]. Purification was conducted by RP-HPLC (60–100% MeCN in 0.05% aqueous NH_4OH). Isolated as a white solid. ^{1}H NMR (400 MHz, MeOD, 298 K): δ 7.68–7.62 (m, 2H), 7.51–7.45 (m, 1H), 7.41–7.25 (m, 7H), 7.17–7.09 (m, 2H), 7.09–7.03 (m, 1H), 6.88 (td, *J* = 7.4, 1.3 Hz, 1H), 5.14 (s, 2H), 4.65–4.57 (m, 1H), 4.35–4.27 (m, 1H), 4.16–4.08 (m, 1H), 4.02–3.93 (m, 1H), 3.37–3.22 (m, 3H), 2.34–2.20 (m, 1H), 1.73–1.61 (m, 1H), 1.14 (d, *J* = 6.9 Hz, 3H), 1.05 (d, *J* = 6.9 Hz, 3H). ^{13}C{^{1}H} NMR (101 MHz, MeOD, 298 K): δ 170.5, 157.0, 155.8, 138.8, 138.0, 135.6, 132.7, ff129.6, 129.4, 129.2, 128.9, 128.4, 127.6, 127.4, 122.1, 113.8, 75.9, 68.6, 51.9, 46.8, 42.5, 30.3, 27.9, 23.3, 23.2. HRMS (TOF, ES+) $C_{29}H_{33}N_2O_4$ $[M + H]^+$ calcd mass 473.2435, found 473.2434.

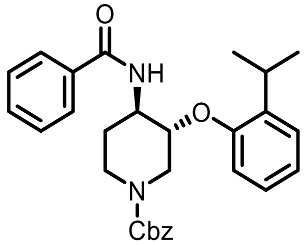

Benzyl (3*RS*,4*RS*)-4-benzamido-3-(2-isopropylphenoxy)piperidine-1-carboxylate (4db). Isolated as a white solid. ^{1}H NMR (400 MHz, DMSO-d_6, 343 K): δ 8.24 (d, *J* = 7.4 Hz, 1H), 7.80–7.73 (m, 2H), 7.52–7.45 (m, 1H), 7.45–7.29 (m, 7H), 7.16–7.12 (m, 1H), 7.11–6.98 (m, 2H), 6.91–6.85 (m, 1H), 5.16 (d, *J* = 12.5 Hz, 1H), 5.06 (d, *J* = 12.5 Hz, 1H), 4.45–4.30 (m, 2H), 4.16 (dd, *J* = 13.0, 2.3 Hz, 1H), 3.95–3.85 (m, 1H), 3.35–3.15 (m, 2H), 3.13–3.09 (m, 1H), 2.04–1.95 (m, 1H), 1.74–1.62 (m, 1H), 1.09 (d, *J* = 6.9 Hz, 3H), 1.00 (d, *J* = 6.9 Hz, 3H). ^{13}C{^{1}H} NMR (101 MHz, DMSO-d_6, 343 K): δ 166.1, 154.4, 154.1, 136.7, 136.5, 134.5, 130.7, 128.1, 127.7, 127.5, 127.2, 126.9, 126.2, 125.9, 120.6, 112.1, 73.2, 66.2, 49.6, 45.2, 41.6, 29.0, 26.0, 22.2, 22.2. HRMS (TOF, ES+) $C_{29}H_{33}N_2O_4$ $[M + H]^+$ calcd mass 473.2435, found 473.2432.

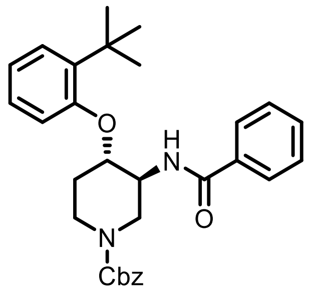

Benzyl (3*SR*,4*SR*)-3-benzamido-4-(2-(*tert*-butyl)phenoxy)piperidine-1-carboxylate (4ea). Followed General Procedure C-(b) with benzyl (3*SR*,4*SR*)-3-benzamido-4-(2-isopropylphenoxy)piperidine-1-carboxylate (30.0 mg, 89.2 µmol) and 2-*tert*-butylphenol to give benzyl (3*SR*,4*SR*)-3-benzamido-4-(*p*-tolyloxy)piperidine-1-carboxylate as the main product [26.4 mg, 61%, Regio isomer ratio = 4.7 : 1 (determined by LCMS)]. Purification was conducted by RP-HPLC (60–100% MeCN in 0.05% aqueous NH_4OH). Isolated as a white solid. ^{1}H NMR (400 MHz, DMSO-d_6, 343 K): δ 8.33 (d, J = 8.0 Hz, 1H), 7.78–7.69 (m, 2H), 7.52–7.27 (m, 8H), 7.24–7.07 (m, 3H), 6.86–6.79 (m, 1H), 5.19–5.08 (m, 2H), 4.77 (td, J = 8.8, 4.1 Hz, 1H), 4.30–4.19 (m, 1H), 4.14–4.04 (m, 1H), 3.98–3.88 (m, 1H), 3.30–3.20 (m, 1H), 3.13–3.10 (m, 1H), 2.38–2.28 (m, 1H), 1.58–1.46 (m, 1H), 1.28 (s, 9H). ^{13}C{^{1}H} NMR (101 MHz, DMSO-d_6, 343 K): δ 166.4, 155.0, 154.3, 137.3, 136.7, 134.5, 130.7, 128.0, 127.7, 127.5, 127.1, 126.9, 126.5, 126.2, 119.6, 112.1, 72.9, 66.2, 49.9, 45.7, 41.0, 34.0, 29.5, 28.3. HRMS (TOF, ES+) $C_{30}H_{35}N_2O_4$ $[M + H]^+$ calcd mass 487.2591, found 487.2591.

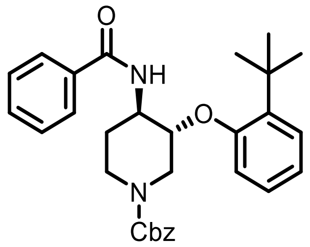

Benzyl (3*RS*,4*RS*)-4-benzamido-3-(2-(*tert*-butyl)phenoxy)piperidine-1-carboxylate (4eb). Isolated as a white solid. ^{1}H NMR (400 MHz, DMSO-d_6, 343 K): δ 8.29 (d, J = 7.8 Hz, 1H), 7.80–7.73 (m, 2H), 7.52–7.45 (m, 1H), 7.44–7.29 (m, 7H), 7.21 (d, J = 7.7 Hz, 1H), 7.15–6.97 (m, 2H), 6.88–6.79 (m, 1H), 5.16 (d, J = 12.5 Hz, 1H), 5.06 (d, J = 12.5 Hz, 1H), 4.61–4.52 (m, 1H), 4.46–4.35 (m, 1H), 4.18 (dd, J = 13.5, 3.3 Hz, 1H), 3.94–3.84 (m, 1H), 3.39–3.29 (m, 1H), 3.28–3.17 (m, 1H), 2.11–2.00 (m, 1H), 1.74–1.61 (m, 1H), 1.27 (s, 9H). ^{13}C{^{1}H} NMR (101 MHz, DMSO-d_6, 343 K): δ 166.2, 155.0, 154.3, 137.2, 136.4, 134.6, 130.6, 128.1, 127.7, 127.6, 127.3, 126.9, 126.5, 126.3, 119.8, 111.4, 71.4, 66.3, 49.4, 44.6, 41.4, 34.0, 29.4, 29.0. HRMS (TOF, ES+) $C_{30}H_{35}N_2O_4$ $[M + H]^+$ calcd mass 487.2591, found 487.2591.

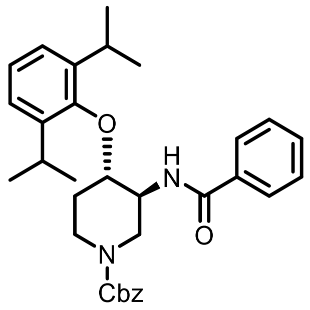

Benzyl (3*SR*,4*SR*)-3-benzamido-4-(2,6-diisopropylphenoxy)piperidine-1-carboxylate (4fa). Followed General Procedure C-(b) with benzyl (3*SR*,4*SR*)-3-benzamido-4-(2-isopropylphenoxy)piperidine-1-carboxylate (30.0 mg, 89.2 µmol) and 2,6-diisopropylphenol to give benzyl (3*SR*,4*SR*)-3-benzamido-4-(2,6-diisopropylphenoxy)piperidine-1-carboxylate as the main product [25.7 mg, 56%, Regio isomer ratio = 5.1 : 1 (determined by LCMS)]. Purification was conducted by RP-HPLC (60–100% MeCN in 0.05% aqueous NH_4OH). Isolated as a white solid. ^{1}H NMR (400 MHz, DMSO-d_6, 298 K): δ 8.52 (d, J = 8.3 Hz, 1H), 7.84–7.77 (m, 2H), 7.56–7.50 (m, 1H), 7.50–7.43 (m, 2H), 7.41–7.28 (m, 5H), 7.11–7.00 (m, 3H), 5.16–5.04 (m, 2H), 4.27–4.07 (m, 2H), 4.05–3.82 (m, 2H), 3.42–3.04 (m, 3H), 2.92 (t, J = 10.9 Hz, 1H), 1.91–1.76 (m, 1H), 1.59–1.46 (m, 1H), 1.17–1.02 (m, 12H). ^{13}C{^{1}H} NMR (101 MHz, DMSO-d_6, 298 K): δ 166.4, 154.4, 150.1, 141.6, 136.9, 134.4, 131.3, 128.4, 128.2, 127.9, 127.6, 127.4, 124.3, 123.8, 80.1, 66.4, 50.7, 45.6, 41.6, 29.1, 26.0, 24.2, 23.7. HRMS (TOF, ES+) $C_{32}H_{39}N_2O_4$ [M + H]$^+$ calcd mass 515.2904, found 515.2902.

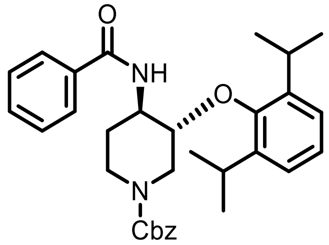

Benzyl (3*RS*,4*RS*)-4-benzamido-3-(2,6-diisopropylphenoxy)piperidine-1-carboxylate (4fb). Isolated as a white solid. ^{1}H NMR (400 MHz, DMSO-d_6, 343 K): δ 8.27 (d, J = 8.5 Hz, 1H), 7.85–7.78 (m, 2H), 7.54–7.48 (m, 1H), 7.48–7.41 (m, 2H), 7.36–7.15 (m, 5H), 7.12–7.02 (m, 3H), 5.09–4.97 (m, 2H), 4.44–4.32 (m, 1H), 4.06–3.87 (m, 3H), 3.47–3.33 (m, 2H), 3.20–3.04 (m, 2H), 1.99–1.89 (m, 1H), 1.87–1.75 (m, 1H), 1.09 (t, J = 7.3 Hz, 12H). ^{13}C{^{1}H} NMR (101 MHz, DMSO-d_6, 343 K): δ 165.8, 154.1, 150.0, 141.1, 136.4, 134.6, 130.7, 128.0, 127.7, 127.4, 127.0, 126.8, 124.0, 123.5, 78.4, 66.0, 50.8, 45.7, 41.9, 29.2, 25.6, 23.7, 23.2. HRMS (TOF, ES+) $C_{32}H_{38}N_2NaO_4$ [M + Na]$^+$ calcd mass 537.2724, found 537.2722.

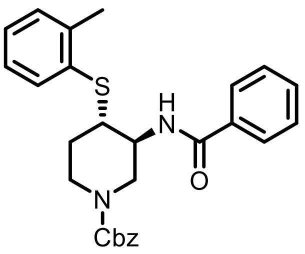

Benzyl (3*SR*,4*SR*)-3-benzamido-4-(*o*-tolylthio)piperidine-1-carboxylate (4ga). Followed General Procedure C-(b) with benzyl (3*SR*,4*SR*)-3-benzamido-4-(2-isopropylphenoxy)piperidine-1-carboxylate (30.0 mg, 89.2 µmol) and *o*-thiocresol to give benzyl (3*SR*,4*SR*)-3-benzamido-4-(*o*-tolylthio)piperidine-1-carboxylate as the main product [36.1 mg, 88%, Regio isomer ratio = 5.7 : 1 (determined by LCMS)]. Purification was conducted by RP-HPLC (23–63% MeCN in 0.05% aqueous NH_4OH). Isolated as a white solid. ^{1}H NMR (400 MHz, DMSO-d_6, 343 K): δ 8.29 (d, J = 8.5 Hz, 1H), 7.80–7.75 (m, 2H), 7.57–7.40 (m, 4H), 7.39–7.27 (m, 5H), 7.23–7.12 (m, 3H), 5.15–5.05 (m, 2H), 4.17–4.07 (m, 1H), 4.01–3.89 (m, 2H), 3.51 (td, J = 10.7, 4.1 Hz, 1H), 3.05–2.89 (m, 2H), 2.33 (s, 3H), 2.12–2.01 (m, 1H), 1.57–1.43 (m, 1H). ^{13}C{^{1}H} NMR (101 MHz, DMSO-d_6, 343 K): δ 166.0, 154.1, 139.4, 136.6, 134.2, 132.5, 132.4, 130.8, 129.9, 128.0, 127.8, 127.4, 127.1, 127.0, 126.9, 126.1, 66.1, 50.0, 47.8, 47.3, 42.9, 31.3, 20.1. HRMS (TOF, ES+) $C_{27}H_{29}N_2O_3S$ $[M + H]^+$ calcd mass 461.1893, found 461.1892.

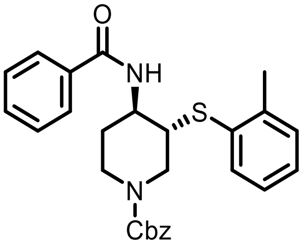

Benzyl (3*RS*,4*RS*)-4-benzamido-3-(o-tolylthio)piperidine-1-carboxylate (4gb). Isolated as a white solid. ^{1}H NMR (400 MHz, DMSO-d_6, 343 K): δ 8.31 (d, J = 8.6 Hz, 1H), 7.85–7.78 (m, 2H), 7.55–7.49 (m, 1H), 7.48–7.40 (m, 3H), 7.40–7.24 (m, 5H), 7.21 (d, J = 7.8 Hz, 1H), 7.18–7.12 (m, 1H), 7.12–7.02 (m, 1H), 5.11 (d, J = 12.6 Hz, 1H), 5.03 (d, J = 12.6 Hz, 1H), 4.18–4.06 (m, 2H), 4.04–3.96 (m, 1H), 3.39 (td, J = 10.7, 4.3 Hz, 1H), 3.11–2.95 (m, 2H), 2.30 (s, 3H), 2.02–1.92 (m, 1H), 1.74–1.59 (m, 1H). ^{13}C{^{1}H} NMR (101 MHz, DMSO-d_6, 343 K): δ 165.6, 153.9, 138.6, 136.4, 134.4, 132.7, 130.8, 130.7, 130.0, 128.1, 127.9, 127.5, 127.1, 126.9, 126.6, 126.2, 66.1, 49.8, 48.1, 48.0, 42.5, 31.6, 19.9. HRMS (TOF, ES+) $C_{27}H_{29}N_2O_3S$ $[M + H]^+$ calcd mass 461.1893, found 461.1895.

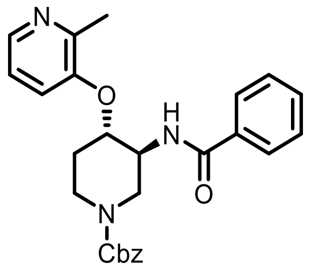

Benzyl (3*SR*,4*SR*)-3-benzamido-4-((2-methylpyridin-3-yl)oxy)piperidine-1-carboxylate (4ha). Followed General Procedure C-(b) with benzyl (3*SR*,4*SR*)-3-benzamido-4-(2-isopropylphenoxy)piperidine-1-carboxylate (30.0 mg, 89.2 µmol) and 2-methyl-3-pyridinol to give benzyl (3*SR*,4*SR*)-3-benzamido-4-((2-methylpyridin-3-yl)oxy)piperidine-1-carboxylate as a main product [29.8 mg, 75%, Regio isomer ratio = 5.7 : 1 (determined by LCMS)]. Purification was conducted by RP-HPLC (29–69% MeCN in 0.05% aqueous NH_4OH). Isolated as a white solid. ^{1}H NMR (400 MHz, MeOD, 298 K): δ 7.94 (dd, *J* = 4.9, 1.3 Hz, 1H), 7.67–7.55 (m, 3H), 7.54–7.46 (m, 1H), 7.43–7.26 (m, 7H), 7.22 (dd, *J* = 8.4, 4.9 Hz, 1H), 5.16 (s, 2H), 4.69–4.60 (m, 1H), 4.35–4.26 (m, 1H), 4.17–4.07 (m, 1H), 4.05–3.95 (m, 1H), 3.36–3.27 (m, 2H), 2.37 (s, 3H), 2.33–2.20 (m, 1H), 1.82–1.68 (m, 1H). ^{13}C{^{1}H} NMR (101 MHz, MeOD, 298 K): δ 170.6, 157.0, 154.0, 150.2, 140.9, 138.0, 135.5, 132.8, 129.6, 129.5, 129.2, 129.0, 128.3, 123.6, 121.8, 76.9, 68.6, 51.7, 46.7, 42.4, 30.3, 18.9. HRMS (TOF, ES+) $C_{26}H_{28}N_3O_4$ $[M + H]^+$ calcd mass 446.2074, found 446.2077.

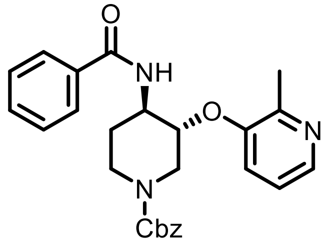

Benzyl (3*RS*,4*RS*)-4-benzamido-3-((2-methylpyridin-3-yl)oxy)piperidine-1-carboxylate (4hb). Isolated as a white solid. ^{1}H NMR (400 MHz, DMSO-d_6, 343 K): δ 8.23 (d, *J* = 7.5 Hz, 1H), 8.02–7.96 (m, 1H), 7.78–7.71 (m, 2H), 7.53–7.47 (m, 1H), 7.46–7.28 (m, 8H), 7.19–7.01 (m, 1H), 5.16 (d, *J* = 12.5 Hz, 1H), 5.07 (d, *J* = 12.5 Hz, 1H), 4.44–4.29 (m, 2H), 4.13–4.04 (m, 1H), 3.93–3.83 (m, 1H), 3.39–3.27 (m, 2H), 2.26 (s, 3H), 2.05–1.94 (m, 1H), 1.75–1.63 (m, 1H). ^{13}C{^{1}H} NMR (101 MHz, DMSO-d_6, 343 K): δ 166.3, 154.4, 151.5, 148.2, 140.3, 136.5, 134.5, 130.8, 128.1, 127.8, 127.6, 127.2, 126.9, 121.5, 119.1, 73.9, 66.2, 49.4, 45.1, 41.5, 28.6, 18.5. HRMS (TOF, ES+) $C_{26}H_{28}N_3O_4$ $[M + H]^+$ calcd mass 446.2074, found 446.2074.

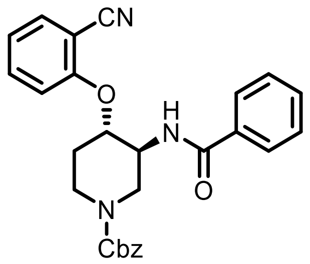

Benzyl (3*SR*,4*SR*)-3-benzamido-4-(2-cyanophenoxy)piperidine-1-carboxylate (4ia). Followed General Procedure C-(b) with benzyl (3*SR*,4*SR*)-3-benzamido-4-(2-isopropylphenoxy)piperidine-1-carboxylate (30.0 mg, 89.2 µmol) and 2-cyanophenol to give benzyl (3*SR*,4*SR*)-3-benzamido-4-(2-cyanophenoxy)piperidine-1-carboxylate as a main product [33.6 mg, 83%, Regio isomer ratio = 5.2 : 1 (determined by LCMS)]. Purification was conducted by RP-HPLC (26–66% MeCN in 0.05% aqueous NH_4OH). Isolated as a white solid. ^1H NMR (400 MHz, MeOD, 298 K): δ 7.66 (d, J = 7.6 Hz, 2H), 7.62–7.54 (m, 2H), 7.54–7.45 (m, 1H), 7.43–7.24 (m, 8H), 7.05 (m, 1H), 5.14 (s, 2H), 4.85–4.78 (m, 1H), 4.28 (td, J = 7.3, 4.1 Hz, 1H), 4.09–3.95 (m, 1H), 3.93–3.81 (m, 1H), 3.54–3.39 (m, 2H), 2.24 (m, 1H), 1.79 (m, 1H). $^{13}C\{^1H\}$ NMR (101 MHz, MeOD, 298 K): δ 170.6, 160.8, 157.1, 137.9, 135.9, 135.5, 134.9, 132.7, 129.6, 129.5, 129.2, 128.9, 128.4, 122.8, 117.2, 115.8, 103.8, 76.6, 68.6, 51.1, 46.1, 41.9, 29.5. HRMS (TOF, ES+) $C_{27}H_{26}N_3O_4$ $[M + H]^+$ calcd mass 456.1918, found 456.1916.

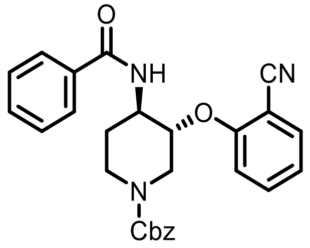

Benzyl (3*SR*,4*SR*)-3-benzamido-4-(2-cyanophenoxy)piperidine-1-carboxylate (4ib). Isolated as a white solid. ^1H NMR (400 MHz, DMSO-d_6, 343 K): δ 8.28 (d, J = 7.7 Hz, 1H), 7.79–7.72 (m, 2H), 7.65 (dd, J = 7.7, 1.7 Hz, 1H), 7.62–7.54 (m, 1H), 7.53–7.47 (m, 1H), 7.46–7.27 (m, 8H), 7.12–7.04 (m, 1H), 5.14 (d, J = 12.5 Hz, 1H), 5.07 (d, J = 12.5 Hz, 1H), 4.68–4.61 (m, 1H), 4.38–4.27 (m, 1H), 4.10–4.01 (m, 1H), 3.88–3.80 (m, 1H), 3.51–3.29 (m, 2H), 2.11–2.00 (m, 1H), 1.76–1.63 (m, 1H). $^{13}C\{^1H\}$ NMR (101 MHz, DMSO-d_6, 343 K): δ 166.3, 158.9, 154.4, 136.5, 134.4, 134.4, 133.5, 130.8, 128.1, 127.8, 127.5, 127.2, 127.0, 121.4, 115.5, 114.2, 101.8, 74.3, 66.3, 49.2, 44.6, 41.1, 28.2. HRMS (TOF, ES+) $C_{27}H_{26}N_3O_4$ $[M + H]^+$ calcd mass 456.1918, found 456.1916.

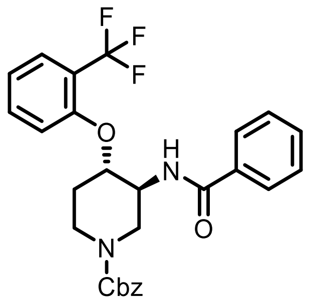

Benzyl (3*SR*,4*SR*)-3-benzamido-4-(2-(trifluoromethyl)phenoxy)piperidine-1-carboxylate (4ja). Followed General Procedure C-(b) with benzyl (3*SR*,4*SR*)-3-benzamido-4-(2-isopropylphenoxy)piperidine-1-carboxylate (30.0 mg, 89.2 μmol) and 2-(trifluoromethyl)phenol to give benzyl (3*SR*,4*SR*)-3-benzamido-4-(2-cyanophenoxy)piperidine-1-carboxylate as a main product [36.8 mg, 83%, Regio isomer ratio = 5.9 : 1 (determined by LCMS)]. Purification was conducted by RP-HPLC (37–77% MeCN in 0.05% aqueous NH_4OH). Isolated as a white solid. ^1H NMR (400 MHz, MeOD, 298 K): δ 7.71–7.64 (m, 2H), 7.60–7.54 (m, 2H), 7.53–7.47 (m, 1H), 7.46–7.24 (m, 8H), 7.05 (t, *J* = 7.6 Hz, 1H), 5.15 (s, 2H), 4.86–4.79 (m, 1H), 4.32–4.23 (m, 1H), 3.95 (d, *J* = 13.2 Hz, 1H), 3.84–3.73 (m, 1H), 3.59–3.50 (m, 2H), 2.36–2.14 (m, 1H), 1.83–1.70 (m, 1H). $^{13}C\{^1H\}$ NMR (101 MHz, MeOD, 298 K): δ 170.7, 157.2, 156.6, 138.0, 135.6, 134.8, 132.7, 129.6, 129.5, 129.2, 128.9, 128.4, 128.1 (q, *J* = 5.3 Hz), 125.2 (q, *J* = 271.9 Hz), 121.6, 120.3 (q, *J* = 30.1 Hz), 115.2, 75.1, 68.6, 50.9, 45.9, 41.7, 29.0. HRMS (TOF, ES+) $C_{27}H_{26}F_3N_2O_4$ $[M + H]^+$ calcd mass 499.1839, found 499.1838.

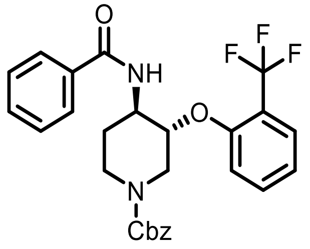

Benzyl (3*RS*,4*RS*)-4-benzamido-3-(2-(trifluoromethyl)phenoxy)piperidine-1-carboxylate (4jb). Isolated as a white solid. ^1H NMR (400 MHz, DMSO-d_6, 343 K): δ 8.27 (d, *J* = 7.6 Hz, 1H), 7.81–7.73 (m, 2H), 7.60–7.47 (m, 3H), 7.46–7.24 (m, 8H), 7.08 (t, *J* = 7.6 Hz, 1H), 5.13 (d, *J* = 12.5 Hz, 1H), 5.02 (d, *J* = 12.5 Hz, 1H), 4.68–4.60 (m, 1H), 4.36–4.26 (m, 1H), 4.08–3.99 (m, 1H), 3.87–3.75 (m, 1H), 3.48–3.28 (m, 2H), 2.10–1.98 (m, 1H), 1.74–1.61 (m, 1H). $^{13}C\{^1H\}$ NMR (101 MHz, DMSO-d_6, 343 K): δ 166.3, 155.0, 154.4, 136.5, 134.5, 133.6, 130.7, 128.1, 127.8, 127.5, 127.2, 127.0, 126.6 (q, *J* = 5.2 Hz), 123.3 (q, *J* = 273.3 Hz), 120.3, 117.7 (q, *J* = 30.3 Hz), 114.0, 73.3, 66.2, 48.9, 44.4, 41.0, 28.1. HRMS (TOF, ES+) $C_{27}H_{26}F_3N_2O_4$ $[M + H]^+$ calcd mass 499.1839, found 499.1840.

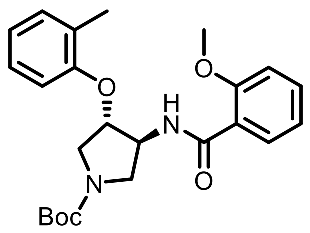

tert-Butyl (3*SR*,4*SR*)-3-(2-methoxybenzamido)-4-(*o*-tolyloxy)pyrrolidine-1-carboxylate (6a). Followed General Procedure C-(a) with *tert*-butyl 6-(2-methoxybenzoyl)-3,6-diazabicyclo[3.1.0]hexane-3-carboxylate (28.4 mg, 89.2 μmol) and *o*-cresol to give *tert*-butyl (3*SR*,4*SR*)-3-(2-methoxybenzamido)-4-(*o*-tolyloxy)pyrrolidine-1-carboxylate (35.8 mg, 94%) as a white solid after purification by column chromatography (0–100% EtOAc in hexane). ^{1}H NMR (400 MHz, DMSO-d_6, 343 K): δ 8.27 (d, *J* = 6.7 Hz, 1H), 7.67 (dt, *J* = 7.6, 1.9 Hz, 1H), 7.51–7.43 (m, 1H), 7.25–7.09 (m, 4H), 7.08–6.99 (m, 1H), 6.89 (td, *J* = 7.3, 1.4 Hz, 1H), 4.94–4.88 (m, 1H), 4.56–4.47 (m, 1H), 3.86 (s, 3H), 3.78–3.67 (m, 2H), 3.50–3.40 (m, 2H), 2.16 (s, 3H), 1.44 (s, 9H). ^{13}C{^{1}H} NMR (101 MHz, DMSO-d_6, 343 K): δ 165.4, 156.7, 154.7, 153.6, 131.8, 130.4, 129.7, 126.6, 126.5, 123.5, 120.8, 120.2, 113.2, 112.0, 78.7, 78.5, 55.8, 53.2, 49.3, 48.9, 27.9, 15.4. HRMS (TOF, ES+) $C_{24}H_{31}N_2O_5$ $[M + H]^+$ calcd mass 427.2227, found 427.2227.

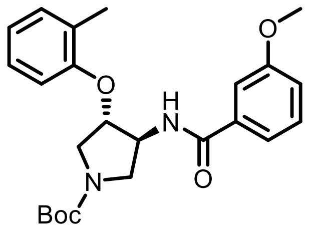

tert-Butyl (3*SR*,4*SR*)-3-(3-methoxybenzamido)-4-(o-tolyloxy)pyrrolidine-1-carboxylate (6b). Followed General Procedure C-(a) with *tert*-butyl 6-(3-methoxybenzoyl)-3,6-diazabicyclo[3.1.0]hexane-3-carboxylate (28.4 mg, 89.2 μmol) and *o*-cresol to give *tert*-butyl (3*SR*,4*SR*)-3-(3-methoxybenzamido)-4-(*o*-tolyloxy)pyrrolidine-1-carboxylate (34.0 mg, 89%) as a white solid after purification by column chromatography (0–100% EtOAc in hexane). ^{1}H NMR (400 MHz, DMSO-d_6, 343 K): δ 8.54 (d, *J* = 6.4 Hz, 1H), 7.49–7.44 (m, 1H), 7.44–7.41 (m, 1H), 7.38 (t, *J* = 7.9 Hz, 1H), 7.22–7.13 (m, 3H), 7.13–7.08 (m, 1H), 6.88 (td, *J* = 7.1, 1.7 Hz, 1H), 4.91–4.84 (m, 1H), 4.59–4.50 (m, 1H), 3.82 (s, 3H), 3.78–3.69 (m, 2H), 3.53–3.41 (m, 2H), 2.14 (s, 3H), 1.43 (s, 9H). ^{13}C{^{1}H} NMR (101 MHz, DMSO-d_6, 343 K): δ 166.4, 159.0, 154.7, 153.5, 135.3, 130.4, 129.0, 126.6, 126.5, 120.8, 119.5, 116.8, 113.3, 112.9, 78.8, 78.4, 55.1, 53.6, 49.3, 48.7, 27.9, 15.3. HRMS (TOF, ES+) $C_{24}H_{31}N_2O_5$ $[M + H]^+$ calcd mass 427.2227, found 427.2222.

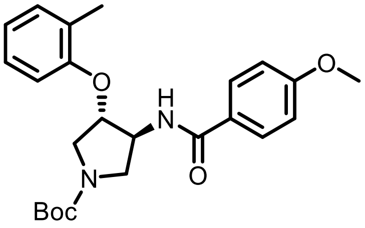

***tert*-Butyl (3*SR*,4*SR*)-3-(4-methoxybenzamido)-4-(*o*-tolyloxy)pyrrolidine-1-carboxylate (6c).** Followed General Procedure C-(a) with *tert*-butyl 6-(4-methoxybenzoyl)-3,6-diazabicyclo[3.1.0]hexane-3-carboxylate (28.4 mg, 89.2 μmol) and *o*-cresol to give *tert*-butyl (3*SR*,4*SR*)-3-(4-methoxybenzamido)-4-(*o*-tolyloxy)pyrrolidine-1-carboxylate (quantitative yield) as a white solid after purification by column chromatography (0–100% EtOAc in hexane). ^{1}H NMR (400 MHz, $CDCl_3$, 298 K): δ 7.79 (dd, *J* = 8.9, 2.5 Hz, 2H), 7.32–7.26 (m, 1H), 7.20 (t, *J* = 7.8 Hz, 1H), 7.16–7.10 (m, 1H), 6.95–6.85 (m, 3H), 6.58–6.36 (m, 1H), 4.93–4.88 (m, 1H), 4.69–4.62 (m, 1H), 3.84 (m, 4H), 3.74–3.59 (m, 2H), 3.54 (d, *J* = 11.9 Hz, 1H), 2.19 (s, 3H), 1.49 (s, 9H). ^{13}C{^{1}H} NMR (101 MHz, $CDCl_3$, 298 K): δ 167.4, 162.6, 155.1, 155.0, 131.0, 129.1, 127.3, 127.2, 126.0, 121.3, 113.9, 112.9, 80.3, 78.5, 55.5, 54.1, 50.1, 49.5, 28.6, 16.4. HRMS (TOF, ES+) $C_{24}H_{31}N_2O_5$ $[M + H]^+$ calcd mass 427.2227, found 427.2226.

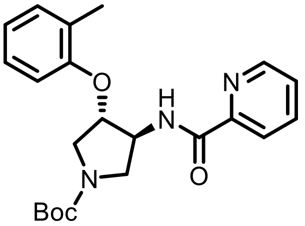

***tert*-Butyl (3*SR*,4*SR*)-3-(picolinamido)-4-(*o*-tolyloxy)pyrrolidine-1-carboxylate (6d).** Followed General Procedure C-(a) with *tert*-butyl 6-picolinoyl-3,6-diazabicyclo[3.1.0]hexane-3-carboxylate (25.8 mg, 89.2 μmol) and *o*-cresol to give *tert*-butyl (3*SR*,4*SR*)-3-(picolinamido)-4-(*o*-tolyloxy)pyrrolidine-1-carboxylate (28.2 mg, 80%) as a white solid after purification by column chromatography (0–100% EtOAc in hexane). ^{1}H NMR (400 MHz, DMSO-d_6, 343 K): δ 8.85 (d, *J* = 7.2 Hz, 1H), 8.65 (d, *J* = 4.7 Hz, 1H), 8.06 (d, *J* = 7.8 Hz, 1H), 7.99 (td, *J* = 7.7, 1.7 Hz, 1H), 7.63–7.57 (m, 1H), 7.18–7.10 (m, 3H), 6.91–6.83 (m, 1H), 5.04–4.96 (m, 1H), 4.64–4.54 (m, 1H), 3.84–3.71 (m, 2H), 3.52–3.40 (m, 2H), 2.14 (s, 3H), 1.43 (s, 9H). ^{13}C{^{1}H} NMR (101 MHz, DMSO-d_6, 343 K): δ 164.1, 154.8, 153.5, 149.4, 148.0, 137.4, 130.4, 126.6, 126.5, 126.3, 121.7, 120.8, 113.2, 78.7, 78.5, 53.3, 49.4, 48.7, 27.9, 15.4. HRMS (TOF, ES+) $C_{22}H_{28}N_3O_4$ $[M + H]^+$ calcd mass 398.2074, found 398.2073.

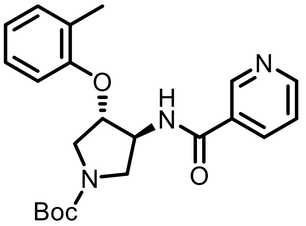

***tert*-Butyl (3*SR*,4*SR*)-3-(nicotinamido)-4-(*o*-tolyloxy)pyrrolidine-1-carboxylate (6e).** Followed General Procedure C-(a) with *tert*-butyl 6-nicotinoyl-3,6-diazabicyclo[3.1.0]hexane-3-carboxylate (25.8 mg, 89.2 μmol) and *o*-cresol to give *tert*-butyl (3*SR*,4*SR*)-3-(nicotinamido)-4-(*o*-tolyloxy)pyrrolidine-1-carboxylate (31.3 mg, 88%) as a white solid after purification by column chromatography (0–100% EtOAc in hexane). ^{1}H NMR (400 MHz, DMSO-d_6, 343 K): δ 9.10–8.97 (m, 1H), 8.79 (d, *J* = 6.4 Hz, 1H), 8.75–8.69 (m, 1H), 8.23 (dt, *J* = 8.0, 1.9 Hz, 1H), 7.55–7.47 (m, 1H), 7.23–7.12 (m, 3H), 6.88 (td, *J* = 7.1, 1.7 Hz, 1H), 4.92–4.85 (m, 1H), 4.60–4.52 (m, 1H), 3.80–3.70 (m, 2H), 3.53–3.42 (m, 2H), 2.14 (s, 3H), 1.43 (s, 9H). ^{13}C{^{1}H} NMR (101 MHz, DMSO-d_6, 343 K): δ 165.2, 154.7, 153.5, 151.4, 148.2, 135.2, 130.4, 129.6, 126.6, 126.5, 123.1, 120.9, 113.3, 78.8, 78.4, 53.6, 49.3, 48.6, 27.9, 15.4. HRMS (TOF, ES+) $C_{22}H_{28}N_3O_4$ $[M + H]^+$ calcd mass 398.2074, found 398.2079.

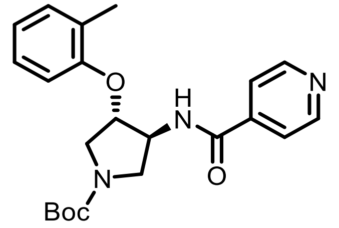

***tert*-Butyl (3*SR*,4*SR*)-3-(isonicotinamido)-4-(*o*-tolyloxy)pyrrolidine-1-carboxylate (6f).** Followed General Procedure C-(a) with *tert*-butyl 6-isonicotinoyl-3,6-diazabicyclo[3.1.0]hexane-3-carboxylate (25.8 mg, 89.2 μmol) and *o*-cresol to give *tert*-butyl (3*SR*,4*SR*)-3-(isonicotinamido)-4-(*o*-tolyloxy)pyrrolidine-1-carboxylate (22.2 mg, 63%) as a white solid after purification by column chromatography (0–100% EtOAc in hexane). ^{1}H NMR (400 MHz, DMSO-d_6, 343 K): δ 9.04 (d, *J* = 6.5 Hz, 1H), 8.91 (d, *J* = 6.1 Hz, 2H), 7.99–7.93 (m, 2H), 7.33 (m, 3H), 7.10–6.99 (m, 1H), 5.09–5.02 (m, 1H), 4.77–4.69 (m, 1H), 3.95–3.86 (m, 2H), 3.70–3.59 (m, 2H), 2.31 (s, 3H), 1.60 (s, 9H). ^{13}C{^{1}H} NMR (101 MHz, DMSO-d_6, 343 K): δ 165.1, 154.6, 153.5, 149.5, 141.1, 130.4, 126.6, 126.5, 121.3, 120.9, 113.3, 78.8, 78.4, 53.7, 49.3, 48.5, 27.9, 15.3. HRMS (TOF, ES+) $C_{22}H_{28}N_3O_4$ $[M + H]^+$ calcd mass 398.2074, found 398.2074.

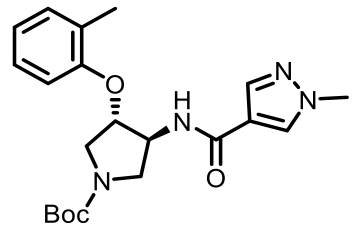

***tert*-Butyl (3*SR*,4*SR*)-3-(1-methyl-1*H*-pyrazole-4-carboxamido)-4-(*o*-tolyloxy)pyrrolidine-1-carboxylate (6g).** Followed General Procedure C-(a) with *tert*-butyl 6-(1-methyl-1*H*-pyrazole-4-carbonyl)-3,6-diazabicyclo[3.1.0]hexane-3-carboxylate (26.1 mg, 89.2 μmol) and *o*-cresol to give *tert*-butyl (3*SR*,4*SR*)-3-(1-methyl-1*H*-pyrazole-4-carboxamido)-4-(*o*-tolyloxy)pyrrolidine-1-

carboxylate (30.7 mg, 86%) as a white solid after purification by column chromatography (0–100% EtOAc in hexane), ^{1}H NMR (400 MHz, $CDCl_3$, 298 K): δ 7.99–7.87 (m, 2H), 7.40–7.08 (m, 3H), 6.88 (t, J = 7.9 Hz, 2H), 4.97–4.77 (m, 1H), 4.71–4.49 (m, 1H), 3.89 (s, 3H), 3.79 (dd, J = 11.8, 5.3 Hz, 1H), 3.72–3.38 (m, 3H), 2.18 (s, 3H), 1.48 (s, 9H). ^{13}C{^{1}H} NMR (101 MHz, $CDCl_3$, 298 K): δ 163.0, 155.1, 155.0, 138.9, 132.2, 131.0, 127.2, 127.2, 121.3, 118.3, 112.9, 80.4, 78.6, 53.6, 50.3, 49.1, 39.4, 28.7, 16.4. HRMS (TOF, ES+) $C_{21}H_{29}N_4O_4$ $[M + H]^+$ calcd mass 401.2183, found 401.2183.

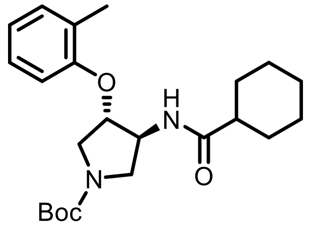

***tert*-Butyl (3*SR*,4*SR*)-3-(cyclohexanecarboxamido)-4-(*o*-tolyloxy)pyrrolidine-1-carboxylate (6h).** Followed General Procedure C-(a) with *tert*-butyl 6-(cyclohexanecarbonyl)-3,6-diazabicyclo[3.1.0]hexane-3-carboxylate (26.3 mg, 89.2 μmol) and *o*-cresol to give *tert*-butyl (3*SR*,4*SR*)-3-(cyclohexanecarboxamido)-4-(*o*-tolyloxy)pyrrolidine-1-carboxylate (27.6 mg, 77%) as a white solid after purification by column chromatography (0–100% EtOAc in hexane). ^{1}H NMR (400 MHz, $CDCl_3$, 298 K): δ 7.08–6.99 (m, 1H), 6.97–6.89 (m, 1H), 6.88–6.82 (m, 1H), 6.65–6.57 (m, 1H), 5.86–5.52 (m, 1H), 4.57–4.41 (m, 1H), 4.26–4.11 (m, 1H), 3.47 (dd, J = 11.8, 5.2 Hz, 1H), 3.39–3.27 (m, 2H), 3.21–3.03 (m, 1H), 1.91 (s, 3H), 1.89–1.82 (m, 1H), 1.61–1.50 (m, 4H), 1.45–1.32 (m, 1H), 1.23 (s, 9H), 1.28–1.13 (m, 2H), 1.08–0.91 (m, 3H). ^{13}C{^{1}H} NMR (101 MHz, $CDCl_3$, 298 K): δ 176.8, 155.1, 154.9, 130.9, 127.2, 127.2, 121.2, 112.8, 80.2, 78.4, 53.3, 50.1, 49.3, 45.3, 29.9, 29.6, 28.6, 25.8, 25.7, 25.7, 16.4. HRMS (TOF, ES+) $C_{23}H_{35}N_2O_4$ $[M + H]^+$ calcd mass 403.2591, found 403.2591.

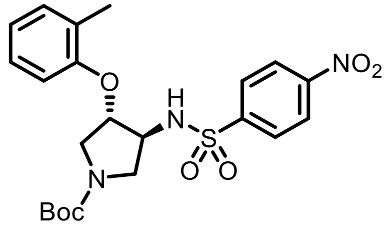

***tert*-Butyl (3*SR*,4*SR*)-3-((4-nitrophenyl)sulfonamido)-4-(*o*-tolyloxy)pyrrolidine-1-carboxylate (6i).** Followed General Procedure C-(a) with *tert*-butyl 6-((4-nitrophenyl)sulfonyl)-3,6-diazabicyclo[3.1.0]hexane-3-carboxylate (33.0 mg, 89.2 μmol) and *o*-cresol to give *tert*-butyl (3*SR*,4*SR*)-3-((4-nitrophenyl)sulfonamido)-4-(*o*-tolyloxy)pyrrolidine-1-carboxylate (28.0 mg, 66%) as a white solid after purification by column chromatography (0–100% EtOAc in hexane). ^{1}H NMR (400 MHz, DMSO-d_6, 343 K): δ 8.56 (d, J = 5.8 Hz, 1H), 8.39–8.31 (m, 2H), 8.11–8.03 (m, 2H), 7.07 (m, 2H), 6.88–6.76 (m, 2H), 4.74–4.66 (m, 1H), 3.85–3.78 (m, 1H), 3.74–3.66 (m, 1H), 3.58–3.49 (m, 1H), 3.31 (dd, J = 12.1, 2.4 Hz, 1H), 3.21 (dd, J = 12.1, 3.3 Hz, 1H), 2.02 (s, 3H), 1.39 (s, 9H). ^{13}C{^{1}H} NMR (101 MHz, DMSO-d_6, 343 K): δ 154.2, 153.2, 149.4,

146.1, 130.5, 127.8, 126.5, 126.5, 124.2, 121.0, 112.8, 78.9, 78.6, 55.9, 49.0, 48.8, 27.8, 15.2. HRMS (TOF, ES+) $C_{22}H_{27}N_3NaO_7S$ $[M + Na]^+$ calcd mass 500.1462, found 500.1459.

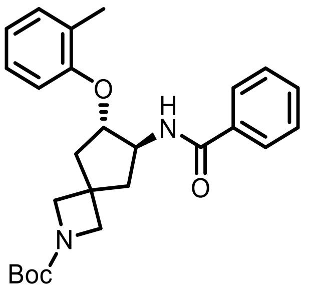

***tert*-Butyl (6*SR*,7*SR*)-6-benzamido-7-(*o*-tolyloxy)-2-azaspiro[3.4]octane-2-carboxylate (6j).** Followed General Procedure C-(a) with *tert*-butyl 6'-benzoyl-6'-azaspiro[azetidine-3,3'-bicyclo[3.1.0]hexane]-1-carboxylate (29.3 mg, 89.2 μmol) and *o*-cresol to give *tert*-butyl (6*SR*,7*SR*)-6-benzamido-7-(*o*-tolyloxy)-2-azaspiro[3.4]octane-2-carboxylate (34.3 mg, 88%) as a white solid after purification by column chromatography (0–100% EtOAc in hexane). ^{1}H NMR (400 MHz, MeOD, 298 K): δ 7.83–7.75 (m, 2H), 7.56–7.48 (m, 1H), 7.47–7.39 (m, 2H), 7.14–7.06 (m, 2H), 7.01 (d, *J* = 8.8 Hz, 1H), 6.84–6.77 (m, 1H), 4.83–4.76 (m, 1H), 4.55–4.46 (m, 1H), 3.98–3.84 (m, 4H), 2.60–2.49 (m, 1H), 2.42–2.30 (m, 1H), 2.23–2.18 (m, 1H), 2.16 (s, 3H), 2.03–1.93 (m, 1H), 1.44 (s, 9H). ^{13}C{^{1}H} NMR (101 MHz, MeOD, 298 K): δ 170.1, 158.1, 157.0, 135.6, 132.7, 131.8, 129.5, 128.4, 128.0, 127.9, 121.7, 113.6, 83.6, 80.9, 64.3, 60.5, 57.7, 43.5, 43.2, 40.3, 28.7, 16.7. HRMS (TOF, ES+) $C_{26}H_{33}N_2O_4$ $[M + H]^+$ calcd mass 437.2435, found 437.2439.

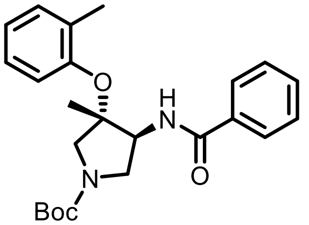

***tert*-Butyl (3*SR*,4*SR*)-4-benzamido-3-methyl-3-(*o*-tolyloxy)pyrrolidine-1-carboxylate (6ka).** Followed General Procedure C-(a) with *tert*-butyl 6-benzoyl-1-methyl-3,6-diazabicyclo[3.1.0]hexane-3-carboxylate (27.0 mg, 89.2 μmol), Cs_2CO_3 (146 mg, 446 μmol) and *o*-cresol (48.2 mg, 446 μmol) at 140 °C for 40 min to give *tert*-butyl (3*SR*,4*SR*)-4-benzamido-3-methyl-3-(*o*-tolyloxy)pyrrolidine-1-carboxylate as the main product [26.6 mg, 73%, Regio isomer ratio = 13 : 1 (determined by LCMS)]. Purification was conducted by RP-HPLC (15–75% MeCN in 0.05% aqueous NH_4OH). Isolated as a white solid. ^{1}H NMR (400 MHz, DMSO-*d*$_6$, 343 K): δ 8.50 (d, *J* = 8.7 Hz, 1H), 7.92–7.84 (m, 2H), 7.58–7.51 (m, 1H), 7.51–7.44 (m, 2H), 7.21–7.09 (m, 3H), 6.93 (ddd, *J* = 7.2, 7.1, 1.7 Hz, 1H), 4.99 (d, *J* = 7.8 Hz, 1H), 3.81 (dd, *J* = 11.3, 7.2 Hz, 1H), 3.75 (d, *J* = 12.2 Hz, 1H), 3.46 (d, *J* = 12.2 Hz, 1H), 3.31 (dd, *J* = 11.3, 2.8 Hz, 1H), 2.14 (s, 3H), 1.48–1.33 (m, 12H). ^{13}C{^{1}H} NMR (101 MHz, DMSO-*d*$_6$, 343 K): δ 166.7, 153.3, 152.6, 134.2, 130.9, 130.7, 129.6, 127.8, 127.2, 126.1, 121.9, 118.9, 78.3, 54.9,

54.3, 49.7, 27.9, 16.8, 15.8. HRMS (TOF, ES+) $C_{24}H_{31}N_2O_4$ $[M + H]^+$ calcd mass 411.2278, found 411.2275.

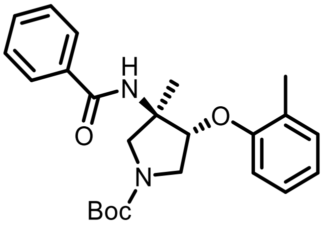

***tert*-Butyl (3*RS*,4*SR*)-3-benzamido-3-methyl-4-(*o*-tolyloxy)pyrrolidine-1-carboxylate (6kb).** Isolated as a white solid. **6kb** was fully characterized in NH-free form **6kb'**. HRMS (TOF, ES+) $C_{24}H_{31}N_2O_4$ $[M + H]^+$ calcd mass 411.2278, found 411.2274.

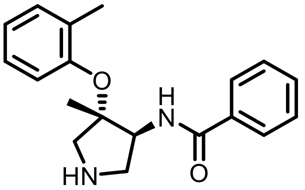

***N*-((3*SR*,4*SR*)-4-methyl-4-(*o*-tolyloxy)pyrrolidin-3-yl)benzamide (6ka').** To a solution of *tert*-butyl (3*SR*,4*SR*)-4-benzamido-3-methyl-3-(*o*-tolyloxy)pyrrolidine-1-carboxylate (47.9 mg, 117 µmol) in DCM (1.0 mL), TFA (1.0 mL) was added at rt. The mixture was stirred at rt for 10 min and concentrated. To this mixture, sat. aq. $NaHCO_3$ (1.0 mL) was added, and the mixture was extracted with DCM (3x1.0 mL) and concentrated. The crude material was purified by RP-HPLC (10–50% MeCN in 0.05% aqueous NH_4OH) and concentrated to give *N*-((3*SR*,4*SR*)-4-methyl-4-(*o*-tolyloxy)pyrrolidin-3-yl)benzamide (33.0 mg, 91%) as a white solid. ^1H NMR (400 MHz, DMSO-d_6, 298 K): δ 8.42 (d, *J* = 9.1 Hz, 1H), 7.92–7.88 (m, 2H), 7.57–7.51 (m, 1H), 7.51–7.44 (m, 2H), 7.27–7.21 (m, 1H), 7.17–7.12 (m, 1H), 7.11–7.05 (m, 1H), 6.85 (td, *J* = 7.4, 1.1 Hz, 1H), 4.94–4.84 (m, 1H), 3.35 (dd, *J* = 11.9, 7.3 Hz, 1H), 3.24 (d, *J* = 12.3 Hz, 1H), 2.92 (d, *J* = 12.2 Hz, 1H), 2.79 (dd, *J* = 11.9, 4.7 Hz, 1H), 2.17–2.09 (m, 3H), 1.40–1.31 (m, 3H). $^{13}C\{^1H\}$ NMR (101 MHz, DMSO-d_6, 298 K): δ 166.5, 153.5, 134.6, 131.3, 130.7, 129.2, 128.3, 127.5, 126.3, 121.1, 117.8, 89.2, 58.6, 57.4, 51.8, 17.4, 16.7. HRMS (TOF, ES+) $C_{19}H_{23}N_2O_2$ $[M + H]^+$ calcd mass 311.1754, found 311.1755.

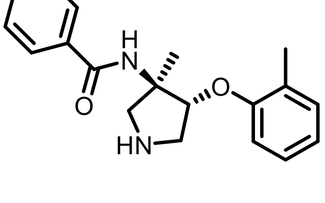

***N*-((3*RS*,4*SR*)-3-Methyl-4-(*o*-tolyloxy)pyrrolidin-3-yl)benzamide (6kb').** To a solution of *tert*-butyl (3*RS*,4*SR*)-3-benzamido-3-methyl-4-(*o*-tolyloxy)pyrrolidine-1-carboxylate (10.9 mg, 26.6 µmol) in DCM (0.5 mL), TFA (0.5 mL) was added at rt. The mixture was stirred at rt for 10 min and concentrated. To this mixture, sat. aq. $NaHCO_3$ (1.0 mL) was added, and the mixture was extracted with DCM (3x1.0 mL) and concentrated. The crude material was purified by RP-HPLC

(20–60% MeCN in 0.05% aqueous NH_4OH) and concentrated to give *N*-((3*RS*,4*SR*)-3-methyl-4-(*o*-tolyloxy)pyrrolidin-3-yl)benzamide (6.1 mg, 74%) as a white solid. ^{1}H NMR (400 MHz, $CDCl_3$, 298 K): δ 7.78–7.73 (m, 2H), 7.54–7.48 (m, 1H), 7.47–7.40 (m, 2H), 7.16–7.09 (m, 2H), 6.91 (d, *J* = 8.0 Hz, 1H), 6.88–6.82 (m, 1H), 6.31 (s, 1H), 5.25 (dd, *J* = 5.5, 4.0 Hz, 1H), 3.56 (dd, *J* = 12.4, 5.5 Hz, 1H), 3.36 (d, *J* = 11.5 Hz, 1H), 3.21 (d, *J* = 11.5 Hz, 1H), 3.02 (dd, *J* = 12.4, 4.0 Hz, 1H), 2.24 (s, 3H), 2.20–2.06 (m, 1H), 1.63 (s, 3H). ^{13}C{^{1}H} NMR (101 MHz, $CDCl_3$, 298 K): δ 167.6, 156.0, 135.0, 131.8, 131.0, 128.8, 127.1, 127.0, 120.7, 112.2, 80.9, 64.2, 58.5, 52.4, 19.2, 16.7. HRMS (TOF, ES+) $C_{19}H_{23}N_2O_2$ [M + H]$^+$ calcd mass 311.1754, found 311.1753.

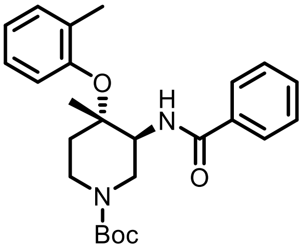

***tert*-Butyl (3*SR*,4*SR*)-3-benzamido-4-methyl-4-(*o*-tolyloxy)piperidine-1-carboxylate (6l).** Followed General Procedure C-(a) with *tert*-butyl 7-benzoyl-6-methyl-3,7-diazabicyclo[4.1.0]heptane-3-carboxylate (28.2 mg, 89.2 µmol), Cs_2CO_3 (146 mg, 446 µmol) and *o*-cresol (48.2 mg, 446 µmol) at 140 °C for 80 min to give *tert*-butyl (3*SR*,4*SR*)-3-benzamido-4-methyl-4-(*o*-tolyloxy)piperidine-1-carboxylate (18.1 mg, 48%) as a white solid after purification by RP-HPLC (37–77% MeCN in 0.05% aqueous NH_4OH). ^{1}H NMR (400 MHz, $CDCl_3$, 298 K): δ 7.80–7.73 (m, 2H), 7.57–7.49 (m, 1H), 7.45 (ddt, *J* = 8.3, 6.5, 1.3 Hz, 2H), 7.20–7.16 (m, 1H), 7.13 (td, *J* = 7.7, 1.8 Hz, 1H), 7.06 (dd, *J* = 8.2, 1.3 Hz, 1H), 6.95 (td, *J* = 7.3, 1.3 Hz, 1H), 6.45 (d, *J* = 8.7 Hz, 1H), 4.73 (brd, *J* = 8.9, 1H), 4.09–3.92 (m, 2H), 3.67 (dd, *J* = 13.5, 2.5 Hz, 1H), 3.39–3.30 (m, 1H), 2.28 (s, 3H), 2.07 (brd, *J* = 14.5 Hz, 1H), 1.75 (ddd, *J* = 14.5, 12.4, 4.9 Hz, 1H), 1.46 (s, 9H), 1.30 (s, 3H). ^{13}C{^{1}H} NMR (101 MHz, $CDCl_3$, 298 K): δ 167.4, 155.8, 153.6, 134.6, 131.9, 131.4, 131.1, 128.8, 127.0, 126.7, 122.7, 120.2, 80.5, 79.4, 51.6, 45.1, 40.1, 34.4, 28.5, 22.1, 17.5. HRMS (TOF, ES+) $C_{25}H_{33}N_2O_4$ [M + H]$^+$ calcd mass 425.2435, found 425.2434.

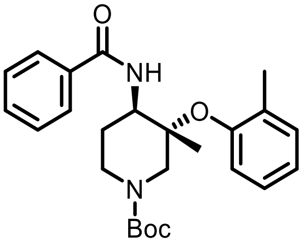

***tert*-Butyl (3*RS*,4*RS*)-4-benzamido-3-methyl-3-(*o*-tolyloxy)piperidine-1-carboxylate (6ma).** Followed General Procedure C-(a) with *tert*-butyl 7-benzoyl-1-methyl-3,7-diazabicyclo[4.1.0]heptane-3-carboxylate (28.2 mg, 89.2 µmol), Cs_2CO_3 (146 mg, 446 µmol) and *o*-cresol (48.2 mg, 446 µmol) at 140 °C for 80 min to give *tert*-butyl (3*RS*,4*RS*)-4-benzamido-3-methyl-3-(*o*-tolyloxy)piperidine-1-carboxylate as the main product (22.7 mg, 60%, Regio isomer

Ratio = 4.4 : 1) after purification by RP-HPLC (32–72% MeCN in 0.05% aqueous NH_4OH). Isolated as a white solid. ^1H NMR (400 MHz, DMSO-d_6, 343 K): δ 8.02 (d, J = 9.4 Hz, 1H), 7.90–7.82 (m, 2H), 7.56–7.50 (m, 1H), 7.47 (dd, J = 8.2, 6.5 Hz, 2H), 7.15 (d, J = 7.4 Hz, 1H), 7.12–7.07 (m, 2H), 6.97–6.89 (m, 1H), 4.67 (td, J = 8.7, 4.4 Hz, 1H), 3.84 (d, J = 13.5 Hz, 1H), 3.74–3.60 (m, 1H), 3.50 (d, J = 13.5 Hz, 1H), 3.37 (ddd, J = 13.0, 8.2, 4.1 Hz, 1H), 2.14 (s, 3H), 2.00–1.88 (m, 1H), 1.70–1.58 (m, 1H), 1.40 (s, 9H), 1.21 (s, 3H). $^{13}C\{^1H\}$ NMR (101 MHz, DMSO-d_6, 343 K): δ 166.5, 153.7, 152.9, 134.8, 130.7, 130.6, 127.8, 127.2, 126.0, 122.3, 120.8, 79.5, 78.5, 51.6, 50.4, 40.0, 27.7, 27.6, 17.2, 16.3. HRMS (TOF, ES+) $C_{25}H_{33}N_2O_4$ $[M + H]^+$ calcd mass 425.2440, found 425.2435.

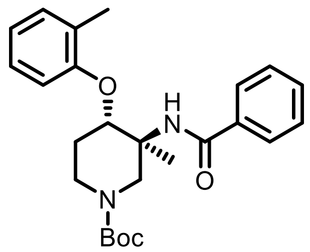

***tert*-Butyl (3*SR*,4*SR*)-3-benzamido-3-methyl-4-(*o*-tolyloxy)piperidine-1-carboxylate (6mb).** Isolated as a white solid. ^1H NMR (400 MHz, DMSO-d_6, 343 K): δ 7.73–7.64 (m, 2H), 7.55–7.46 (m, 1H), 7.46–7.37 (m, 3H), 7.15–7.08 (m, 2H), 6.97 (d, J = 7.6 Hz, 1H), 6.82 (td, J = 7.4, 1.2 Hz, 1H), 5.29 (m, 1H), 4.01 (d, J = 12.8 Hz, 1H), 3.68 (d, J = 13.4 Hz, 1H), 3.57 (ddd, J = 12.1, 7.6, 4.3 Hz, 1H), 3.36 (ddd, J = 13.4, 8.2, 4.8 Hz, 1H), 2.21 (s, 3H), 2.03–1.91 (m, 1H), 1.72–1.59 (m, 1H), 1.47 (s, 3H), 1.40 (s, 9H). $^{13}C\{^1H\}$ NMR (101 MHz, DMSO-d_6, 343 K): δ 166.6, 155.3, 154.2, 135.2, 130.7, 130.4, 127.8, 126.8, 126.54, 126.52, 120.2, 112.8, 78.8, 74.0, 56.3, 48.5, 39.7, 27.7, 25.7, 17.7, 15.7. HRMS (TOF, ES+) $C_{25}H_{33}N_2O_4$ $[M + H]^+$ calcd mass 425.2435, found 425.2433.

6. Synthetic procedures and characterization data for reference compounds

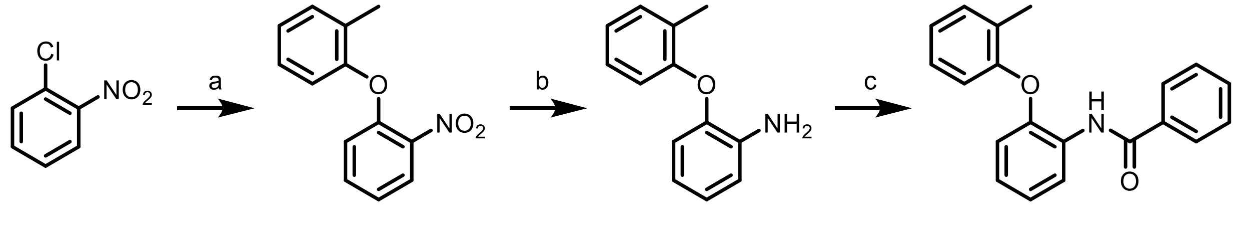

d1 **d2** **d**

Scheme S4. Synthesis of reference compounds a) Ar-Cl (1.0 equiv), Cs_2CO_3 (1.1 equiv), *o*-cresol (1.1 equiv), THF, 140 °C, microwave irradiation. b) PtO, H_2 (1 atm), MeOH, rt. c) BzCl (2.0 equiv), NEt_3 (4.0 equiv), DCM, 0 °C, 62% (3 steps).

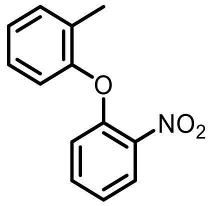

1-Methyl-2-(2-nitrophenoxy)benzene (d1). To a solution of 1-chloro-2-nitrobenzene (200 mg, 1.27 mmol) in THF (6.35 mL, 0.20 M) was added Cs_2CO_3 (458 mg, 1.40 mmol) followed by *o*-cresol (151 mg, 1.40 mmol). The resulting mixture was stirred at 140 °C for 2 h under microwave irradiation. After cooling to rt, sat. aq. $NaHCO_3$ (5.0 mL) was added, and the mixture was extracted with DCM (3 × 5.0 mL) and concentrated to give 1-methyl-2-(2-nitrophenoxy)benzene as a pale yellow oil. This material was used for the next reaction without any further purification. HRMS (TOF, ES+) $C_{13}H_{12}NO_3$ $[M + H]^+$ calcd mass 230.0812, found 230.0811.

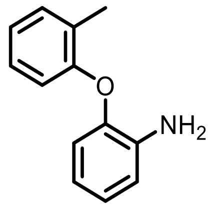

2-(*o*-Tolyloxy)aniline (d2). To a flask containing PtO (53.6 mg, 254 μmol) was added MeOH (6.35 mL) followed by 1-methyl-2-(2-nitrophenoxy)benzene. A hydrogen balloon was added and the flask was vacuum purged. The mixture was allowed to stir for 2.5 h at rt. The mixture was filtered through a Celite pad and concentrated to afford 2-(*o*-tolyloxy)aniline as a pale brown oil. This material was used for the next reaction without any further purification. HRMS (TOF, ES+) $C_{13}H_{14}NO$ $[M + H]^+$ calcd mass 200.1070, found 200.1072.

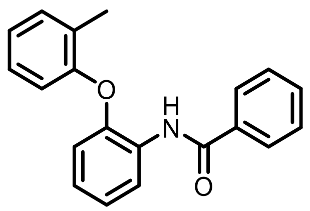

***N*-(2-(*o*-Tolyloxy)phenyl)benzamide (d).** To a solution of 2-(*o*-tolyloxy)aniline in DCM (6.35 mL) was added NEt_3 (708 µL, 5.08 mmol) followed by benzoylchloride (295 µL, 2.54 mmol) at 0 °C. The resulting mixture was stirred at 0 °C for 30 min. To this mixture, sat. aq. $NaHCO_3$ (5.0 mL) was added, and the mixture was extracted with DCM (3 × 5.0 mL) and concentrated. This crude material was purified by RP-HPLC (32–72% MeCN in 0.05% aqueous NH_4OH) and concentrated to give N-(2-(*o*-tolyloxy)phenyl)benzamide [237 mg, 782 µmol, 62% (3 steps)] as a colorless oil. ^1H NMR (400 MHz, $CDCl_3$, 298 K): δ 8.68–8.61 (m, 2H), 7.91–7.83 (m, 2H), 7.58–7.52 (m, 1H), 7.51–7.45 (m, 2H), 7.33–7.28 (m, 1H), 7.25–7.18 (m, 1H), 7.17–7.10 (m, 2H), 7.04–6.93 (m, 2H), 6.69 (dd, J = 8.2, 1.4 Hz, 1H), 2.28 (s, 3H). $^{13}C\{^1H\}$ NMR (101 MHz, $CDCl_3$, 298 K): δ 165.4, 153.7, 146.6, 135.2, 132.0, 131.8, 129.9, 129.0, 129.0, 127.6, 127.1, 124.9, 124.2, 123.4, 120.7, 119.8, 115.6, 16.3. HRMS (TOF, ES+) $C_{20}H_{18}NO_2$ $[M + H]^+$ calcd mass 304.1332, found 304.1339.

7. ^{1}H, ^{13}C{^{1}H} NMR , 2D NMR spectra and key relative stereochemistry determination

^{1}H NMR spectrum (400 MHz, DMSO-d_6, 343 K) of compound **S2b**

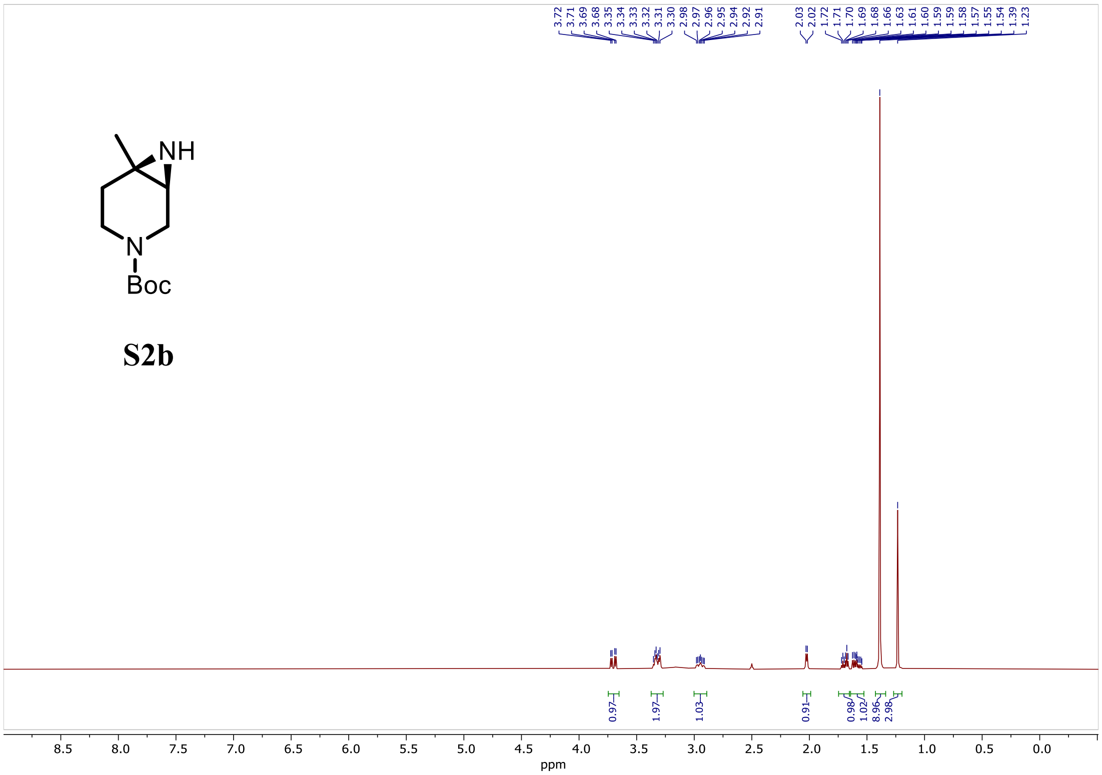

^{13}C{^{1}H} NMR spectrum (101 MHz, DMSO-d_6, 343 K) of compound **S2b**

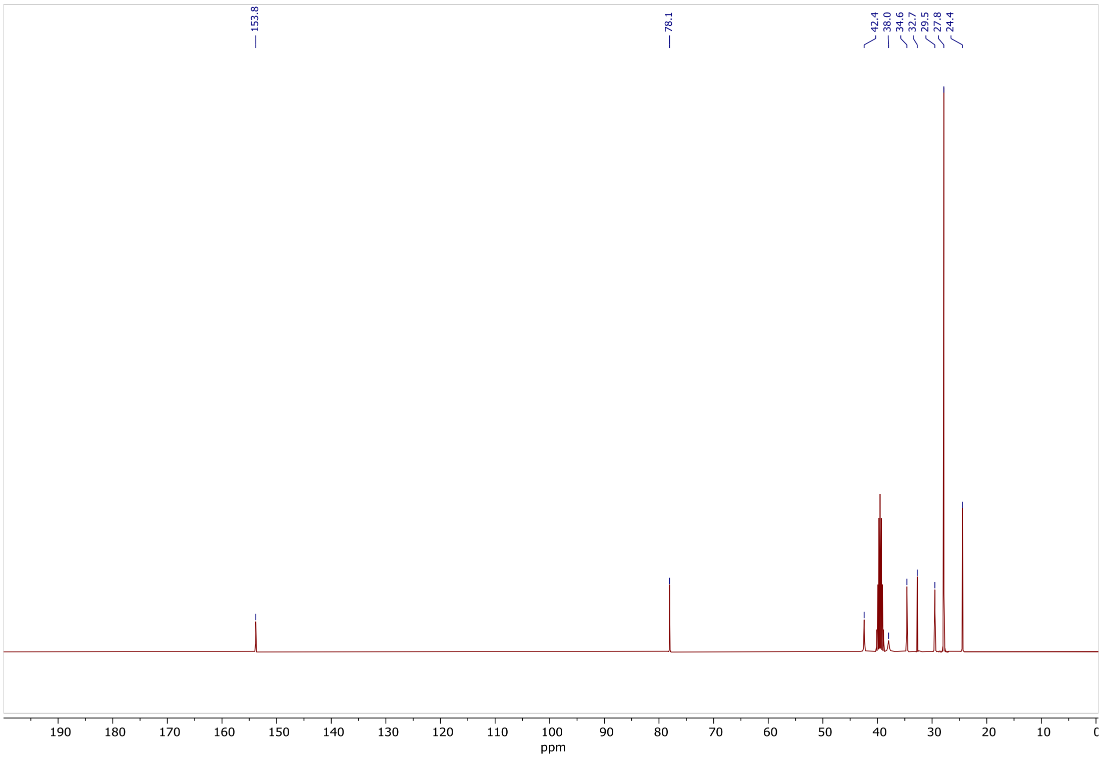

^{1}H NMR spectrum (400 MHz, DMSO-d_6, 298 K) of compound **1a**

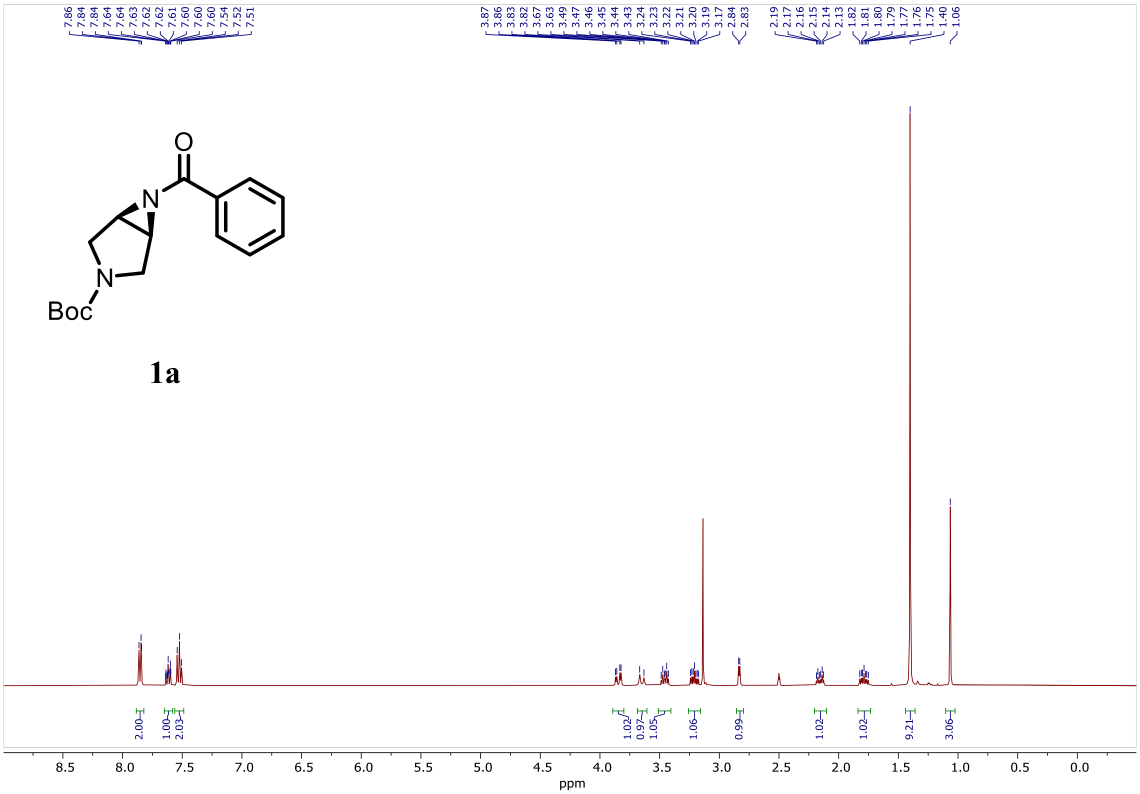

$^{13}C\{^{1}H\}$ NMR spectrum (101 MHz, DMSO-d_6, 298 K) of compound **1a**

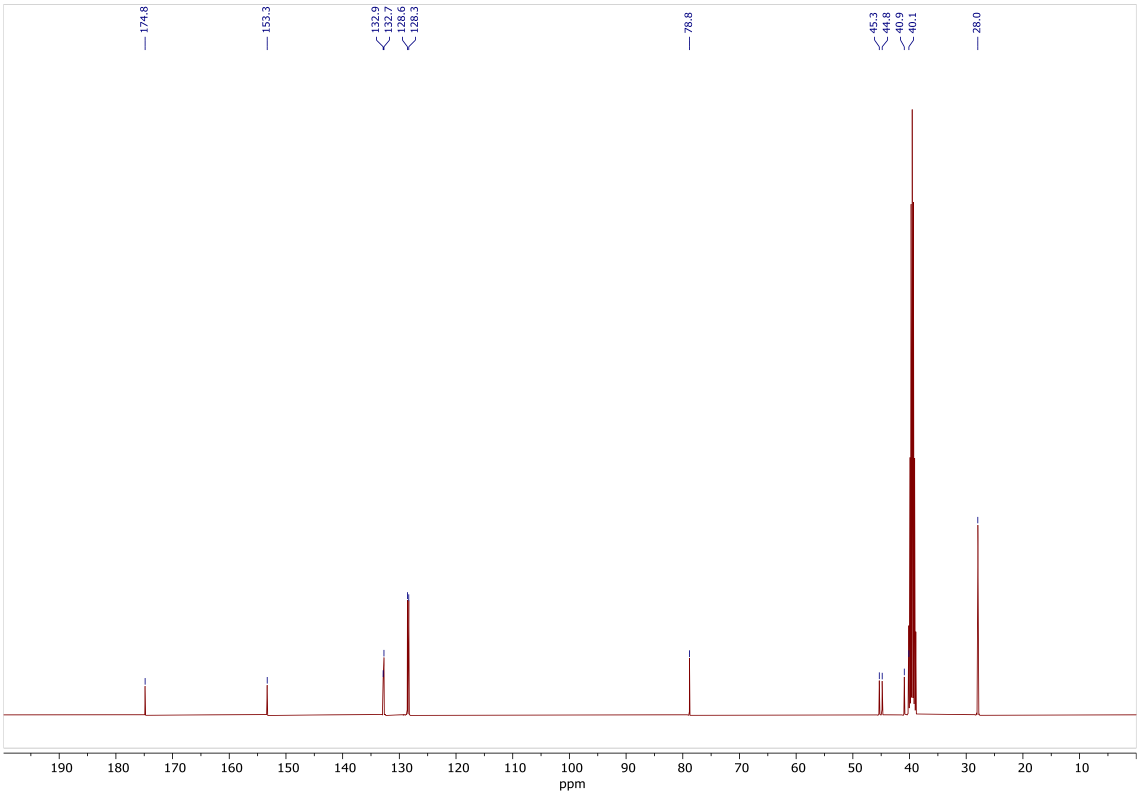

^{1}H NMR spectrum (400 MHz, DMSO-d_6, 343 K) of compound **5l**

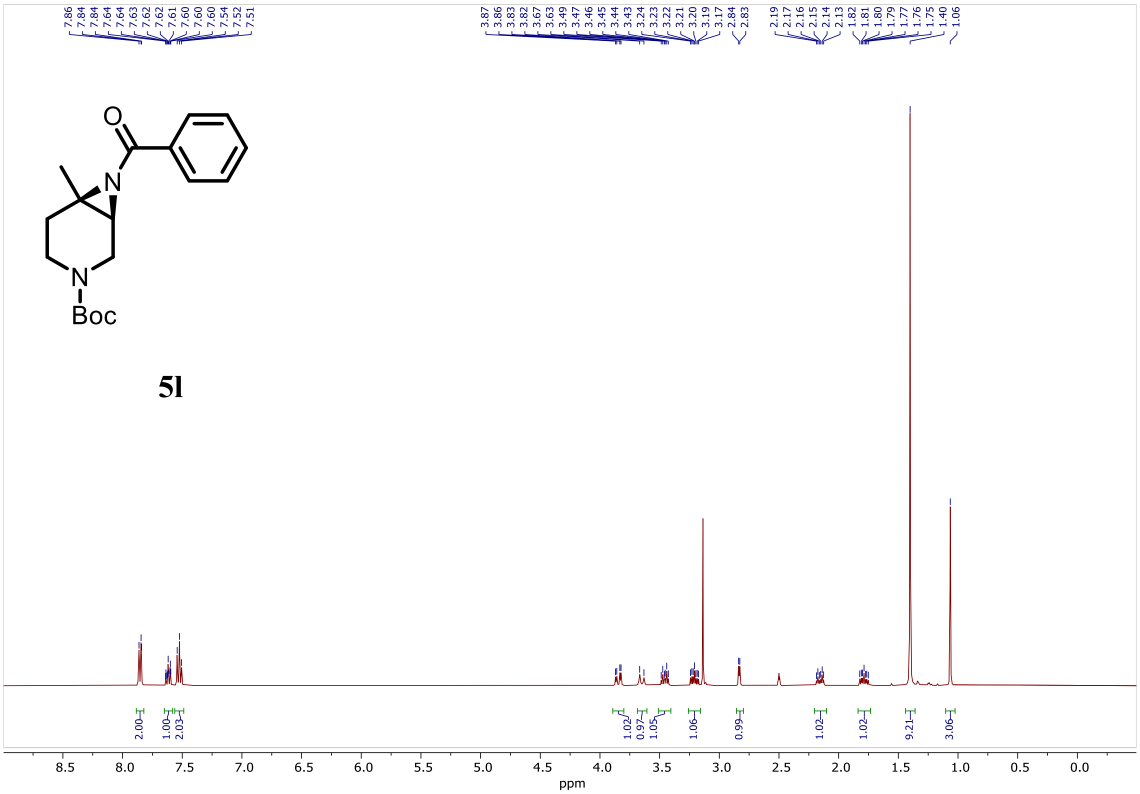

$^{13}C\{^{1}H\}$ NMR spectrum (101 MHz, DMSO-d_6, 343 K) of compound **51**

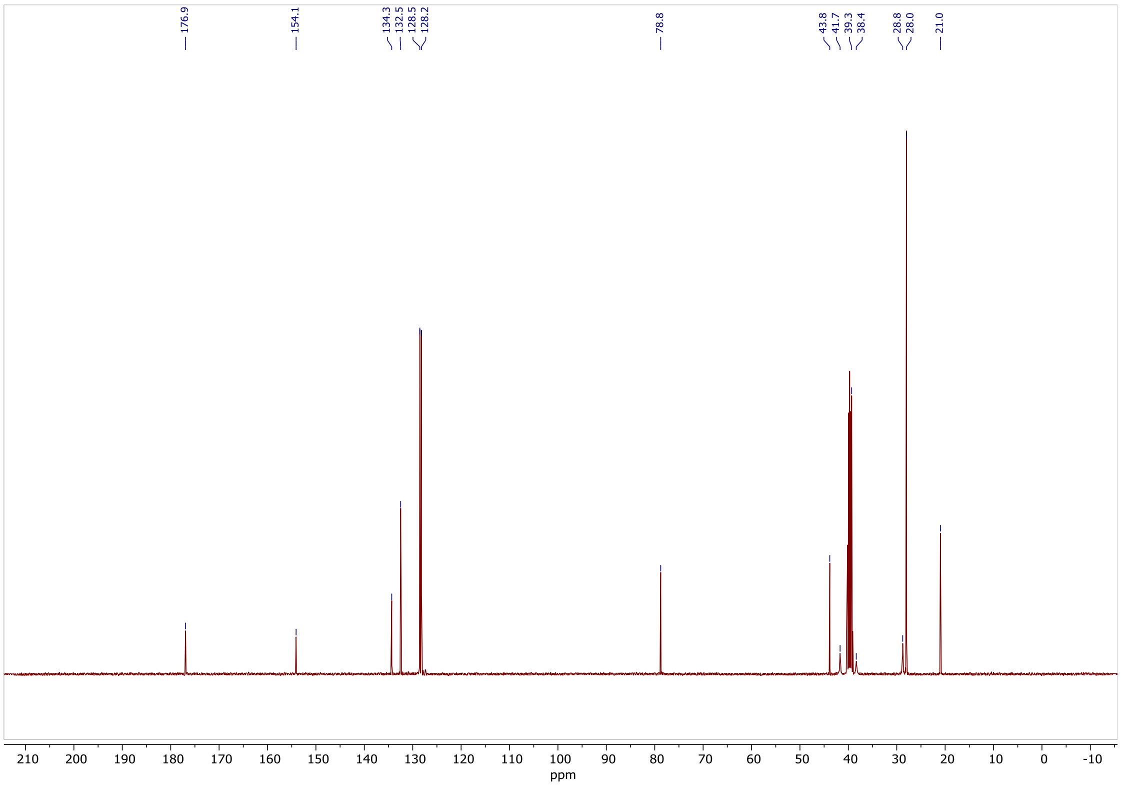

^{1}H NMR spectrum (400 MHz, DMSO-d_6, 343 K) of compound **3**

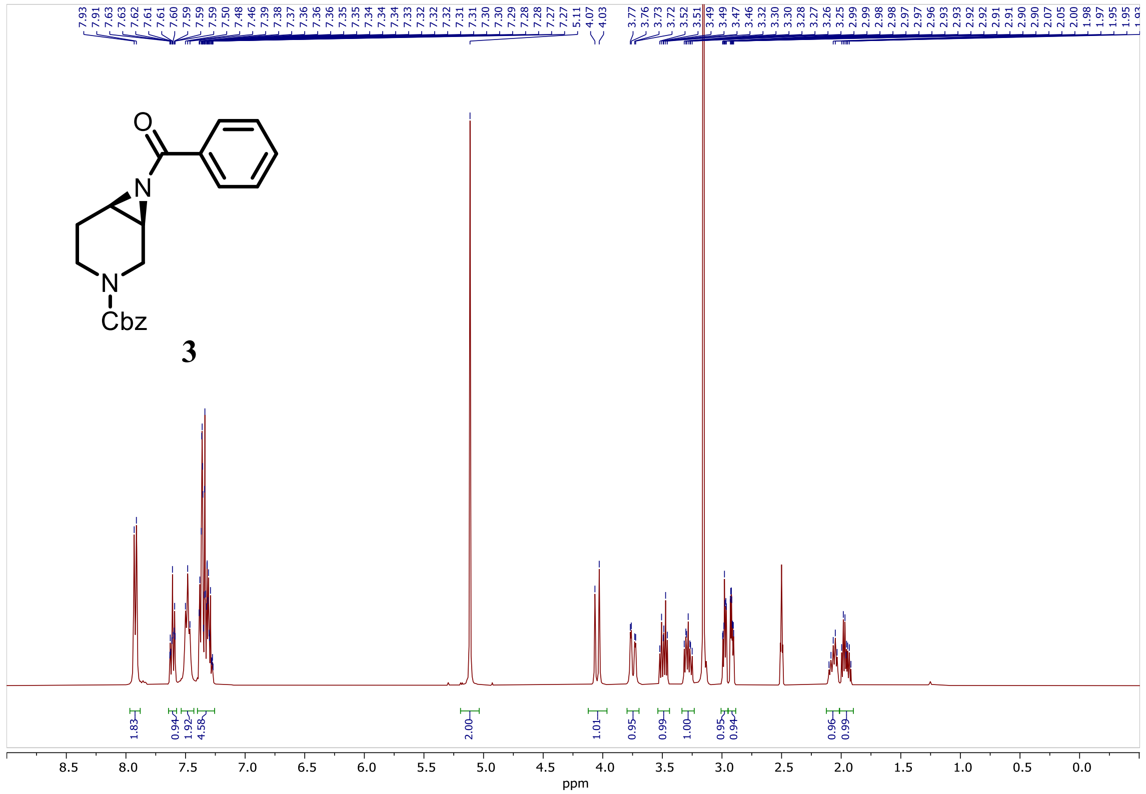

$^{13}C\{^{1}H\}$ NMR spectrum (101 MHz, DMSO-d_6, 343 K) of compound **3**

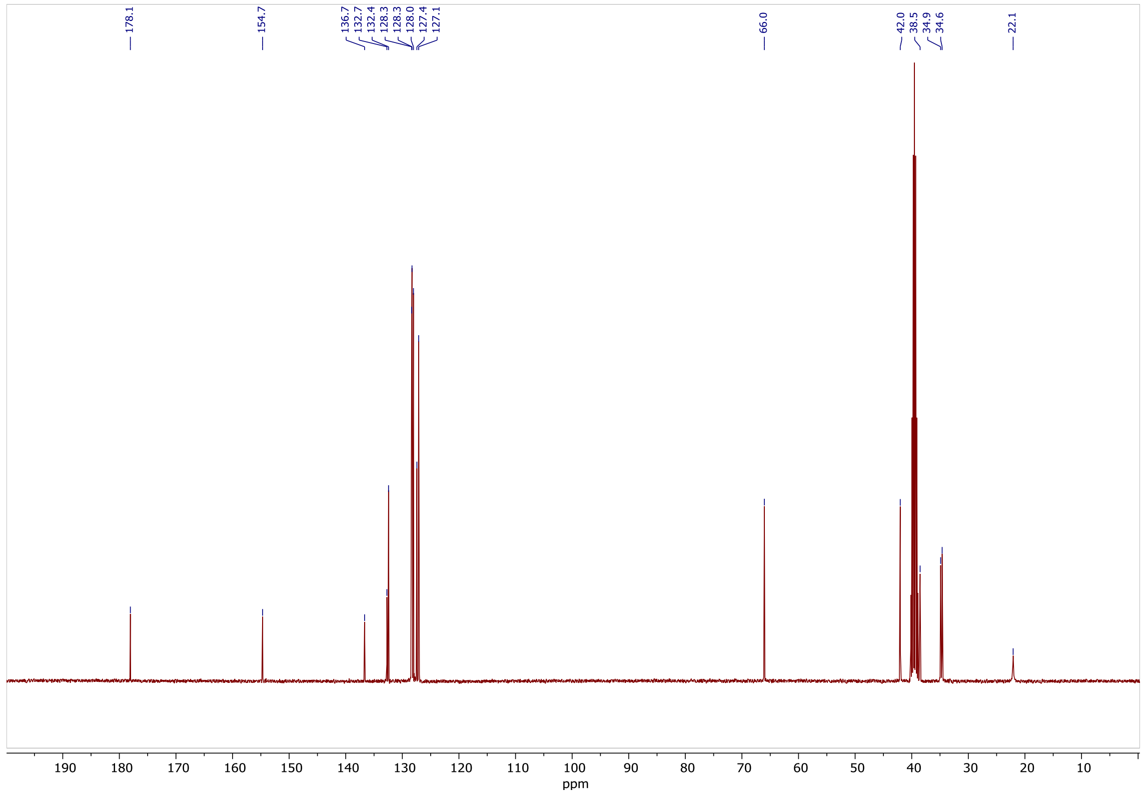

^{1}H NMR spectrum (400 MHz, DMSO-d_6, 298 K) of compound **S2a**

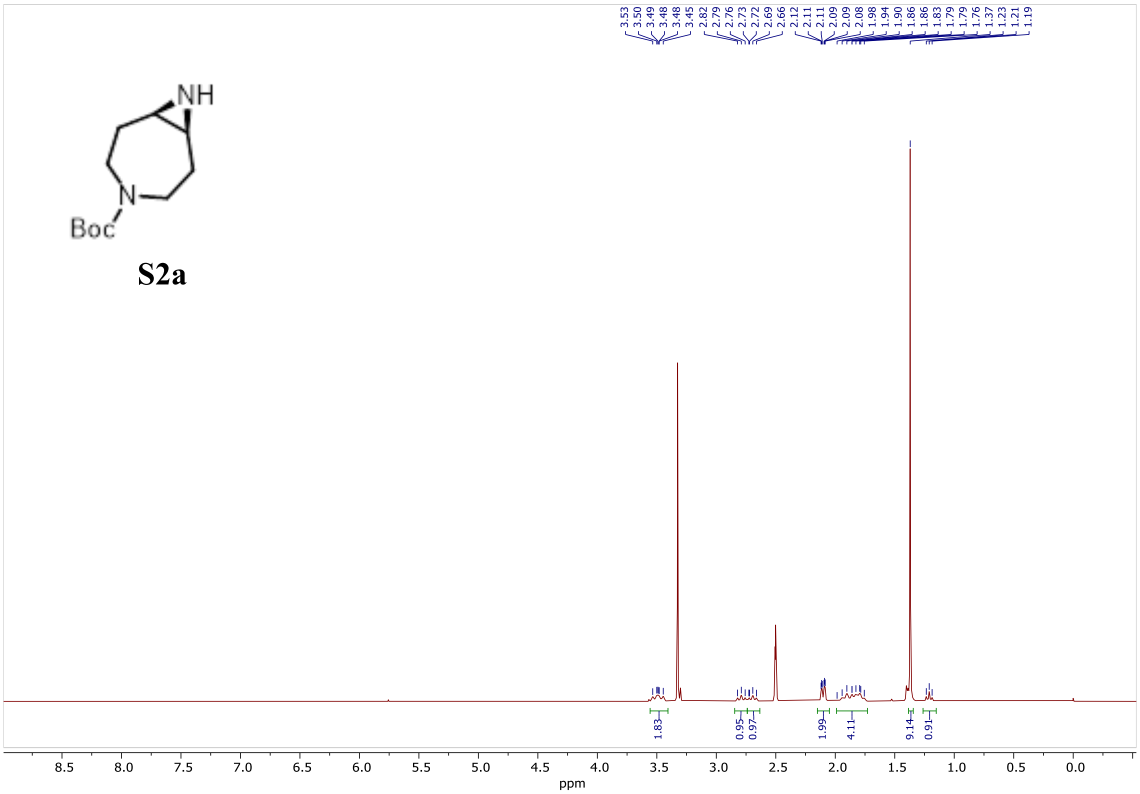

$^{13}C\{^{1}H\}$ NMR spectrum (101 MHz, DMSO-d_6, 298 K) of compound **S2a**

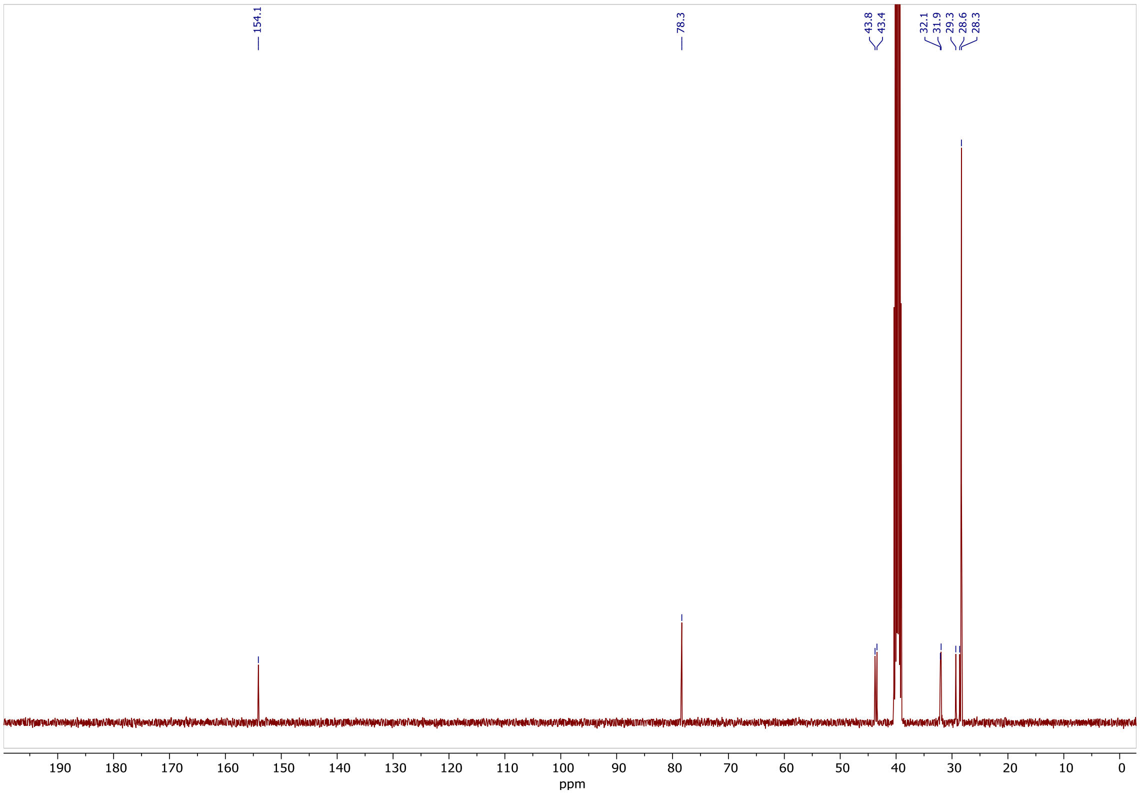

^{1}H NMR spectrum (400 MHz, DMSO-d_6, 298 K) of compound **1b**

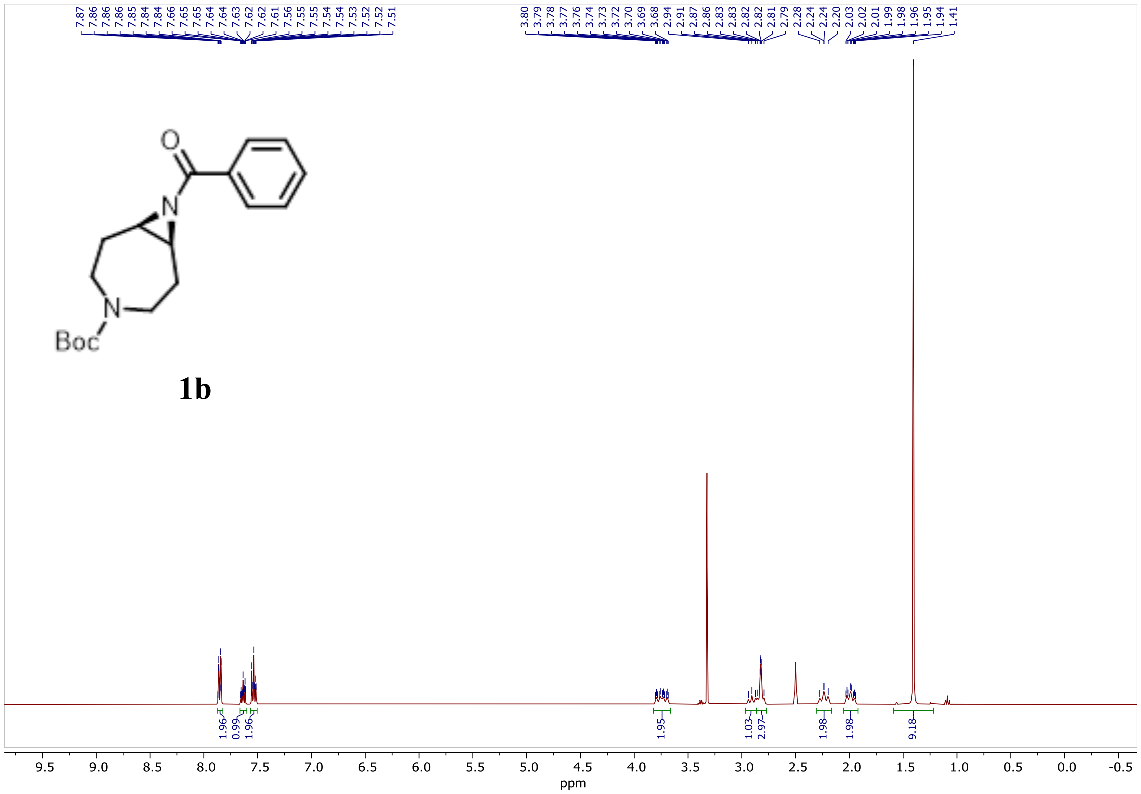

$^{13}C\{^{1}H\}$ NMR spectrum (101 MHz, DMSO-d_6, 298 K) of compound **1b**

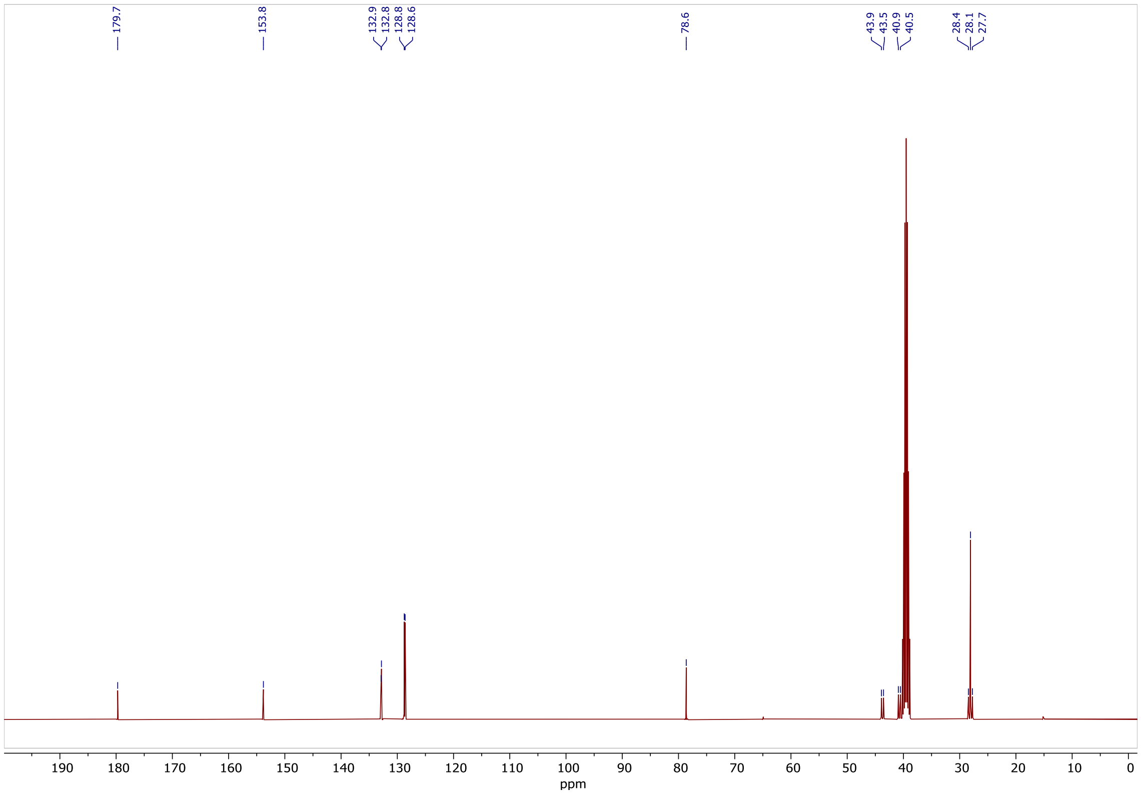

^{1}H NMR spectrum (400 MHz, $CDCl_3$, 298 K) of compound **5a**

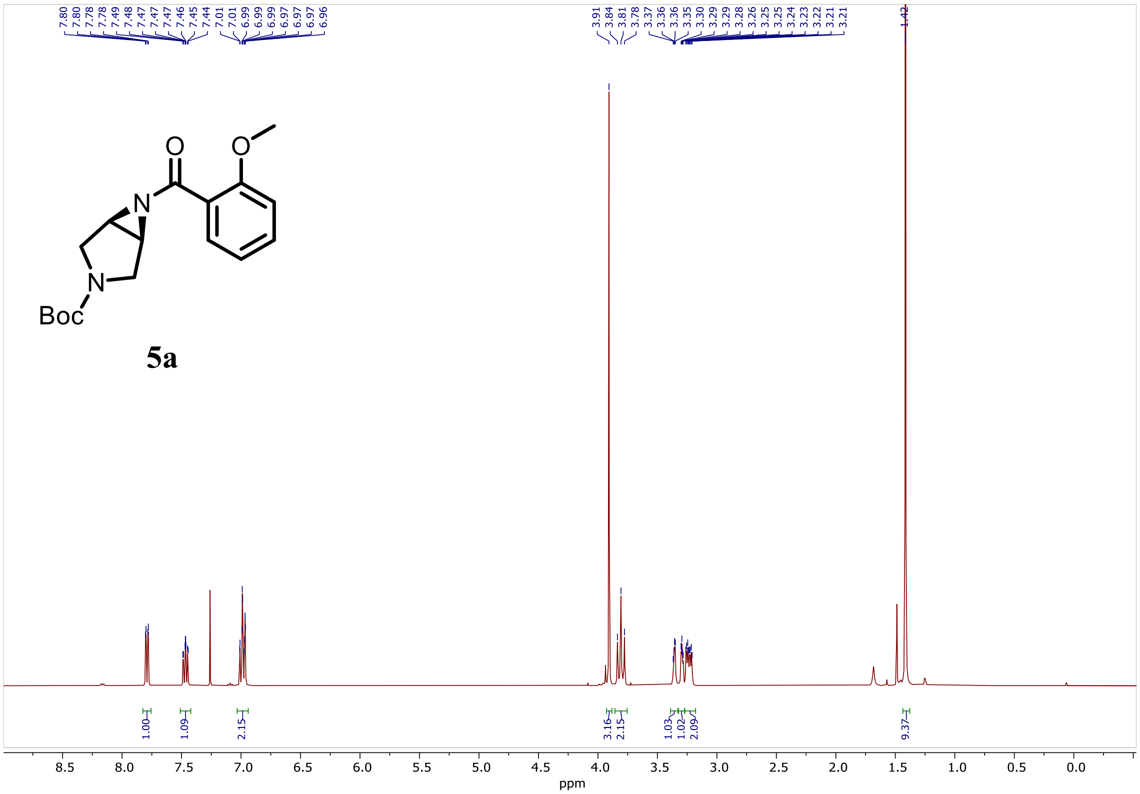

$^{13}C\{^{1}H\}$ NMR spectrum (101 MHz, $CDCl_3$, 298 K) of compound **5a**

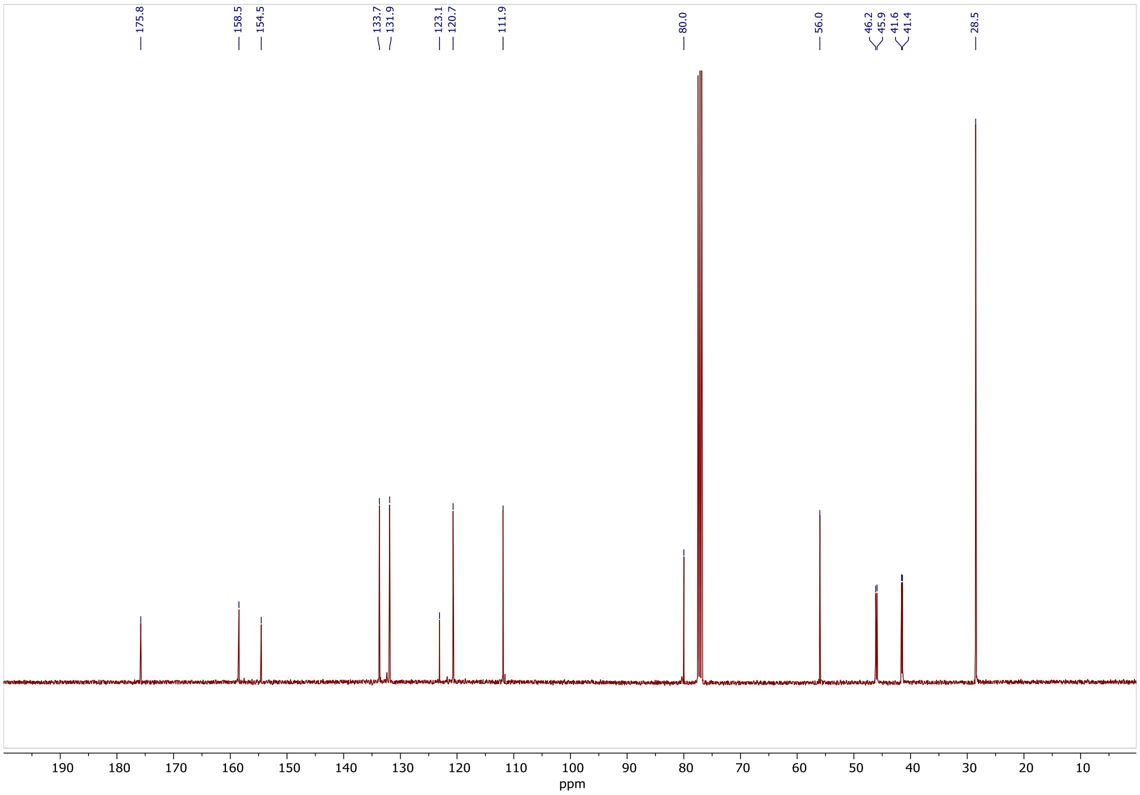

^{1}H NMR spectrum (400 MHz, $CDCl_3$, 298 K) of compound **5b**

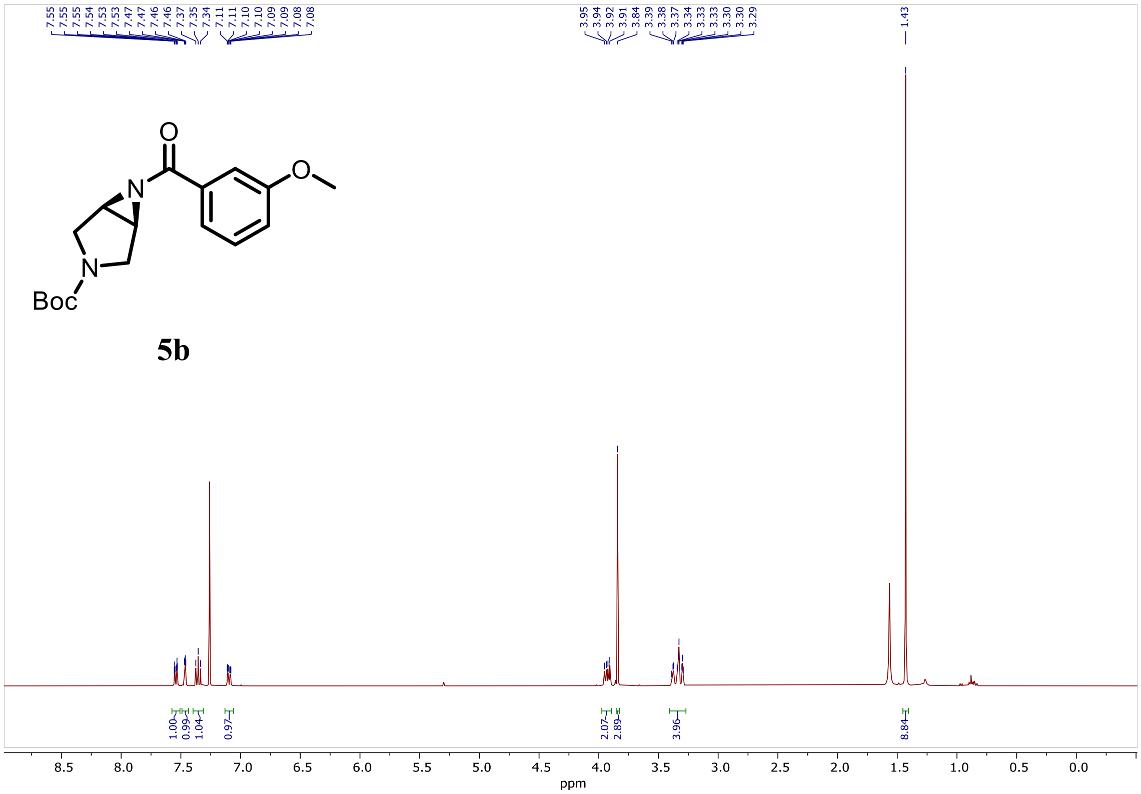

^{13}C{^{1}H} NMR spectrum (101 MHz, $CDCl_3$, 298 K) of compound **5b**

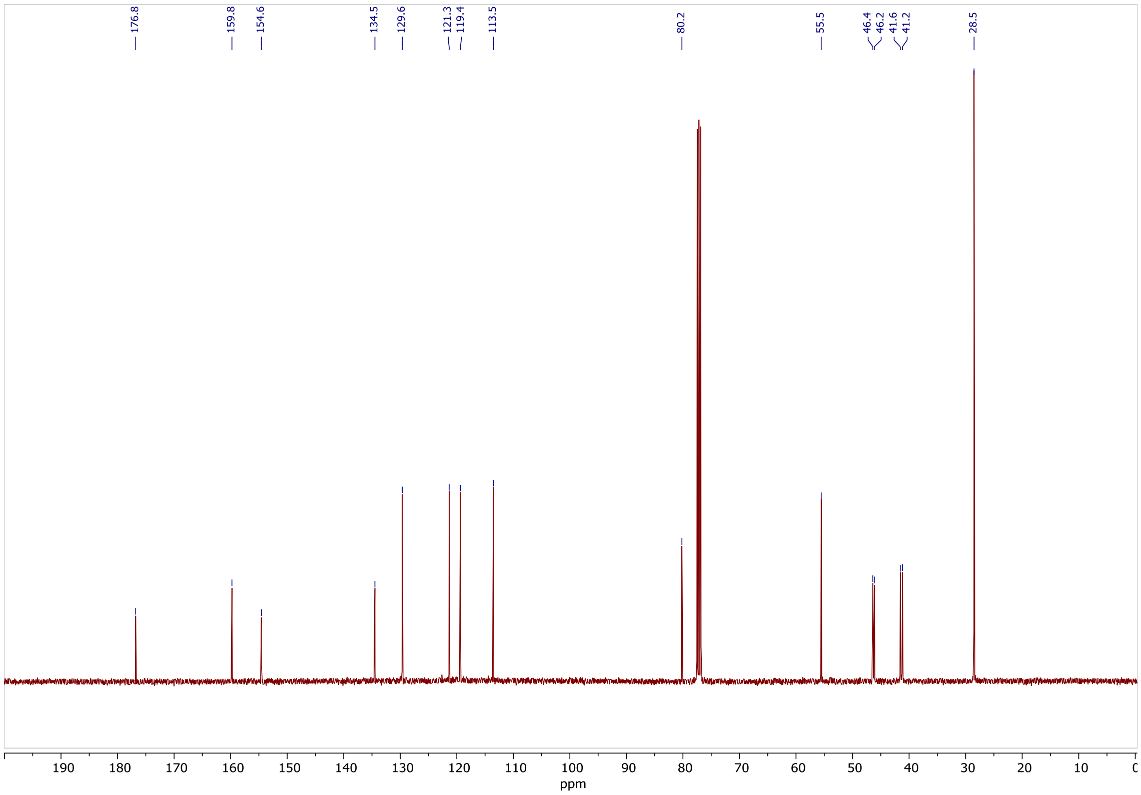

^{1}H NMR spectrum (400 MHz, $CDCl_3$, 298 K) of compound **5c**

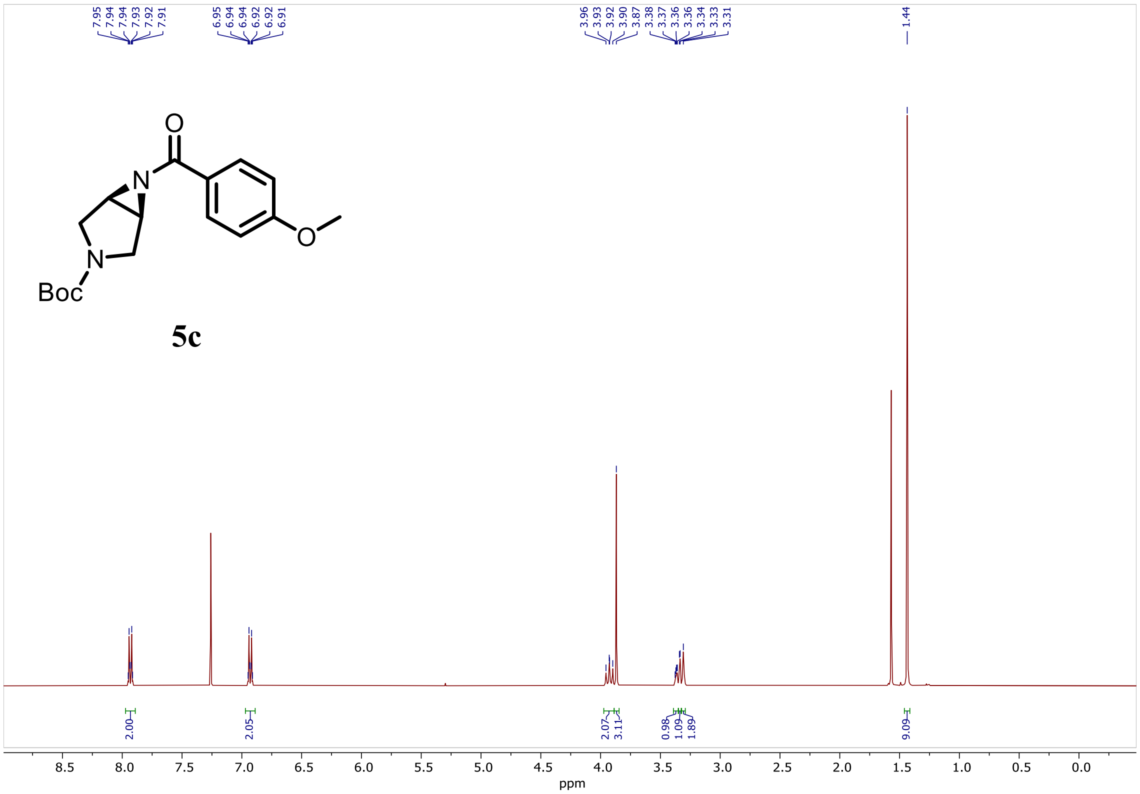

$^{13}C\{^{1}H\}$ NMR spectrum (101 MHz, $CDCl_3$, 298 K) of compound **5c**

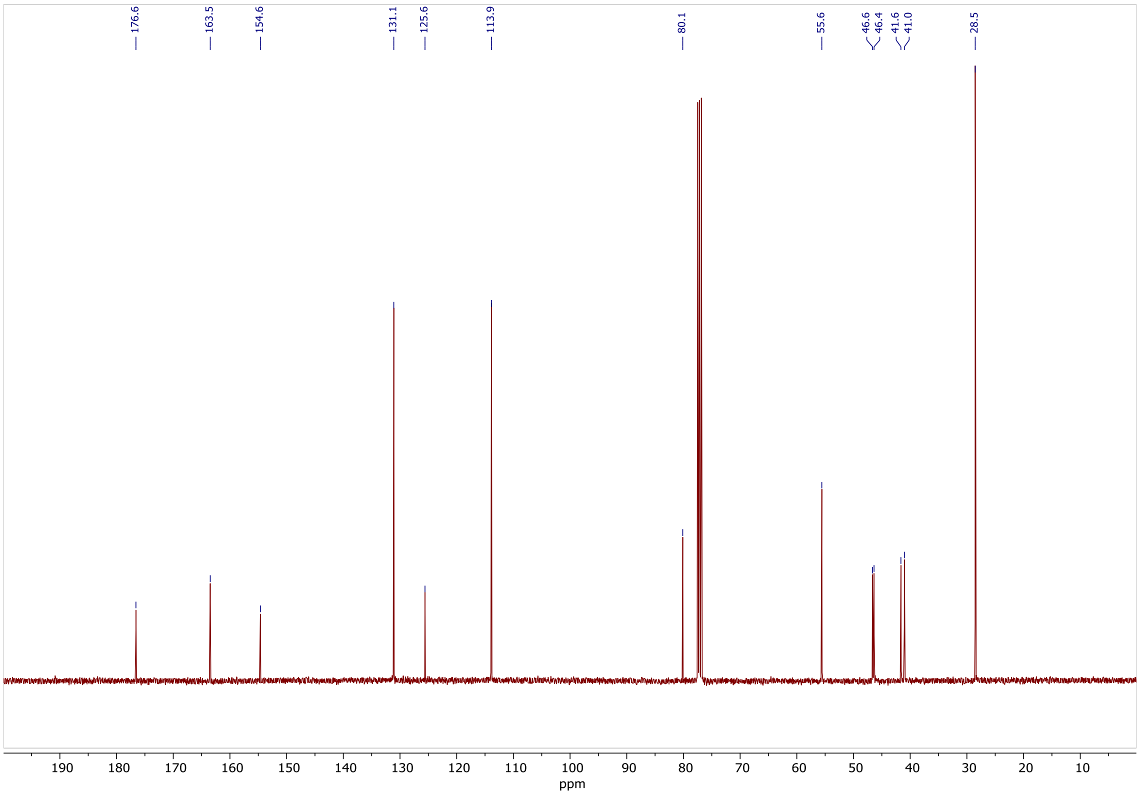

^{1}H NMR spectrum (400 MHz, $CDCl_3$, 298 K) of compound **5d**

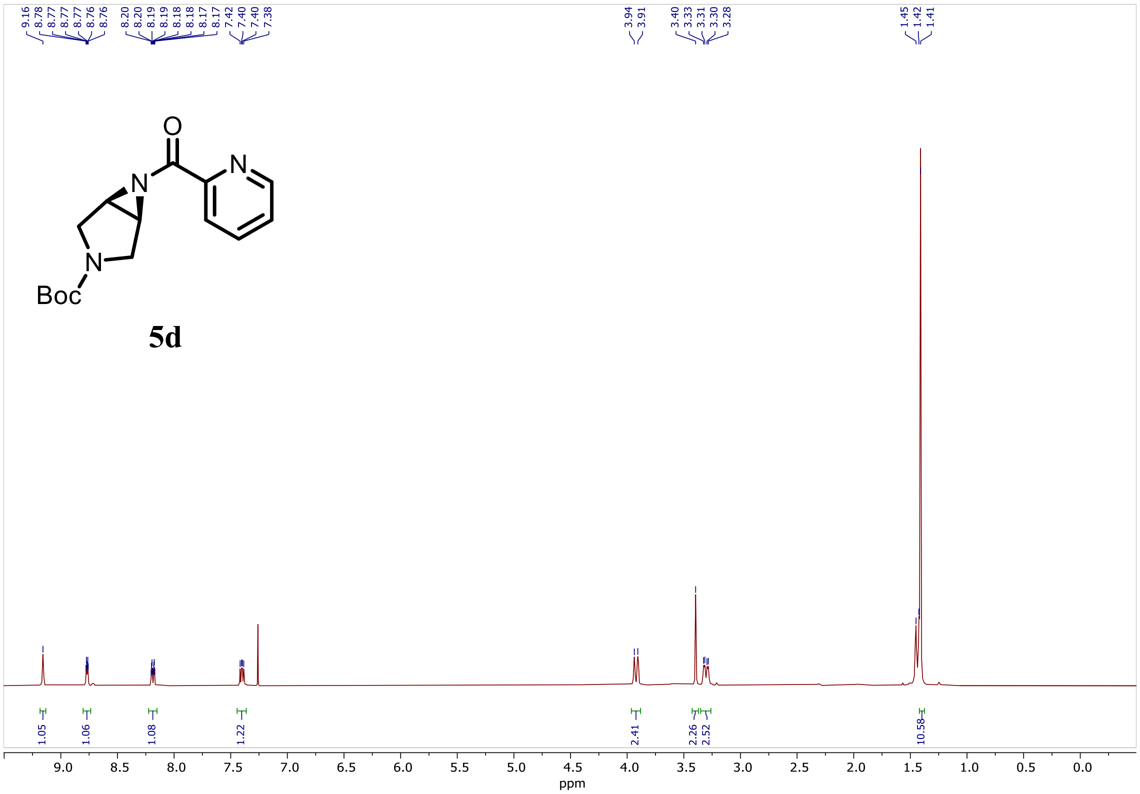

^{13}C{^{1}H} NMR spectrum (101 MHz, $CDCl_3$, 298 K) of compound **5d**

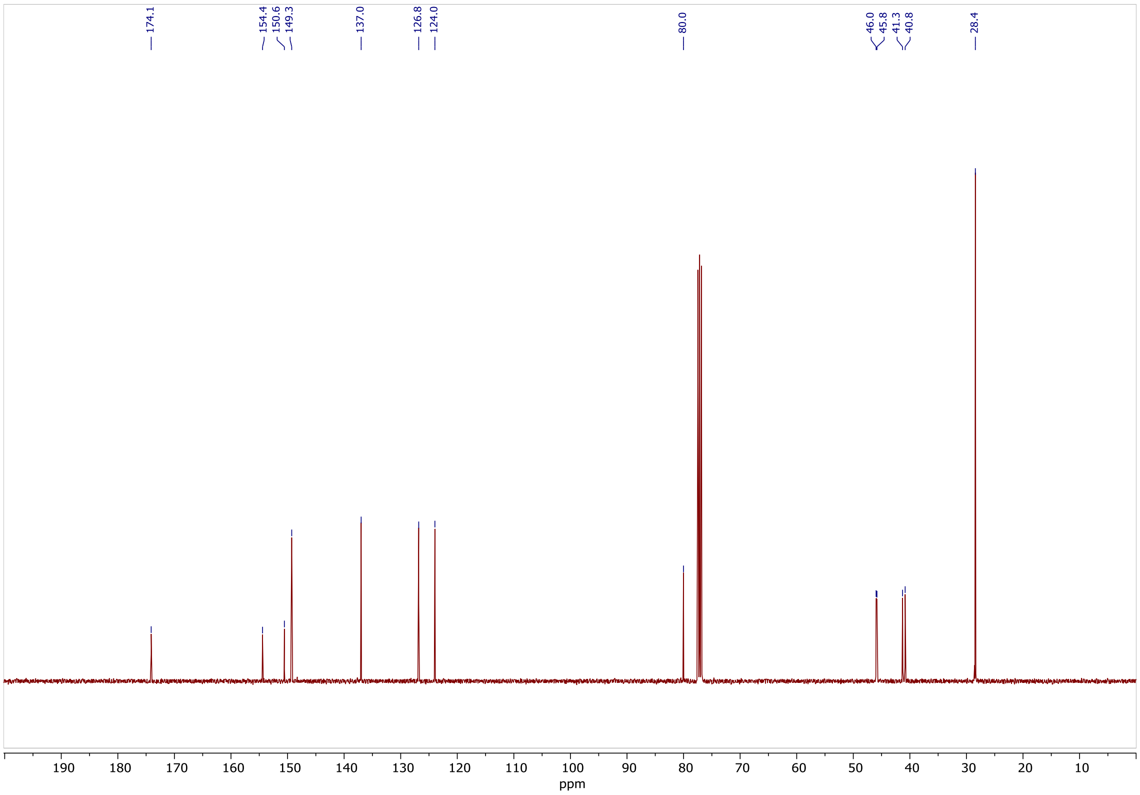

^{1}H NMR spectrum (400 MHz, $CDCl_3$, 298 K) of compound **5e**

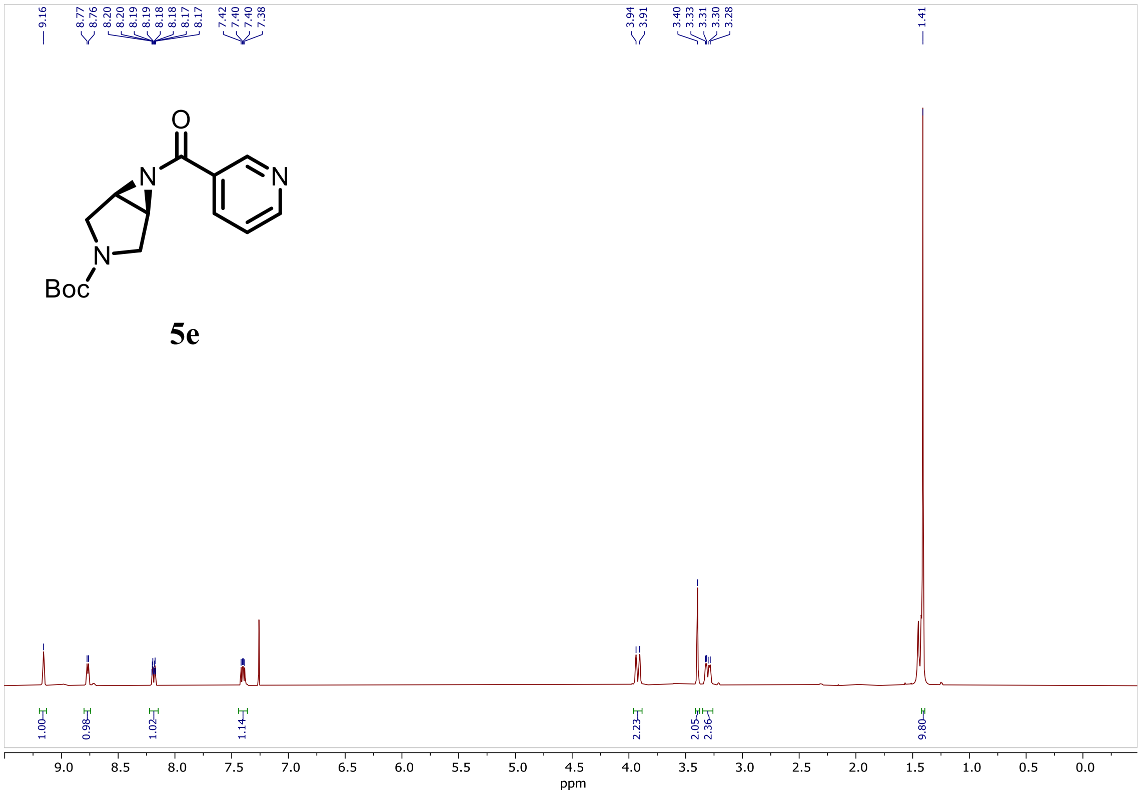

$^{13}C\{^{1}H\}$ NMR spectrum (101 MHz, $CDCl_3$, 298 K) of compound **5e**

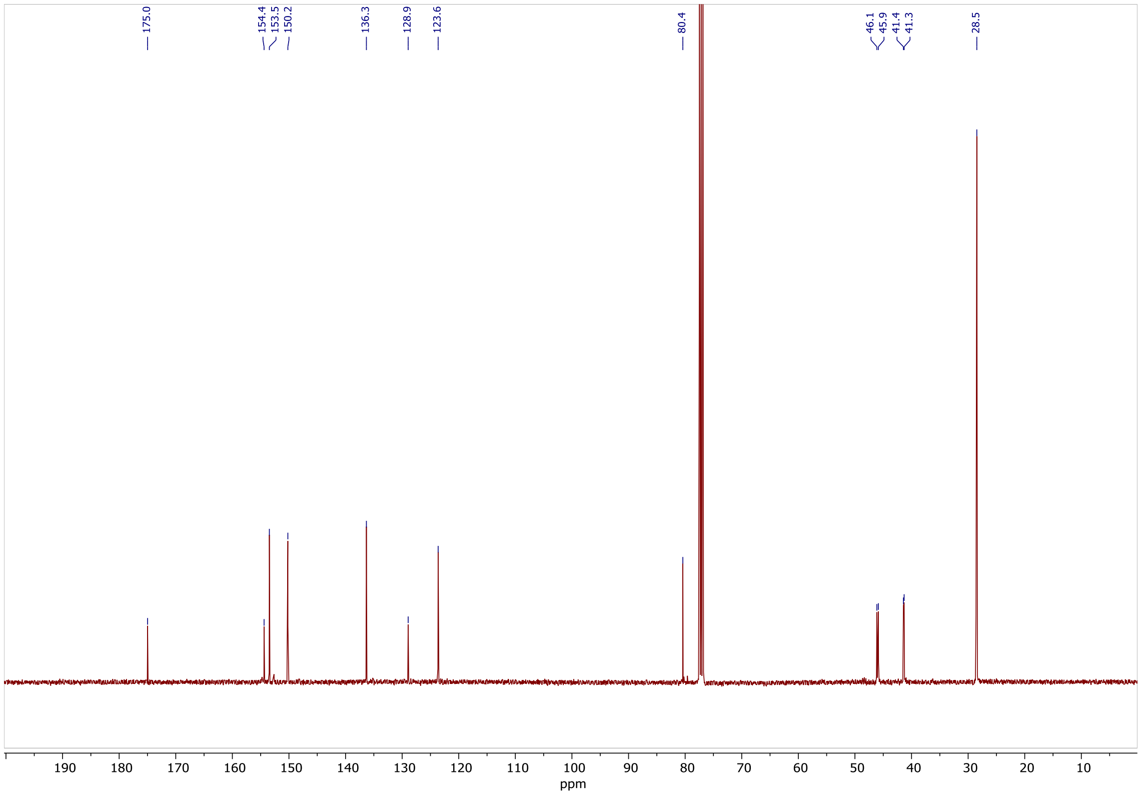

^1H NMR spectrum (400 MHz, DMSO-d_6, 343 K) of compound **5f**

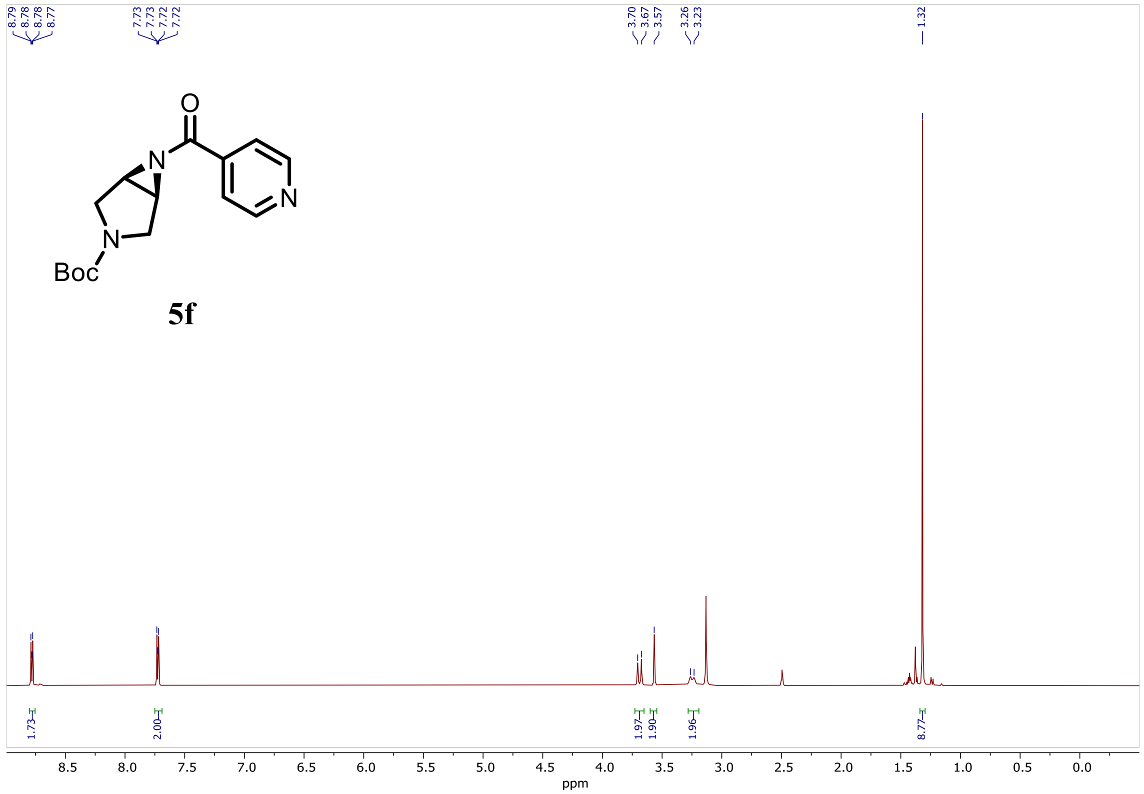

$^{13}C\{^1H\}$ NMR spectrum (101 MHz, DMSO-d_6, 343 K) of compound **5f**

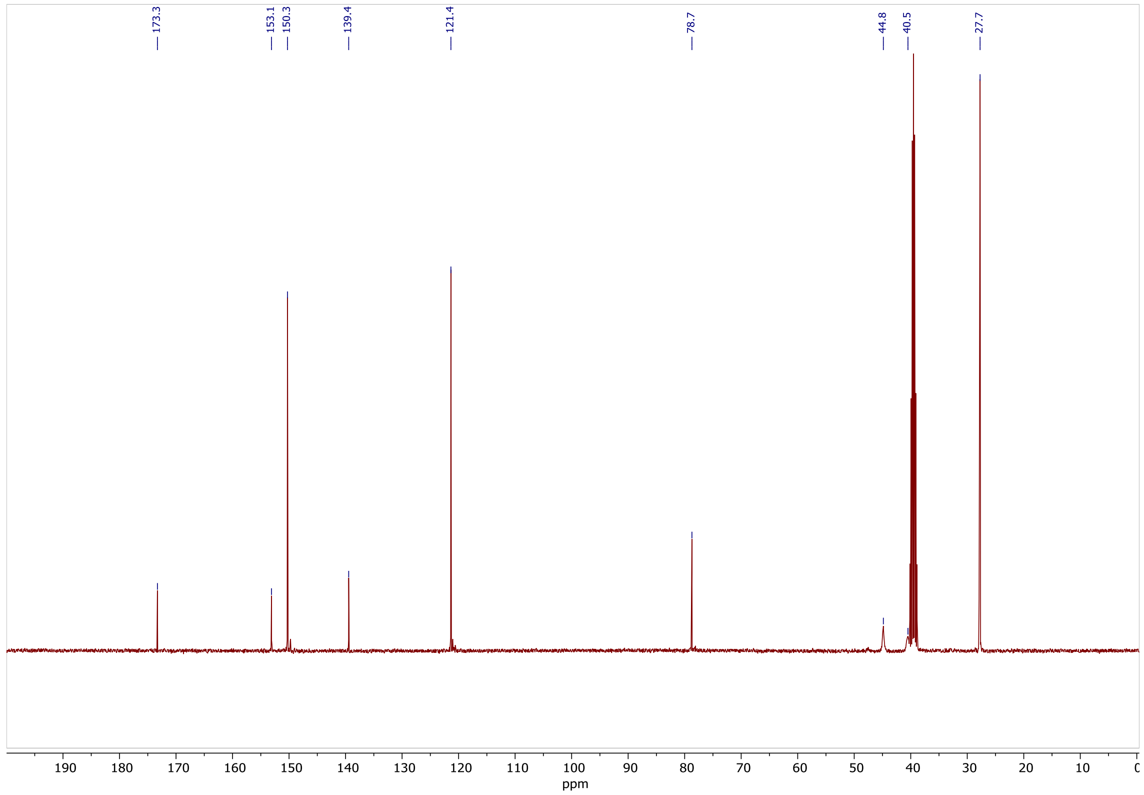

^{1}H NMR spectrum (400 MHz, $CDCl_3$, 298 K) of compound **5g**

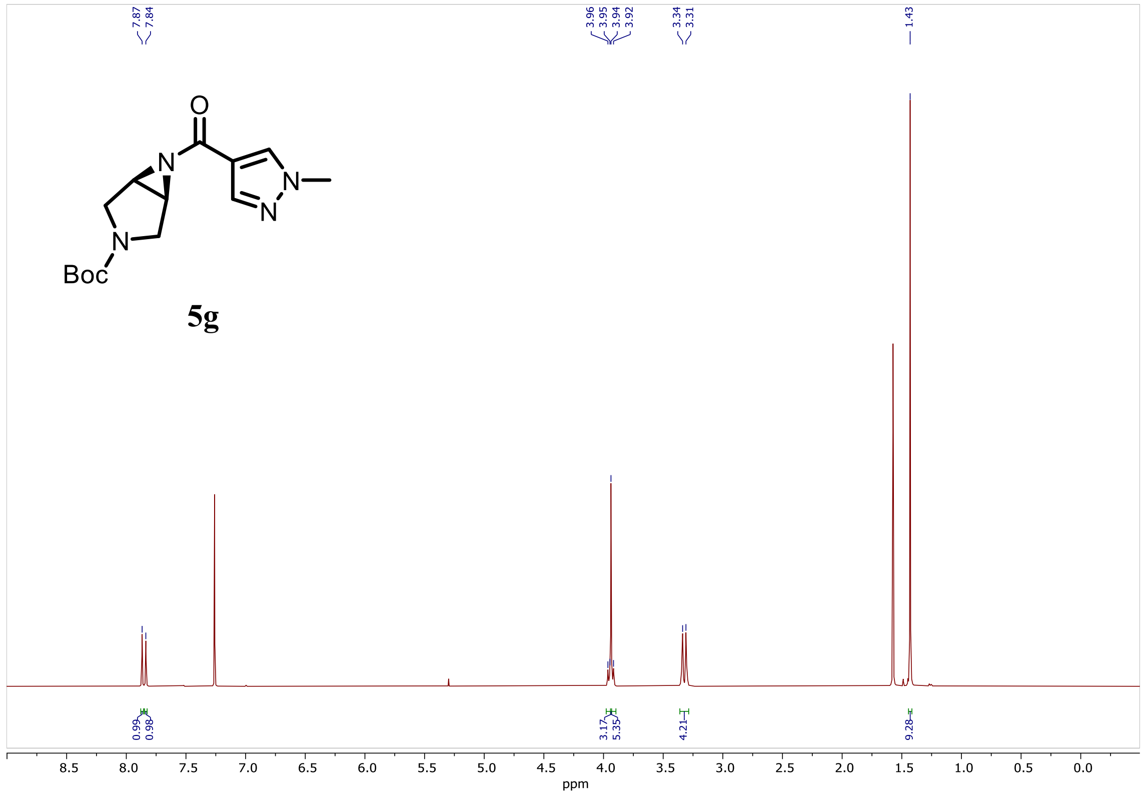

^{13}C{^{1}H} NMR spectrum (101 MHz, $CDCl_3$, 298 K) of compound **5g**

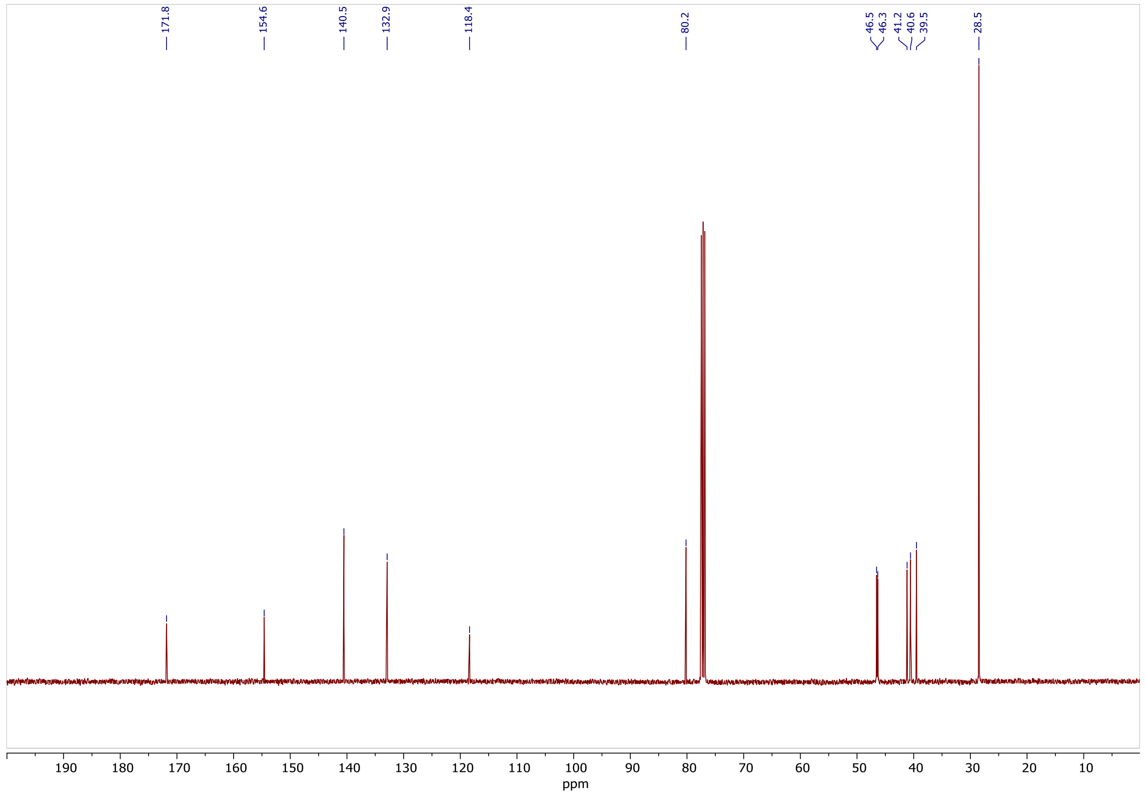

^{1}H NMR spectrum (400 MHz, $CDCl_3$, 298 K) of compound **5h**

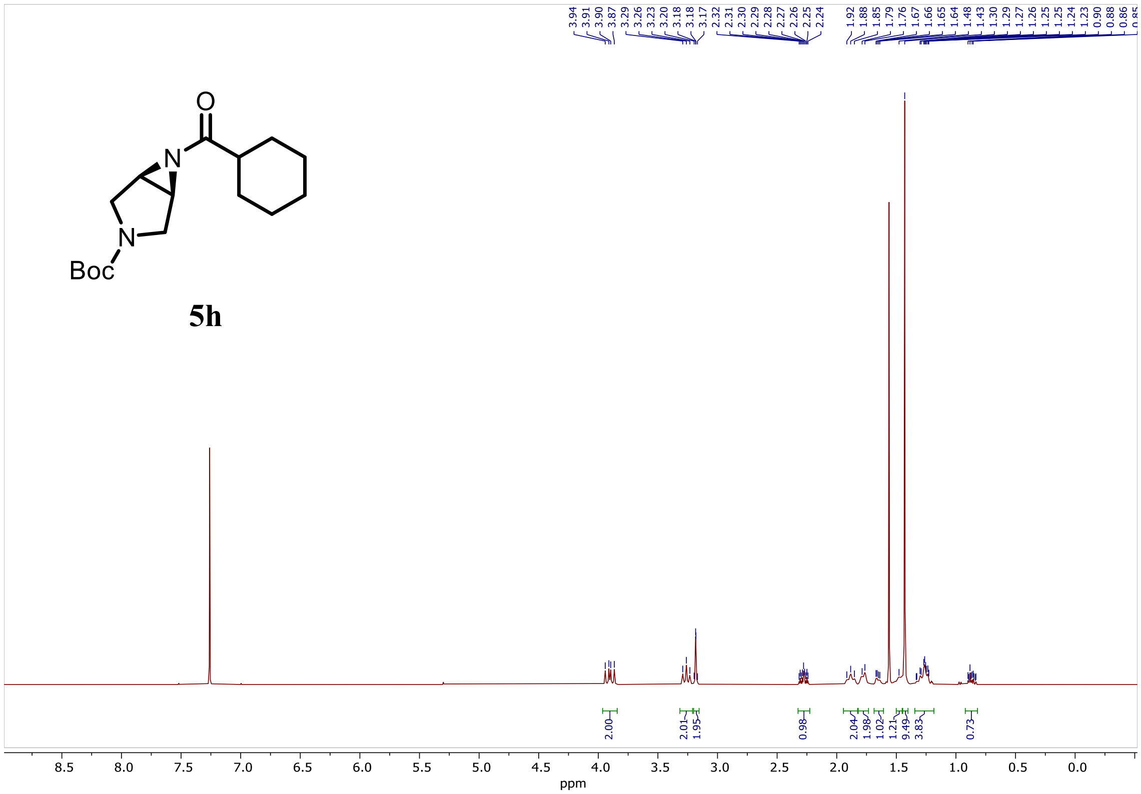

^{13}C{^{1}H} NMR spectrum (101 MHz, $CDCl_3$, 298 K) of compound **5h**

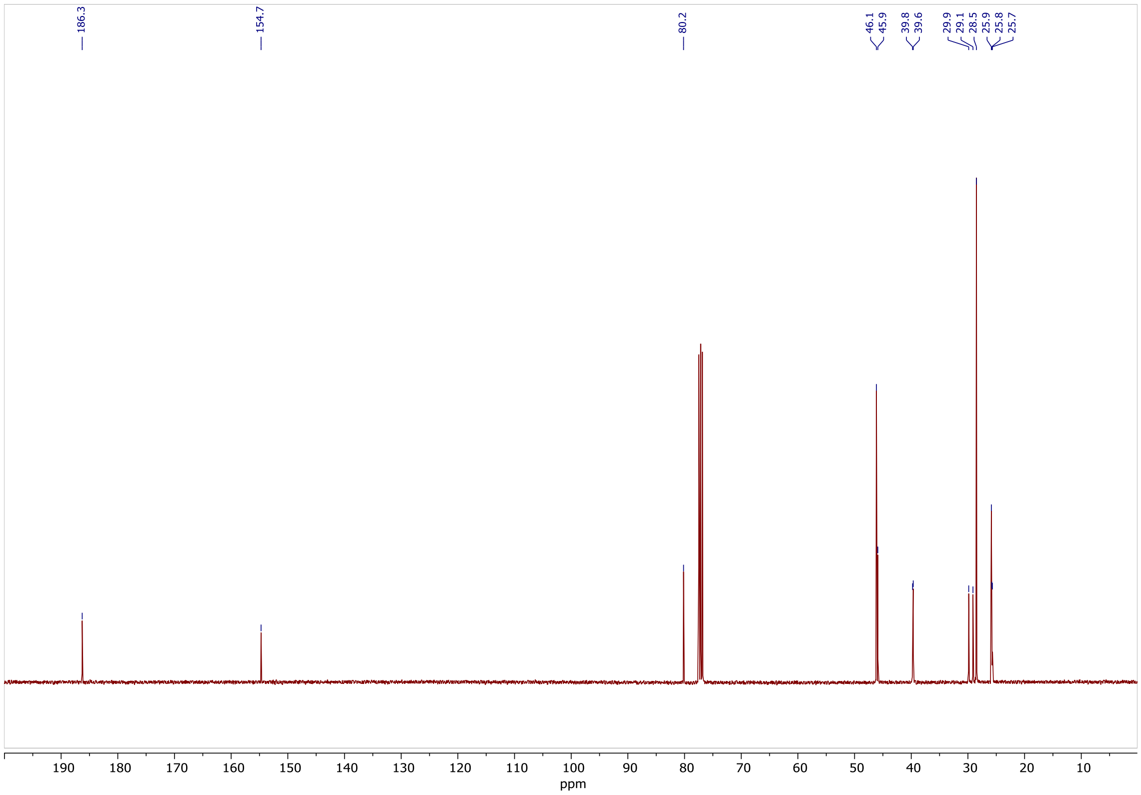

^{1}H NMR spectrum (400 MHz, $CDCl_3$, 298 K) of compound **5i**

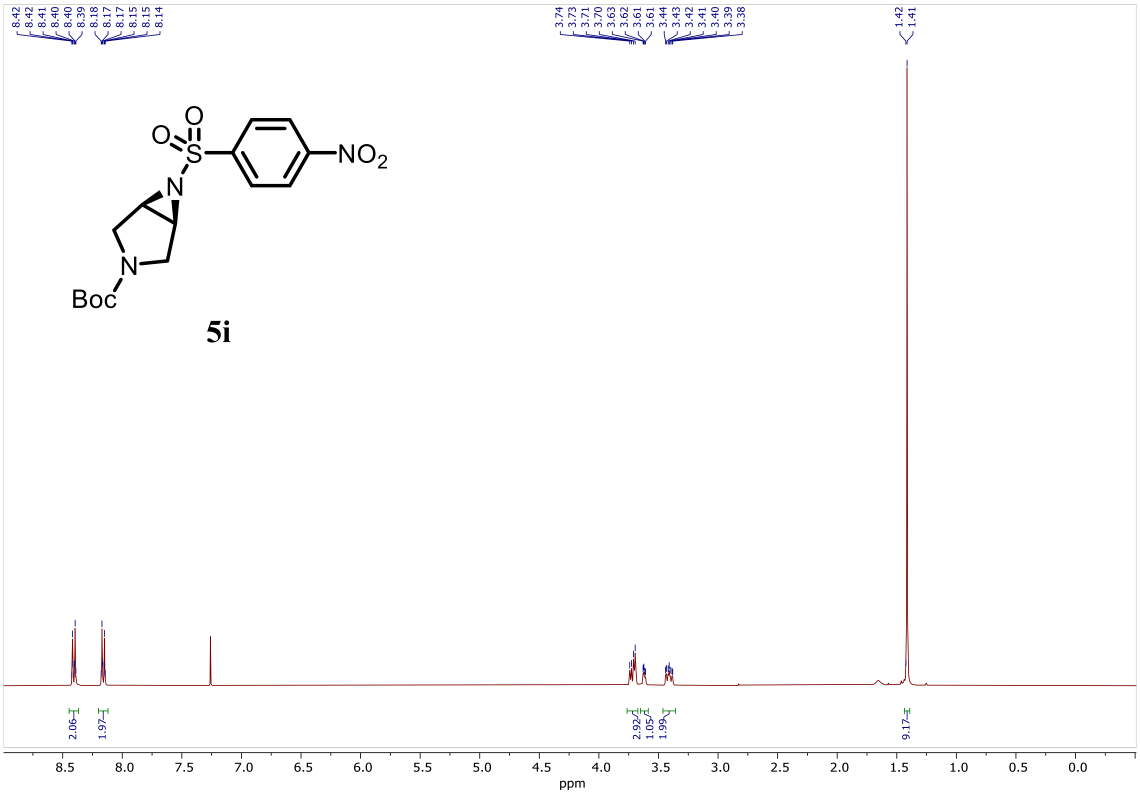

$^{13}C\{^{1}H\}$ NMR spectrum (101 MHz, $CDCl_3$, 298 K) of compound **5i**

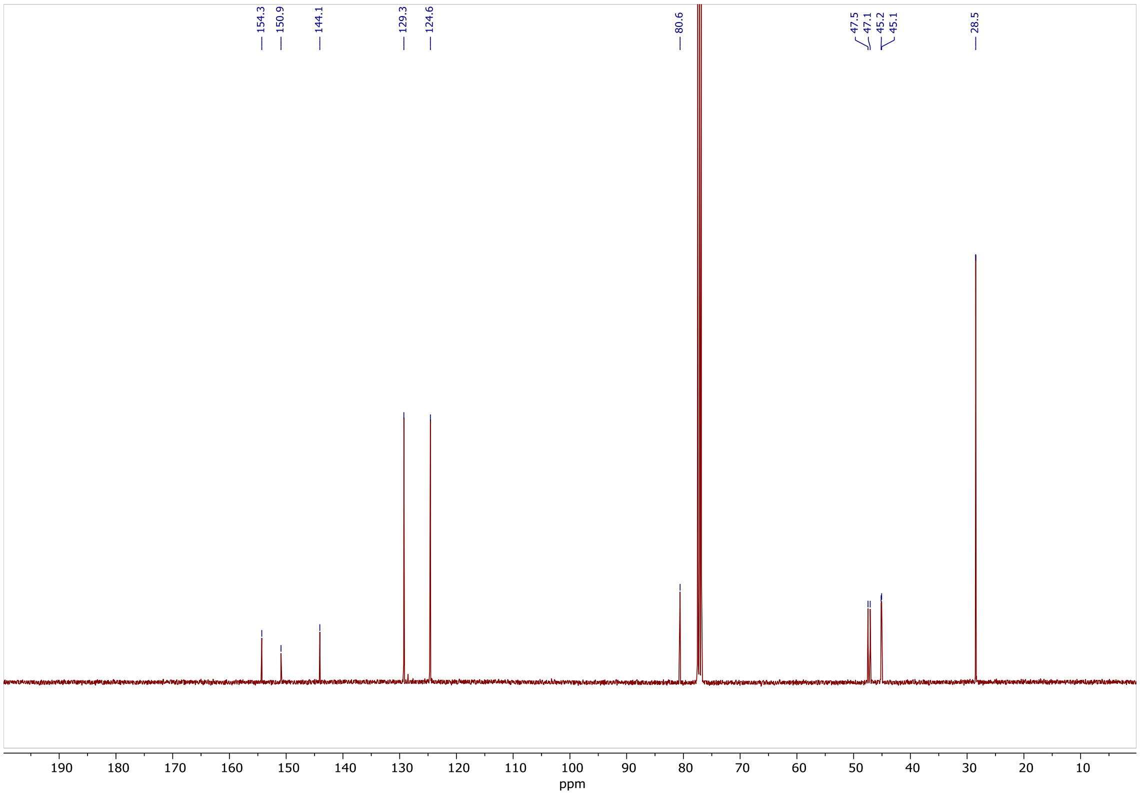

^{1}H NMR spectrum (400 MHz, $CDCl_3$, 298 K) of compound **S2f**

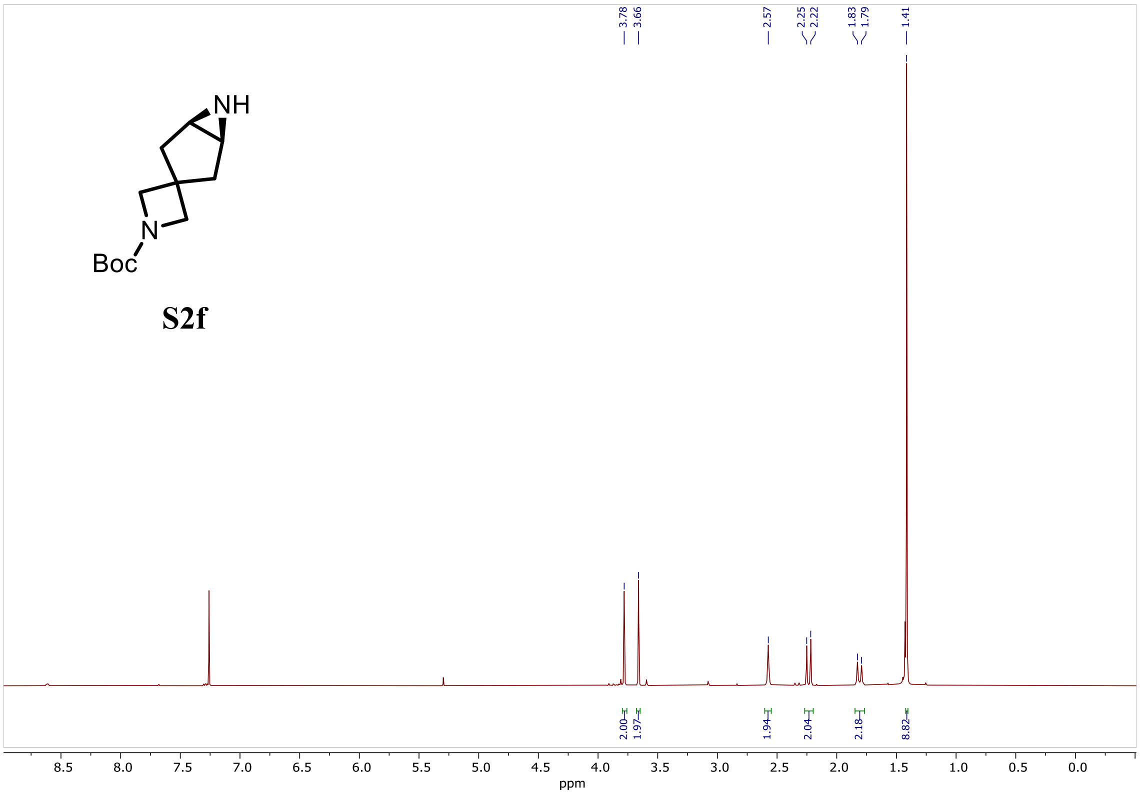

^{13}C{^{1}H} NMR spectrum (101 MHz, $CDCl_3$, 298 K) of compound **S2f**

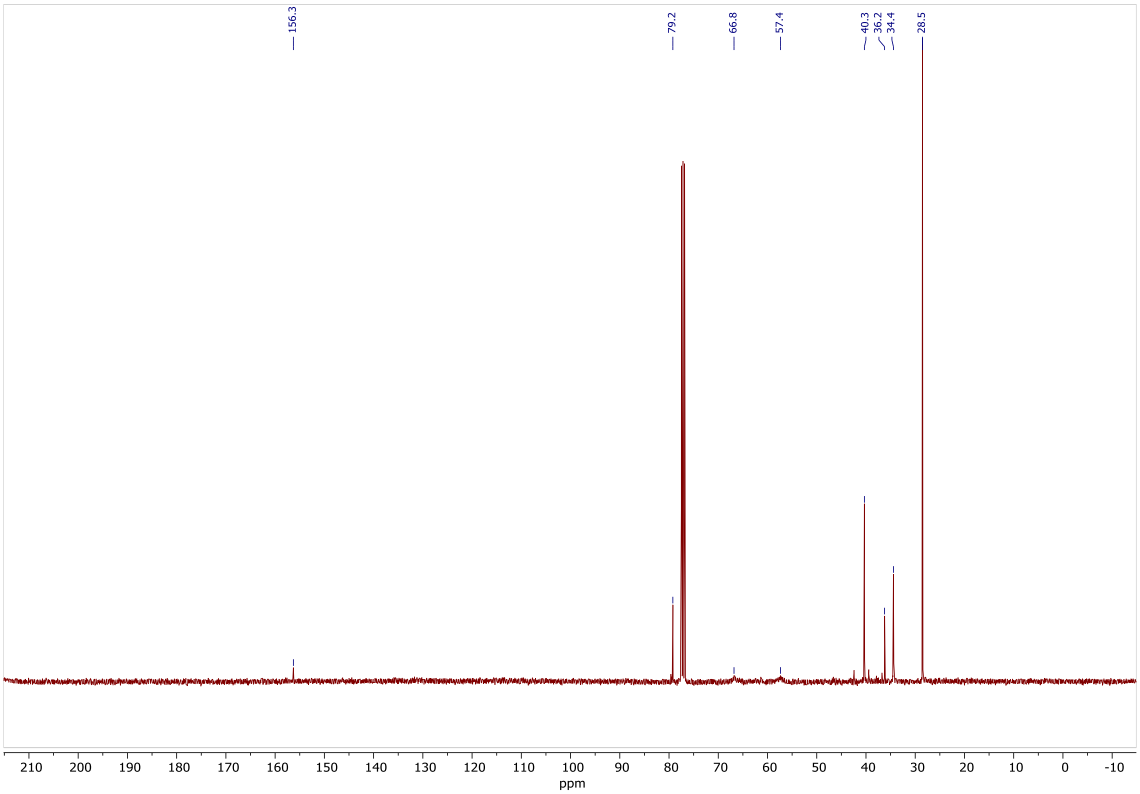

^{1}H NMR spectrum (400 MHz, $CDCl_3$, 298 K) of compound **5j**

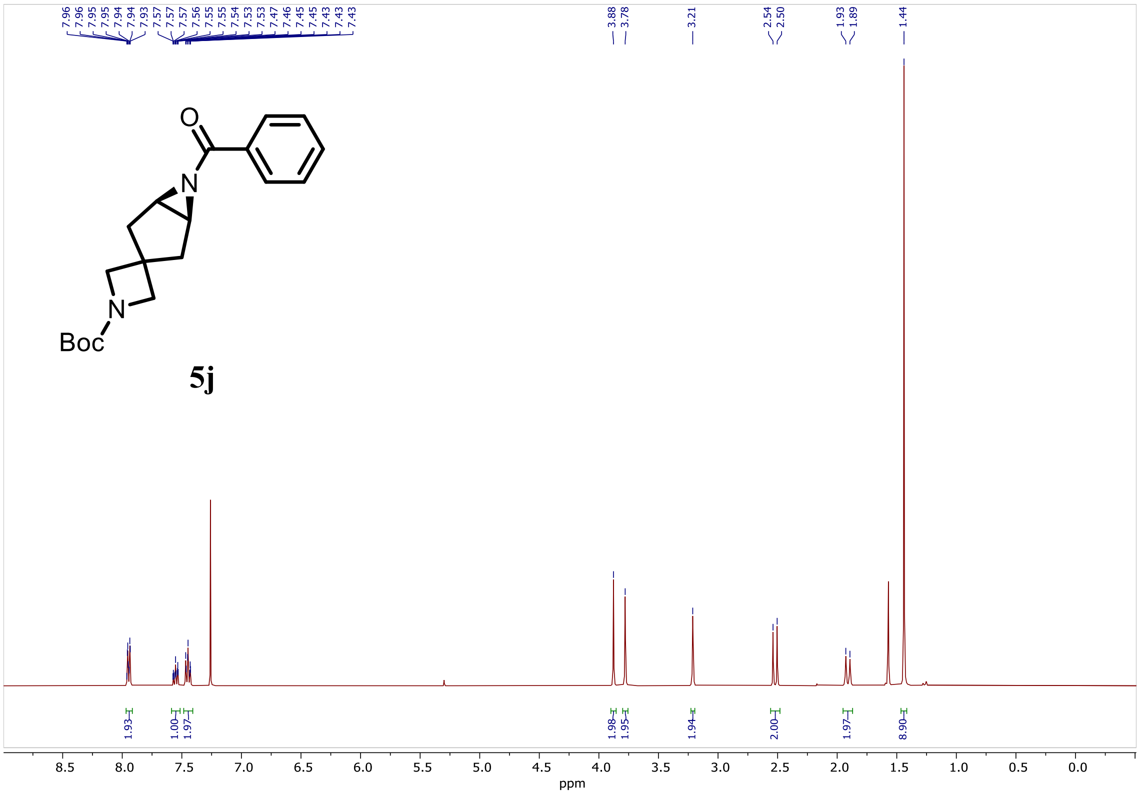

$^{13}C\{^{1}H\}$ NMR spectrum (101 MHz, $CDCl_3$, 298 K) of compound **5j**

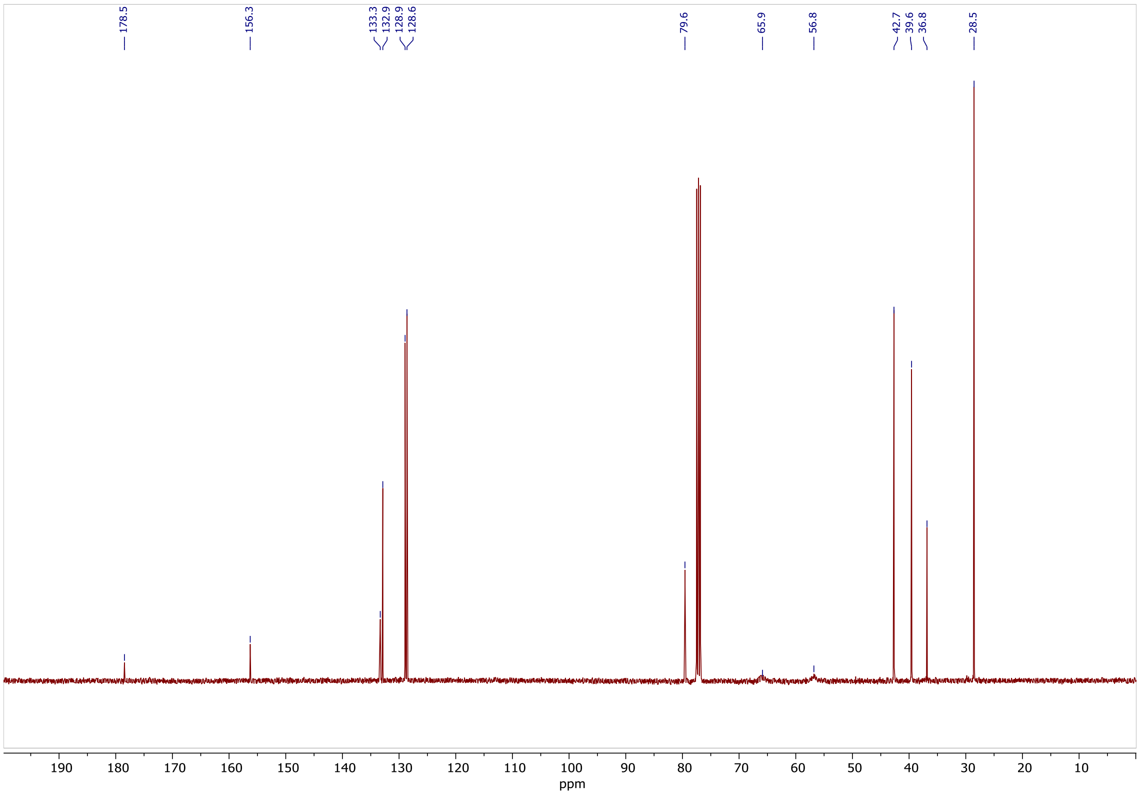

^{1}H NMR spectrum (400 MHz, DMSO-d_6, 343 K) of compound **5k**

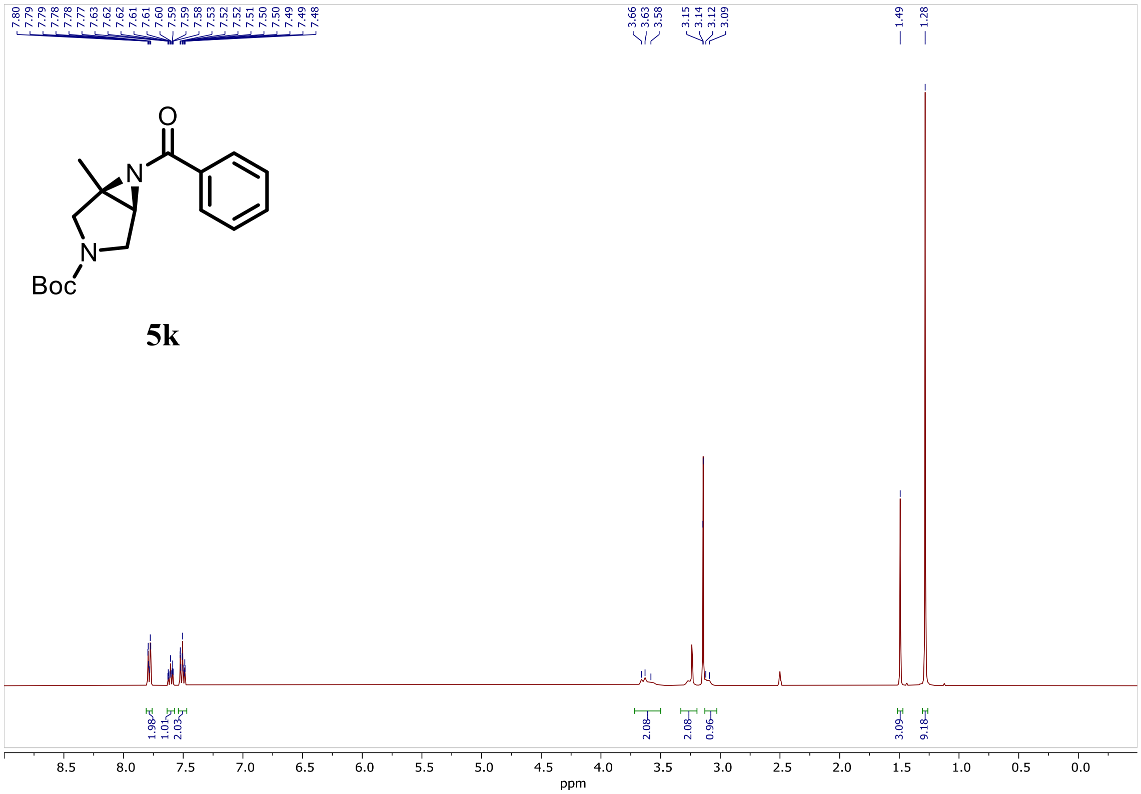

^{13}C{^{1}H} NMR spectrum (101 MHz, DMSO-d_6, 343 K) of compound **5k**

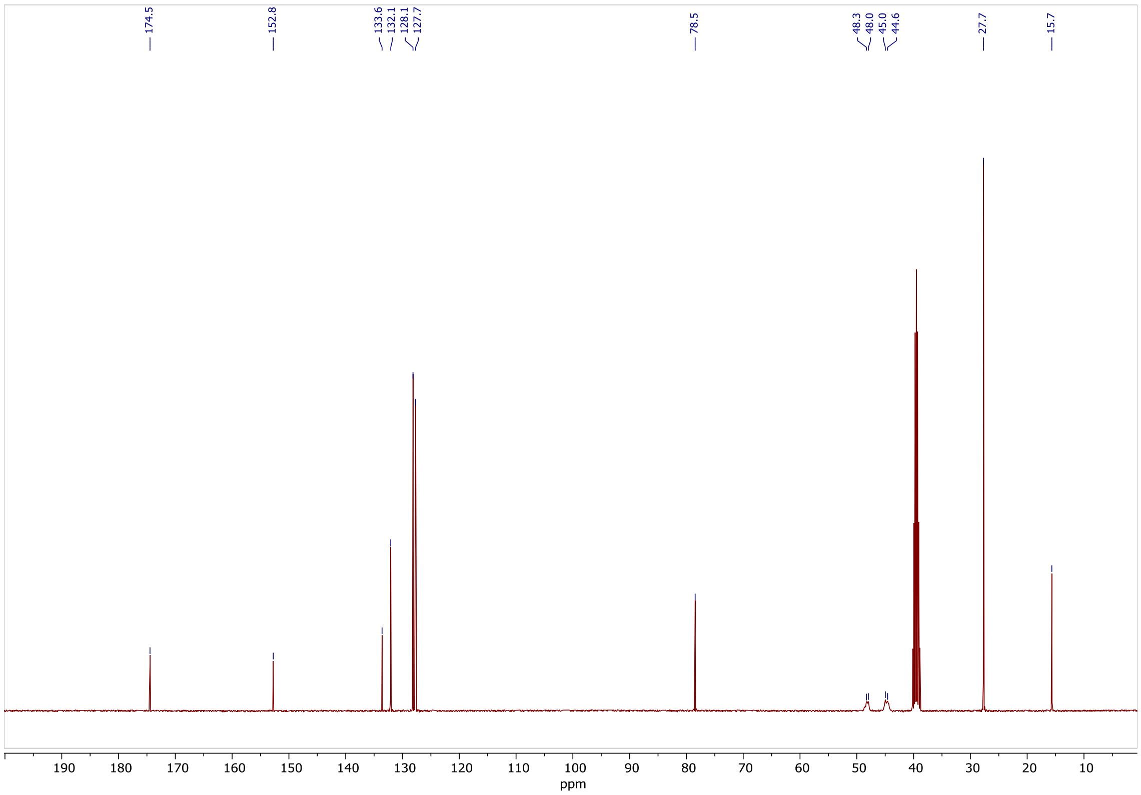

HSQC NMR spectrum (400 MHz, 101 MHz, DMSO-d_6, 343 K) of compound **5k**

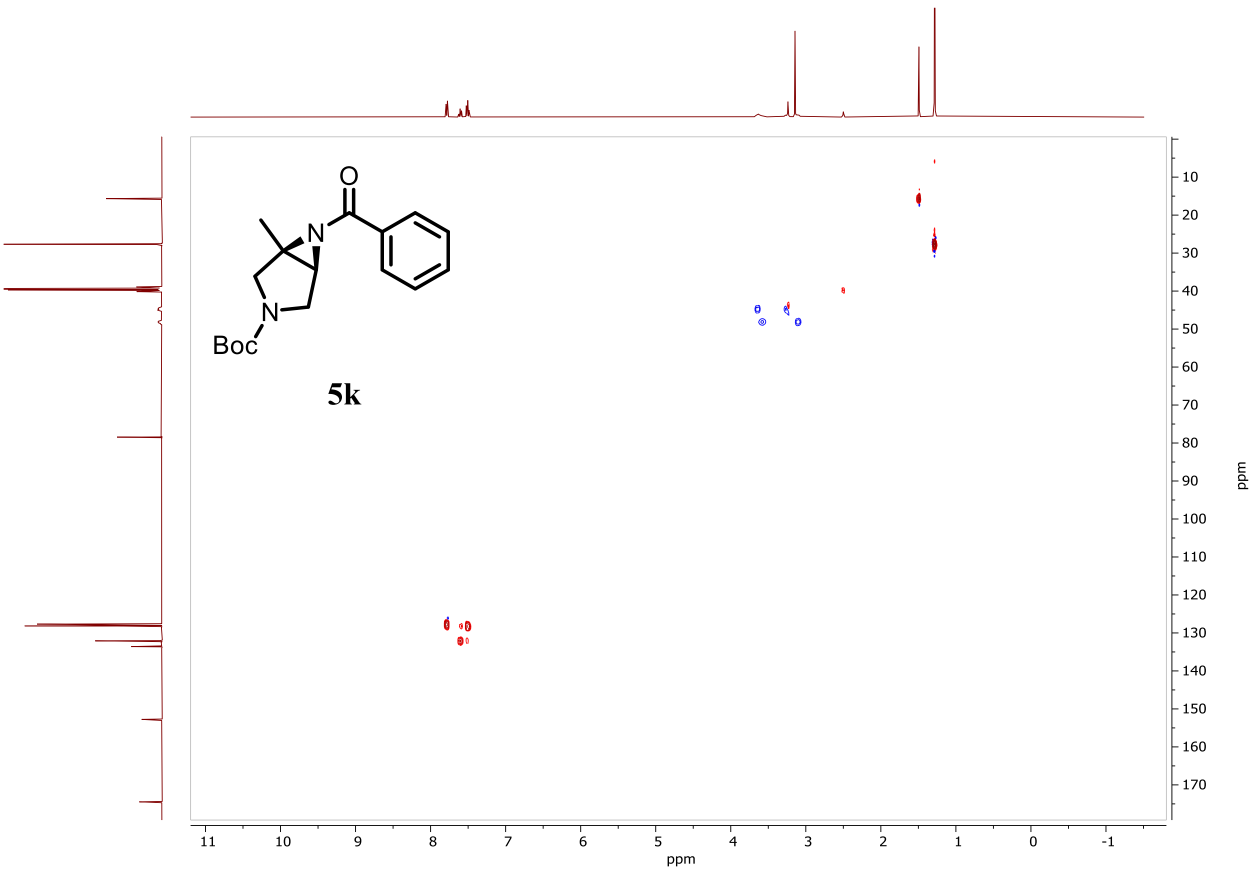

^{1}H NMR spectrum (400 MHz, DMSO-d_6, 343 K) of compound **S2d**

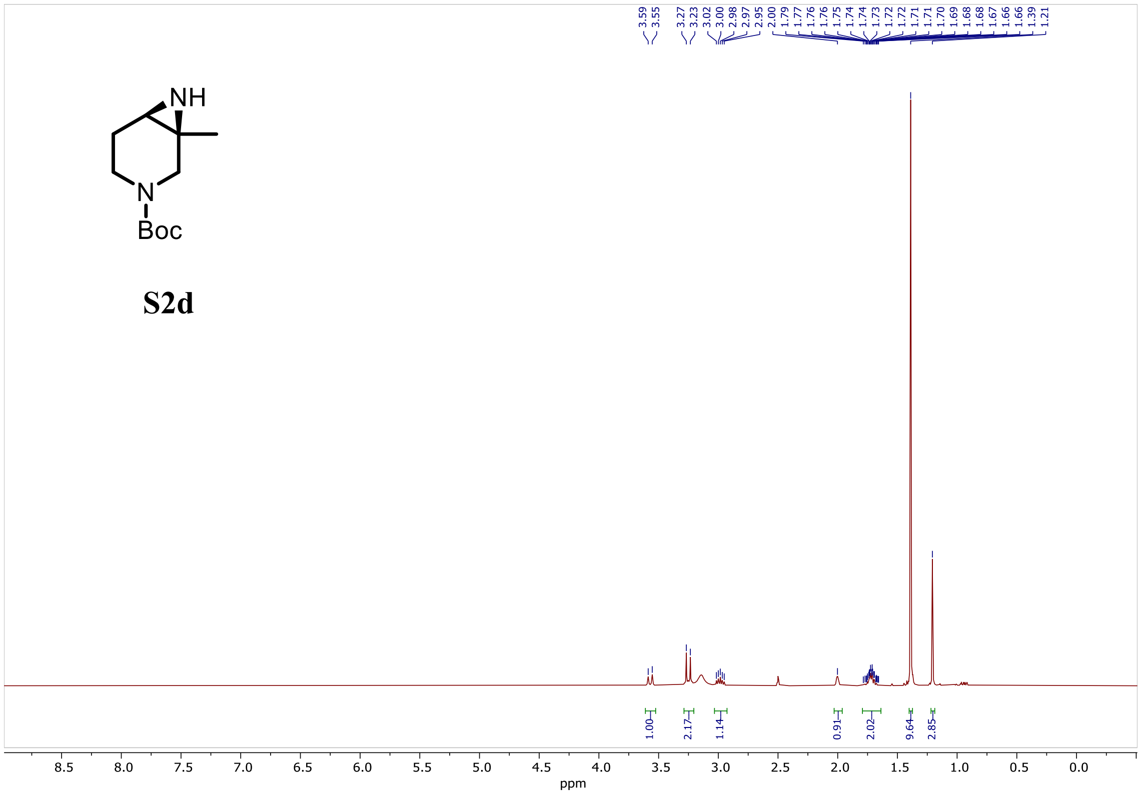

$^{13}C\{^{1}H\}$ NMR spectrum (101 MHz, DMSO-d_6, 343 K) of compound **S2d**

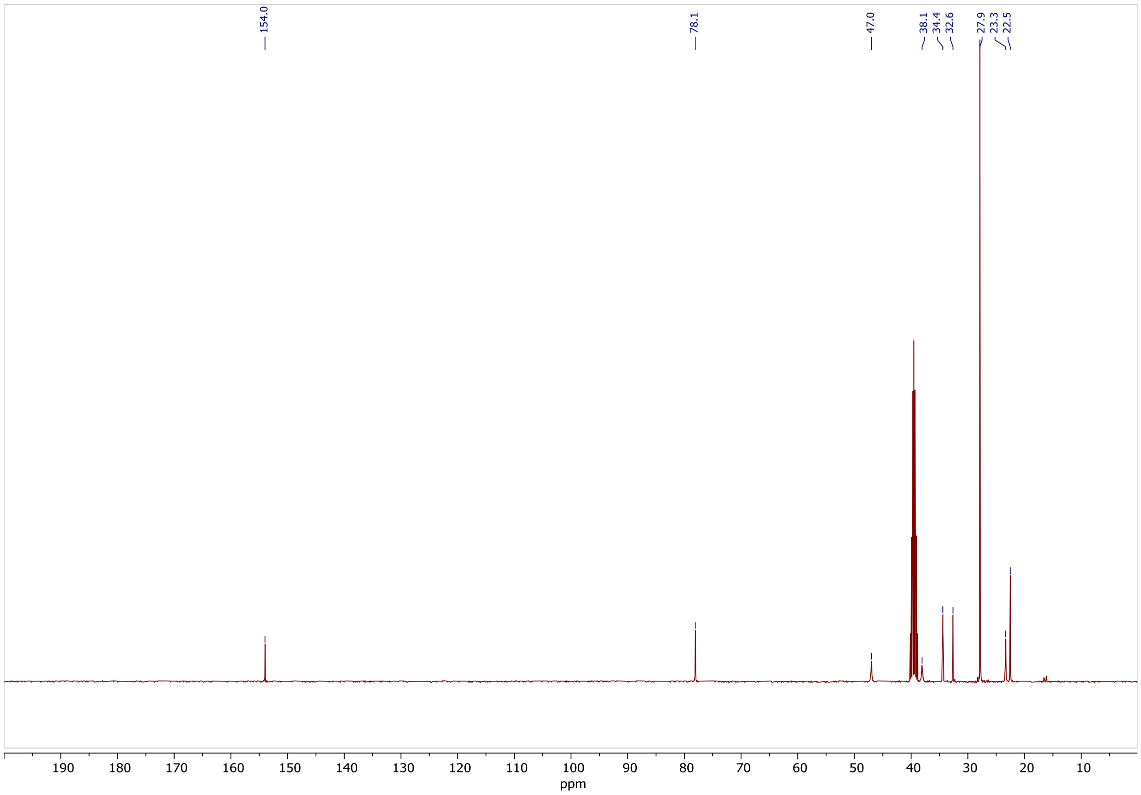

^{1}H NMR spectrum (400 MHz, DMSO-d_6, 343 K) of compound **5m**

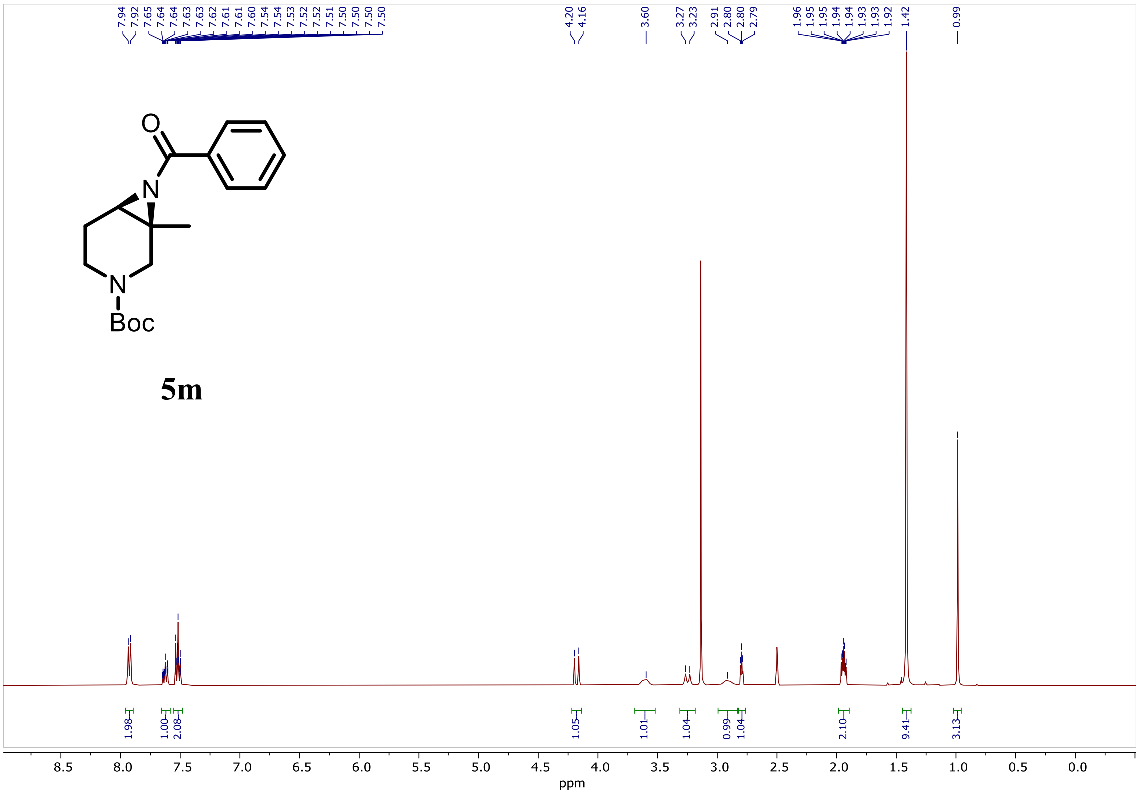

^{13}C{^{1}H} NMR spectrum (101 MHz, DMSO-d_6, 343 K) of compound **5m**

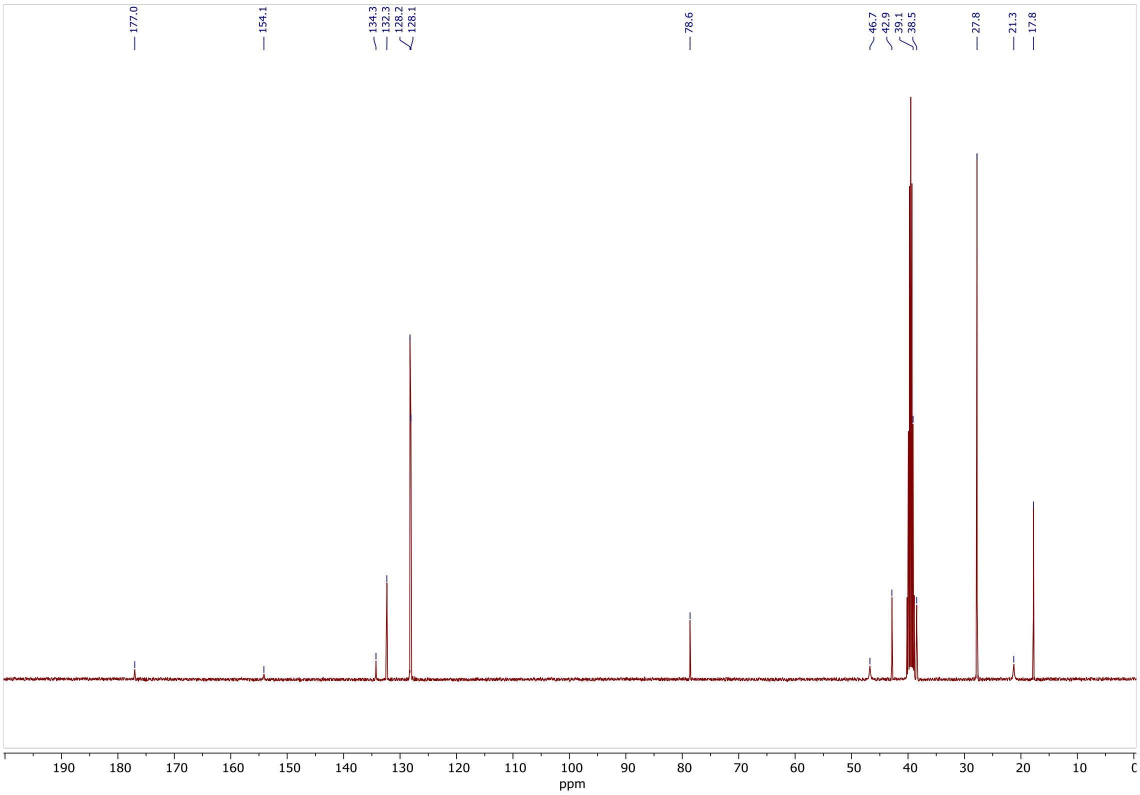

^{1}H NMR spectrum (400 MHz, $CDCl_3$, 298 K) of compound **S1a**

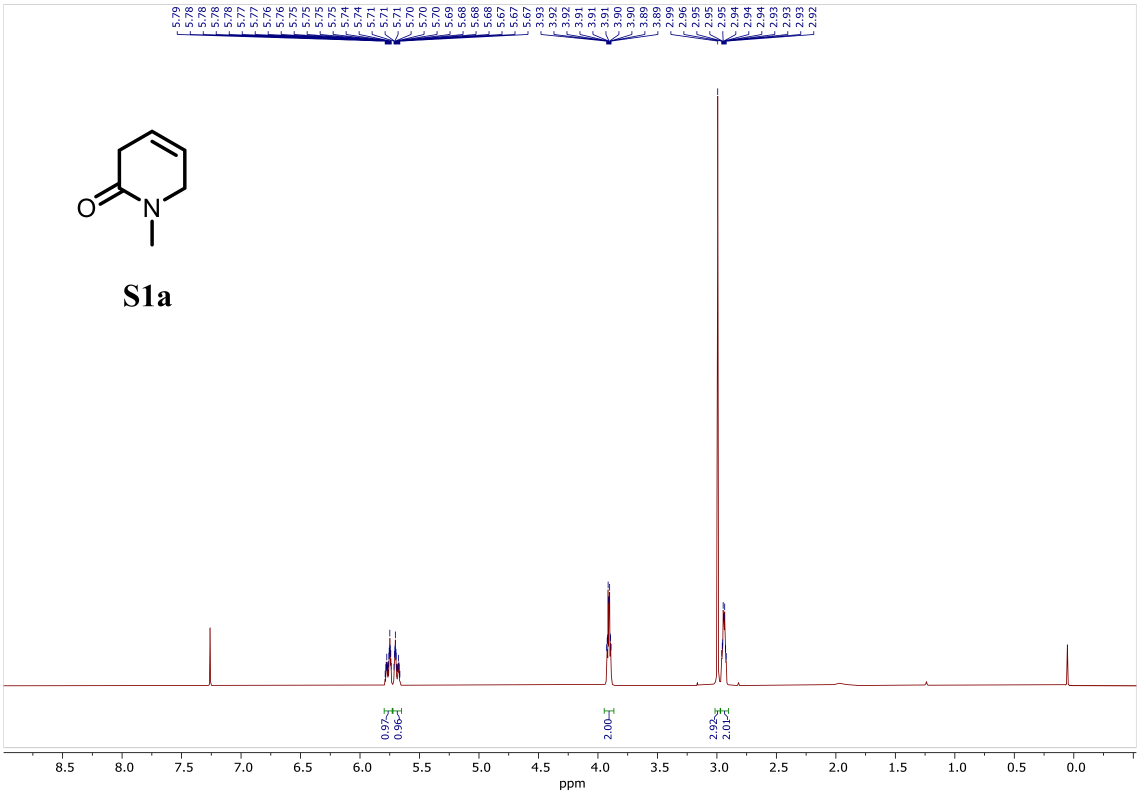

$^{13}C\{^{1}H\}$ NMR spectrum (101 MHz, $CDCl_3$, 298 K) of compound **S1a**

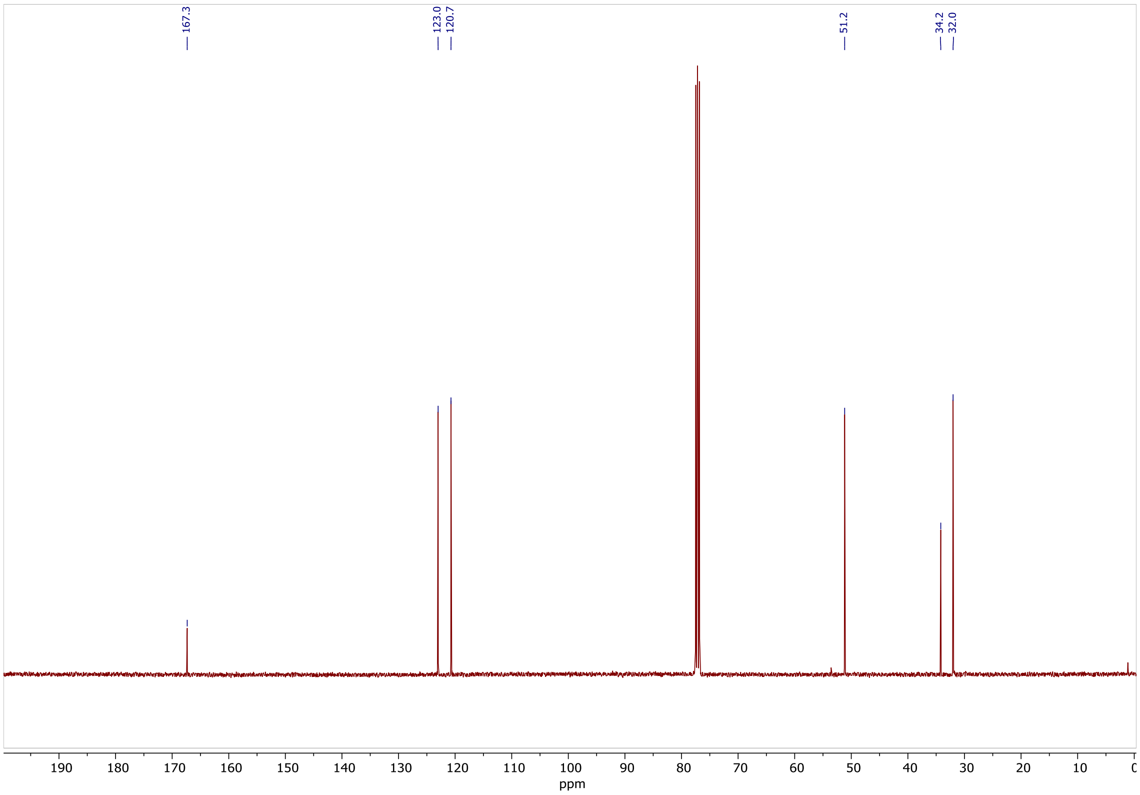

^{1}H NMR spectrum (400 MHz, CDCl$_3$, 298 K) of compound **5n**

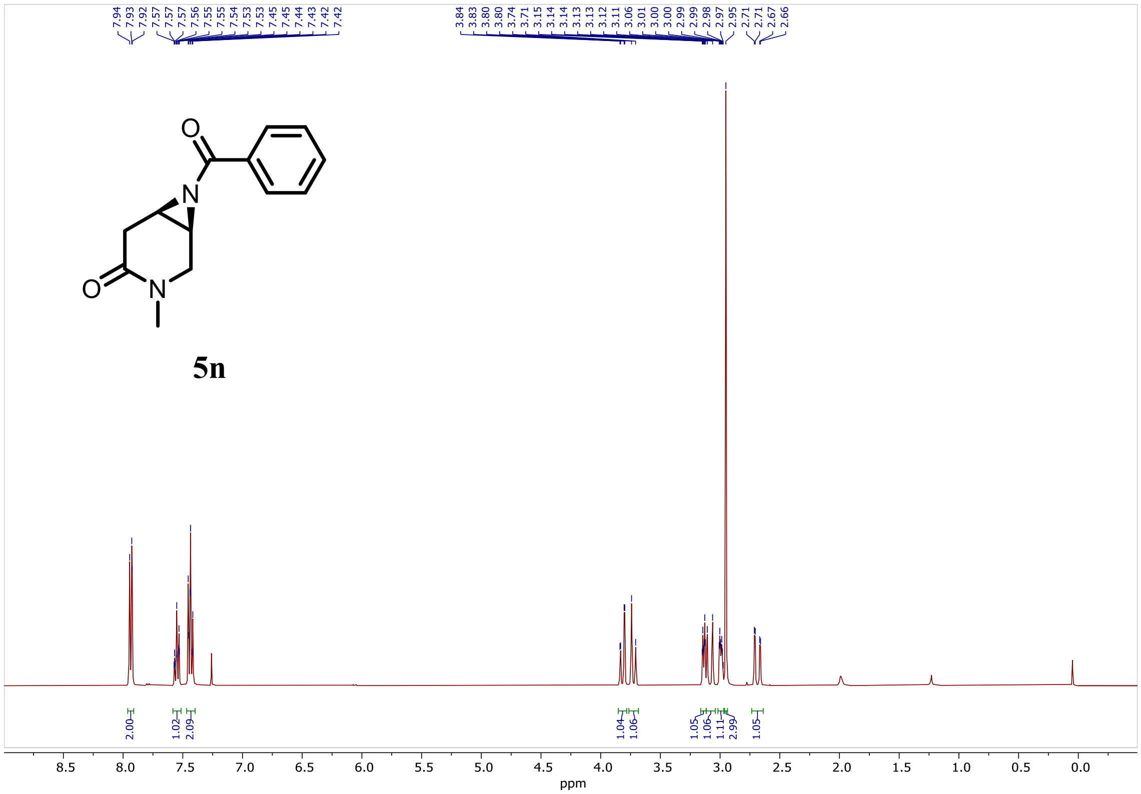

^{13}C{^{1}H} NMR spectrum (101 MHz, CDCl$_3$, 298 K) of compound **5n**

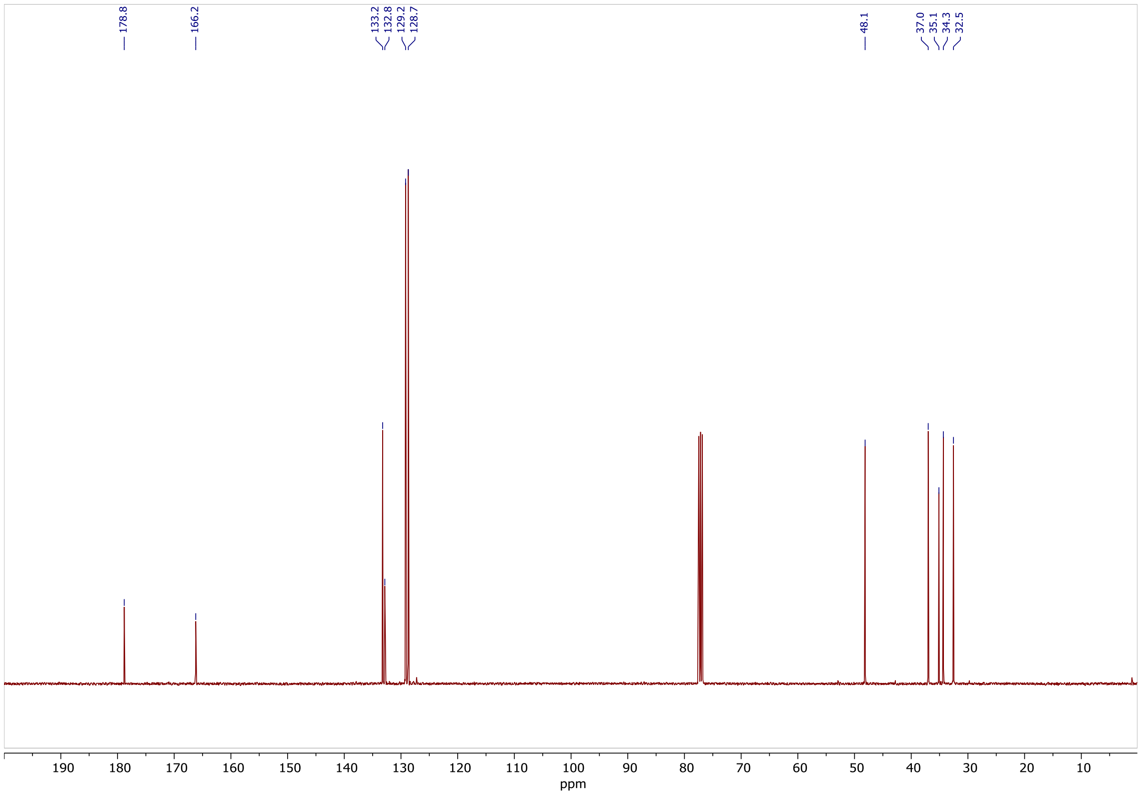

^{1}H NMR spectrum (400 MHz, DMSO-d_6, 343 K) of compound **S1b**

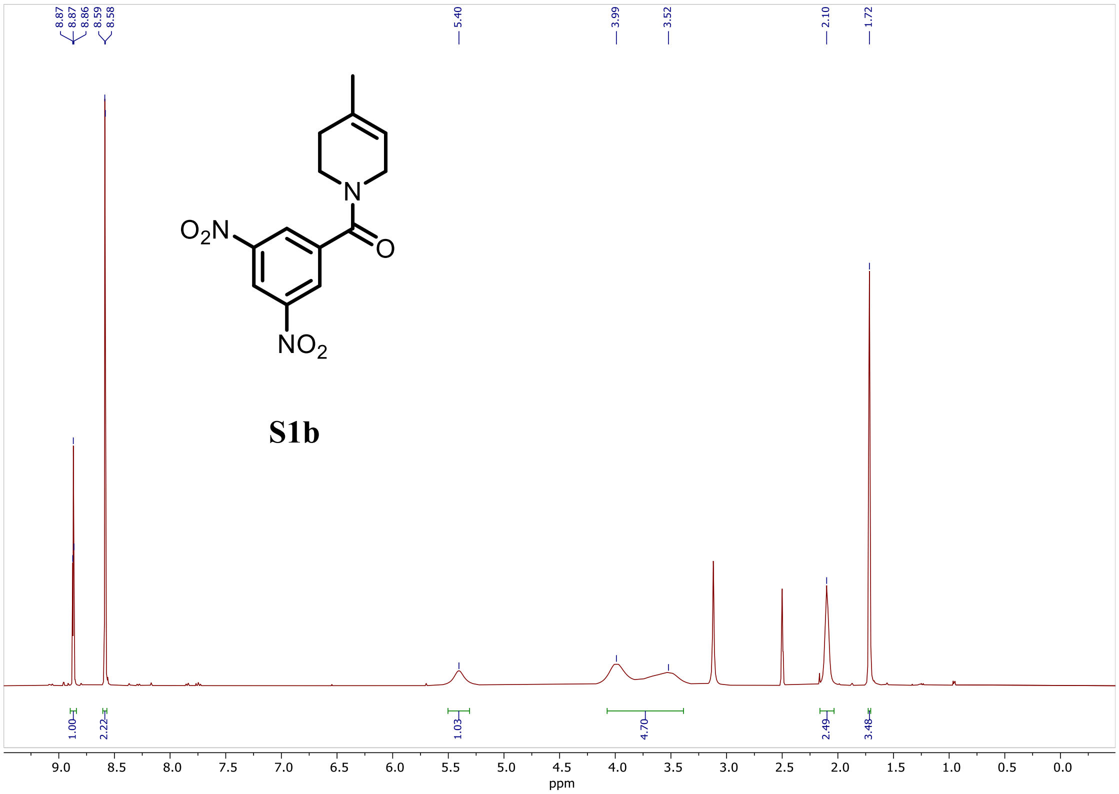

$^{13}C\{^{1}H\}$ NMR spectrum (101 MHz, DMSO-d_6, 343 K) of compound **S1b**

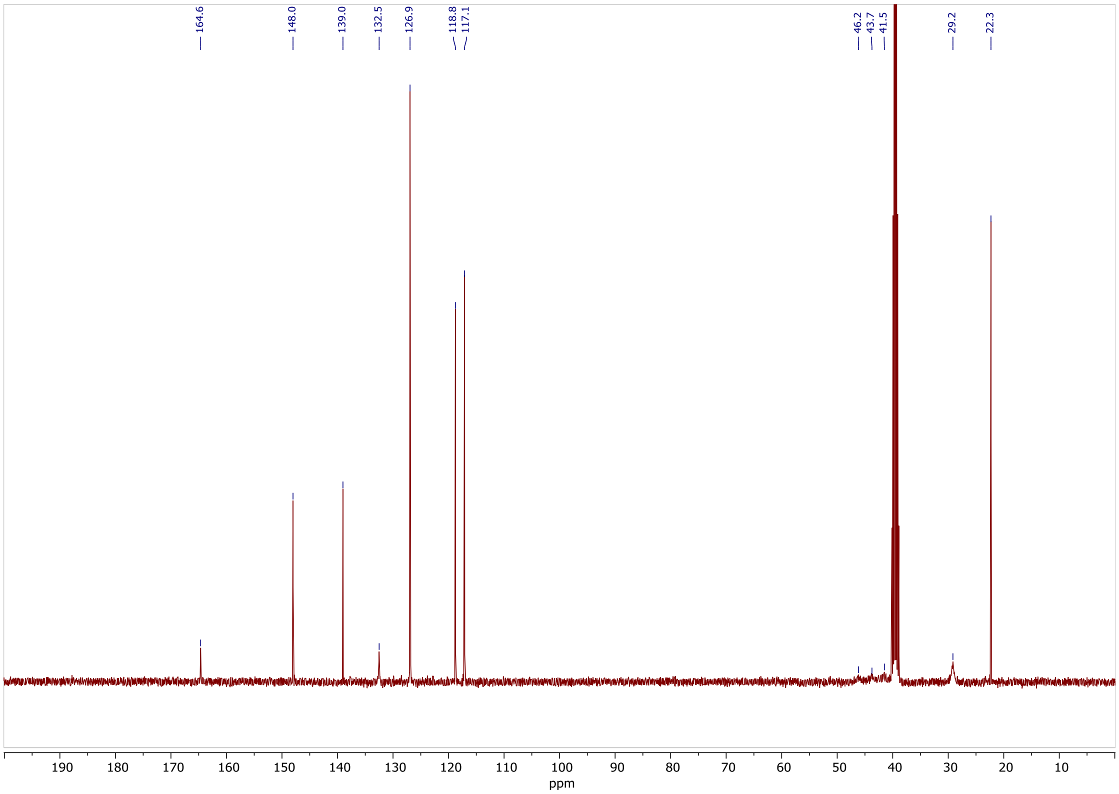

HSQC NMR spectrum (400 MHz, 101 MHz, DMSO-d_6, 343 K) of compound **S1b**

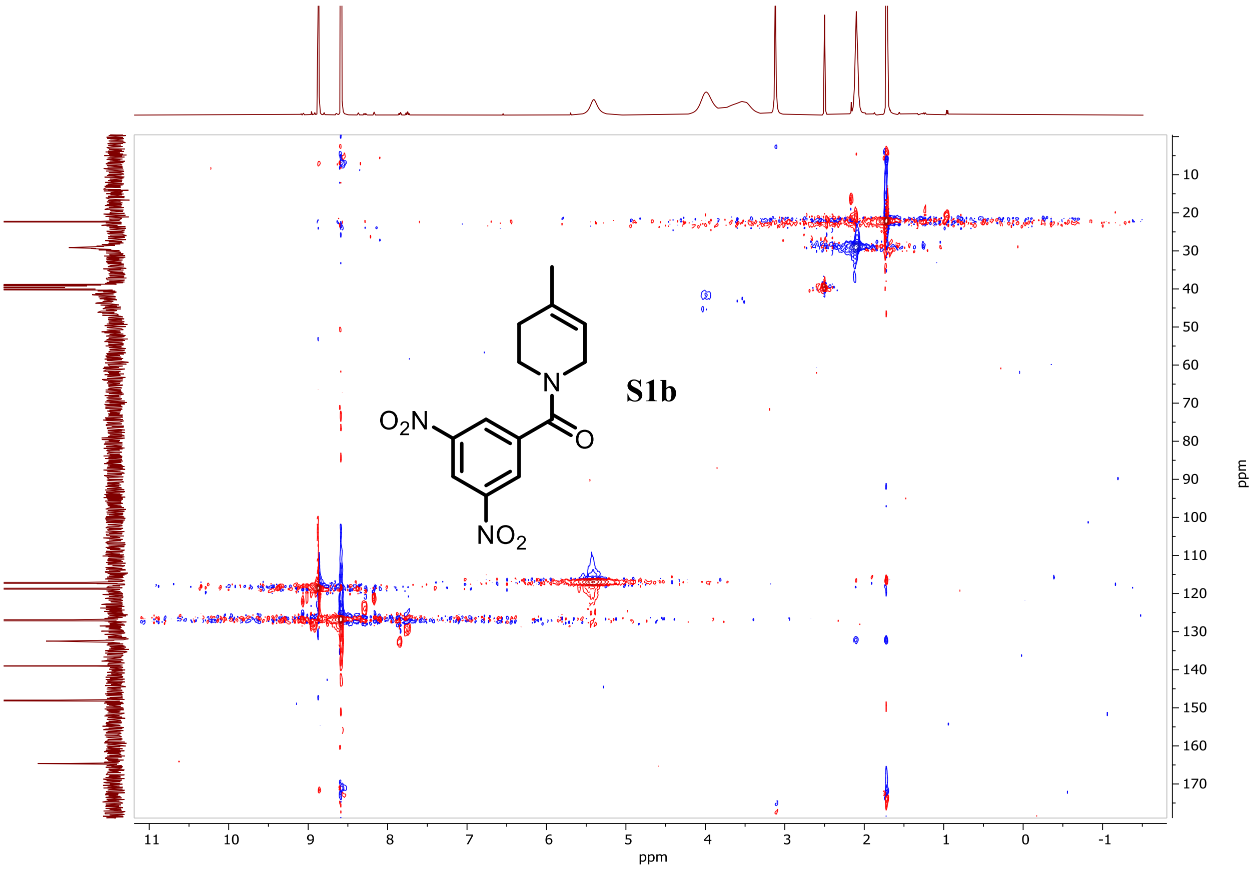

^{1}H NMR spectrum (400 MHz, DMSO-d_6, 343 K) of compound **S2g**

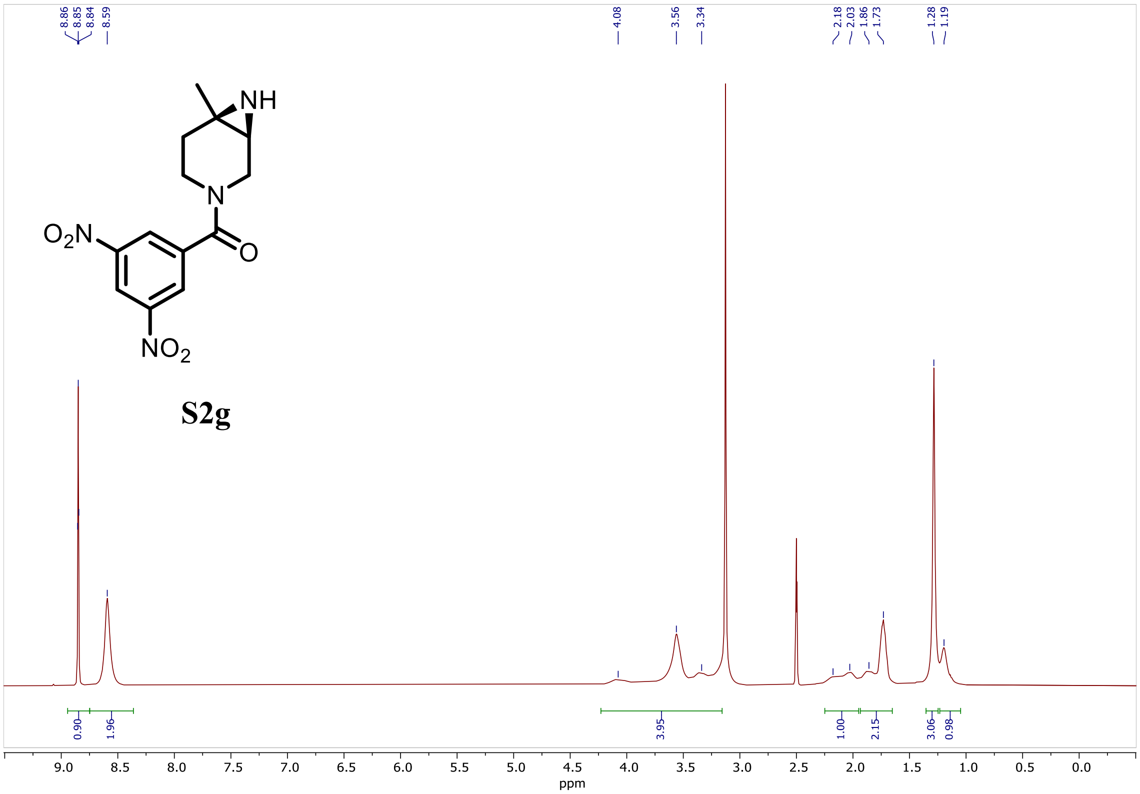

$^{13}C\{^{1}H\}$ NMR spectrum (101 MHz, DMSO-d_6, 343 K) of compound **S2g**

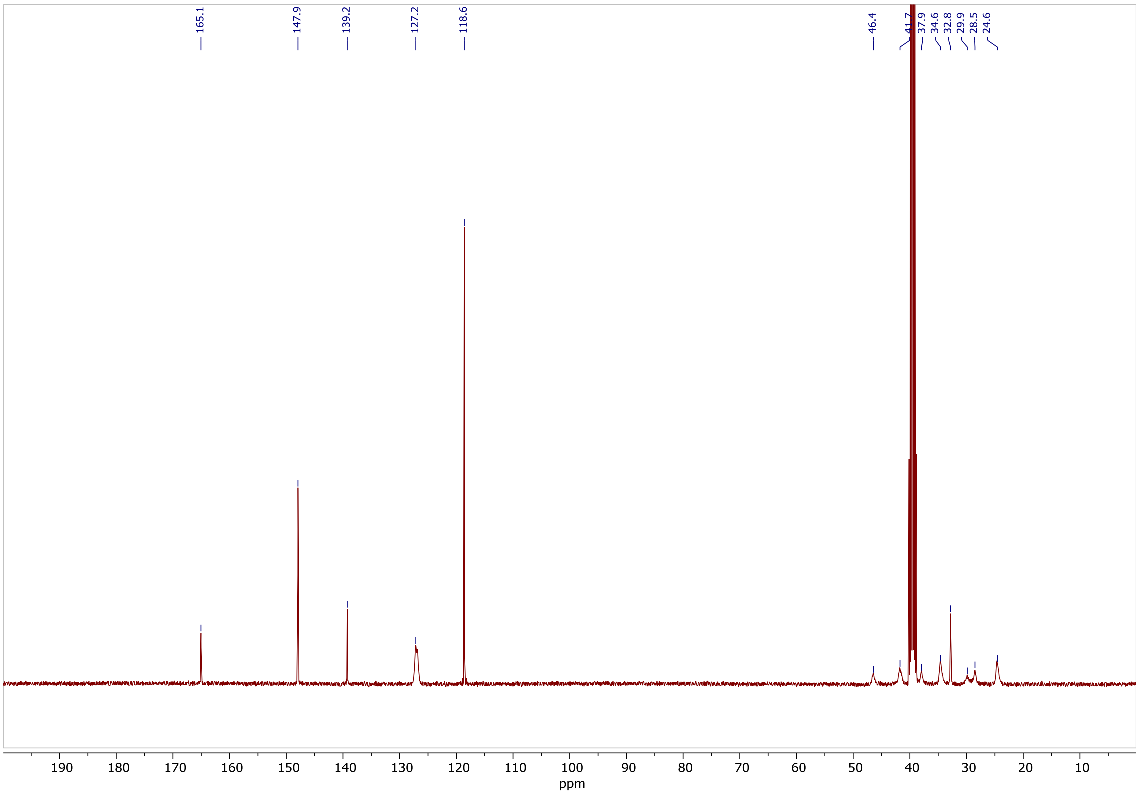

HSQC NMR spectrum (400 MHz, 101 MHz, DMSO-d6, 343 K) of compound **S2g**

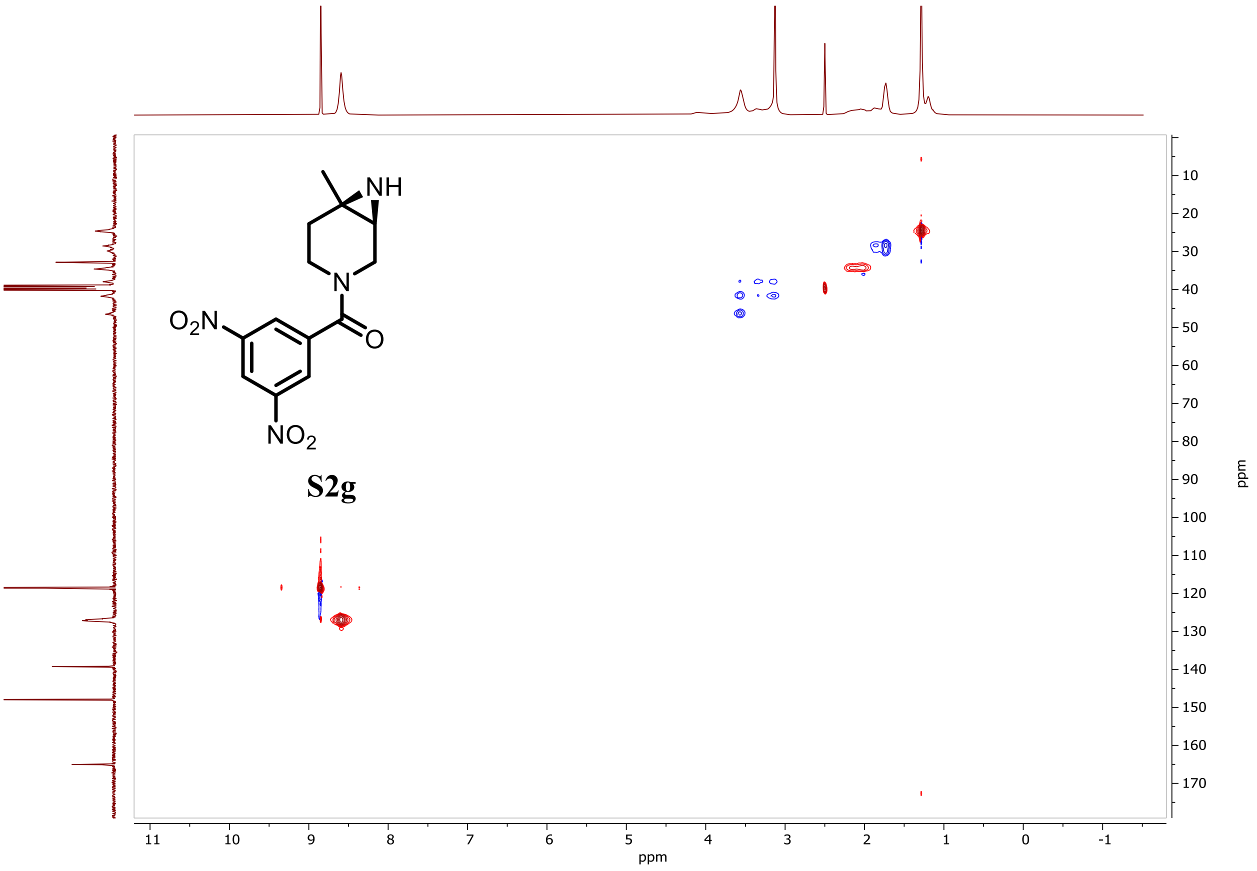

^{1}H NMR spectrum (400 MHz, DMSO-d6, 343 K) of compound **7**

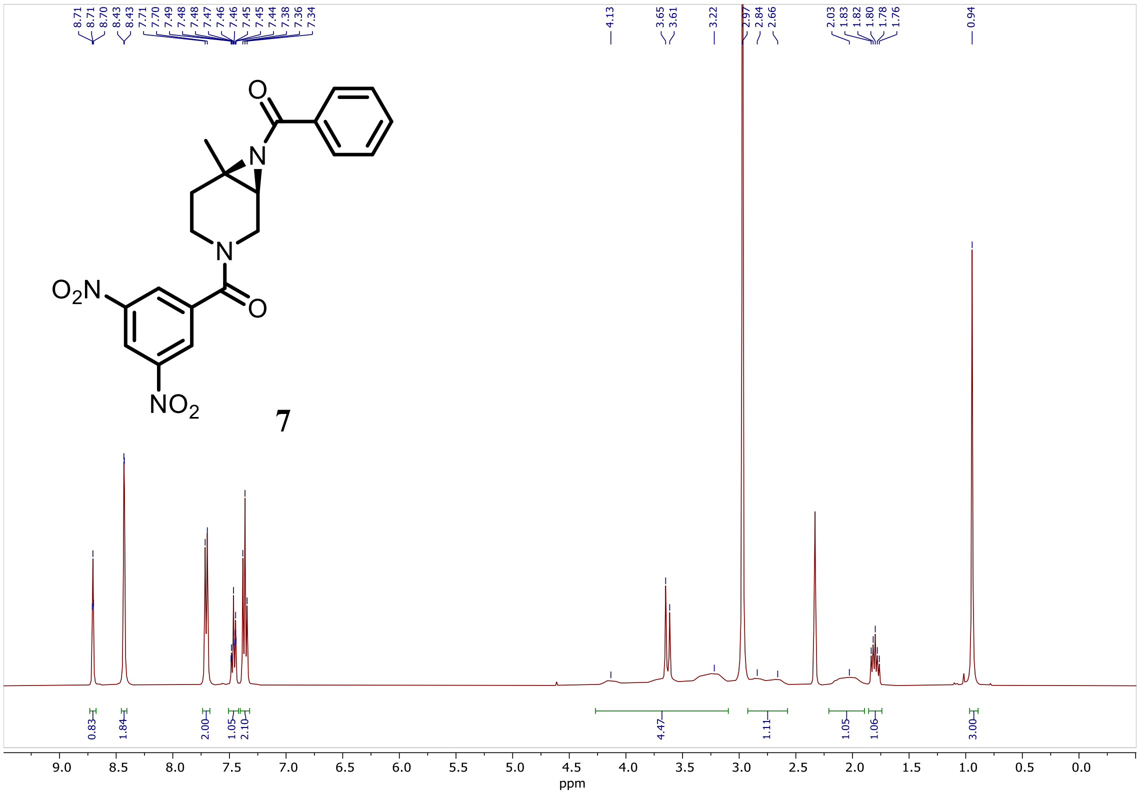

^{13}C{^{1}H} NMR spectrum (101 MHz, DMSO-d6, 343 K) of compound **7**

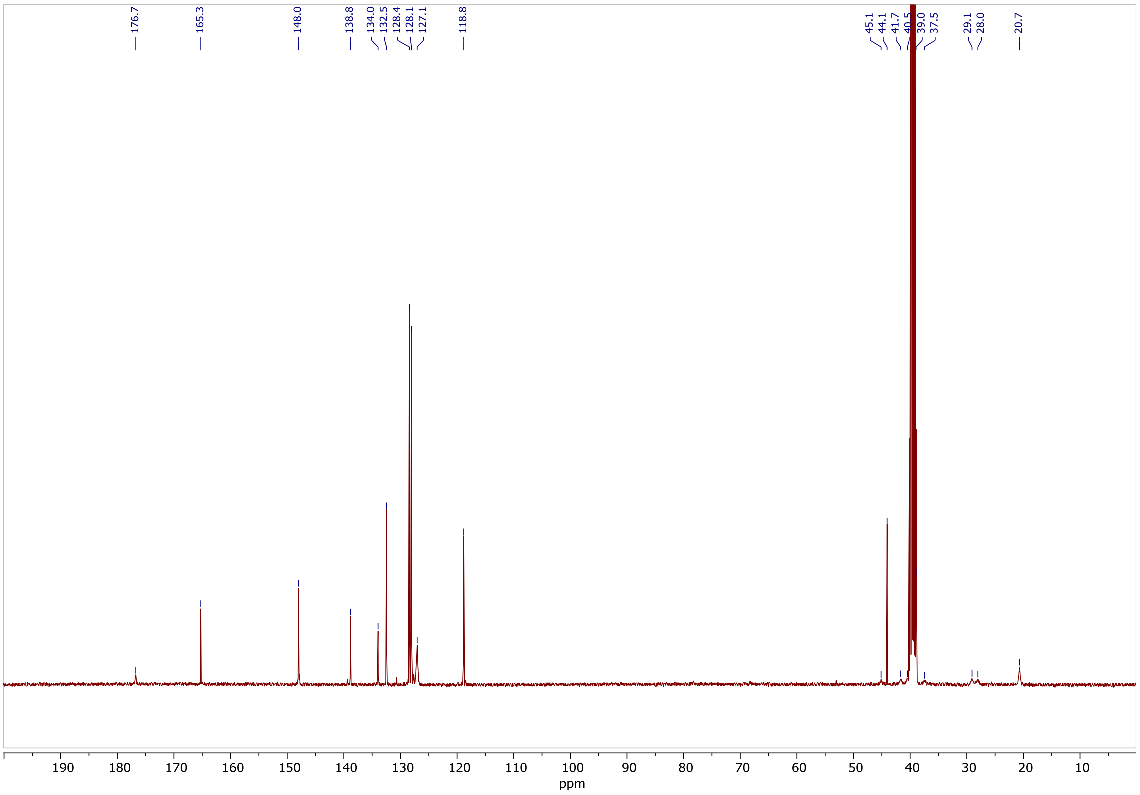

HSQC NMR spectrum (400 MHz, 101 MHz, DMSO-d_6, 343 K) of compound **7**

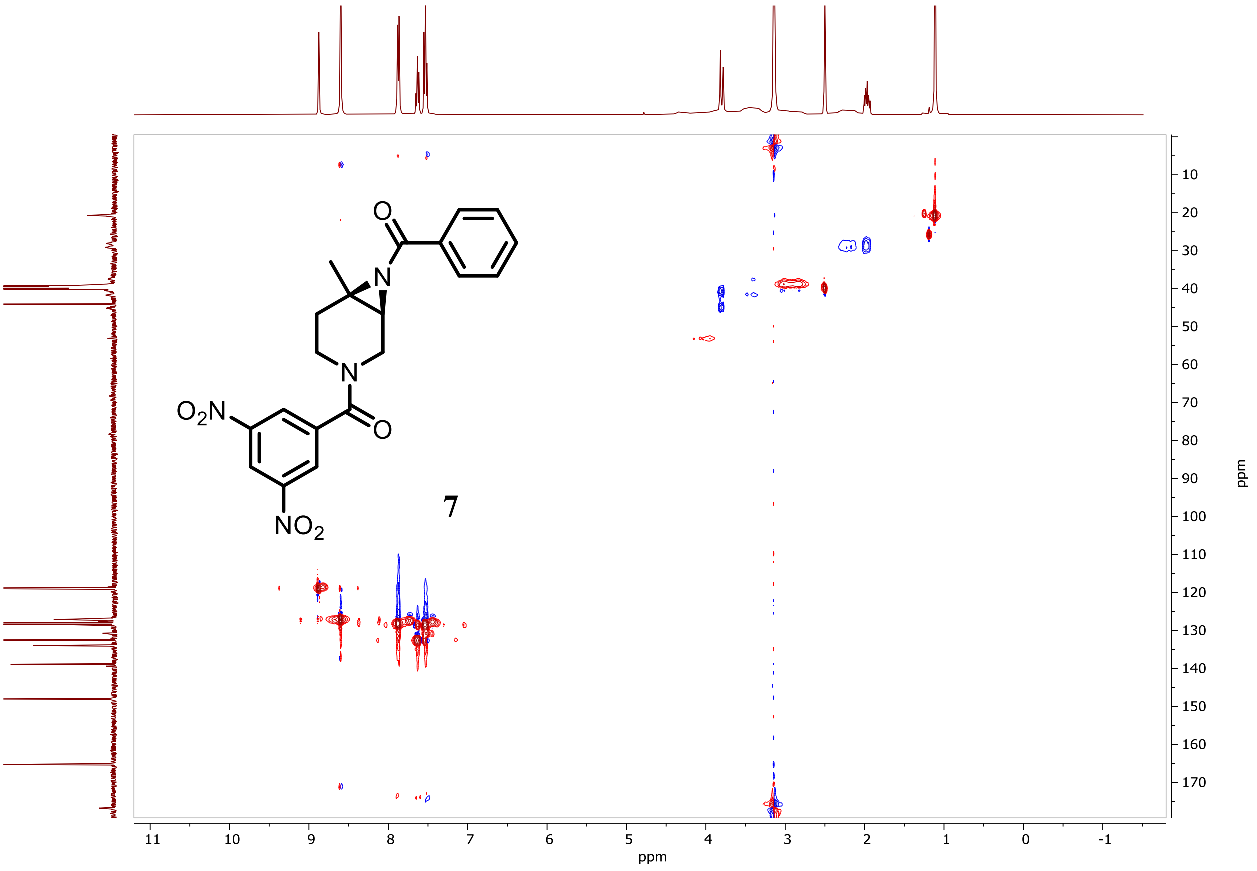

^{1}H NMR spectrum (400 MHz, DMSO-d_6, 343 K) of compound **2a**

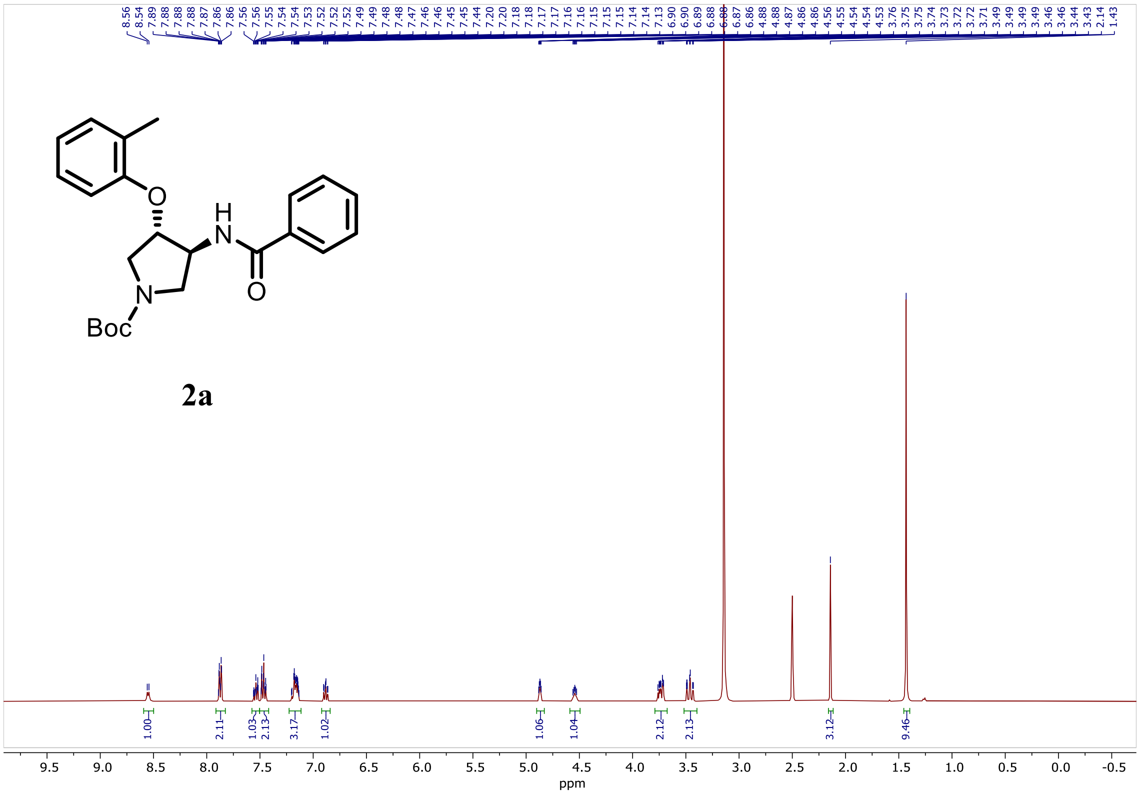

$^{13}C\{^{1}H\}$ NMR spectrum (101 MHz, DMSO-d_6, 343 K) of compound **2a**

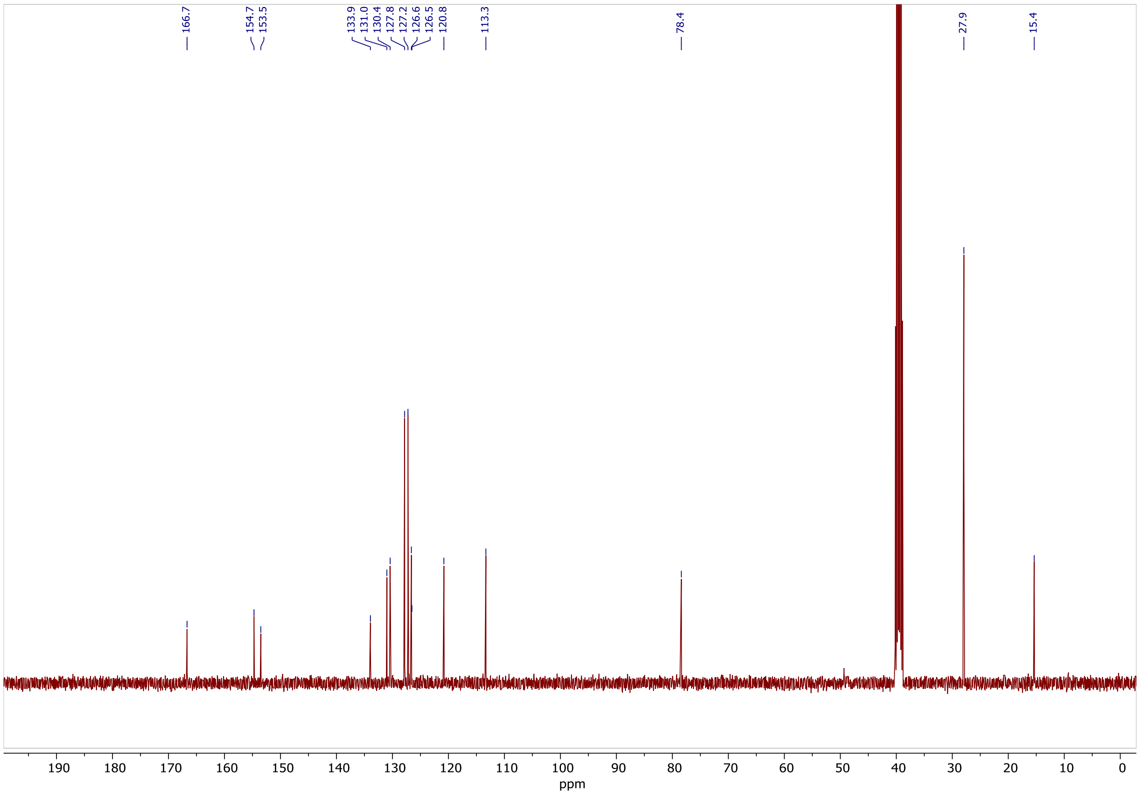

HSQC NMR spectrum (400 MHz, 101 MHz, DMSO-d_6, 343 K) of compound **2a**

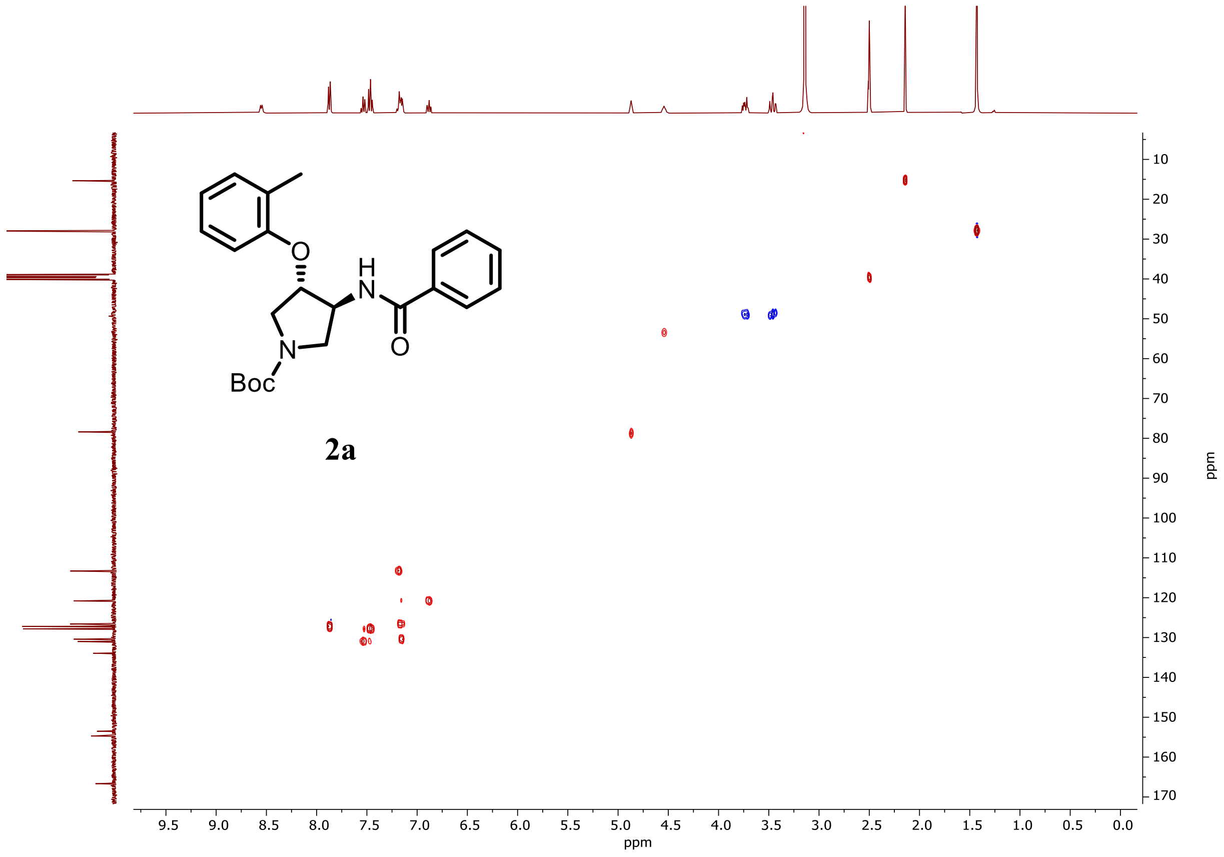

^{1}H NMR spectrum (400 MHz, $CDCl_3$, 298 K) of compound **f**

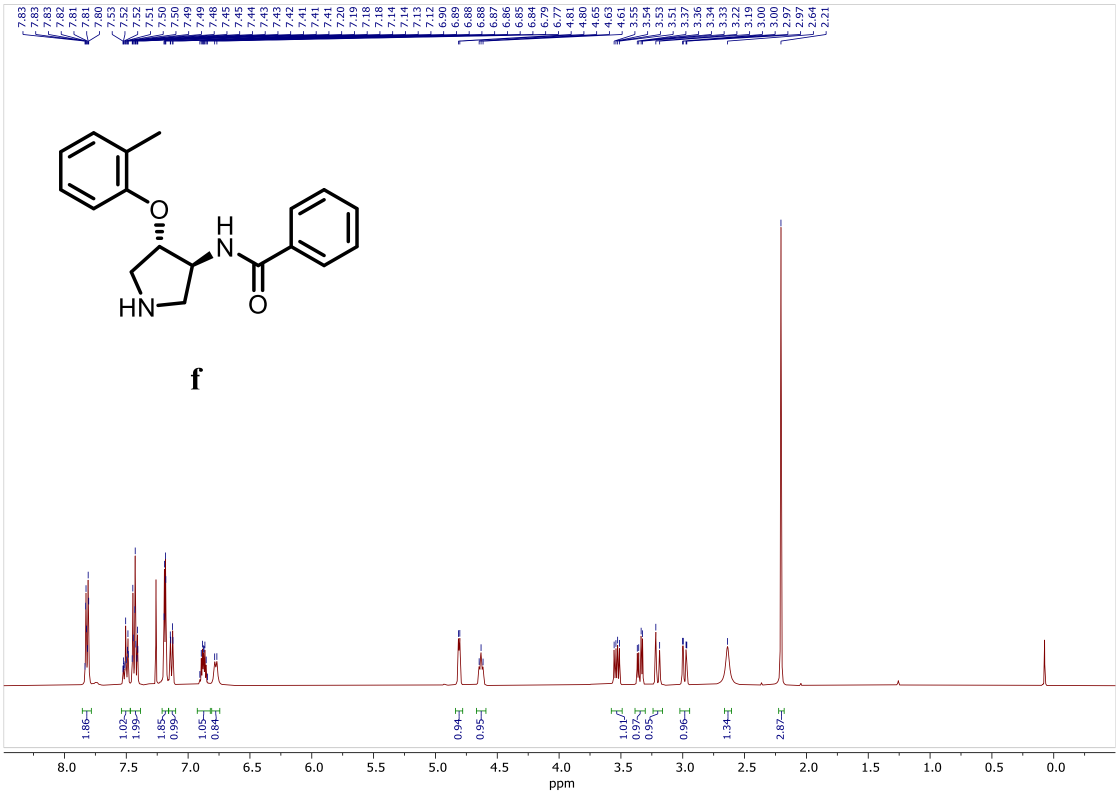

^{13}C{^{1}H} NMR spectrum (101 MHz, $CDCl_3$, 298 K) of compound **f**

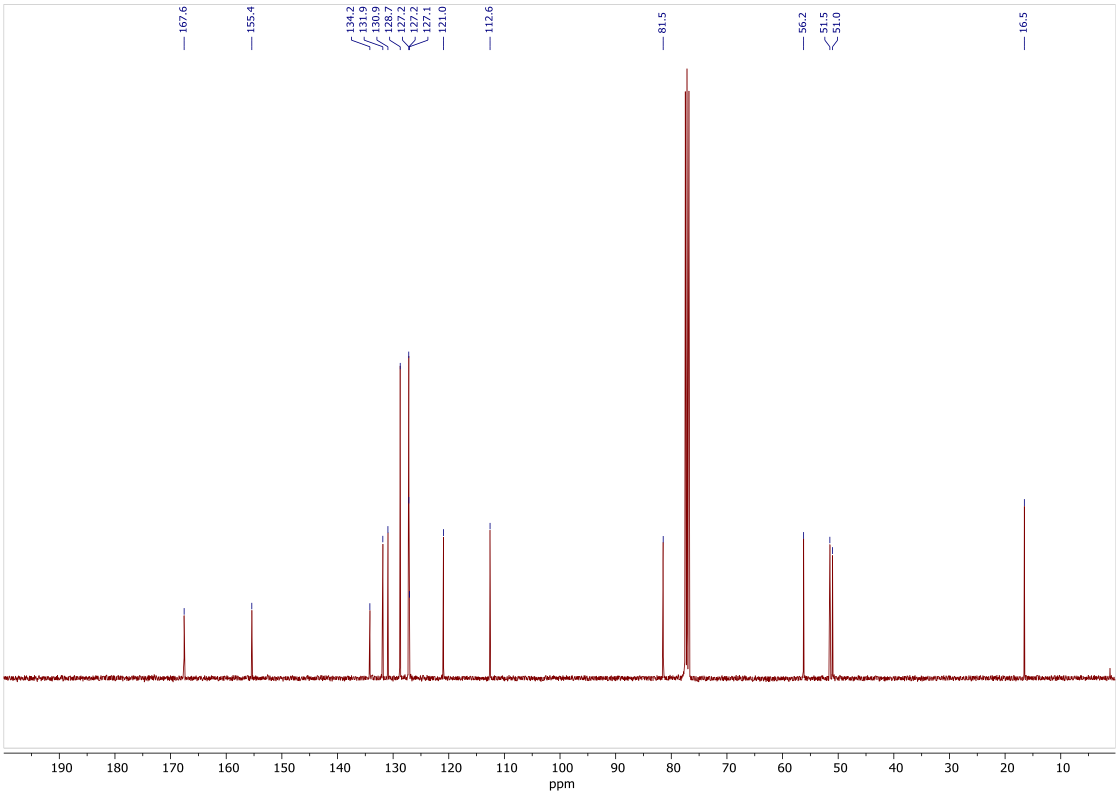

^{1}H NMR spectrum (400 MHz, DMSO-d_6, 343 K) of compound **2b**

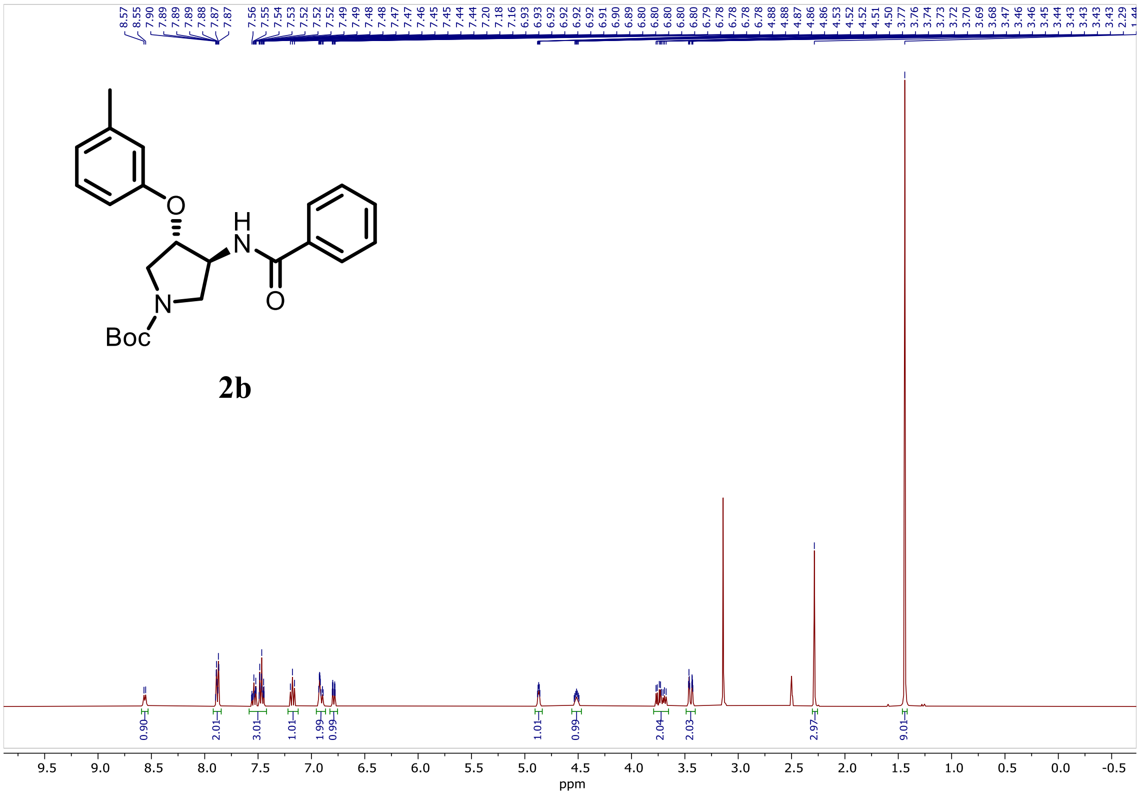

^{13}C{^{1}H} NMR spectrum (101 MHz, DMSO-d_6, 343 K) of compound **2b**

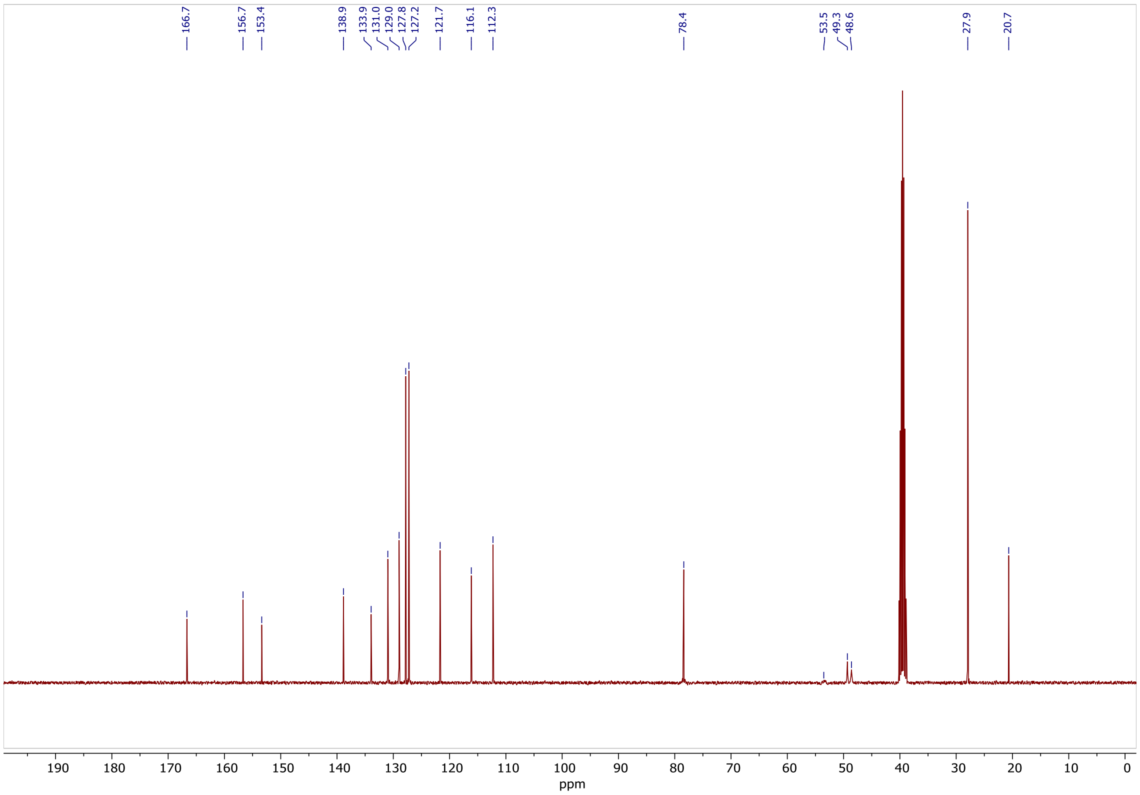

HSQC NMR spectrum (400 MHz, 101 MHz, DMSO-d_6, 343 K) of compound **2b**

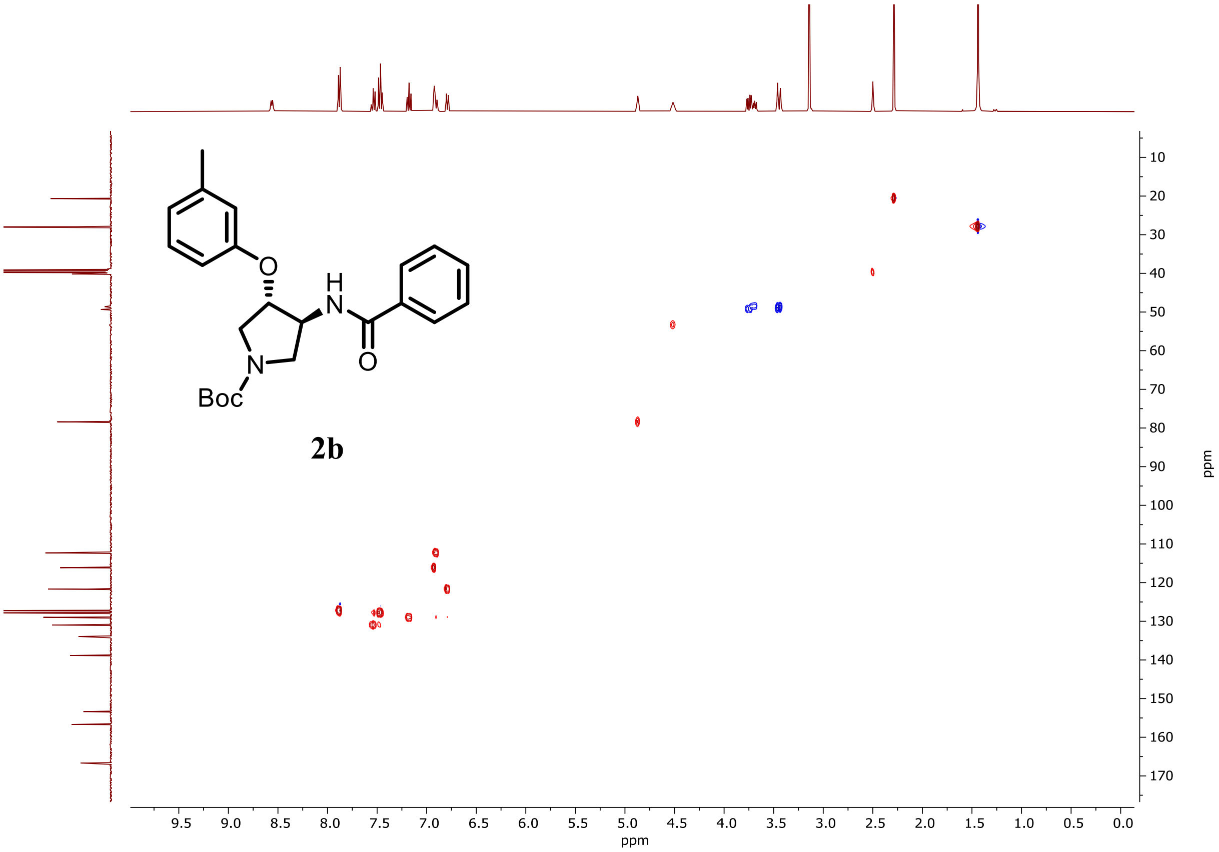

^{1}H NMR spectrum (400 MHz, DMSO-d_6, 343 K) of compound **2c**

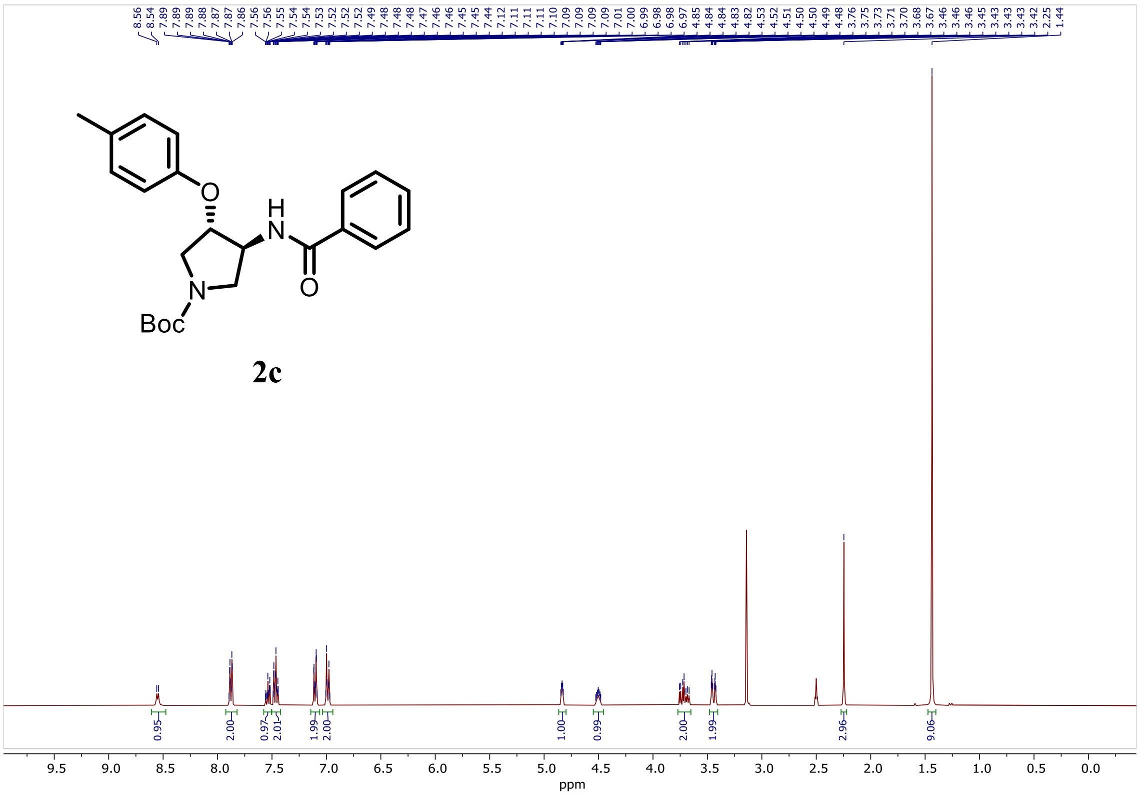

^{13}C{^{1}H} NMR spectrum (101 MHz, DMSO-d_6, 343 K) of compound **2c**

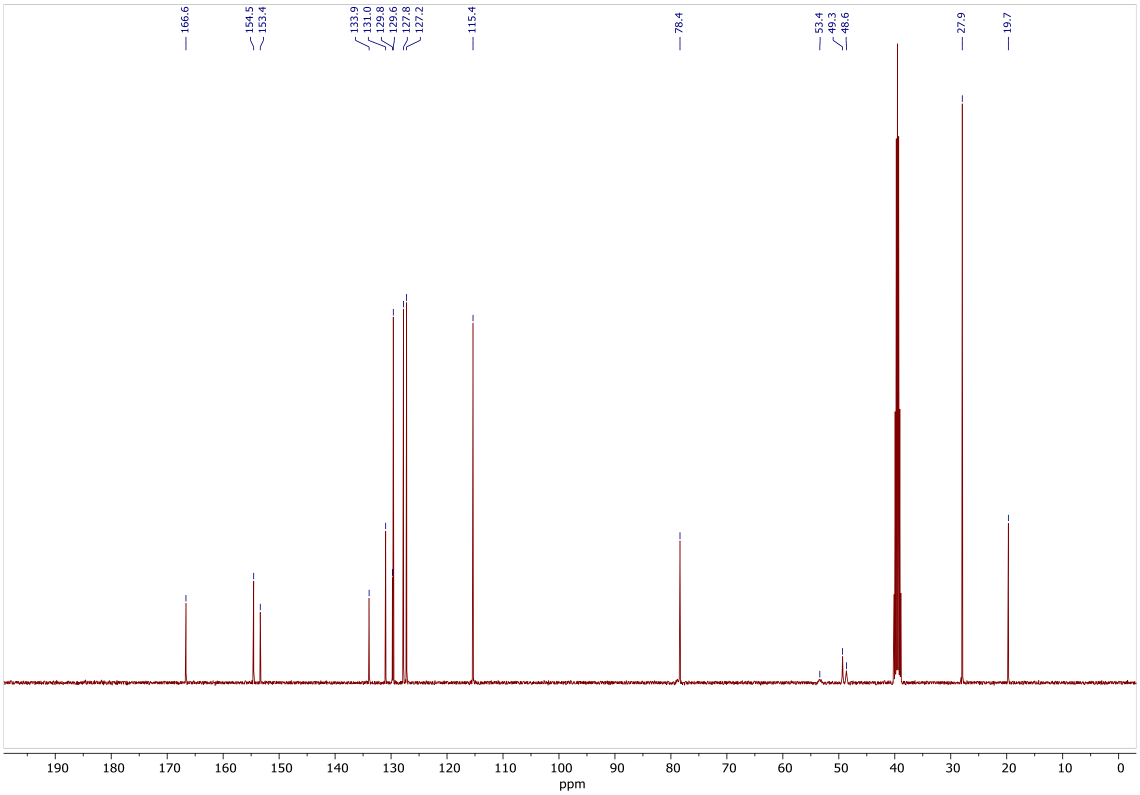

HSQC NMR spectrum (400 MHz, 101 MHz, DMSO-d_6, 343 K) of compound **2c**

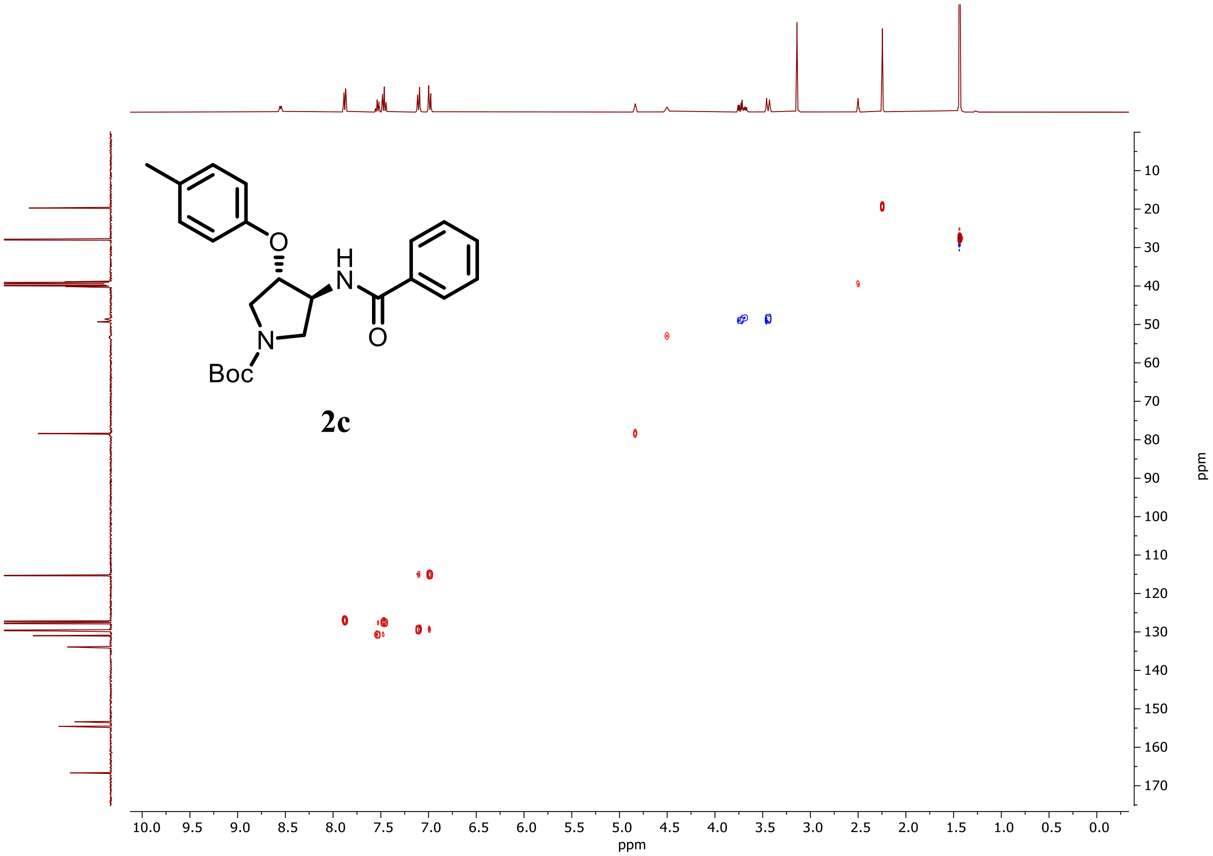

^{1}H NMR spectrum (400 MHz, DMSO-d_6, 343 K) of compound **2d**

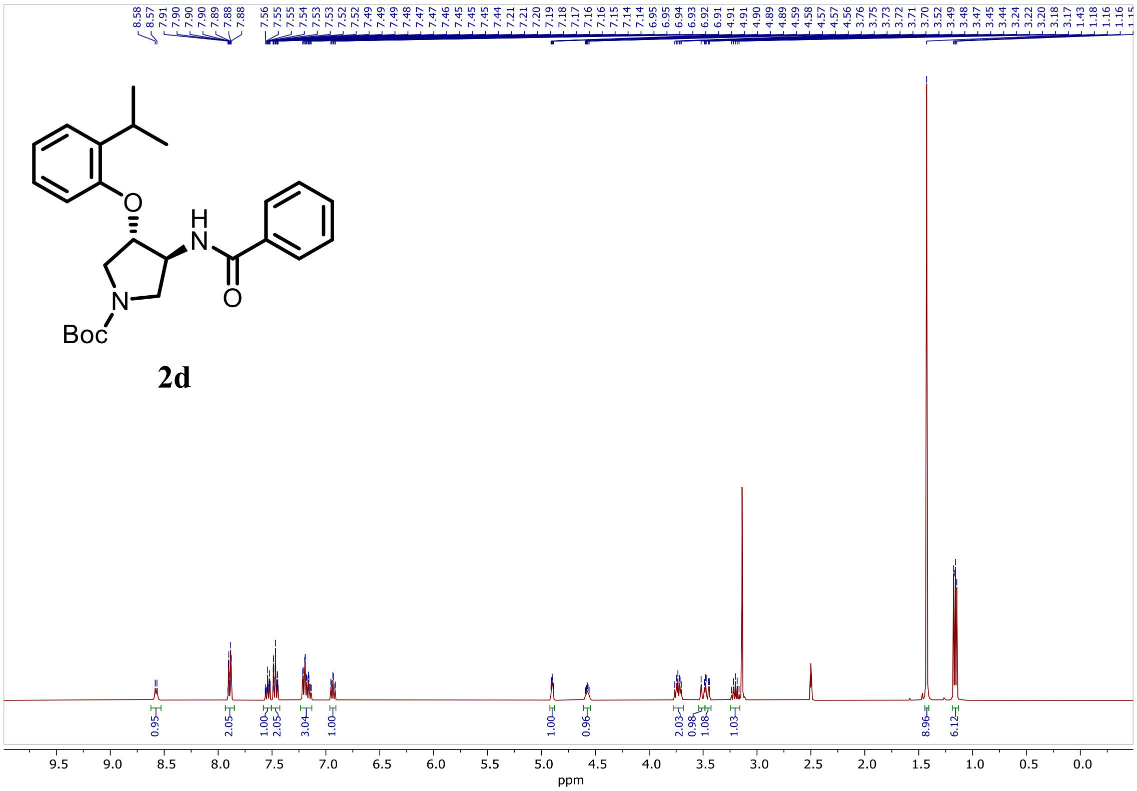

^{13}C{^{1}H} NMR spectrum (101 MHz, DMSO-d_6, 343 K) of compound **2d**

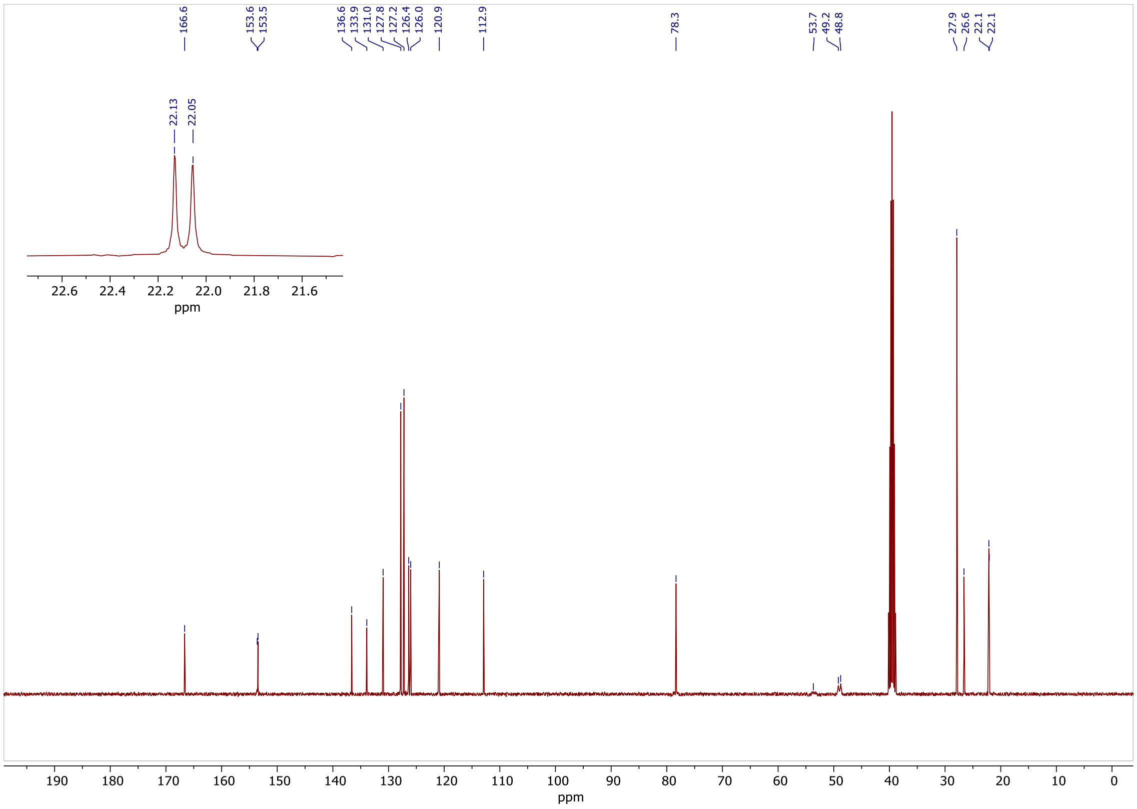

HSQC NMR spectrum (400 MHz, 101 MHz, DMSO-d_6, 343 K) of compound **2d**

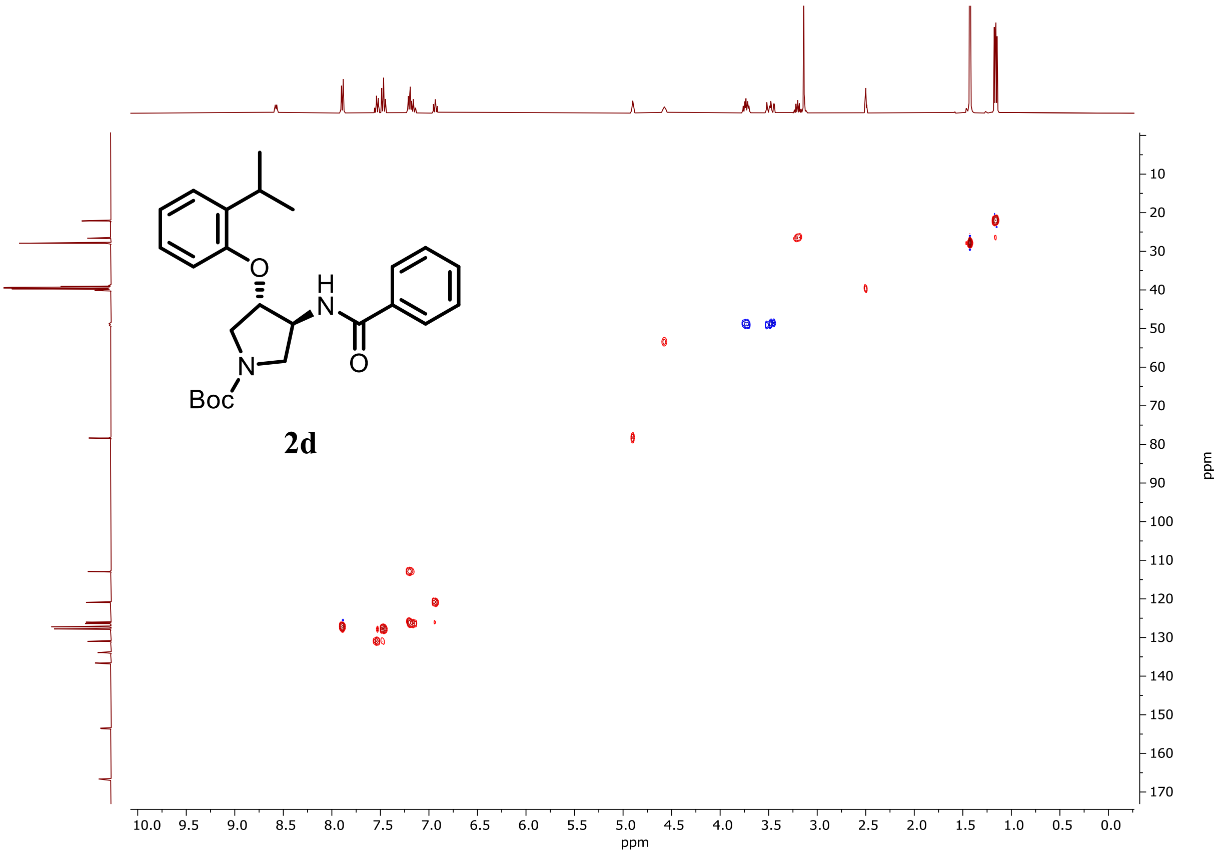

^{1}H NMR spectrum (400 MHz, DMSO-d_6, 343 K) of compound **2e**

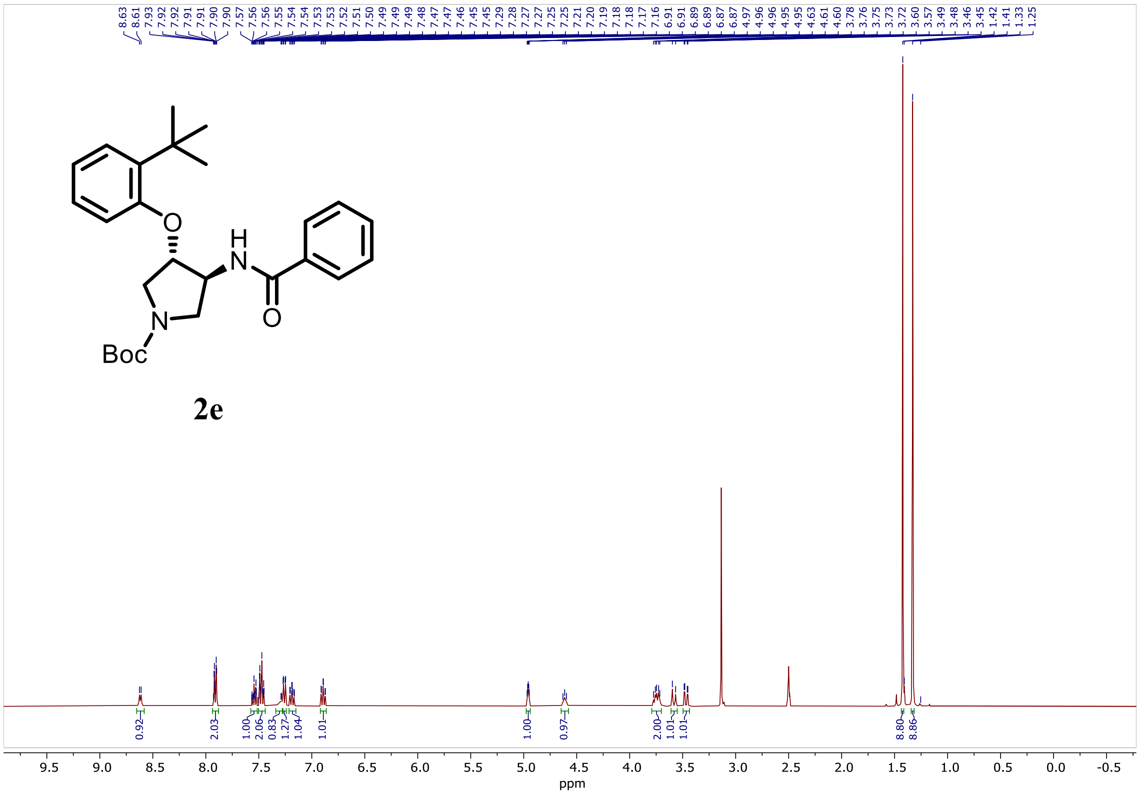

^{13}C{^{1}H} NMR spectrum (101 MHz, DMSO-d_6, 343 K) of compound **2e**

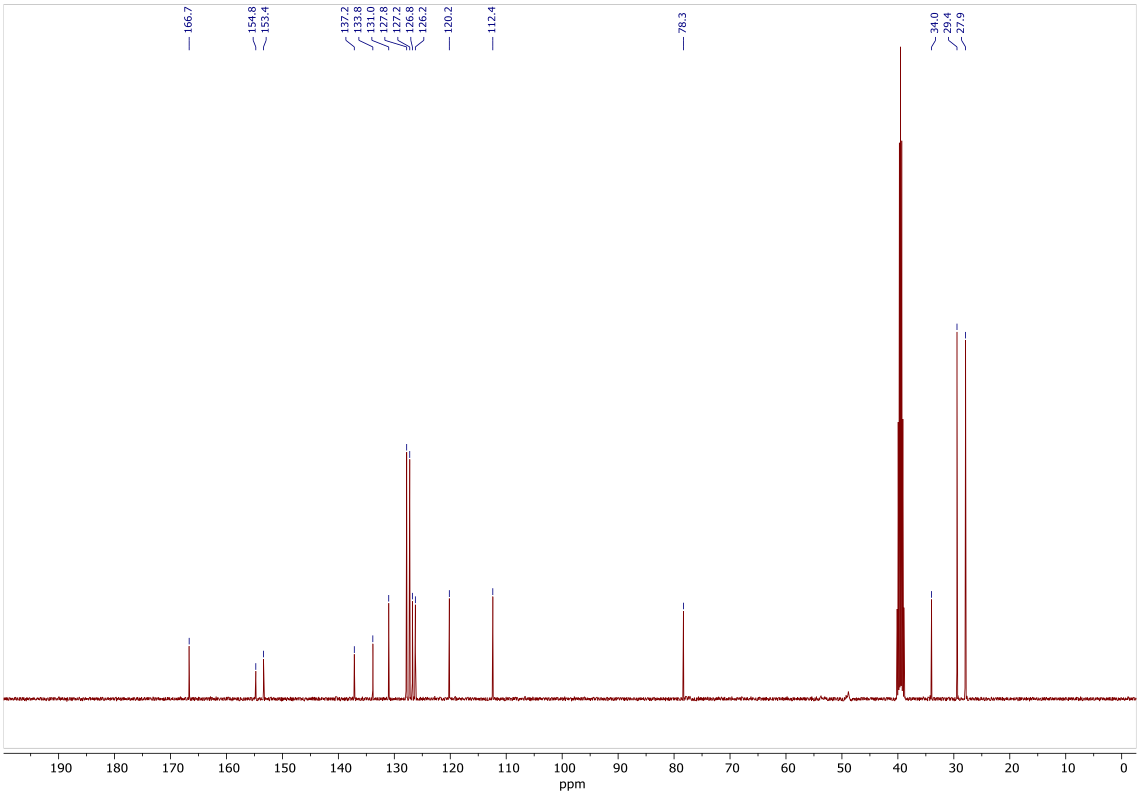

HSQC NMR spectrum (400 MHz, 101 MHz, DMSO-d_6, 343 K) of compound **2e**

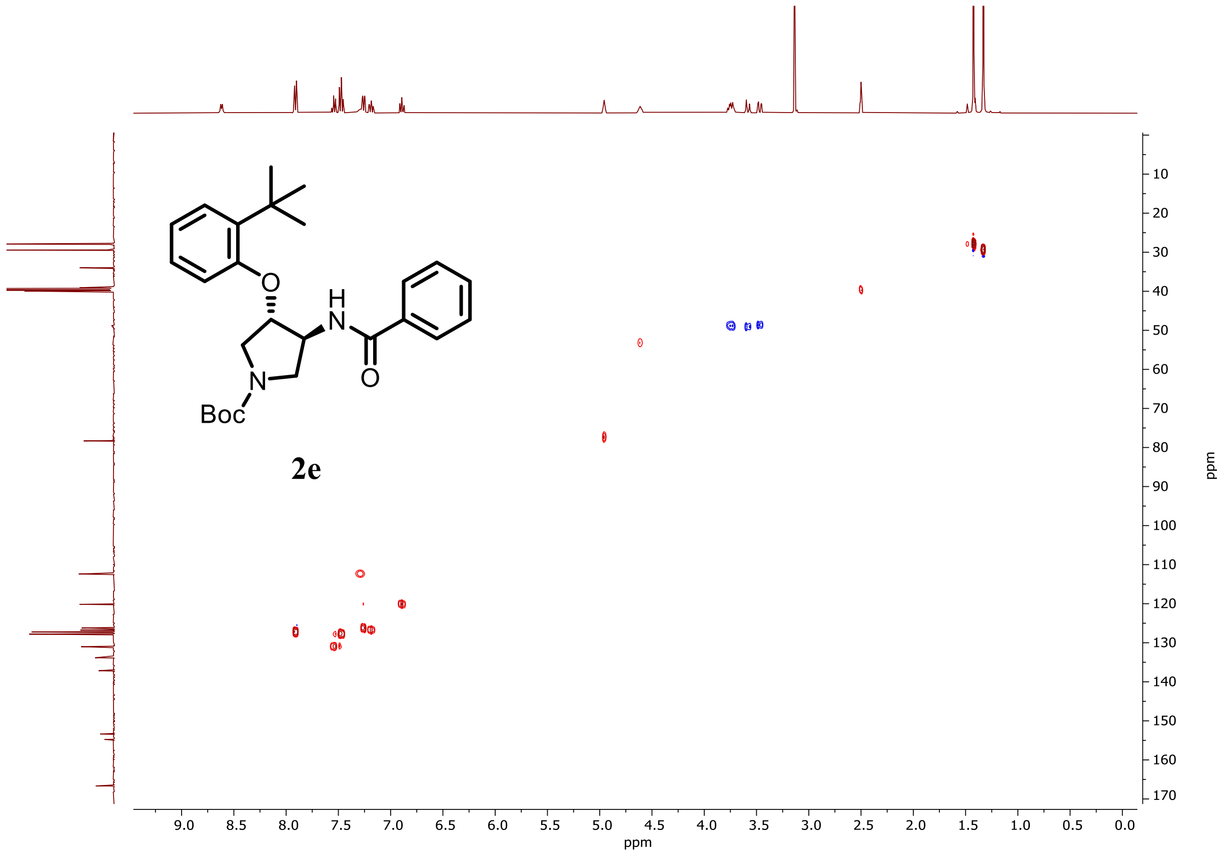

^{1}H NMR spectrum (400 MHz, DMSO-d_6, 343 K) of compound **2f**

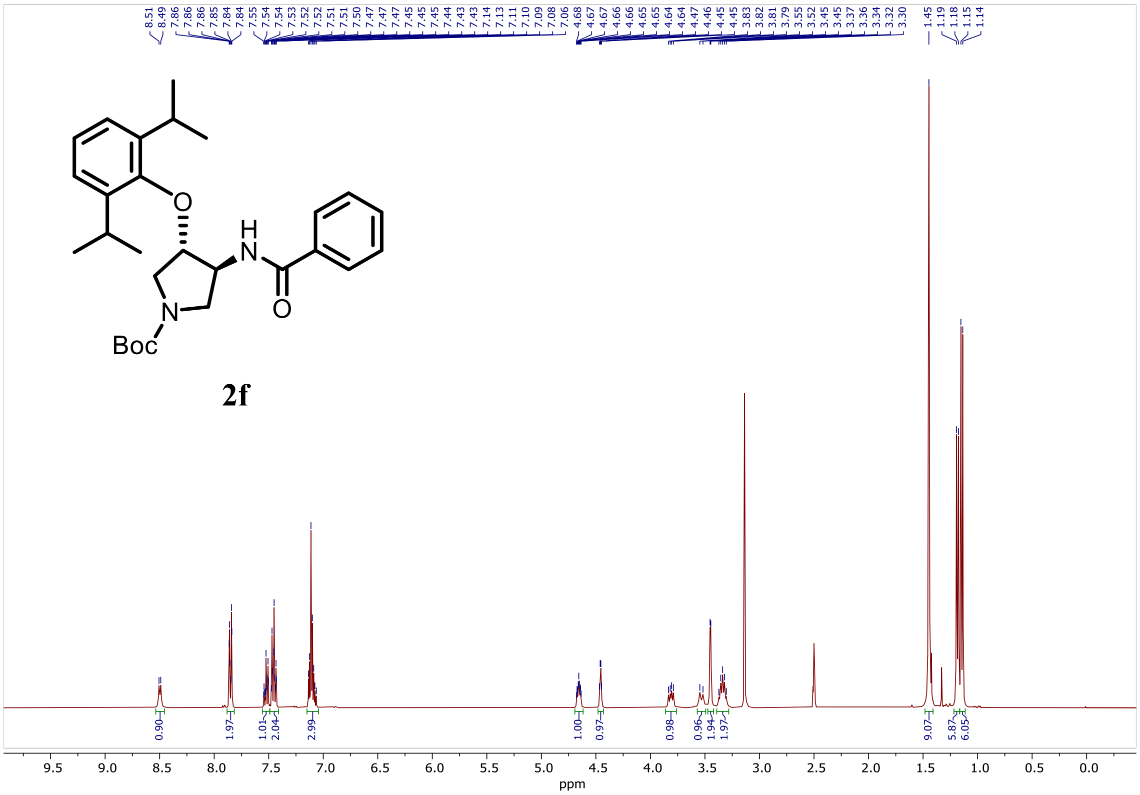

^{13}C{^{1}H} NMR spectrum (101 MHz, DMSO-d_6, 343 K) of compound **2f**

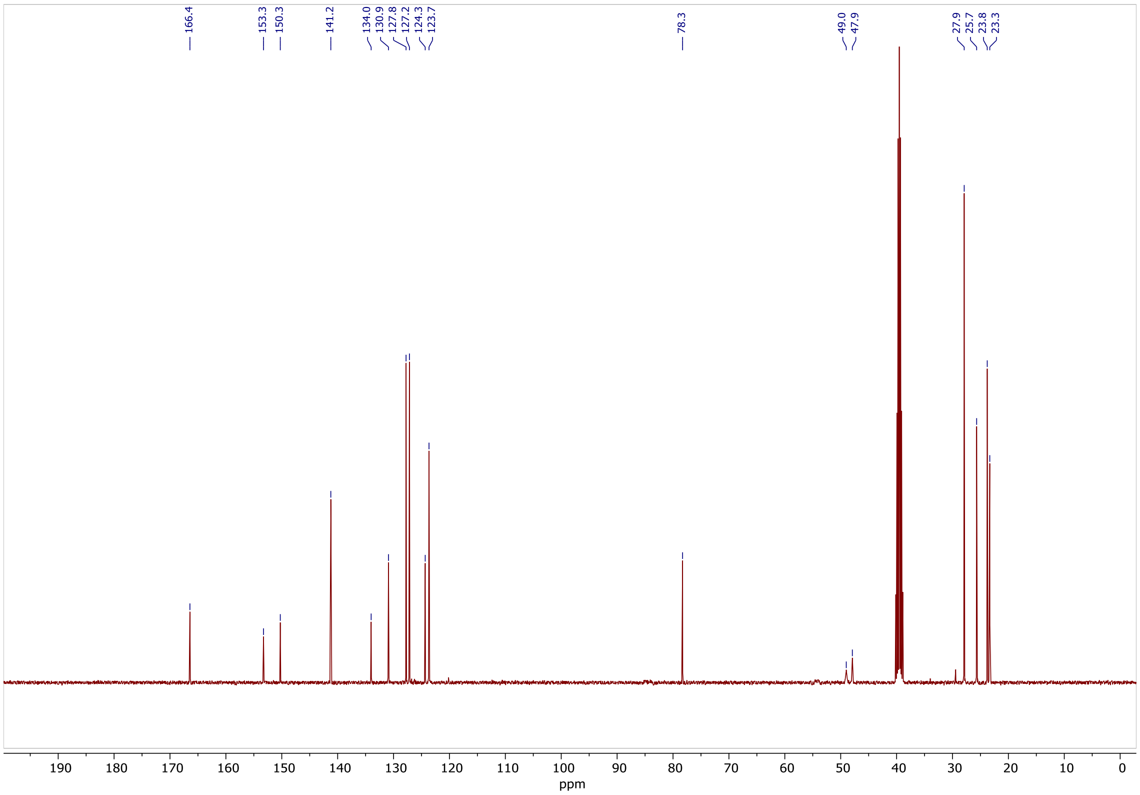

HSQC NMR spectrum (400 MHz, 101 MHz, DMSO-d_6, 343 K) of compound **2f**

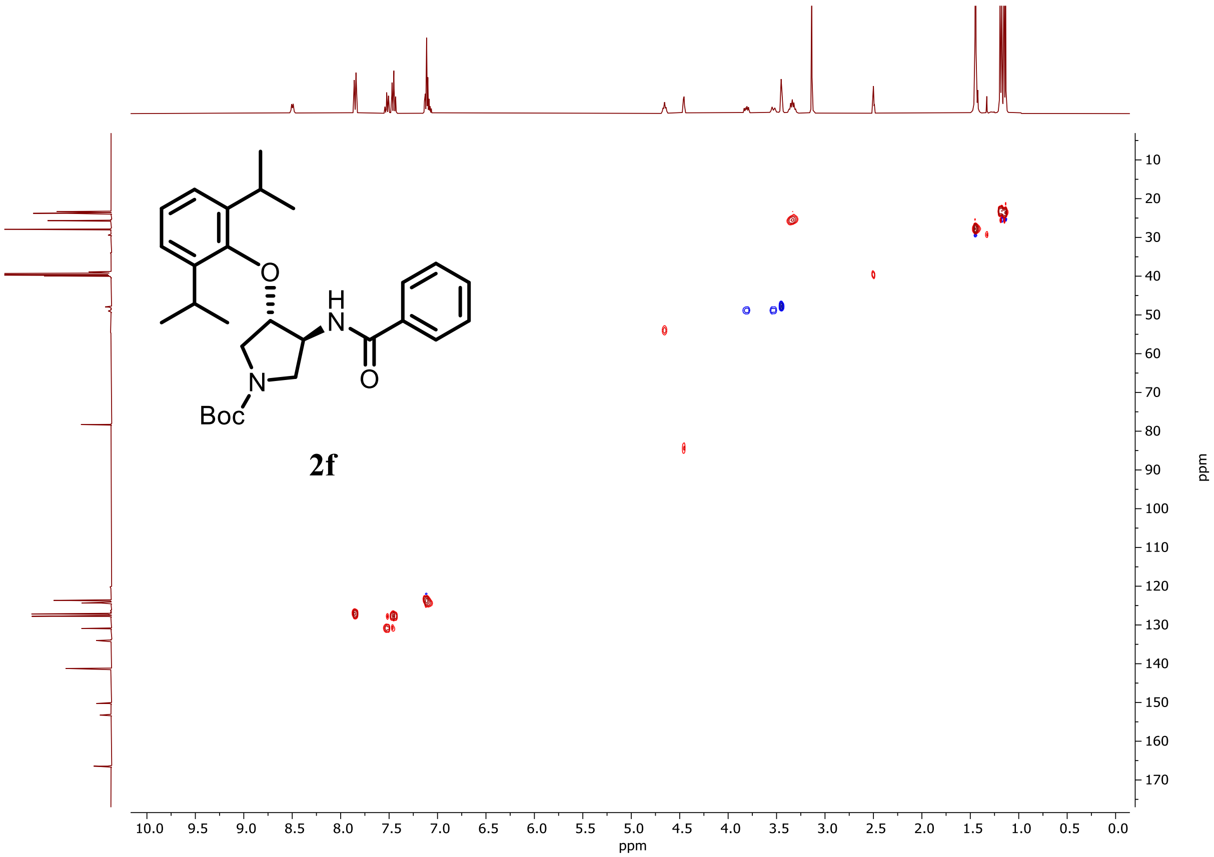

^{1}H NMR spectrum (400 MHz, DMSO-d_6, 343 K) of compound **2g**

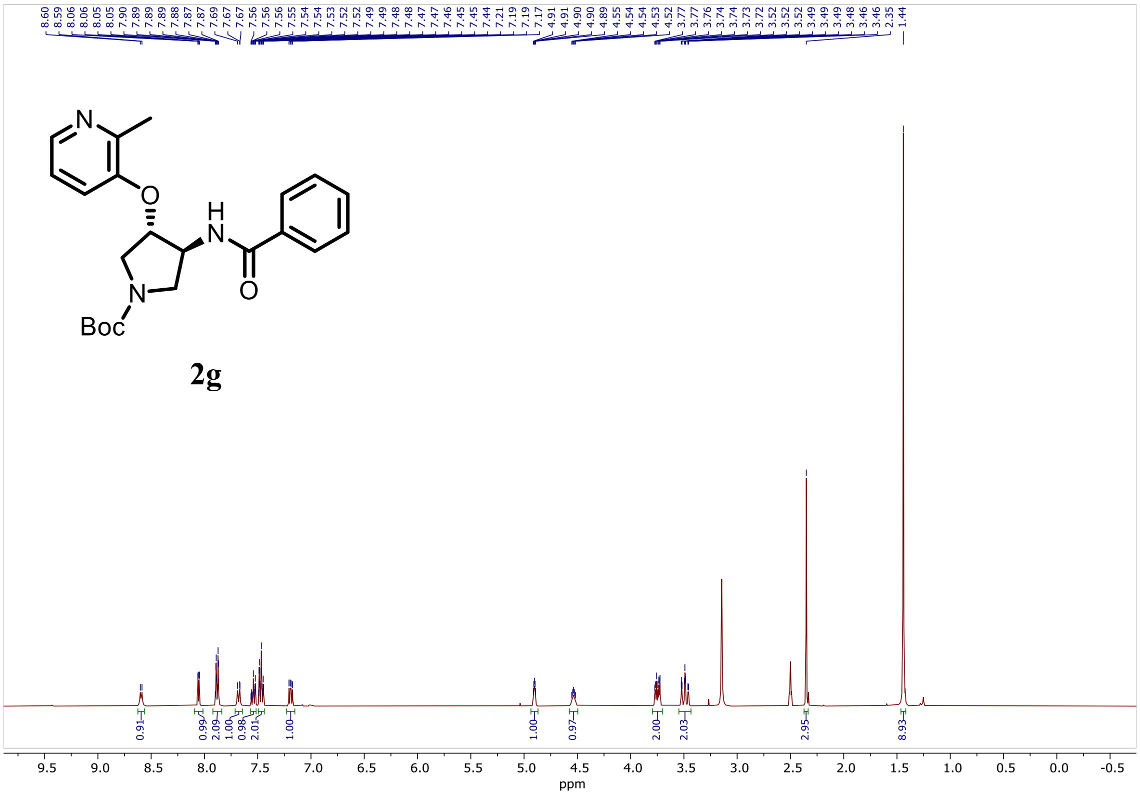

^{13}C{^{1}H} NMR spectrum (101 MHz, DMSO-d_6, 343 K) of compound **2g**

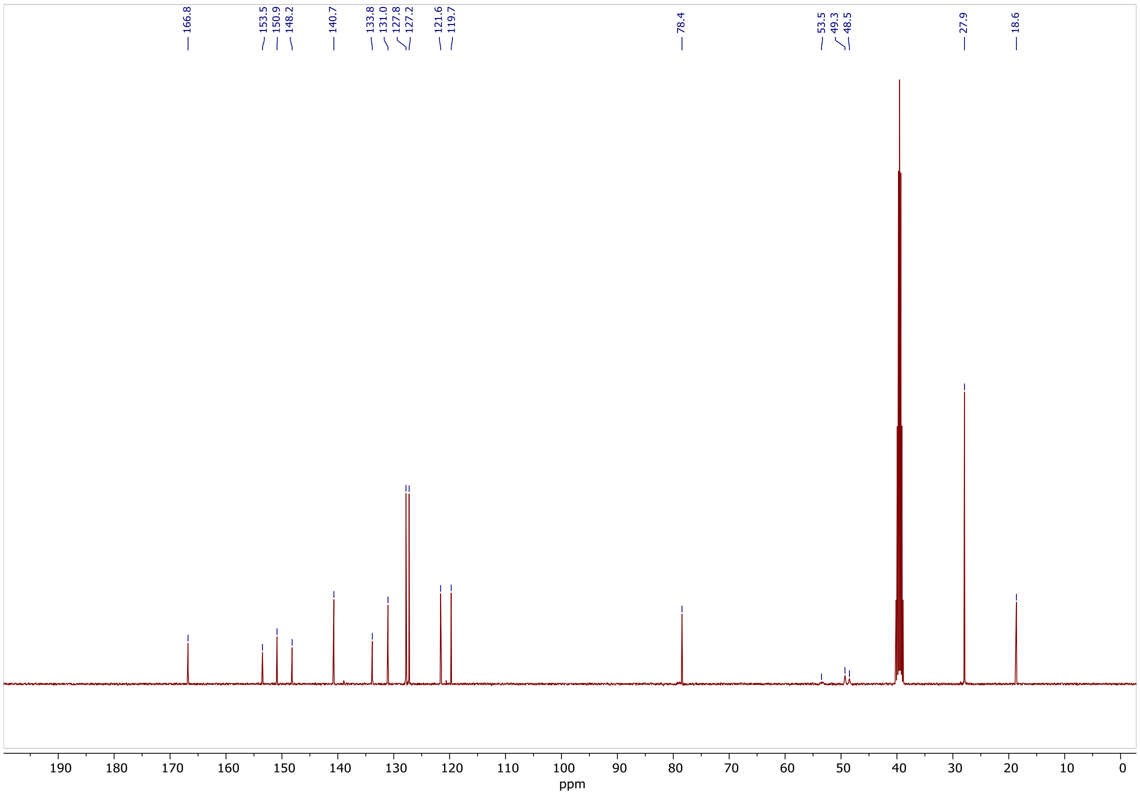

HSQC NMR spectrum (400 MHz, 101 MHz, DMSO-d_6, 343 K) of compound **2g**

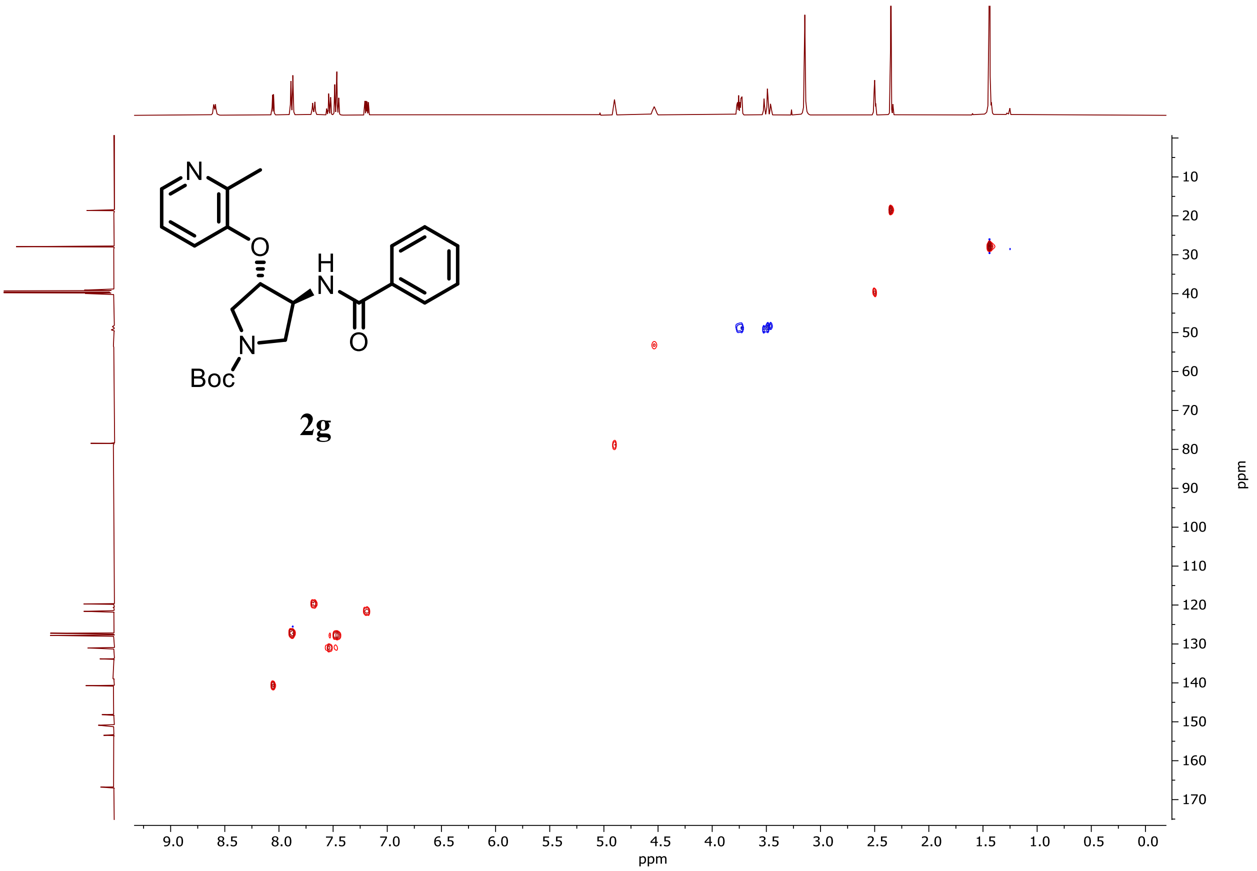

^{1}H NMR spectrum (400 MHz, DMSO-d_6, 343 K) of compound **2h**

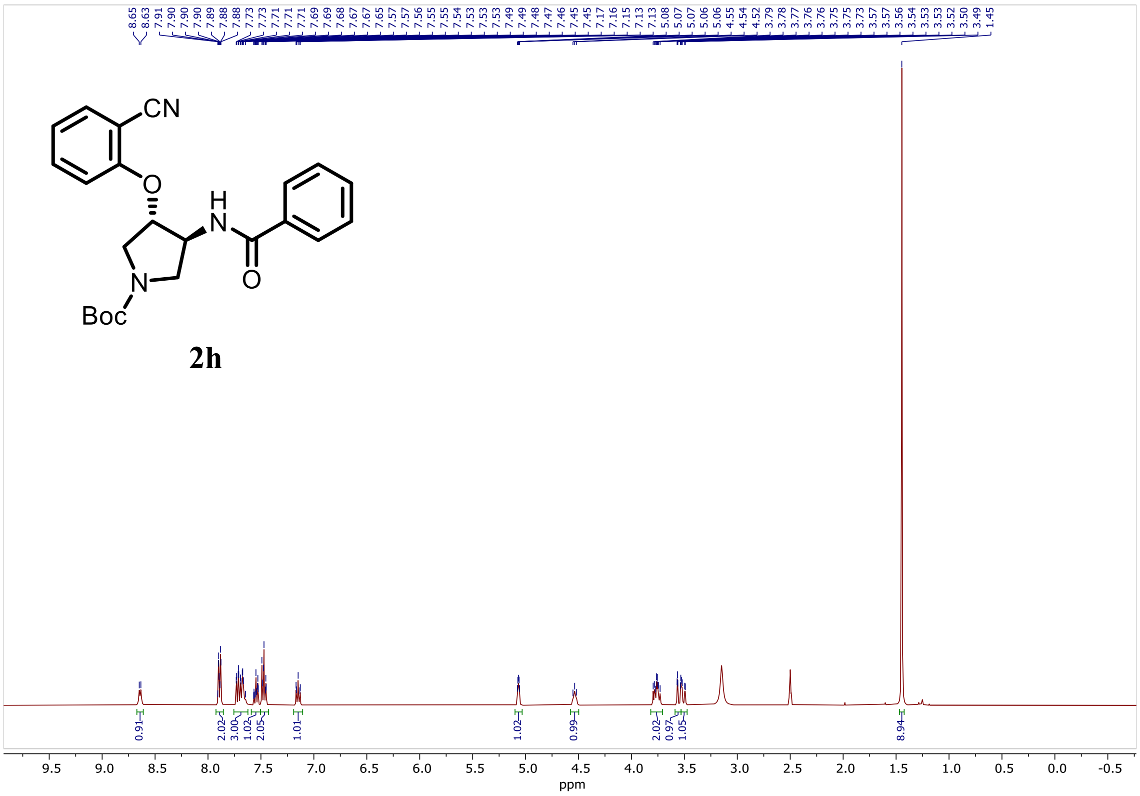

$^{13}C\{^{1}H\}$ NMR spectrum (101 MHz, DMSO-d_6, 343 K) of compound **2h**

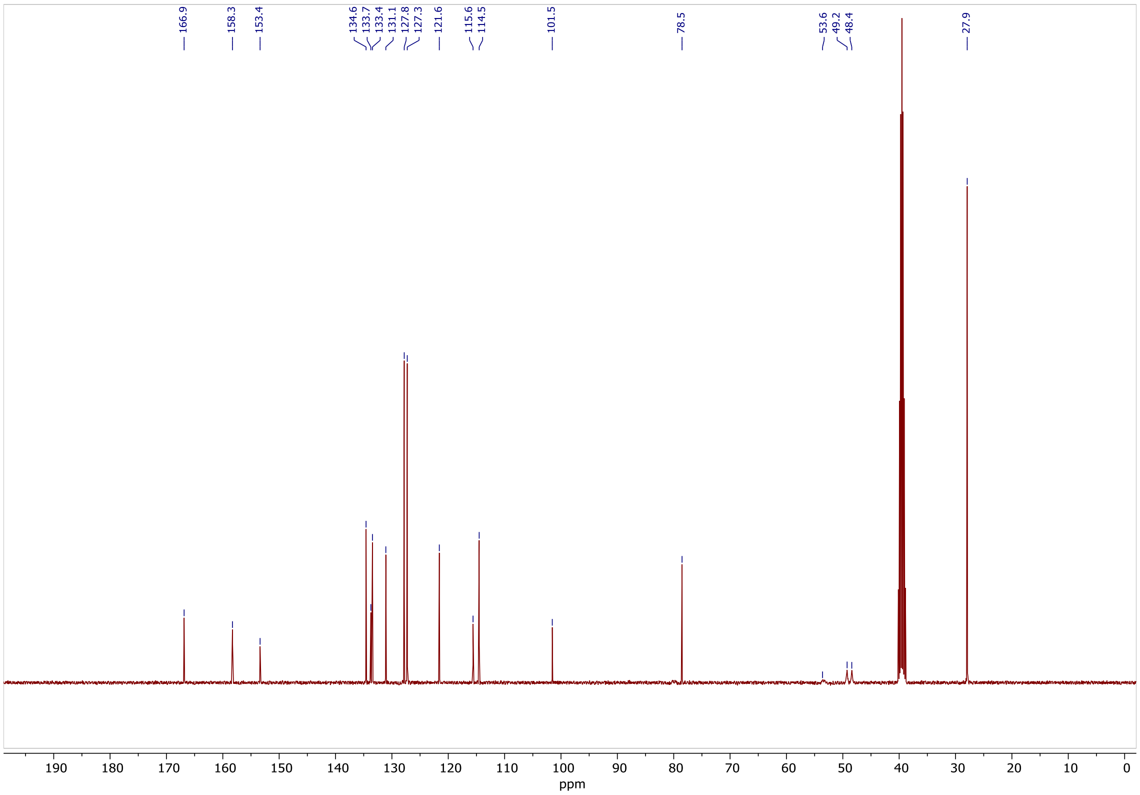

HSQC NMR spectrum (400 MHz, 101 MHz, DMSO-d_6, 343 K) of compound **2h**

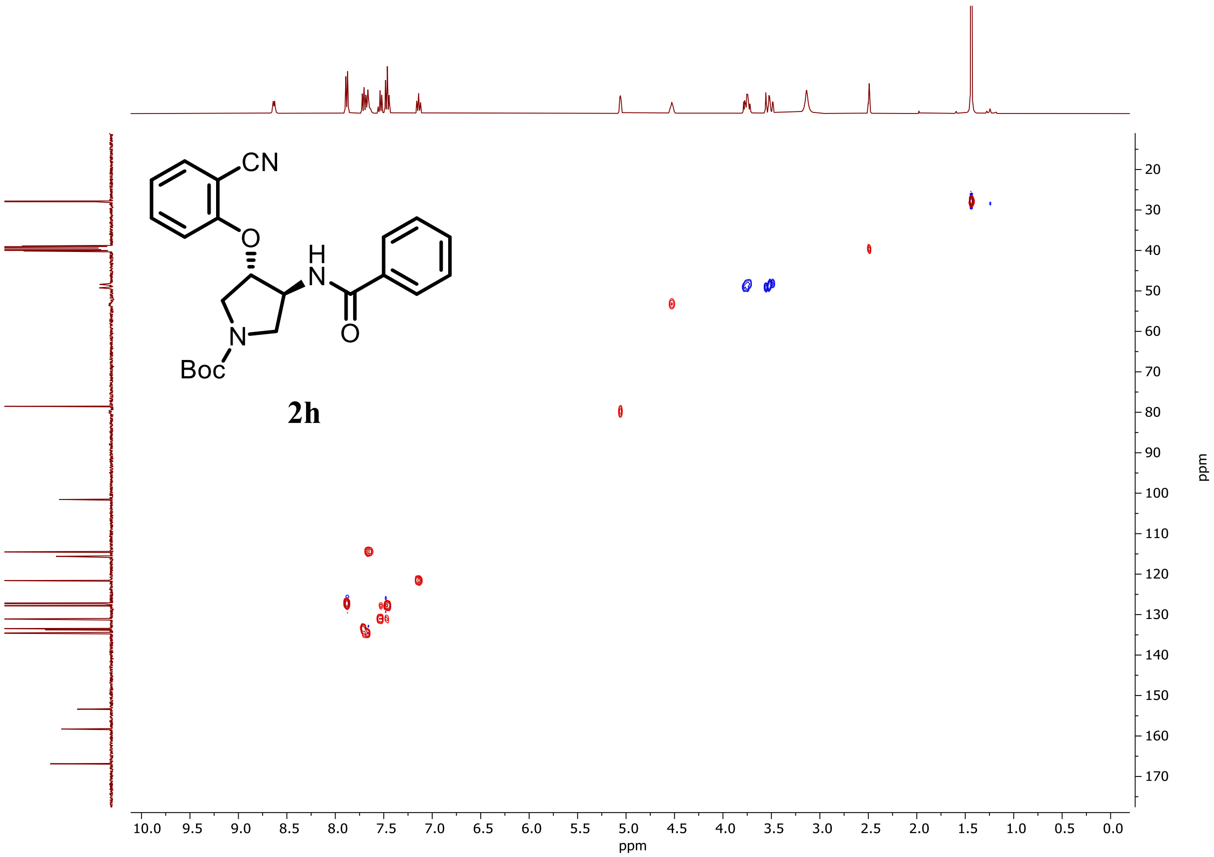

^{1}H NMR spectrum (400 MHz, DMSO-d_6, 343 K) of compound **2i**

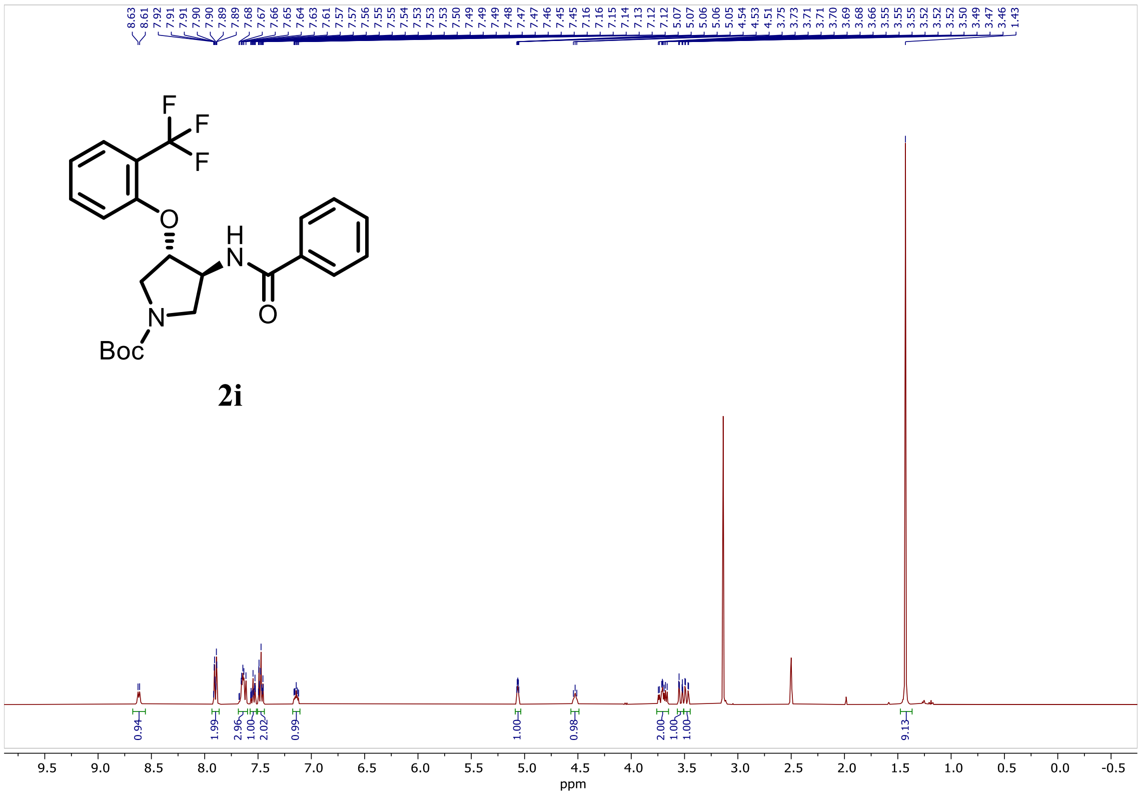

$^{13}C\{^{1}H\}$ NMR spectrum (101 MHz, DMSO-d_6, 343 K) of compound **2i**

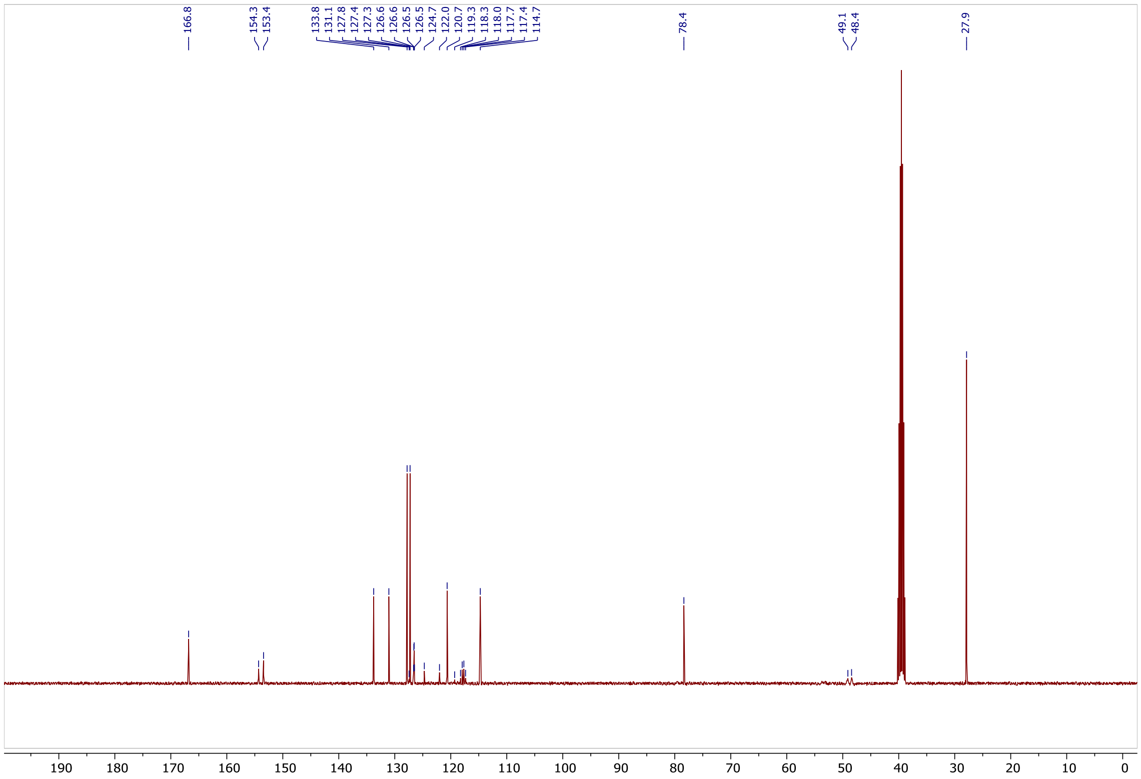

HSQC NMR spectrum (400 MHz, 101 MHz, DMSO-d_6, 343 K) of compound **2i**

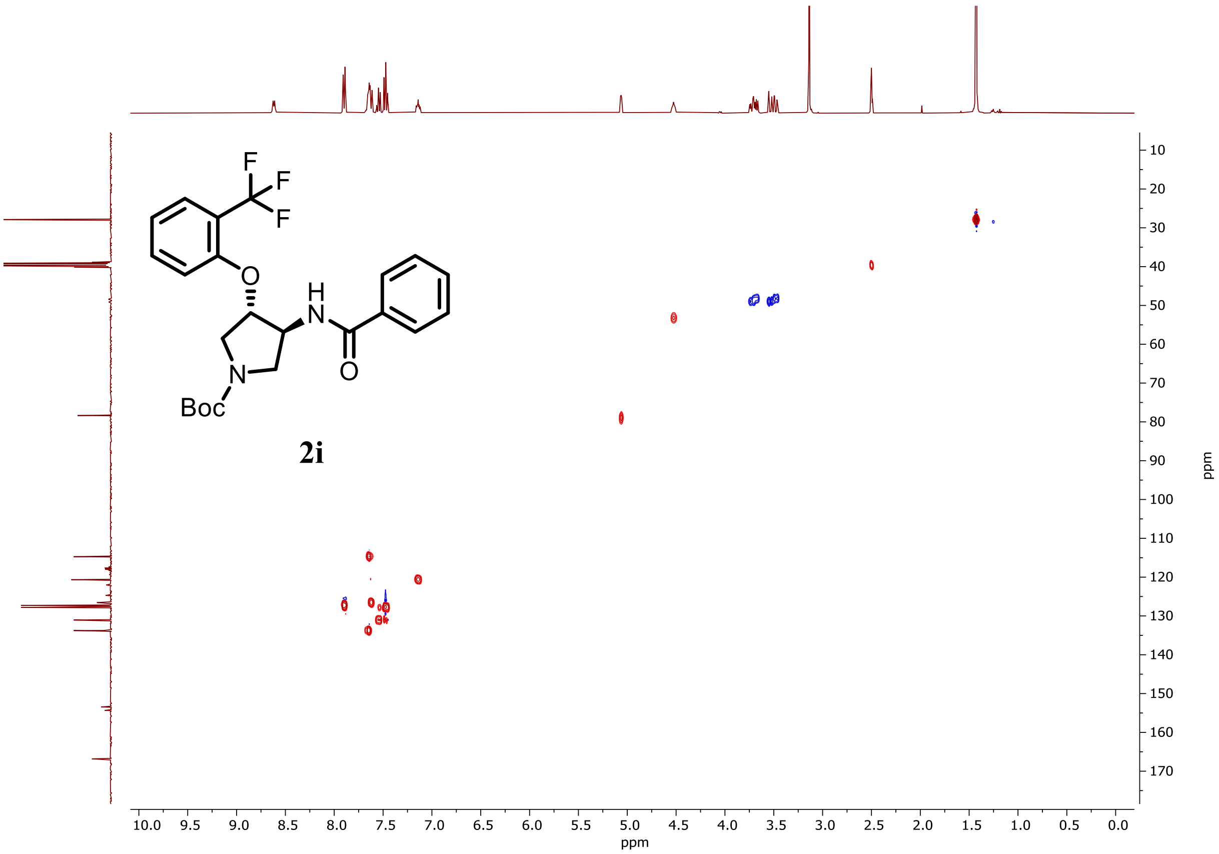

^{1}H NMR spectrum (400 MHz, DMSO-d_6, 343 K) of compound **2j’**

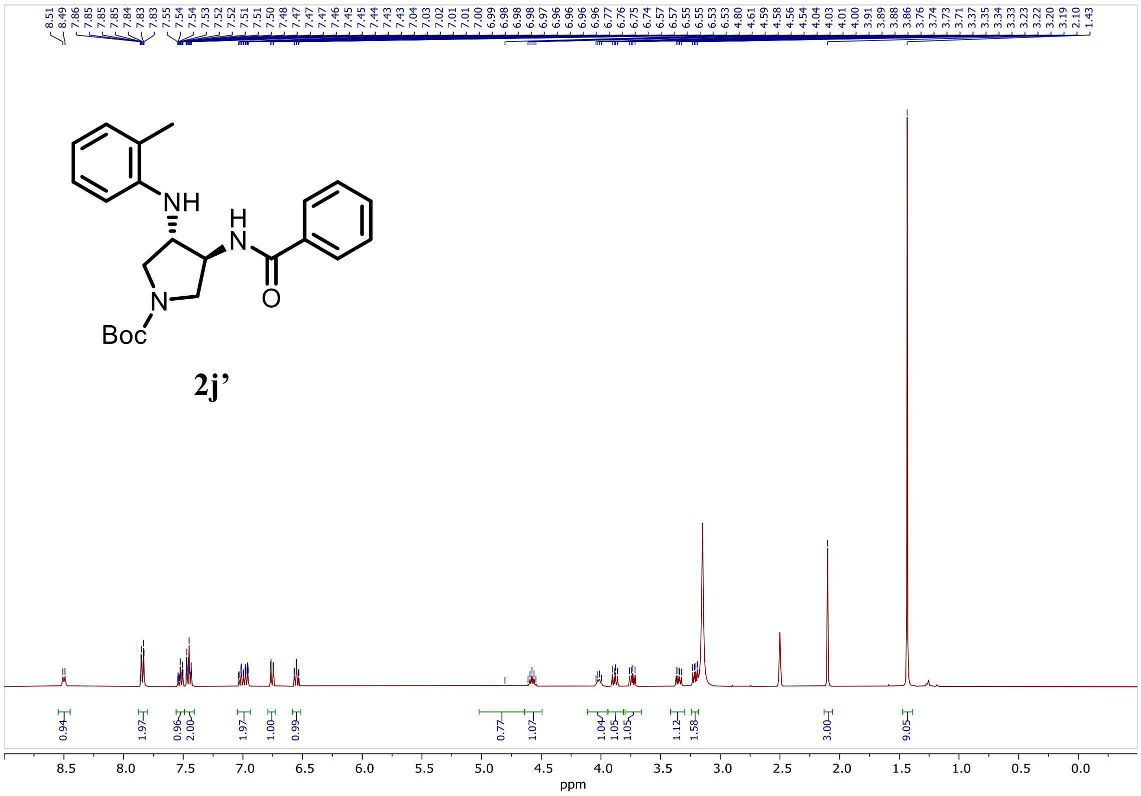

^{13}C{^{1}H} NMR spectrum (101 MHz, DMSO-d_6, 343 K) of compound **2j’**

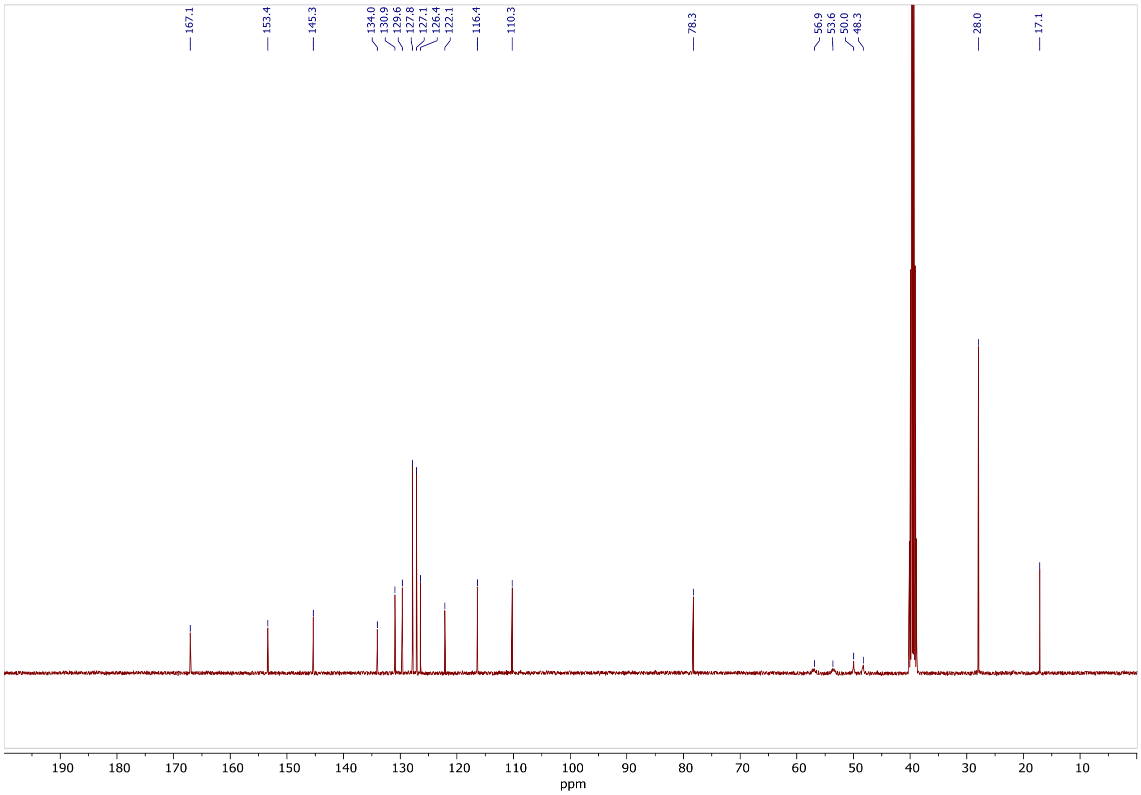

^{1}H NMR spectrum (400 MHz, DMSO-d_6, 343 K) of compound **2k**

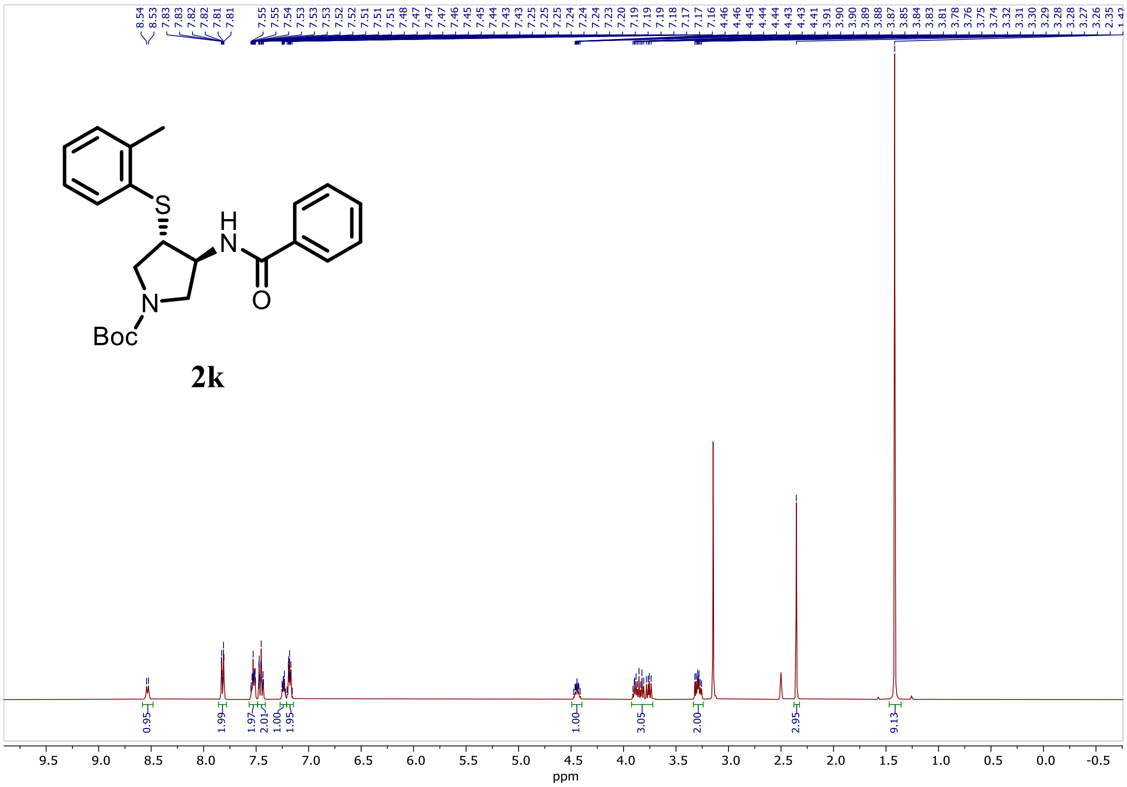

^{13}C{^{1}H} NMR spectrum (101 MHz, DMSO-d_6, 343 K) of compound **2k**

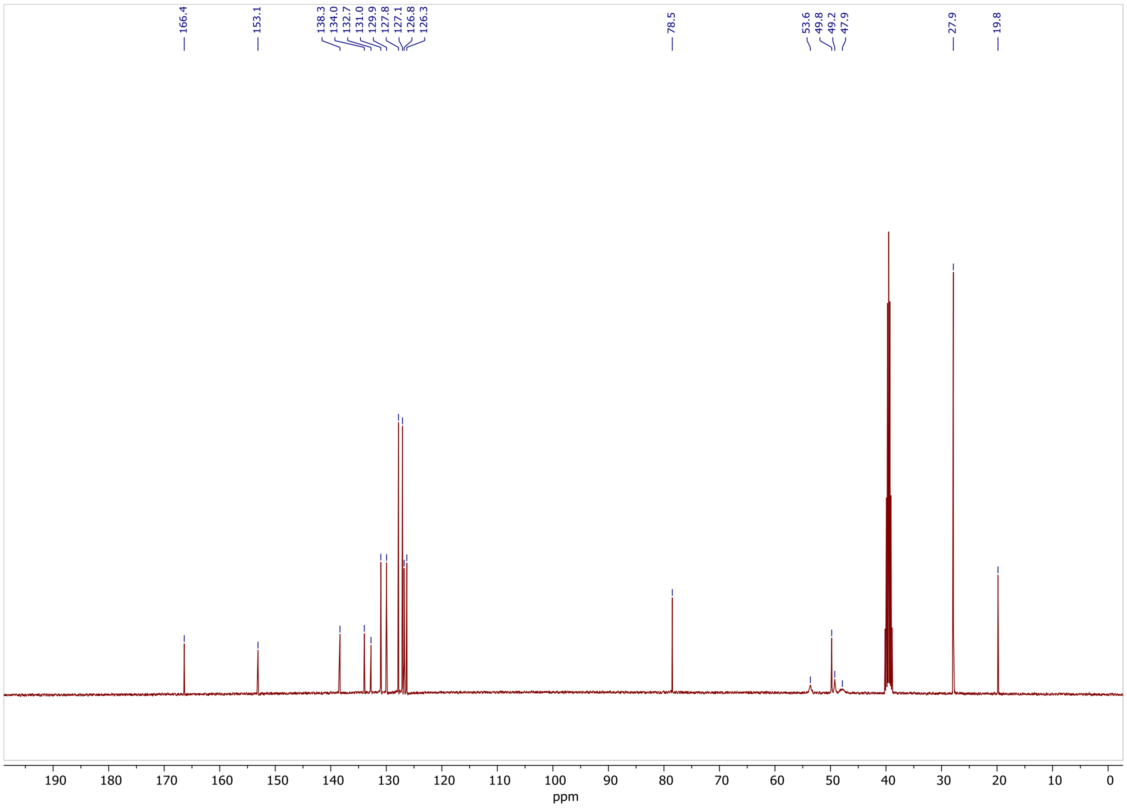

HSQC NMR spectrum (400 MHz, 101 MHz, DMSO-d_6, 343 K) of compound **2k**

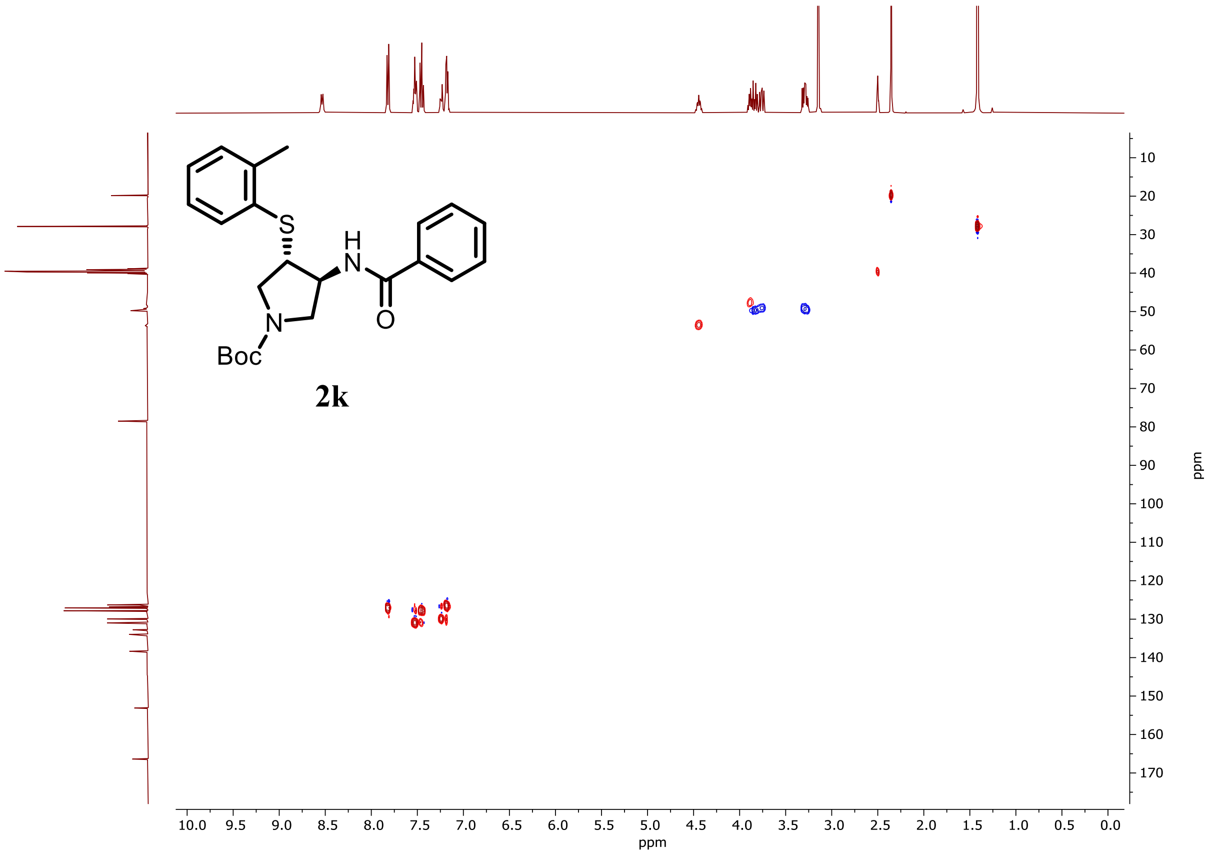

^{1}H NMR spectrum (400 MHz, DMSO-d_6, 343 K) of compound **2l**

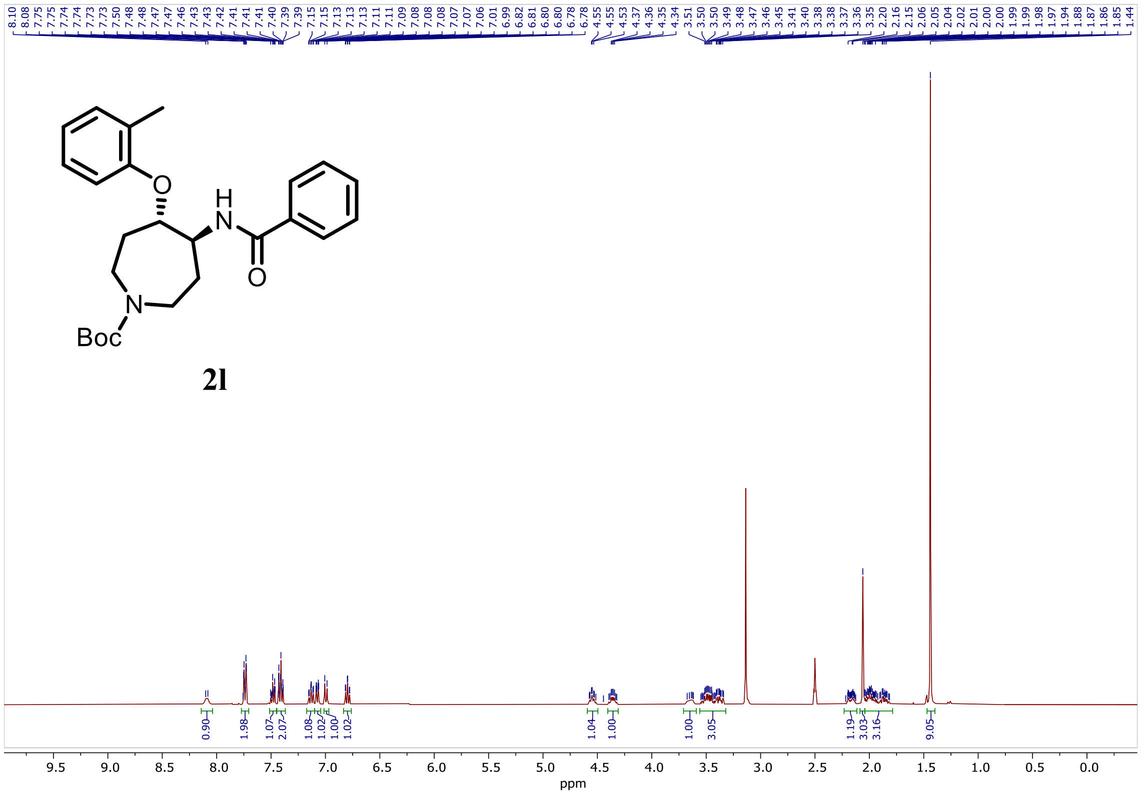

$^{13}C\{^{1}H\}$ NMR spectrum (101 MHz, DMSO-d_6, 343 K) of compound **2l**

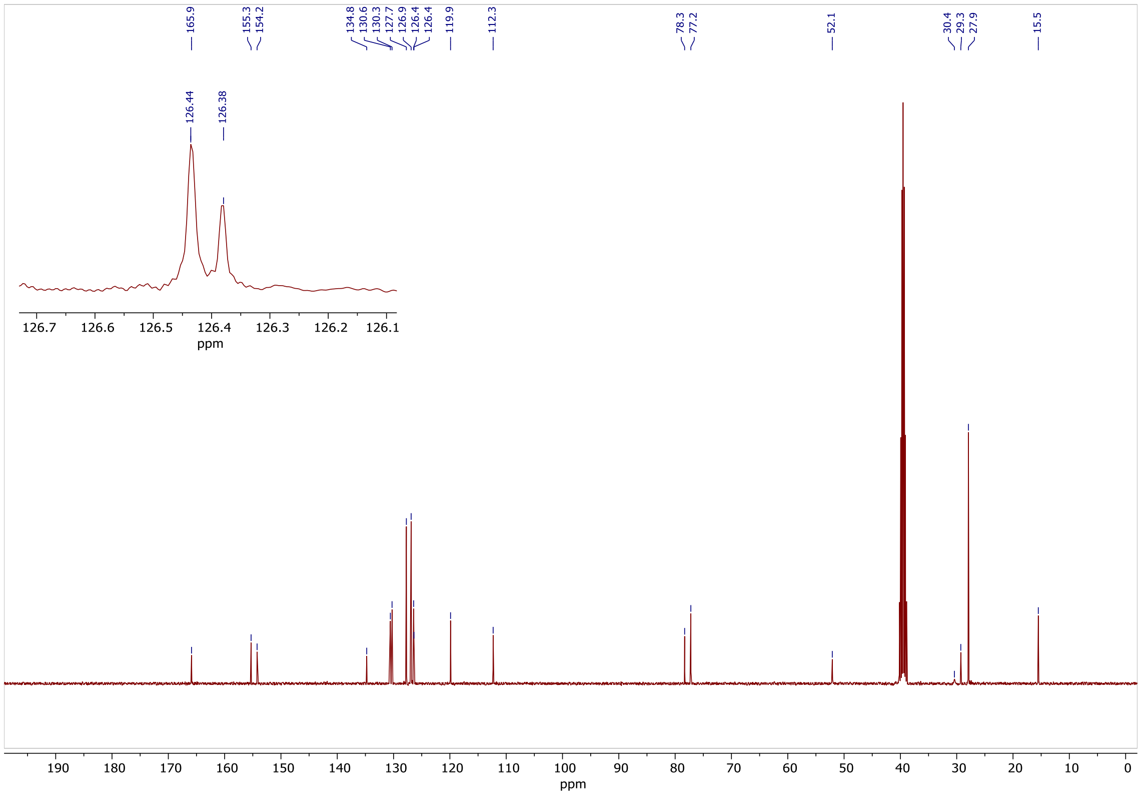

HSQC NMR spectrum (400 MHz, 101 MHz, DMSO-d_6, 343 K) of compound **2l**

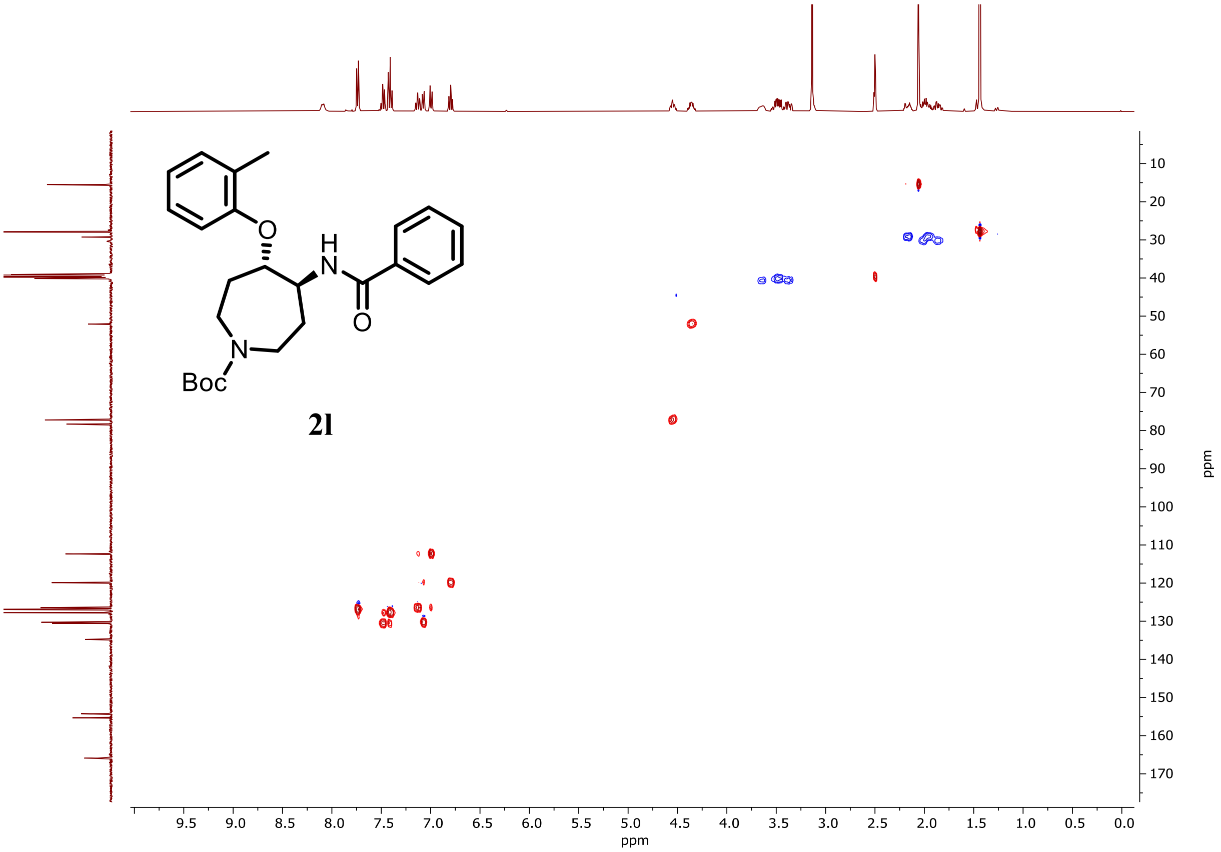

^{1}H NMR spectrum (400 MHz, $CDCl_3$, 298 K) of compound **g**

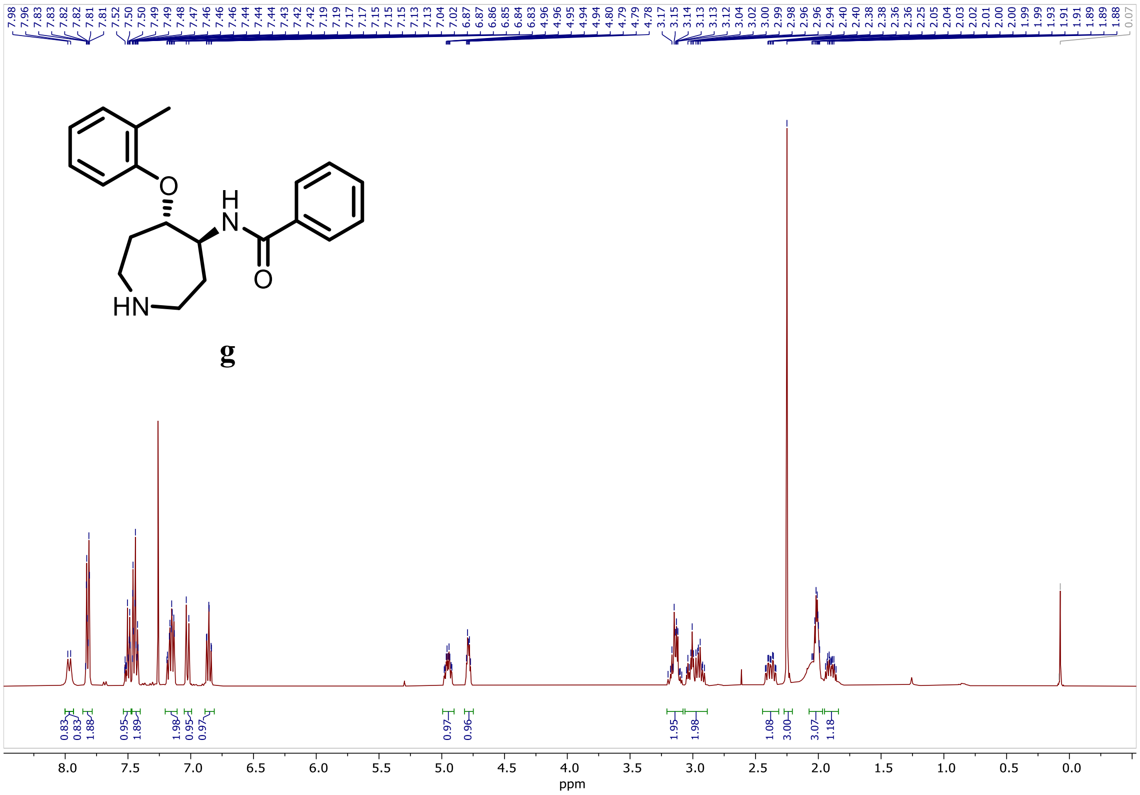

^{13}C{^{1}H} NMR spectrum (101 MHz, $CDCl_3$, 298 K) of compound **g**

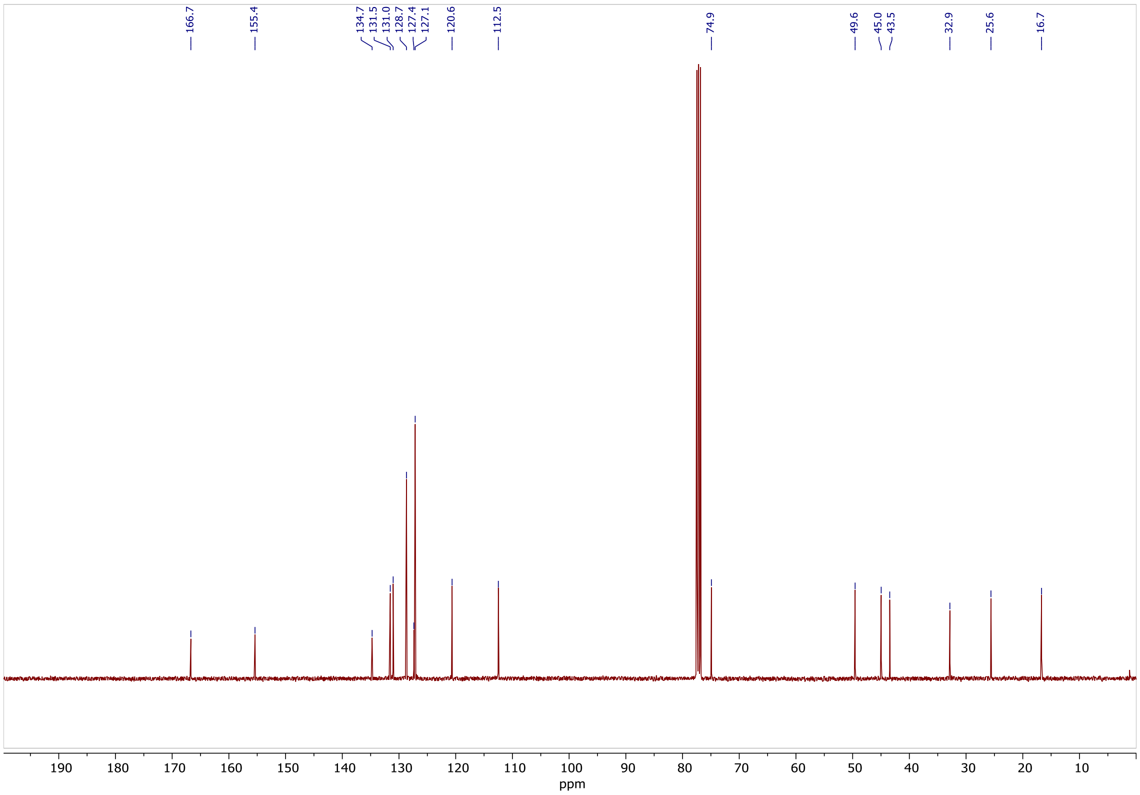

^{1}H NMR spectrum (400 MHz, DMSO-d_6, 343 K) of compound **2m**

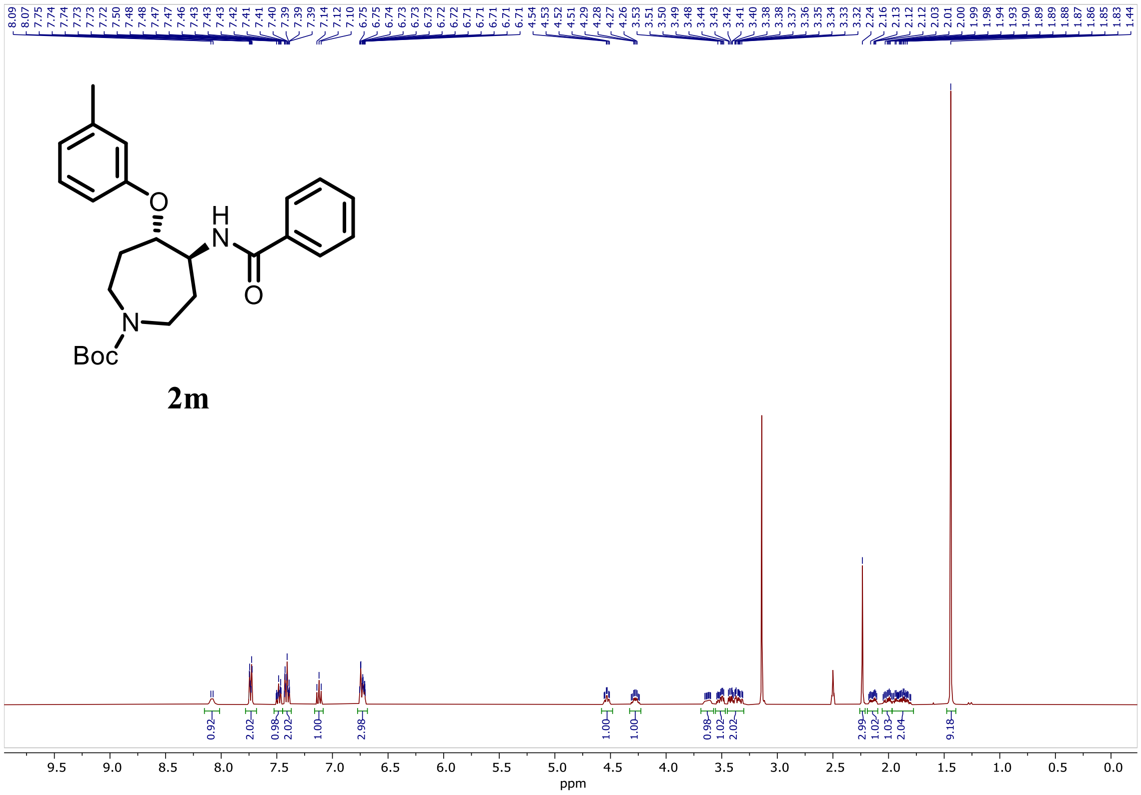

^{13}C{^{1}H} NMR spectrum (101 MHz, DMSO-d_6, 343 K) of compound **2m**

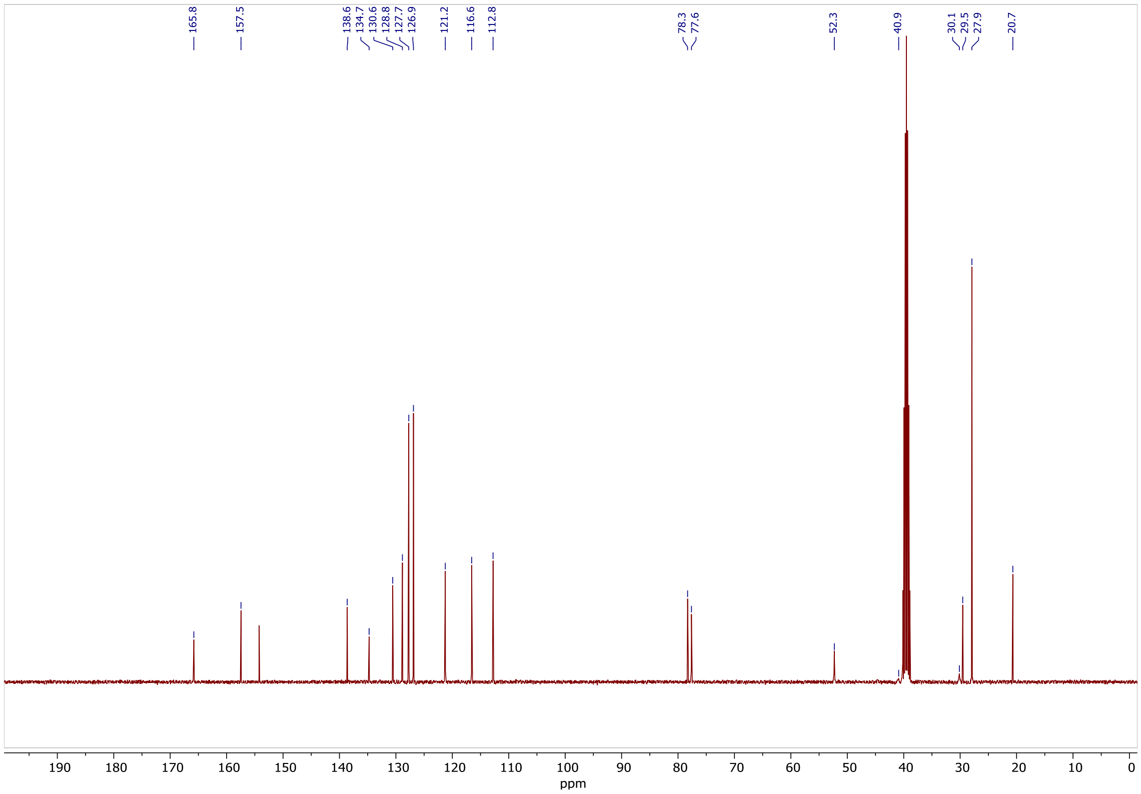

HSQC NMR spectrum (400 MHz, 101 MHz, DMSO-d_6, 343 K) of compound **2m**

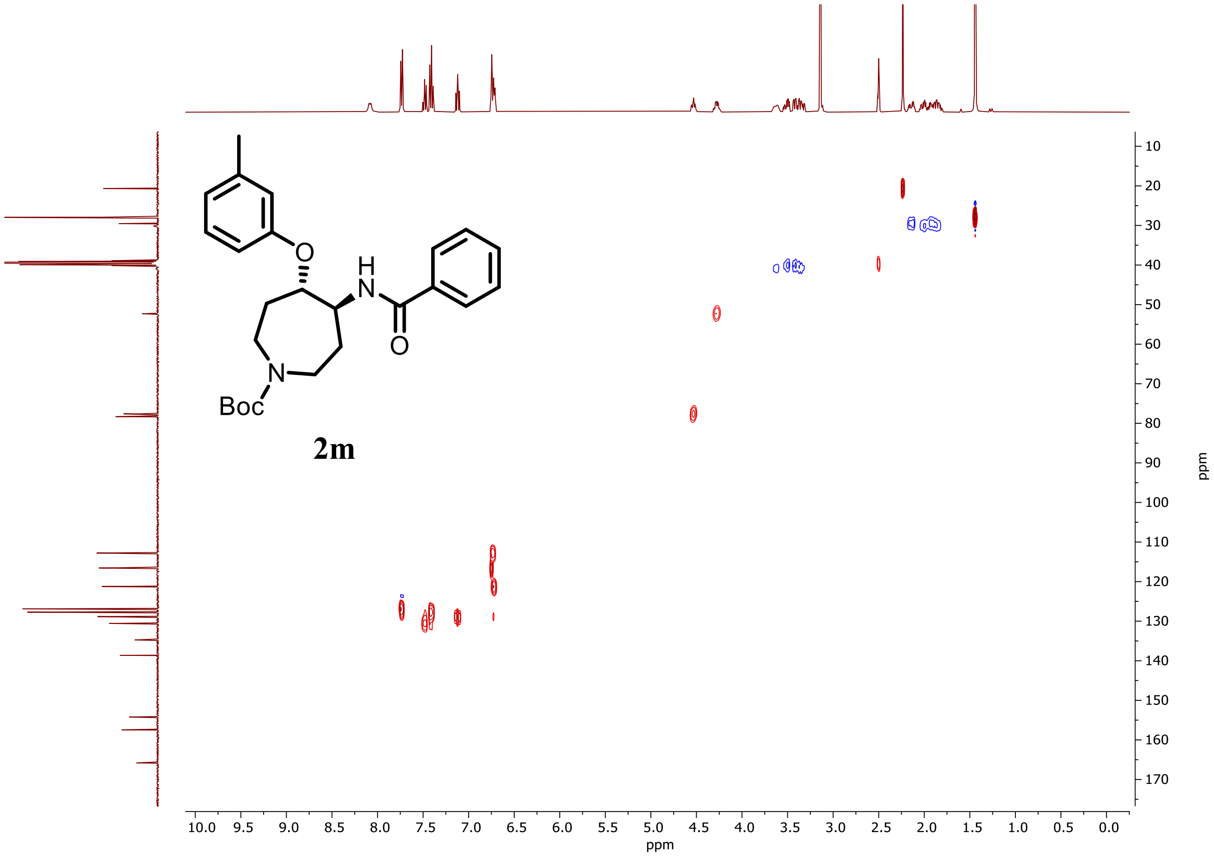

^{1}H NMR spectrum (400 MHz, DMSO-d_6, 343 K) of compound **2n**

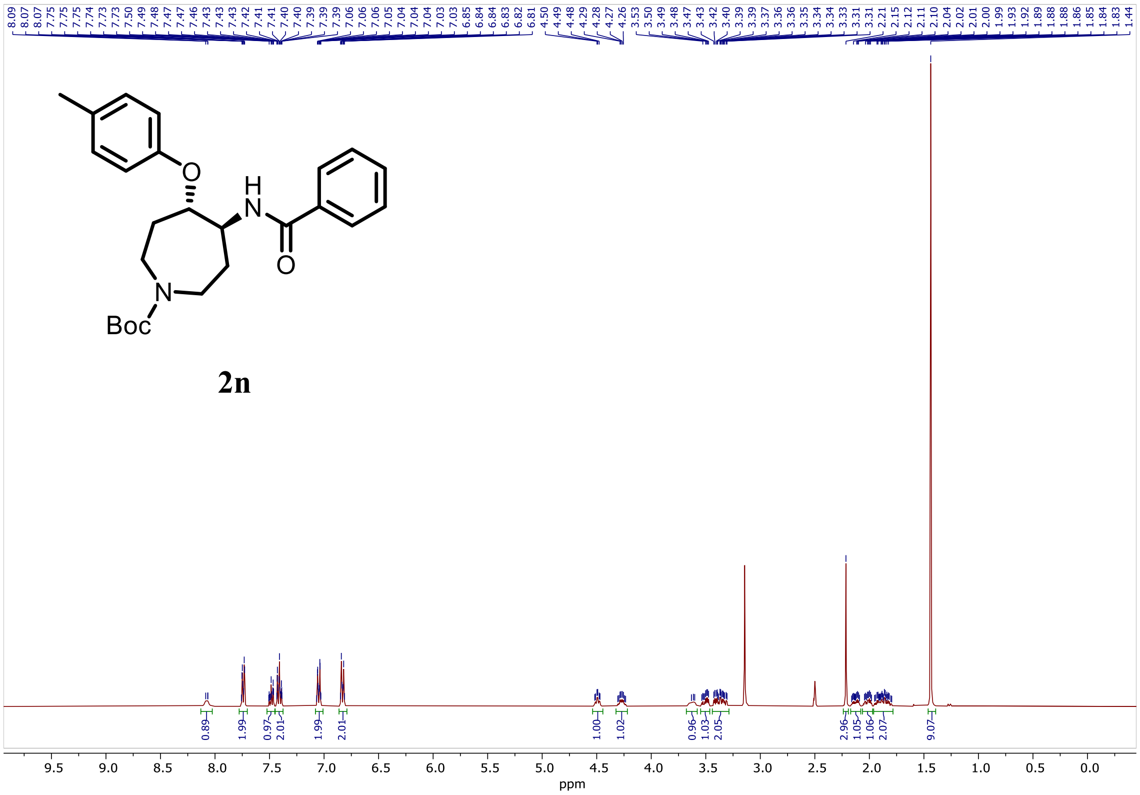

^{13}C{^{1}H} NMR spectrum (101 MHz, DMSO-d_6, 343 K) of compound **2n**

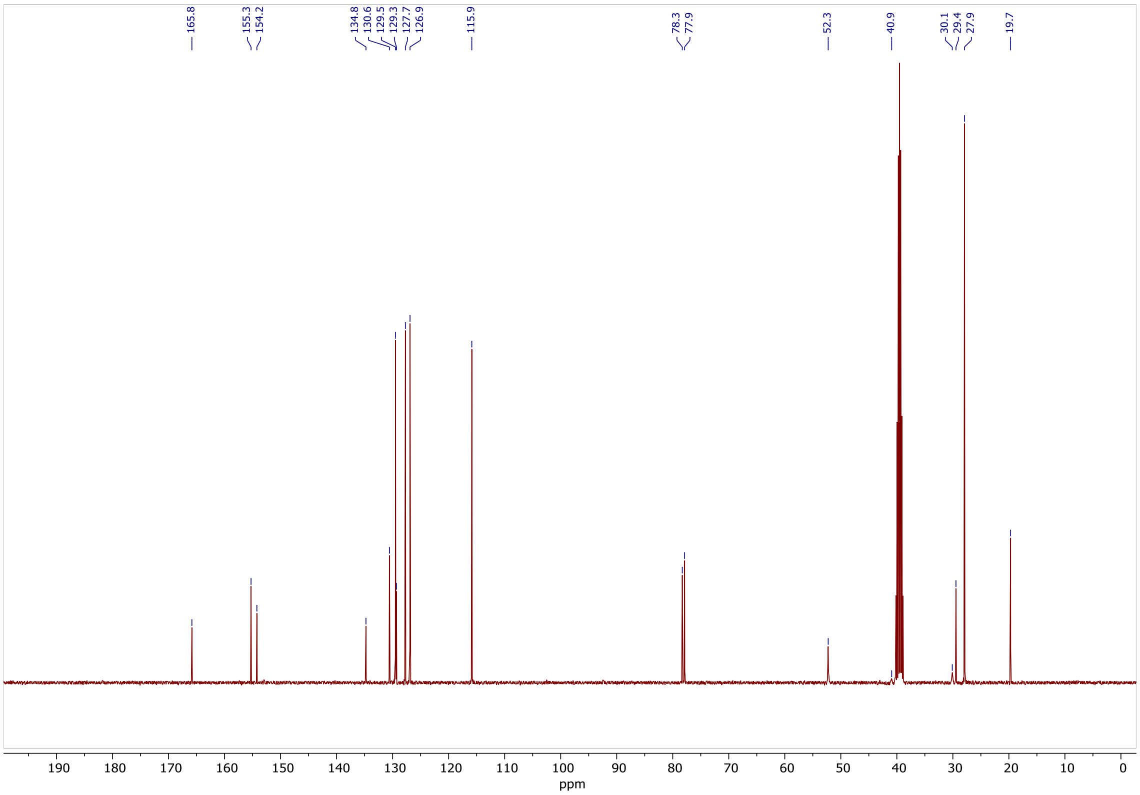

HSQC NMR spectrum (400 MHz, 101 MHz, DMSO-d_6, 343 K) of compound **2n**

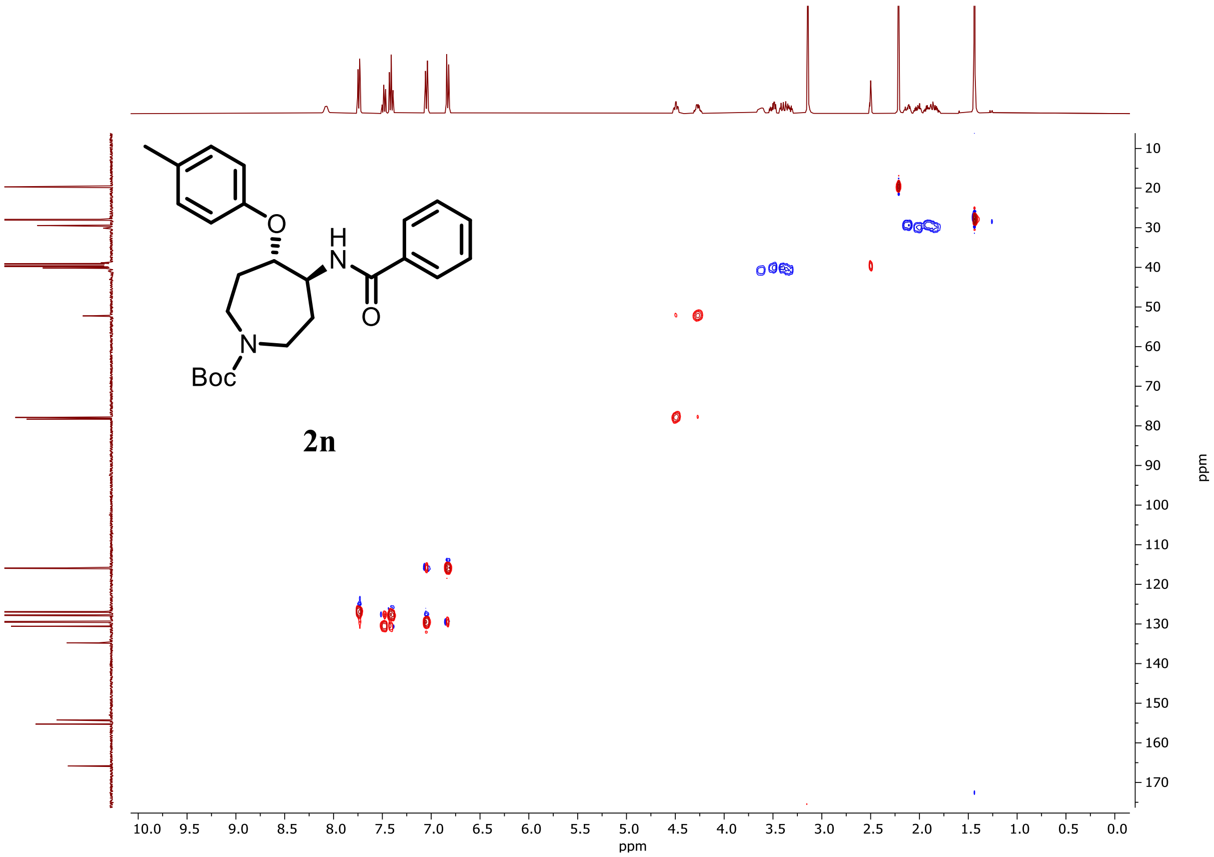

^{1}H NMR spectrum (400 MHz, DMSO-d_6, 343 K) of compound **2o**

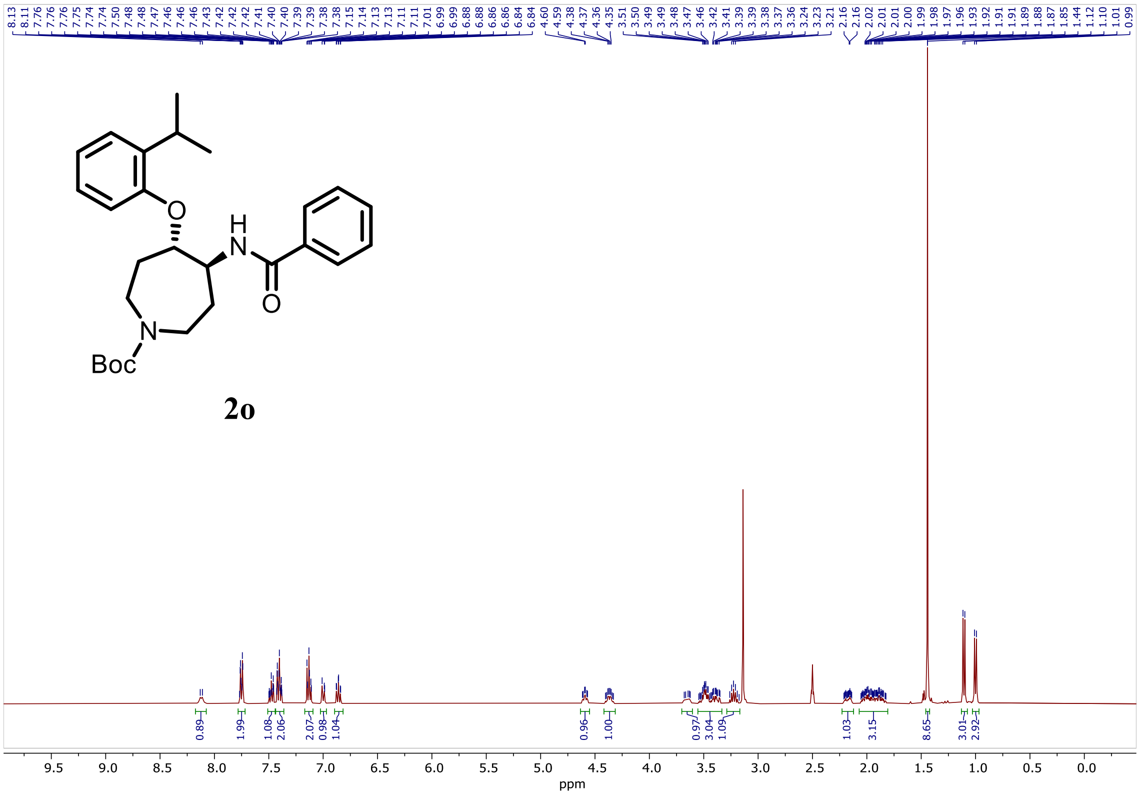

^{13}C{^{1}H} NMR spectrum (101 MHz, DMSO-d_6, 343 K) of compound **2o**

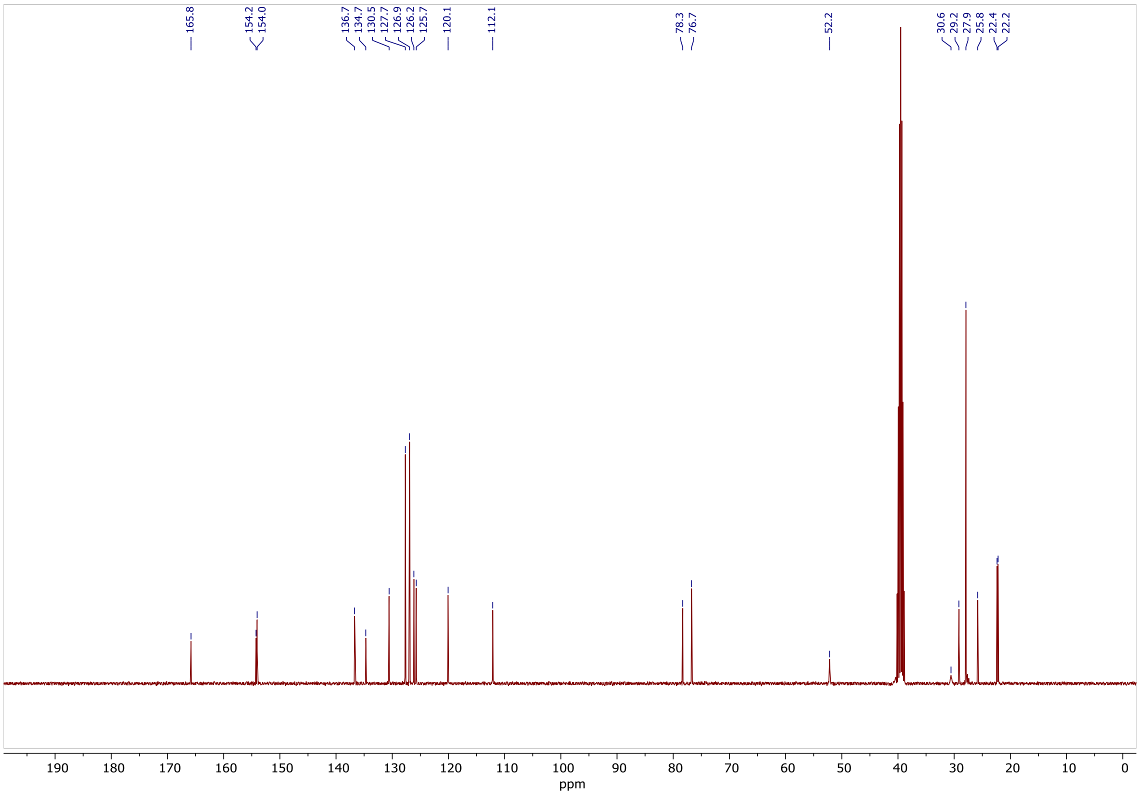

HSQC NMR spectrum (400 MHz, 101 MHz, DMSO-d_6, 343 K) of compound **2o**

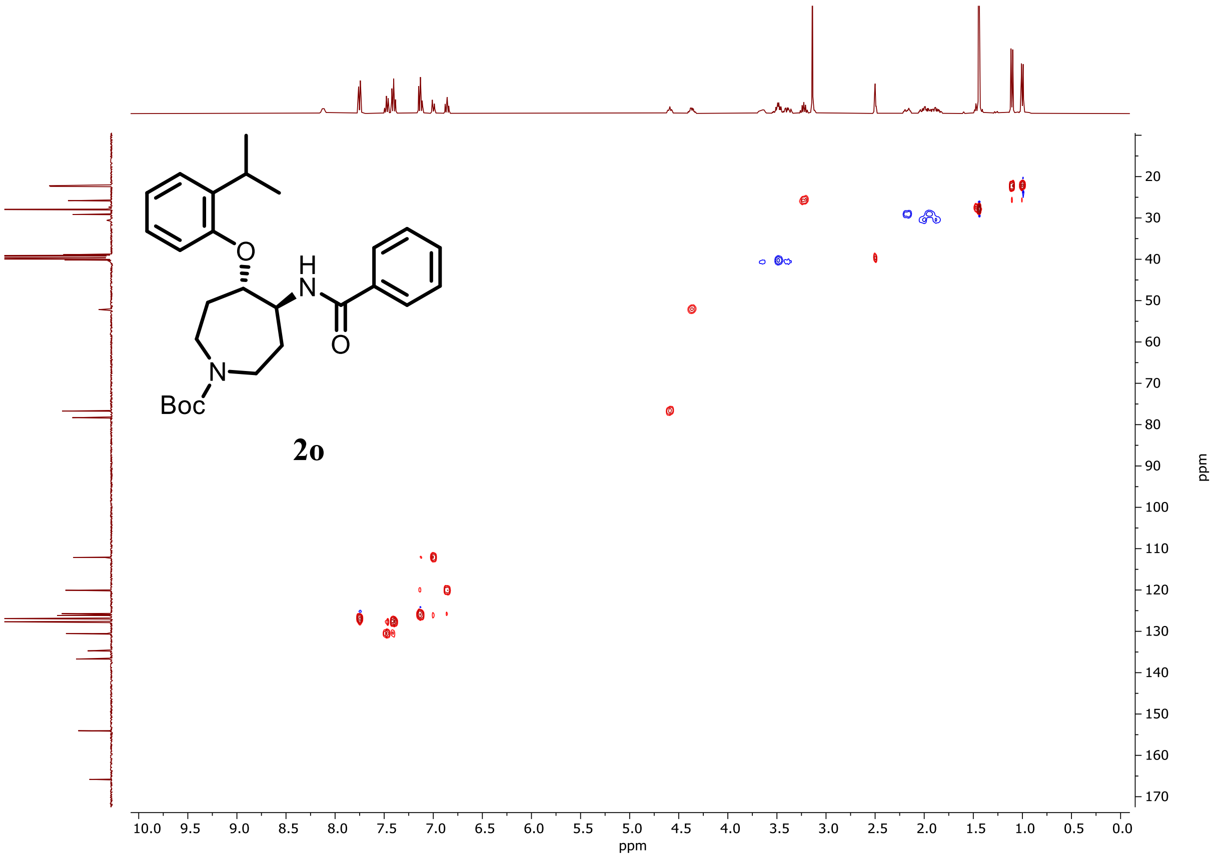

^{1}H NMR spectrum (400 MHz, DMSO-d_6, 343 K) of compound **2p**

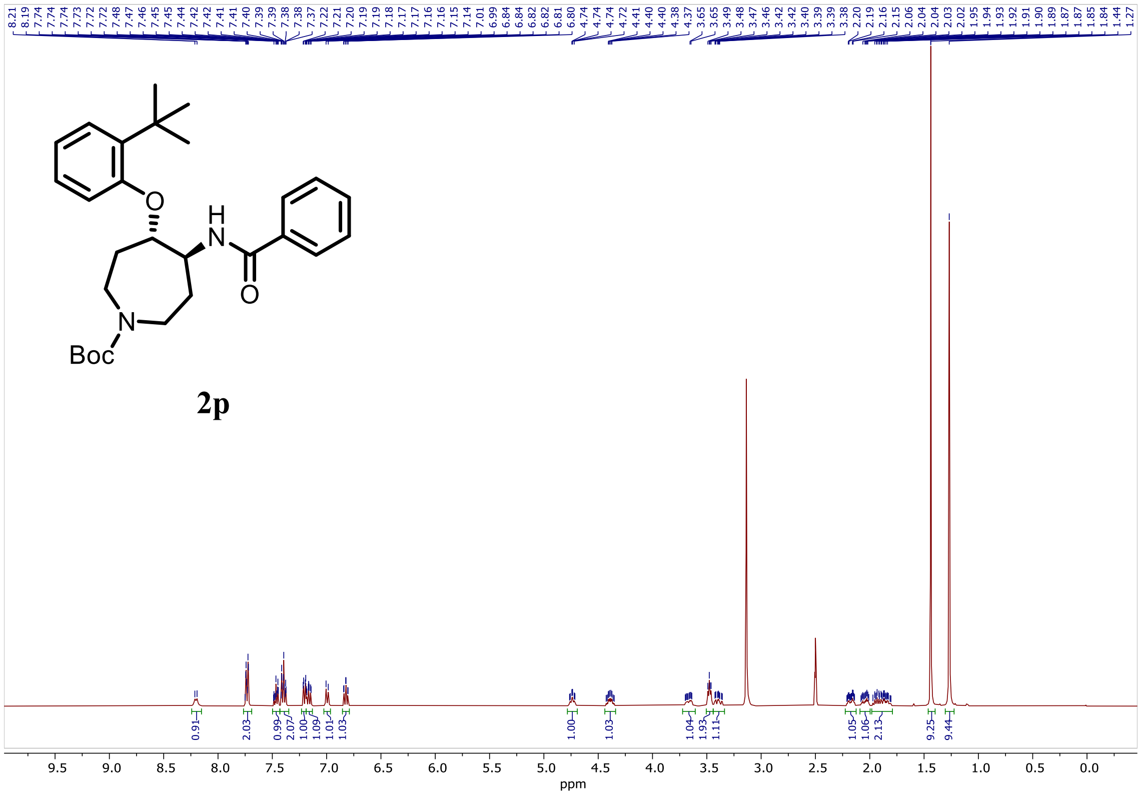

^{13}C{^{1}H} NMR spectrum (101 MHz, DMSO-d_6, 343 K) of compound **2p**

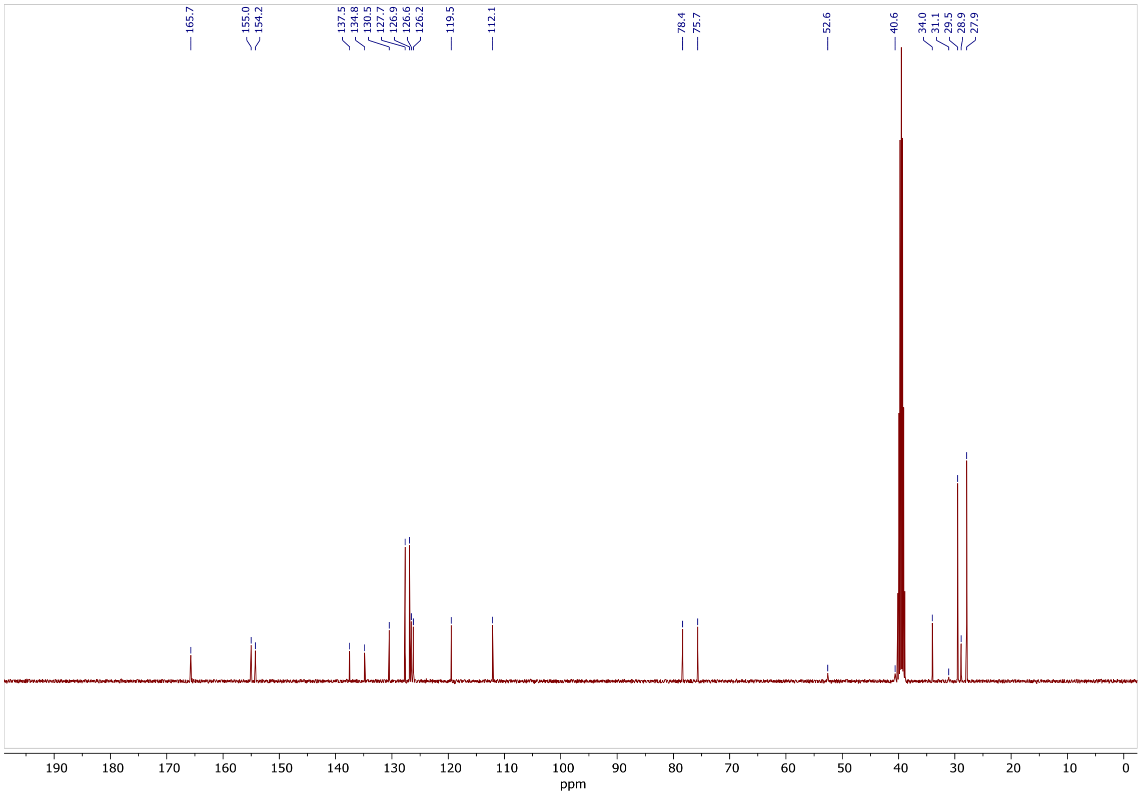

HSQC NMR spectrum (400 MHz, 101 MHz, DMSO-d_6, 343 K) of compound **2p**

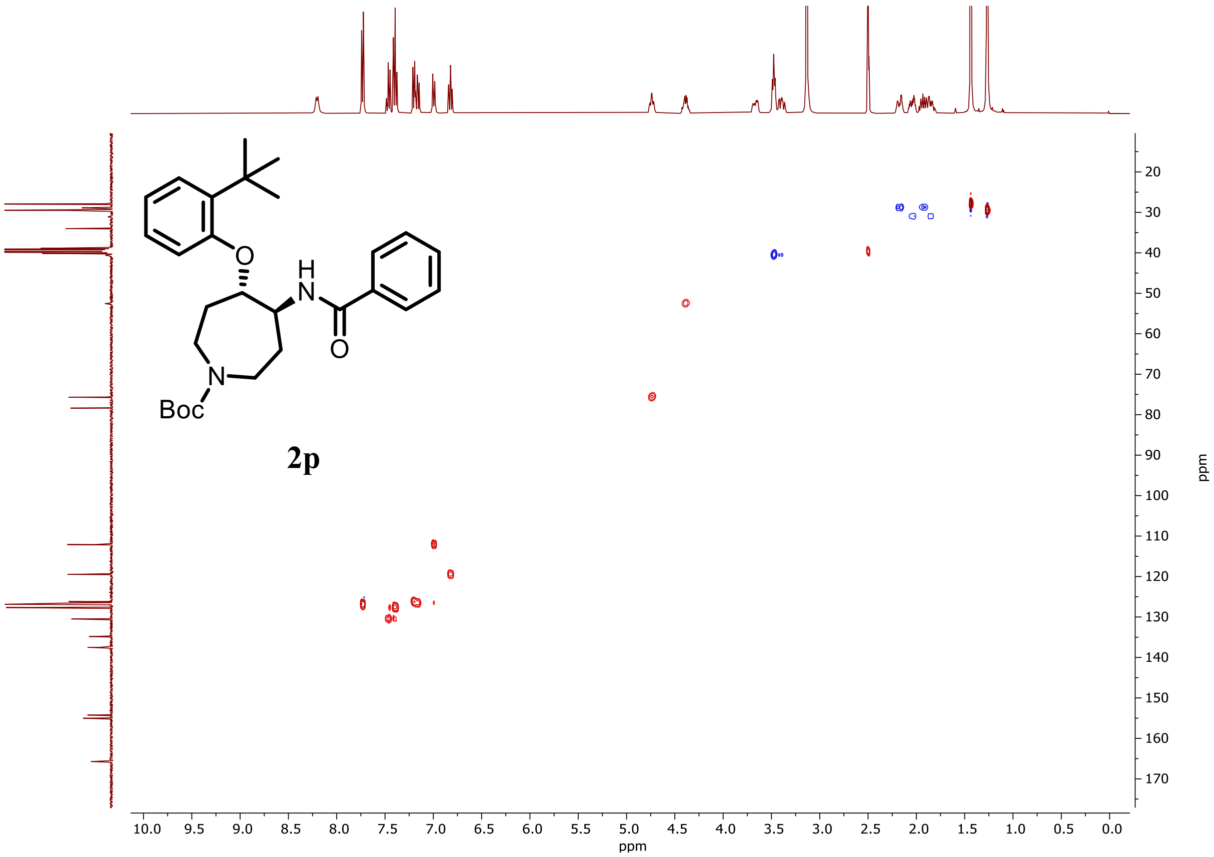

^{1}H NMR spectrum (400 MHz, DMSO-d_6, 343 K) of compound **2q**

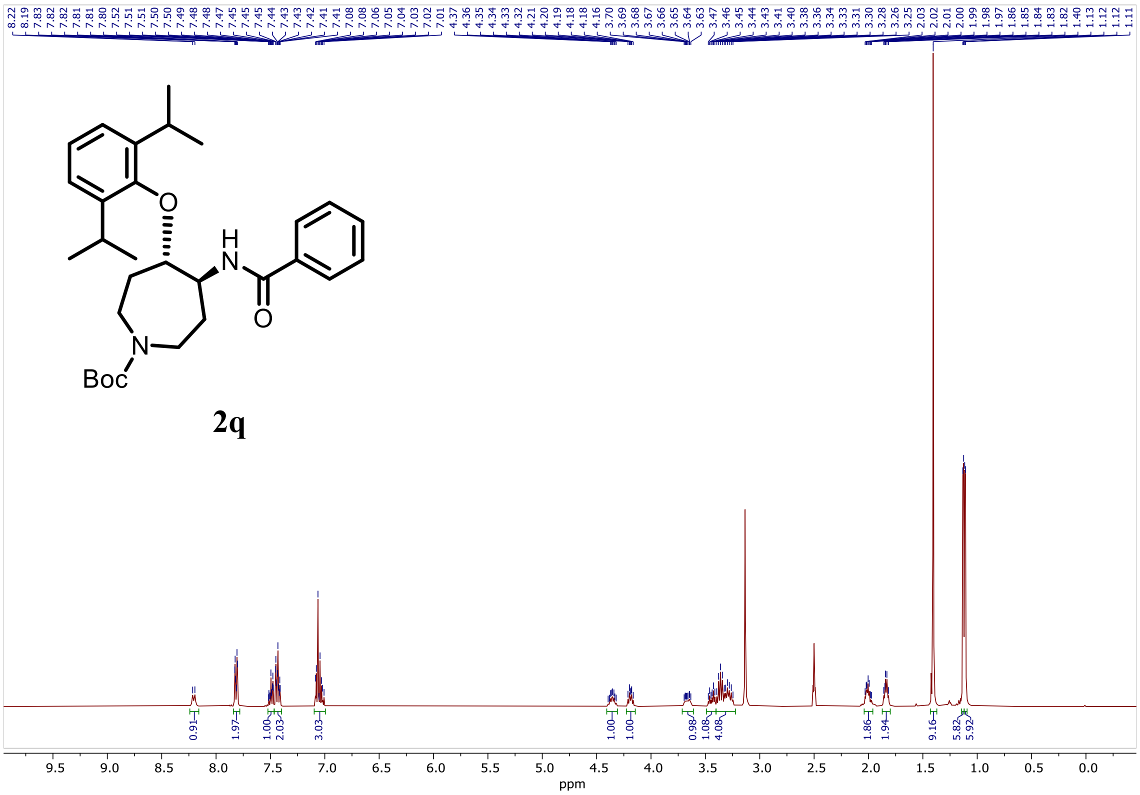

^{13}C{^{1}H} NMR spectrum (101 MHz, DMSO-d_6, 343 K) of compound **2q**

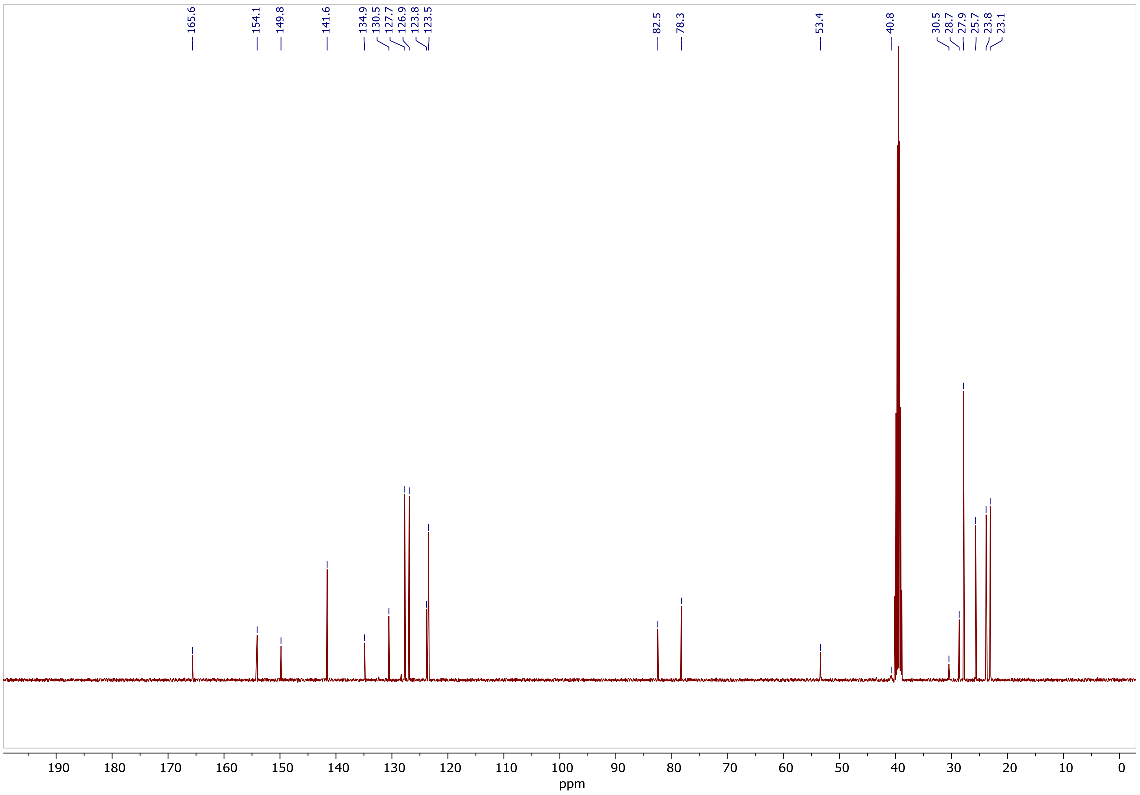

HSQC NMR spectrum (400 MHz, 101 MHz, DMSO-d_6, 343 K) of compound **2q**

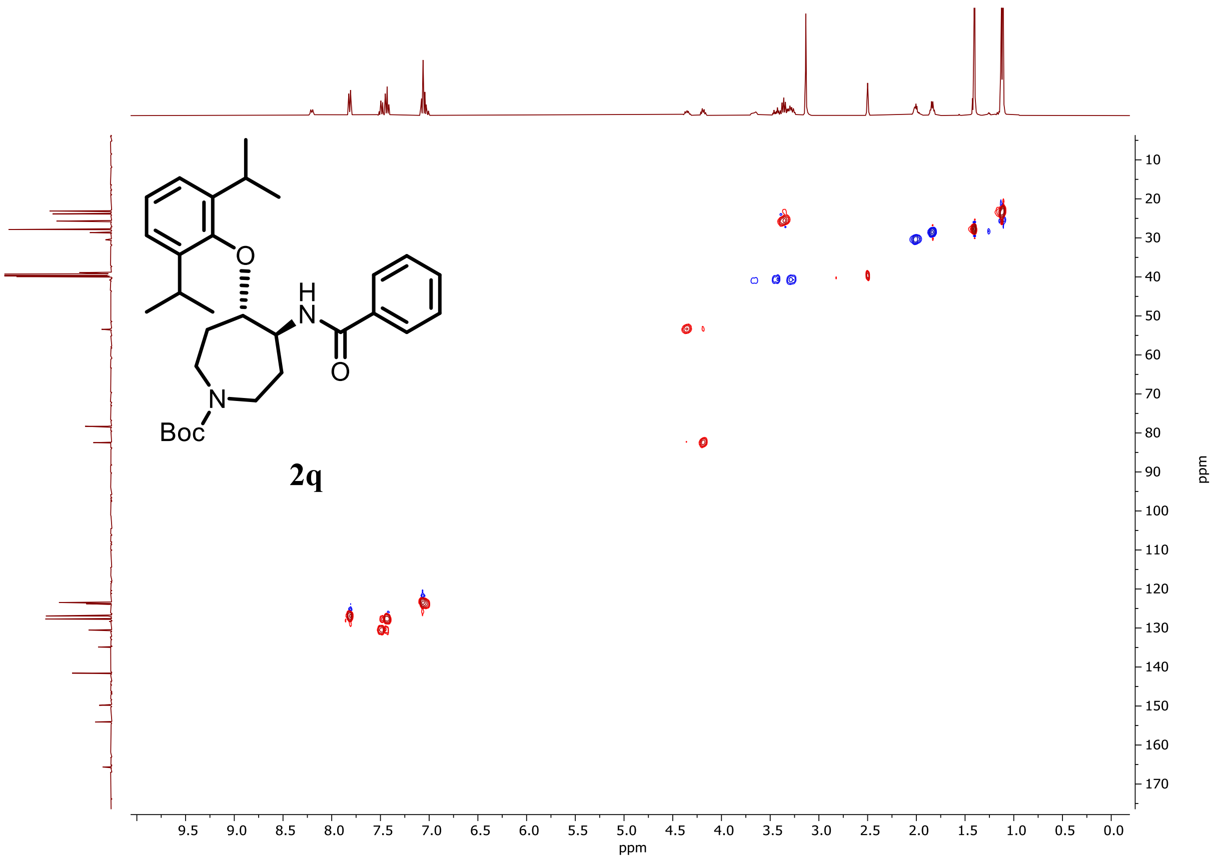

^{1}H NMR spectrum (400 MHz, DMSO-d_6, 343 K) of compound **2r**

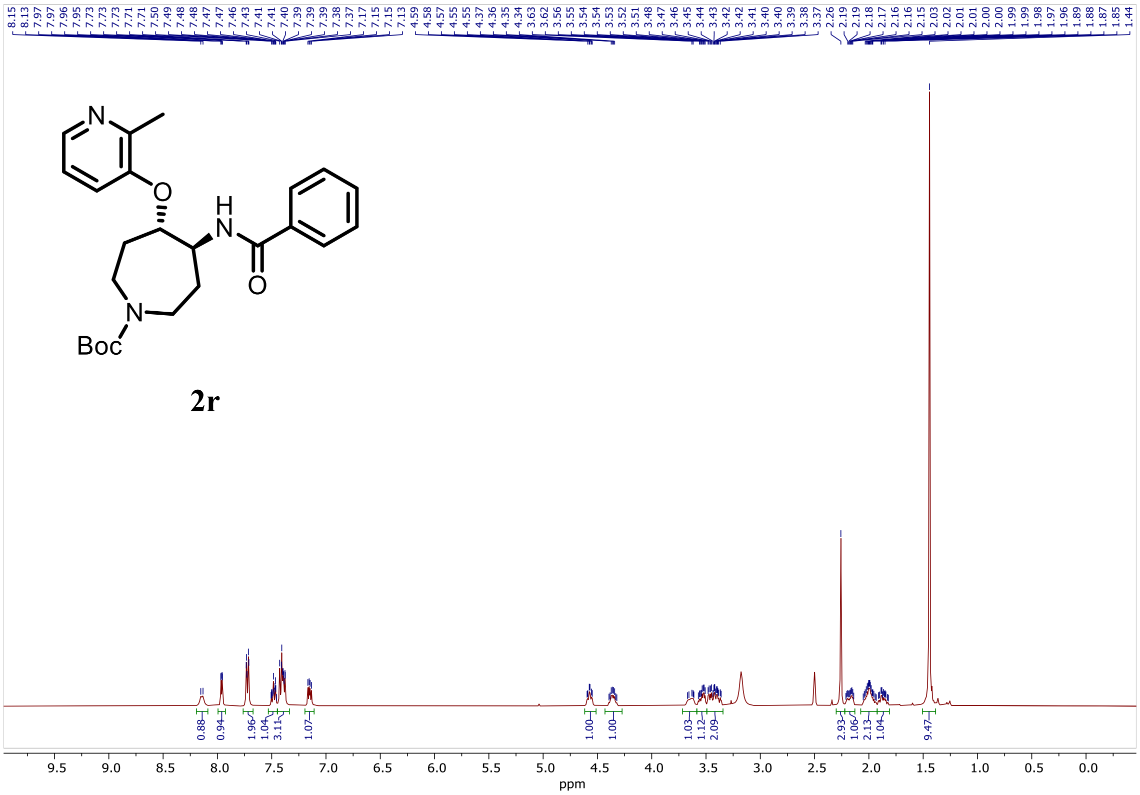

^{13}C{^{1}H} NMR spectrum (101 MHz, DMSO-d_6, 343 K) of compound **2r**

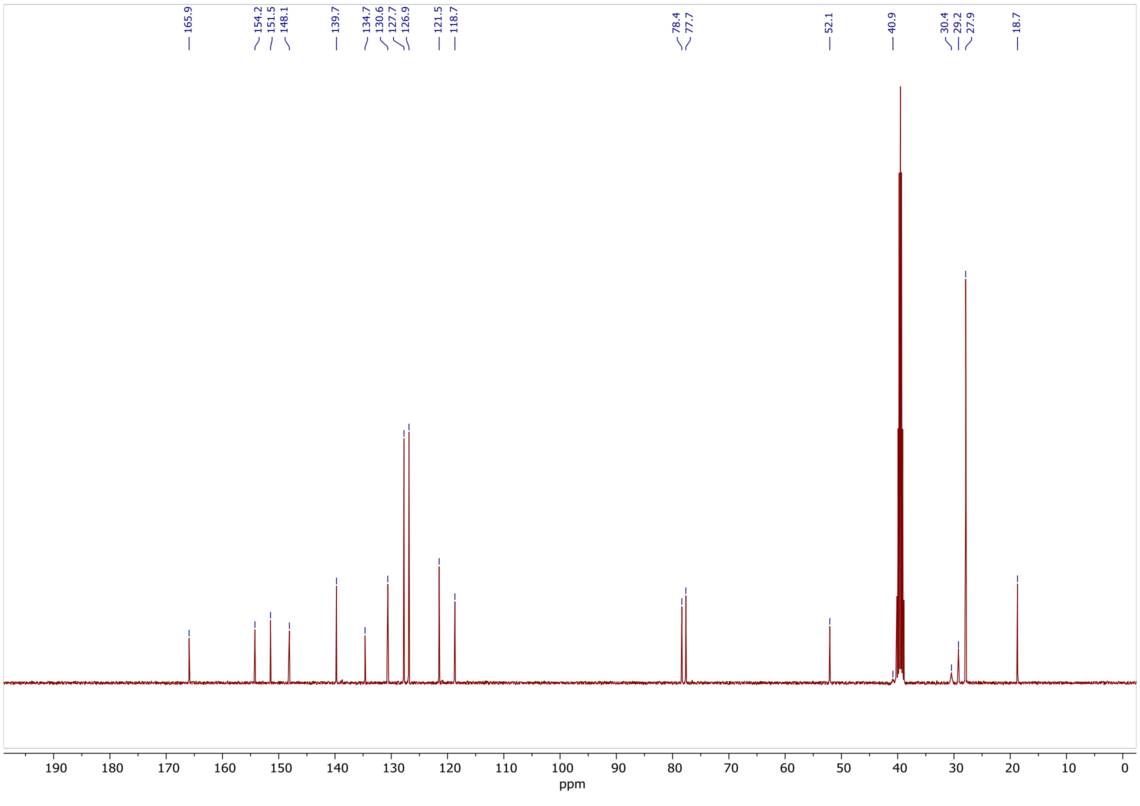

HSQC NMR spectrum (400 MHz, 101 MHz, DMSO-d_6, 343 K) of compound **2r**

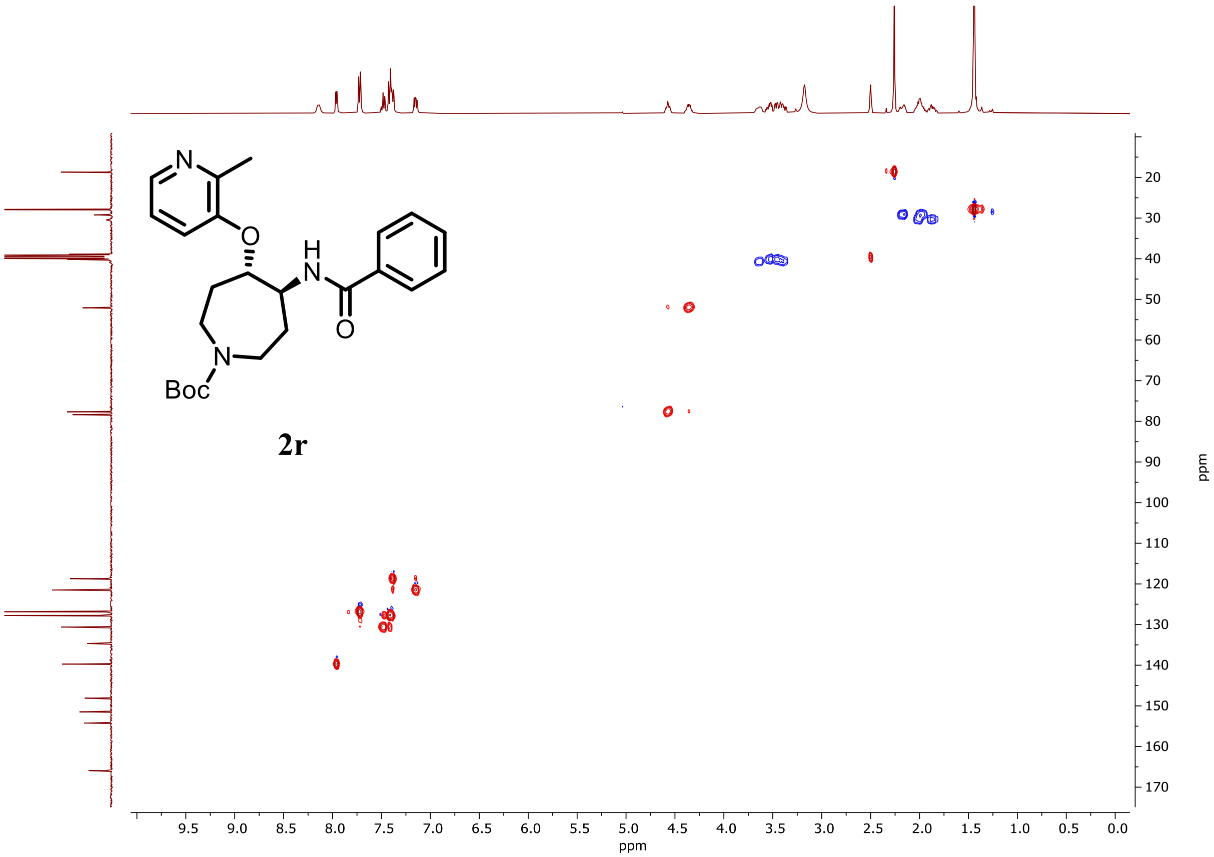

^{1}H NMR spectrum (400 MHz, DMSO-d_6, 343 K) of compound **4aa**

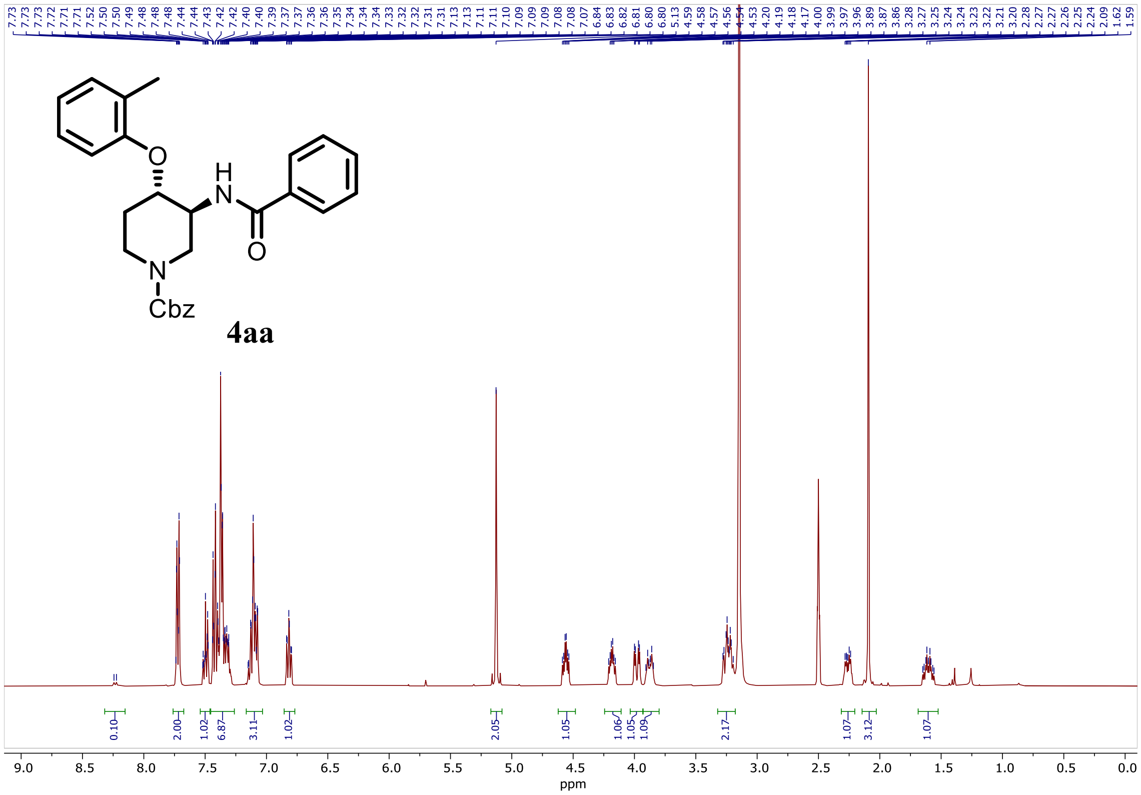

$^{13}C\{^{1}H\}$ NMR spectrum (101 MHz, DMSO-d_6, 343 K) of compound **4aa**

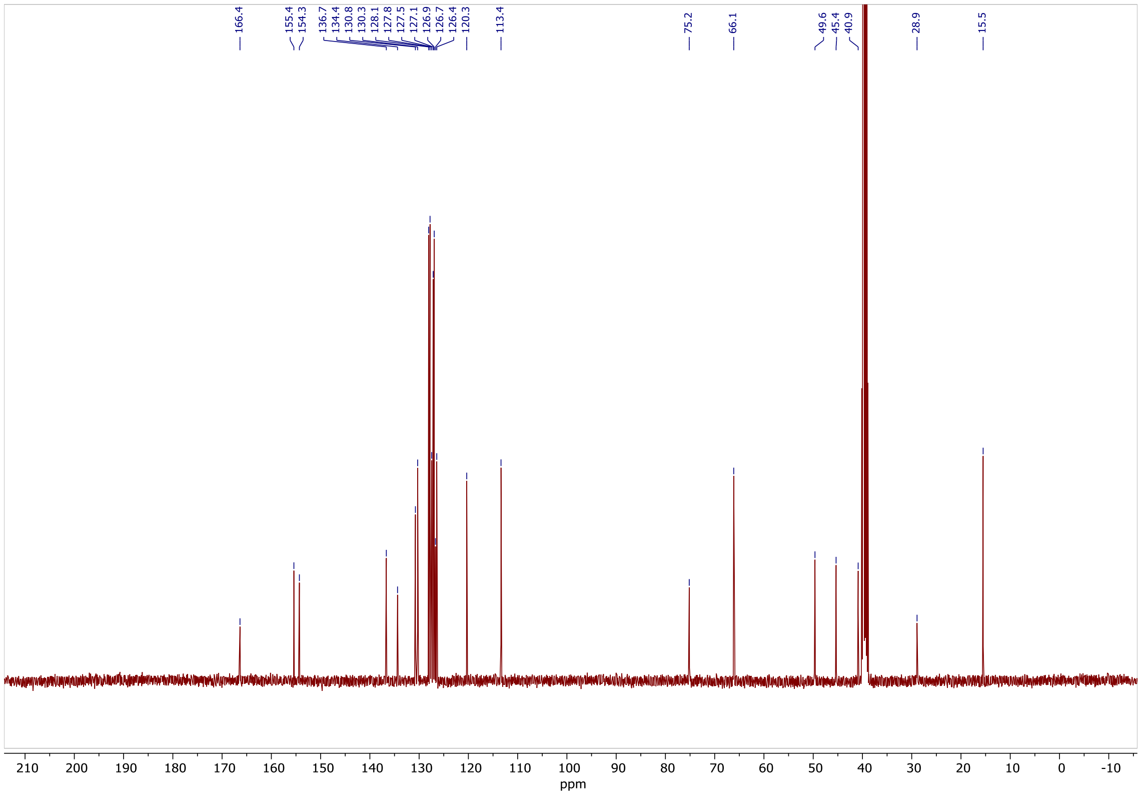

HSQC NMR spectrum (400 MHz, 101 MHz, DMSO-d_6, 343 K) of compound **4aa**

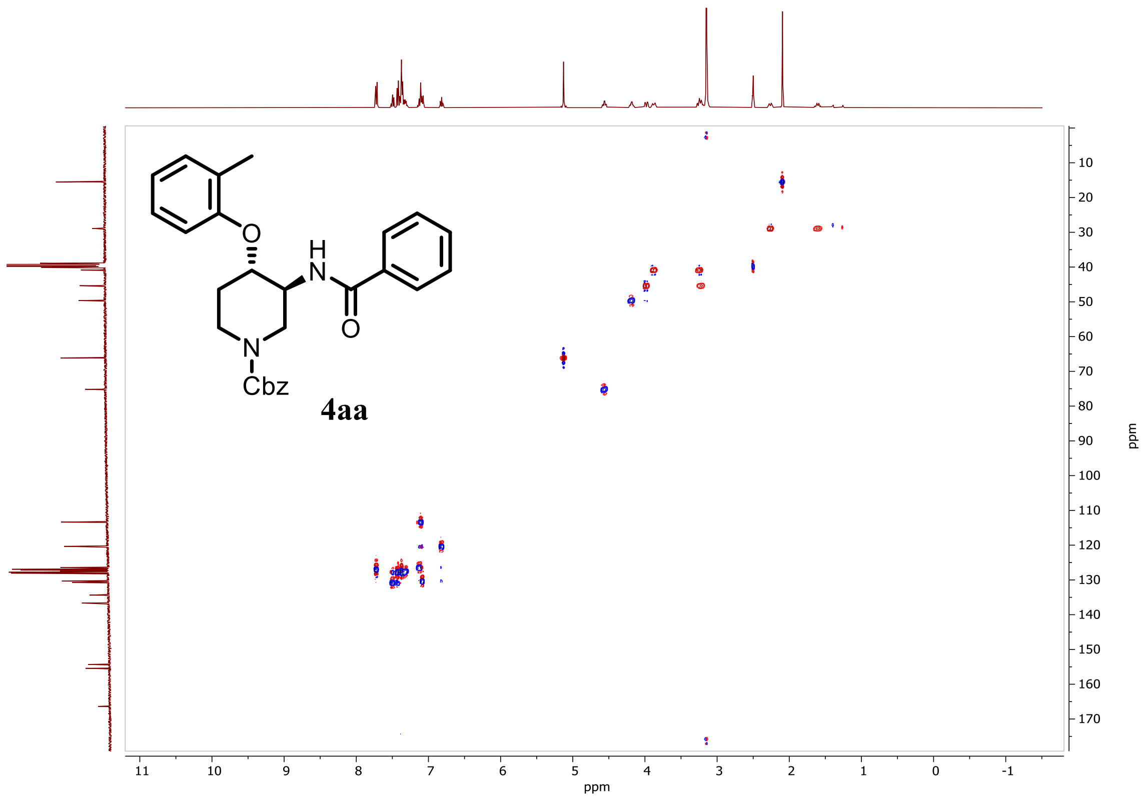

NOESY NMR spectrum (400 MHz, DMSO-d_6, 343 K) of compound **4aa**

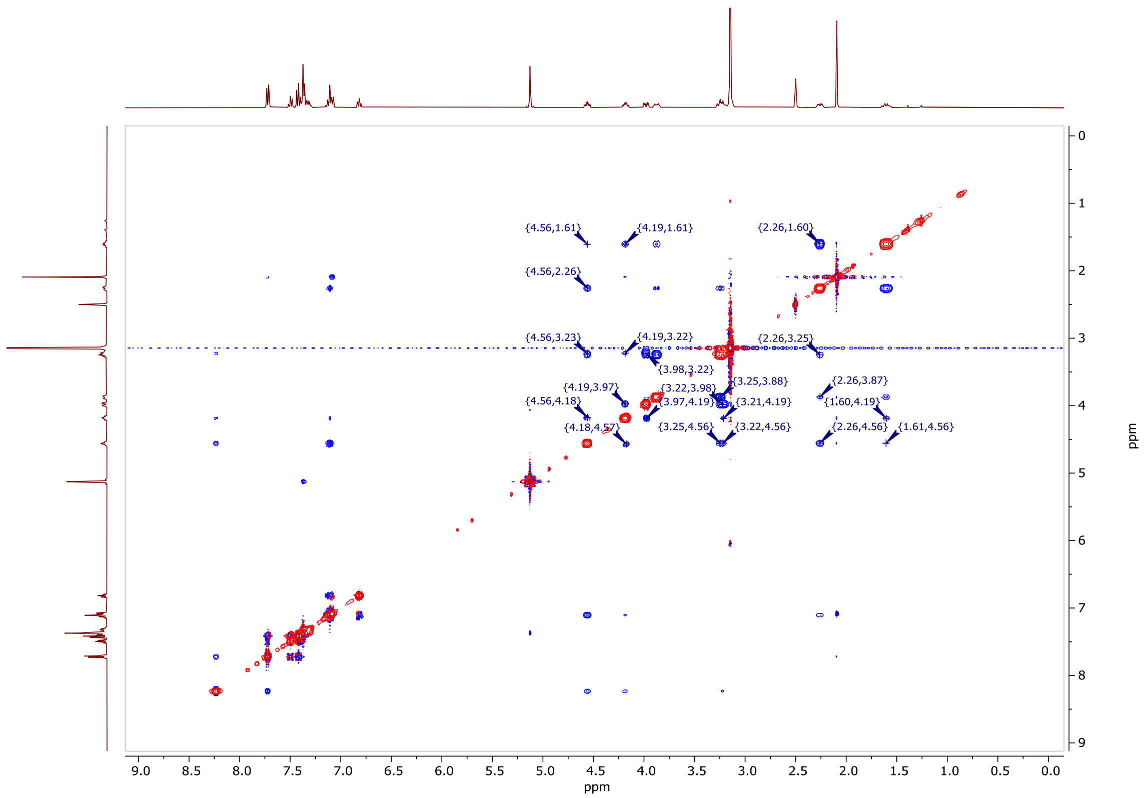

The major product, **4aa**, from the reaction of **3** with *o*-cresol was elucidated as such. The piperidine ring was assigned by analysis of HSQC and NOESY spectra. A methylene carbon observed at δ_C 45.4 was assigned as CH_2-2. The two attached protons δ_H 3.98 (dd, J = 13.2, 2.9 Hz, 1H) and δ_H 3.22 (m, 1H) both have through-space correlations to the methyne proton at δ_H 4.18 (m, 1H) attached to a nitrogen-bearing carbon at δ_c 49.6, which indicate their neighboring carbon is the benzamide-bearing carbon, CH-3. The proton at δ_C 4.18 has a through-space correlation with the methyne proton at δ_H 4.56 (m, 1H) attached to an oxygen-bearing carbon at δ_C 75.2 making this carbon the *o*-cresol-bearing C-4. The methyne CH-4 proton has through space correlations with both methylene protons, δ_H 2.26 (m, 1H) and δ_H 1.60 (m, 1H), attached to the carbon at δ_C 28.9 making this methylene carbon CH_2-5. The reaming methylene carbon at δ_C 40.9 is the piperidine-nitrogen-bearing carbon C-6. This assignment can also be confirmed by through-space correlations with both protons from CH_2-5 to the methylene protons attached to CH_2-6 at δ_H 3.88 (m, 1H) and δ_H 3.24 (m, 1H).

The relative stereochemistry for the piperidine was determined by analysis of the through-space NOESY correlations. CH-4, the methyne proton attached to the *o*-cresol-bearing carbon, has 1,3-diaxial through-space correlations with the protons at δ_H 3.22 and δ_H 3.24 indicating these protons have to be on the same side of the ring. CH-3, the methyne proton attached the benzamide-bearing carbon, has a 1,3-diaxial through-space correlation with the proton at δ_H 1.60. These through-space correlations all indicate the relative orientation of the benzamide and *o*-cresol moieties are *trans* (Figure S2).

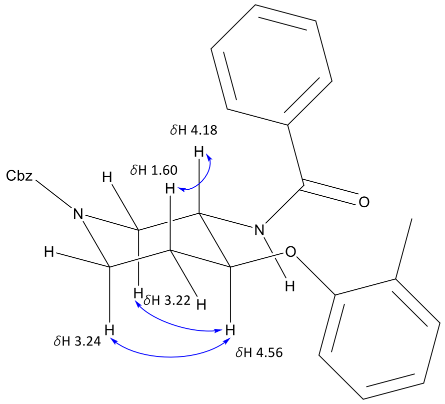

Figure S2. Key NOESY correlations for **4aa**.

^{1}H NMR spectrum (400 MHz, DMSO-d_6, 343 K) of compound **4ab**

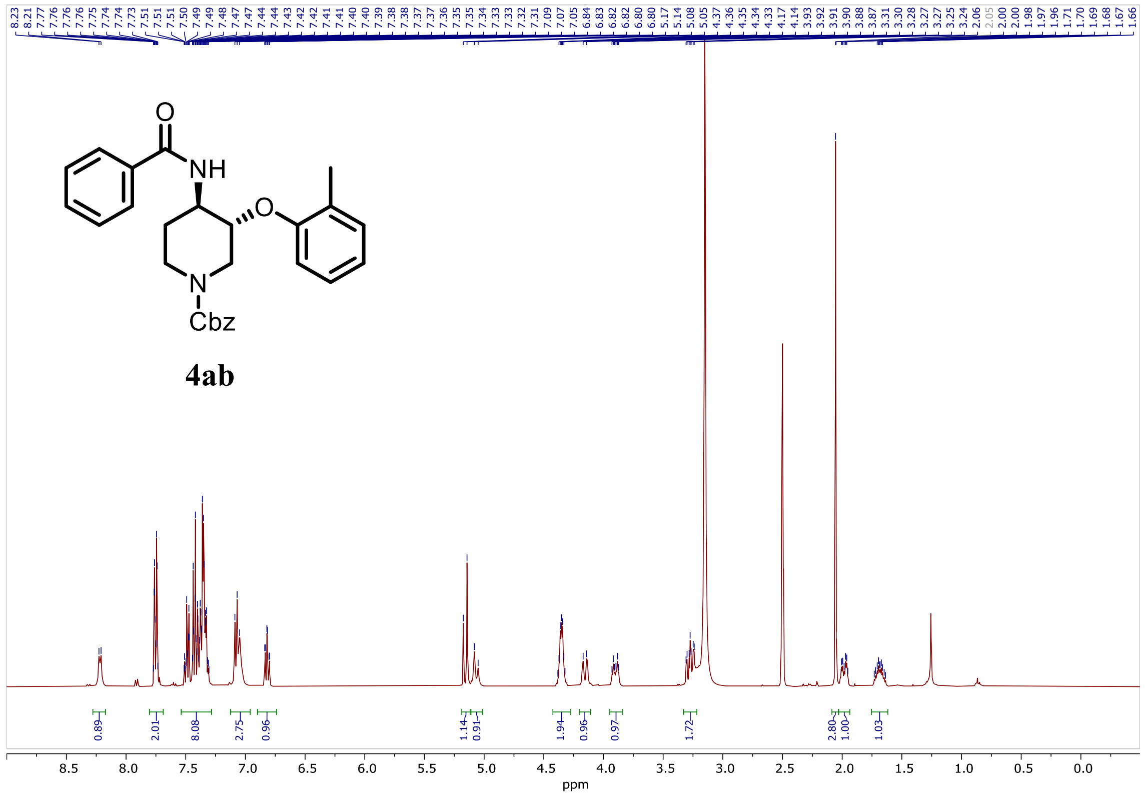

^{13}C{^{1}H} NMR spectrum (101 MHz, DMSO-d_6, 343 K) of compound **4ab**

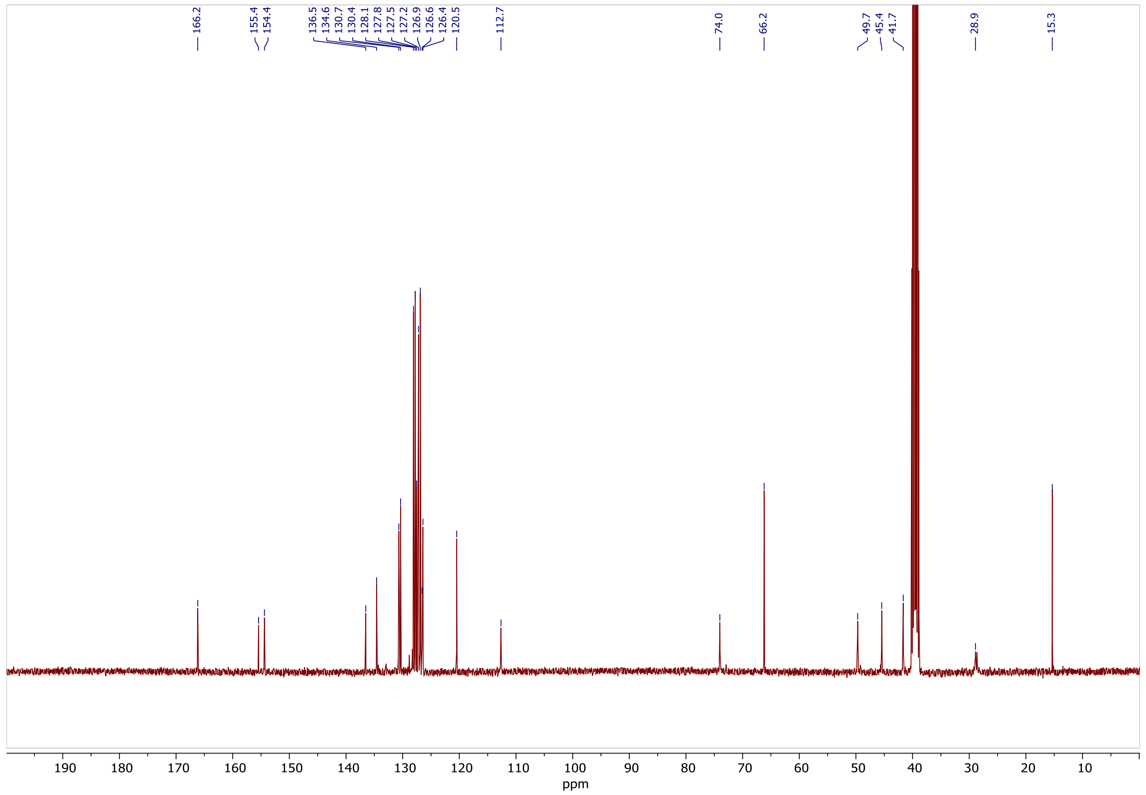

^{1}H NMR spectrum (400 MHz, $CDCl_3$, 298 K) of compound **e**

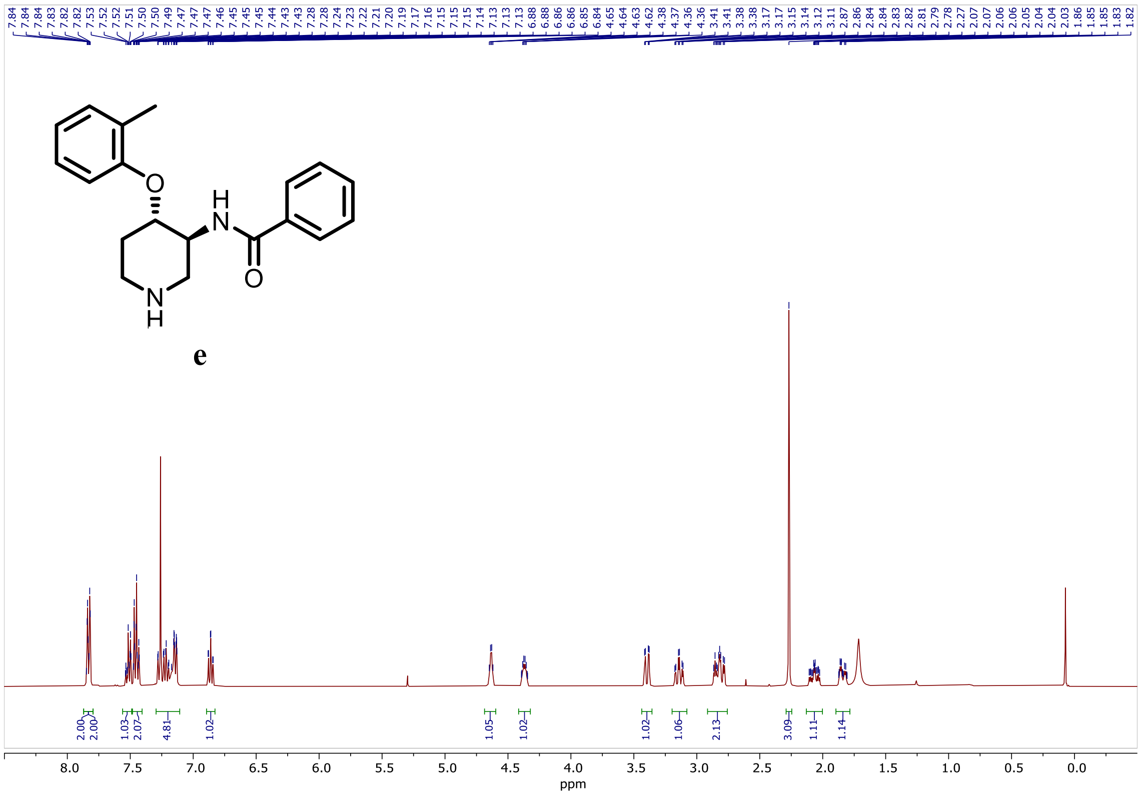

^{13}C{^{1}H} NMR spectrum (101 MHz, $CDCl_3$, 298 K) of compound **e**

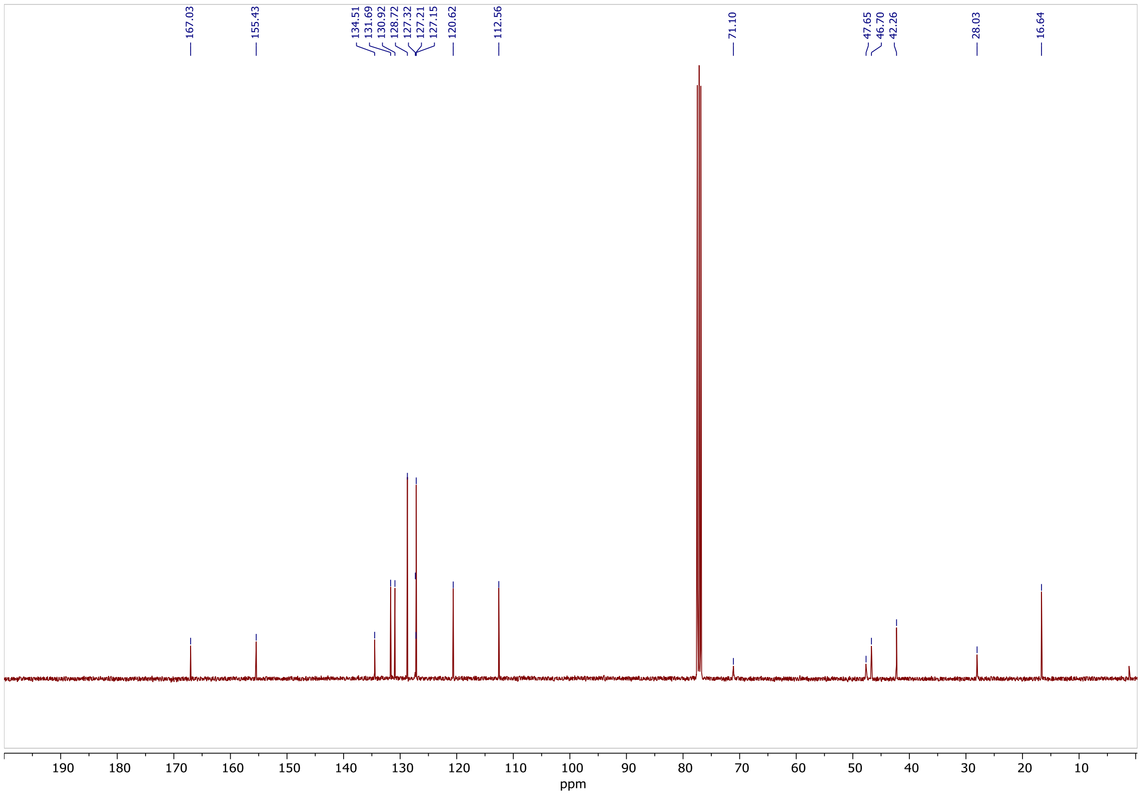

^{1}H NMR spectrum (400 MHz, MeOD, 298 K) of compound **4ba**

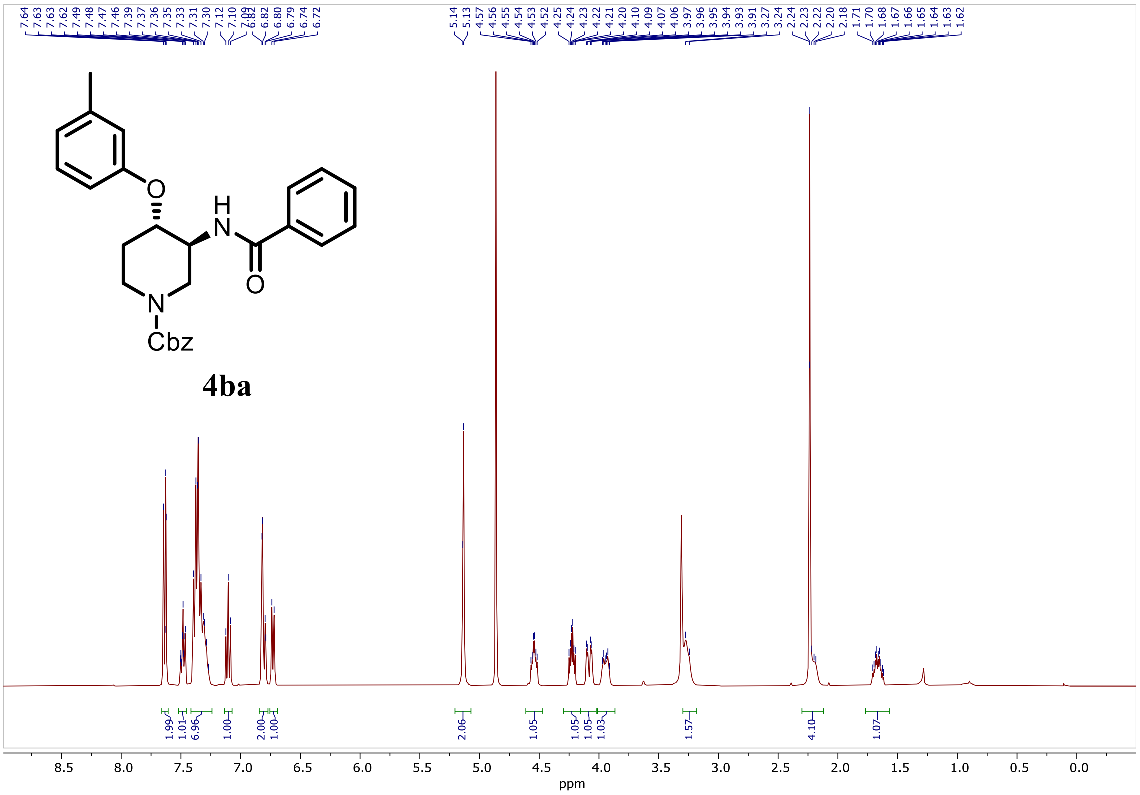

^{13}C{^{1}H} NMR spectrum (101 MHz, MeOD, 298 K) of compound **4ba**

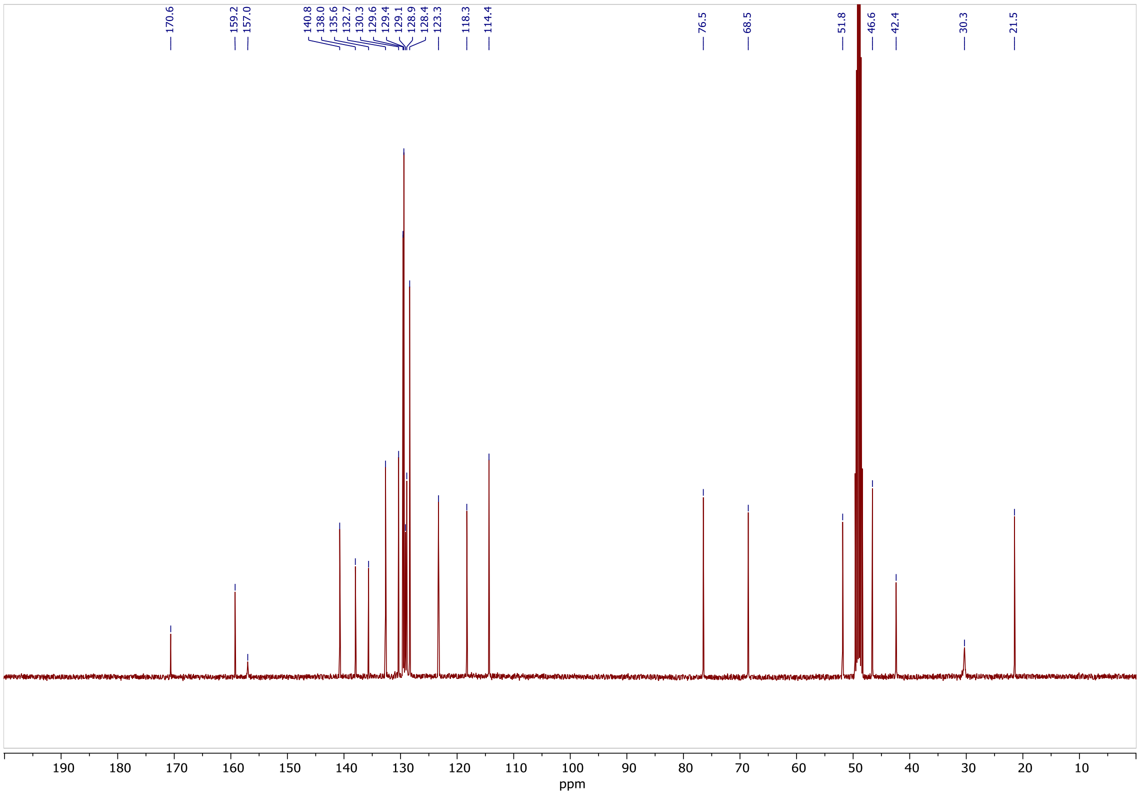

^{1}H NMR spectrum (400 MHz, DMSO-d_6, 343 K) of compound **4bb**

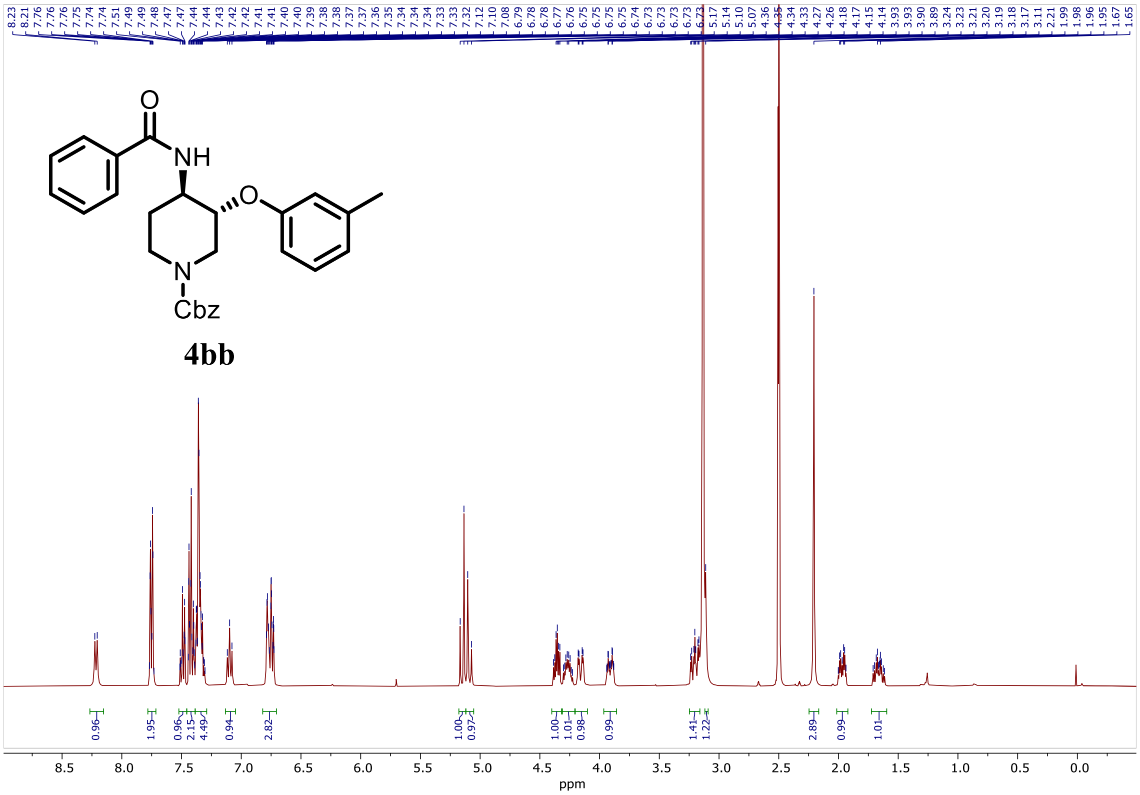

^{13}C{^{1}H} NMR spectrum (101 MHz, DMSO-d_6, 343 K) of compound **4bb**

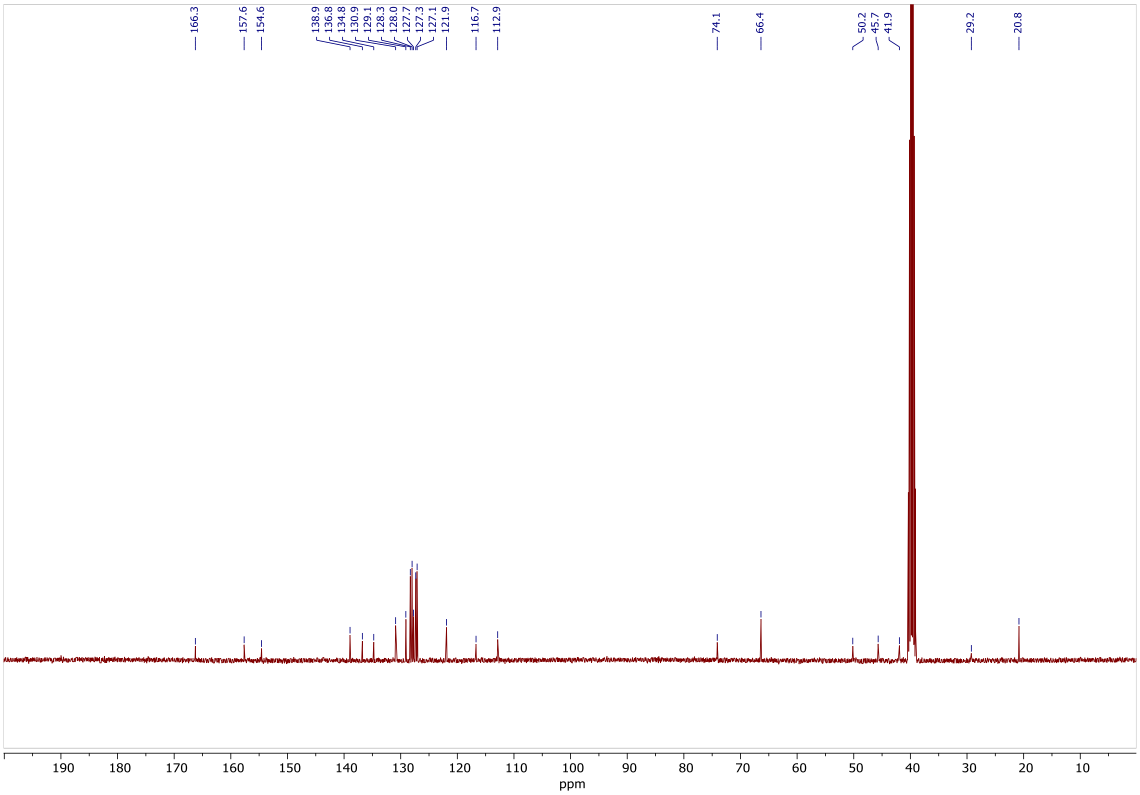

^{1}H NMR spectrum (400 MHz, MeOD, 298 K) of compound **4ca**

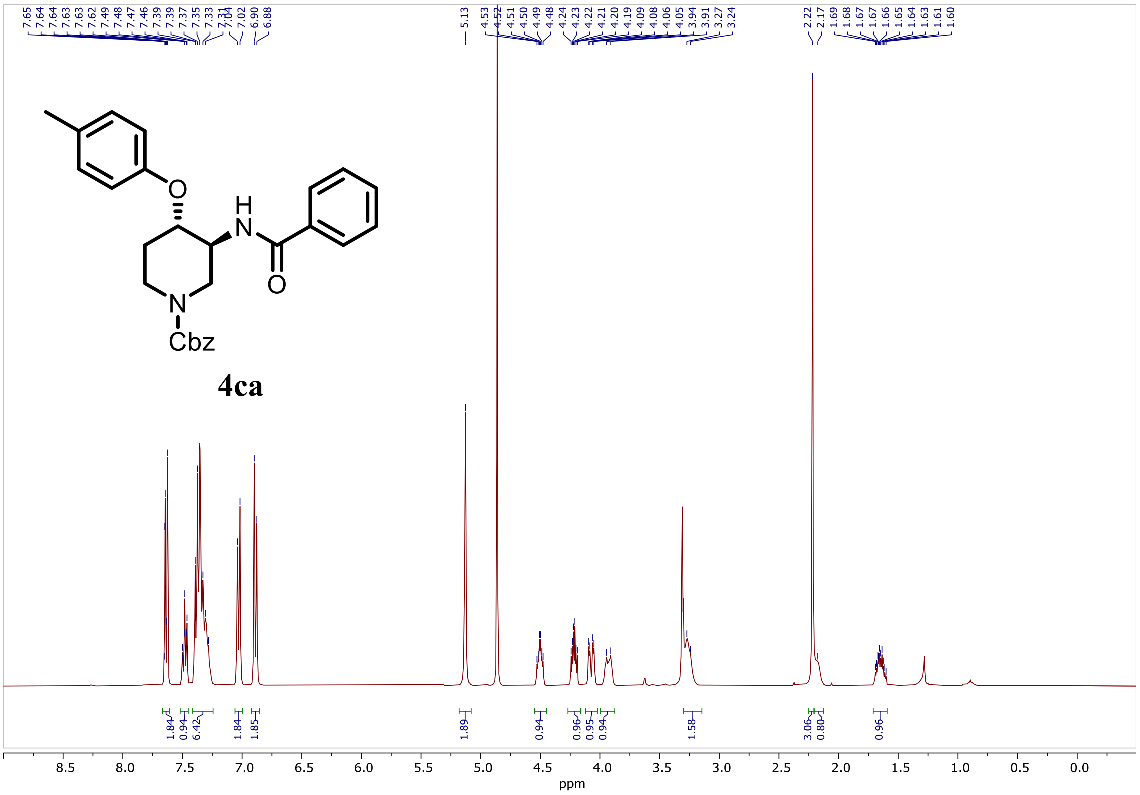

$^{13}C\{^{1}H\}$ NMR spectrum (101 MHz, MeOD, 298 K) of compound **4ca**

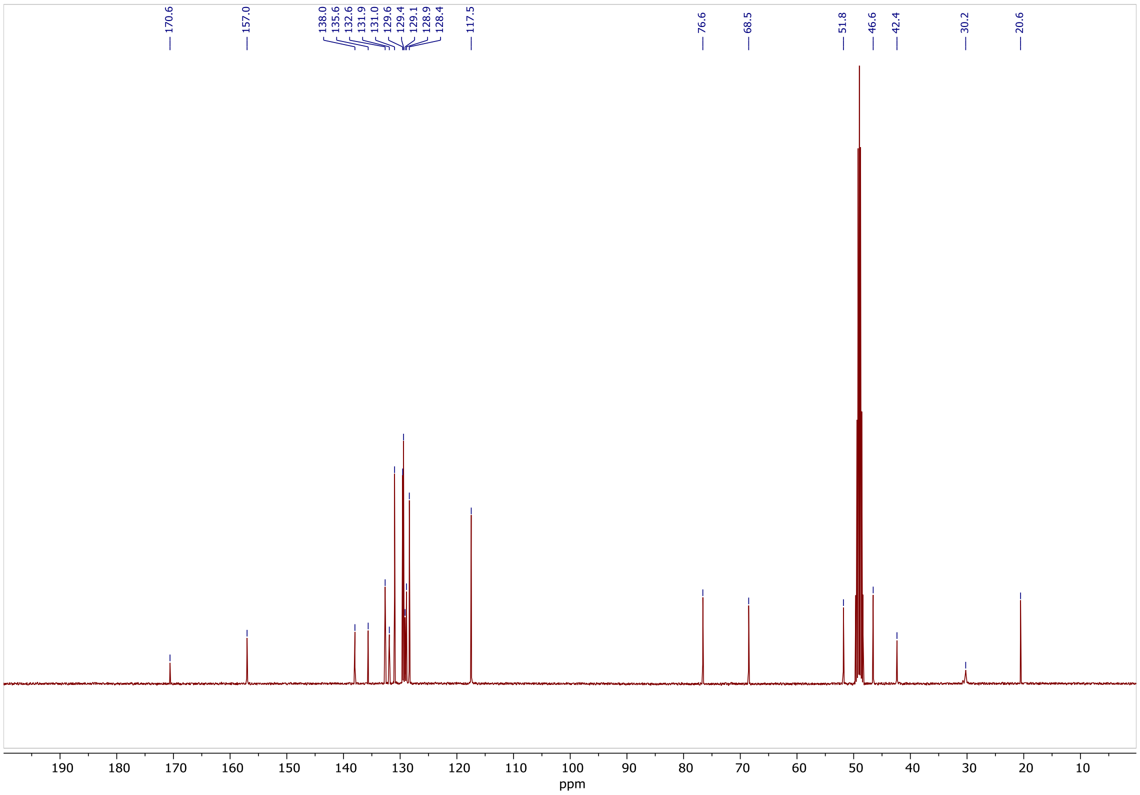

^{1}H NMR spectrum (400 MHz, DMSO-d_6, 343 K) of compound **4cb**

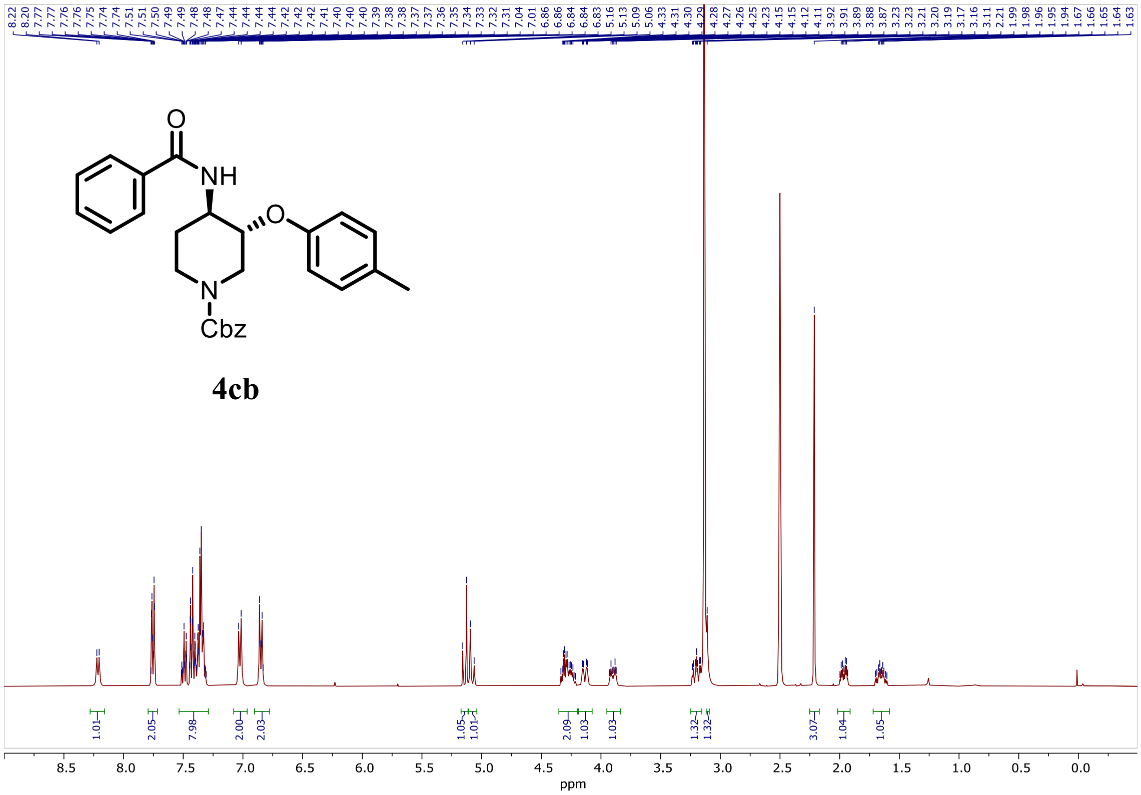

$^{13}C\{^{1}H\}$ NMR spectrum (101 MHz, DMSO-d_6, 343 K) of compound **4cb**

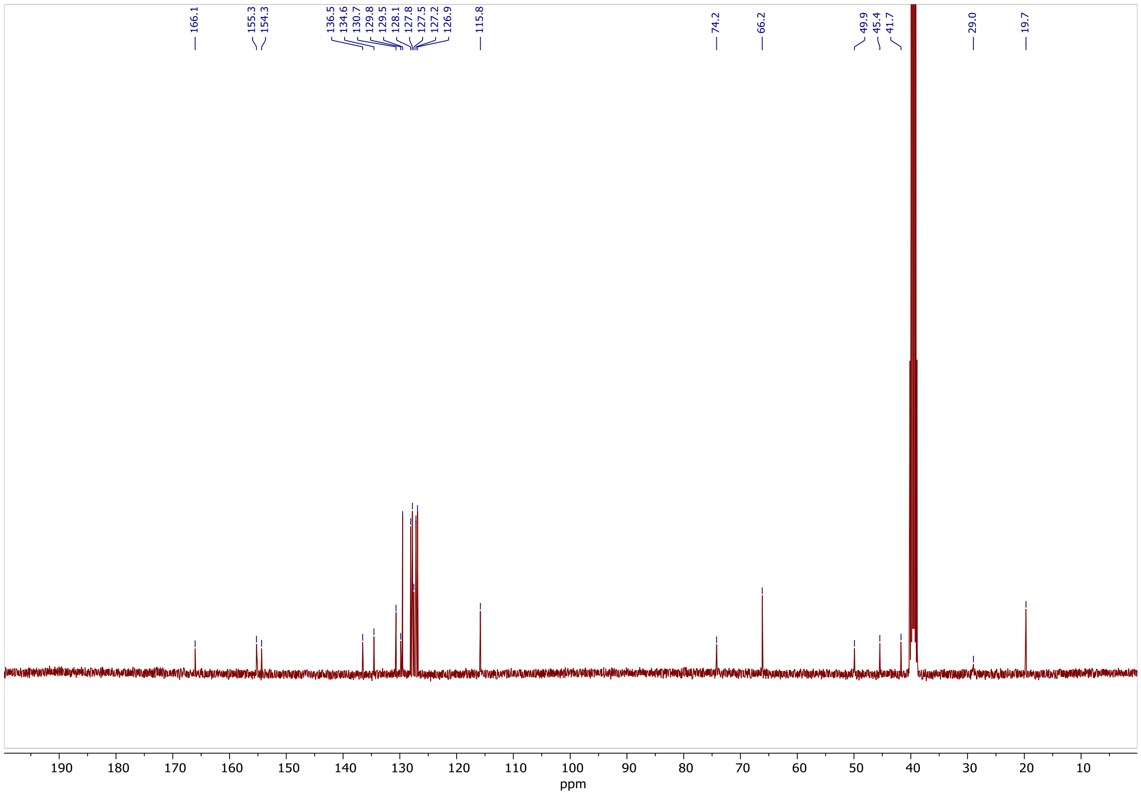

^{1}H NMR spectrum (400 MHz, MeOD, 298 K) of compound **4da**

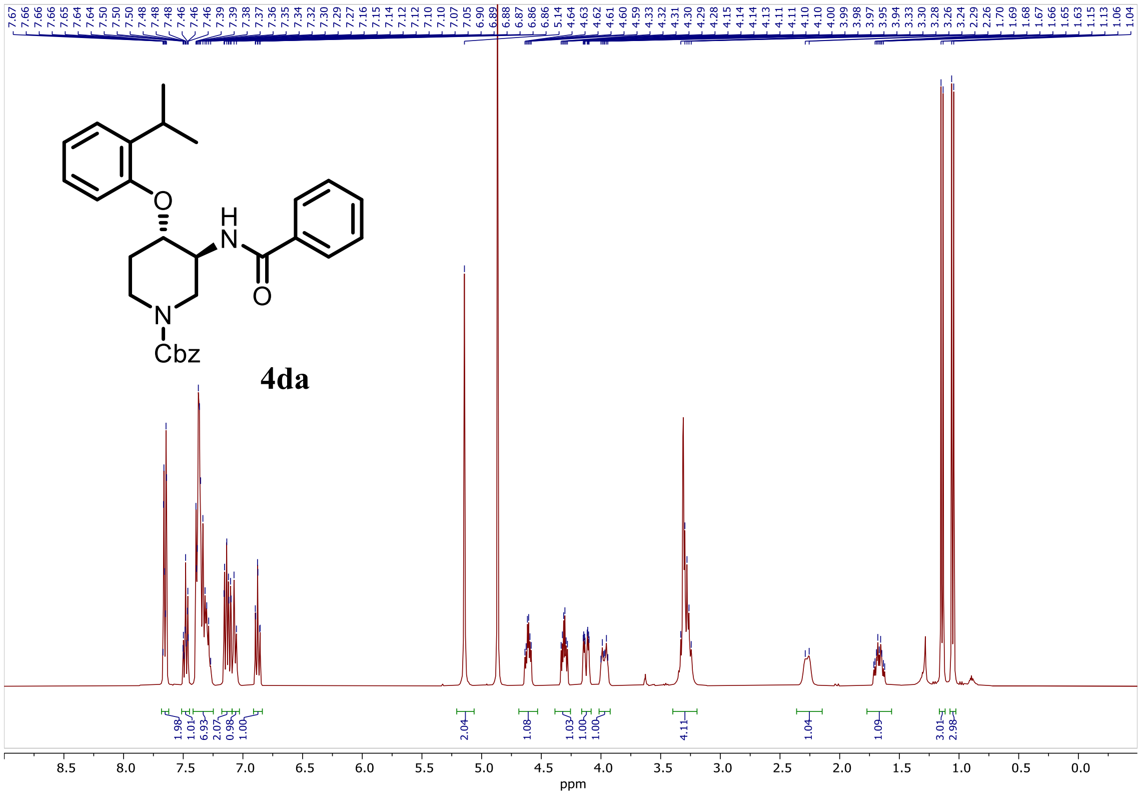

^{13}C{^{1}H} NMR spectrum (101 MHz, MeOD, 298 K) of compound **4da**

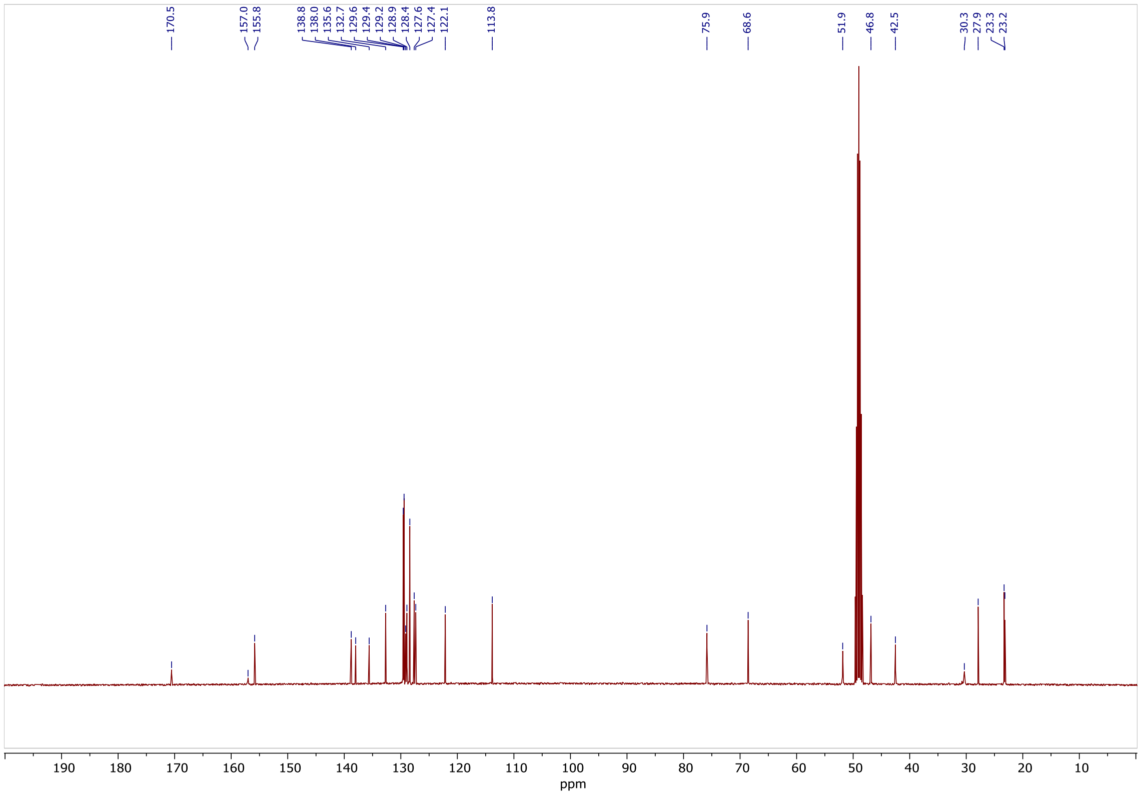

^{1}H NMR spectrum (400 MHz, DMSO-d_6, 343 K) of compound **4db**

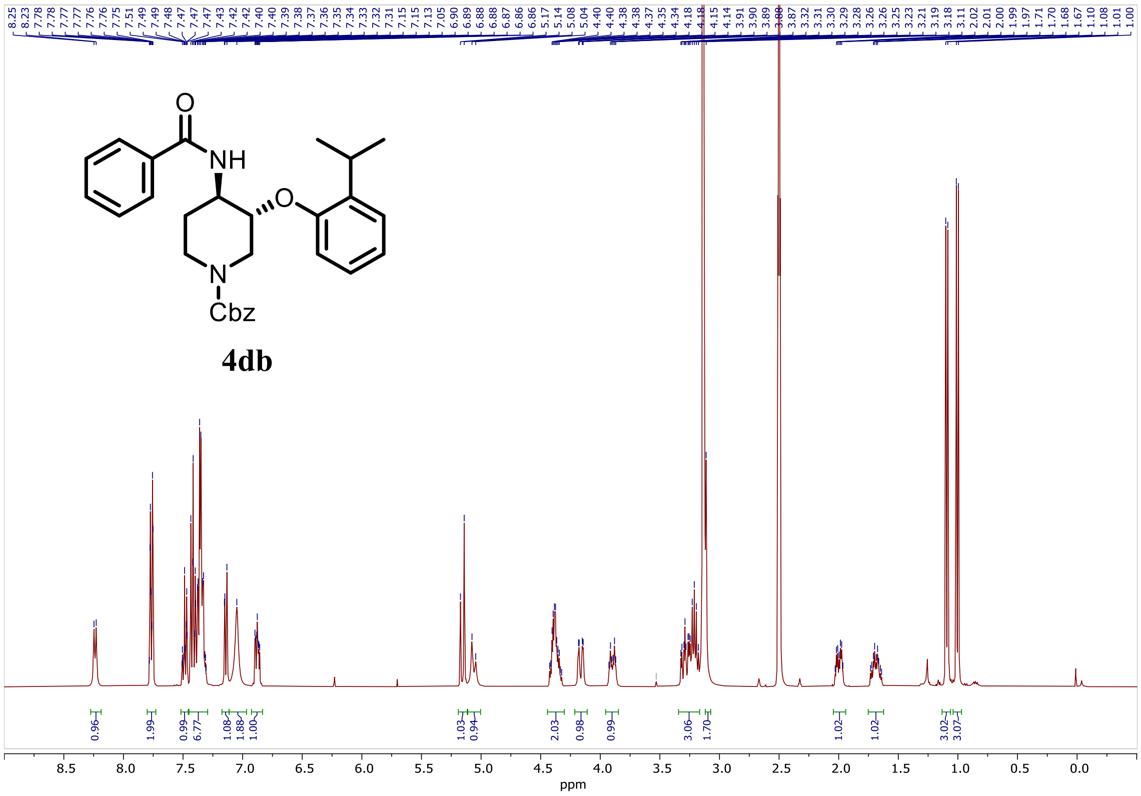

$^{13}C\{^{1}H\}$ NMR spectrum (101 MHz, DMSO-d_6, 343 K) of compound **4db**

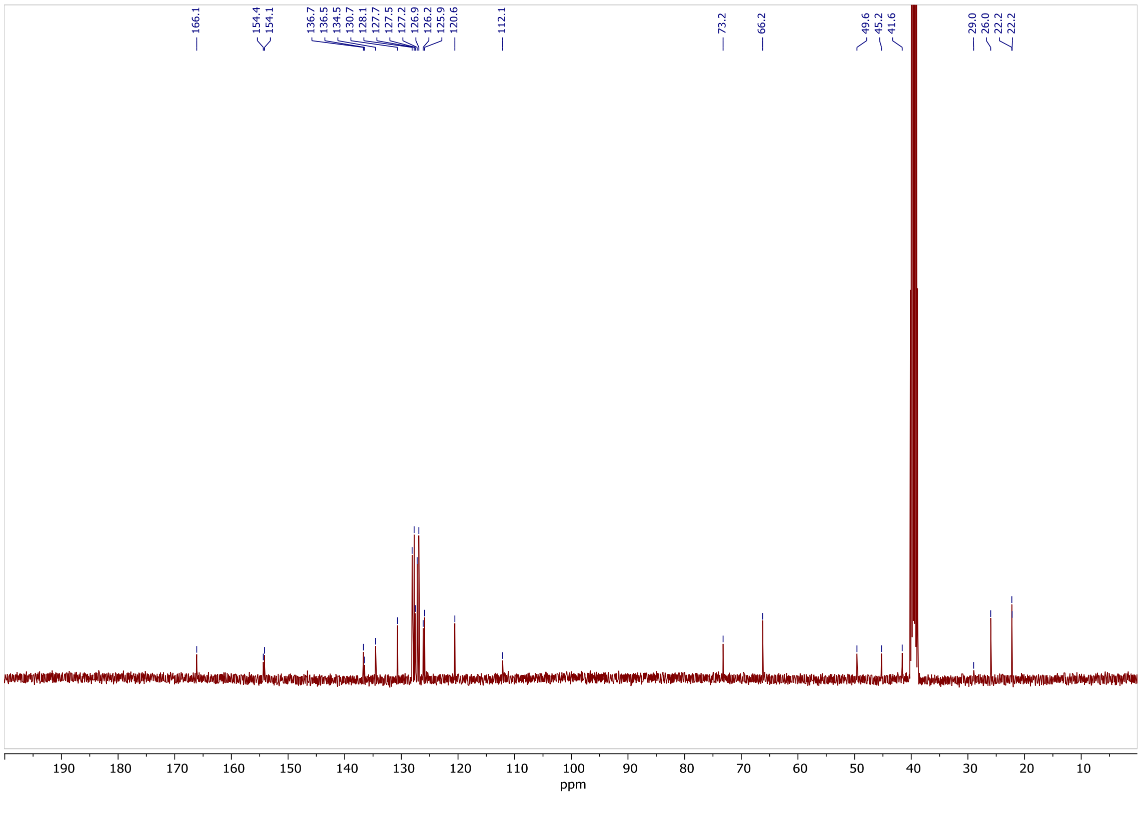

^{1}H NMR spectrum (400 MHz, DMSO-d_6, 343 K) of compound **4ea**

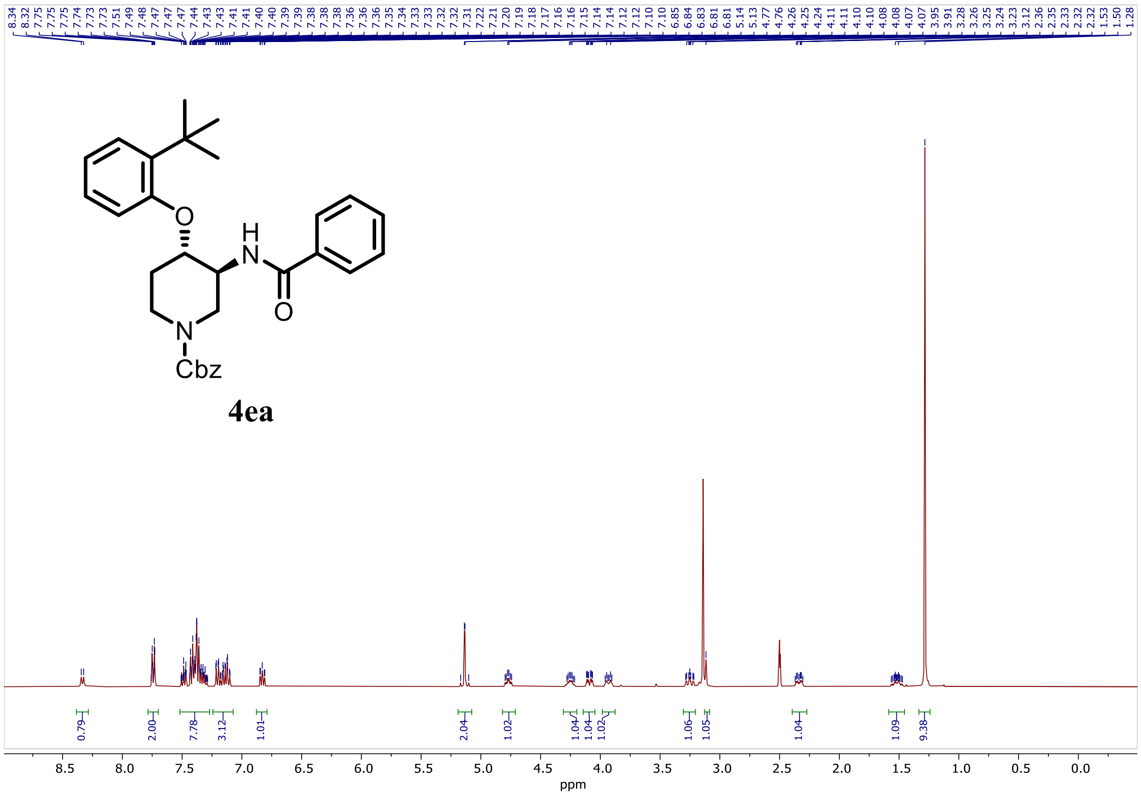

^{13}C{^{1}H} NMR spectrum (101 MHz, DMSO-d_6, 343 K) of compound **4ea**

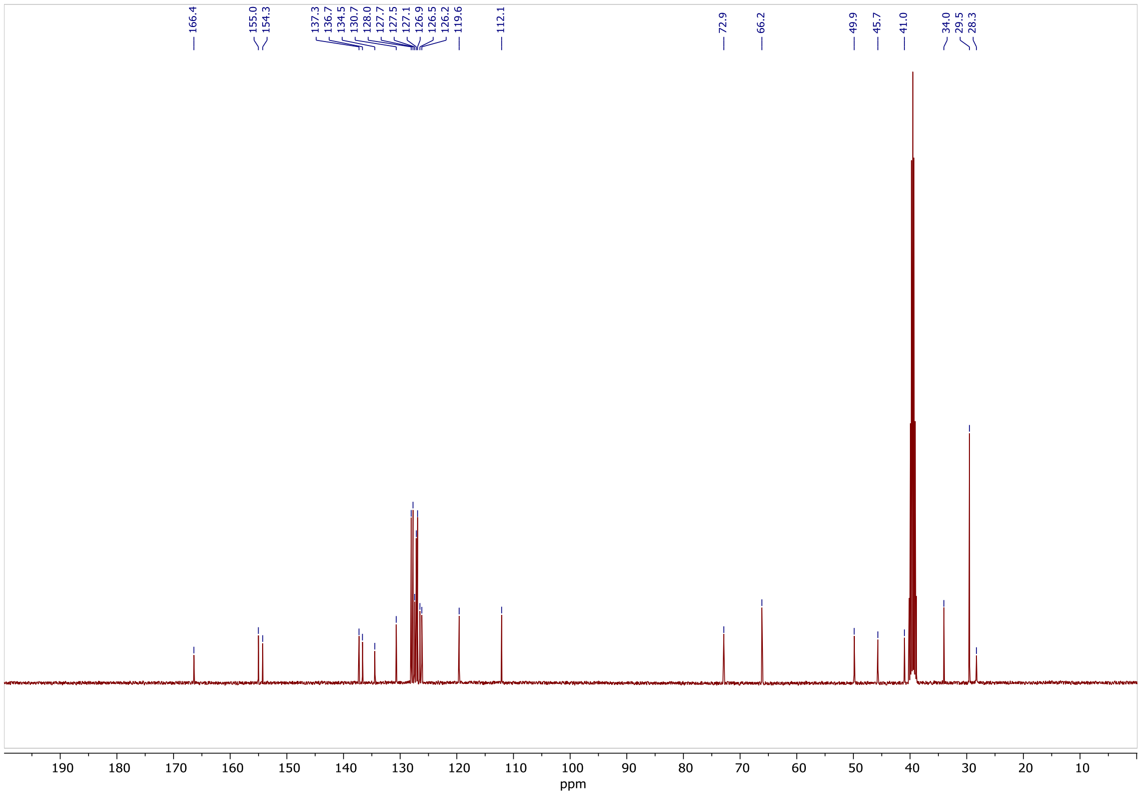

^{1}H NMR spectrum (400 MHz, DMSO-d_6, 343 K) of compound **4eb**

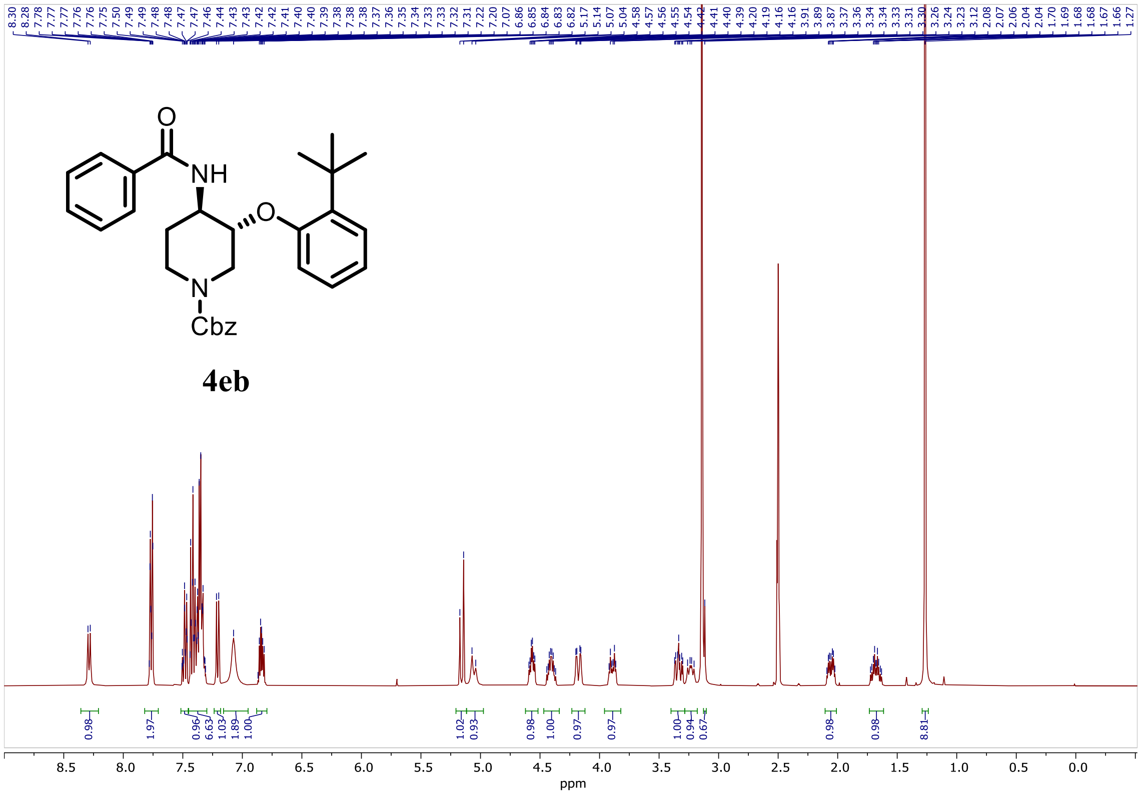

^{13}C{^{1}H} NMR spectrum (101 MHz, DMSO-d_6, 343 K) of compound **4eb**

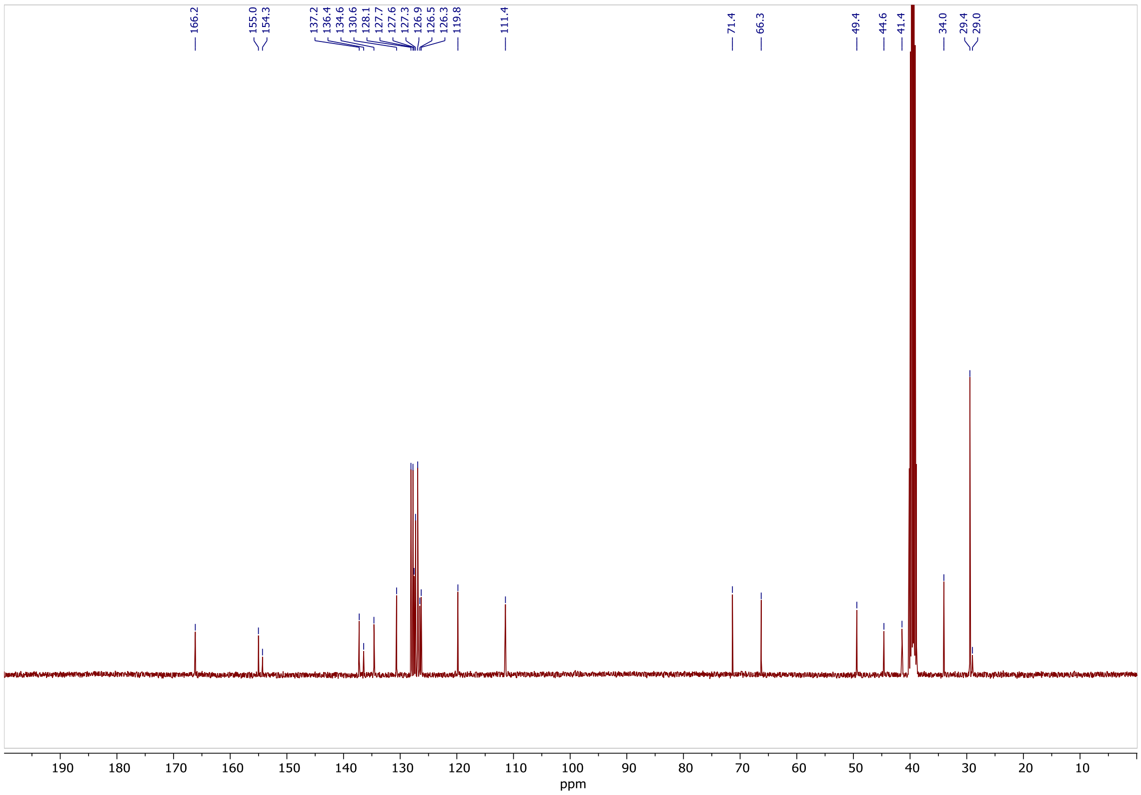

^{1}H NMR spectrum (400 MHz, DMSO-d_6, 298 K) of compound **4fa**

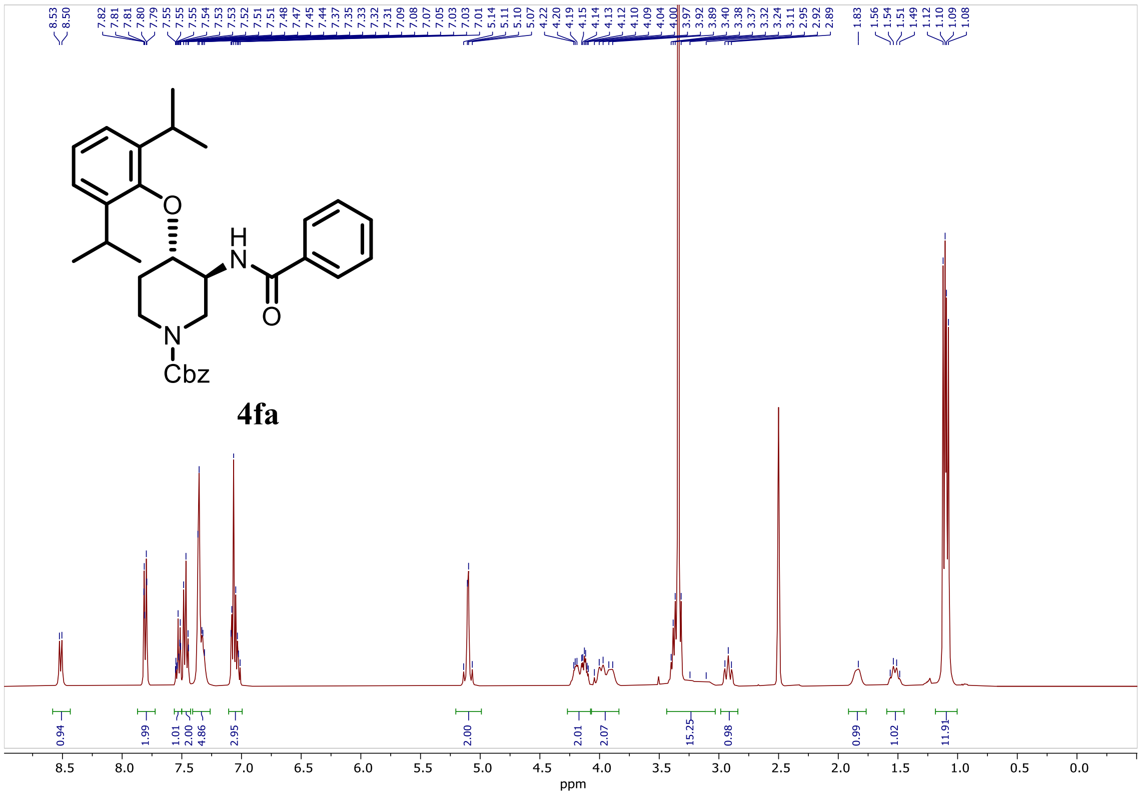

$^{13}C\{^{1}H\}$ NMR spectrum (101 MHz, DMSO-d_6, 298 K) of compound **4fa**

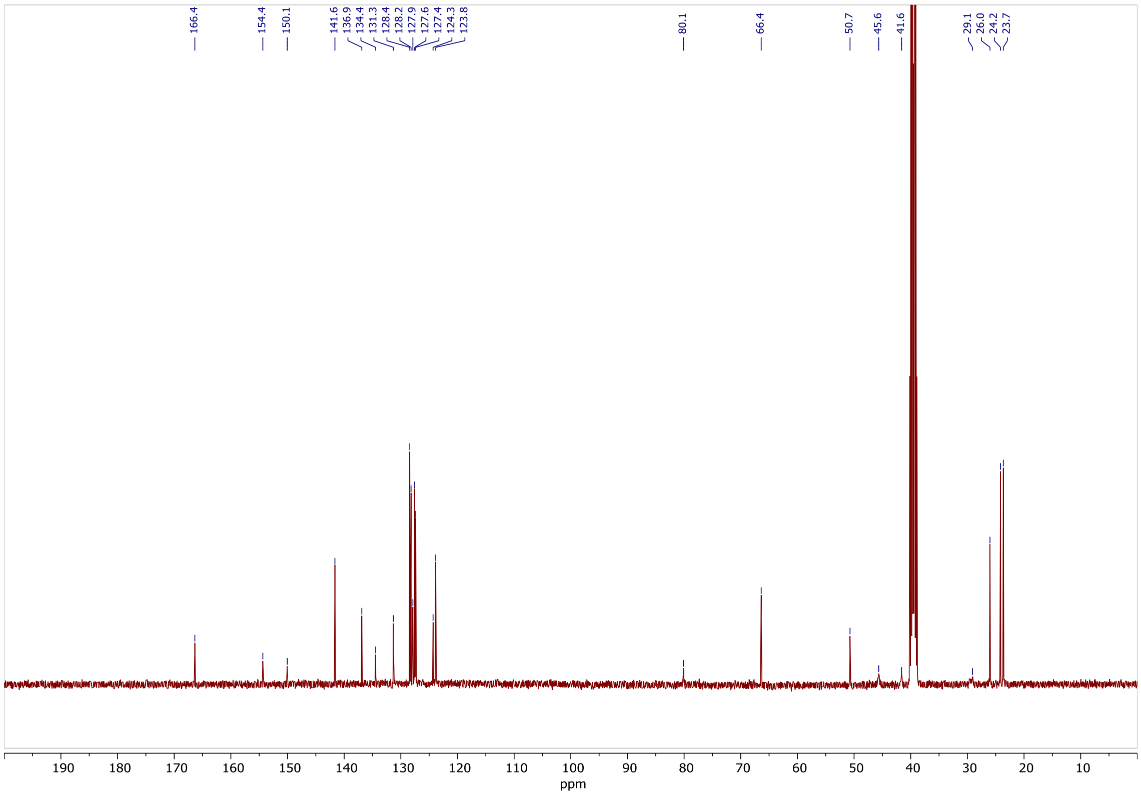

^{1}H NMR spectrum (400 MHz, DMSO-d_6, 343 K) of compound **4fb**

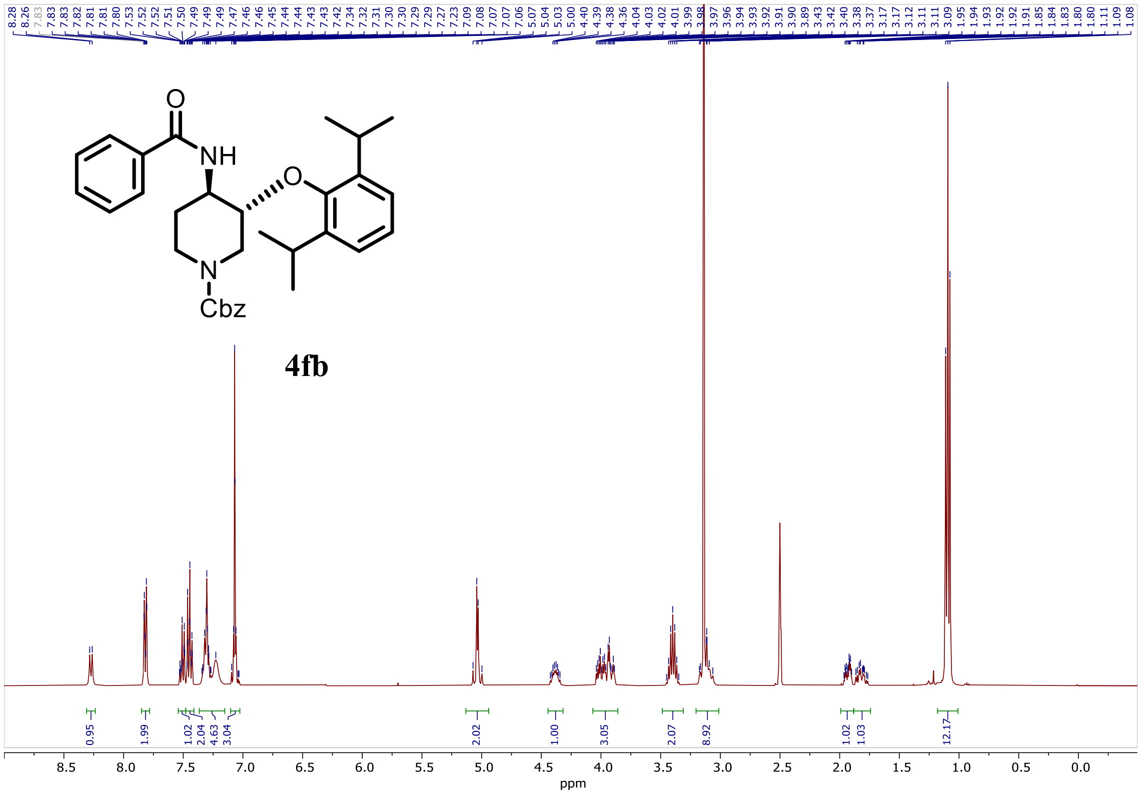

$^{13}C\{^{1}H\}$ NMR spectrum (101 MHz, DMSO-d_6, 343 K) of compound **4fb**

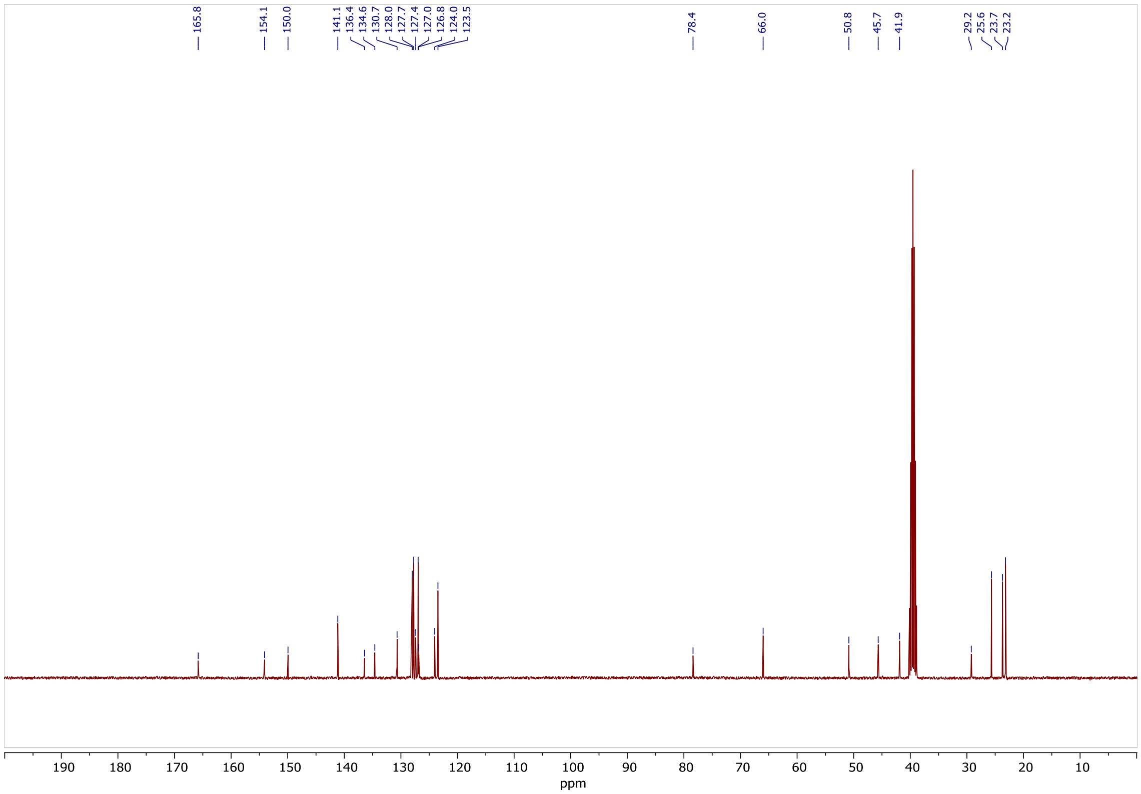

^{1}H NMR spectrum (400 MHz, DMSO-d_6, 343 K) of compound **4ga**

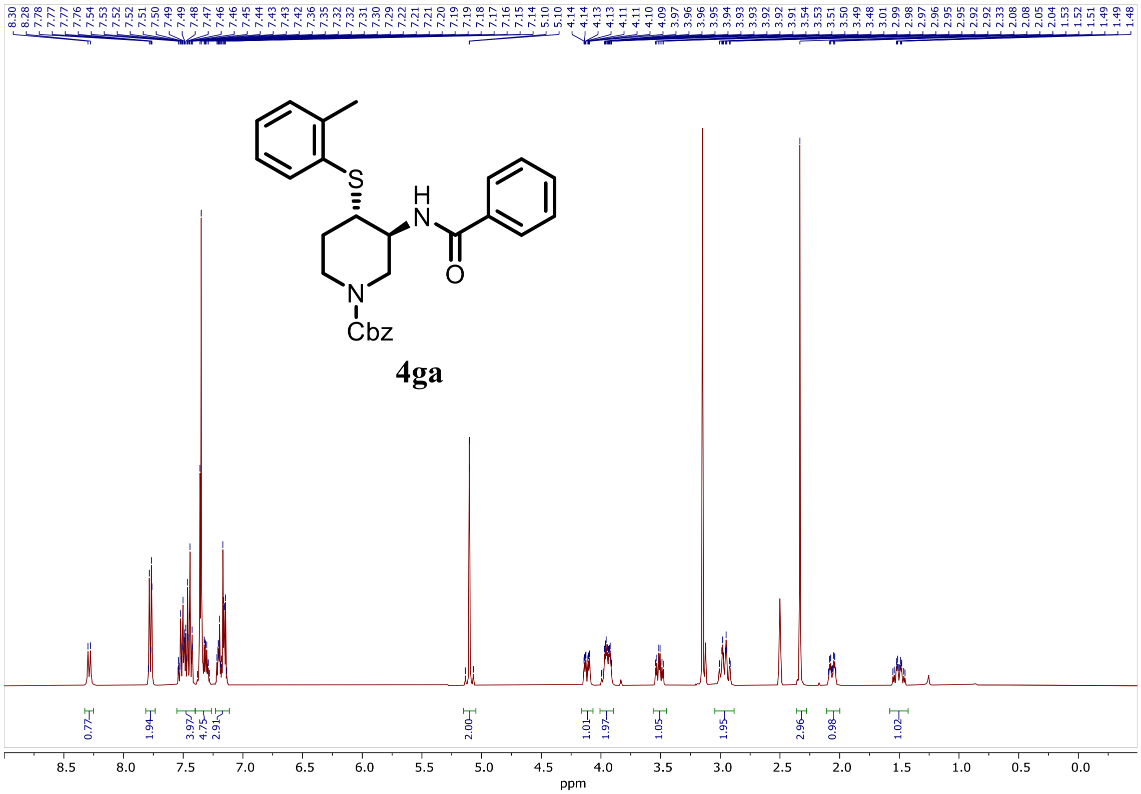

^{13}C{^{1}H} NMR spectrum (101 MHz, DMSO-d_6, 343 K) of compound **4ga**

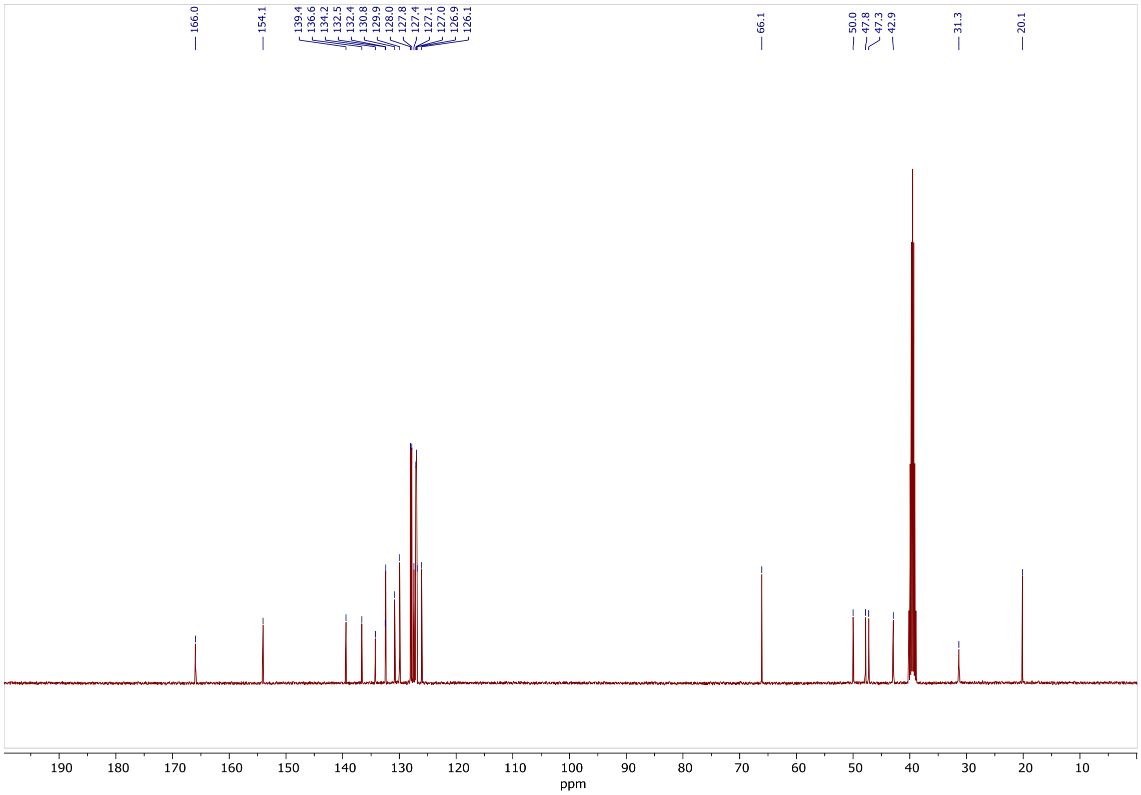

^{1}H NMR spectrum (400 MHz, DMSO-d_6, 343 K) of compound **4gb**

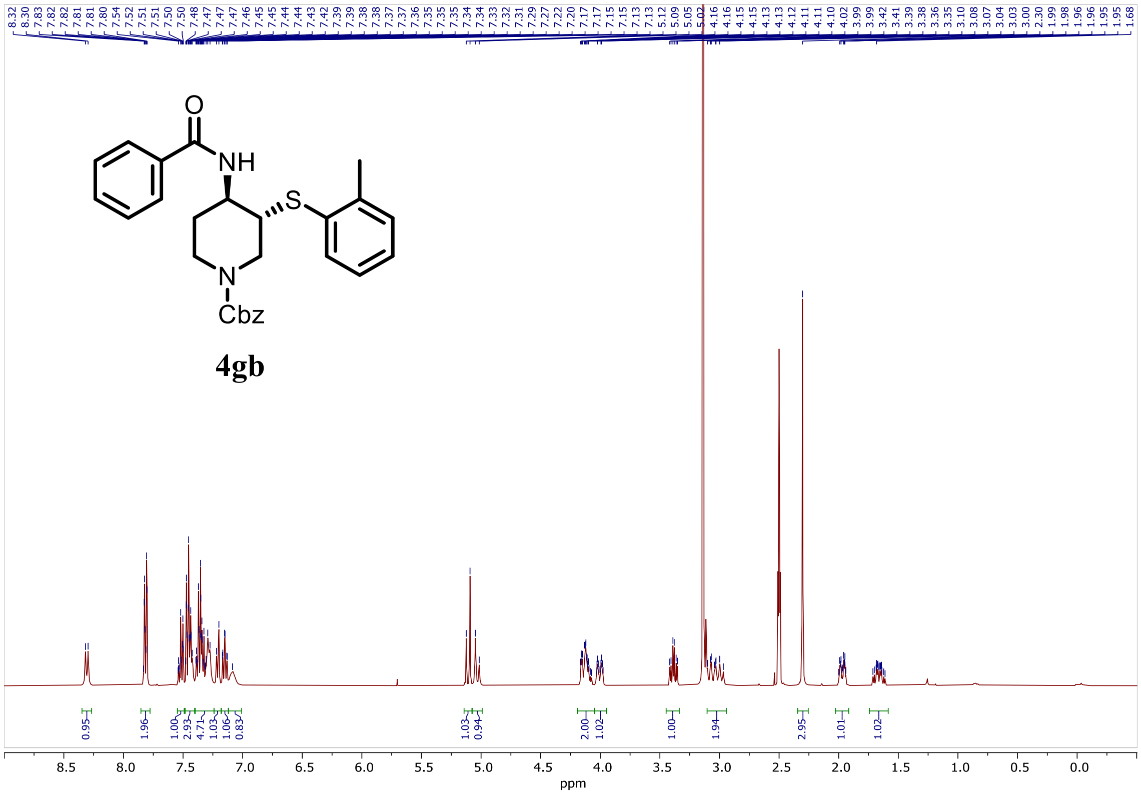

^{13}C{^{1}H} NMR spectrum (101 MHz, DMSO-d_6, 343 K) of compound **4gb**

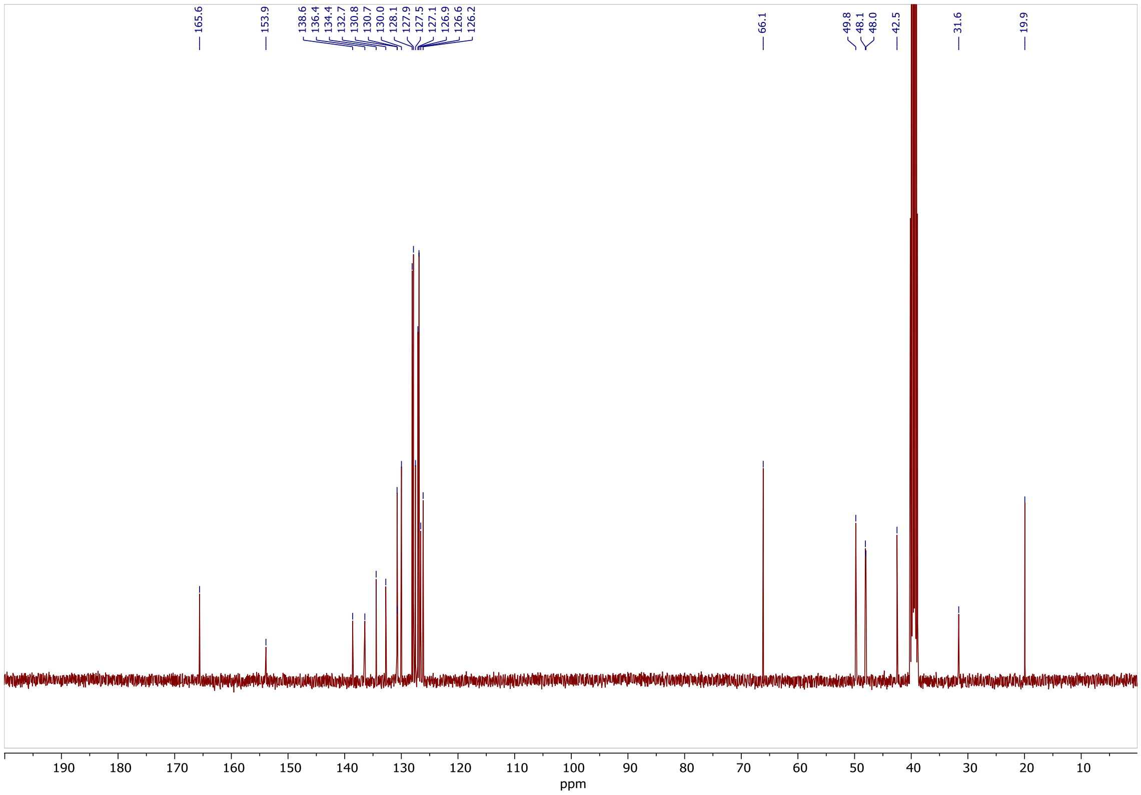

^{1}H NMR spectrum (400 MHz, MeOD, 298 K) of compound **4ha**

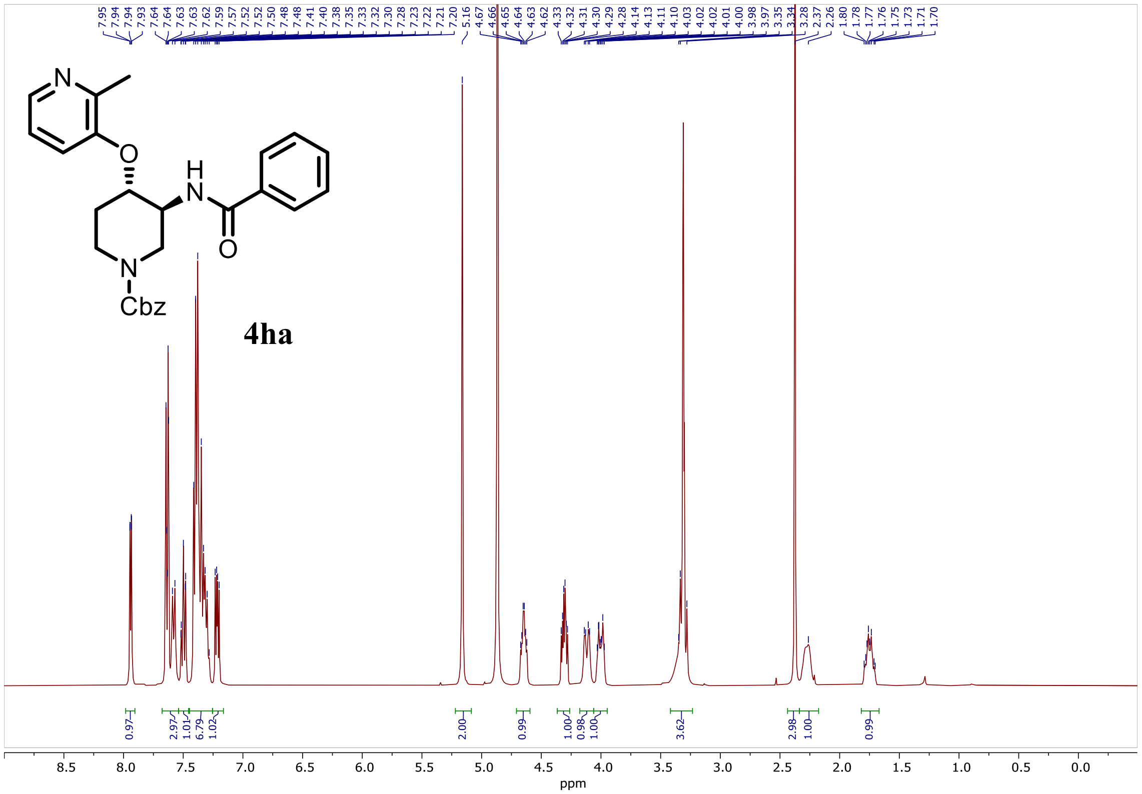

$^{13}C\{^{1}H\}$ NMR spectrum (101 MHz, MeOD, 298 K) of compound **4ha**

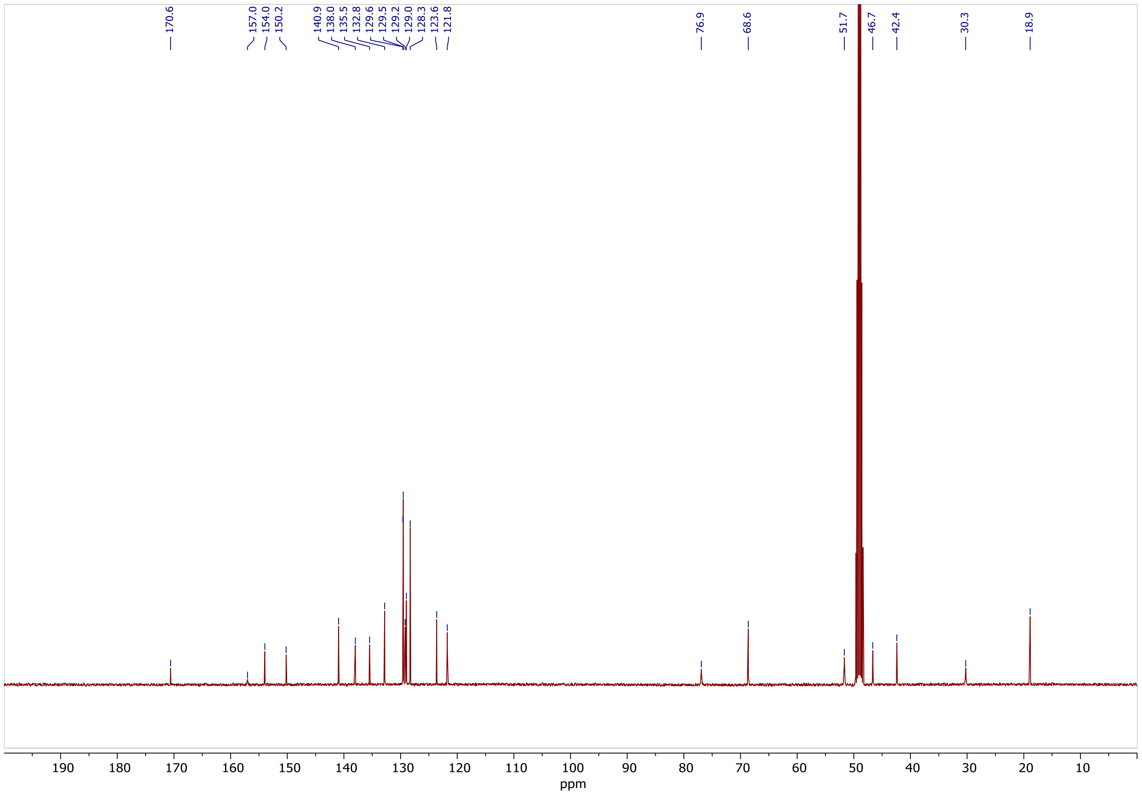

^{1}H NMR spectrum (400 MHz, DMSO-d_6, 343 K) of compound **4hb**

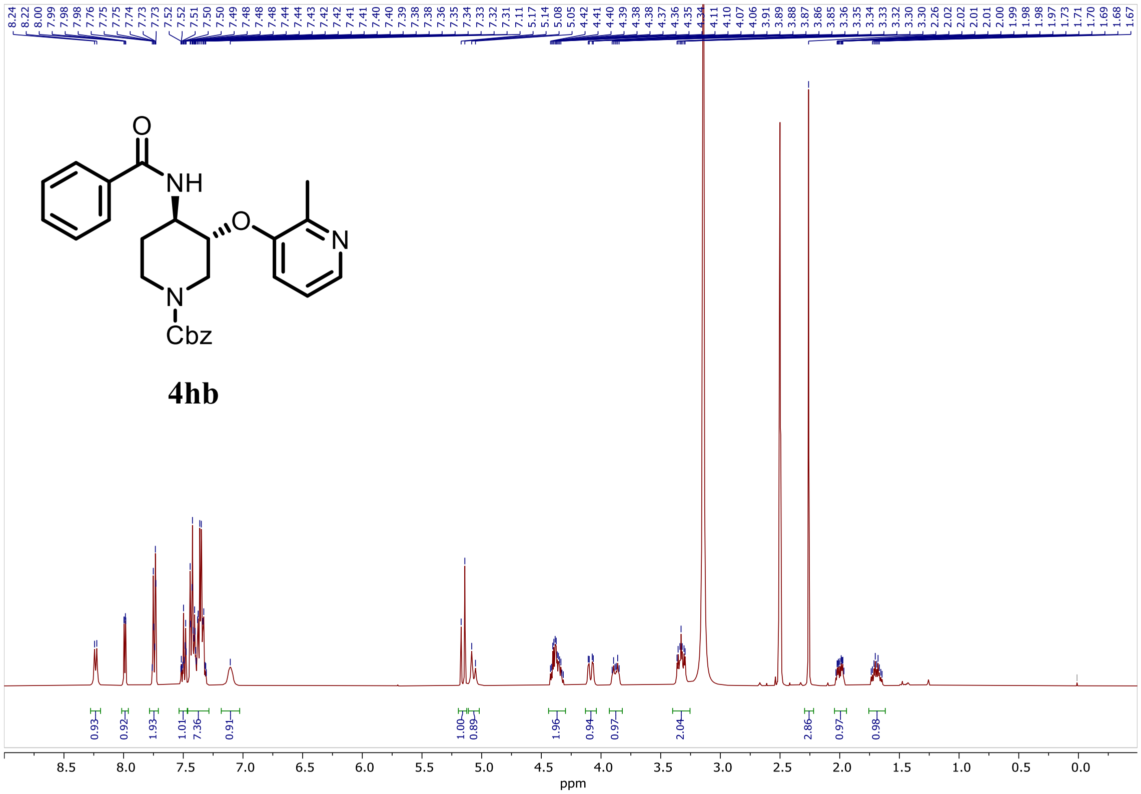

^{13}C{^{1}H} NMR spectrum (101 MHz, DMSO-d_6, 343 K) of compound **4hb**

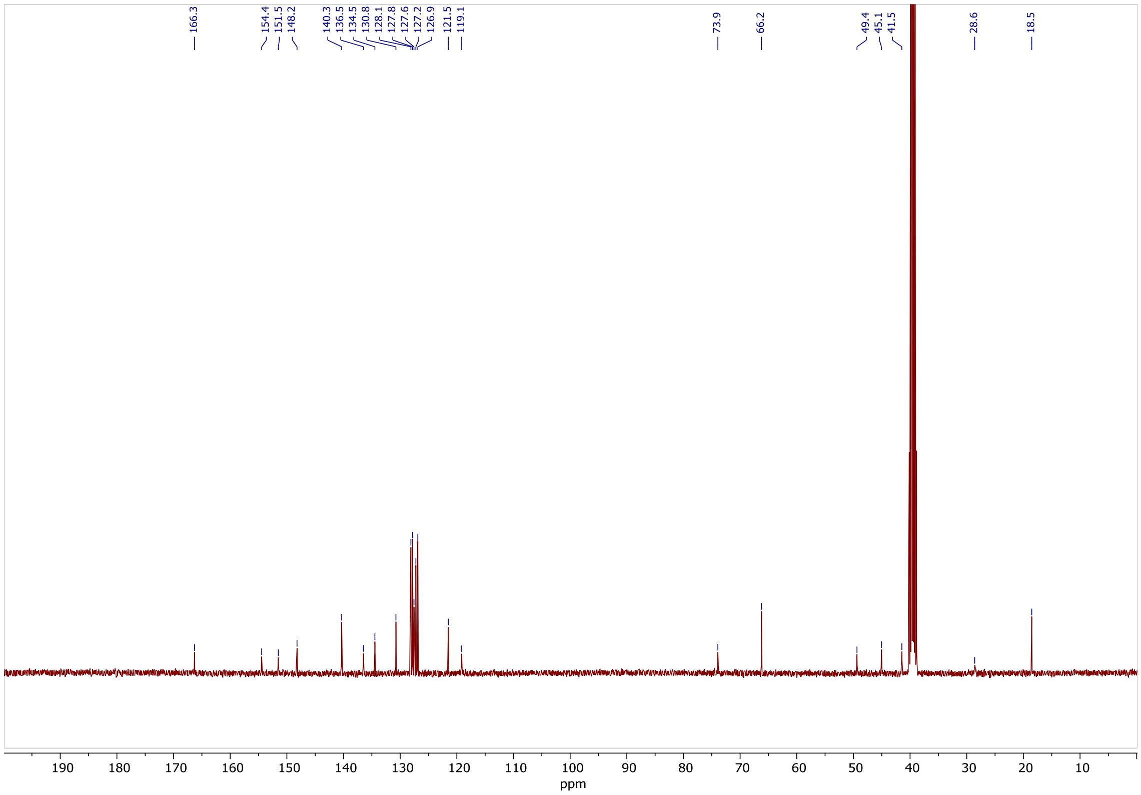

^{1}H NMR spectrum (400 MHz, MeOD, 298 K) of compound **4ia**

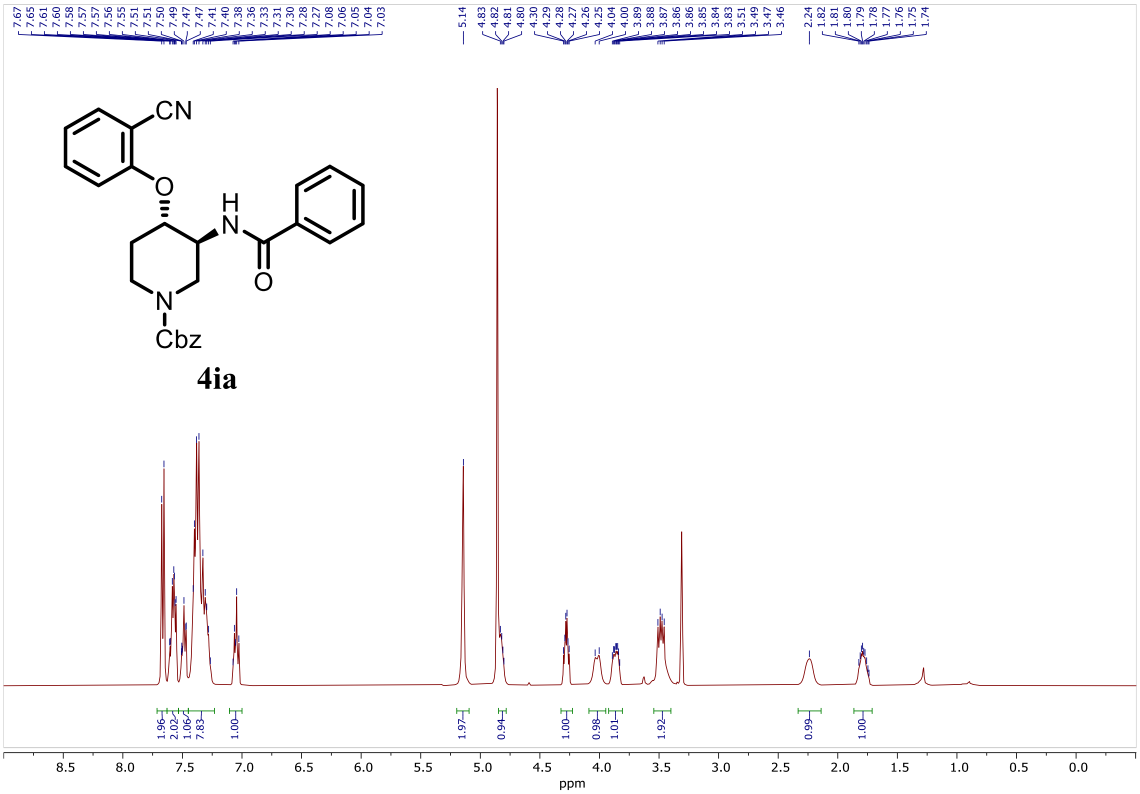

^{13}C{^{1}H} NMR spectrum (101 MHz, MeOD, 298 K) of compound **4ia**

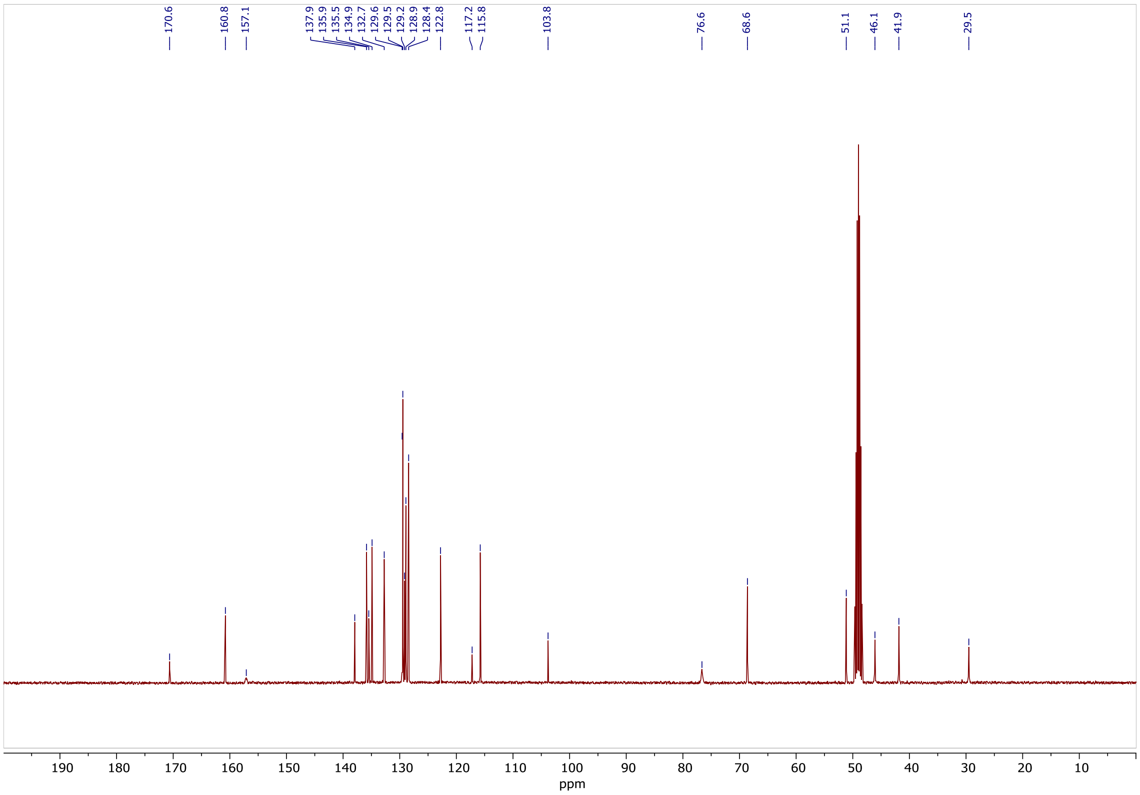

^{1}H NMR spectrum (400 MHz, DMSO-d_6, 343 K) of compound **4ib**

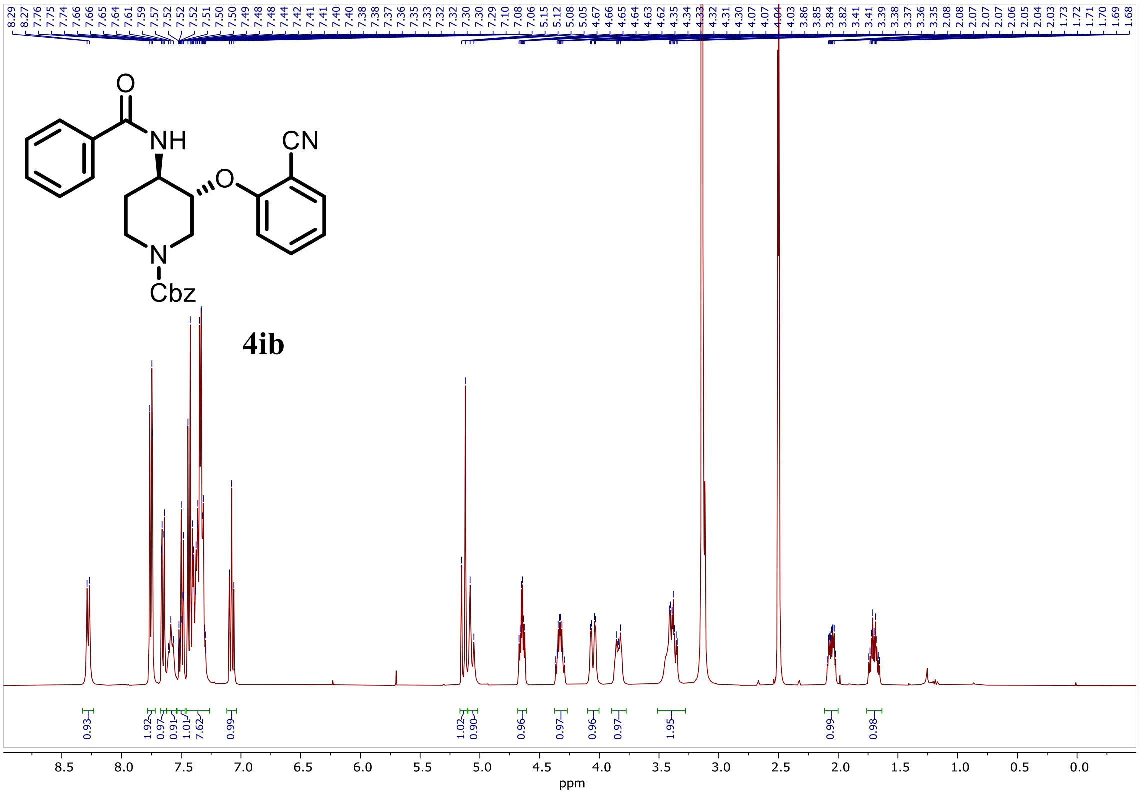

^{13}C{^{1}H} NMR spectrum (101 MHz, DMSO-d_6, 343 K) of compound **4ib**

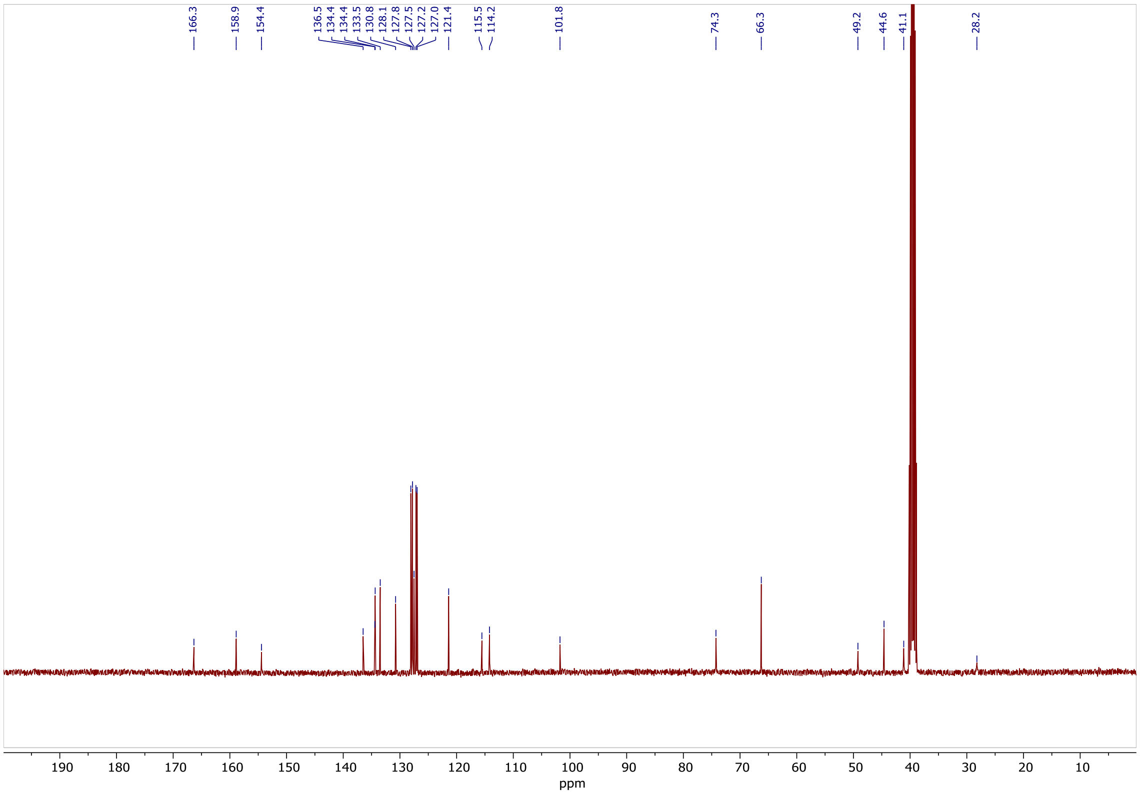

^{1}H NMR spectrum (400 MHz, MeOD, 298 K) of compound **4ja**

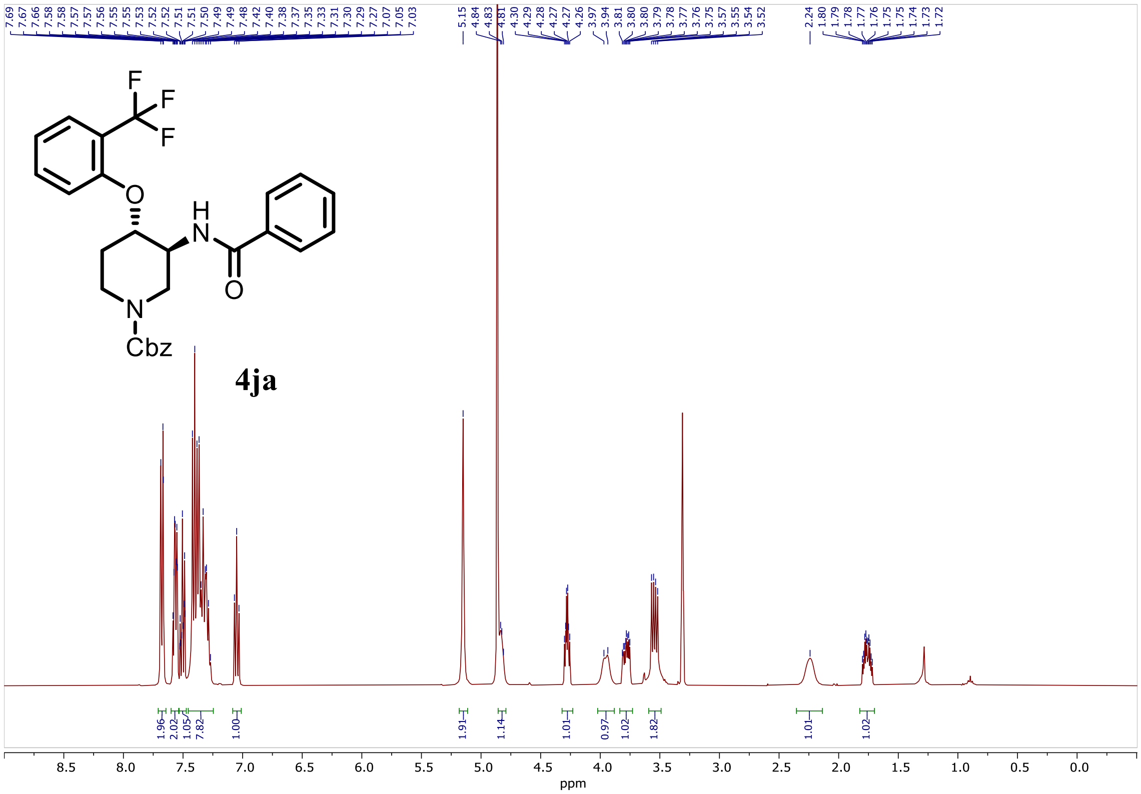

$^{13}C\{^{1}H\}$ NMR spectrum (101 MHz, MeOD, 298 K) of compound **4ja**

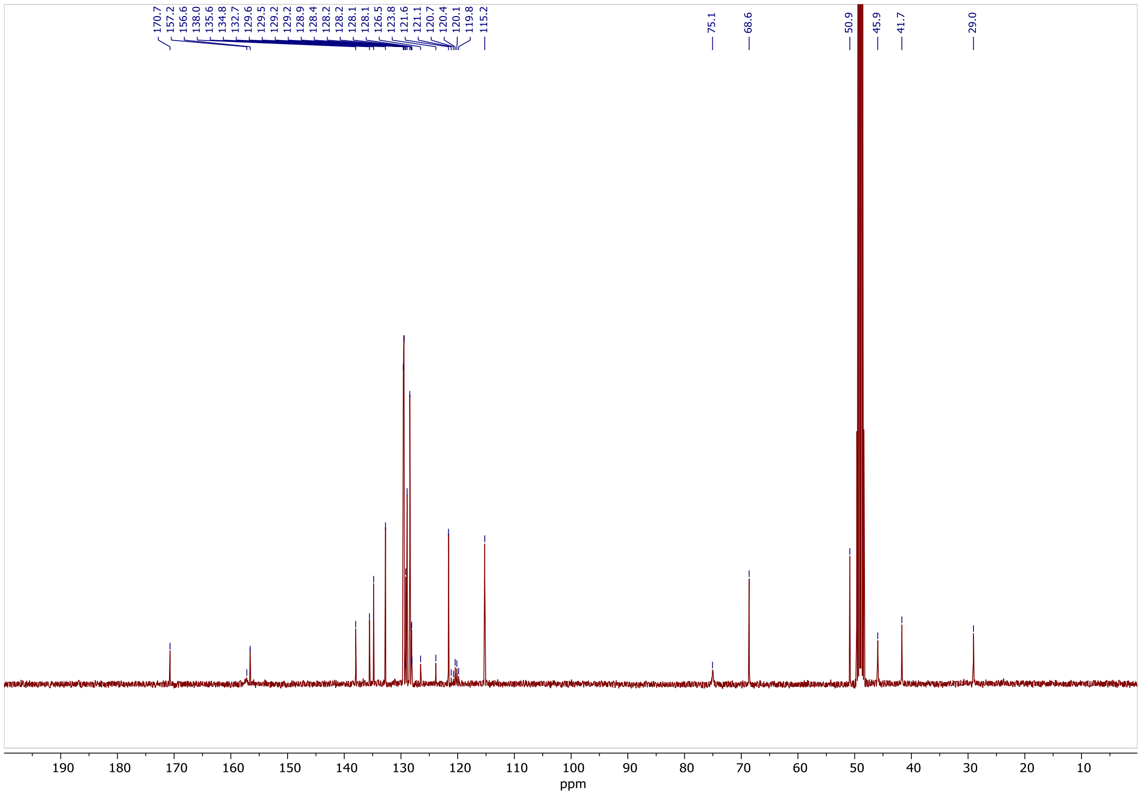

^{1}H NMR spectrum (400 MHz, DMSO-d_6, 343 K) of compound **4jb**

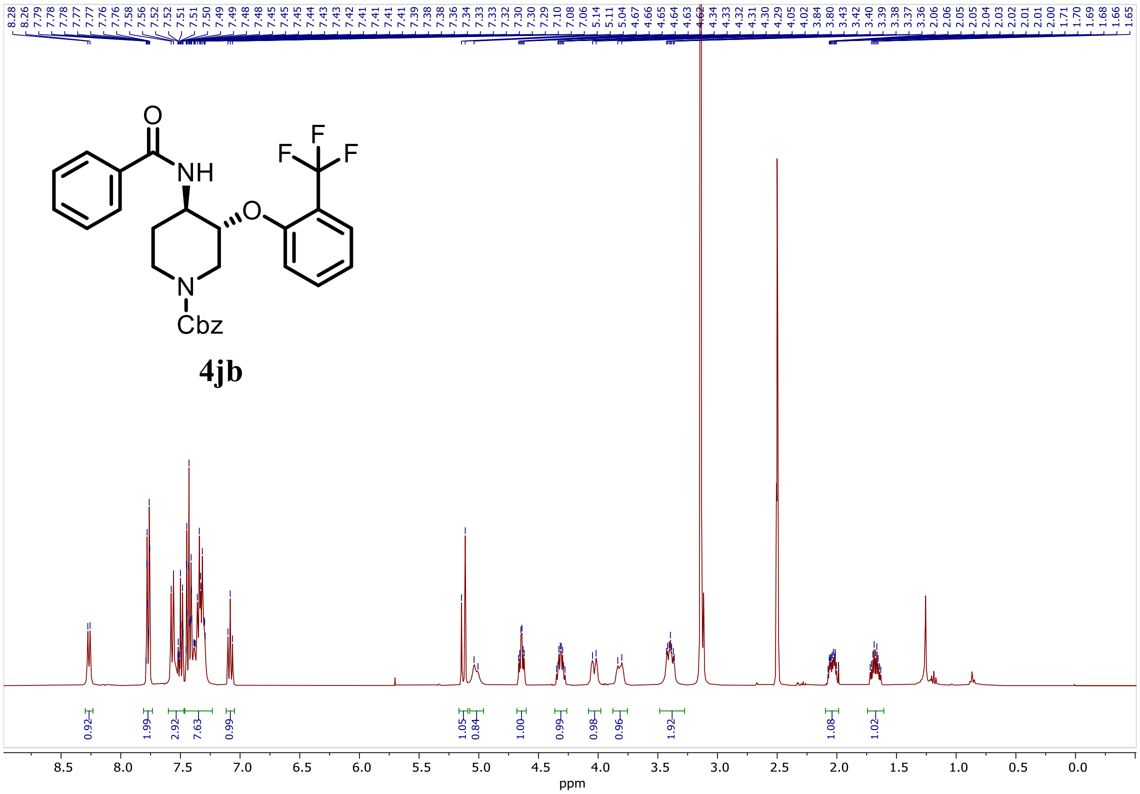

$^{13}C\{^{1}H\}$ NMR spectrum (101 MHz, DMSO-d_6, 343 K) of compound **4jb**

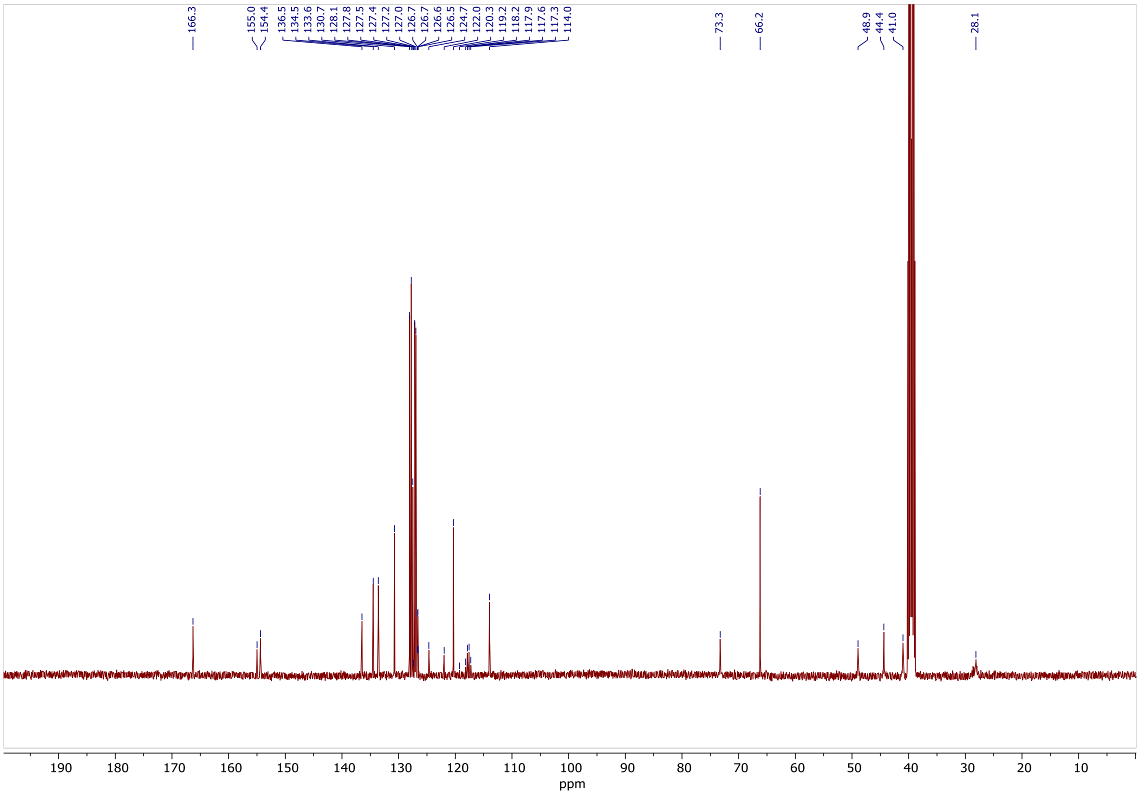

^{1}H NMR spectrum (400 MHz, DMSO-d_6, 343 K) of compound **6a**

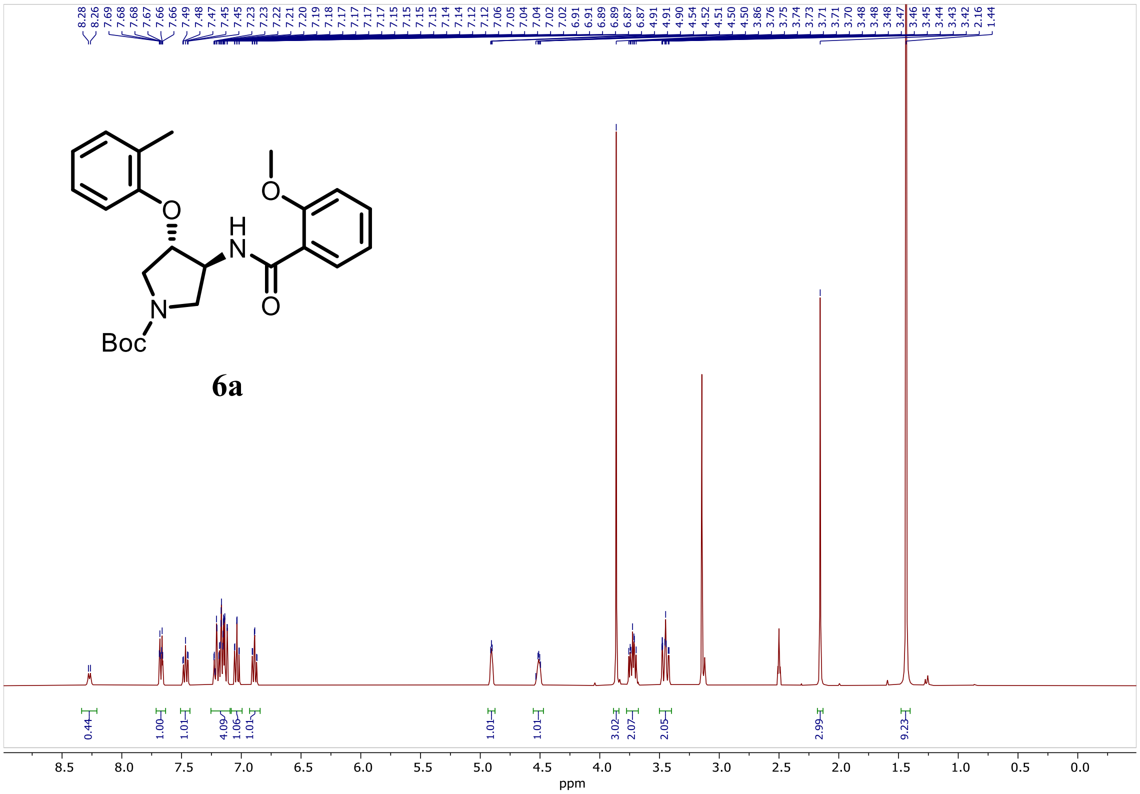

^{13}C{^{1}H} NMR spectrum (101 MHz, DMSO--d_6, 343 K) of compound **6a**

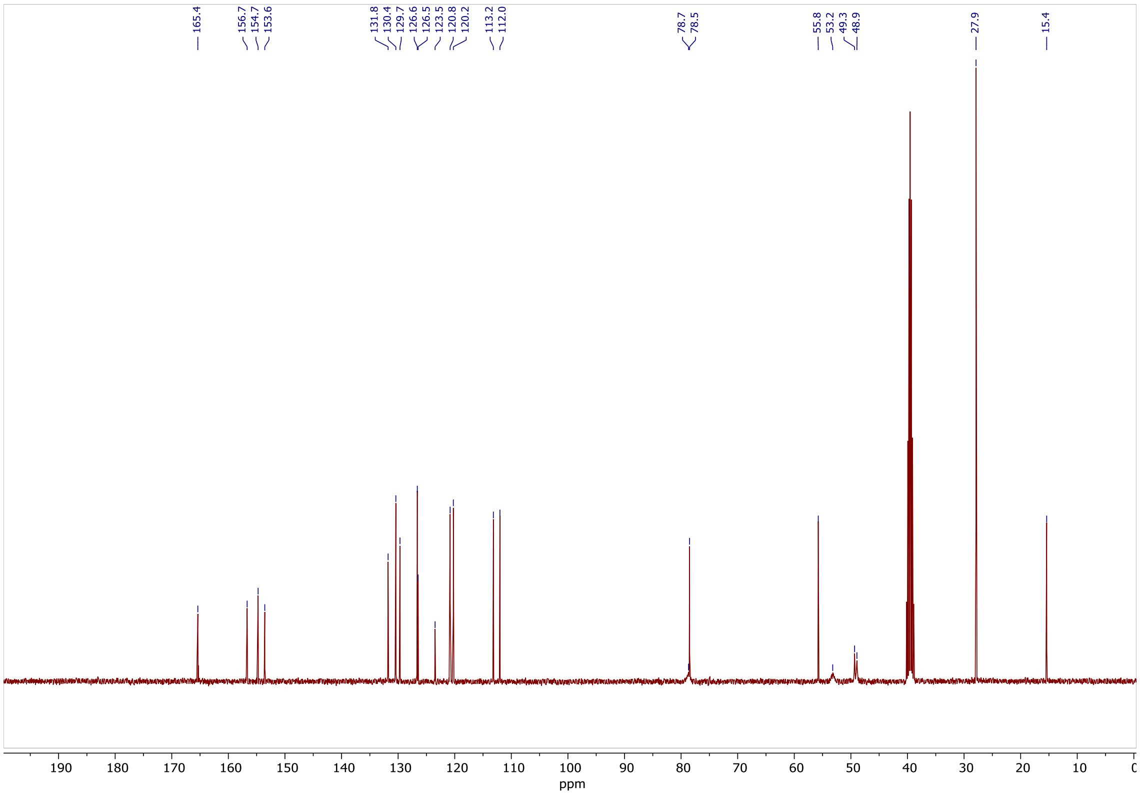

^{1}H NMR spectrum (400 MHz, DMSO-d_6, 343 K) of compound **6b**

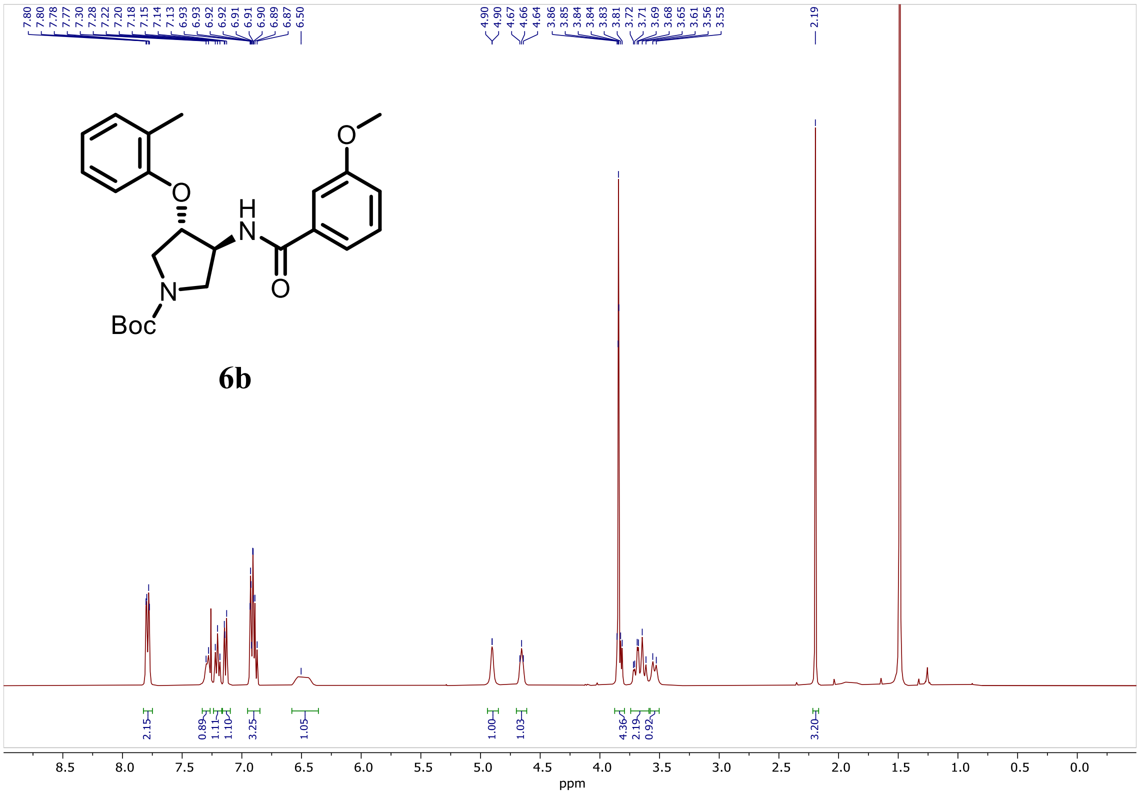

^{13}C{^{1}H} NMR spectrum (101 MHz, DMSO-d_6, 343 K) of compound **6b**

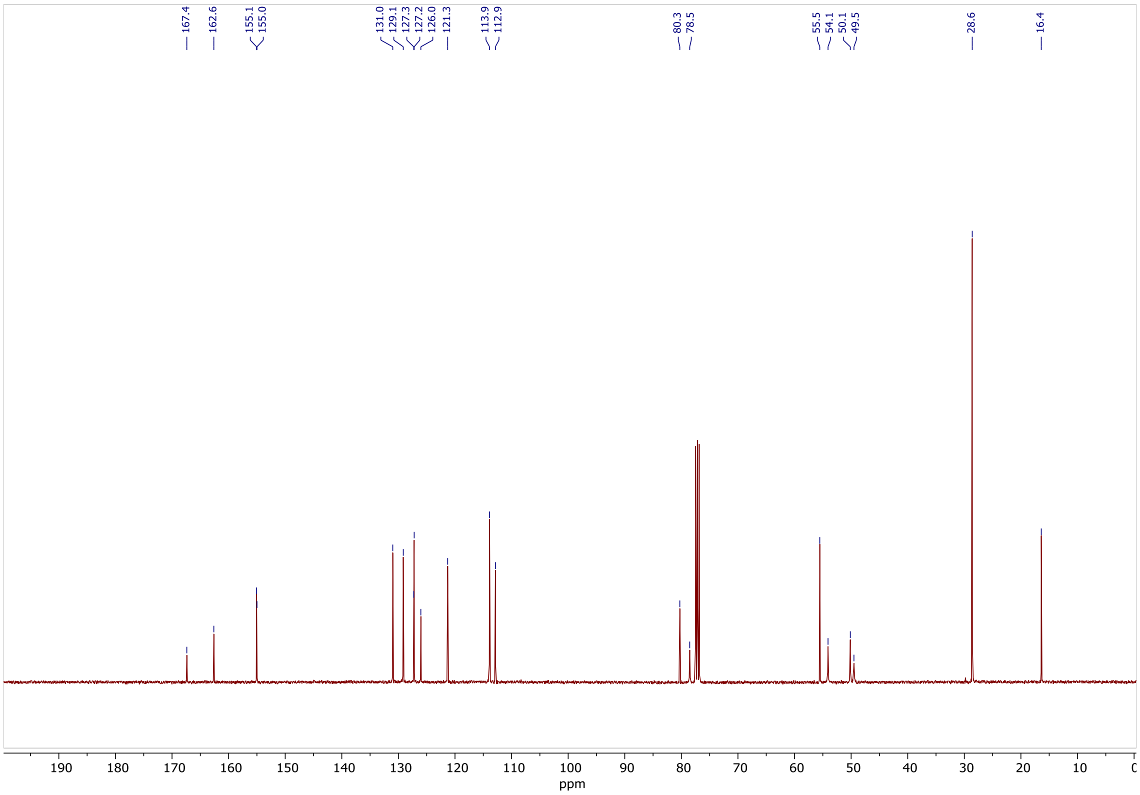

^{1}H NMR spectrum (400 MHz, $CDCl_3$, 298 K) of compound **6c**

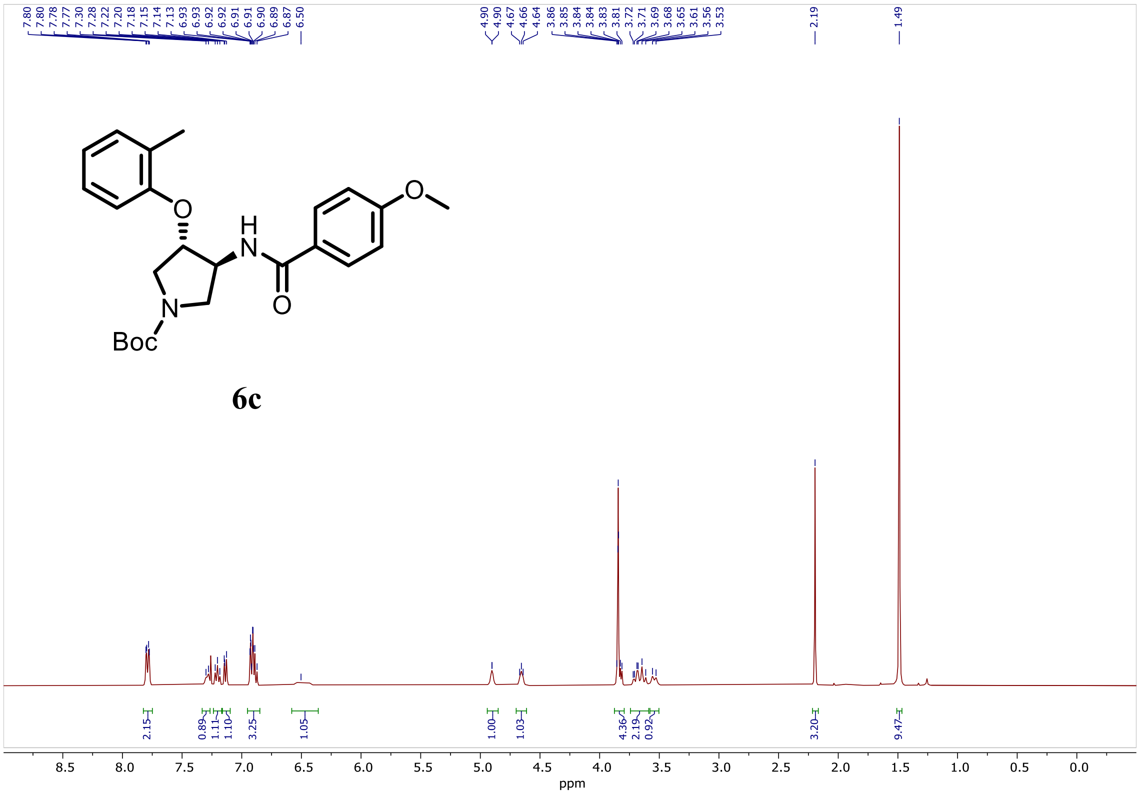

^{13}C{^{1}H} NMR spectrum (101 MHz, $CDCl_3$, 298 K) of compound **6c**

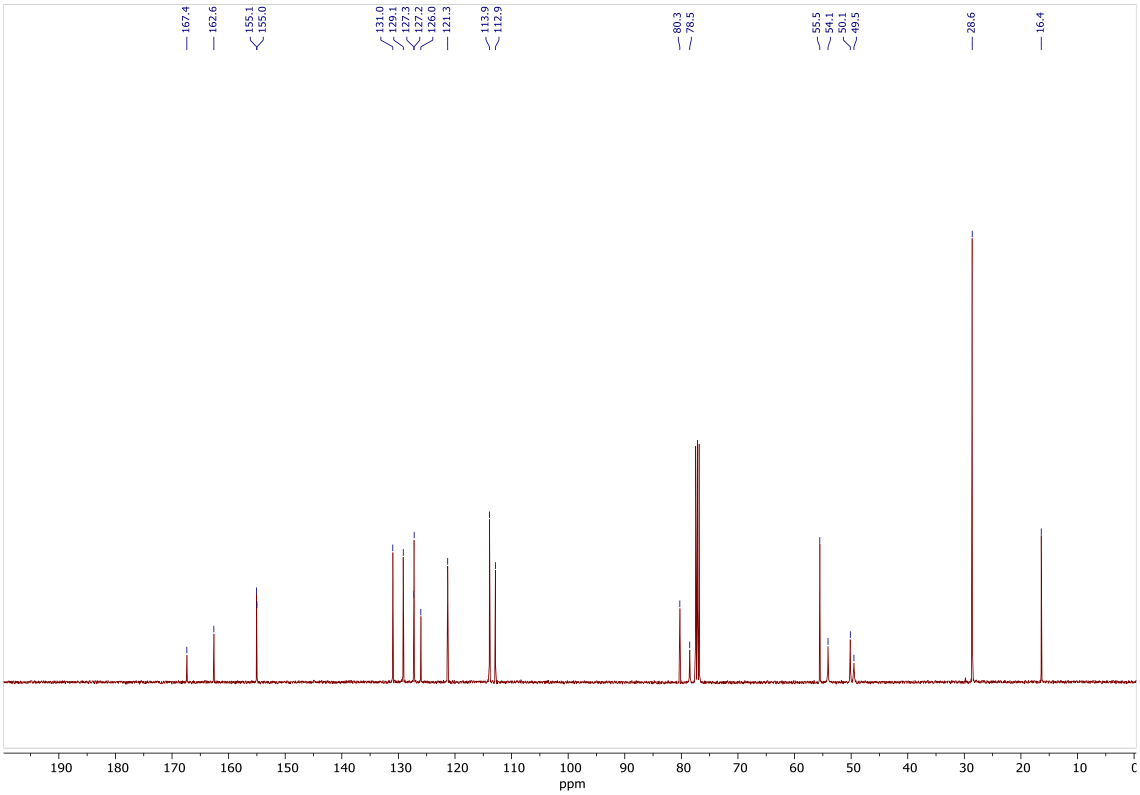

^{1}H NMR spectrum (400 MHz, DMSO-d_6, 343 K) of compound **6d**

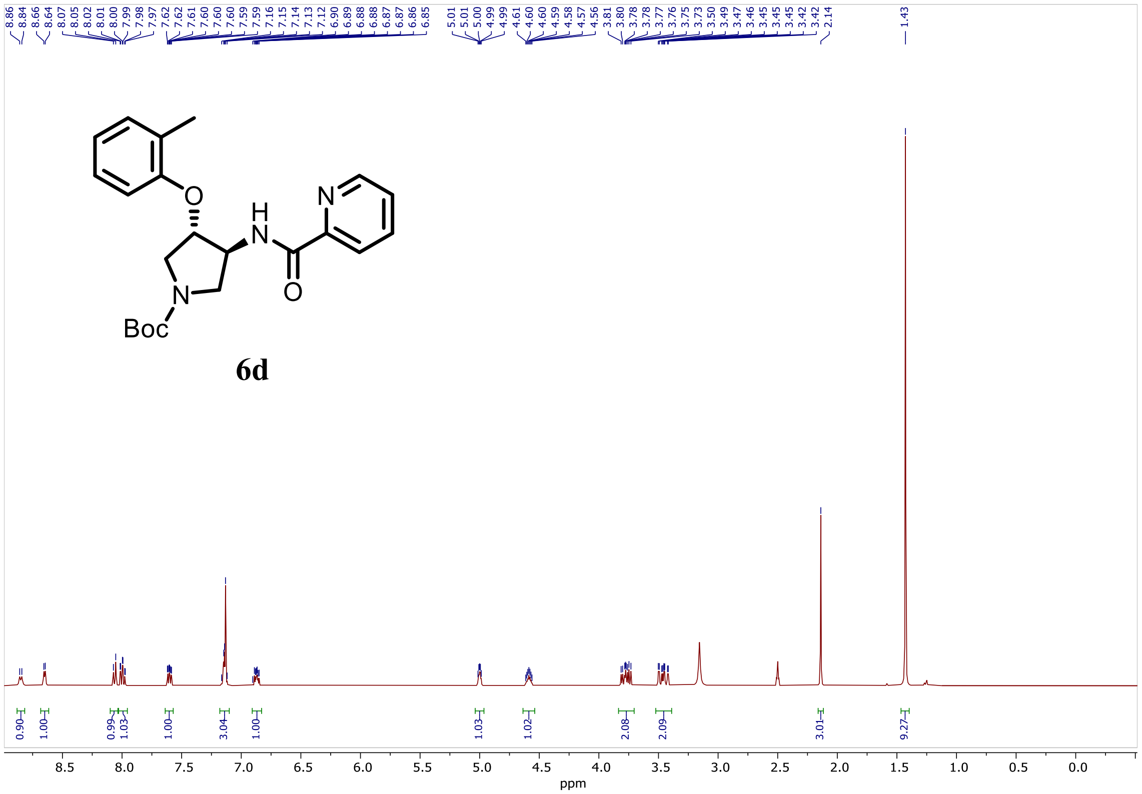

^{13}C{^{1}H} NMR spectrum (101 MHz, DMSO-d_6, 343 K) of compound **6d**

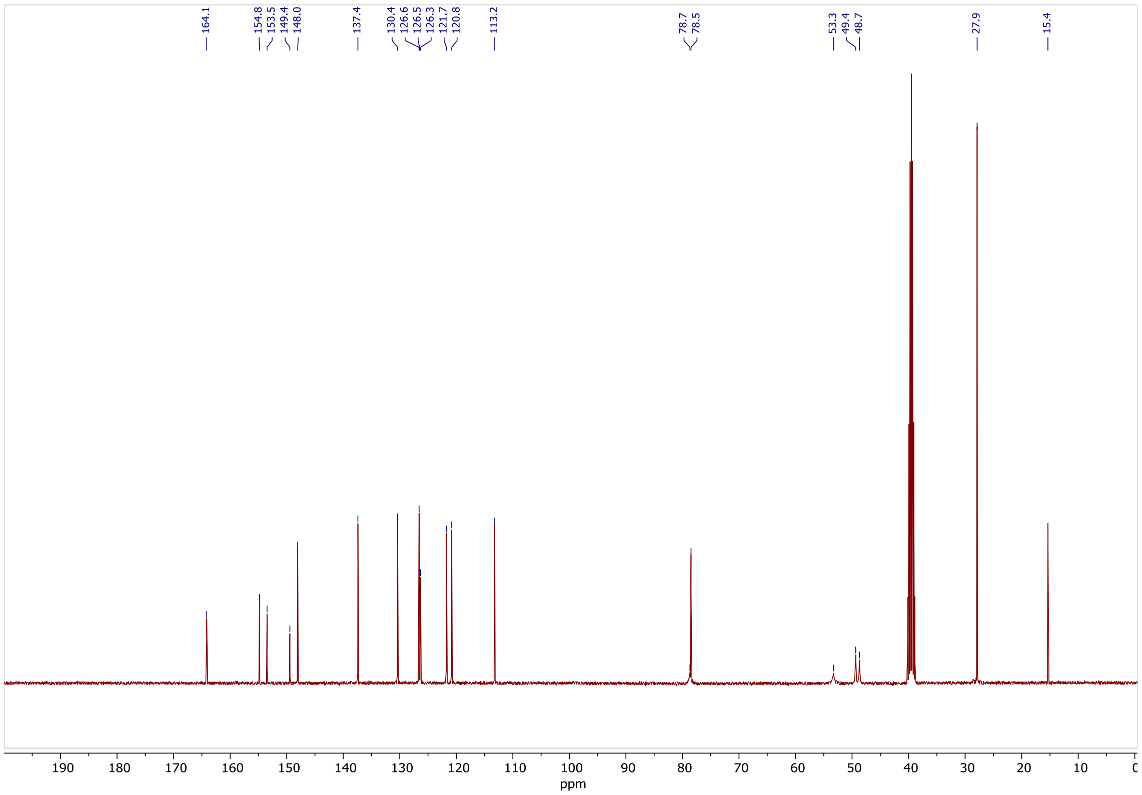

^{1}H NMR spectrum (400 MHz, DMSO-d_6, 343 K) of compound **6e**

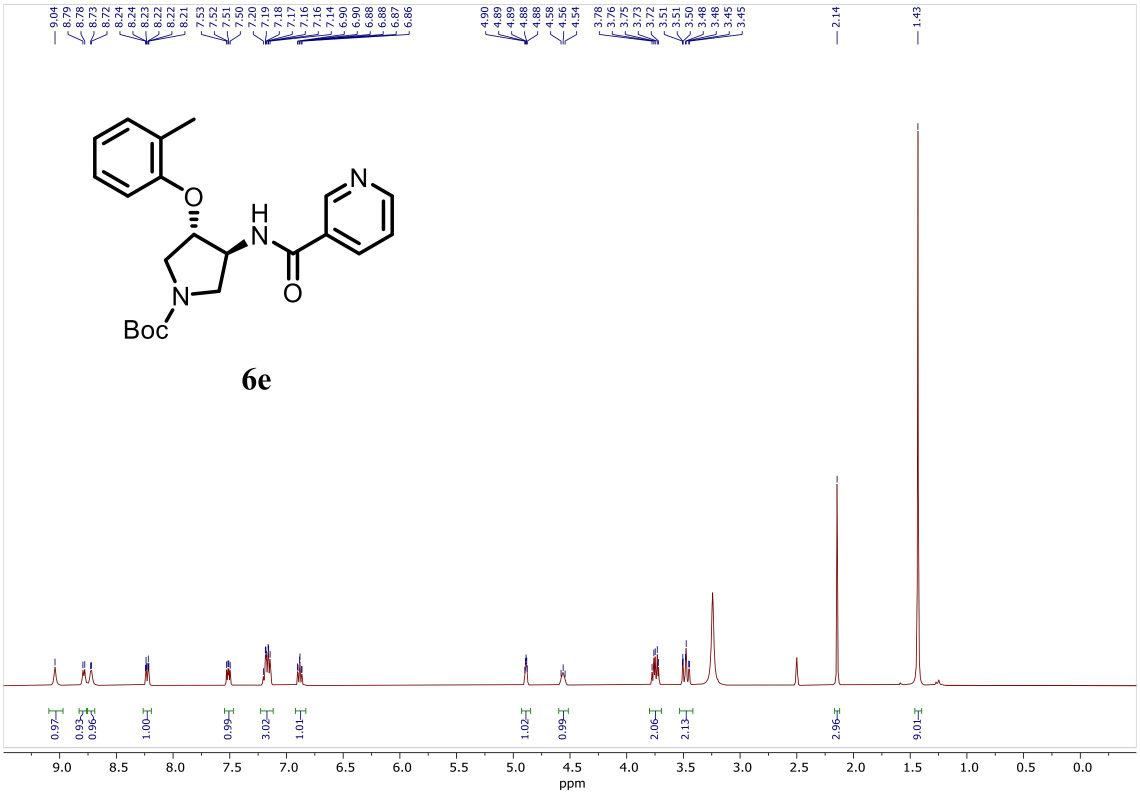

$^{13}C\{^{1}H\}$ NMR spectrum (101 MHz, DMSO-d_6, 343 K) of compound **6e**

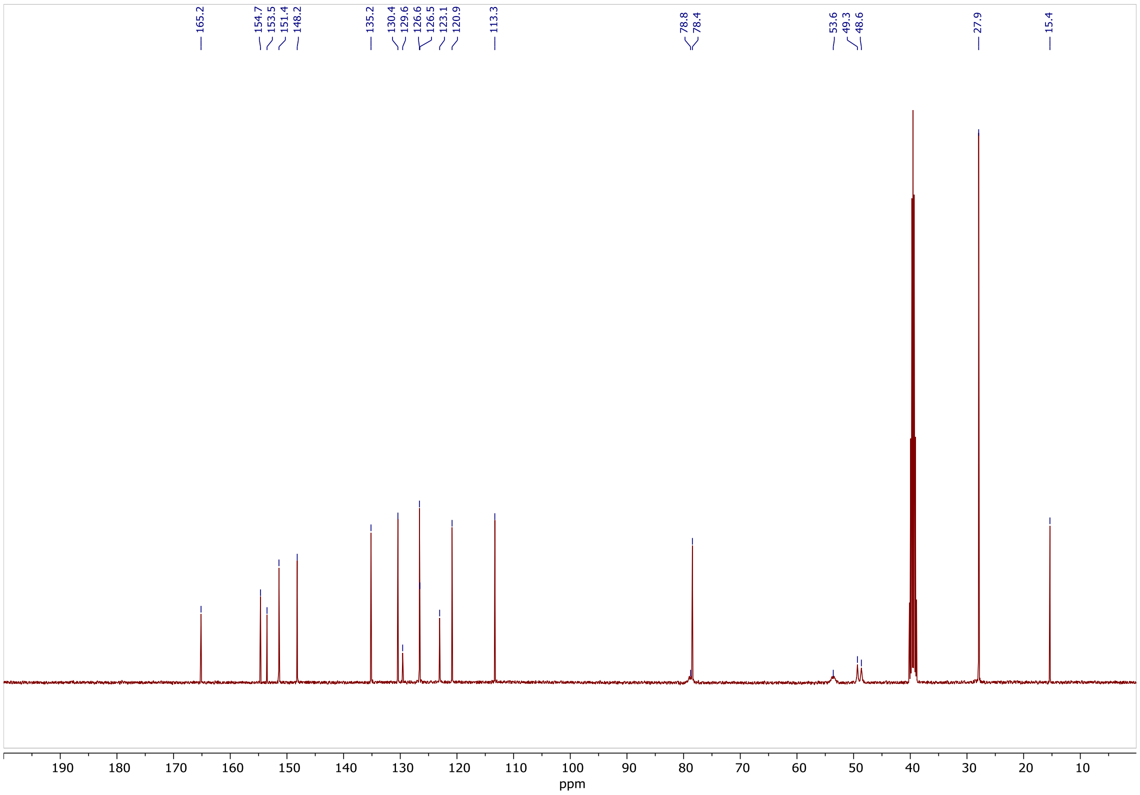

^{1}H NMR spectrum (400 MHz, DMSO-d_6, 343 K) of compound **6f**

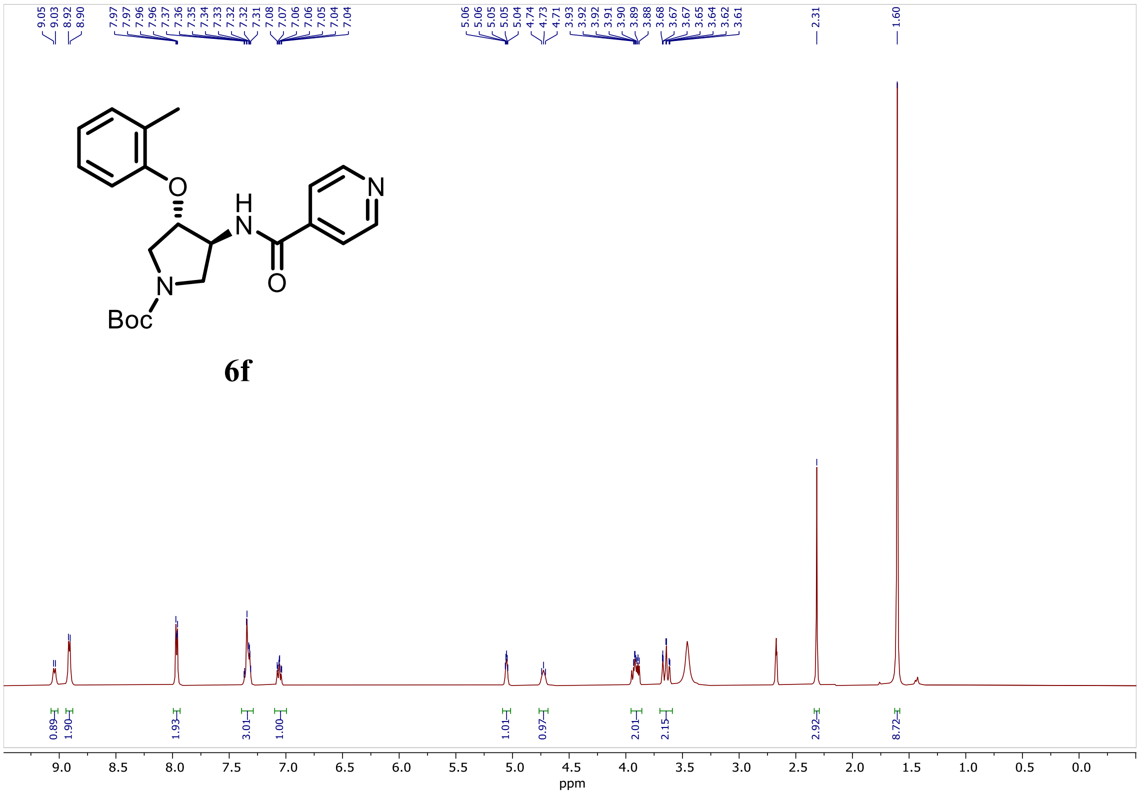

^{13}C{^{1}H} NMR spectrum (101 MHz, DMSO-d_6, 343 K) of compound **6f**

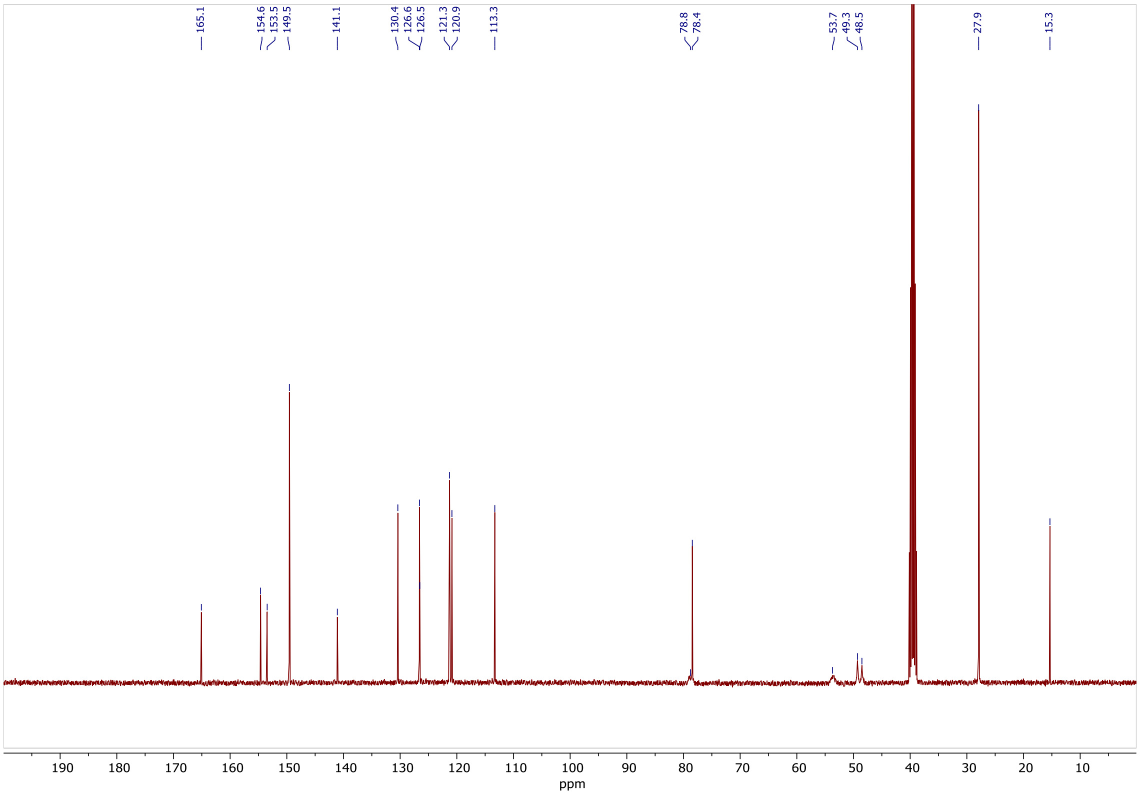

^{1}H NMR spectrum (400 MHz, $CDCl_3$, 298 K) of compound **6g**

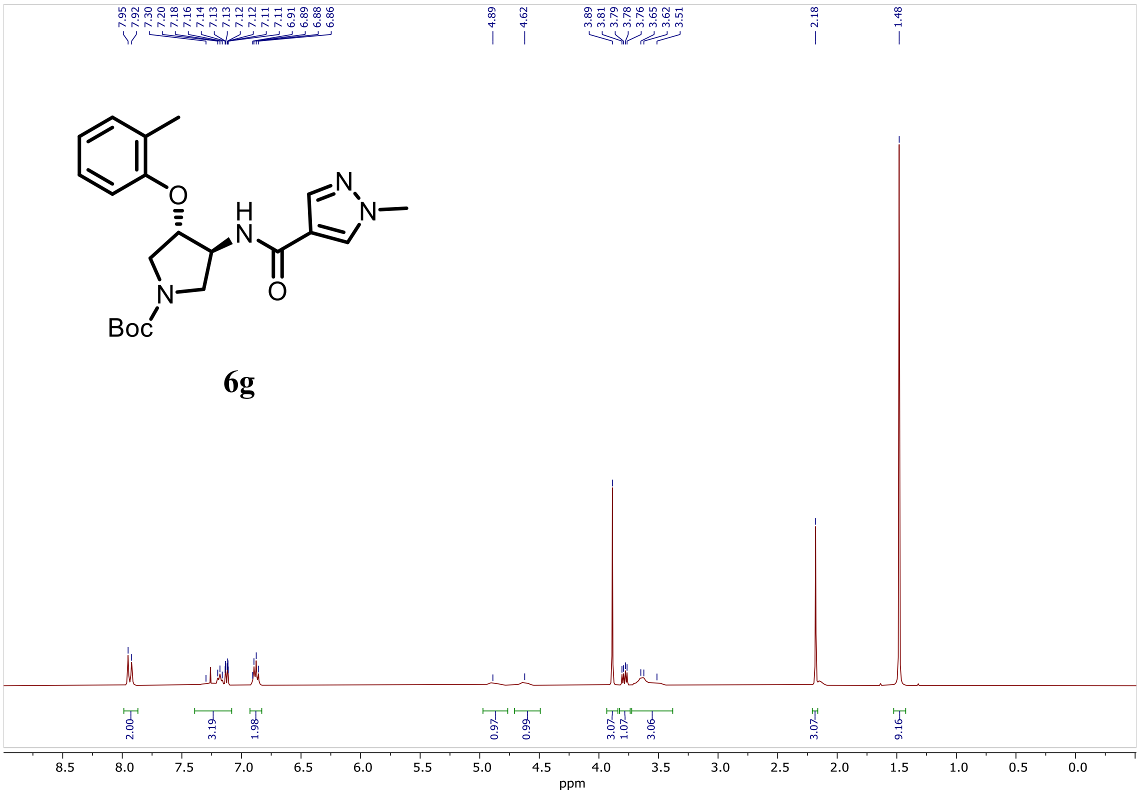

$^{13}C\{^{1}H\}$ NMR spectrum (101 MHz, $CDCl_3$, 298 K) of compound **6g**

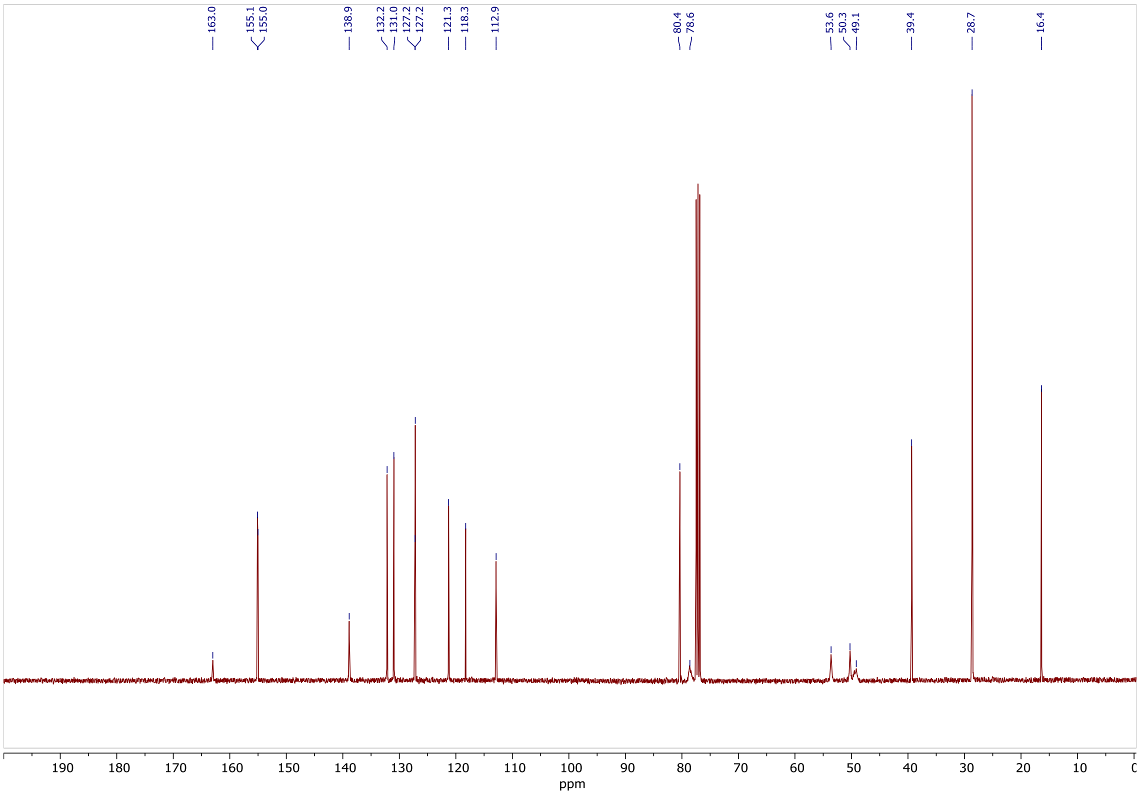

^{1}H NMR spectrum (400 MHz, $CDCl_3$, 298 K) of compound **6h**

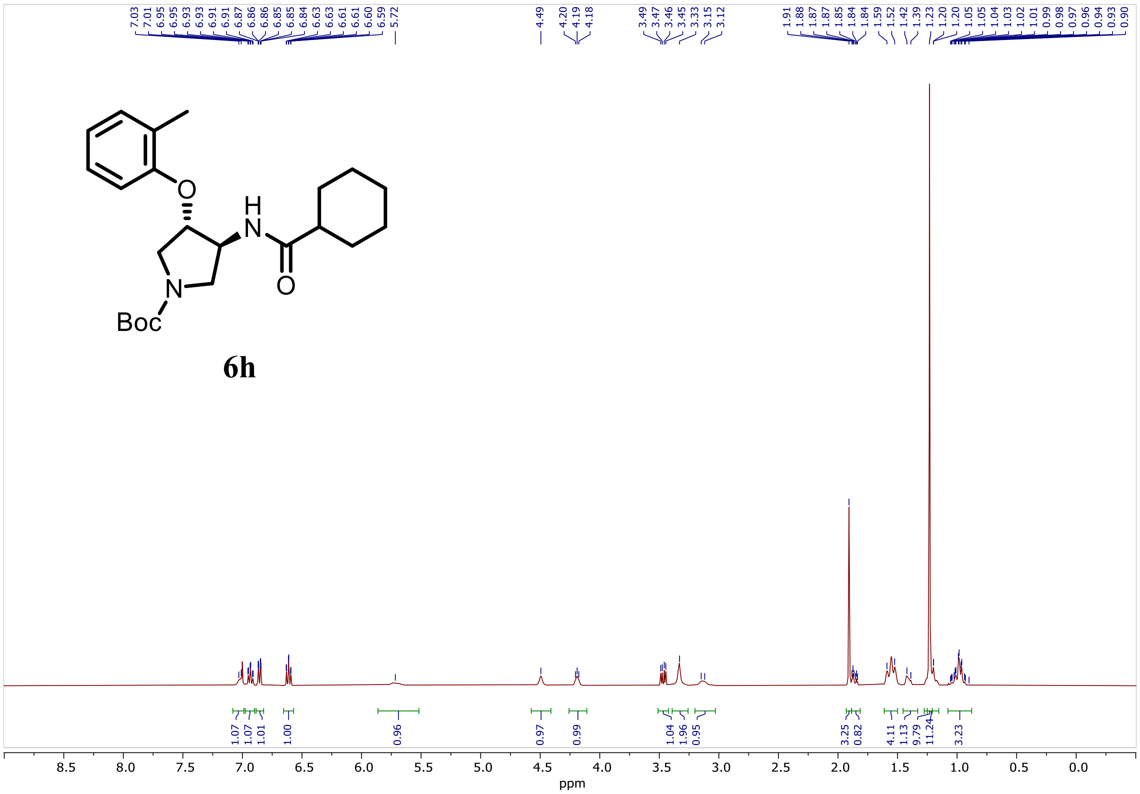

^{13}C{^{1}H} NMR spectrum (101 MHz, $CDCl_3$, 298 K) of compound **6h**

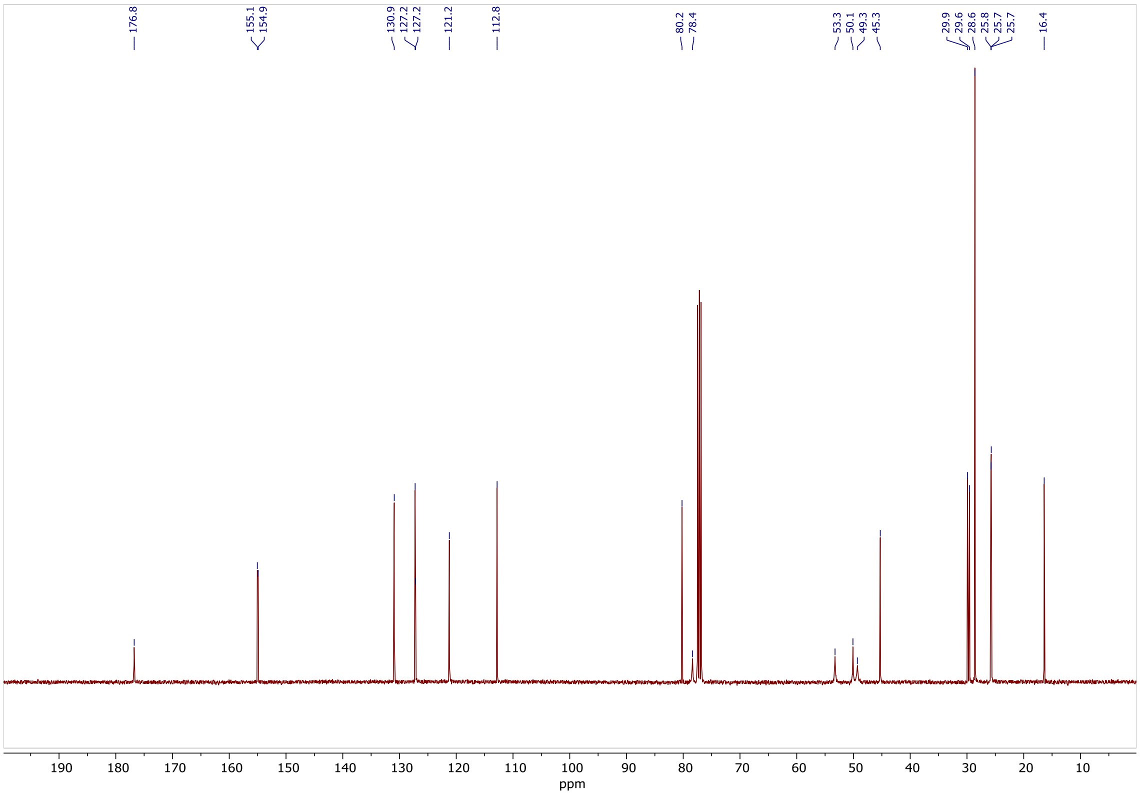

^{1}H NMR spectrum (400 MHz, DMSO-d_6, 343 K) of compound **6i**

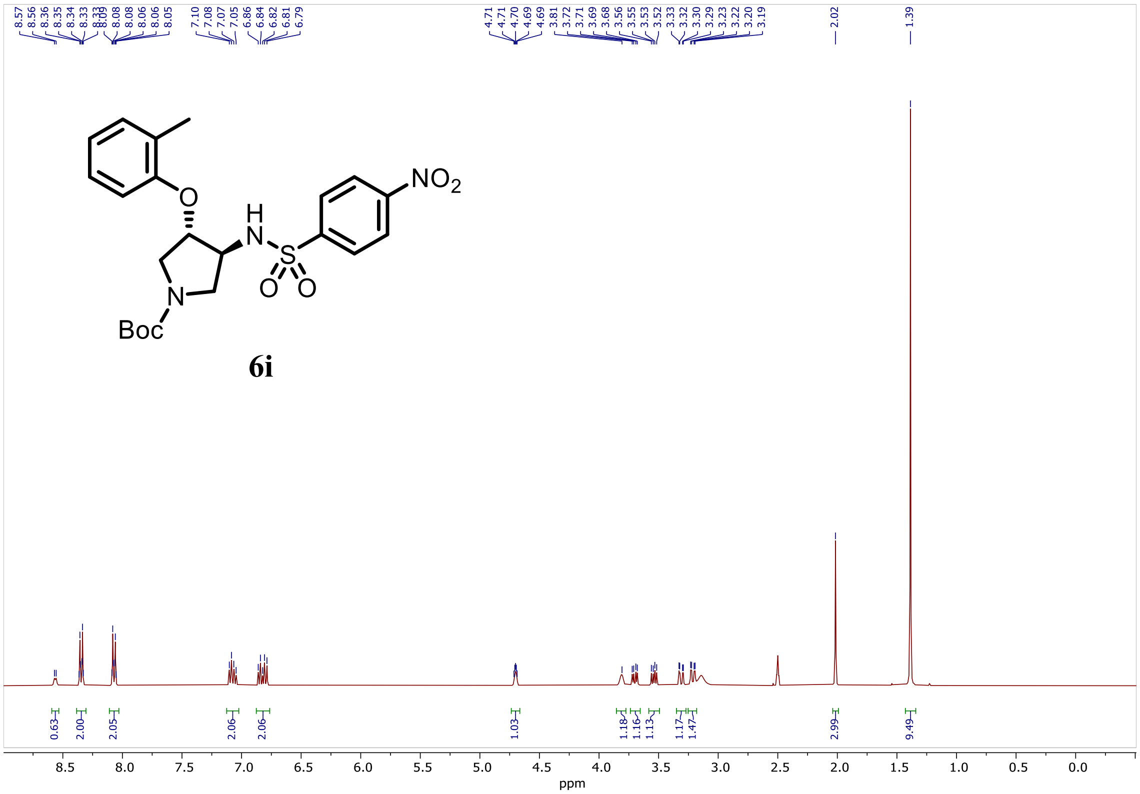

^{13}C{^{1}H} NMR spectrum (101 MHz, DMSO-d_6, 343 K) of compound **6i**

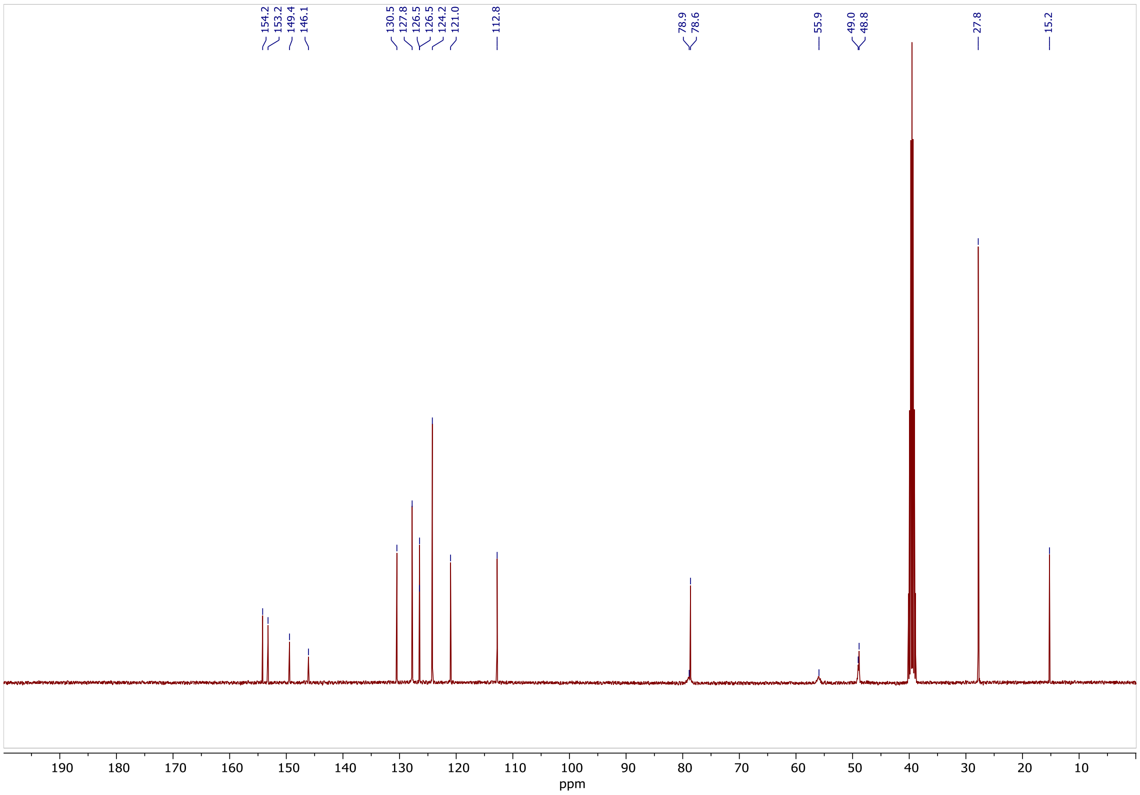

^{1}H NMR spectrum (400 MHz, MeOD, 298 K) of compound **6j**

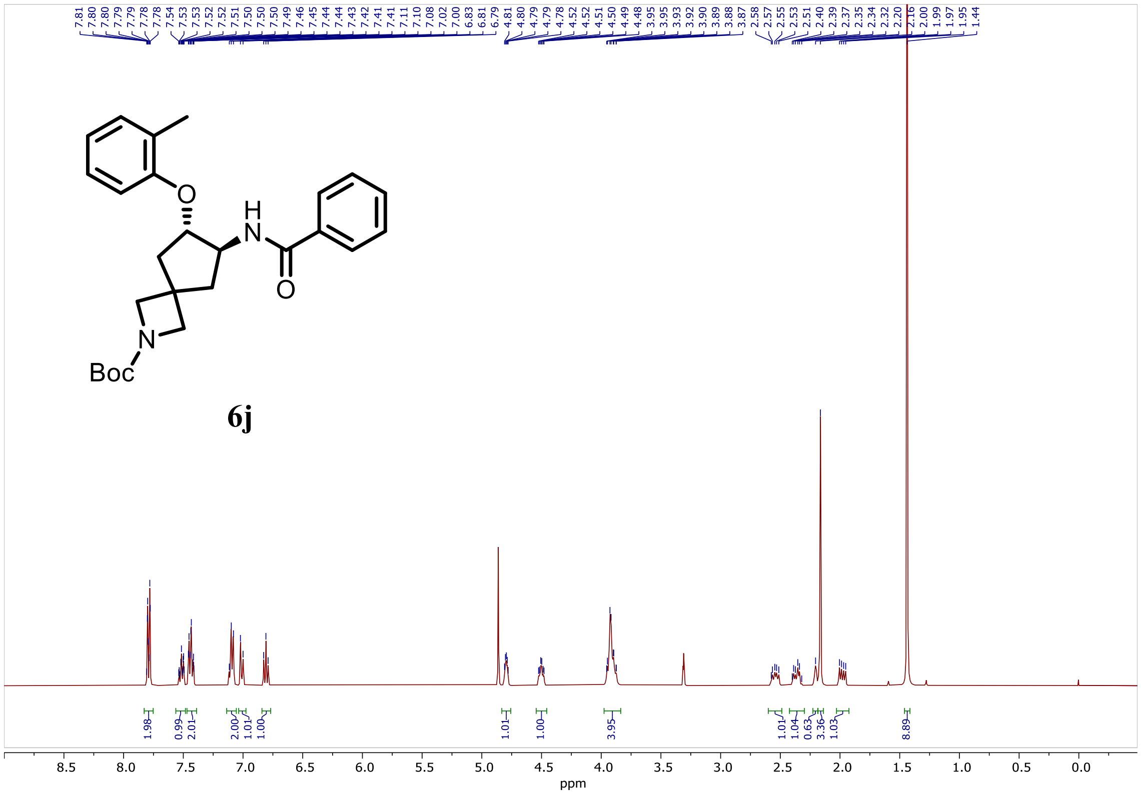

^{13}C{^{1}H} NMR spectrum (101 MHz, MeOD, 298 K) of compound **6j**

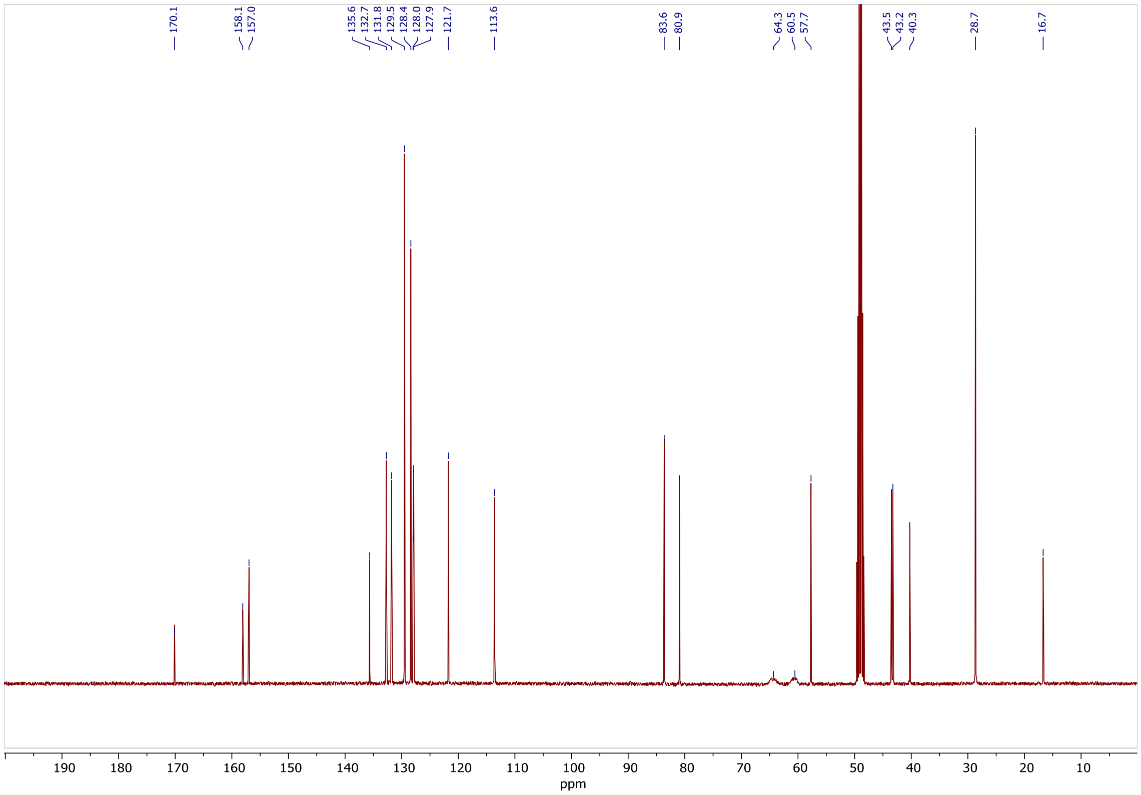

^{1}H NMR spectrum (400 MHz, DMSO-d_6, 343 K) of compound **6ka**

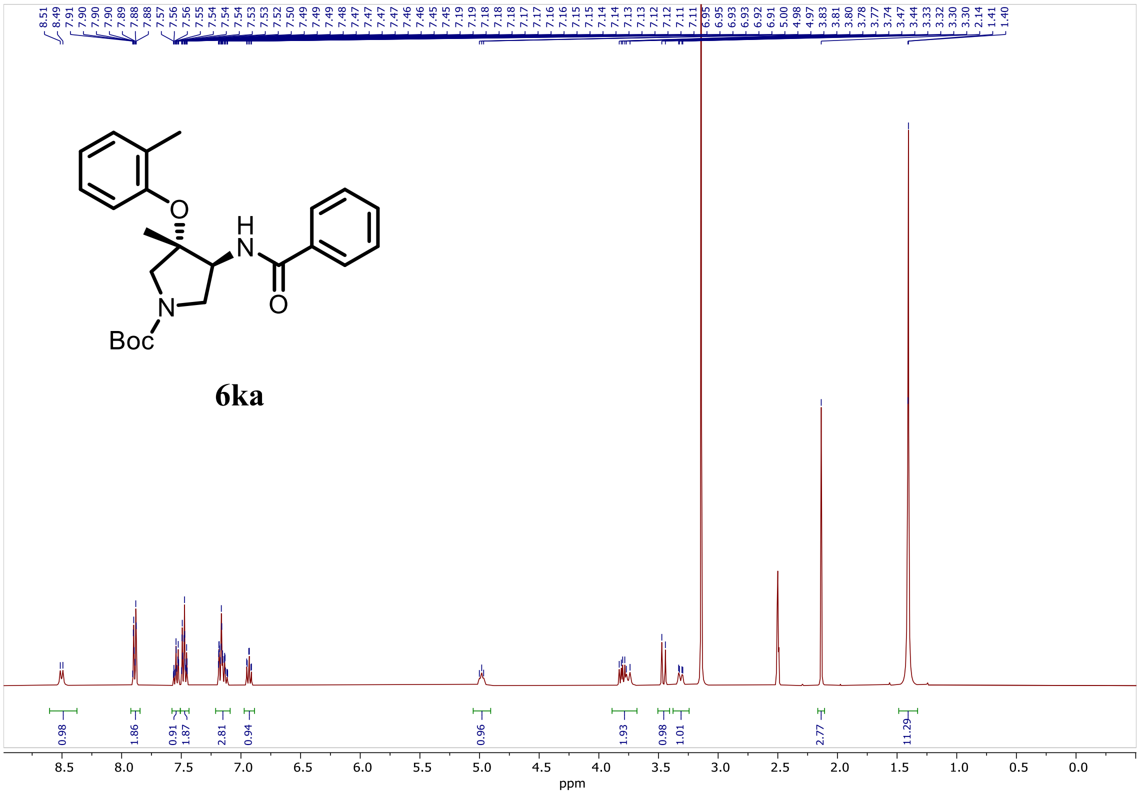

$^{13}C\{^{1}H\}$ NMR spectrum (101 MHz, DMSO-d_6, 343 K) of compound **6ka**

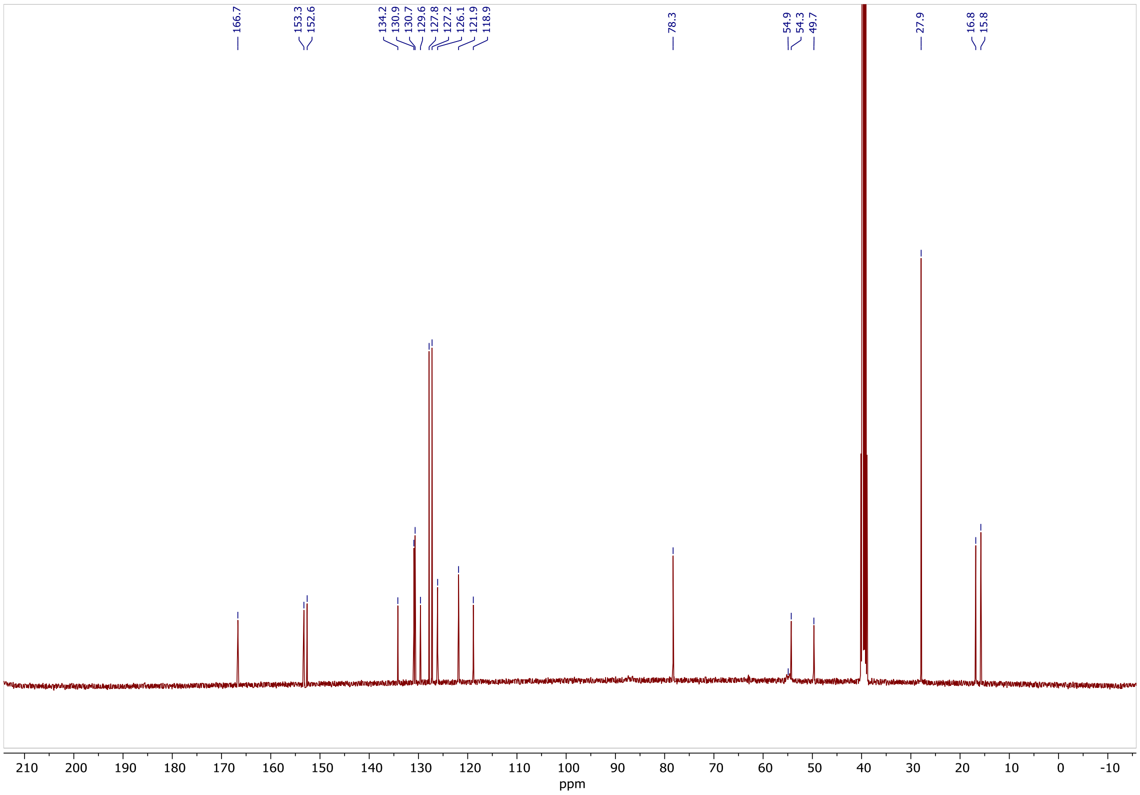

HSQC NMR spectrum (400 MHz, 101 MHz, DMSO-d_6, 343 K) of compound **6ka**

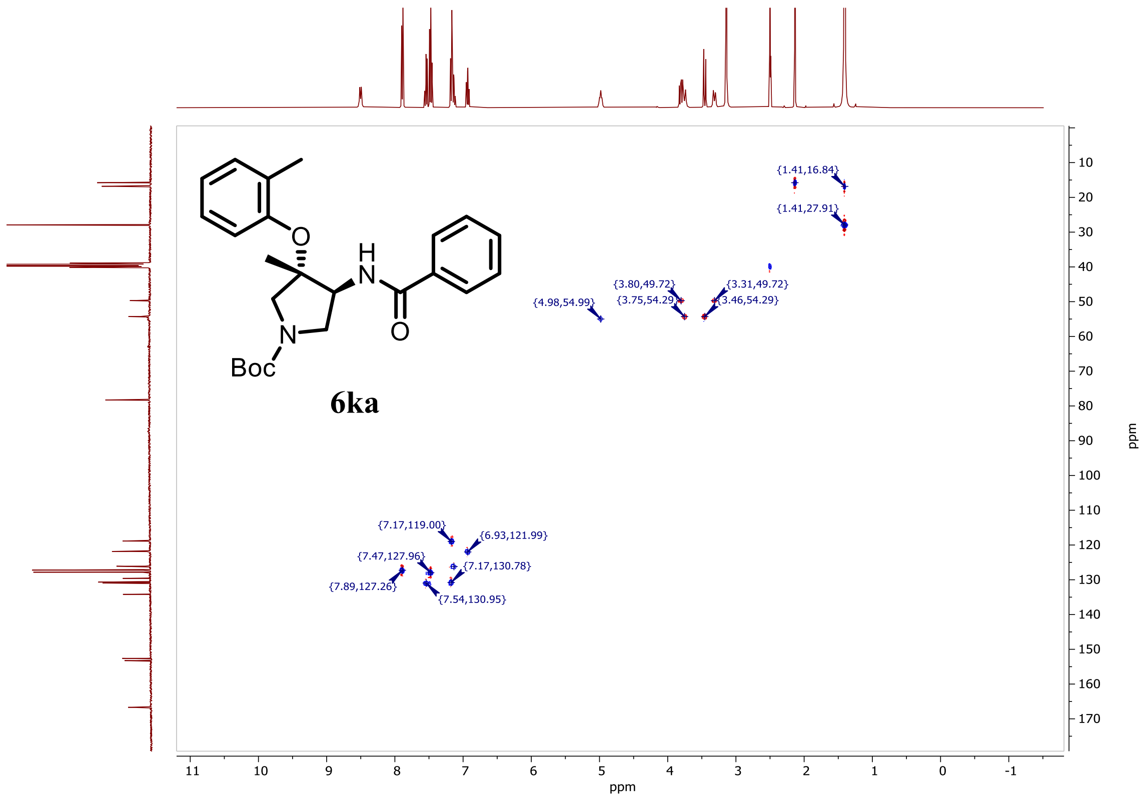

NOESY NMR spectrum (400 MHz, DMSO-d_6, 343 K) of compound **6ka**

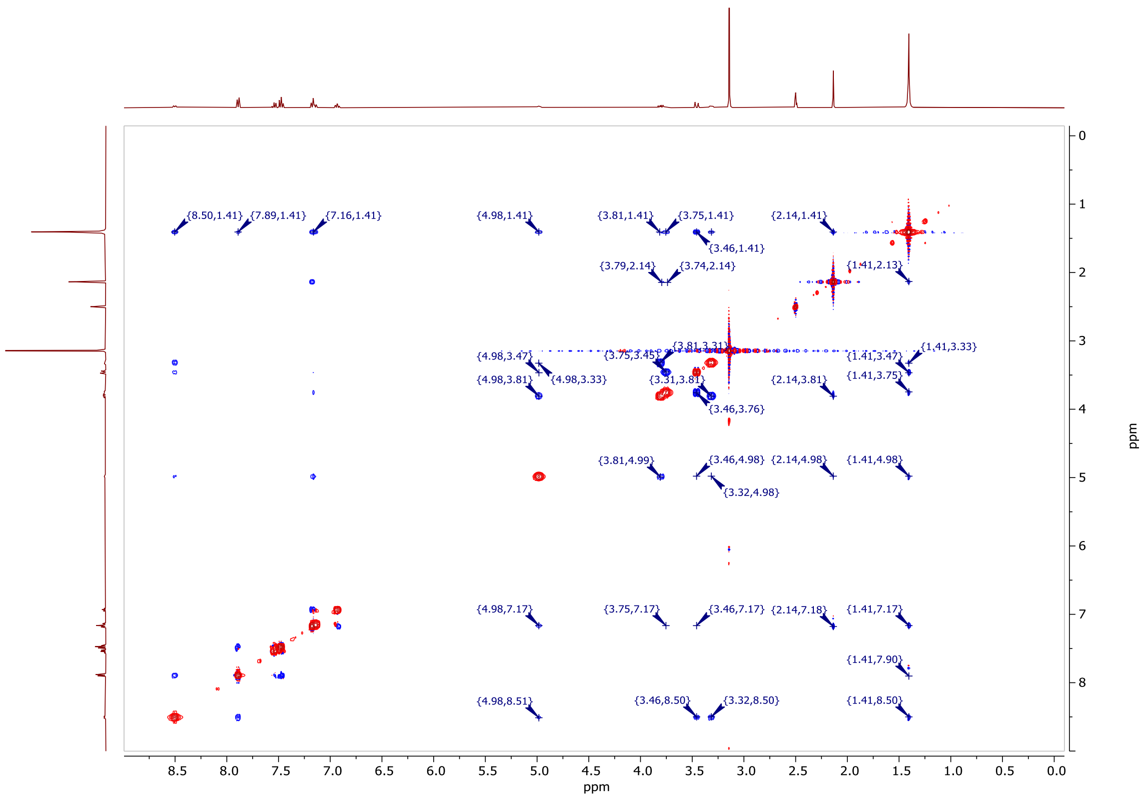

^{1}H NMR spectrum (400 MHz, DMSO-d_6, 298 K) of compound **6ka’**

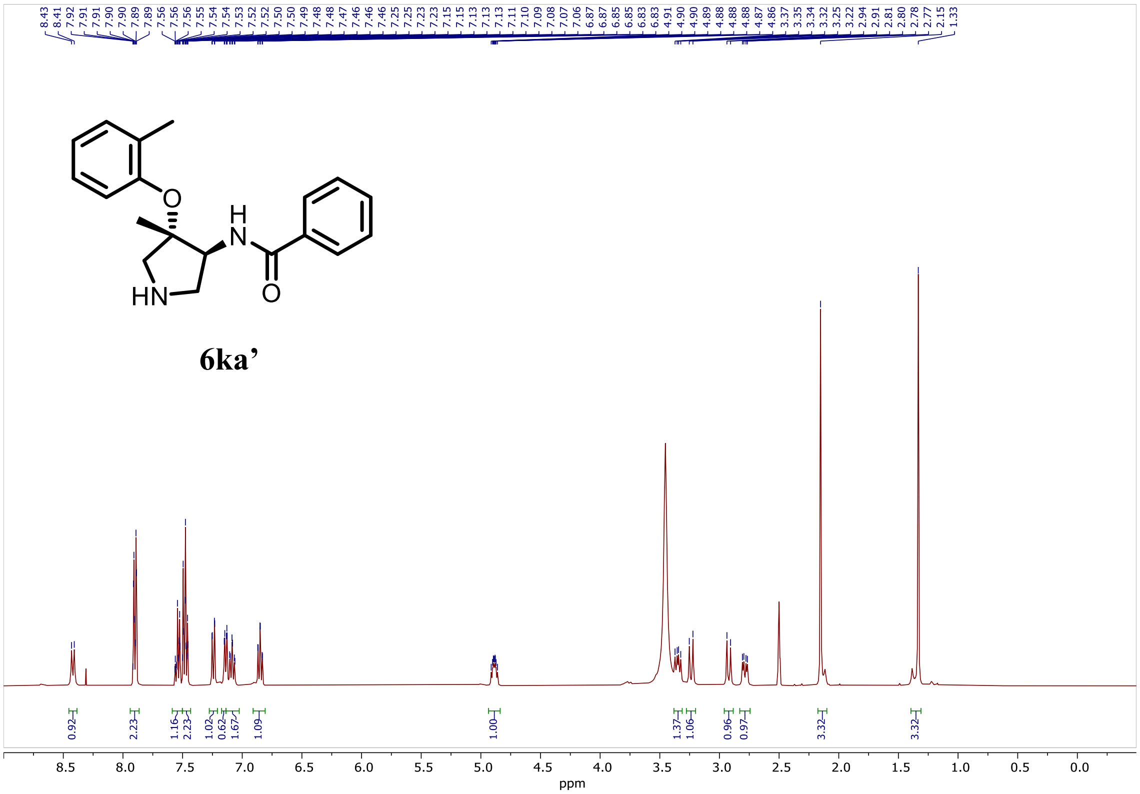

^{13}C{^{1}H} NMR spectrum (101 MHz, DMSO-d_6, 298 K) of compound **6ka’**

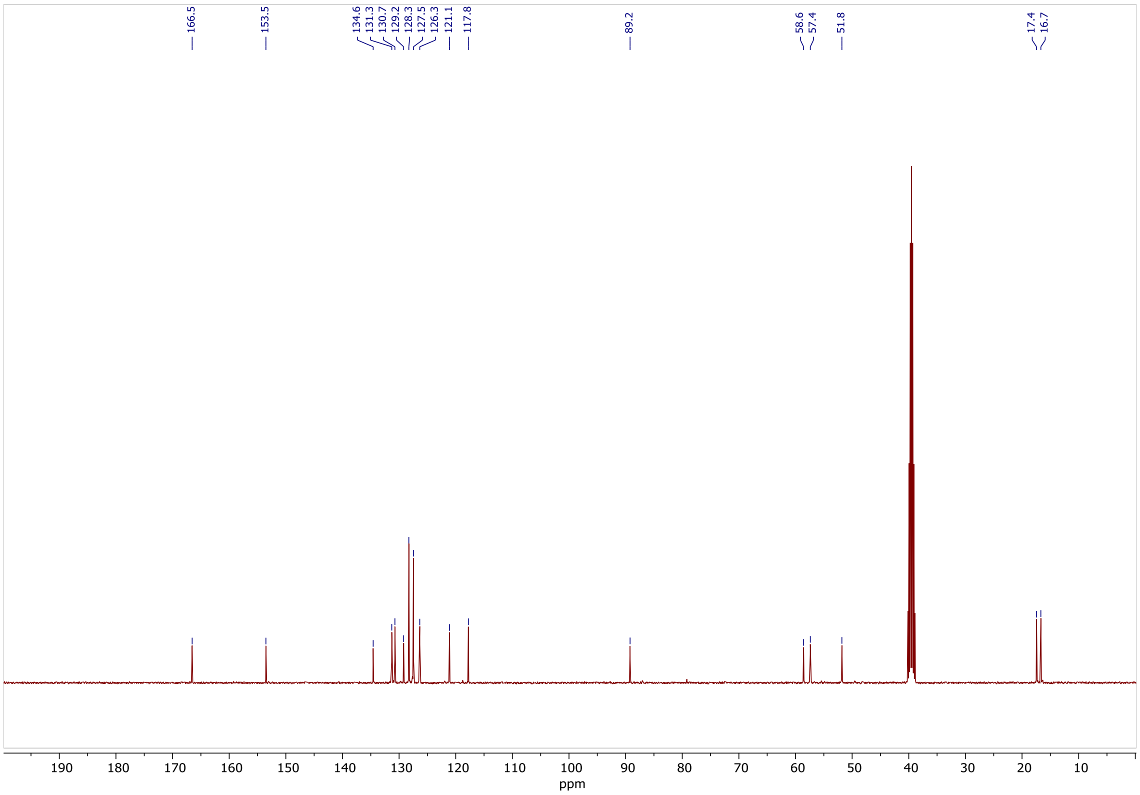

HSQC NMR spectrum (400 MHz, 101 MHz, DMSO-d_6, 298 K) of compound **6ka’**

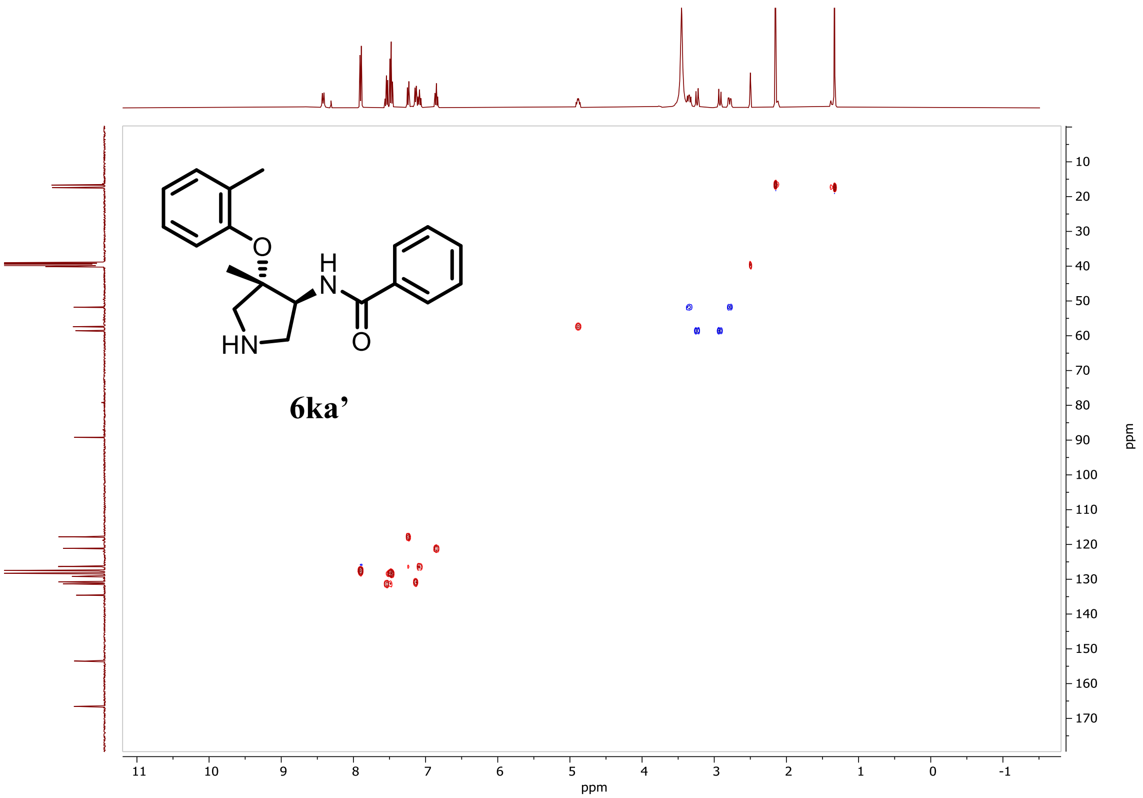

NOESY NMR spectrum (400 MHz, DMSO-d_6, 298 K) of compound **6ka’**

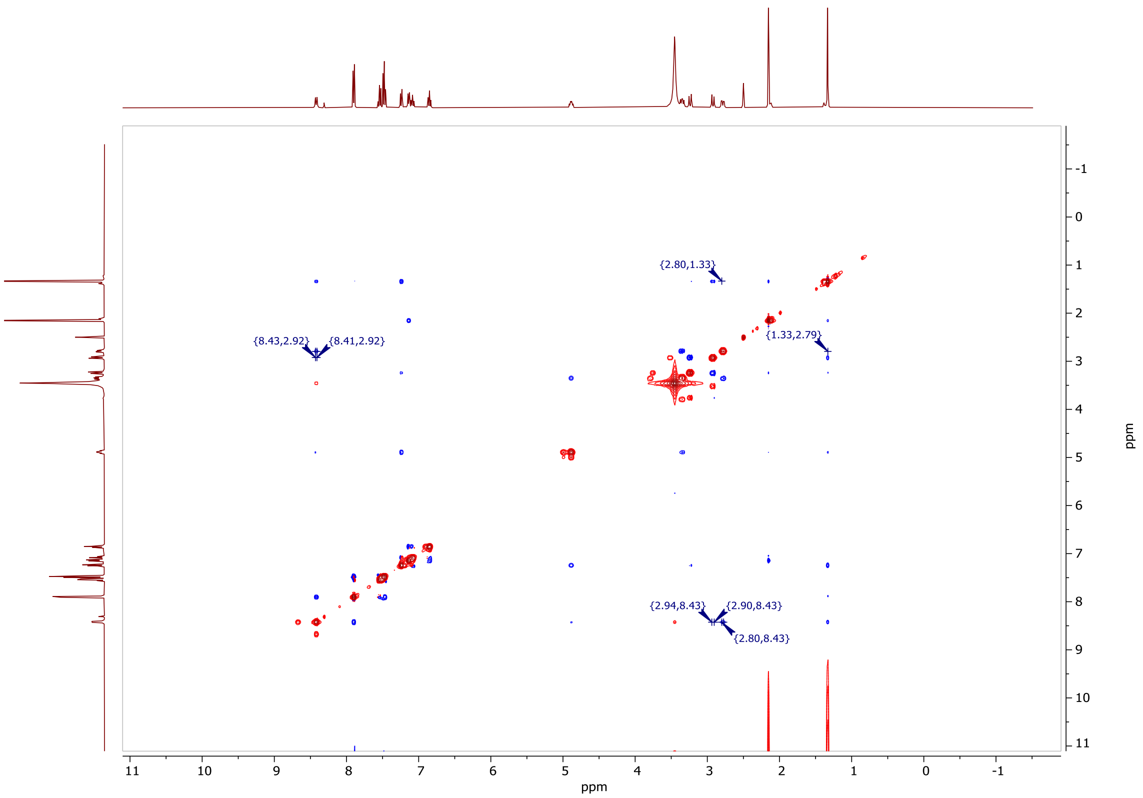

Analysis of the HSQC spectrum for **6ka’** confirms the nucleophilic attack of the aziridine ring by *o*-cresol occurred on the methyl-containing carbon resulting in the oxygen-bearing-quaternary carbon at δ_C 89.2 and the methyne correlation at δ_H 4.94–4.84 (m, 1H) to the nitrogen-bearing carbon at δ_C 57.4. NOESY correlations were used to confirm the relative stereochemistry of the pyrrolidine ring moiety. Through-space correlations between protons at δ_H 2.92 and δ_H 8.42, between protons at δ_H 8.42 and 2.79, and between protons at δ_H 2.79 and 1.33 indicate all three protons are on the same side of pyrrolidine ring. This observation indicates the *trans* relationships between *o*-cresol ether and benzamide moieties (Figure S3).

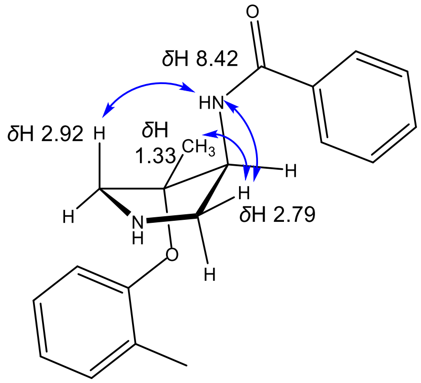

Figure S3. Key NOESY correlations for **6ka’**.

^{1}H NMR spectrum (400 MHz, $CDCl_3$, 298 K) of compound **6kb'**

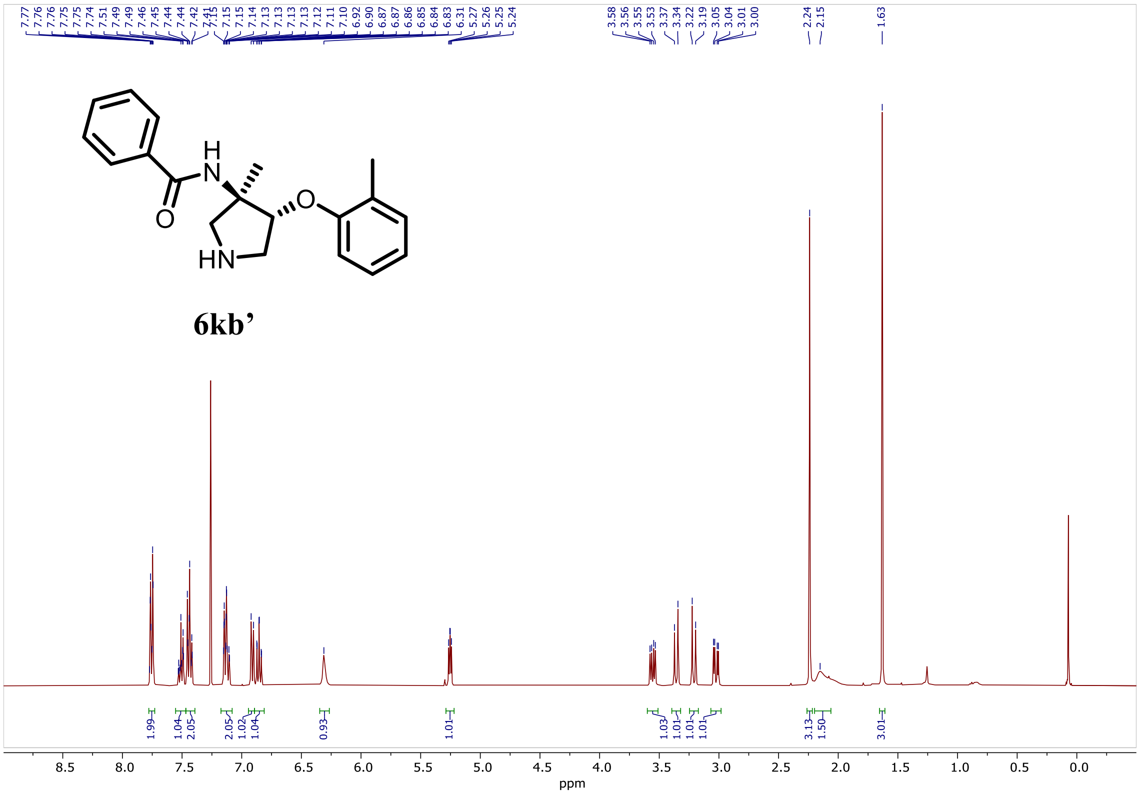

^{13}C{^{1}H} NMR spectrum (101 MHz, $CDCl_3$, 298 K) of compound **6kb'**

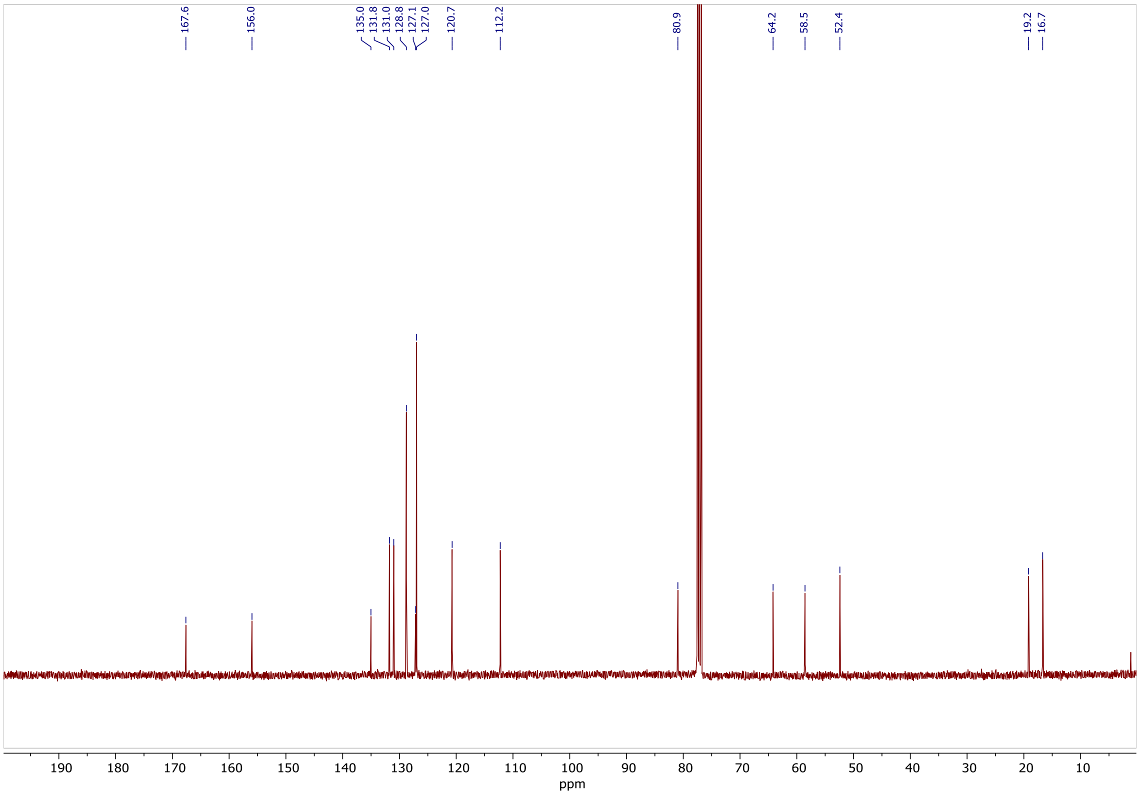

HSQC NMR spectrum (400 MHz, 101 MHz, $CDCl_3$, 298 K) of compound **6kb’**

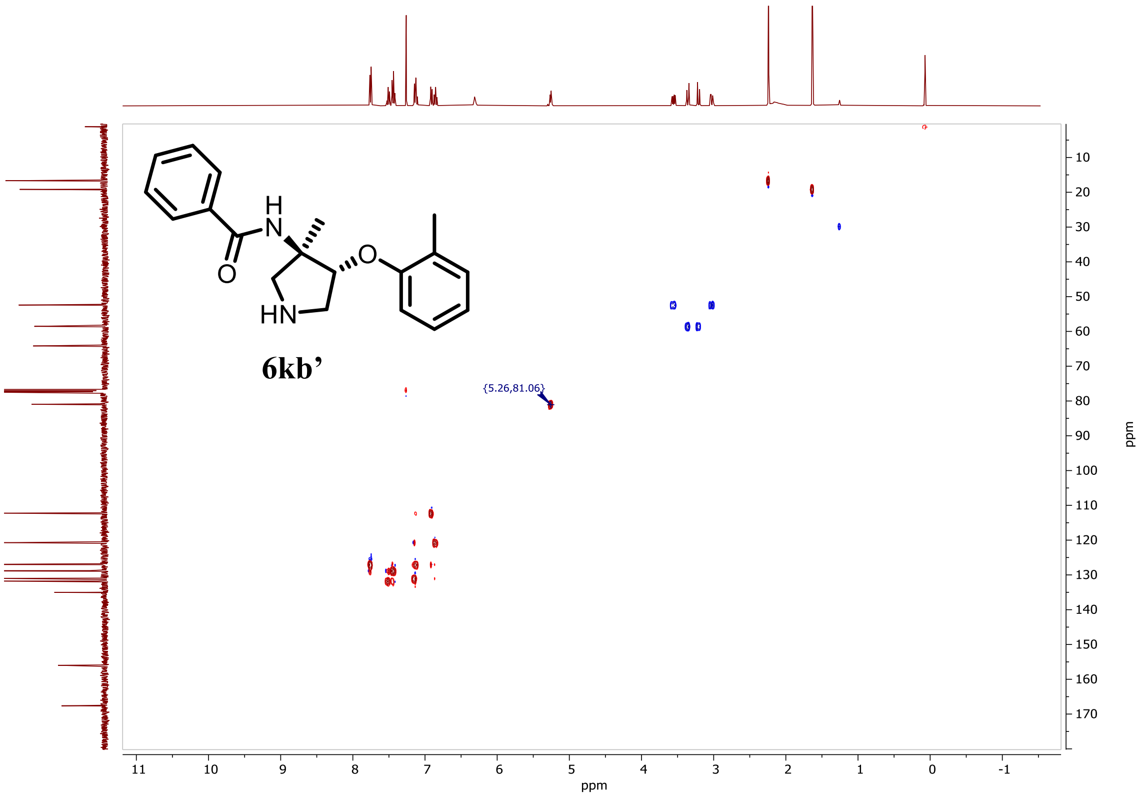

NOESY NMR spectrum (400 MHz, $CDCl_3$, 298 K) of compound **6kb’**

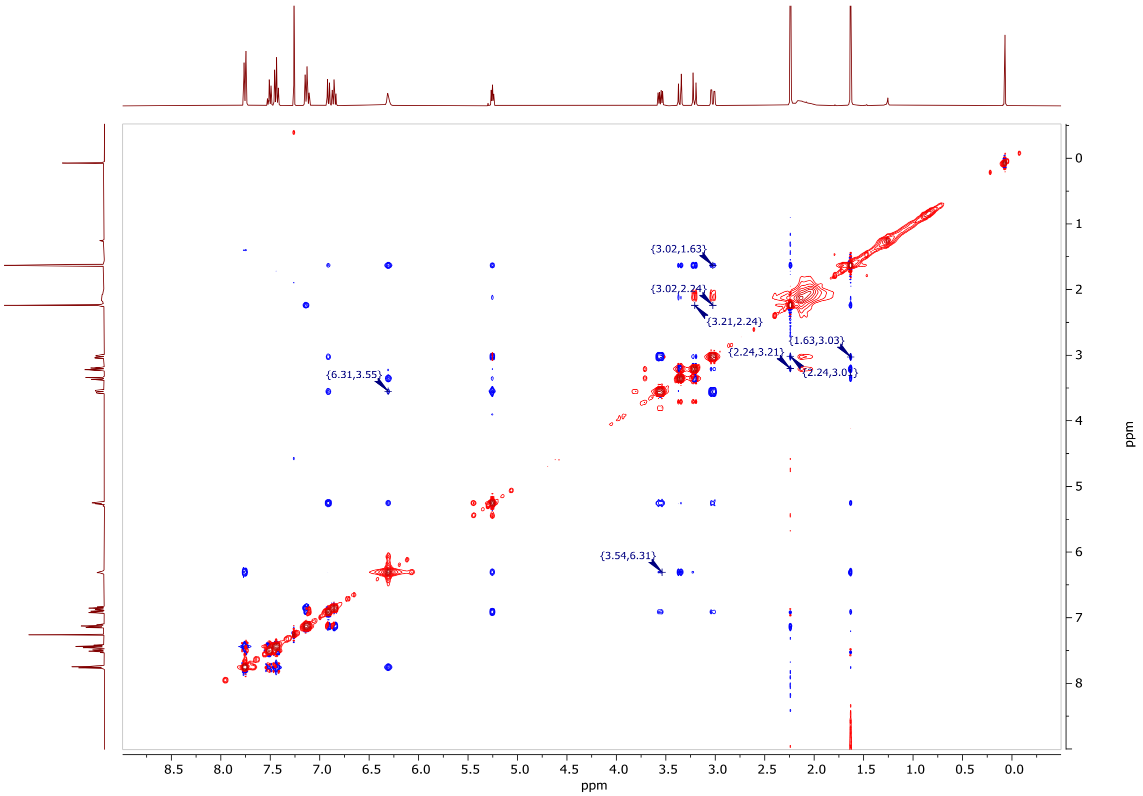

Analysis of the HSQC spectrum for **6kb'** confirms the oxygen-bearing methyne correlation at δ_H 5.25 (dd, J = 5.5, 4.0 Hz, 1H) to the carbon at δ_C 80.9 and methyl-bearing-quaternary carbon at δ_C 64.2. NOESY correlations were used to confirm the relative stereochemistry of the pyrrolidine ring moiety. Through-space correlations between protons at δ_H 6.31 and δ_H 3.56, between protons at δ_H 3.02 and 1.63, between protons at δ_H 3.21 and 2.24 and between protons at δ_H 3.02 and 2.24 indicate that the methyl group and the o-cresoloxy group are the same side of pyrrolidine ring whereas the benzamide group is the opposite side of the ring. This observation indicates the *trans* relationships between *o*-cresol ether and benzamide moieties (Figure S4).

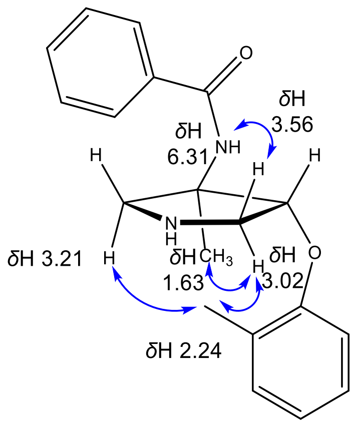

Figure S4. Key NOESY correlations for **6kb'**.

^{1}H NMR spectrum (400 MHz, $CDCl_3$, 298 K) of compound **6l**

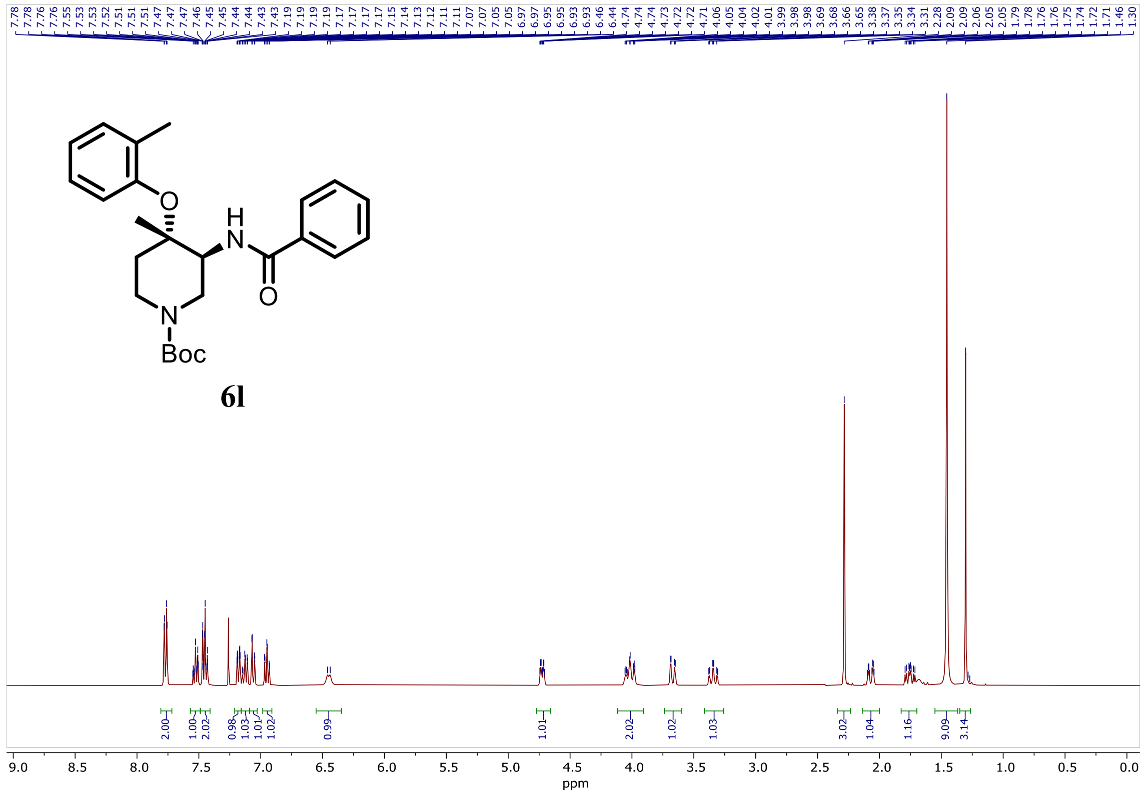

$^{13}C\{^{1}H\}$ NMR spectrum (101 MHz, $CDCl_3$, 298 K) of compound **6l**

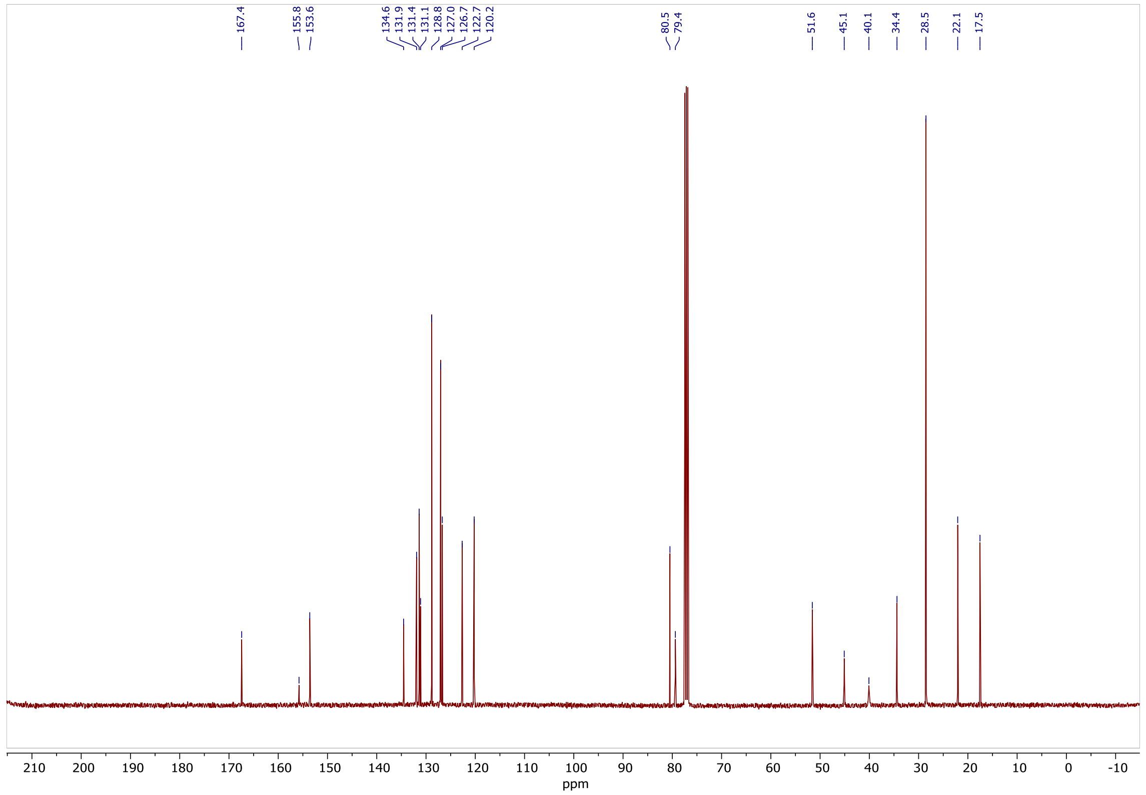

HSQC NMR spectrum (400 MHz, 101 MHz, $CDCl_3$, 298 K) of compound **6l**

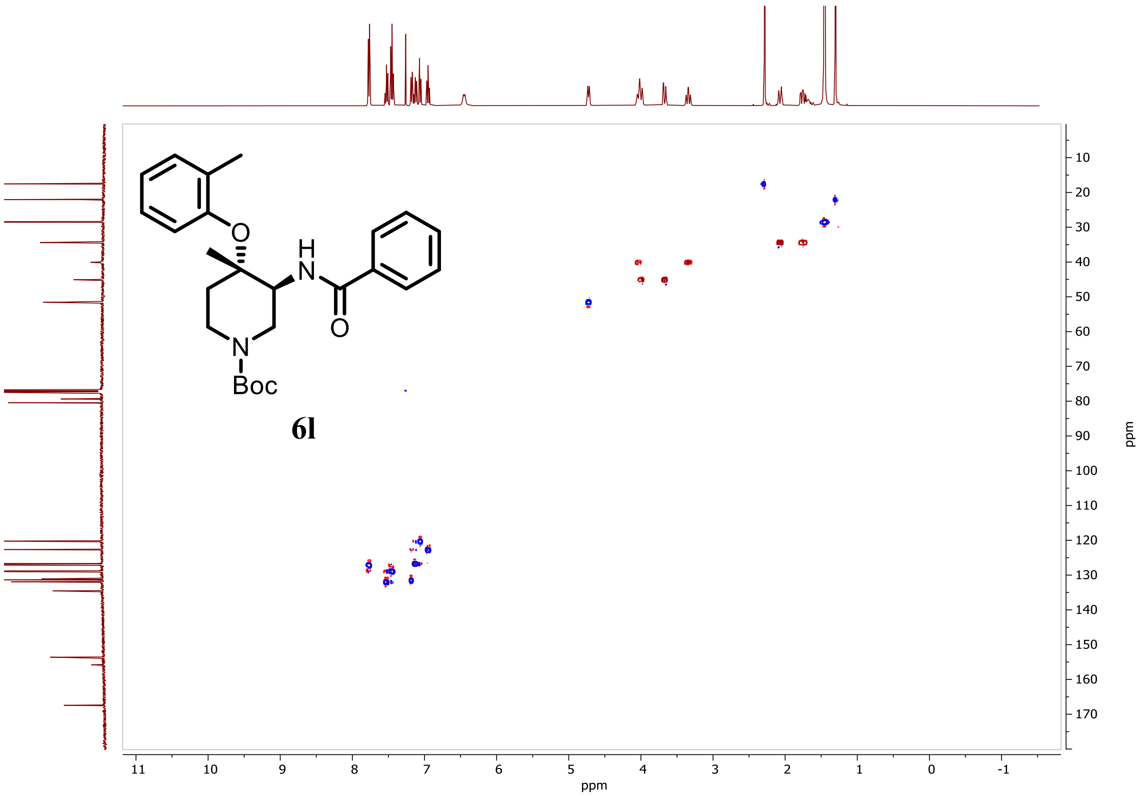

NOESY NMR spectrum (400 MHz, $CDCl_3$, 298 K) of compound **6l**

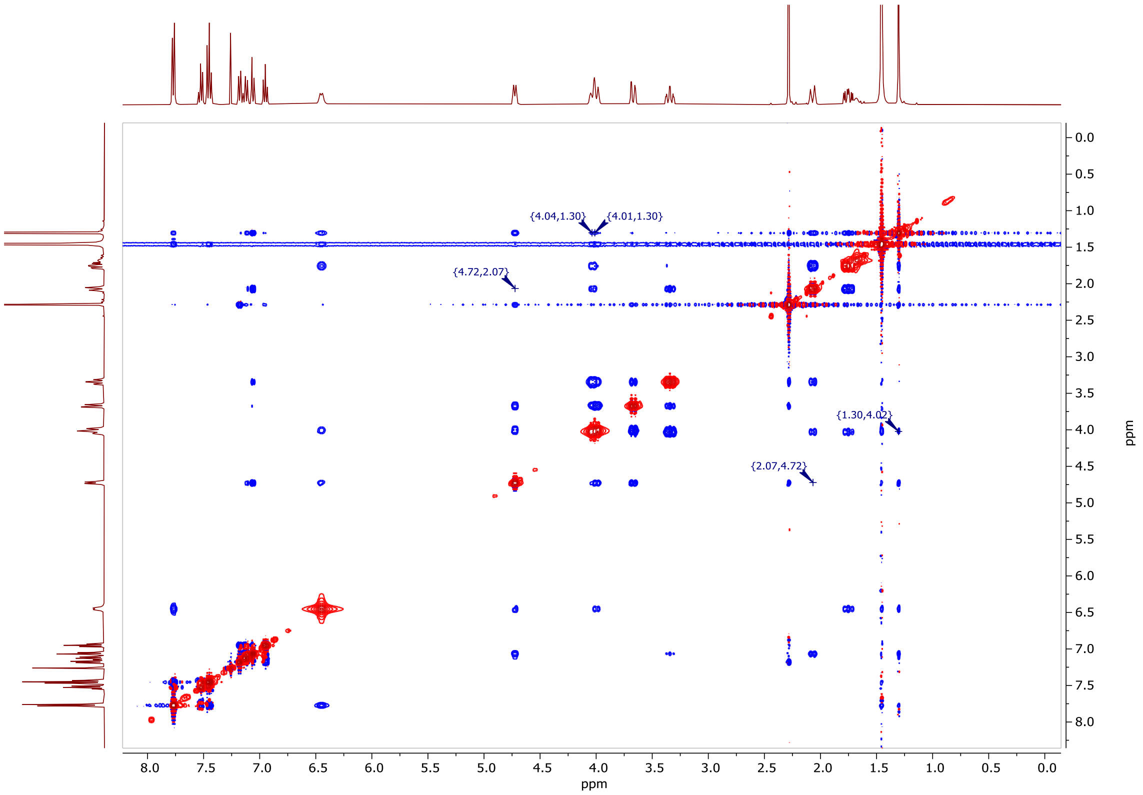

For **6l** attack at C-4 was confirmed by analyzing the HSQC spectrum. The nitrogen-bearing carbon at δ_C 51.6 (C-3) was observed as a methyne while the oxygen-bearing carbon at δ_C 79.4 (C-4) was observed as quaternary. The only way for C-4 to be quaternary is if the *o*-cresol attacked the carbon bearing the methyl group. The piperidine ring was characterized so the through-space correlations can be used to determine the relative configuration. C-3 is attached to the proton at δ_H 4.73 (brd, J = 8.9 Hz, 1H, H-3) and C-4 is attached to the methyl group at δ_H 1.30 (3H, s). C-2 was assigned to the carbon at δ_C 45.1 and is attached to two diastereotopic protons at δ_H 4.00 (m, 1H, H-2a) and δ_H 3.67 (dd, J = 13.5, 2.5 Hz, 1H, H-2b). The remaining methylene units in the HSQC at δ_C 34.4 and δ_C 40.1 were assigned to C-5 and C-6, respectively, based on the chemical shift of C-5 being more deshielded do to its attachment to the nitrogen of the piperidine ring. The two diastereotopic protons attached to C-5 are at δ_H 2.07 (brd, J = 14.5 Hz, 1H, H-5a) and δ_H 1.57 (ddd, J = 14.5, 12.4, 4.9 Hz, 1H, H-5b) and the two diastereotopic protons for C-6 are at δ_H 4.03 (m, 1H, H-6a) and δ_H 3.34 (m, 1H, H-2b). Through-space 1,3-diaxial correlations between the methyl at δ_H 1.30 and the two protons at δ_H 4.03 and δ_H 4.00 indicate all three are on the same side of the ring. Another 1,3-diaxial through-space correlation between δ_H 2.07 and δ_H 4.73 indicate that these two protons are on the opposite side of the ring from the previous three correlations. These observations confirm the *o*-cresol and benzamide moieties must be *trans* relative to one another (Figure S4).

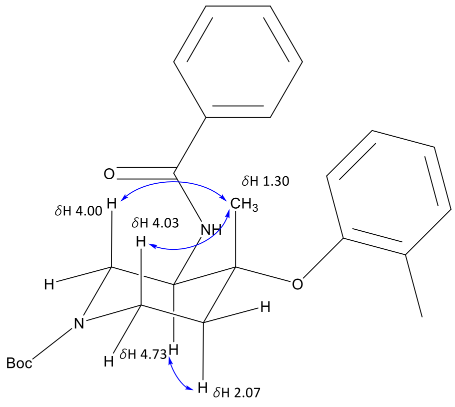

Figure S4. Key NOESY correlations for **6l**

^{1}H NMR spectrum (400 MHz, DMSO-d_6, 343 K) of compound **6ma**

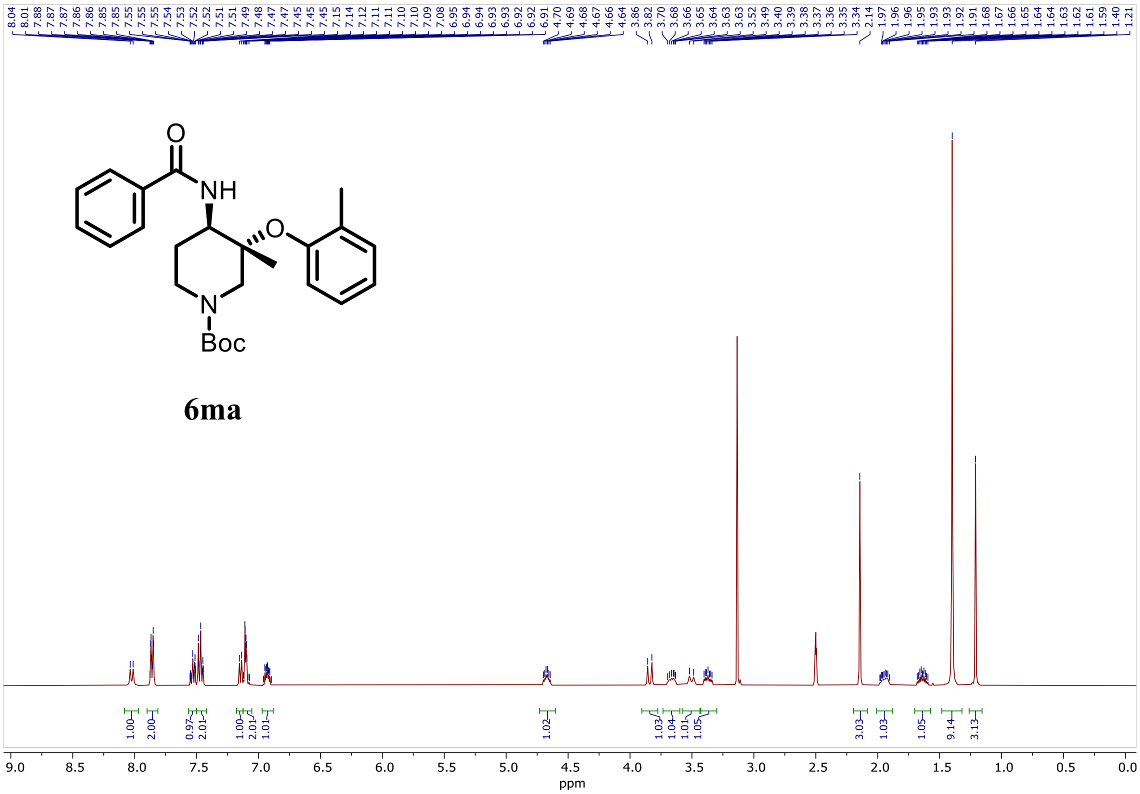

$^{13}C\{^{1}H\}$ NMR spectrum (101 MHz, DMSO-d_6, 343 K) of compound **6ma**

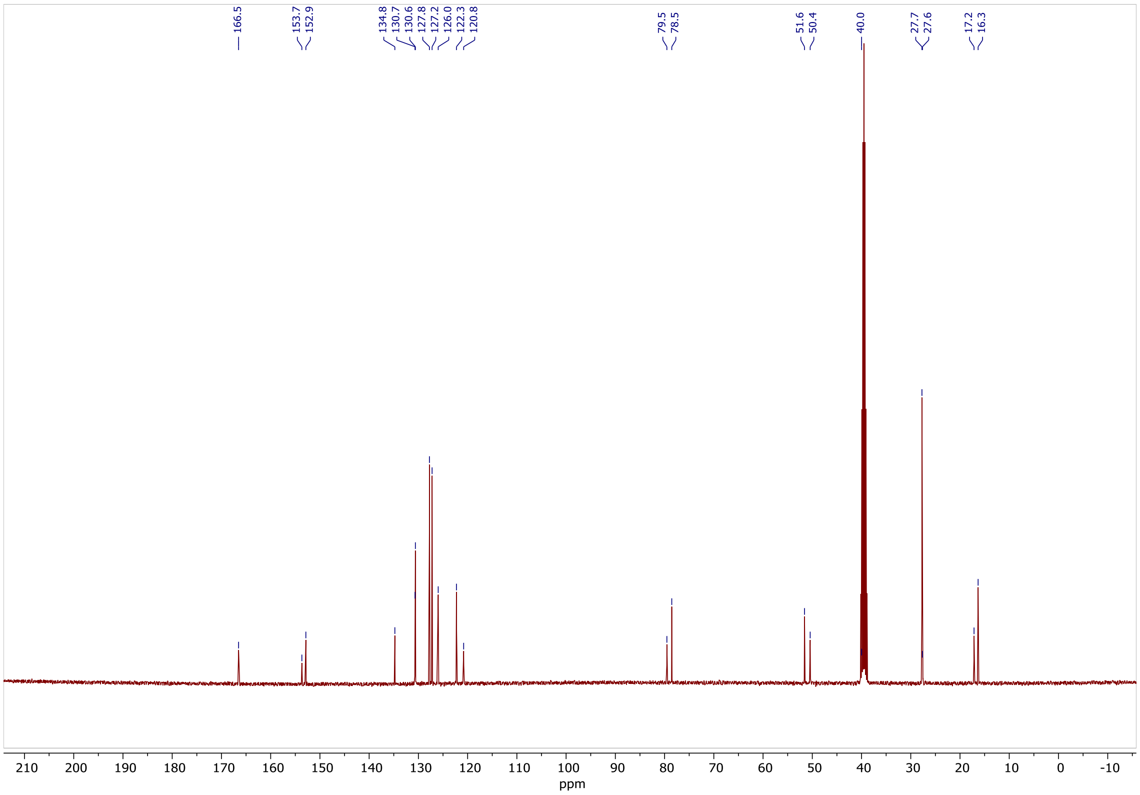

HSQC NMR spectrum (400 MHz, 101 MHz, DMSO-d_6, 343 K) of compound **6ma**

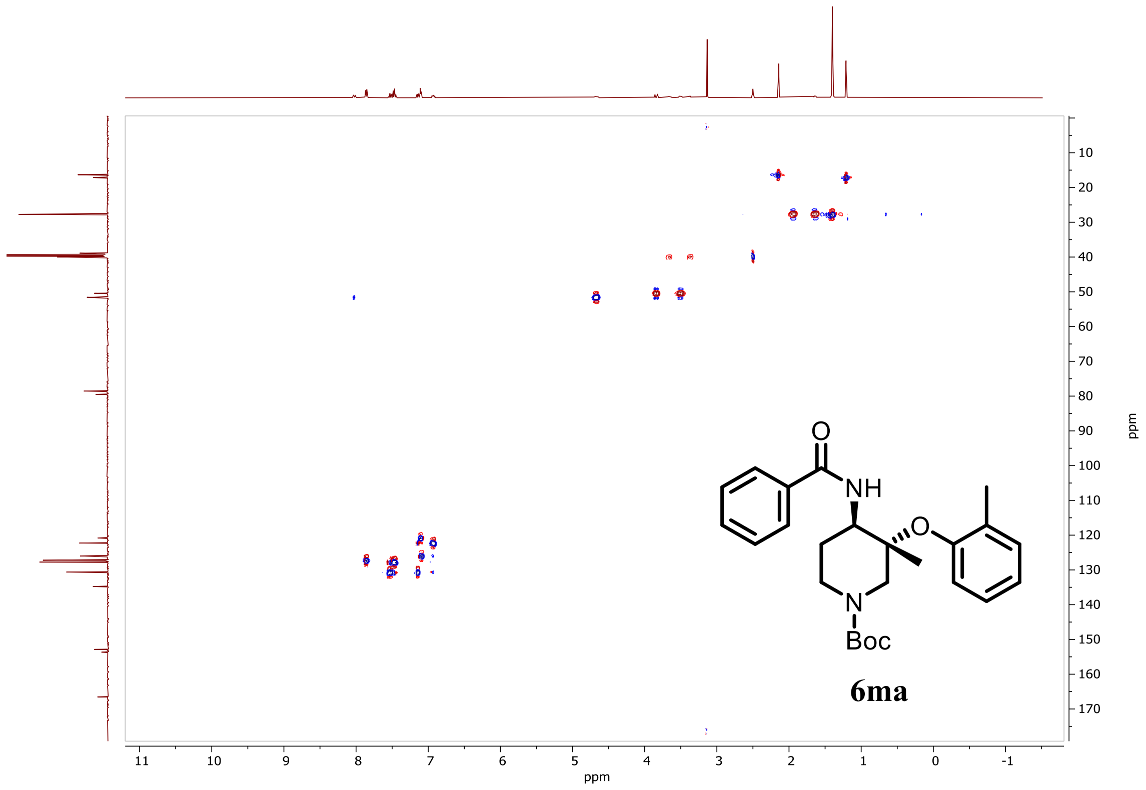

NOESY NMR spectrum (400 MHz, DMSO-d_6, 343 K) of compound **6ma**

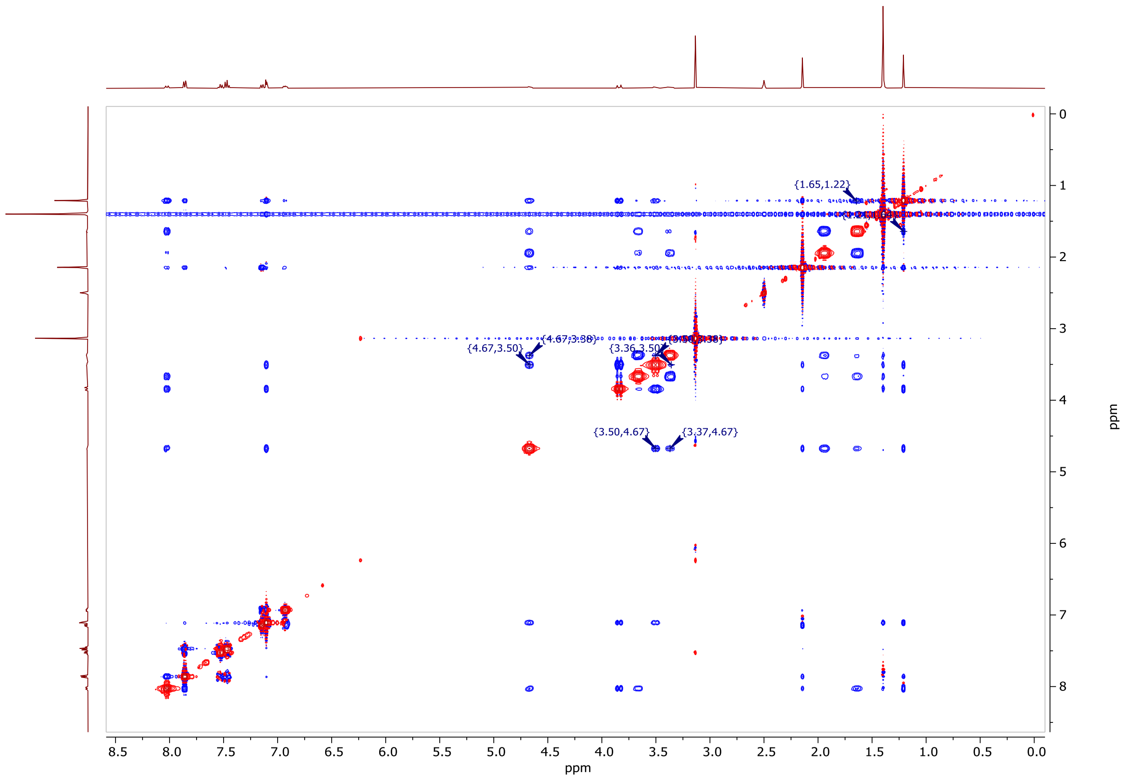

In order to assign the regiochemistry of **6ma** the piperidine ring was fully characterized by NMR. Analysis of the HSQC spectrum demonstrated that the carbon resonance at δ_C 50.4 (CH_2-2) was connected to the two diastereotopic protons with resonances at δ_H 3.84 (1H, d, J = 13.5 Hz, H-2a) and δ_H 3.50 (1H, d, J = 13.5 Hz, H-2b). The geminal couplings of these two protons indicates this methylene unit is at position 2 on the piperidine ring. Positions 3 and 4 on the piperidine ring were again deduced by interoperation of the HSQC spectrum. The methyne proton at δ_H 4.67 (1H, ddd, J = 8.7, 8.7, 4.4 Hz, H-4) was determined to be connected to the carbon resonance at δ_H 51.6 (CH-4), which identifies the methyne as a nitrogen-bearing carbon and not an oxygen-bearing carbon. The oxygen bearing carbon with a resonance at δ_C 78.5 (C-3) was determined to be a quaternary carbon by the HSQC spectrum. These observations indicate that position 3 must be the oxygen-bearing quaternary carbon due to the location of the methyl group in **5m**, and position 4 must be the nitrogen-bearing methyne unit. Positions 5 and 6 belong to the remaining methylene carbons in the HSQC spectrum and were assigned by comparing their chemical shifts. The more shielded carbon resonance at δ_C 27.6 (CH_2-5) with the two attached diastereotopic proton resonances at δ_H 1.95 (1H, m, H-5a) and δ_H 1.64 (1H, m, H-5b) was assigned to position 5 and the more deshielded carbon at δ_C 40.0 (CH_2-6) with its two diastereotopic proton resonances at δ_H 3.66 (1H, m, H-6a) and δ_H 3.37 (1H, ddd, J = 13.0, 8.2, 4.1 Hz, H-6b) was assigned as position 6 being the other nitrogen-bearing carbon on the piperidine ring.

NOESY correlations were used to confirm the relative stereochemistry of the piperidine ring moiety. Through-space 1,3-diaxial correlations between δ_H 3.50 (H-2b), δ_H 3.37 (H-6b), and δ_H 4.67 (H-4) indicate all three protons are on the same side of piperidine ring. Another 1,3-diaxial through-space correlation between the proton at δ_H 1.64 (H-5b) and the methyl group at δ_H 1.21 (3H, s) was also observed. This observation indicates the methyl group at δ_H 1.21 is attached to the piperidine ring and is on the same side of the ring as the proton at δ_H 1.64 (H-5b). Since δ_H 4.67 (H-4) and the methyl at δ_H 1.21 are on opposite sides of the ring then the *o*-cresol and benzamide moieties must be *trans* (Figure S5).

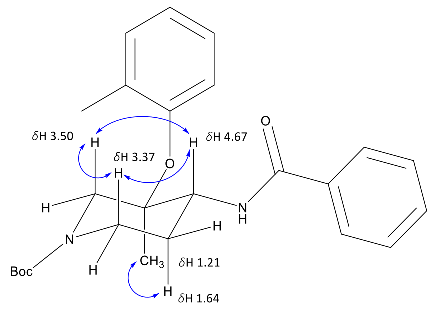

Figure S5. Key NOESY correlations for **6ma**.

^{1}H NMR spectrum (400 MHz, DMSO-d_6, 343 K) of compound **6mb**

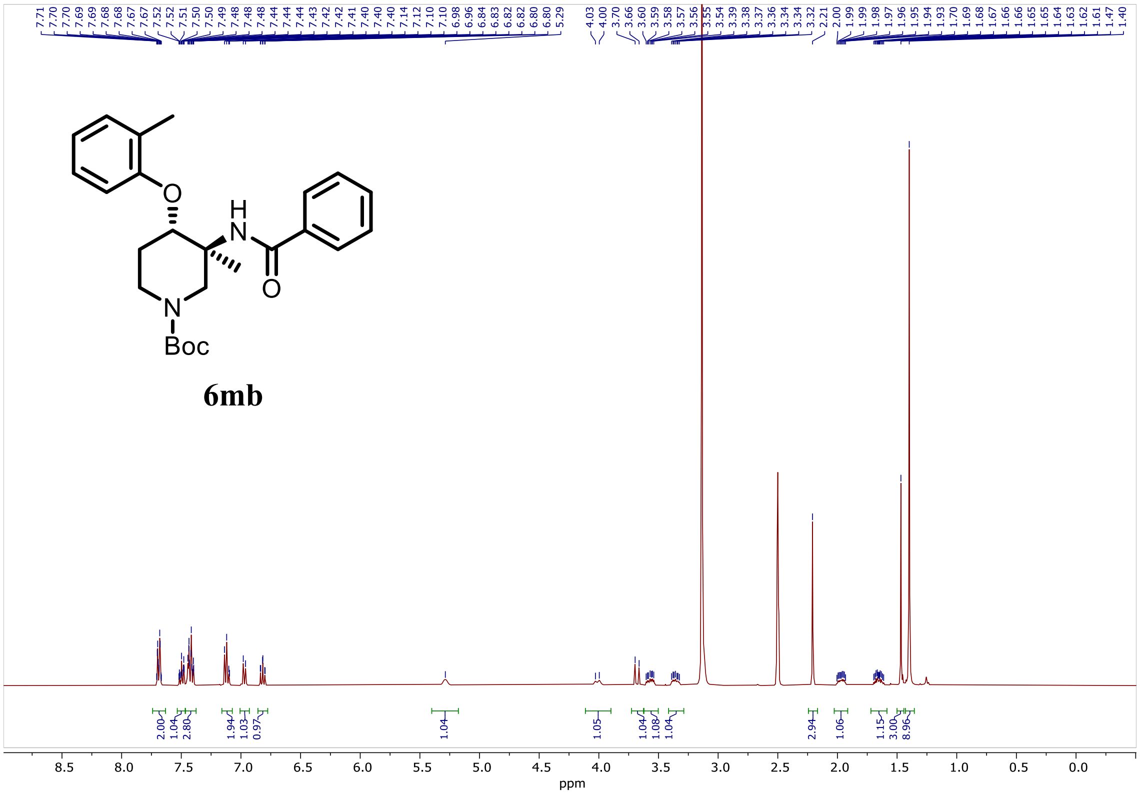

^{13}C{^{1}H} NMR spectrum (101 MHz, DMSO-d6, 343 K) of compound **6mb**

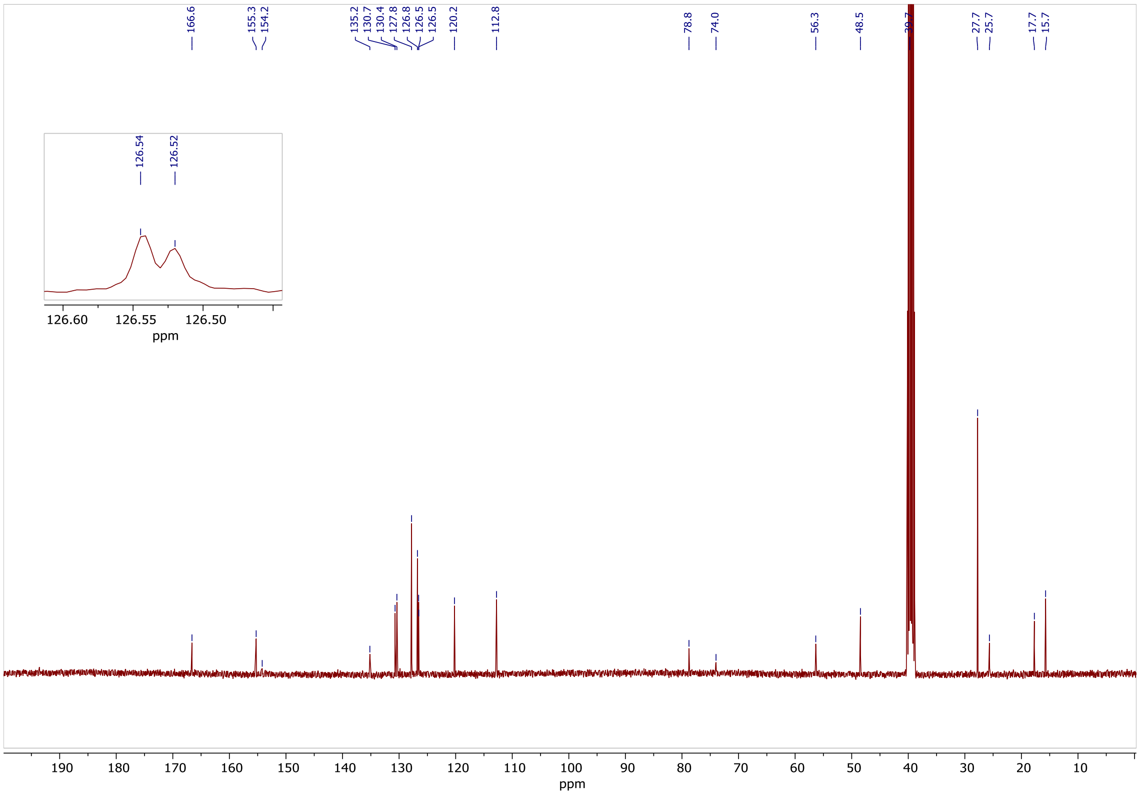

HSQC NMR spectrum (400 MHz, 101 MHz, DMSO-d_6, 343 K) of compound **6mb**

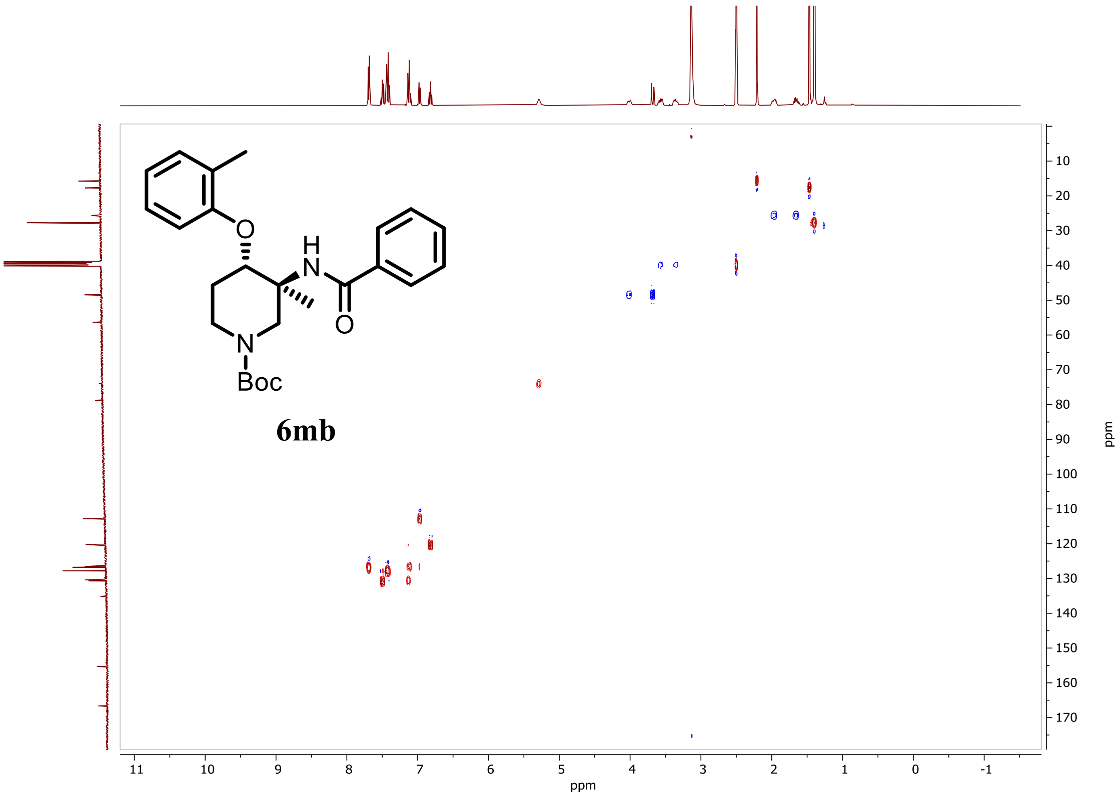

NOESY NMR spectrum (400 MHz, DMSO-d_6, 343 K) of compound **6mb**

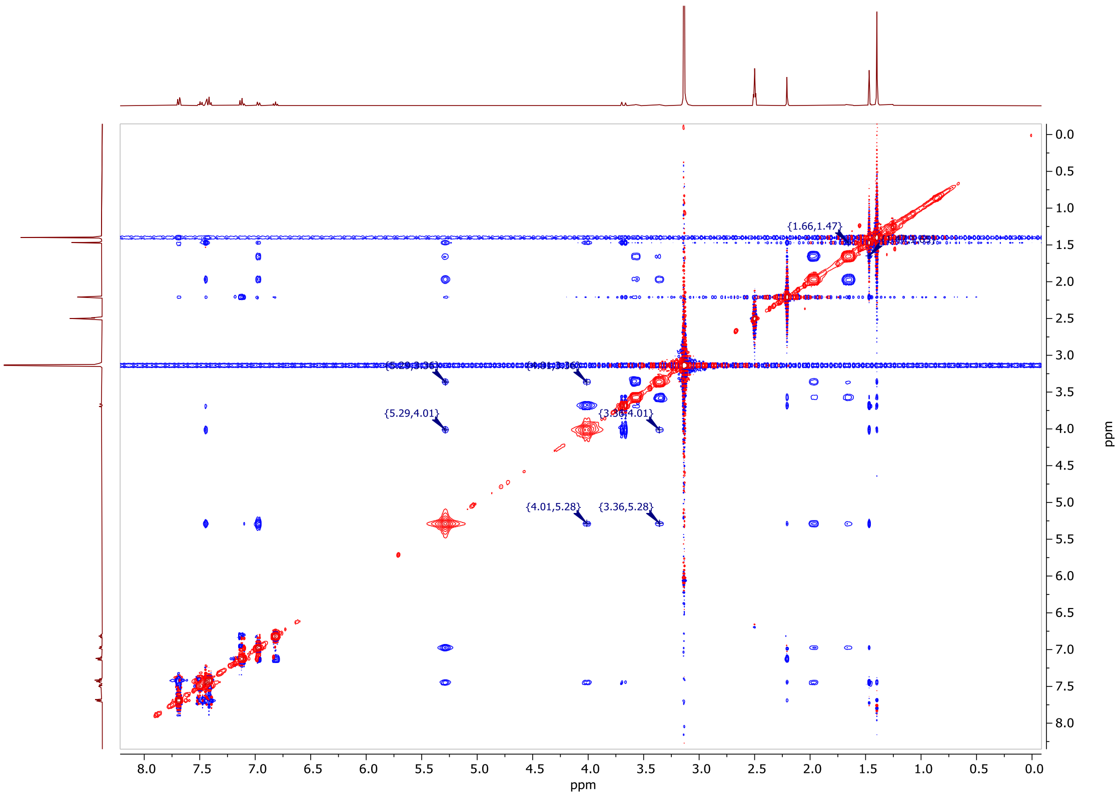

Analysis of the HSQC spectrum for the minor product, **6mb**, demonstrated that the nitrogen-bearing carbon at δ_C 56.3 (C-3) is quaternary and the oxygen-bearing carbon at δ_C 74.0 (CH-4) is a methyne, the opposite of the major product. Through-space 1,3-diaxial NOESY correlations between δ_H 4.01 (d, J = 12.8 Hz, 1H, H-2a), δ_H 3.36 (ddd, J = 13.4, 8.2, 4.8 Hz, 1H, H-6b), and δ_H 5.29 (m, 1H, H-4) indicate all three protons are on the same side of piperidine ring. Another 1,3-diaxial through-space correlation between the proton at δ_H 1.65 (m, 1H, H-5b) and the methyl group at δ_H 1.47 (3H, s) was also observed. These through-space correlations all suggest the *o*-cresol and benzamide moieties are *trans* (Figure S6).

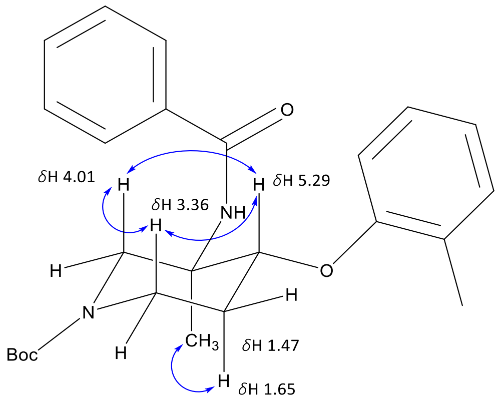

Figure S6. Key NOESY correlations for **6mb**.

^{1}H NMR spectrum (400 MHz, $CDCl_3$, 298 K) of compound **d**

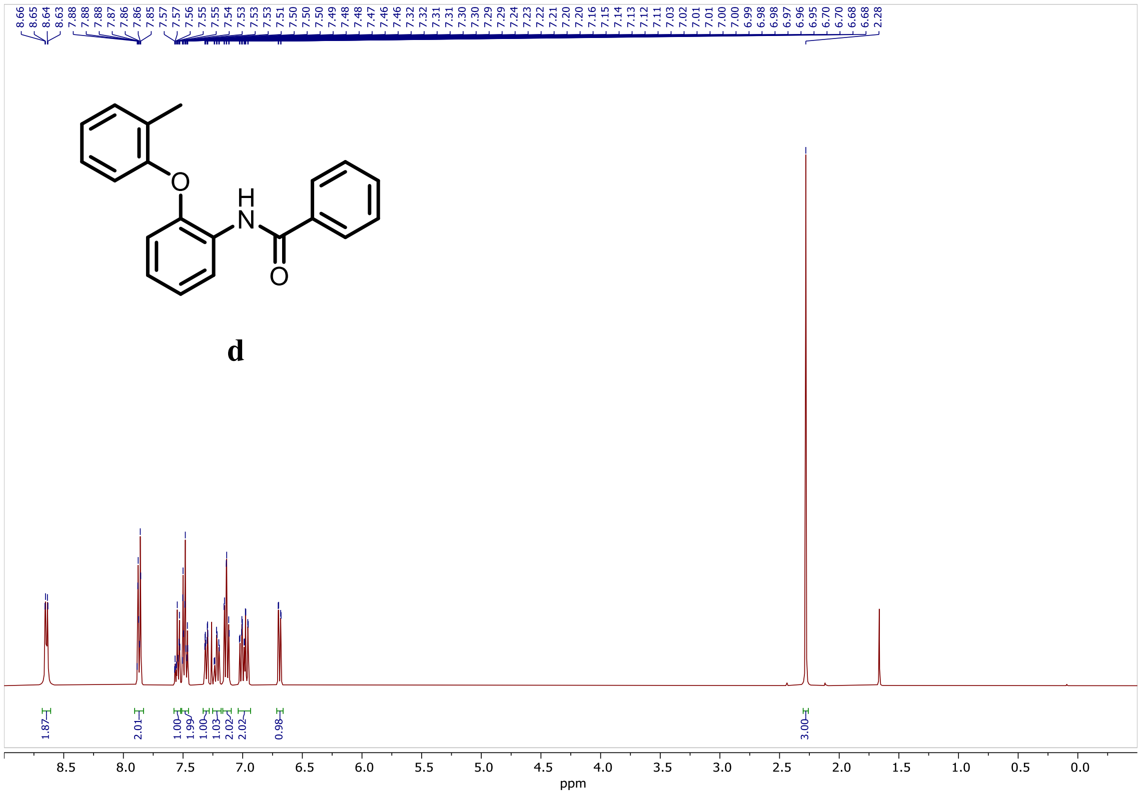

$^{13}C\{^{1}H\}$ NMR spectrum (101 MHz, $CDCl_3$, 298 K) of compound **d**

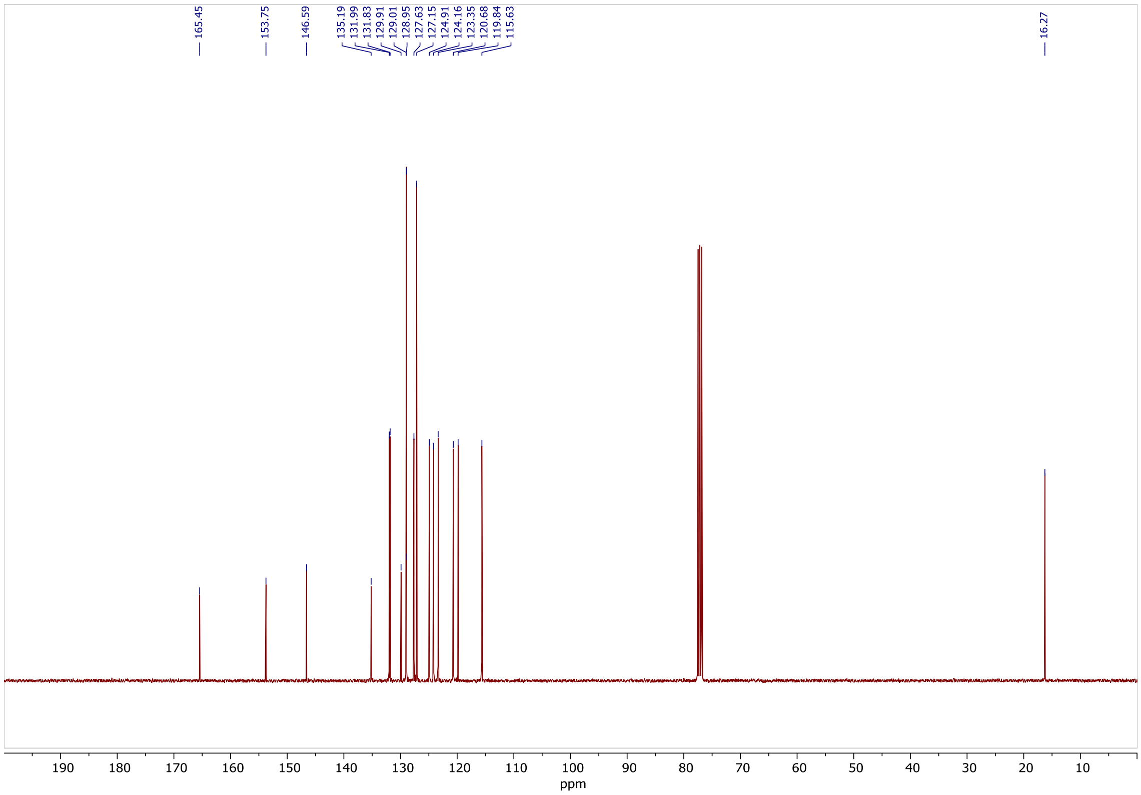

8. X ray crystallographic data

Details of crystalline growing conditions

A crystal of **2a** was obtained by slow evaporation of acetone solution of **2a**.

Each crystal of **5m** and **7** were obtained by slow evaporation of solvent A into a solution of solvent B in the following table;

compound	solvent A	solvent B
**5m**	Hexane	Et_2O
**7**	Et_2O	THF

Table S2. Crystalline growing solvent

Details of crystallographic refinement

General Methods. A suitable crystal of each sample was selected for analysis and mounted in a polyimide loop. Crystal samples were handled under immersion oil and quickly transferred to a cold nitrogen stream. All measurements were made on a Rigaku Oxford Diffraction Supernova Eos CCD with filtered Cu-Kα radiation at a temperature of 100 K. Using Olex2[4], the structure was solved with the ShelXT structure solution program using Direct Methods and refined with the ShelXL refinement package[5] using Least Squares minimization.

Compounds **2a** and **7** were refined without additional restraints.

Compound **5m**

The structure was refined without additional restraint. The refined Flack and Hoof parameters are 0.2(2) and 0.19(7) respectively. Refinement with a racemic twin law (0.8:0.2) did not improve the R value, but since the crystals were obtained from a racemic mixture the enantiopurity of the crystals cannot be confidently assigned.

Table S3. Crystal data and structure refinement for **2a**.

Empirical formula	$C_{23}H_{28}N_2O_4$	
Formula weight	396.47	
Temperature	100.01(10) K	
Wavelength	1.54184 Å	
Crystal system	Triclinic	
Space group	P-1	
Unit cell dimensions	a = 10.0222(3) Å	α = 95.076(2)°
	b = 10.3905(3) Å	β = 107.770(3)°
	c = 11.1743(4) Å	γ = 109.055(3)°
Volume	1024.28(6) $Å^3$	
Z	2	
Density (calculated)	1.286 Mg/m^3	
Absorption coefficient	0.712 mm^{-1}	
F(000)	424	
Crystal size	0.157 x 0.142 x 0.082 mm^3	
Theta range for data collection	4.249 to 71.771°.	
Index ranges	-12<=h<=12, -12<=k<=12, -13<=l<=13	
Reflections collected	20480	
Independent reflections	3952 [R(int) = 0.0367]	
Completeness to theta = 67.684°	99.9 %	
Absorption correction	Gaussian	
Max. and min. transmission	1.000 and 0.817	
Refinement method	Full-matrix least-squares on F^2	
Data / restraints / parameters	3952 / 0 / 270	
Goodness-of-fit on F^2	1.043	
Final R indices [I>2sigma(I)]	R1 = 0.0334, wR2 = 0.0827	
R indices (all data)	R1 = 0.0366, wR2 = 0.0849	
Largest diff. peak and hole	0.292 and -0.226 $e/Å^{-3}$	

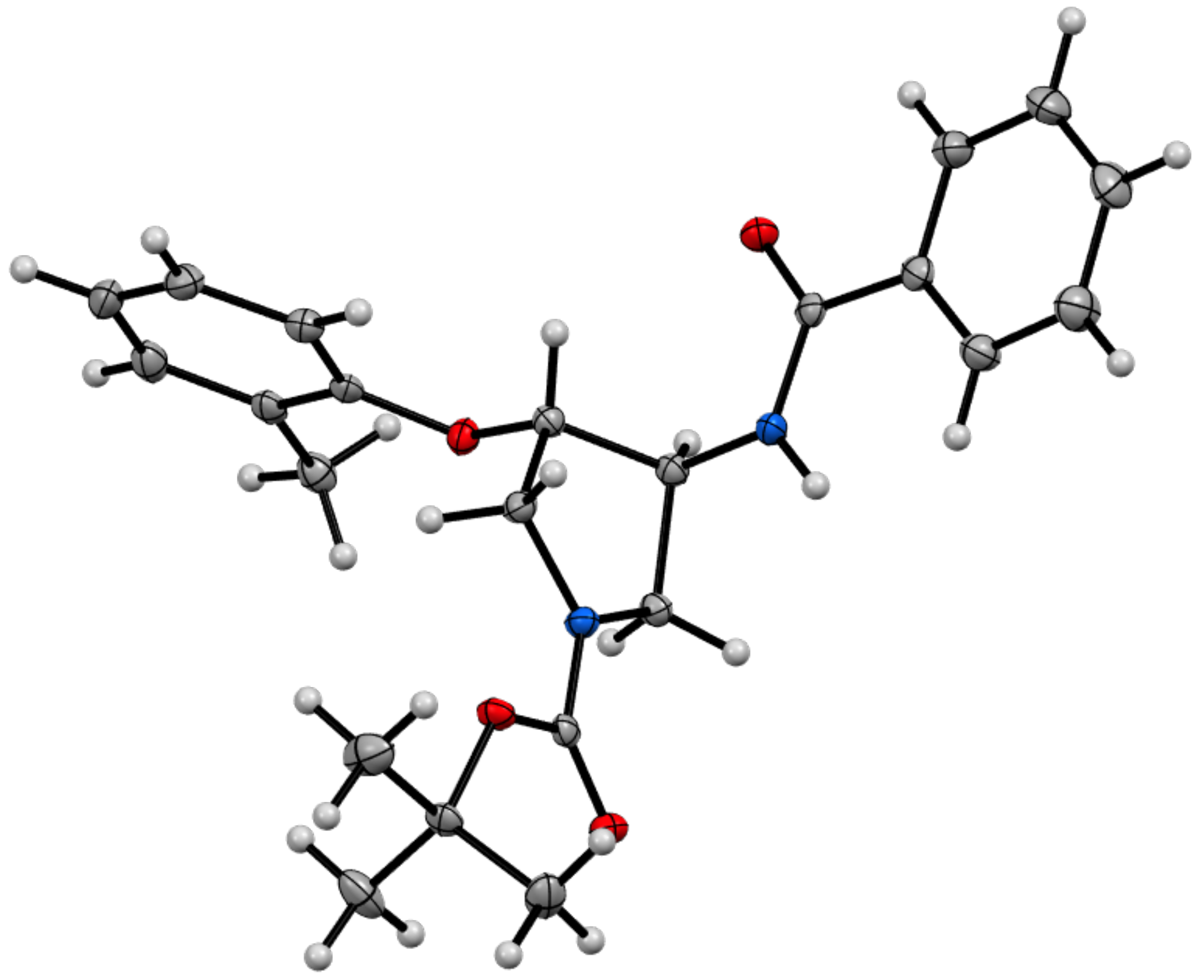

Figure S7. ORTEP of **2a**. Ellipsoids shown at 50% probability.

Table S4. Crystal data and structure refinement for **5m**.

Empirical formula	$C_{18}H_{24}N_2O_3$	
Formula weight	316.39	
Temperature	100.00(10) K	
Wavelength	1.54184 Å	
Crystal system	Orthorhombic	
Space group	$P2_12_12_1$	
Unit cell dimensions	a = 6.01420(10) Å	α = 90°
	b = 12.25730(10) Å	β = 90°
	c = 22.5986(2) Å	γ = 90°
Volume	1665.92(3) Å^3	
Z	4	
Density (calculated)	1.261 Mg/m^3	
Absorption coefficient	0.694 mm^{-1}	
F(000)	680	
Crystal size	0.51 x 0.05 x 0.03 mm^3	
Theta range for data collection	3.912 to 71.758°.	
Index ranges	-7<=h<=6, -15<=k<=15, -27<=l<=27	
Reflections collected	16367	
Independent reflections	3239 [R(int) = 0.0377]	
Completeness to theta = 67.684°	100.0 %	
Absorption correction	Gaussian	
Max. and min. transmission	1.000 and 0.666	
Refinement method	Full-matrix least-squares on F^2	
Data / restraints / parameters	3239 / 0 / 212	
Goodness-of-fit on F^2	1.047	
Final R indices [I>2sigma(I)]	R1 = 0.0275, wR2 = 0.0679	
R indices (all data)	R1 = 0.0289, wR2 = 0.0691	
Absolute structure parameter	0.18(8)	

Largest diff. peak and hole	0.156 and -0.166 e/Å^{-3}

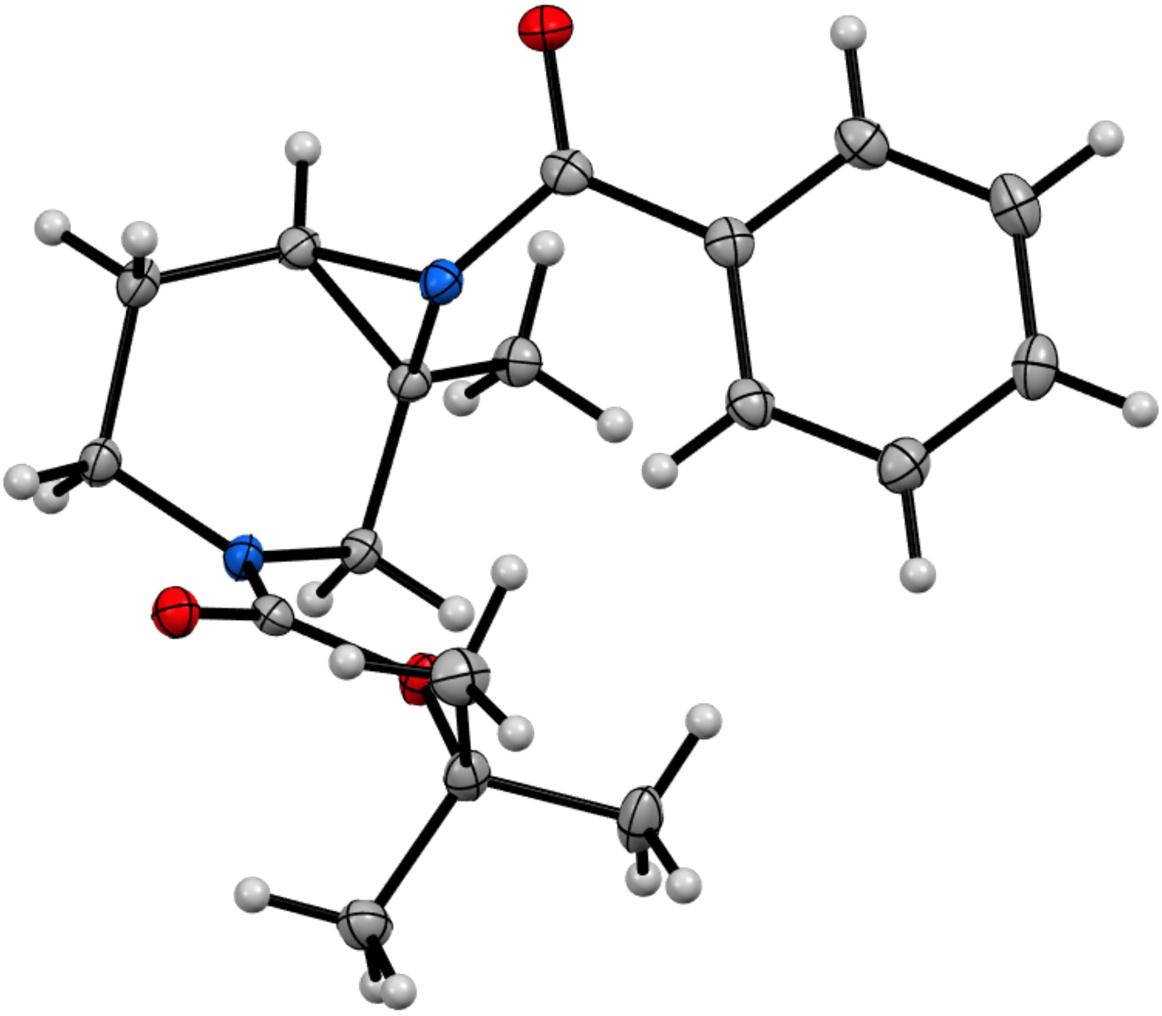

Figure S8. ORTEP of **5m**. Ellipsoids shown at 50% probability.

Table S5. Crystal data and structure refinement for **7**.

Empirical formula	$C_{20}H_{18}N_4O_6$	
Formula weight	410.38	
Temperature	100.00(10) K	
Wavelength	1.54184 Å	
Crystal system	Monoclinic	
Space group	P 1 21/c 1	
Unit cell dimensions	a = 15.2807(2) Å	α = 90°
	b = 13.8656(2) Å	β = 96.7490(10)°
	c = 8.87250(10) Å	γ = 90°
Volume	1866.84(4) $Å^3$	
Z	4	
Density (calculated)	1.460 Mg/m^3	
Absorption coefficient	0.927 mm^{-1}	
F(000)	856	
Crystal size	0.17 x 0.15 x 0.14 mm^3	
Theta range for data collection	2.912 to 71.813°.	
Index ranges	-18<=h<=18, -17<=k<=16, -10<=l<=8	
Reflections collected	16908	
Independent reflections	3630 [R(int) = 0.0317]	
Completeness to theta = 67.684°	100.0 %	
Absorption correction	Gaussian	
Max. and min. transmission	1.000 and 0.722	
Refinement method	Full-matrix least-squares on F^2	
Data / restraints / parameters	3630 / 0 / 272	
Goodness-of-fit on F^2	1.025	
Final R indices [I>2sigma(I)]	R1 = 0.0348, wR2 = 0.0896	
R indices (all data)	R1 = 0.0380, wR2 = 0.0920	
Largest diff. peak and hole	0.242 and -0.187 e/$Å^{-3}$	

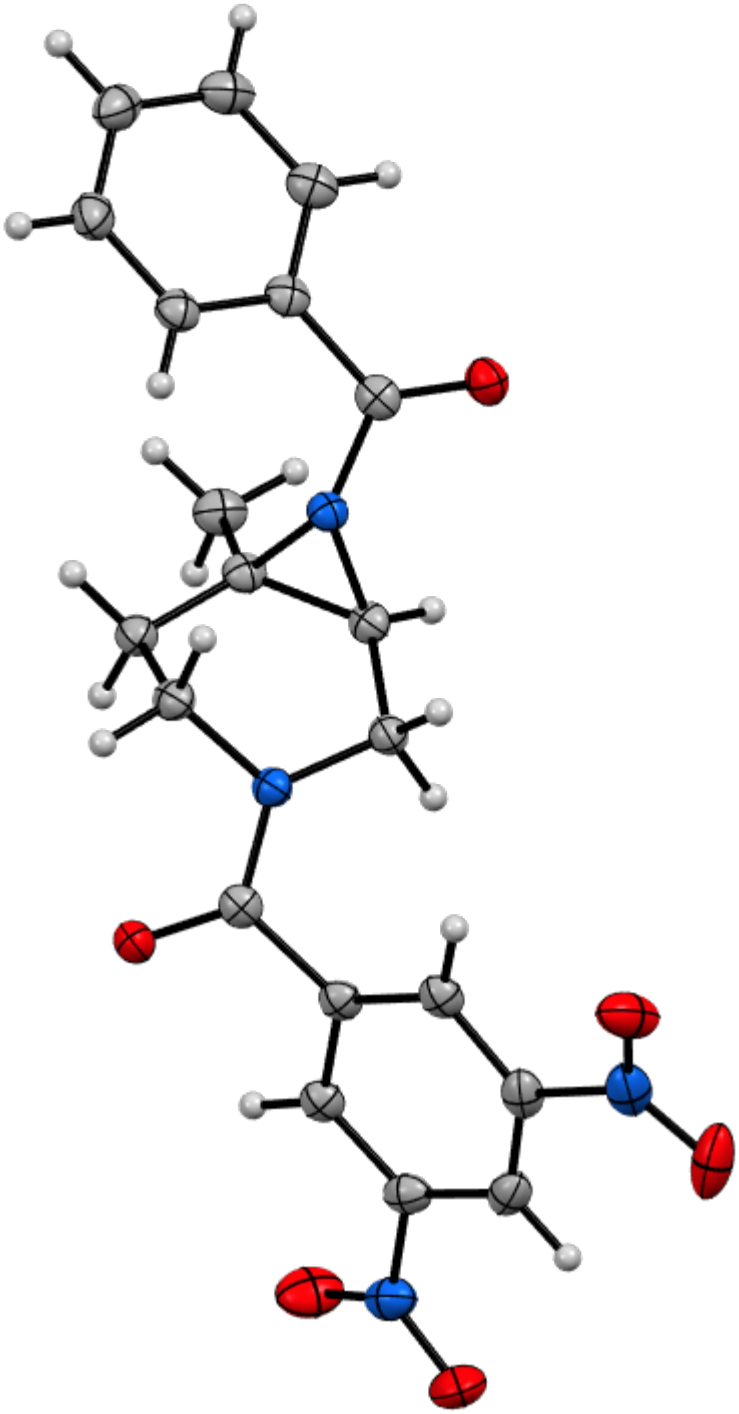

Figure S9. ORTEP of **7**. Ellipsoids shown at 50% probability.

9. Physicochemical property calculation for generated library

A 3D plot was generated based on the following structure and calculated property values. Clog P value was calculated with ChemDraw 20.1.

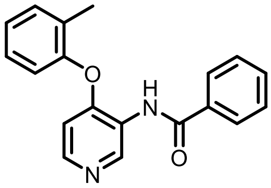
a

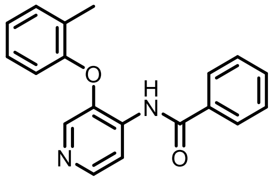
b

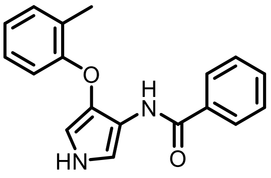
c

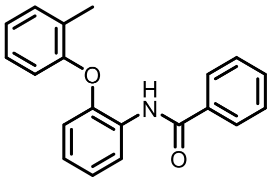
d

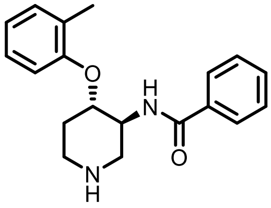
e

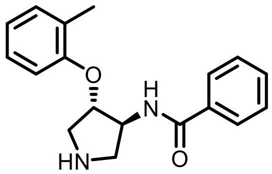
f

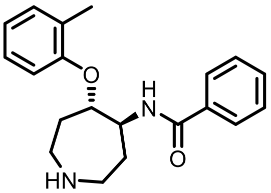
g

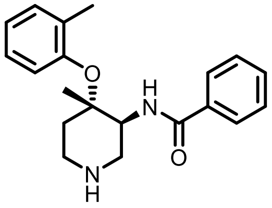
h

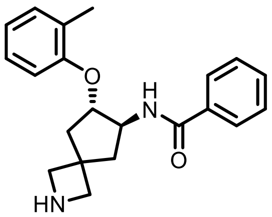
i

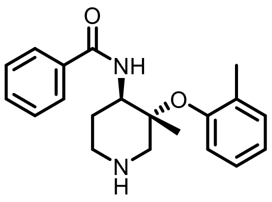
j

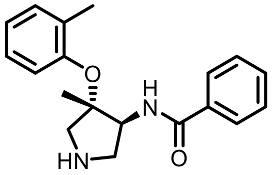
k

Compound	Classification	Molecular Weight	ClogP	Fsp^3
a	aromatic ring core	304.35	3.988	0.0526
b	aromatic ring core	304.35	3.988	0.0526
c	aromatic ring core	292.34	3.263	0.0556
d	aromatic ring core	303.36	4.657	0.0500
e	saturated ring core	310.40	3.163	0.3158
f	saturated ring core	296.37	3.151	0.2778
g	saturated ring core	324.42	2.898	0.3500
h	saturated ring core	324.42	3.682	0.3500
i	saturated ring core	336.44	2.653	0.3810
j	saturated ring core	324.42	3.406	0.3500
K	saturated ring core	310.40	3.670	0.3158

Table S6. Calculated physicochemical properties.

10. Kinetic Solubility assay

Kinetic Solubility assay protocol

Adapted standard shake flask method to run in 1 mL 96-deep-well plates at a concentration of 100 µM in McIlvaine Buffer at pH values of 6.8 from 10 mM DMSO stock solutions. Compounds are prepared in triplicate and incubated in buffer at room temperature for 18 hours while shaking at 700 RPM. After incubation the 96-deep-well plate is centrifuged at 5000 *g* for 10 minutes, half of the volume is transferred to another deep well plate and centrifuged again at 5000 *g* for 10 minutes. 200 µL are transferred from each well to a Greiner Bio-one 200 µL 96-well V-bottom plate and sealed. A six-point calibration curve is prepared for each compound ranging from 100 µM down to 0.5 µM. All samples are analyzed via UV-UHPLC on an Agilent 1290 Infinity (binary pump, auto-sampler, column compartment at 55 ˚C, and PDA) with a Phenomenex Kinetex EVO C18, 50 x 1 mm, 1.7 µm, 100 Å column, at 0.5 mL. Injections of 3 µL are analyzed with gradient elution using Milli-Q water with 0.05% trifluoroacetic acid (A1) and acetonitrile with 0.05% trifluoroacetic acid (B1) from 95:5 A1/B1 to 5:95 A1/B1 over 1.3 minutes with a 0.2 minute hold at 5:95 A1/B1. Wavelengths at 215 nm and 254 nm are monitored, peaks are integrated, and the peak area and peak height are used with linear regression analysis from the calibration curves to determine the solubility values.[6]

Kinetic Solubility assay data

A kinetic solubility for **d**, **e**, **f,** and **g** was calculated in the following Table.

Compound	Kinetic solubility (µM, pH=6.8)
d	5.74 ± 0.03
e	90.67 ± 0.32
f	79.07 ± 0.44
g	92.17 ± 0.72

Table S7. Kinetic solubility for representative compounds.

11. References

[1] C. G. Espino, K. W. Fiori, M. Kim, J. Du Bois, *J. Am. Chem. Soc.* **2004**, *126*, 15378–15379.

[2] Z. Ma, Z. Zhou, L. Kürti, *Angew. Chem. Int. Ed.* **2017**, *56*, 9886-9890.

[3] G. J. Sośnicki, *Tetrahedron Lett.* **2009**, *50*, 178-181.

[4] O. V. Dolomanov, L. J. Bourhis, R. J. Gildea, J. A. K. Howard, H. Puschmann, *J. Appl. Crystallogr.* **2009**, *42*, 339-341.

[5] S. G.M., *Acta. Crystallogr. A.* **2008**, *64*, 112-122.

[6] a) B. Lin, H. J. Pease, *J. Pharm. Biomed. Anal.* **2016**, *122*, 126-140; b) T. Yamashita, Y. Dohta, T. Nakamura, T. Fukami, *J. Chromatogr. A.* **2008**, *1182*, 72-76; c) McIlvaine, T. C., *J. Biol. Chem.* **1921**, *49*, 183-186.
